# Aberrations in medically certified sick leave and primary healthcare consultations in Norway in 2023 compared to pre-COVID-19-pandemic trends

**DOI:** 10.1186/s13690-024-01411-4

**Published:** 2024-10-22

**Authors:** Richard Aubrey White, Chi Zhang, Beatriz Valcarcel Salamanca, Aslaug Angelsen, Dinastry Pramadita Zakiudin, Aristomo Andries, Saranda Kabashi, Lene Lehmann Moberg

**Affiliations:** 1 Norwegian Institute of Public Health, Oslo, Norway; 2 University of Oslo, Oslo, Norway; 3 Norwegian University of Science and Technology, Trondheim, Norway; 4 Norwegian Labour and Welfare Administration, Oslo, Norway

**Keywords:** COVID-19, Sick leave, Primary healthcare, Long COVID, PASC, Post-acute sequelae of COVID-19

## Abstract

**Background:**

Since 2022, Norway has employed a vaccine-only COVID-19 strategy. Primary healthcare in Norway uses International Classification of Primary Care version 2 (ICPC-2) codes. This study aims to systematically compare medically certified sick leave and primary healthcare consultations in 2023 with the pre-pandemic 2010–2019 trends, and subsequently estimate the magnitude of these changes.

**Methods:**

For the respective outcomes of (A) working person-years lost to medically certified sick leave (WYLSL) and (B) number of primary healthcare consultations, 556 and 85 ICPC-2 code combinations were extracted from the Norwegian Labour and Welfare Administration’s sick leave registry and the Norwegian Syndromic Surveillance System. For each ICPC-2 code combination, a Bayesian linear regression was performed using data between 2010 and 2019 to estimate an expected baseline for 2023, which was then used to calculate the deviation from the pre-pandemic trend. A false discovery rate of 5% was used to account for multiple testing.

**Results:**

All years from 2020 to 2023 had excess WYLSL, corresponding to 14,491 (90% PI: 8,935 to 20,016) in 2020, 12,911 (90% PI: 5,916 to 19,996) in 2021, 21,263 (90% PI: 12,627 to 29,864) in 2022, and 24,466 (90% PI: 14,023 to 34,705) in 2023. This corresponded to an economic loss of approximately 1.5 billion USD in 2023. Excess WYLSL due to A* (General and unspecified) increased from 2020 to 2023, with an estimated excess of 4,136 WYLSL in 2023 (69% higher than expected). More than half of this increase was explained by A04 (Weakness/tiredness general), whose excess WYLSL in 2023 were estimated at 2,640 (80% higher than expected). The excess in A04 (Weakness/tiredness general) corresponded to an economic loss of 161 million USD and accounted for 11% of the total excess WYLSL in 2023. The excess WYLSL in R* (Respiratory) in 2023 was 3,408, which correspond to an economic loss of 207 million USD and accounted for 14% of the total excess in 2023.

**Conclusions:**

Significant excesses in working person-years lost to medically certified sick leave and primary healthcare consultations in 2023. A sizable proportion of the excesses were due to diseases/symptoms associated with acute and post-acute sequelae of COVID-19.

**Supplementary Information:**

The online version contains supplementary material available at 10.1186/s13690-024-01411-4.

## Abstract (Norwegian)

**Bakgrunn:**

Siden 2022 har Norge hatt en «kun vaksine»-basert strategi for covid-19. Primærhelsetjenesten i Norge bruker ICPC-2-koder. Denne studien har som mål å systematisk sammenligne legemeldt sykefravær og konsultasjoner i primærhelsetjenesten i 2023 med trendene fra før pandemien i perioden 2010–2019, og deretter estimere størrelsen på disse endringene.

**Metoder:**

For utfallene (A) tapte arbeidsår (tapte arbeidsdager delt på 250) på grunn av legemeldte sykefravær og (B) antall konsultasjoner i primærhelsetjenesten, ble 556 og 85 ICPC-2-kodekombinasjoner hentet fra NAVs sykefraværsregister og Det norske syndromiske overvåkingssystemet. For hver ICPC-2-kodekombinasjon ble det utført en Bayesiansk lineær regresjon ved bruk av data fra 2010–2019 for å estimere en forventet baseline for 2023, som deretter ble brukt til å beregne avviket fra trendene før pandemien. En falsk oppdagelsesrate på 5% ble brukt.

**Resultater:**

Alle år fra 2020 til 2023 viste et overskudd av tapte arbeidsår, tilsvarende 14 491 (90% PI: 8935 til 20 016) i 2020, 12 911 (90% PI: 5916 til 19 996) i 2021, 21 263 (90% PI: 12 627 til 29 864) i 2022 og 24 466 (90% PI: 14 023 til 34 705) i 2023. Dette tilsvarte et økonomisk tap på omtrent 16,7 milliarder NOK i 2023. Overskuddet i tapte arbeidsår pga. A* (Allment og uspesifisert) økte fra 2020 til 2023, med et beregnet overskudd på 4136 tapte arbeidsår i 2023 (69% høyere enn forventet). Mer enn halvparten av denne økningen ble forklart av A04 (Slapphet/tretthet), hvor overskudd i tapte arbeidsår i 2023 ble beregnet til 2640 (80% høyere enn forventet). Overskuddet i A04 (Slapphet/tretthet) tilsvarte et økonomisk tap på 1,8 milliarder NOK. Overskuddet i tapte arbeidsår pga. R* (Luftveier) i 2023 var 3408, noe som tilsvarte et økonomisk tap på 2,3 milliarder NOK og utgjorde 14% av det totale overskuddet i 2023.

**Fortolkning:**

Betydelig overskudd i både tapte arbeidsår på grunn av legemeldt sykefravær og konsultasjoner i primærhelsetjenesten i 2023. En betydelig del av overskuddene skyldes sykdommer/symptomer knyttet til akutt covid-19 og senfølger av covid-19.

Text box 1. Contributions to the literature

- Discussions about increased sick leave in Norway need more information about how the COVID-19 pandemic has affected people’s health and occupational health.
- Prior to this study, there had not been a systematic examination of the aberrations in medically certified sick leave and primary healthcare consultations in Norway in 2023 compared to pre-COVID-19 pandemic trends.
- A sizable proportion of the excesses in 2023 were due to diseases/symptoms associated with acute and post-acute sequelae of COVID-19.

## Background

During the first four years of the ongoing COVID-19 pandemic, there were an estimated 19.5 to 35.2 million global excess deaths, which is approximately the same number of lives lost to the HIV/AIDS pandemic over a forty-year period [1, 2]. In addition to acute mortality, even mild disease from COVID-19 can result in post-acute sequelae in multiple organ systems that may persist over many years [3]. There is evidence that the risk of post-acute sequelae is cumulative for each infection; in one study, Canadians with one, two and three or more self-reported COVID-19 infections had respectively a 14.6%, 25.4%, and 37.9% absolute risk of experiencing long-term symptoms [4]. A Spanish/British study also indicated that persistent symptoms are more common after reinfection compared to the first infection [5]. Furthermore, remission is not guaranteed: The aforementioned Canadian study reported that of the people reporting long-term symptoms in summer of 2022, 72.5% continue to experience symptoms one year later [4]. A Danish study reported that more than 50% of patients at a post-COVID clinic showed no improvement after 1.5 years [6].

Throughout 2020 and 2021, Norway implemented comprehensive and effective public health interventions against COVID-19 and did not observe any excess mortality [7]. However, in early 2022 Norway adopted a new COVID-19 strategy (“vaccine-only strategy”), where frequent and repeated infection was considered desirable to maintain herd-immunity [8–10]. The strategy was intended to protect hospitals from being overwhelmed by larger surges [8–10]. Infectious disease control measures (with the exception of vaccination) have therefore become undesirable, leading to a lack of government initiatives to reduce infection through measures such as improved indoor air quality through ventilation and/or air filtration. In 2022 excess mortality was estimated at 11.5% [7]. By the onset of winter 2023/2024, it was estimated that between 80 and 100% of the population had been infected at least once with SARS-COV-2, primarily after the implementation of the vaccine-only strategy in 2022 [9]. Excess mortality in 2023 was estimated at 5.6%, with a marked increase in excess mortality amongst 0–19-year-olds (14.6%) and 20–39-year-olds (9.7%) [11].

In accordance with the Norwegian government’s COVID-19 strategy, risk assessments from the Norwegian Institute of Public Health (NIPH; Norwegian: Folkehelseinstituttet) do not consider the consequences of long-term sequelae; they instead focus solely on hospitalization, ICU-usage, and acute deaths [9]. Regarding post-acute sequelae of COVID-19 in Norway, considerable emphasis is placed on rapid reviews from the NIPH [12].

From 2020 to 2022 medically certified sick leave increased, primarily due to COVID-19 International Classification of Primary Care version 2 (ICPC-2) codes [13]. In 2023 medically certified sick leave with a COVID-19 ICPC-2 code decreased, but medically certified sick leave increased for ICPC-2 codes that may be related to post-acute sequelae of COVID-19 [13]. The ICPC-2 code A04 (“Weakness/tiredness general”) increased considerably from 2020 to 2022 and has continued to increase in 2023 [13]. Medically certified sick leave due to the psychological ICPC-2 codes (P*) also increased in 2023 [13].

Regarding changes in Norwegian primary healthcare usage since 2020, most publications have focused on person-level changes in specific diseases/symptoms. Typical findings are that COVID-19 is associated with increased primary healthcare usage for pulmonary, neurological, and general complaints, which is in line with international research [14].

This study aims to systematically compare medically certified sick leave and primary healthcare consultations in 2023 with the pre-pandemic 2010–2019 trends, and subsequently estimate the magnitude of these changes. Furthermore, we investigate temporal associations with a proxy for community COVID-19 spread.

## Methods

### Data sources

Primary healthcare in Norway uses ICPC-2 codes. ICPC-2 codes capture the reason for the patient’s encounter or visit to the healthcare provider; approximately half represent diagnoses and half represent symptoms [15].

The Norwegian Labour and Welfare Administration (NAV; Norwegian: Arbeids- og velferdsforvaltninga) owns and manages the sick leave registry. This registry contains information on all employed people (except for self-employed people and seafarers) between 16 and 69 years residing in Norway who have medically certified sick leave. In Norway, the sickness benefit period can last up to one year. Each occurrence of medically certified sick leave has an ICPC-2 code associated with it, as assigned by the medical authority certifying the sick leave. Statistics Norway estimated that in the fourth quarter of 2023 83% of sick leave was medically certified and 17% self-certified.

We extracted two outcome measures from NAV: (1) The number of working person-days lost to medically certified sick leave, which is adjusted for part-time work and graded sickness absence, and (2) New cases of medically certified sick leave, which counts the number of new people registered as sick each week.

The Norwegian Syndromic Surveillance System (NorSySS; Norwegian: Det norske syndromiske overvåkingssystemet) is a public health surveillance system designed to detect outbreaks of infectious diseases and provide early warning for implementation of necessary control measures [16]. NorSySS surveils the number of consultations at general practitioners and out-of-hours primary care facilities. NorSySS’ data source is KUHR (Control and Payment of Health Reimbursements; Norwegian: Kontroll og utbetaling av helserefusjoner), which is a system that manages reimbursement claims from healthcare providers and institutions to the state in Norway. The system is owned by the Norwegian Directorate of Health. KUHR is a system within KPR (Municipal Patient and User Register; Norwegian: Kommunalt pasient- og brukerregister) that contains data from municipalities about individuals who have applied for, receive, or have received health and care services.

We extracted one outcome measure from NorSySS: The number of primary healthcare consultations per ICPC-2 code. A consultation was defined as one interaction with a primary healthcare practitioner that corresponds to one of the following: home visit by a general practitioner (day/night), consultation with a general practitioner (day/night), consultation for being called to the office for immediate help of a patient, e-consultation with a general practitioner and/or emergency room (day/night). These correspond to the tariff codes 11ad, 11ak, 2ad, 2ak, 2fk, 2ae, 2aek.

### Relationship between NAV data and NorSySS data

Both the NAV data and NorSySS data are “initiated” by a primary healthcare physician’s consultation. The NAV data corresponds to consultations for diseases/symptoms that are severe enough to lead to medically certified sick leave, whereas the NorSySS data corresponds to the number of consultations that occur.

Both datasets are focused on primary healthcare, and it is therefore expected that the results will be quite similar. Since the NAV dataset contains 556 ICPC-2 code combinations, while the NorSySS dataset only contains 85 ICPC-2 code combinations, the NAV results will be presented in the main article and the NorSySS results will be primarily presented in the supplemental materials (Additional file 2).

### Working person-years lost to medically certified sick leave and subsequent economic loss

The number of working person-years lost to medically certified sick leave was defined as the number of working person-days lost to medically certified sick leave divided by 250 (the expected number of working days per person per year).

With a median annual salary of 676,320 NOK / 64,000 USD in 2023 [17], the approximate economic loss was calculated by multiplying the median annual salary by the number of working person-years lost to medically certified sick leave.

The amount of money paid by NAV to the recipients is different to the economic loss of the medically certified sick leave.

### Comparing 2023 against a 2010–2019 baseline

From NAV, data was extracted for 556 ICPC-2 code combinations (Supplemental Table 1, Additional file 1), representing the working person-days lost to medically certified sick leave from 2010 to 2023. Data was extracted for males, females, and all sexes combined. These numbers were then rescaled to 2023-population levels using Statistics Norway’s Labor Survey [18].

The aim of this analysis was to use the data from 2010 to 2019 to predict expected baselines for 2020–2023, then calculate the excess values for 2020–2023 by subtracting the observed values from the expected baselines.

To calculate the expected/excess values for 2020 to 2023, one analysis was performed for each combination of: male/female/all sexes, and each ICPC-2 code combination.

For NAV, to investigate the appropriate model for the expected baseline, three linear regressions were performed on data between 2010 and 2019:

1. Outcome: Rate/100k, Covariate: Year as a continuous linear variable.
2. Outcome: Rate/100k, Covariate: Year as a cubic spline with two degrees of freedom.
3. Outcome: Rate/100k, Covariate: Year as a cubic spline with three degrees of freedom.

The model with the lowest AIC was selected, and then a Bayesian linear regression was performed using the selected model between 2010 and 2019, with 4 chains each containing 20,000 iterations. The Bayesian linear regression was implemented using the “rstan” package in R, which uses gradient-based Markov chain Monte Carlo algorithms [19, 20]. The expected baseline for 2020 to 2023 was then calculated by estimating the posterior of the rate/100k. The expected baselines for 2020–2023 were then used to calculate the excess values and corresponding prediction intervals.

The excess values were then restricted to 2022 and 2023 and corrected for multiple testing using false discovery rates (FDR) with a threshold of 0.05. After FDR correction, significant results with an absolute excess value less than 10,000 were discarded due to not being clinically relevant.

### Temporal association with community spread of COVID-19 between 2020 and 2023

There is no consistent data on community spread of COVID-19 in Norway for the entire period of 2020 to 2023. Since the implementation of the “vaccine-only strategy” in 2022, polymerase chain reaction (PCR) testing and subsequent registering of results became an even more unreliable indicator, and wastewater ribonucleic acid (RNA) concentration measurements for SARS-CoV-2 were only performed between mid-2022 and the end of 2023 [21].

A proxy therefore had to be created to identify a temporal correlation with community spread of COVID-19. Three proxies were created by assuming that two doses of a COVID-19 vaccine were 90%, 80% and 70% effective at preventing hospitalization [22]. The proxies were calculated by the formula:

$$\begin{aligned}\mathrm{Proxy}\;\mathrm{for}\;\mathrm{community}\;\mathrm{spread}\;\mathrm{of}\;\mathrm{COVID19}=\\\hspace{1cm}\left(\mathrm{Weekly}\;\mathrm{incidence}\;\mathrm{of}\;\mathrm{COVID19}\;\mathrm{hospitalization}\right)\;\ast\;\\\hspace{1cm}\left(\mathrm{proportion}\;\mathrm{of}\;\mathrm{the}\;\mathrm{population}\;\mathrm{with}\;\mathrm{fewer}\;\mathrm{than}\;\mathrm{two}\;\mathrm{vaccine}\;\mathrm{doses}\right)\;\\\hspace{5cm}+\;\\\hspace{1cm}\left(\mathrm{Weekly}\;\mathrm{incidence}\;\mathrm{of}\;\mathrm{COVID19}\;\mathrm{hospitalization}\right)\;\ast\;\\\hspace{1cm}\left(\mathrm{proportion}\;\mathrm{of}\;\mathrm{the}\;\mathrm{population}\;\mathrm{with}\;\mathrm{at}\;\mathrm{least}\;\mathrm{two}\;\mathrm{vaccine}\;\mathrm{doses}\right)\;/\;\\\hspace{1cm}\left(1-\mathrm{effectiveness}\;\mathrm{of}\;\mathrm{vaccine}\right)\end{aligned}$$

The aim of this proxy was to construct a weekly incidence of COVID-19 hospitalizations in a hypothetical population where no-one is vaccinated, to remove the period effect of pre/post-vaccinated Norway. The proxy with 90% vaccine effectiveness was used for the primary analysis. Sensitivity analyses were then performed using the 80% and 70% proxies.

The ICPC-2 code combinations that were identified as significant from the previous analysis were then extracted as weekly data from 2020 to 2023, all ages and sexes combined.

For NAV, the outcome was the number of new cases of medically certified sick leave for the desired ICPC-2 code combinations. These data were then scaled into rates using the employed workforce population, and then aggregated into quarters (weeks 1–13, 14–26, 27–39, 40–52) by isoyear (2020, 2021, 2022, 2023). The Pearson correlation was then calculated for each ICPC-2 code combination against the proxy for community spread of COVID-19.

The p-values corresponding to the Pearson correlations were then corrected for multiple testing using false discovery rates with a threshold of 0.05. Those that were found to be under the threshold were then graphically displayed as quarterly timeseries.

### Statistical software

All analyses were performed in R, version 4.3.2 [20].

## Results

### 2023 compared to 2010–2019

In NAV there were respectively 35 and 36 ICPC-2 code combinations in 2022 and 2023 that had a statistically significant excess/deficit after false discovery rate correction and exclusion for clinical significance (Supplemental Tables 3 and 4, Additional file 3 and Tables 1 and 2). Examination of the residuals did not reveal any statistical issues with the models (Additional file 5).

**Table 1:** Excess working person-days lost to medically certified sick leave in 2023 for all sexes combined

ICPC-2	Observed	Excess				
	Value in 1000s	Value (90% PI) in 1000s	Ratio (90% PI)	Proportion explained of “Everything”^a^		Economic loss in million USD^b^
				Point	Cumulative	
Everything	65,015	6,116 (3,506 to 8,676)^d^	1.10 (1.06 to 1.15)^d^	-	-	1,489
A* General and unspecified	2,523	1,034 (898 to 1,170)^d^	1.69 (1.55 to 1.86)^d^	17%	17%	252
R* Respiratory	2,646	852 (577 to 1,127)^d^	1.47 (1.28 to 1.74)^d^	14%	31%	207
↳R991/R992 COVID-19^**c**^	498	-	-	8%	-	121
P29 Psychological symptom/complaint other	1,707	727 (607 to 847)^d^	1.74 (1.55 to 1.98)^d^	12%	43%	177
A04 Weakness/tiredness general	1,484	660 (518 to 804)^d^	1.80 (1.54 to 2.18)^d^	11%	-	161
P02 Acute stress reaction	1,896	519 (269 to 774)^d^	1.38 (1.17 to 1.69)^d^	8%	51%	126
P03 Feeling depressed	406	277 (222 to 331)^d^	3.14 (2.20 to 5.40)^d^	5%	56%	67
L02 Back symptom/complaint	744	264 (116 to 410)	1.55 (1.18 to 2.23)	4%	60%	64
L08 Shoulder symptom/complaint	674	257 (199 to 315)^d^	1.62 (1.42 to 1.88)^d^	4%	64%	63
L01 Neck symptom/complain	329	143 (110 to 176)^d^	1.77 (1.51 to 2.15)^d^	2%	67%	35
R74 Upper respiratory infection acute	739	140 (66 to 213)	1.23 (1.1 to 1.41)	2%	69%	34
N01 Headache	298	71 (54 to 88)^d^	1.31 (1.22 to 1.42)^d^	1%	70%	17
A99 General disease NOS	135	65 (40 to 90)^d^	1.94 (1.43 to 3.03)^d^	1%	71%	16
P81 Hyperkinetic disorder	123	58 (40 to 78)^d^	1.91 (1.48 to 2.72)^d^	< 1%	72%	14
L13 Hip symptom/complaint	193	43 (23 to 64)^d^	1.29 (1.13 to 1.50)^d^	< 1%	73%	10
L12 Hand/finger symptom/complaint	118	38 (30 to 47)^d^	1.48 (1.34 to 1.66)^d^	< 1%	73%	9
L11 Wrist symptom/complaint	105	38 (28 to 47)^d^	1.56 (1.37 to 1.81)^d^	< 1%	74%	9
L16 Ankle symptom/complaint	126	31 (26 to 37)^d^	1.33 (1.26 to 1.41)^d^	< 1%	75%	8
A01 Pain general/multiple sites	47	22 (13 to 31)	1.86 (1.36 to 2.86)	< 1%	-	5
R72 Strep throat	47	21 (17 to 25)^d^	1.81 (1.55 to 2.18)^d^	< 1%	-	5
A03 Fever	49	19 (12 to 27)^d^	1.65 (1.32 to 2.20)^d^	< 1%	-	5
L19 Muscle symptom/complaint NOS	27	18 (11 to 25)	3.03 (1.61 to 9.34)	< 1%	75%	4
A77 Viral disease other/NOS	28	14 (11 to 17)^d^	2.02 (1.64 to 2.63)^d^	< 1%	-	3
D04 Rectal/anal pain	45	14 (10 to 18)^d^	1.46 (1.28 to 1.71)^d^	< 1%	75%	3
X21 Breast symptom/complt. female other	23	12 (9 to 16)^d^	2.14 (1.61 to 3.20)^d^	< 1%	76%	3
P19 Drug abuse	34	12 (6 to 18)^d^	1.55 (1.23 to 2.11)^d^	< 1%	76%	3

ICPC-2 codes shown are those where the excess was statistically significantly (for either all sexes, male, or female) after false discovery rate correction with a 5% cutoff and the absolute value of excess was larger than 10,000. Excess value is defined as observed minus expected baseline, excess ratio is defined as observed divided by expected baseline, and both are presented as point estimate (90% prediction interval). Table 1 contains the ICPC-2 codes where excess is positive, and Table 2 contains the ICPC-2 codes where excess is negative (i.e. deficit). Sex-specific results and results from 2022 are shown in Additional File 3. Data from NAV

^a^Excess value of the given ICPC-2 code combination divided by 6116 (the excess value of Everything)

^b^Excess value of the given ICPC-2 code combination divided by 250 (number of workdays), multiplied by 676,320 (median annual salary), divided by 1000 and multiplied by 90 (1000 NOK = 90 USD), and divided by 10^6 (transforming to millions)

^c^COVID-19 does not have an expected baseline

^d^Significant after false discovery rate correction with a 5% cutoff

**Table 2:** Negative excess (deficit) working person-days lost to medically certified sick leave in 2023 for all sexes combined

ICPC-2	Observed	Excess		
	Value in 1000s	Value (90% PI) in 1000s	Ratio (90% PI)	Economic loss in million USD^a^
D92 Diverticular disease	24	-11 (-15 to -6)^b^	0.69 (0.62 to 0.79)^b^	-3
L71 Malignant neoplasm musculoskeletal	11	-11 (-17 to -5)	0.50 (0.39 to 0.68)	-3
R90 Hypertrophy tonsils/adenoids	25	-12 (-17 to -7)^b^	0.67 (0.59 to 0.77)^b^	-3
D87 Stomach function disorder	14	-13 (-22 to -3)	0.53 (0.39 to 0.81)	-3
T99 Endocrine/metab/nutrit. dis. other	44	-13 (-19 to -8)^b^	0.76 (0.69 to 0.85)^b^	-3
T85 Hyperthyroidism/thyrotoxicosis	56	-18 (-23 to -14)^b^	0.75 (0.71 to 0.80)^b^	-4
K76 Ischaemic heart disease w/o angina	61	-19 (-28 to -11)^b^	0.76 (0.69 to 0.85)^b^	-5
B99 Blood/lymph/spleen disease other	30	-20 (-25 to -15)^b^	0.60 (0.55 to 0.67)^b^	-5
N79 Concussion	147	-60 (-82 to -38)^b^	0.71 (0.64 to 0.80)^b^	-15
L81 Injury musculoskeletal NOS	328	-78 (-117 to -38)	0.81 (0.74 to 0.90)	-19

ICPC-2 codes shown are those where the excess was statistically significantly (for either all sexes, male, or female) after false discovery rate correction with a 5% cutoff and the absolute value of excess was larger than 10,000. Excess is defined as (observed minus baseline) and presented as point estimate (90% prediction interval). Table 1 contains the ICPC-2 codes where excess is positive, and Table 2 contains the ICPC-2 codes where excess is negative (i.e. deficit). Sex-specific results and results from 2022 are shown in Additional File 3. Data from NAV

^a^Excess value of the given ICPC-2 code combination divided by 250 (number of workdays), multiplied by 676,320 (median annual salary), divided by 1000 and multiplied by 90 (1000 NOK = 90 USD), and divided by 10^6 (transforming to millions)

^b^Significant after false discovery rate correction with a 5% cutoff

The results were consistent for the ICPC-2 code combinations that existed in both NAV and NorSySS, with the exception of NorSySS finding an excess for D11 + D70 + D73 gastroenteritis, which was not present in the NAV data.

**Fig. 1:**
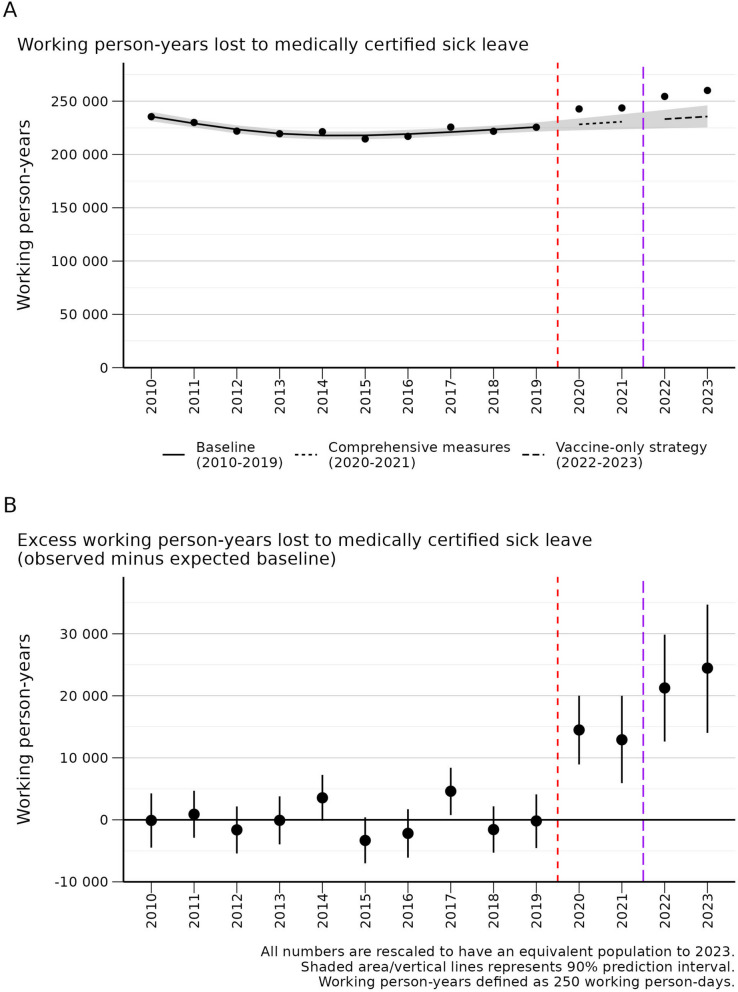
Working person-years lost to medically certified sick leave (2010–2023)

**Fig. 2:**
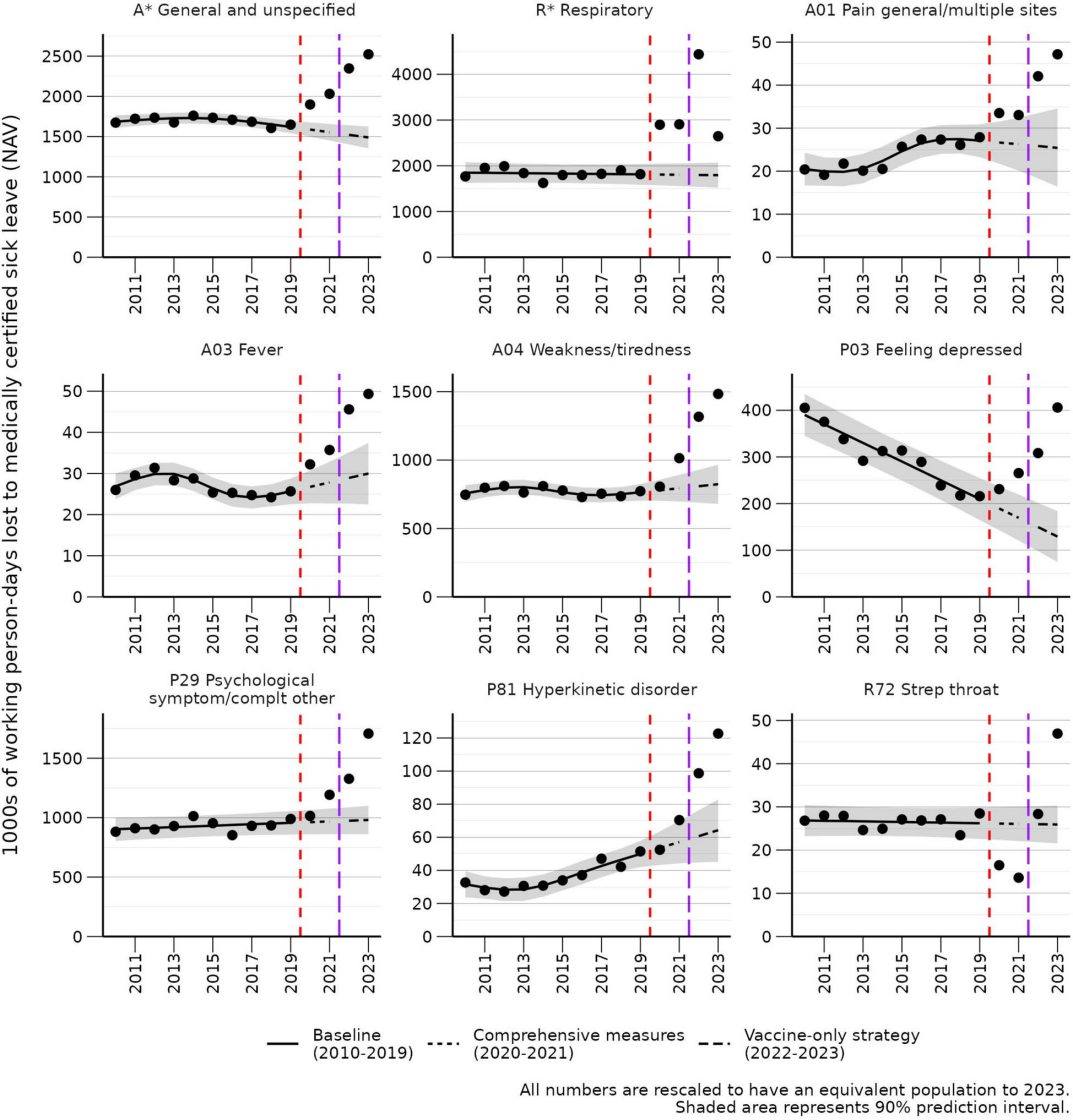
Selected trends in medically certified sick leave where 2023 is higher than expected. Selected trends are shown here. All significant trends, including sex-specific results, are shown in Additional file 3

After visual inspection of the trends for the ICPC-2 code combinations that had significant excesses in 2023, 10 were chosen to be displayed in the main results due to excessive deviation from the expected baseline (Figs. 1 and 2). The remaining ICPC-2 code combinations can be viewed in Additional file 4.

**Fig. 3:**
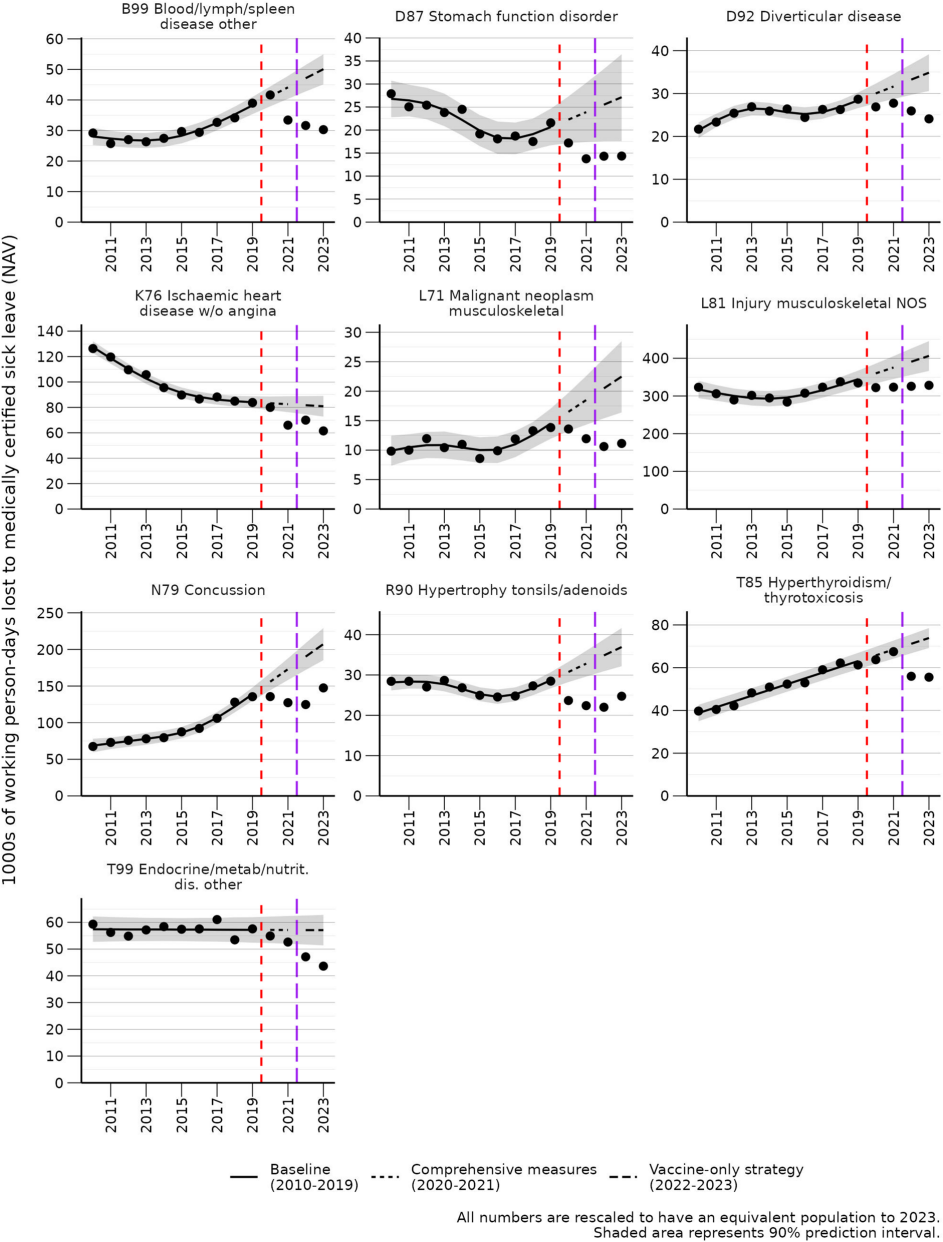
Selected trends in medically certified sick leave where 2023 is lower than expected. Selected trends are shown here. All significant trends, including sex-specific results, are shown in Additional file 3

All 10 ICPC-2 code combinations that had significant deficits in 2023 were displayed in the main results (Fig. 3).

### Excess working person-years lost to medically certified sick leave

All years from 2020 to 2023 have had statistically significant excess working person-years lost to medically certified sick leave, corresponding to 14,491 (90% PI: 8,935 to 20,016) in 2020, 12,911 (90% PI: 5,916 to 19,996) in 2021, 21,263 (90% PI: 12,627 to 29,864) in 2022, and 24,466 (90% PI: 14,023 to 34,705) in 2023 (Table 1; Figs. 1 and 2). This corresponds to an economic loss of approximately 16.5 billion NOK / 1.5 billion USD in 2023 (Table 1).

### Weakness/tiredness general (fatigue)

Excess working person-days lost to medically certified sick leave due A* (General and unspecified) increased from 2020 to 2023, with an estimated excess of 1,034,000 working person-days lost to medically certified sick leave in 2023 (69% higher than expected) (Table 1; Fig. 2). More than half of this increase was explained by A04 (Weakness/tiredness general), whose excess working person-days lost to medically certified sick leave in 2023 were estimated at 660,000 (80% higher than expected) (Table 1; Fig. 2).

The excess in A04 (Weakness/tiredness general) accounted for 11% of the total excess working person-days lost to medically certified sick leave in 2023, corresponding to approximately 2640 working person-years lost to medically certified sick leave and an economic loss of 1.8 billion NOK / 161 million USD (Table 1).

Working person-days lost to medically certified sick leave due to A04 (Weakness/tiredness general) in 2020 remained within expected bounds, while 2021 was slightly above expected, and there was a dramatic increase in 2022 (Fig. 2). There was a further increase in 2023 (Fig. 3).

**Fig. 4:**
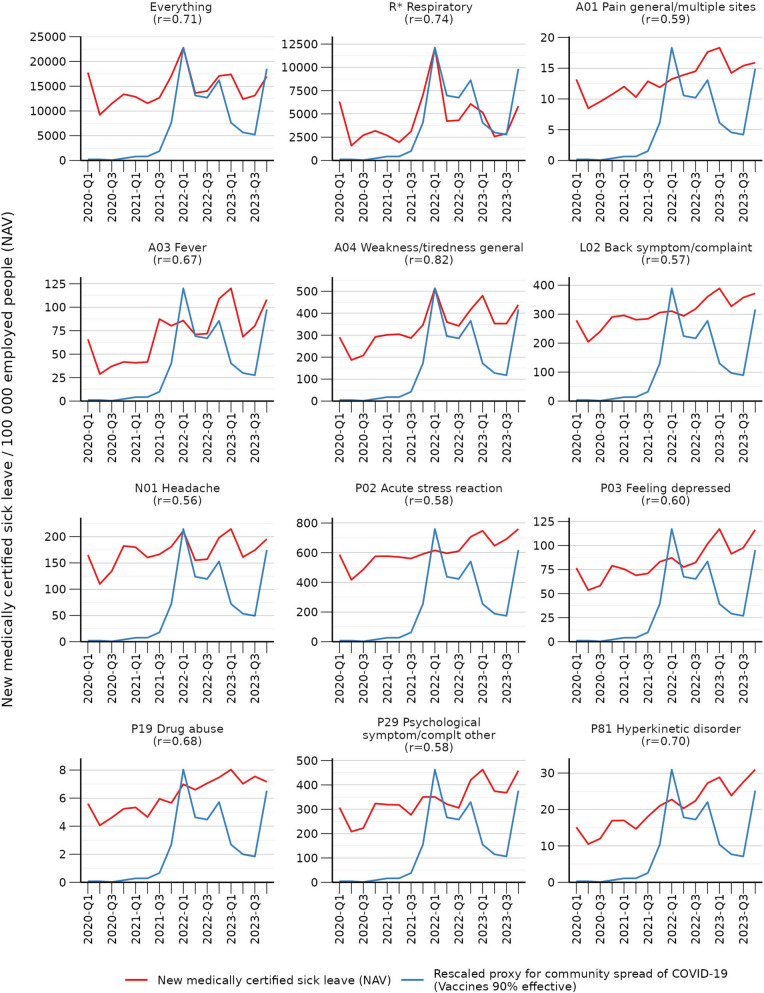
Medically certified sick leave and community spread of COVID-19 (assuming 90% vaccine effectiveness) from 2020-Q1 to 2023-Q4. r = Pearson’s correlation coefficient

There was a strong temporal correlation (*r* = 0.82) for excess lost days of work due to A4 (Weakness/tiredness general) with the proxy for community spread of COVID-19 (Fig. 4).

Findings were consistent in both NAV and NorSySS data (Additional file 2).

### Psychological symptom/complaint other

Excess working person-days lost to medically certified sick leave due to P29 (Psychological symptom/complaint other) increased in 2021, 2022, and 2023, with an estimated excess of 727,000 working person-days lost to medically certified sick leave in 2023 (74% higher than expected) (Table 1; Fig. 2).

The excess in P29 (Psychological symptom/complaint other) accounted for 12% of the total excess working person-days lost to medically certified sick leave in 2023, corresponding to an approximate economic loss of 2 billion NOK / 177 million USD (Table 1).

There was a moderate temporal correlation (*r* = 0.58) with the proxy for community spread of COVID-19 (Fig. 4).

### Respiratory

R* (Respiratory) accounted for 14% of the total excess working person-days lost to medically certified sick leave in 2023, for an economic loss of 2.3 billion NOK / 207 million USD (Table 1; Fig. 2).

Excess working person-days lost to medically certified sick leave due to R* (Respiratory) peaked in 2022, corresponding to the first Omicron wave, yet still remains 47% higher than expected in 2023 (Table 1; Fig. 2).

There was a strong temporal correlation (*r* = 0.74) with the proxy for community spread of COVID-19 (Fig. 4).

Findings were consistent in both NAV and NorSySS data (Additional file 2).

### COVID-19

It was not possible to estimate an expected baseline for COVID-19, as the ICPC-2 codes for the disease did not exist before 2020.

R991/R992 (COVID-19) accounted for 8% of the total excess working person-days lost to medically certified sick leave in 2023, for an economic loss of 1.3 billion NOK / 121 million USD (Table 1). This corresponded to 19% of all working person-days lost to medically certified sick leave for R* (Respiratory) in 2023 (Table 3).

**Table 3:** Working person-days (1000s) lost to medically certified sick leave from 2018 to 2023 for all sexes combined for a subset of ICPC-2 codes

ICPC-2			Working person-days (1000s) lost to medically certified sick leave					
			2018	2019	2020	2021	2022	2023
Everything			55,436	56,384	60,676	60,904	63,609	65,015
R* Respiratory			1,901	1,815	2,898	2,907	4,438	2,646
R991/R992 COVID-19			0	0	717	1,163	2,476	498
R80 Influenza			371	239	216	60	192	273

All numbers are rescaled to 2023-population levels using Statistics Norway’s Labor Survey

### Pain

Excess working person-days lost to medically certified sick leave due to A01 (Pain general/multiple sites) was borderline higher than expected in 2020 and 2021, but significantly higher in 2022 and 2023 (Fig. 2). Furthermore, 2023 was higher than 2022 (Fig. 2).

### Gastroenteritis

This finding only occurred in NorSySS data. D11 + D70 + D73 (Gastroenteritis) had significantly fewer primary healthcare consultations than expected during 2020 and 2021, and significantly more than expected in 2022 and 2023 (Additional file 2). Furthermore, there were more consultations in 2023 than in 2022 (Additional file 2).

There was a moderate temporal correlation with the proxy for community spread of COVID-19 (*r* = 0.67) (Additional file 2).

### Strep throat

Excess working person-days lost to medically certified sick leave due to R72 (Strep throat) was significantly lower than expected during 2020 and 2021, returned to expected levels in 2022, and was significantly higher than expected during 2023 (Fig. 2).

Findings were consistent in both NAV and NorSySS data (Additional file 2).

### Hyperkinetic disorder

The number of working person-days lost to medically certified sick leave for P81 (Hyperkinetic disorder) had a steady consistent increasing trend from 2011 to 2020, however, it the trend increased rapidly in 2021 and even more so in 2022 and 2023 (Fig. 2).

There was a strong temporal correlation (*r* = 0.70) with the proxy for community spread of COVID-19 (Fig. 4).

### Depression

The number of working person-days lost to medically certified sick leave due to P03 (Feeling depressed) was on a steady consistent decline from 2010 to 2019, when it abruptly started to increase in 2020, further increasing in 2021, 2022, and 2023 (Fig. 2).

### Temporal association with community spread of COVID-19 between 2020 and 2023

A proxy for community-spread of COVID-19 (90% vaccine efficacy) was found to be positively temporally correlated with the following ICPC-2 code combinations in NAV: Everything (r: 0.71), R* (Respiratory; r: 0.74); A1 (Pain general/multiple sites; r: 0.59), A03 (Fever; r: 0.67), A04 (Weakness/tiredness general; r: 0.82), L02 (Back symptom/complaint; r: 0.57), N01 (Headache; r: 0.56), P02 (Acute stress reaction; r: 0.58), P03 (Feeling depressed; r: 0.60), P19 (Drug abuse; r: 0.68), P29 (Psychological symptom/complaint other; r: 0.58), P81 (Hyperkinetic disorder; r: 0.70) (Fig. 4).

These findings were nearly identical when using a proxy with 80% and 70% vaccine efficacy (Additional files 5).

Findings were consistent in both NAV and NorSySS data (Additional file 2).

## Discussion

This study provides important insights into temporal changes in medically certified sick leave and primary healthcare utilization in Norway, reflecting systemic changes in healthcare utilization and labor markets. Our findings indicate significant excesses in working person-days lost to medically certified sick leave and primary healthcare consultations in 2023, deviating from the 2010–2019 trends. Many of these changes were found to be temporally correlated with a proxy for community spread of COVID-19.

This is an observational study of aggregated temporal trends, so causal attribution of the findings is not possible. There have been several large changes to Norwegian society between 2020 and 2023, such as the wide-spread introduction of a novel virus (SARS-CoV-2), comprehensive preventative measures in 2020 and 2021, and the cost-of-living-crisis in 2022–2023. All of these may have served to exacerbate existing trends or create new trends. We shall therefore interpret with caution our findings that may be evidence of larger trends identified elsewhere.

### The role of e-consultations in NorSySS

For the purposes of this study, both physical consultations and e-consultations were treated equally in NorSySS. The use of e-consultations in Norway grew dramatically from 2020, which could have affected our results by allowing for a lower-threshold to contact the general practitioner. From 2010 to 2019 the median of the mean annual number of consultations per general practitioner was 1957, with an IQR between 1947 and 1972. For 2020, 2021, 2022, and 2023, the mean annual number of consultations per general practitioner were 2028, 2122, 2047, and 1950 respectively. Given that the mean annual number of consultations per general practitioner in in 2023 had returned to the pre-pandemic norms, we consider it more likely that, in 2023, e-consultations had replaced physical consultations.

### “Immunity debt”, “immunity theft”, and the impact of COVID-19

When considering the concept of “immunity debt”, it is important to note that any exposure to pathogens has an inherent risk of illness or death. Proposed by a group of French researchers, the concept argues that during the comprehensive measures in 2020 and 2021, newborn children were born into an environment with fewer circulating pathogens, hence not exposed to certain pathogens, resulting in them lacking adaptive immunity to these pathogens (compared to their equally aged counterfactuals in 2019) [23]. For some adolescents and adults, adaptive immunity for specific pathogens may have waned during 2020 and 2021. Consequently, when the comprehensive measures were lifted, the population without adaptive immunity to specific pathogens in 2022 was likely larger than in 2019. This suggests that, for a limited time, there would be a surge in some infectious diseases at the population level. This concept argues that exposure to various pathogens will gradually rebuild the population’s adaptive immunity to those pathogens (with an associated increase in illness and/or death for those exposed to the pathogens), eventually stabilizing infection rates to pre-pandemic levels. The concept does not support exposure to pathogens to improve adaptive immunity, as any exposure to pathogens has an inherent risk; vaccination is a safer method of gaining adaptive immunity to specific pathogens. The concept of “immunity debt” does not specify when the “debt” is expected be paid off.

COVID-19 is itself associated with subsequent increased risk of other diseases, which complicates the concept that “immunity debt” is the cause of increased illness after the cessation of comprehensive measures. This concept is known as “immunity theft,” where SARS-CoV-2 infection leaves some people more susceptible to other infections [24]. In support of “immunity theft”, one study in the US showed that COVID-19 was associated with a significantly increased risk of RSV among children aged 0–5 years in both 2022 and 2021, with the authors postulating that their findings “suggest that COVID-19 contributed to the 2022 surge of RSV cases in young children through the large buildup of COVID-19-infected children and the potential long-term adverse effects of COVID-19 on the immune and respiratory system” [25]. Another study found that, in Thailand, COVID-19 pneumonia was strongly associated with a higher hazard of detectable active pulmonary tuberculosis, and it was likely that this hazard was not fully explained by diagnostic bias [26].

For context, COVID-19 (suspected and confirmed) accounted for 56% and 19% of all respiratory-related lost working person-days to medically certified sick leave in Norway in 2022 and 2023, respectively (Table 3). In comparison, influenza accounted for 4% and 10% of these respiratory-related working person-days lost in the same years (Table 3).

Some post-acute sequelae of COVID-19, such as fatigue and respiratory issues, are similar to those of other post-viral illnesses, such as post-acute sequelae of influenza. However, the acute and post-acute burdens of COVID-19 are higher than influenza in a wide variety of organ systems [27]. Frequency of reinfection is a crucial driver of the incidence of post-viral illnesses; individuals are typically infected with influenza once every five years [28]. It is currently unknown how often an individual in Norway is reinfected with SARS-CoV-2.

In accordance with the Norwegian government’s COVID-19 strategy, risk assessments from the Norwegian Institute of Public Health do not consider the consequences of long-term sequelae; they instead focus solely on hospitalization, ICU-usage, and acute deaths [9].

This unique situation raises concerns about the long-term health impacts of repeated SARS-CoV-2 infections. Therefore, the discussion focuses on whether the literature supports that excess illness observed in 2023 could be partially or wholly attributed to acute sequelae of COVID-19, post-acute sequelae of COVID-19, or adverse effects on immune systems due to COVID-19.

### Weakness/tiredness general (fatigue)

This study estimated an economic loss of 1.8 billion NOK / 161 million USD in 2023 due to excess working person-years lost to medically certified A04 (Weakness/tiredness general) sick leave.

Fatigue is one of the most well-known post-acute sequelae of COVID-19 [3]. A Norwegian study showed that people who tested positive for the Delta and Omicron variants of SARS-CoV-2 were more likely to seek healthcare for fatigue (the outcome included A04) in the 126 days post-infection compared to those who tested negative [14]. Another Norwegian study investigated if sickness absence due to COVID-19 was associated with subsequent sick leave due to A04 (Weakness/tiredness general) and found a strong positive association [29]. However, it is important to remember that A04 (Weakness/tiredness general) is a common diagnosis, and the increase may be partially or wholly explained by other causes than COVID-19; the use of the diagnosis has previously fluctuated with conditions in the labor market [30]. These studies indicate that the increase is likely to be associated with COVID-19, but other societal factors cannot be excluded.

The addition of all the deficits in 2023 (L81, N79, B99, K76, T85, T99, D87, R90, L71, D92) summed to negative 255,000 working person-days lost to medically certified sick leave. In comparison, A04’s excess was 660,000. Hence there is no evidence that A04’s excess can be fully explained by a change in coding practices.

If the increase in A04 is partially or wholly related to COVID-19, it could imply that the societal burden will continue to increase as new individuals are infected and develop long-lasting sequelae. A Danish study reported that more than 50% of patients at a post-COVID clinic showed no improvement after 1.5 years [6]. The diagnosis of A04 also corresponds to the diagnosis used for myalgic encephalomyelitis/chronic fatigue syndrome, of which about half of long-covid patients fulfill the criteria [31]. For this patient population, few show benefit of return-to-health/work interventions [32].

### Psychological symptom/complaint other and depression

P29 (Psychological symptom/complaint other) accounts for 12% of the total excess working person-days lost to medically certified sick leave in 2023, for an estimated economic loss of 2 billion NOK / 177 million USD.

The ICPC-2 code P29 (Psychological symptom/complaint other) is used for psychological issues without an obvious source. For example, the Norwegian Directorate of Health’s (Norwegian: Helsedirektoratet) guidance to primary healthcare doctors regarding “burnout” is to record it under P29 [33].

A previous study by the Norwegian Labour and Welfare Administration analyzed all people on medically certified sick leave from 1st of January 2020 to 30th of June 2022 [29]. The study compared 253,460 people with medically certified sick leave due to COVID-19 against 718,328 people with medically certified sick leave for not COVID-19. The study found that within 0–30 days after the end of the medically certified sick leave, 533 people (210/100,000 people) in the COVID-19 group had a medically certified sick leave for P29, compared to 1475 people (205/100,000 people) in the non-COVID-19 group. Within 0–12 weeks, these numbers were 1410 people (556/100,000 people) in the COVID-19 group and 3392 people (472/100,000 people) in the non-COVID-19 group, corresponding to an 18% higher rate in the COVID-19 group. These numbers are not adjusted for confounders.

P03 (Feeling depressed) accounts for 5% of the total excess working person-days lost to medically certified sick leave in 2023, for an estimated economic loss of 740 million NOK / 67 million USD.

In 2020 and 2021 Norway experienced comprehensive measures to prevent the spread of SARS-CoV-2, and in 2022 and 2023 Norway experienced a cost-of-living-crisis. It is expected that these events would negatively affect the population’s mental health. A Norwegian study found that despite the increasing trend, sick leave for common mental disorders were just as common in the control group as in the COVID-19 group between 2020 and 2022 [29]. Sick leave for mental disorders continued to increase in 2023 [13]. Another Norwegian study showed that people who tested positive for the Delta and Omicron variants of SARS-CoV-2 were no more likely to seek healthcare for anxiety/depression in the 126 days post-infection compared to those who tested negative [14]. In contrast, other international studies do show evidence of increased risk of depression after SARS-CoV-2 [3, 34].

### Respiratory

R* (Respiratory) accounted for 14% of the total excess working person-days lost to medically certified sick leave in 2023, for an estimated economic loss of 2.3 billion NOK / 207 million USD.

R* (Respiratory) contained many individual ICPC-2 codes; R991 + R992 (COVID-19 suspected and confirmed) comprised 56% and 19% of all R* (Respiratory) working person-days lost to medically certified sick leave in 2022 and 2023 respectively (Table 3). In comparison, influenza comprised 4% and 10% of all R* (Respiratory) working person-days lost to medically certified sick leave in 2022 and 2023 respectively (Table 3).

This aligns with Norway’s strategy of maintaining a high level of SARS-CoV-2 infection [8–10].

### Pain

Pain is a common post-acute sequela of COVID-19 [35], and the increase in 2023 from 2022 may be indicative of an increased morbidity in the general population due to the cumulative risk of post-acute sequelae, but other factors cannot be excluded.

### Gastroenteritis

A small internal investigation was launched inside the Norwegian Institute of Public Health, but no similar reported increase was found in typical infectious disease pathogens that cause gastroenteritis (data/results not shown). A previous Norwegian study found that within people who had medically certified sick leave, those with R992 (Confirmed COVID-19) were more likely to have subsequent medically certified sick leave due to D73 (Gastroenteritis presumed infection) compared to those who did not have R992 [29]. Furthermore, gastrointestinal issues are well-known post-acute sequelae of COVID-19 [35]. These studies indicate that the increase may be associated with COVID-19, but other factors cannot be excluded.

### Strep throat

Studies in people with post-acute sequelae of COVID-19 have observed immune dysregulation [35], which could make individuals more susceptible to post-COVID infections. One study in Israel found that patients infected with SARS-CoV-2 were at increased risk for streptococcal tonsillitis compared to those who tested negative [36]. The increase may be associated with COVID-19, but other factors cannot be excluded.

### Hyperkinetic disorder

While there is no evidence for a link between COVID-19 and ADHD/ADD, there is strong evidence of neurological and psychological sequelae caused by COVID-19 [34, 35]. These sequelae, such as persistent memory problems [37] and cognition problems [38], may lead a patient to seek out their primary healthcare for an initial screening for ADHD/ADD or similar issues. That is, this increased trend may be partially explained by neurological and psychological post-COVID-19 sequelae causing individuals to seek healthcare for what they believe to be ADHD/ADD, but other factors cannot be excluded.

### Deficits

The number of working person-days lost to medically certified sick leave due to some ICPC-2 codes was lower than expected (Table 2; Fig. 3). Generally, a decline in sick leave may be influenced by changes in political policy regarding sick leave and improved workplace/public health policies [39]. The deficit in medically certified sick leave for K76 (Ischaemic heart disease without angina) contrasts with Norwegian mortality statistics, where the age-standardized mortality rate of cardiovascular disease was higher than expected in both 2022 and 2023 [40, 41]. A plausible explanation may be that the excess mortality due to cardiovascular disease has reduced the number of people alive with cardiovascular disease, and hence the sick leave numbers have reduced. For less serious ailments such as N79 (Concussion) and L81 (Injury musculoskeletal NOS), the increase of remote work may have led to fewer people seeking medically certified sick leave.

### Excess working person-years lost to medically certified sick leave

In 2023, NAV paid out 583 billion NOK / 55 billion USD in benefits, of which 61 billion NOK / 5.7 billion USD corresponded to medically certified sick leave [42]. This study estimates an economic loss of 16.5 billion NOK / 1.5 billion USD in 2023 due to excess working person-years lost to medically certified sick leave.

The excess lost person-days of work in 2020–2021 may be partially explained by COVID-19 isolation rules [43]. The excess lost person-days of work increased from 2021 to 2022, as expected due to the cessation of comprehensive measures; a similar increase in health care utilization and inability to return to work or school was seen in the highly vaccinated West Australian population following the relaxation of infectious disease protective measures [44]. The excess lost person-days of work was higher in 2023 compared to 2022, which may be indicative of an increased morbidity in the general population due to the cumulative risk of post-acute sequelae [45]. High levels of absenteeism due to illness in 2023 have also been documented in Belgium [46], Germany [47], and the UK [48].

### Lowered threshold for staying at home when ill

During 2020 and 2021, the public was advised to “stay at home when ill”. Because of this, it has been hypothesized that the excess sick leave in 2023 can be attributed to a lowered threshold for staying at home when ill. However, if this were the primary force driving the increase in medically certified sick leave, we would expect to see a flat increase across all diseases/symptoms. Instead, we see the opposite: A large increase in diseases/symptoms associated with acute and post-acute sequelae of COVID-19, and no increase or less of an increase in other diseases/symptoms.

### Limitations

The fundamental limitation of this study is that it is a study based on aggregate data analyzing temporal changes. It is not possible to draw causal conclusions from this study, and there can be many reasons for the observed temporal changes, either in part or in whole.

Another limitation of this study is the lack of SARS-CoV-2 status at an individual-level. Due to a lack of testing for SARS-CoV-2 in Norway since early 2022, this was not possible to obtain.

A limitation of the NAV data is that it lacked information on age, which may have affected the results. To ascertain the importance of the age structure on the analyses, three analyses were rerun in NorSySS data, ignoring the age structure. The effect of ignoring age was minimal (ignoring of age structure vs. Table 2 results for thousands of estimated excess consultations in 2023): R** Respiratory infections (357 vs. 359), A04 Weakness/tiredness general (150 vs. 150), and D01 Abdominal pain/cramps general (77 vs. 76). It is likely that the analysis is robust to ignoring age because age is implicitly handled by the time trend.

A further limitation of this study is the inability to capture the full extent of COVID-19 spread due to the lack of reliable community transmission data. Nevertheless, our proxy measure based on vaccine efficacy and hospitalization rates provided valuable insights, showing positive temporal correlations with a range of health conditions.

An additional limitation of this study is that only 85 ICPC-2 code combinations were available from NorSySS, limiting the ability to compare with NAV data.

### Strengths

The NAV data only contained medically certified sick leave. The number of days of self-certified sick leave allowed before a person transitions to medically certified sick leave varies by employer and collective bargaining agreement. Statistics Norway estimated that in the fourth quarter of 2023 83% of sick leave was medically certified and 17% self-certified. This study therefore has good coverage of sick leave data.

The primary strength of this paper is the breadth of the 556 ICPC-2 code combinations from the NAV data. In some cases (respiratory infections, fever, weakness/tiredness, strep throat), the NAV and NorSySS results agreed with each other, strengthening the findings. In addition, the high level of coverage in the NAV and NorSySS data provide a solid data backbone to the study.

## Conclusions

Our findings indicate significant excesses in working person-days lost to medically certified sick leave and primary healthcare consultations in 2023, deviating from the 2010–2019 trends. A sizable proportion of the excesses were due to diseases/symptoms associated with acute and post-acute sequelae of COVID-19, and the magnitudes were large enough to be of societal concern. Health economics studies are needed to evaluate the ongoing impact of COVID-19 on Norwegian society, including both acute and post-acute sequelae.

## Supplementary Information

Additional File 1. ICPC-2 codes extracted for NAV and NorSySS.

Additional File 2. Methods and results for NorSySS analyses.

Additional File 3. A) Working person-days lost to medically certified sick leave (NAV) and b) number of primary health care consultations (NorSySS) for 2022 and 2023, sex-specific results.

Additional File 4. Trends in medically certified sick leave/primary healthcare consultations where 2023 is higher or lower than expected, sex-specific results.

Additional File 5. Residual diagnostic plots for the regressions in Additional File 4.

Additional File 6. Medically certified sick leave and community spread of COVID-19 (assuming 80% and 70% vaccine effectiveness) from 2020-Q1 to 2023-Q4.

## Data Availability

The data that support the findings of this study are available from the Norwegian Labour and Welfare Administration and the Norwegian Syndromic Surveillance System, which were used under license for the current study, and so are not publicly available. Applications for these data may be made to the Norwegian Labour and Welfare Administration (NAV) and the Norwegian Syndromic Surveillance System (NorSySS).

## Abbreviations

COVID-19: Coronavirus disease 2019
FDR: False discovery rate
ICPC-2: International classification of primary care version 2
KPR: Municipal patient and user register
Norwegian: Kommunalt pasient- og brukerregister
KUHR: Control and payment of health reimbursements
Norwegian: Kontroll og utbetaling av helserefusjoner
NAV: Norwegian labour and welfare administration
Norwegian: Arbeids- og velferdsforvaltninga
NIPH: Norwegian institute of public health
Norwegian: Folkehelseinstituttet
NorSySS: Norwegian syndromic surveillance system
Norwegian: Det norske syndromiske overvåkingssystemet
PCR: Polymerase chain reaction
PI: Prediction interval
RNA: Ribonucleic acid
SARS-CoV-2: Severe acute respiratory syndrome coronavirus 2
