# Supplementary material for: Aberrations in medically certified sick leave and primary healthcare consultations in Norway in 2023 compared to pre-COVID-19-pandemic trends
Source: Arch Public Health. 2024 Oct 22;82:187. doi: 10.1186/s13690-024-01411-4 (PMC11495095; doi:10.1186/s13690-024-01411-4)

**Methods and results for NorSySS analyses**

**Comparing 2023 against a 2010-2019 baseline**

From NorSySS, data was extracted for 85 ICPC-2 code combinations (Supplemental Table 1, Additional file 1), representing the number of primary healthcare consultations from 2010 to 2023. Data was extracted for males, females, and all sexes combined. Ages 0-4, 5-14, 20-29, 30-64, 65-69, 70-79, and 80+ were extracted. Data for 15-19-year-olds were excluded due to unreliability due to frequent legislative changes regarding school attendance requiring 15-19-year-olds to obtain doctor’s notes for absences. These numbers were then rescaled to 2023-population levels.

The aim of this analysis was to use the data from 2010-2019 to predict expected baselines for 2020-2023, then calculate the excess values for 2020-2023 by subtracting the observed values from the expected baselines.

To calculate the expected/excess values for 2020 to 2023, one analysis was performed for each combination of: male/female/all sexes, and each ICPC-2 code combination.

For NorSySS, to investigate the appropriate model for the expected baseline, three linear regressions were performed on data between 2010-2019:

1. Outcome: Rate/100k, Covariate: Year as a continuous linear variable.
2. Outcome: Rate/100k, Covariate: Year as a cubic spline with two degrees of freedom.
3. Outcome: Rate/100k, Covariate: Year as a cubic spline with three degrees of freedom.

Each of the models contained an interaction term with categorical age.

The model with the lowest AIC was selected, and then a Bayesian linear regression was performed using the selected model between 2010-2019, with 4 chains each containing 20000 iterations. The Bayesian linear regression was implemented using the “rstan” package in R, which uses gradient-based Markov chain Monte Carlo algorithms (19,20). The expected baseline for 2020 to 2023 was then calculated by estimating the posterior of the rate/100k. The expected baselines for 2020-2023 were then used to calculate the excess values and corresponding prediction intervals.

The excess values were then restricted to 2022 and 2023 and corrected for multiple testing using false discovery rates (FDR) with a threshold of 0.05. After FDR correction, significant results with an absolute excess value less than 10,000 were discarded due to not being clinically relevant.

**Temporal association with community spread of COVID-19 between 2020 and 2023**

For NorSySS, the outcome was the number of primary healthcare consultations for the desired ICPC-2 code combinations. These data were then scaled into rates using the total population, and then aggregated into isoquarters (isoweeks 1-13, 14-26, 27-39, 40-52) by isoyear (2020, 2021, 2022, 2023). The Pearson correlation was then calculated for each ICPC-2 code combination against the proxy for community spread of COVID-19.

The p-values corresponding to the Pearson correlations for both NAV and NorSySS were then corrected for multiple testing using false discovery rates with a threshold of 0.05. Those that were found to be under the threshold were then graphically displayed as quarterly timeseries.

**Supplementary Table 2.** Primary healthcare consultations in 2023 for all sexes combined (NorSySS).

| **ICPC-2** | **Observed** | **Excess** | |
| --- | --- | --- | --- |
|  | **Value in 1000s** | **Value (90% PI) in 1000s** | **Ratio (90% PI)** |
| R** Respiratory infections | 1953 | 359 (227 to 490)* | 1.23 (1.13 to 1.33)* |
| A04 Weakness/tiredness general | 424 | 150 (139 to 160)* | 1.55 (1.49 to 1.61)* |
| D01 Abdominal pain/cramps general | 348 | 76 (61 to 92)* | 1.28 (1.21 to 1.36)* |
| D11+D70+D73 Gastroenteritis | 174 | 44 (27 to 61)* | 1.33 (1.18 to 1.54)* |
| A78 Infectious disease other/NOS | 76 | 42 (28 to 55)* | 2.21 (1.59 to 3.57)* |
| R96 Asthma | 147 | 38 (19 to 57)* | 1.35 (1.14 to 1.63)* |
| R72 Strep throat | 61 | 28 (22 to 34)* | 1.86 (1.58 to 2.26)* |
| A03 Fever | 91 | 28 (16 to 40)* | 1.44 (1.21 to 1.78)* |
| S29 Skin symptom/complaint other | 118 | 24 (13 to 35)* | 1.26 (1.12 to 1.42)* |
| R21 Throat symptom/complaint | 95 | 24 (17 to 31)* | 1.34 (1.22 to 1.48)* |
| D73 Gastroenteritis presumed infection | 70 | 23 (12 to 34)* | 1.50 (1.21 to 1.96)* |
| D11 Diarrhea | 85 | 22 (15 to 29)* | 1.35 (1.22 to 1.51)* |
| R75 Sinusitis acute/chronic | 92 | 21 (17 to 26)* | 1.30 (1.23 to 1.38)* |
| D09 Nausea | 43 | 16 (14 to 19)* | 1.59 (1.46 to 1.75)* |
| D99 Disease digestive system, other | 41 | 12 (7 to 17)* | 1.42 (1.22 to 1.69)* |
| D18 Change feces/bowel movements | 24 | 10 (6 to 15)* | 1.80 (1.36 to 2.64)* |
| H01 Ear pain/earache | 23 | 10 (7 to 14)* | 1.84 (1.41 to 2.65)* |
| A05 Feeling ill | 25 | -14 (-16 to -12)* | 0.64 (0.61 to 0.69)* |

ICPC-2 codes shown are those where the excess was statistically significantly (for either all sexes, male, or female) after false discovery rate correction with a 5% cutoff and the absolute value of excess was larger than 10,000. Excess is defined as (observed minus baseline) and presented as point estimate (90% prediction interval). Sex-specific results and results from 2022 are shown in Additional File 3. Data from NorSySS. *Significant after false discovery rate correction with a 5% cutoff.

**Supplementary Figure 1.** Selected trends in primary healthcare consultations where 2023 is higher or lower than expected.


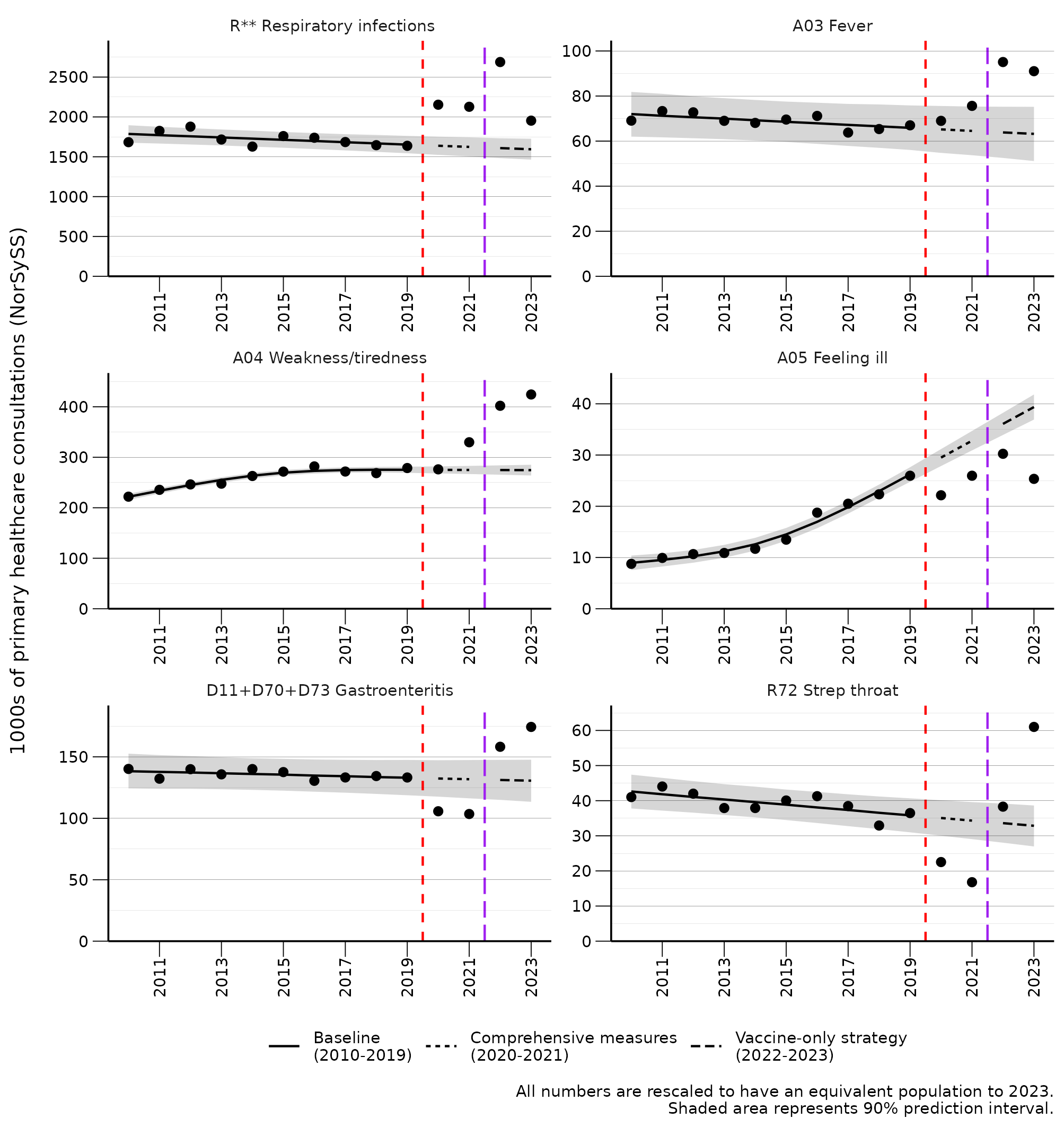
Selected trends are shown here. All significant trends, including sex-specific results, are shown in Additional file 4.

**Supplementary Figure 2.** Primary healthcare consultations and community spread of COVID-19 (assuming 90% vaccine effectiveness) from 2020-Q1 to 2023-Q4.


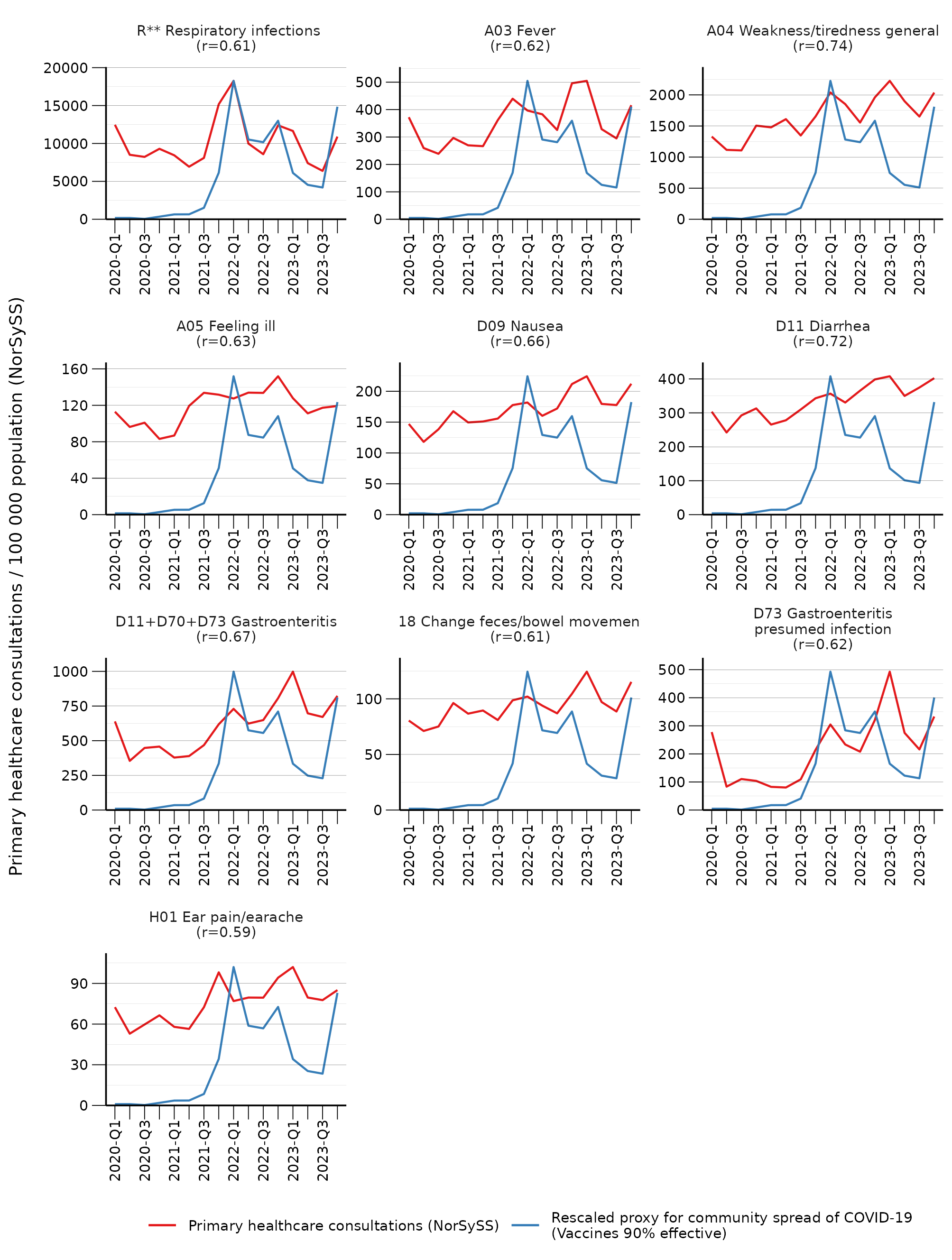


**Supplementary Figure 3.** Primary healthcare consultations and community spread of COVID-19 (assuming 80% vaccine effectiveness) from 2020-Q1 to 2023-Q4.


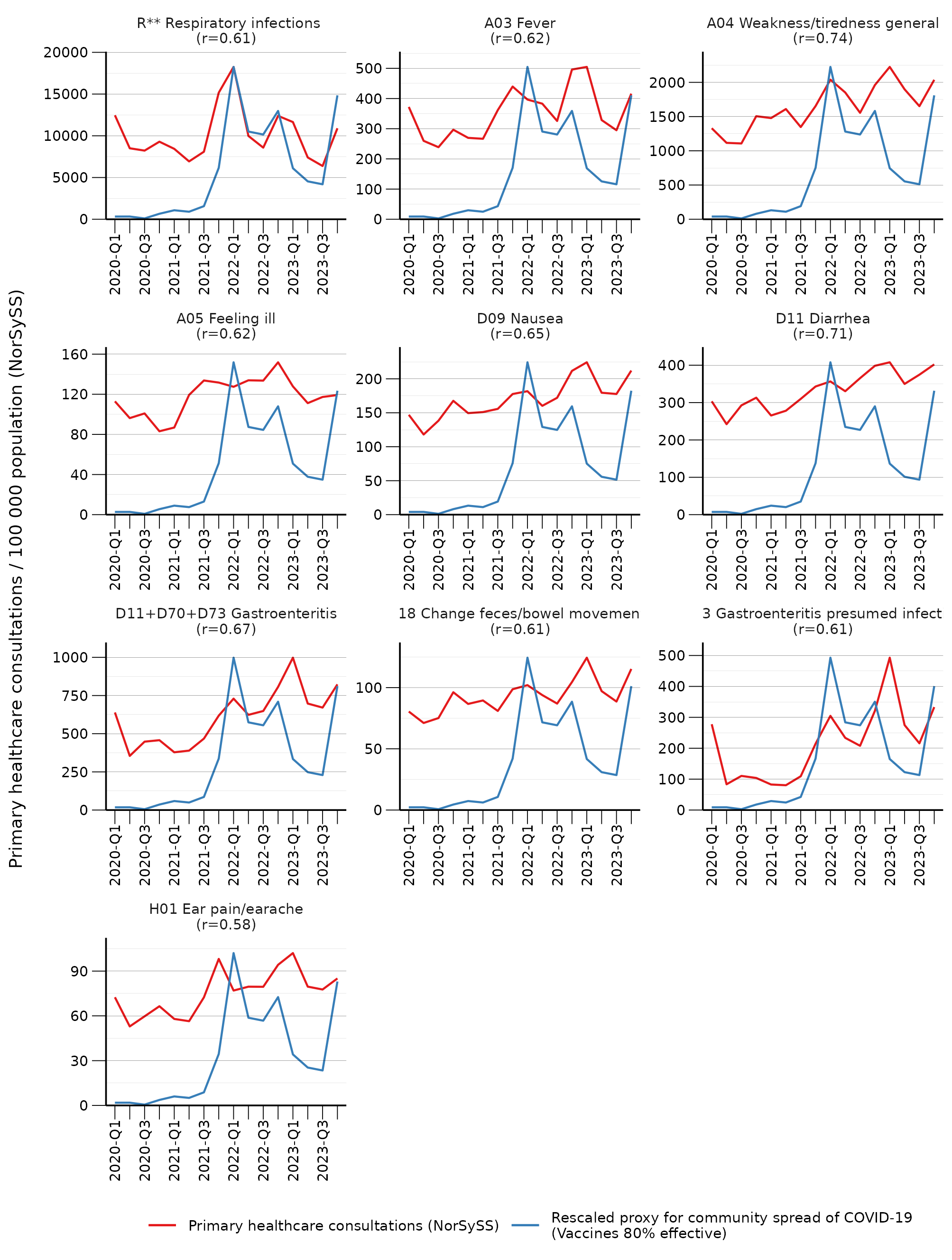


**Supplementary Figure 4.** Primary healthcare consultations and community spread of COVID-19 (assuming 70% vaccine effectiveness) from 2020-Q1 to 2023-Q4.


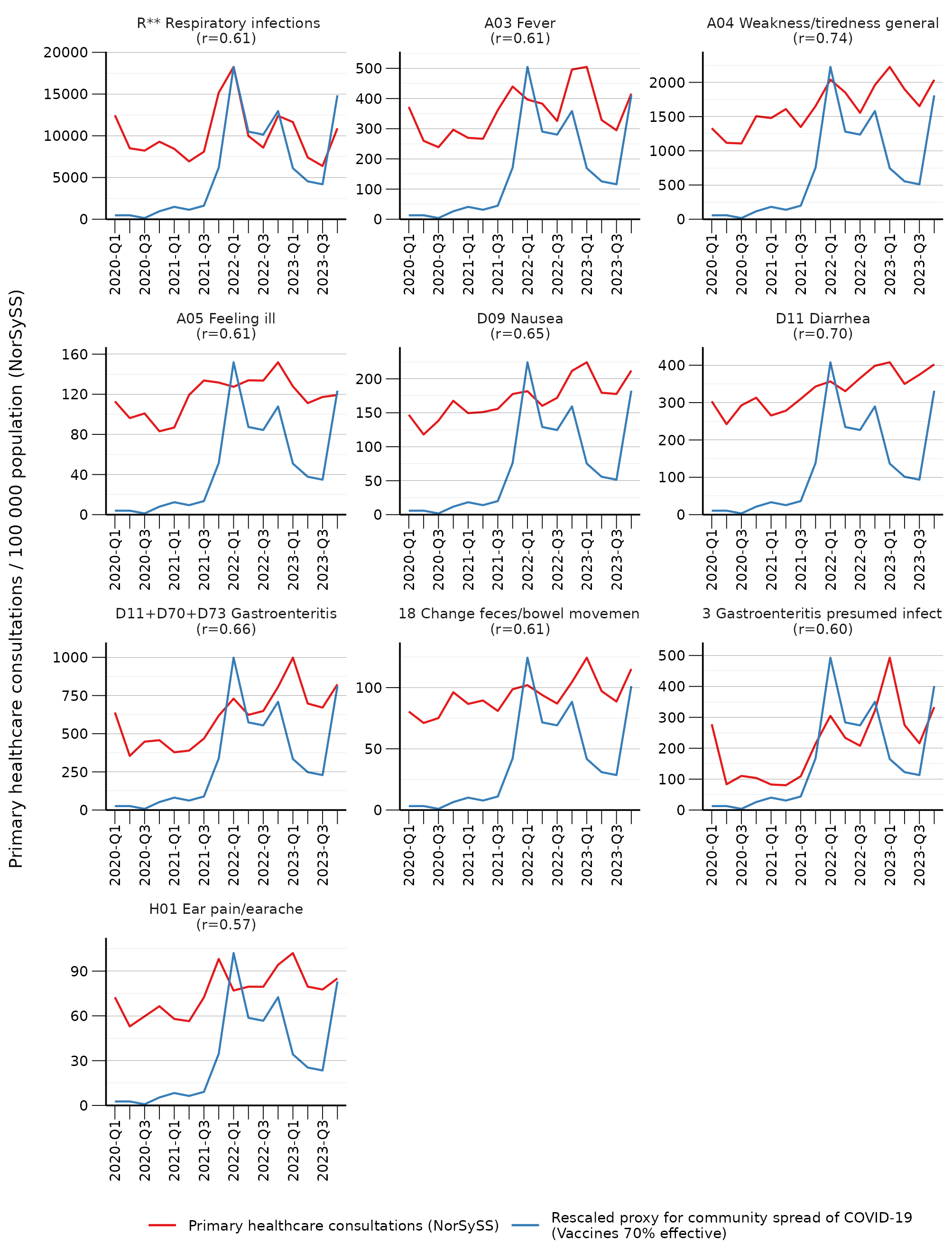

Supplement: Supplementary file 2 — Additional File 2. Methods and results for NorSySS analyses. [file 13690_2024_1411_MOESM2_ESM.docx]
