# Supplementary material for: Aberrations in medically certified sick leave and primary healthcare consultations in Norway in 2023 compared to pre-COVID-19-pandemic trends
Source: Arch Public Health. 2024 Oct 22;82:187. doi: 10.1186/s13690-024-01411-4 (PMC11495095; doi:10.1186/s13690-024-01411-4)
Supplement: Supplementary file 4 — Additional File 4. Trends in medically certified sick leave/primary healthcare consultations where 2023 is higher or lower than expected, sex-specific results. [file 13690_2024_1411_MOESM4_ESM.pdf]

a. NAV: A* General and unspecified

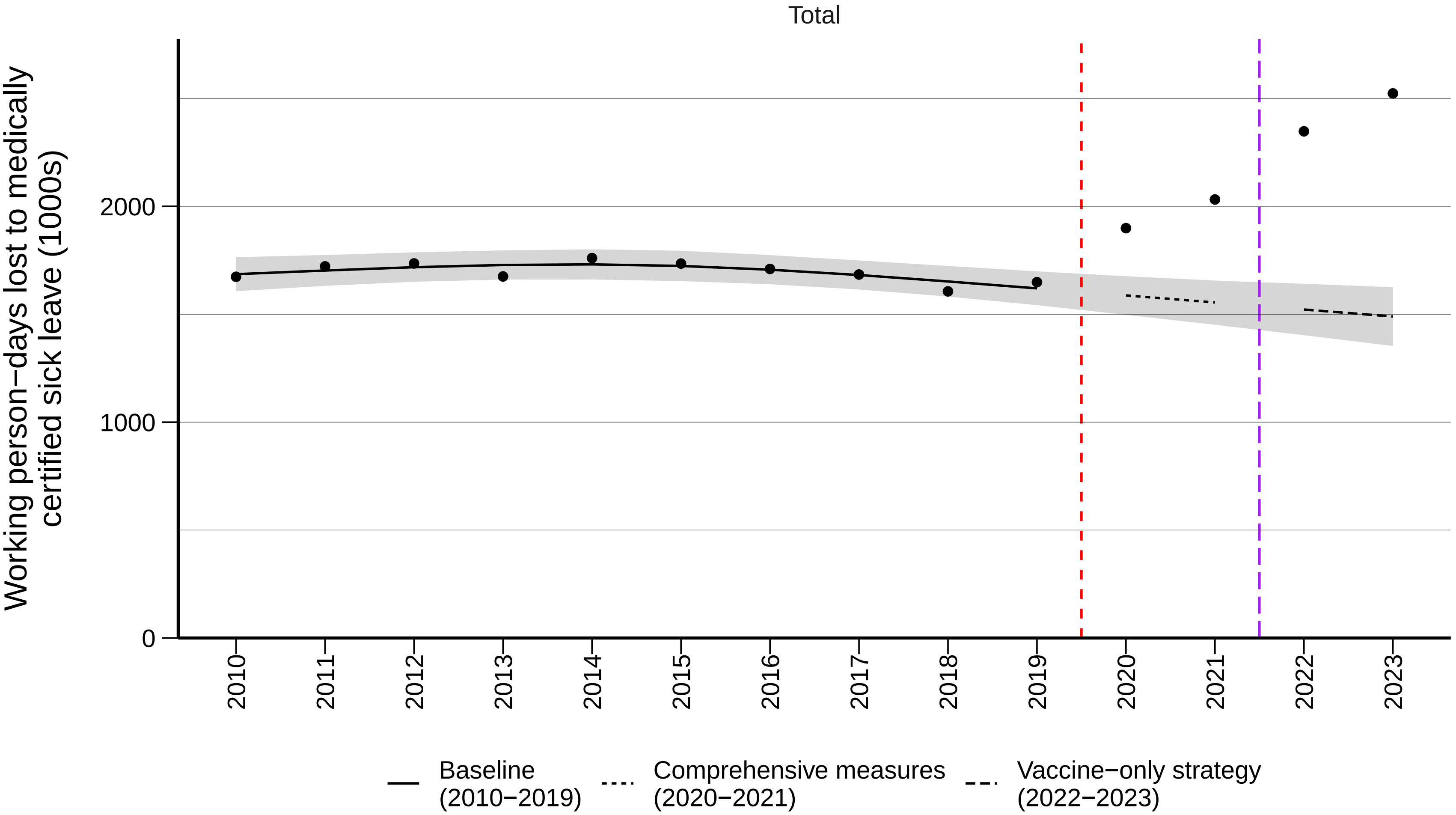

All numbers are rescaled to have an equivalent population to 2023.
Shaded area represents 90% prediction interval.

b. NAV: A* General and unspecified

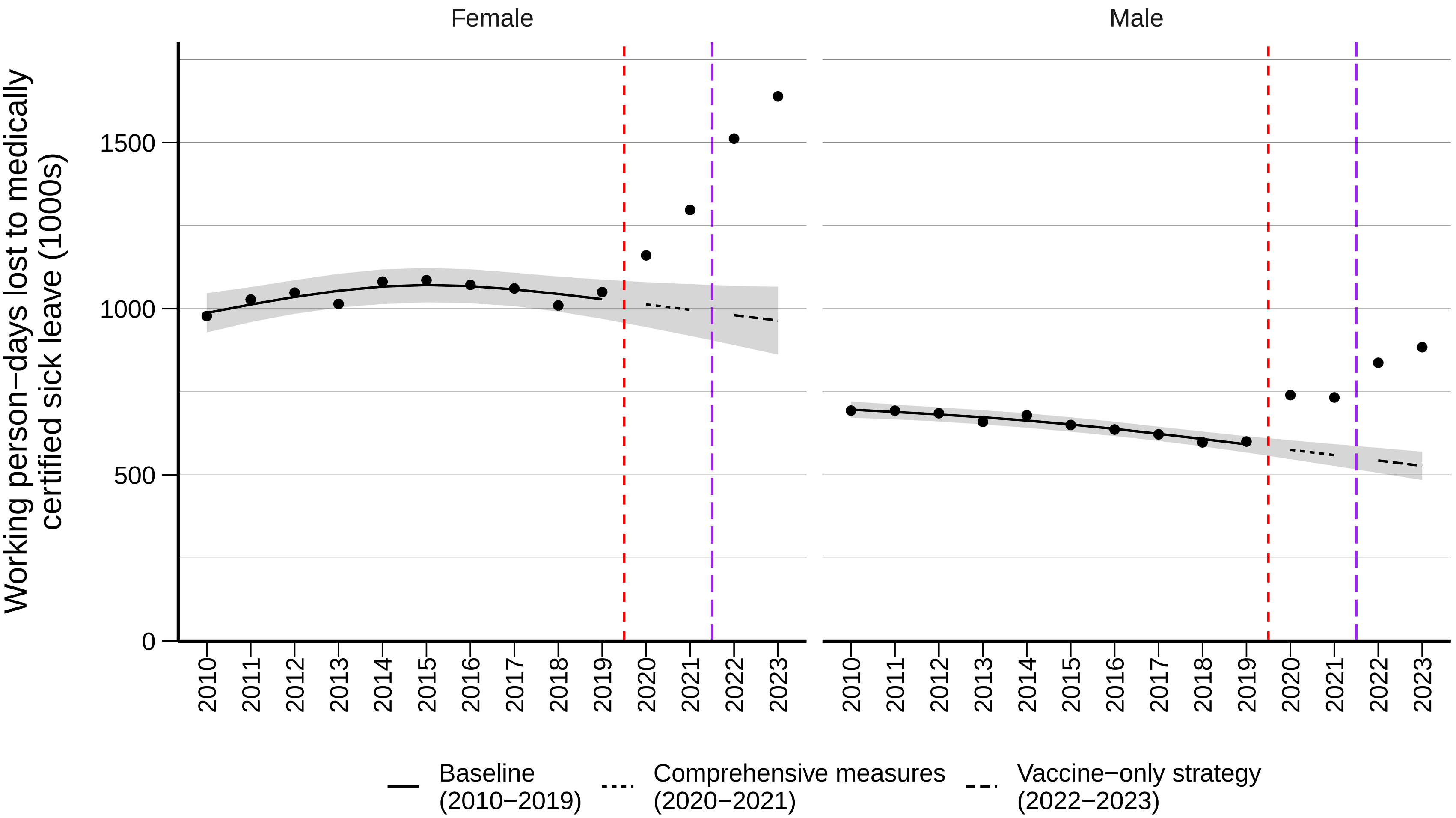

All numbers are rescaled to have an equivalent population to 2023.
Shaded area represents 90% prediction interval.

c. NAV: A01 Pain general/multiple sites

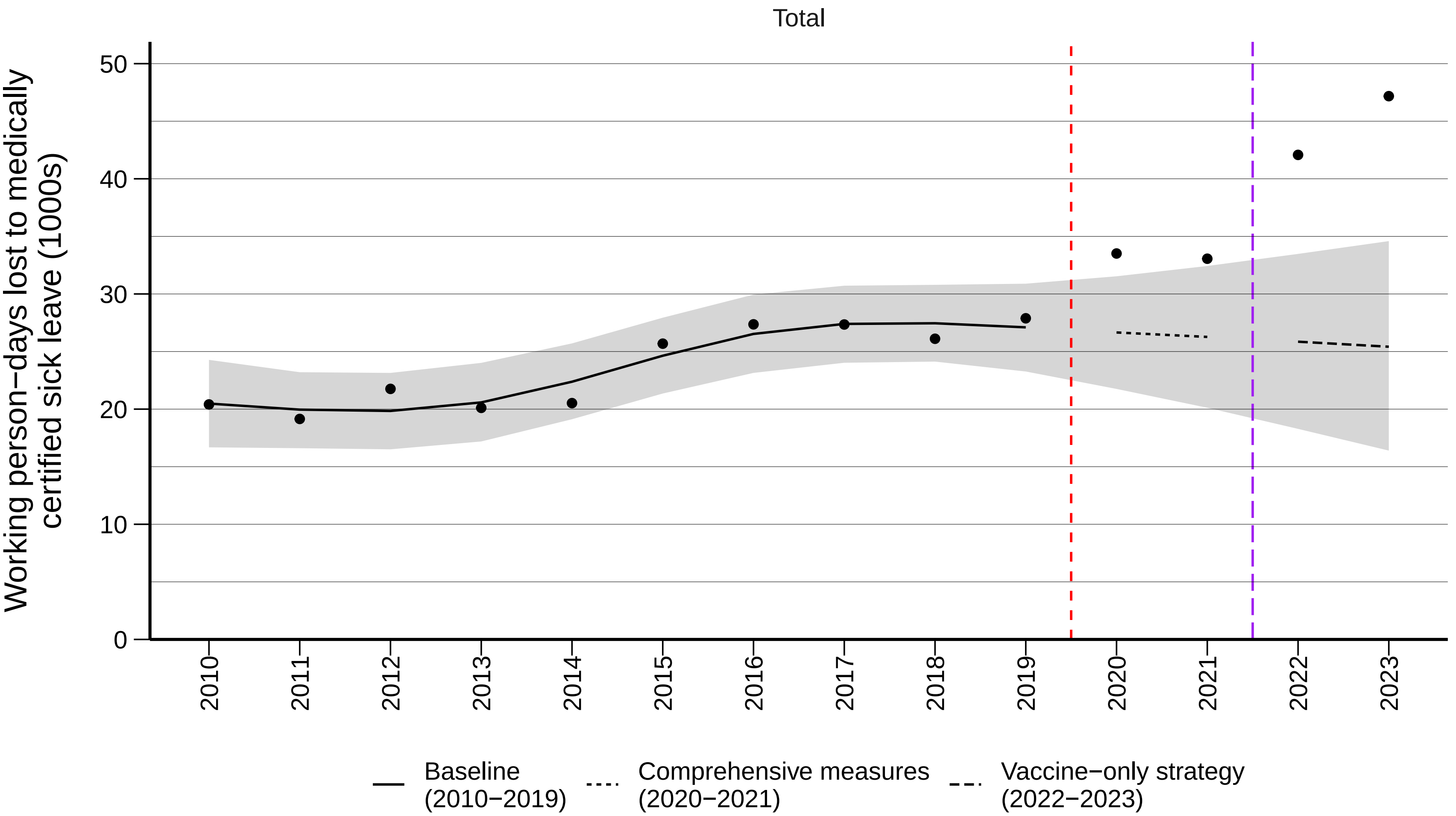

All numbers are rescaled to have an equivalent population to 2023.
Shaded area represents 90% prediction interval.

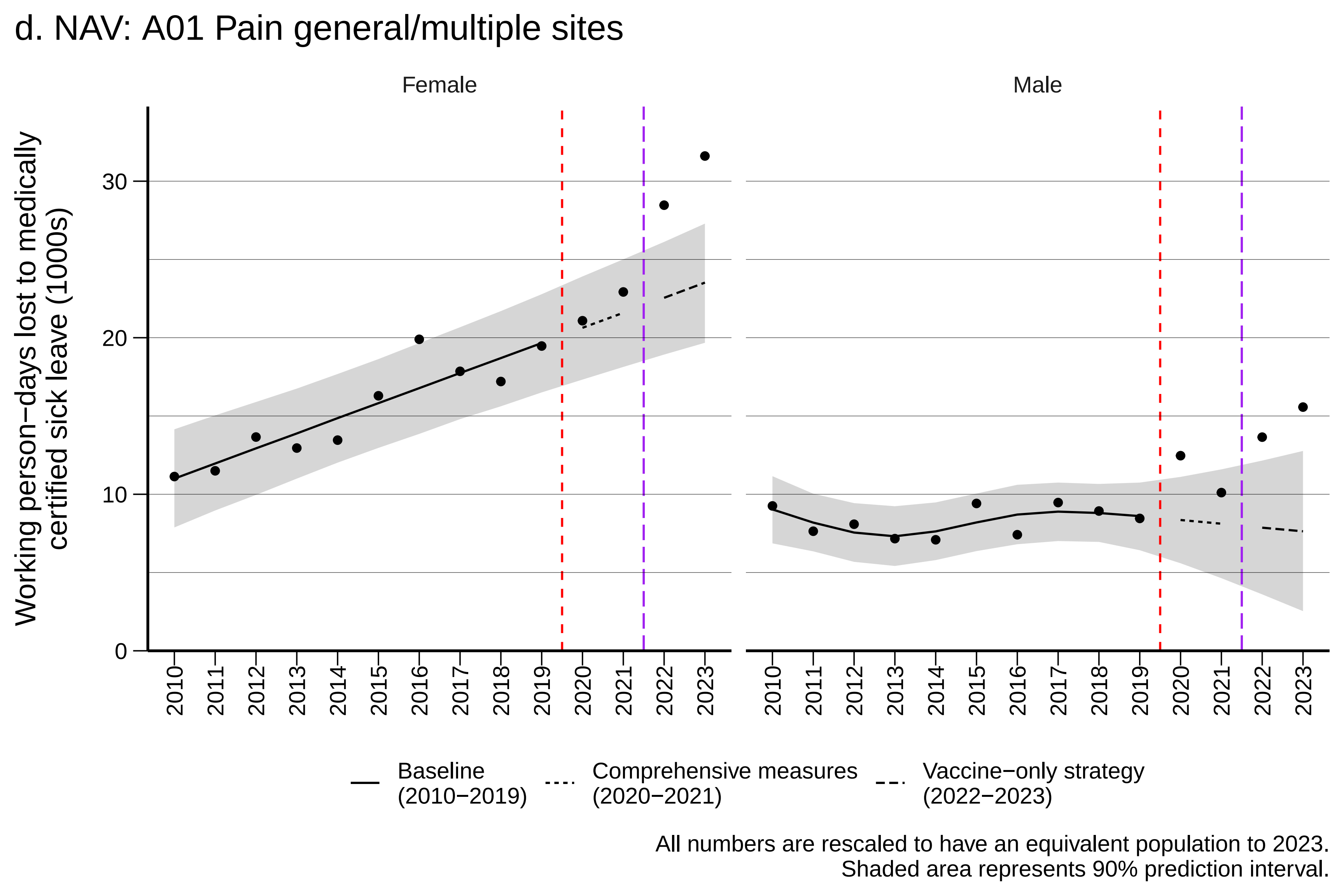
d. NAV: A01 Pain general/multiple sites
Female
Male
Working person−days lost to medically certified sick leave (1000s)
0
10
20
30
2010
2011
2012
2013
2014
2015
2016
2017
2018
2019
2020
2021
2022
2023
Baseline (2010−2019)
Comprehensive measures (2020−2021)
Vaccine−only strategy (2022−2023)
All numbers are rescaled to have an equivalent population to 2023.
Shaded area represents 90% prediction interval.

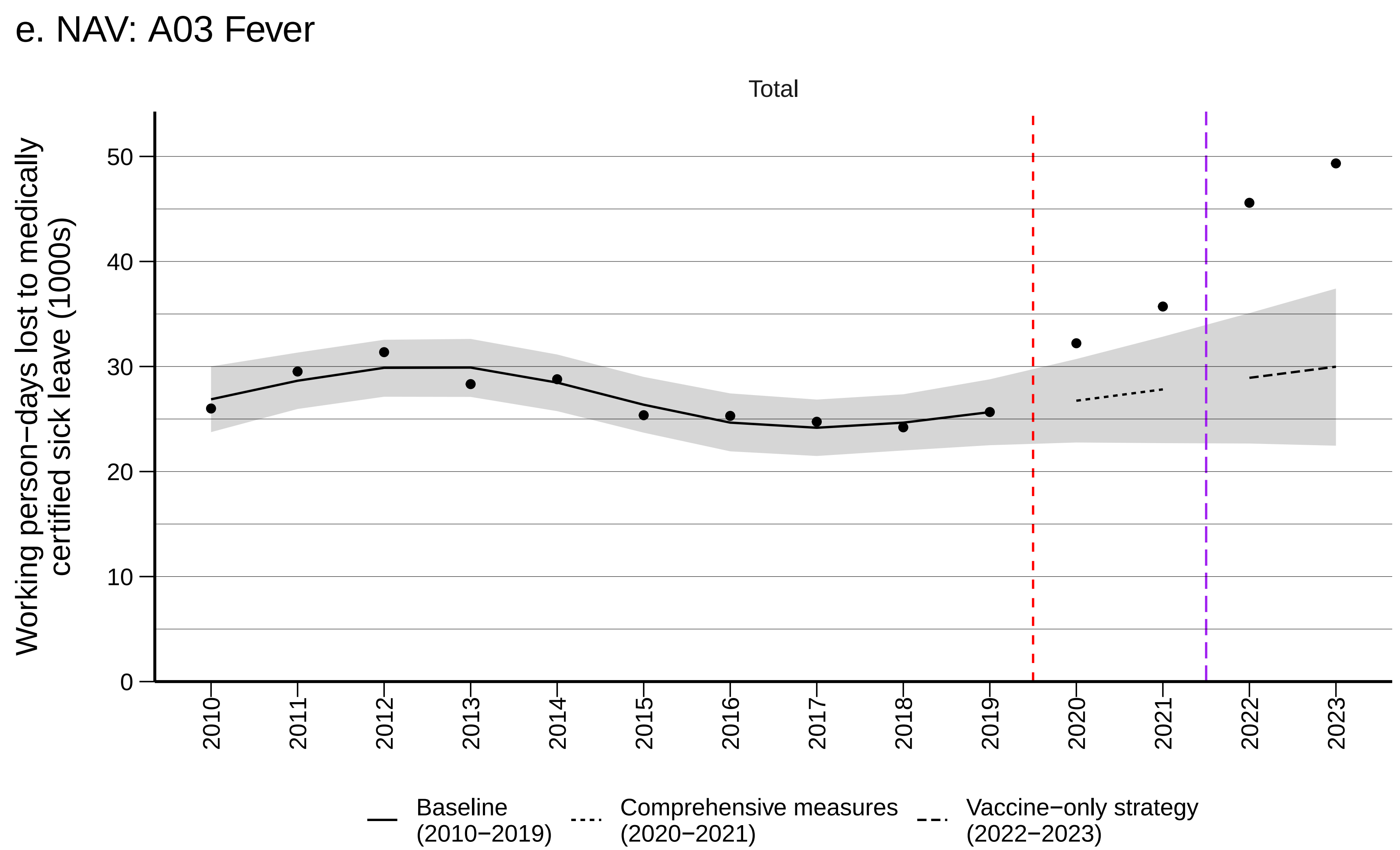

All numbers are rescaled to have an equivalent population to 2023.
Shaded area represents 90% prediction interval.

f. NAV: A03 Fever

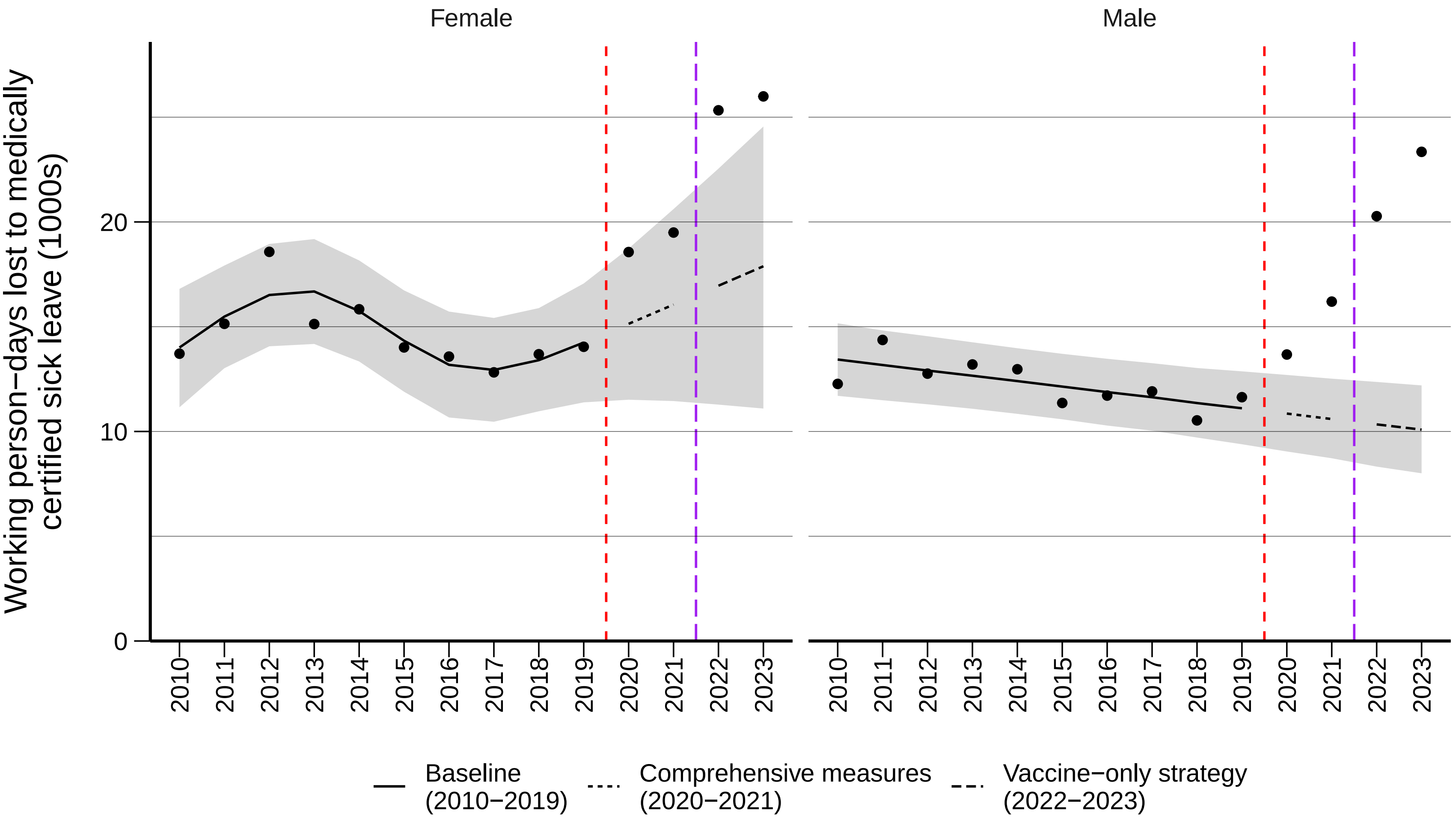

All numbers are rescaled to have an equivalent population to 2023.
Shaded area represents 90% prediction interval.

g. NAV: A04 Weakness/tiredness general

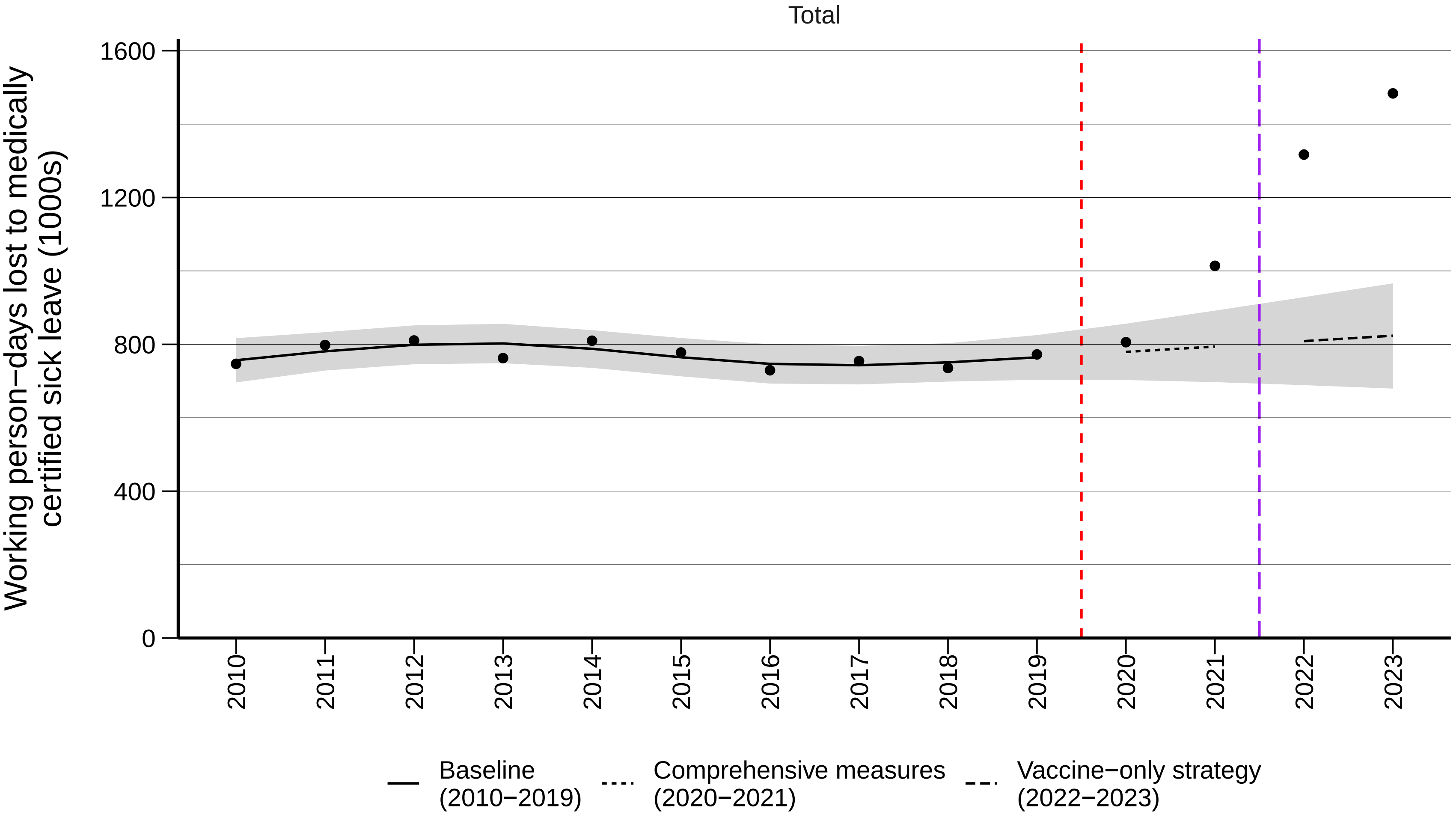

All numbers are rescaled to have an equivalent population to 2023.
Shaded area represents 90% prediction interval.

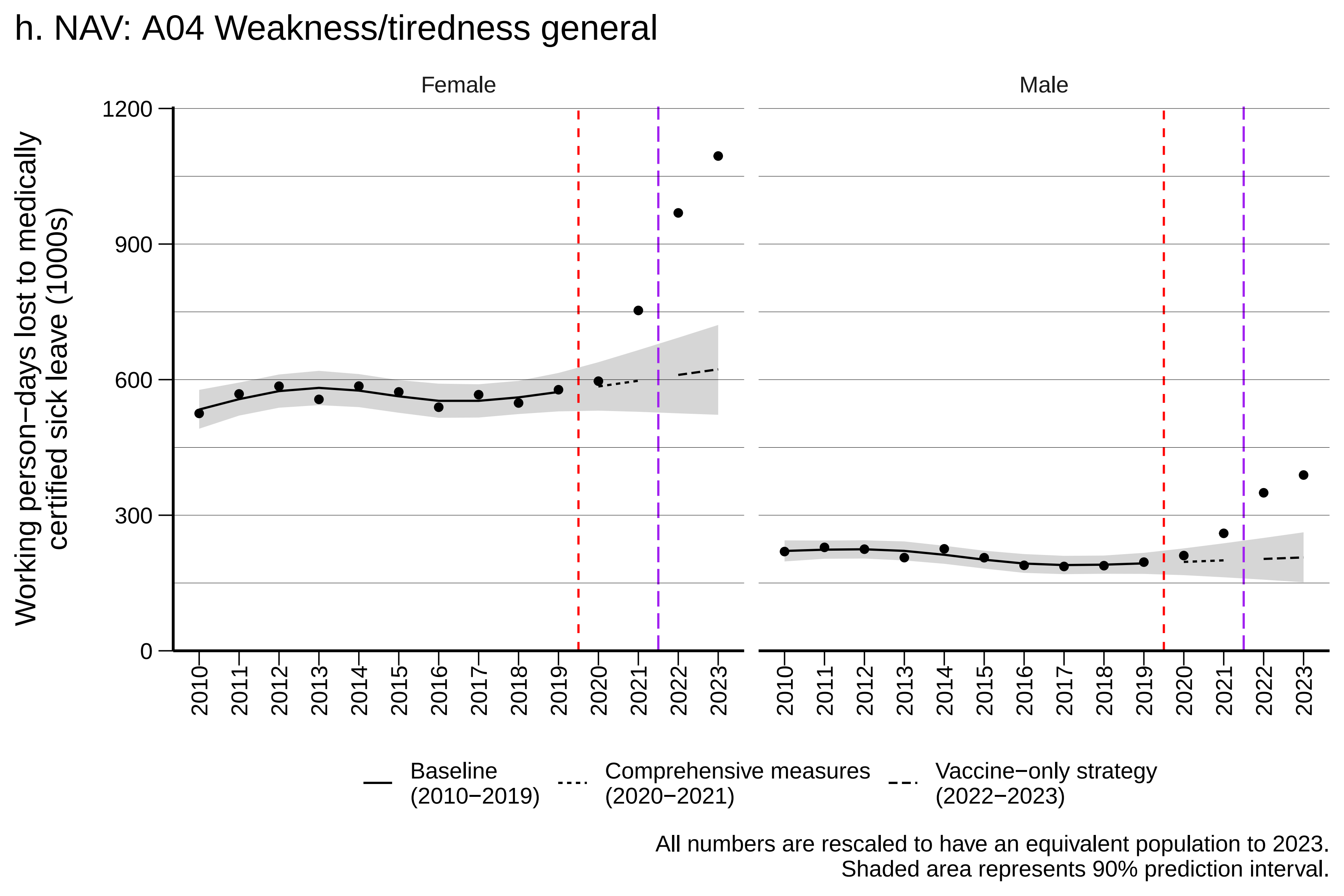
h. NAV: A04 Weakness/tiredness general
Female
Male
Working person−days lost to medically certified sick leave (1000s)
0
300
600
900
1200
2010
2011
2012
2013
2014
2015
2016
2017
2018
2019
2020
2021
2022
2023
Baseline (2010−2019)
Comprehensive measures (2020−2021)
Vaccine−only strategy (2022−2023)
All numbers are rescaled to have an equivalent population to 2023.
Shaded area represents 90% prediction interval.

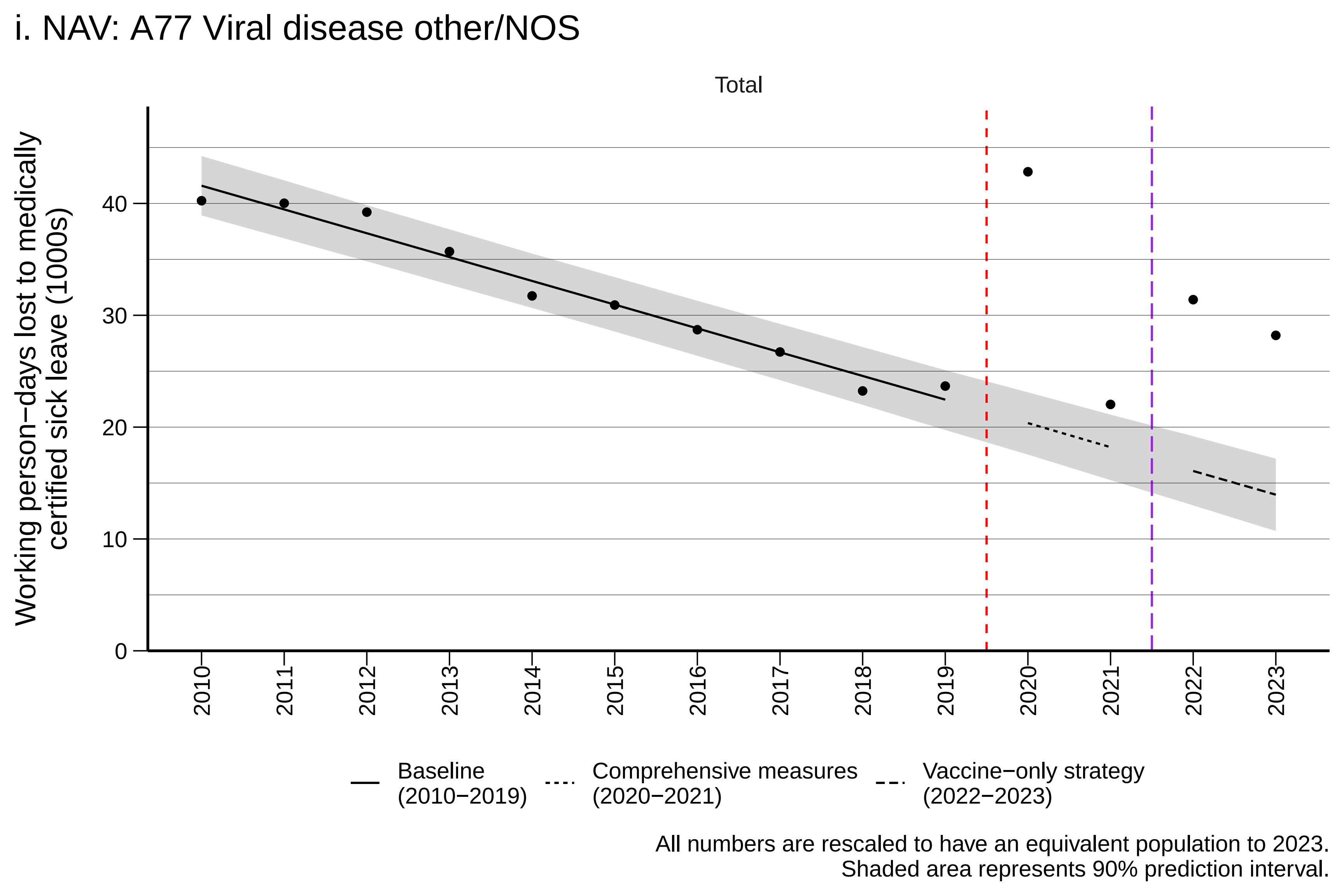

i. NAV: A77 Viral disease other/NOS
Total
Working person−days lost to medically certified sick leave (1000s)
0
10
20
30
40
2010
2011
2012
2013
2014
2015
2016
2017
2018
2019
2020
2021
2022
2023
Baseline (2010−2019)
Comprehensive measures (2020−2021)
Vaccine−only strategy (2022−2023)
All numbers are rescaled to have an equivalent population to 2023.
Shaded area represents 90% prediction interval.

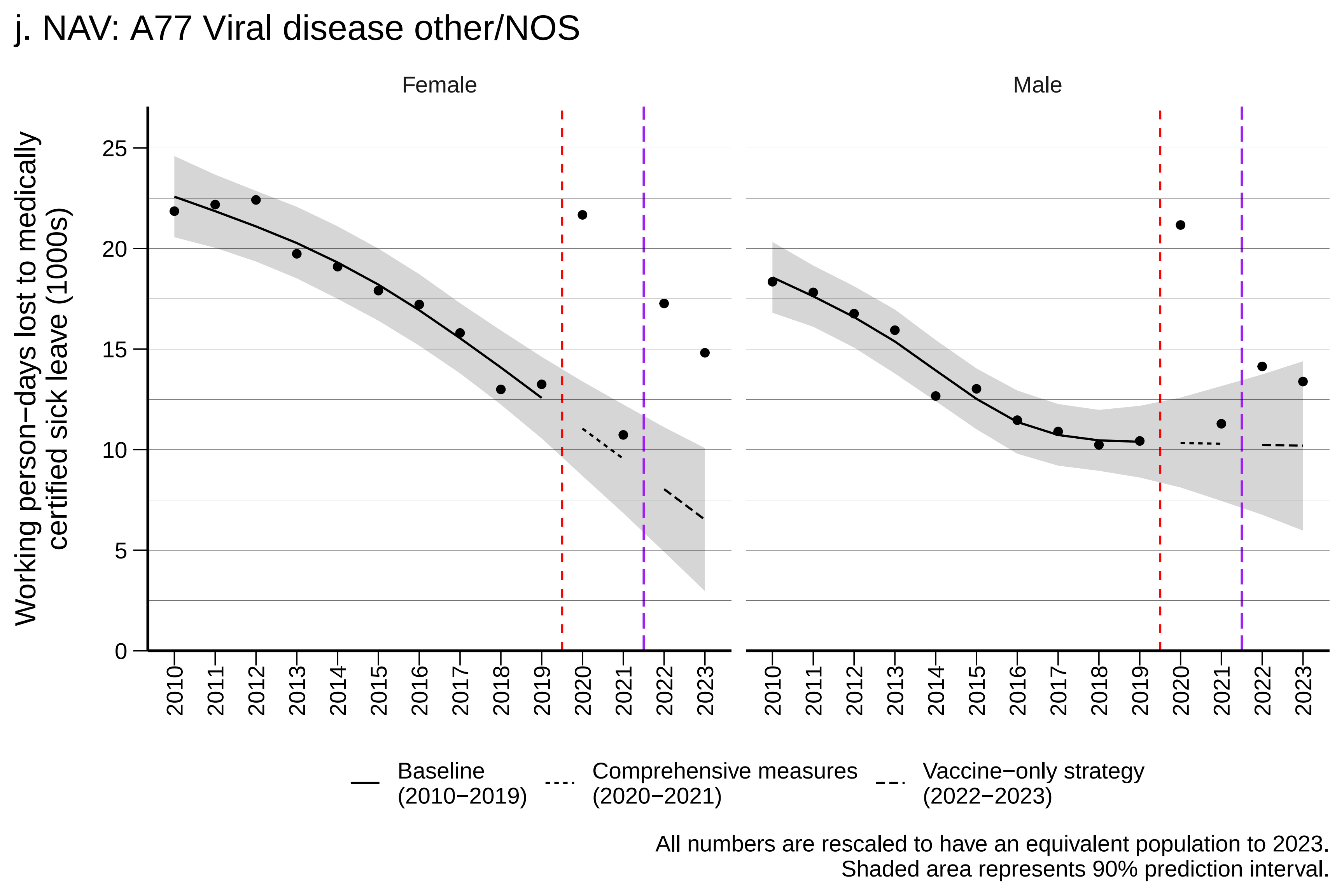
j. NAV: A77 Viral disease other/NOS
Female
Male
Working person−days lost to medically certified sick leave (1000s)
0
5
10
15
20
25
2010
2011
2012
2013
2014
2015
2016
2017
2018
2019
2020
2021
2022
2023
Baseline (2010−2019)
Comprehensive measures (2020−2021)
Vaccine−only strategy (2022−2023)
All numbers are rescaled to have an equivalent population to 2023.
Shaded area represents 90% prediction interval.

k. NAV: A99 General disease NOS

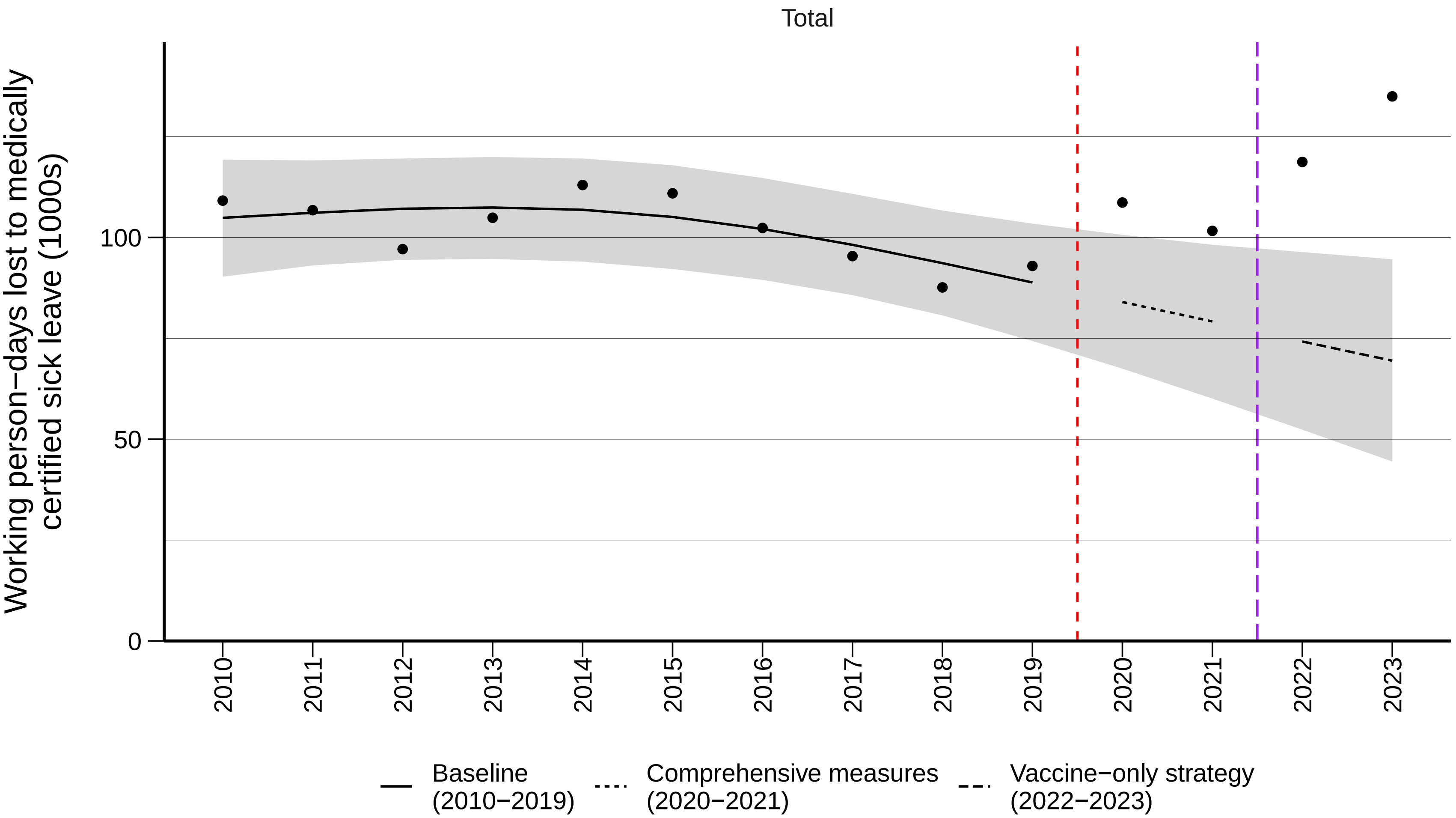

All numbers are rescaled to have an equivalent population to 2023.
Shaded area represents 90% prediction interval.

I. NAV: A99 General disease NOS

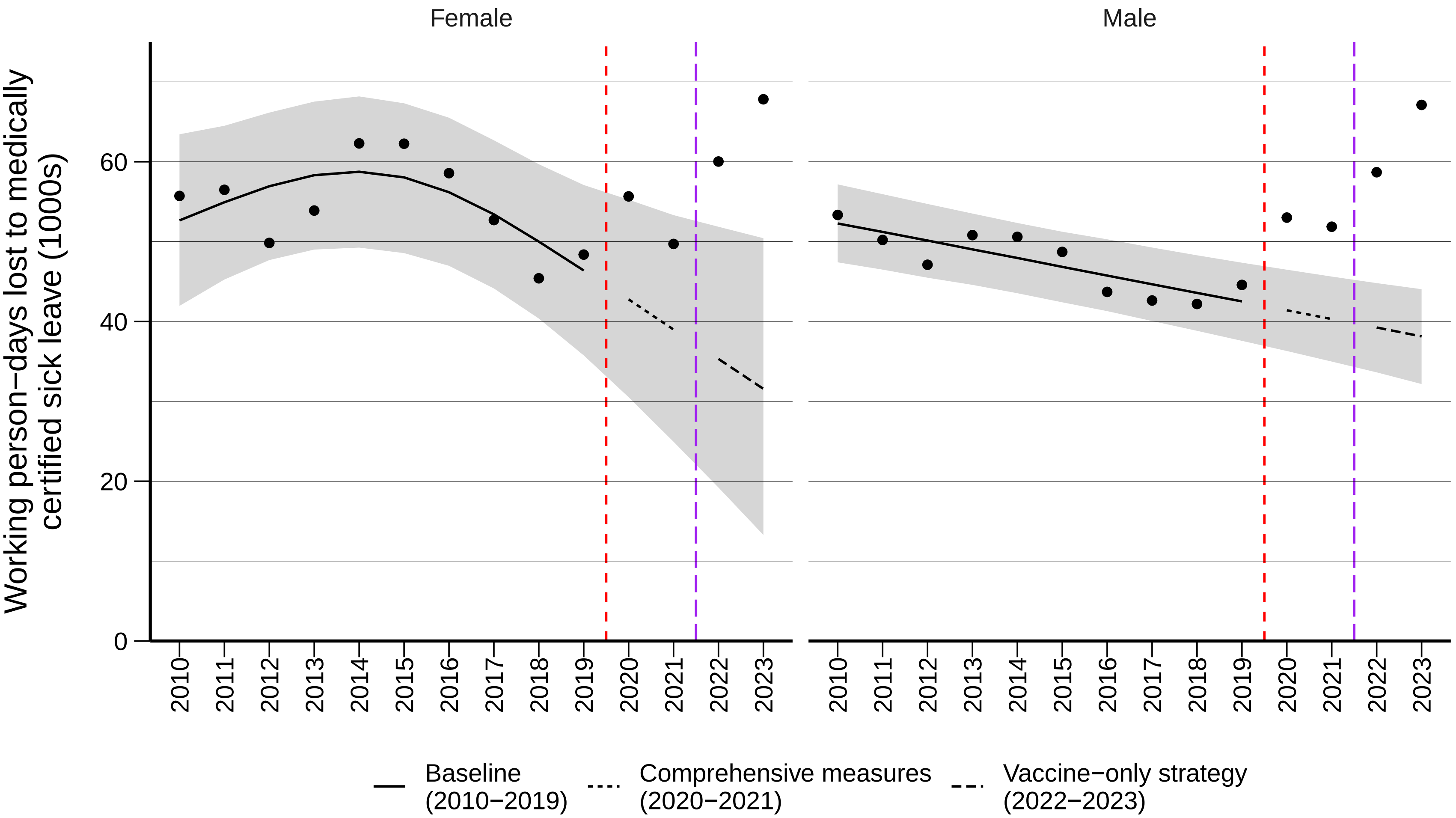

All numbers are rescaled to have an equivalent population to 2023.
Shaded area represents 90% prediction interval.

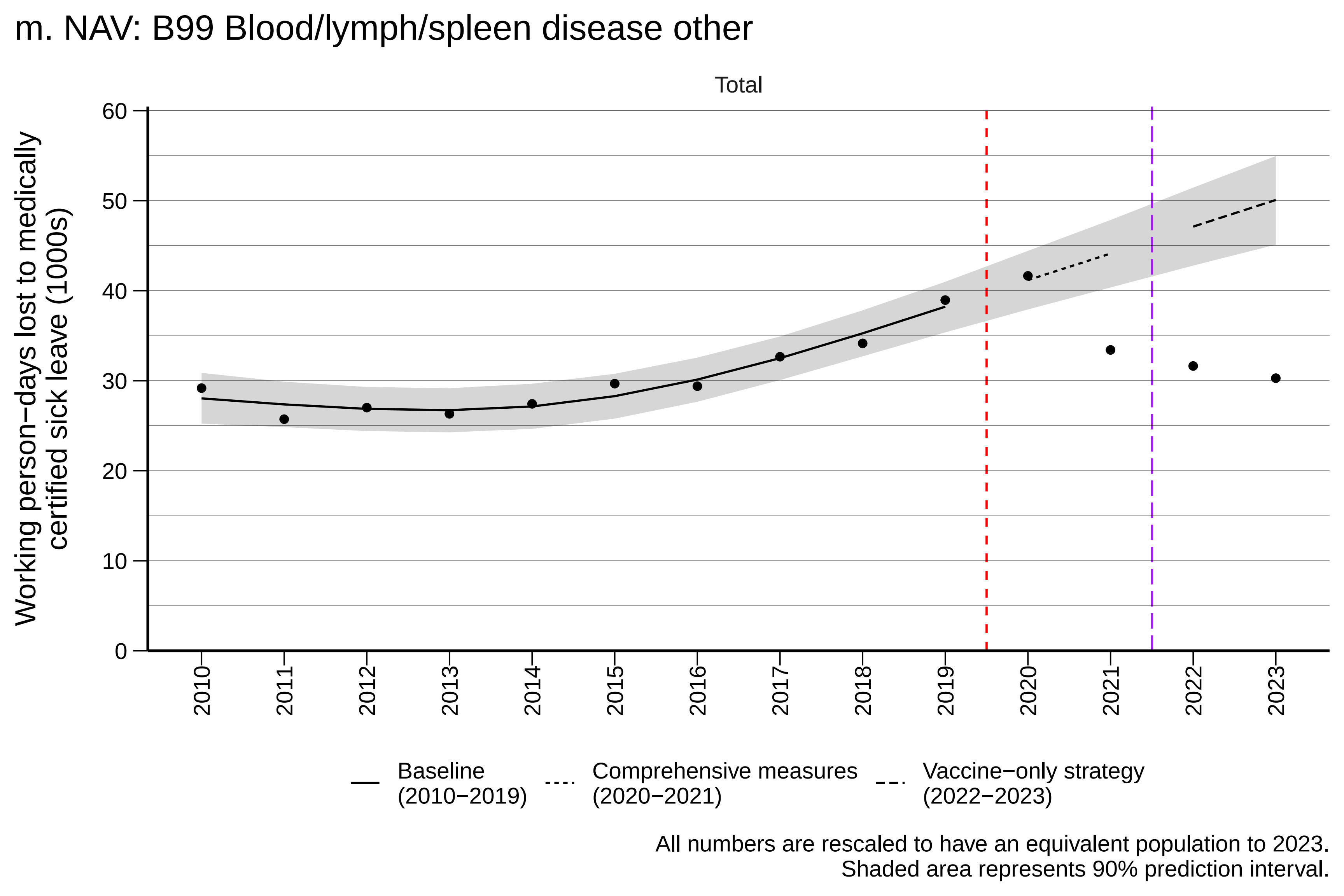
m. NAV: B99 Blood/lymph/spleen disease other
Total
Working person−days lost to medically certified sick leave (1000s)
0
10
20
30
40
50
60
2010
2011
2012
2013
2014
2015
2016
2017
2018
2019
2020
2021
2022
2023
Baseline (2010−2019)
Comprehensive measures (2020−2021)
Vaccine−only strategy (2022−2023)
All numbers are rescaled to have an equivalent population to 2023.
Shaded area represents 90% prediction interval.

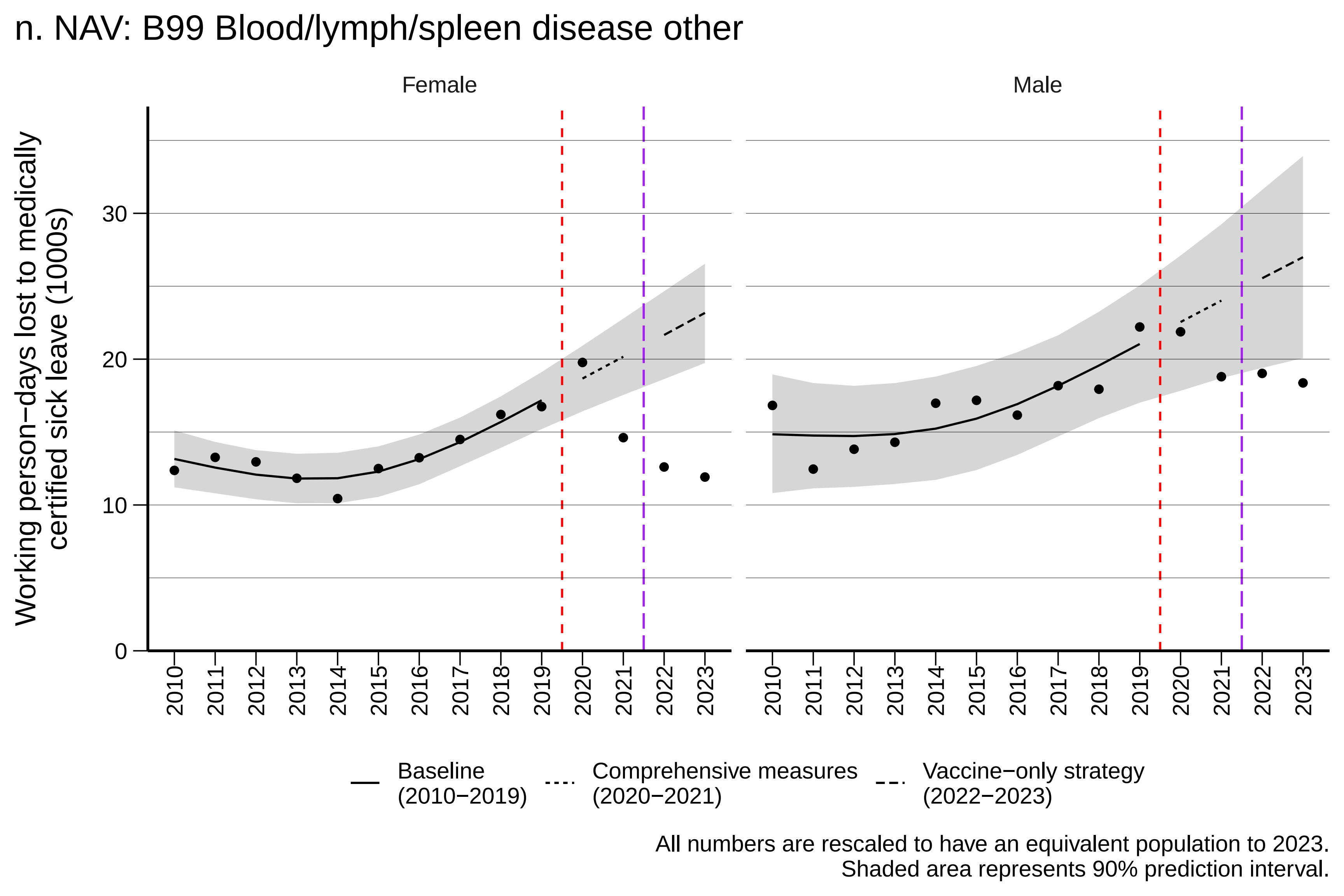
n. NAV: B99 Blood/lymph/spleen disease other
Female
Male
Working person−days lost to medically certified sick leave (1000s)
0
10
20
30
2010
2011
2012
2013
2014
2015
2016
2017
2018
2019
2020
2021
2022
2023
Baseline (2010−2019)
Comprehensive measures (2020−2021)
Vaccine−only strategy (2022−2023)
All numbers are rescaled to have an equivalent population to 2023.
Shaded area represents 90% prediction interval.

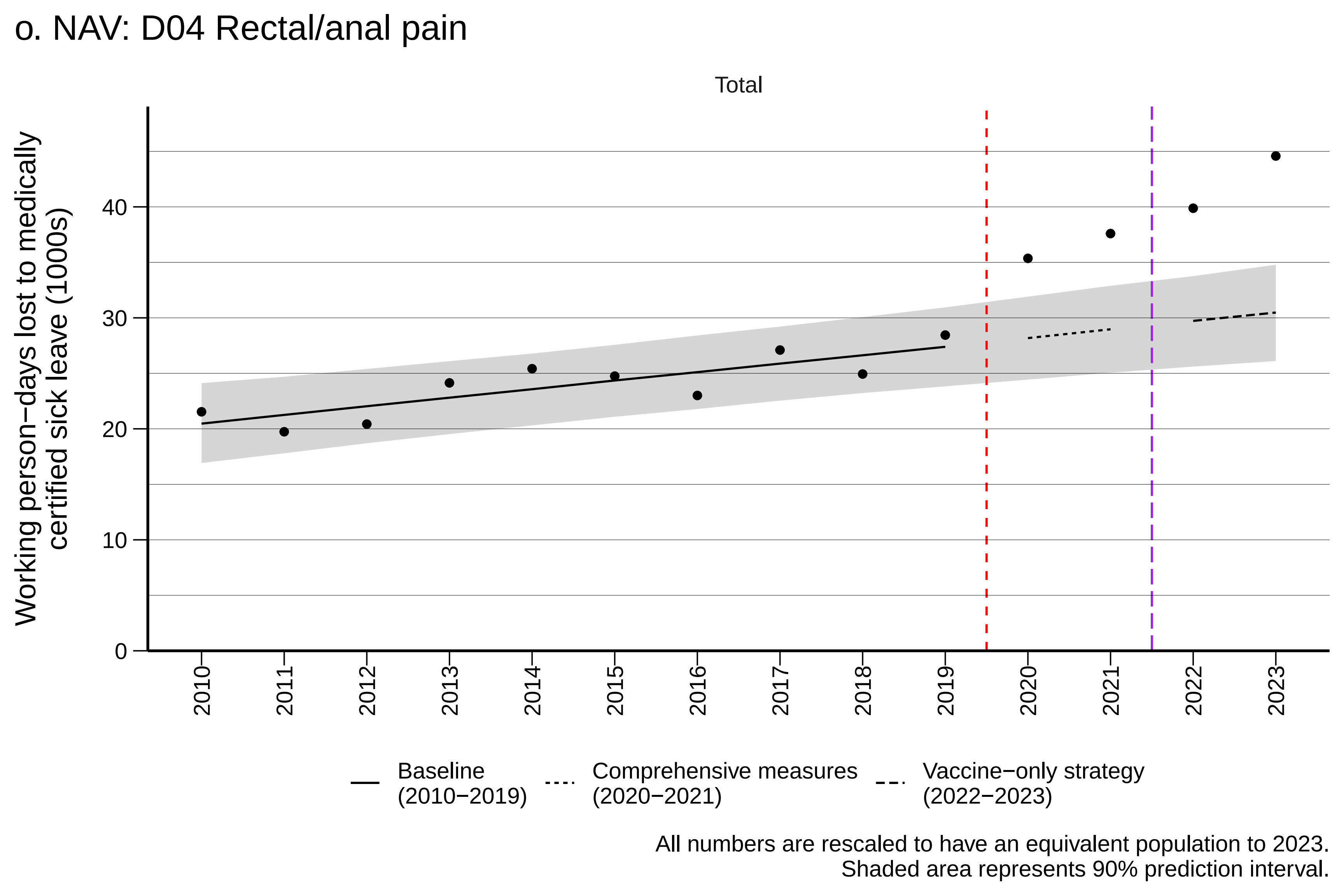

o. NAV: D04 Rectal/anal pain
Total
Working person−days lost to medically certified sick leave (1000s)
0
10
20
30
40
2010
2011
2012
2013
2014
2015
2016
2017
2018
2019
2020
2021
2022
2023
Baseline (2010−2019)
Comprehensive measures (2020−2021)
Vaccine−only strategy (2022−2023)
All numbers are rescaled to have an equivalent population to 2023.
Shaded area represents 90% prediction interval.

p. NAV: D04 Rectal/anal pain

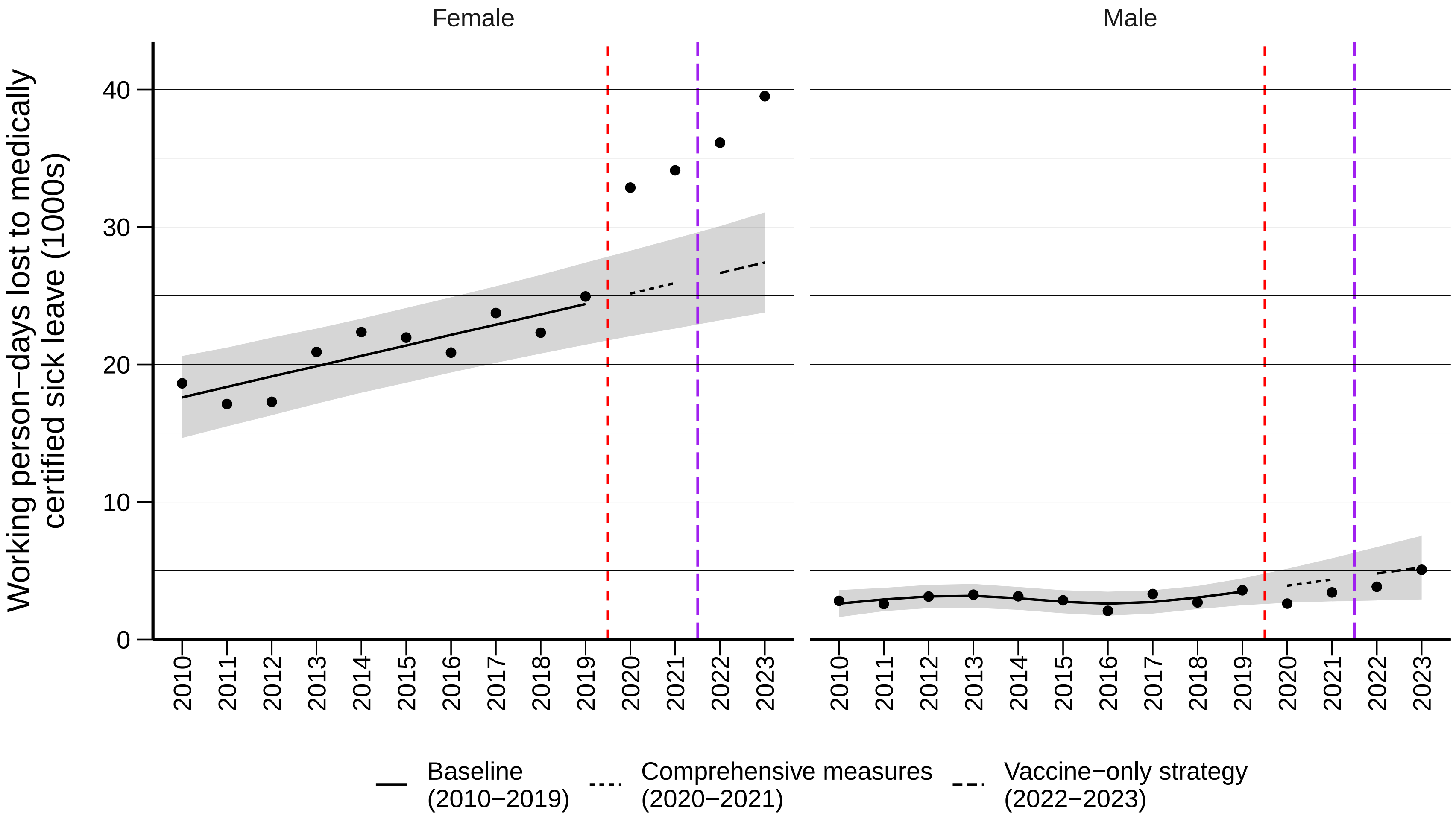

All numbers are rescaled to have an equivalent population to 2023.
Shaded area represents 90% prediction interval.

q. NAV: D87 Stomach function disorder

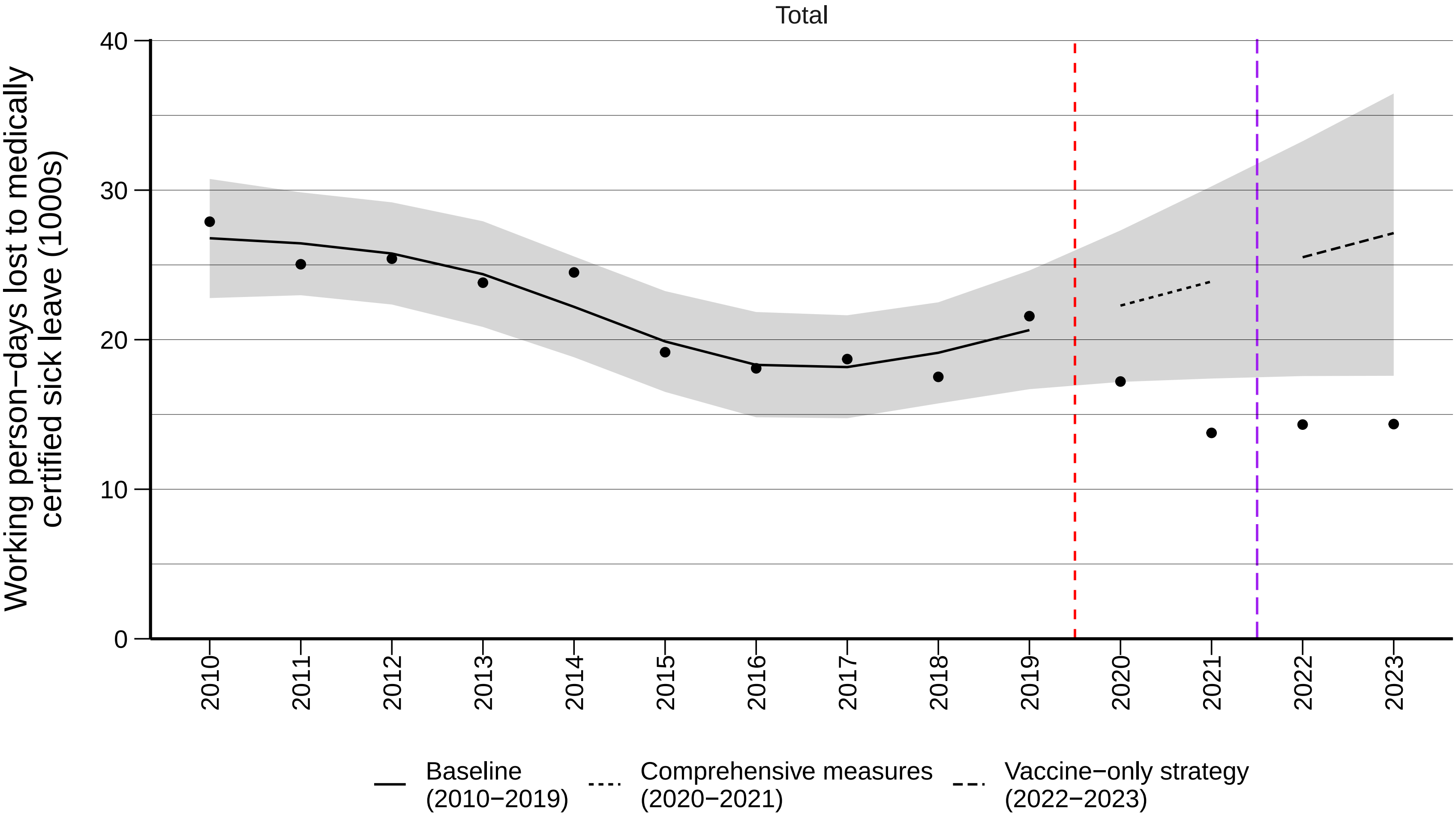

All numbers are rescaled to have an equivalent population to 2023.
Shaded area represents 90% prediction interval.

r. NAV: D87 Stomach function disorder

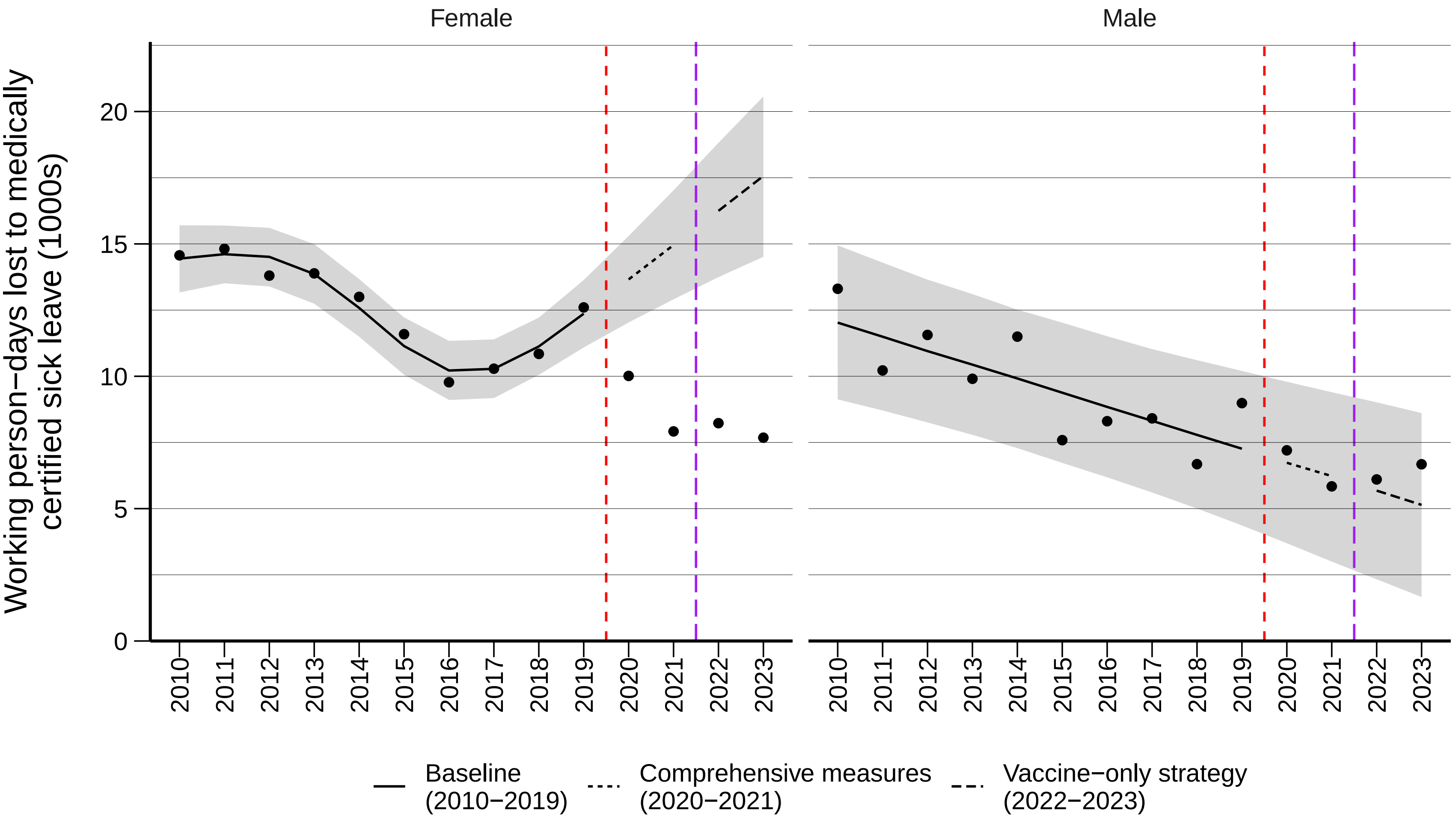

All numbers are rescaled to have an equivalent population to 2023.
Shaded area represents 90% prediction interval.

s. NAV: D92 Diverticular disease

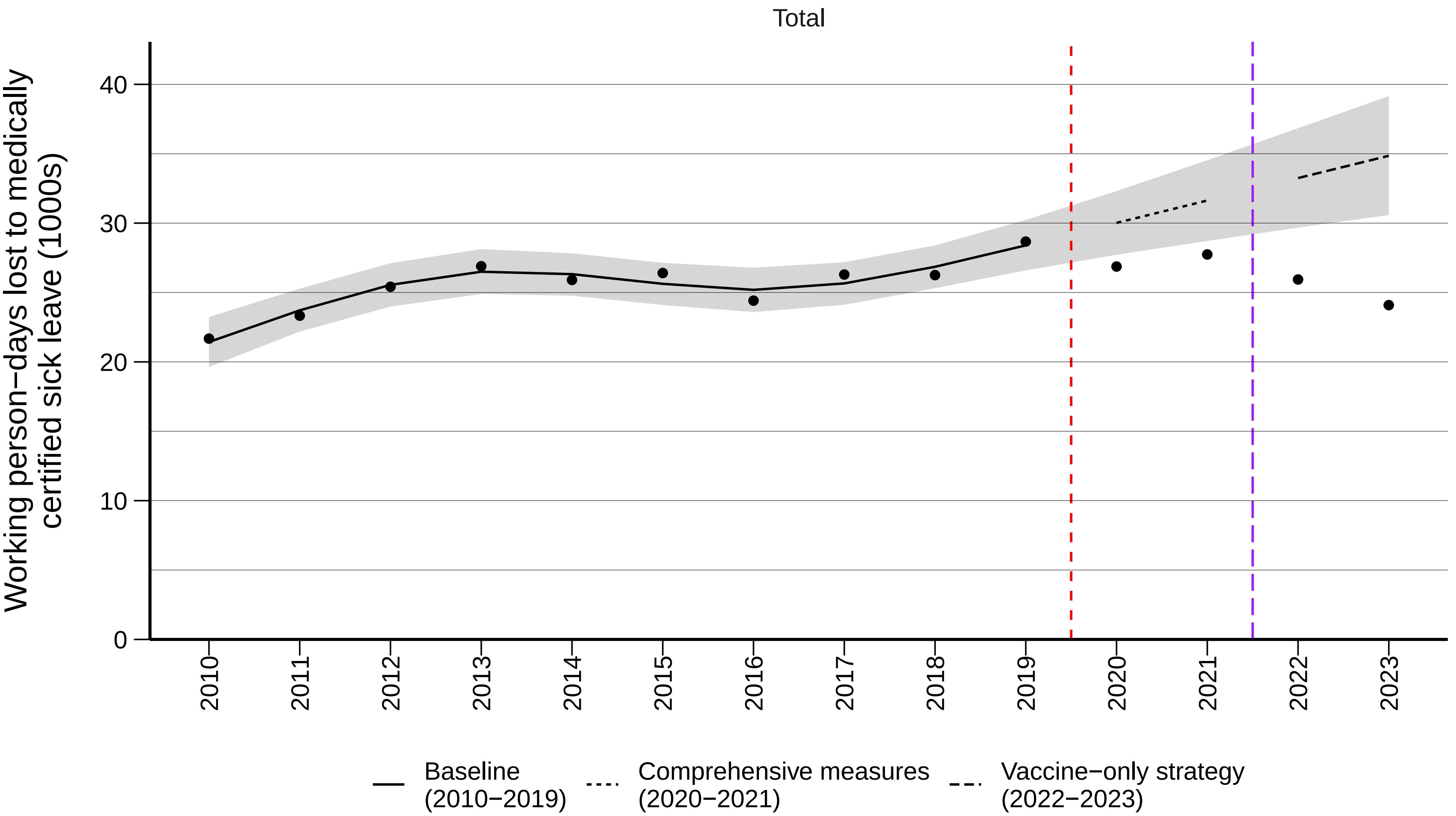

All numbers are rescaled to have an equivalent population to 2023.
Shaded area represents 90% prediction interval.

t. NAV: D92 Diverticular disease

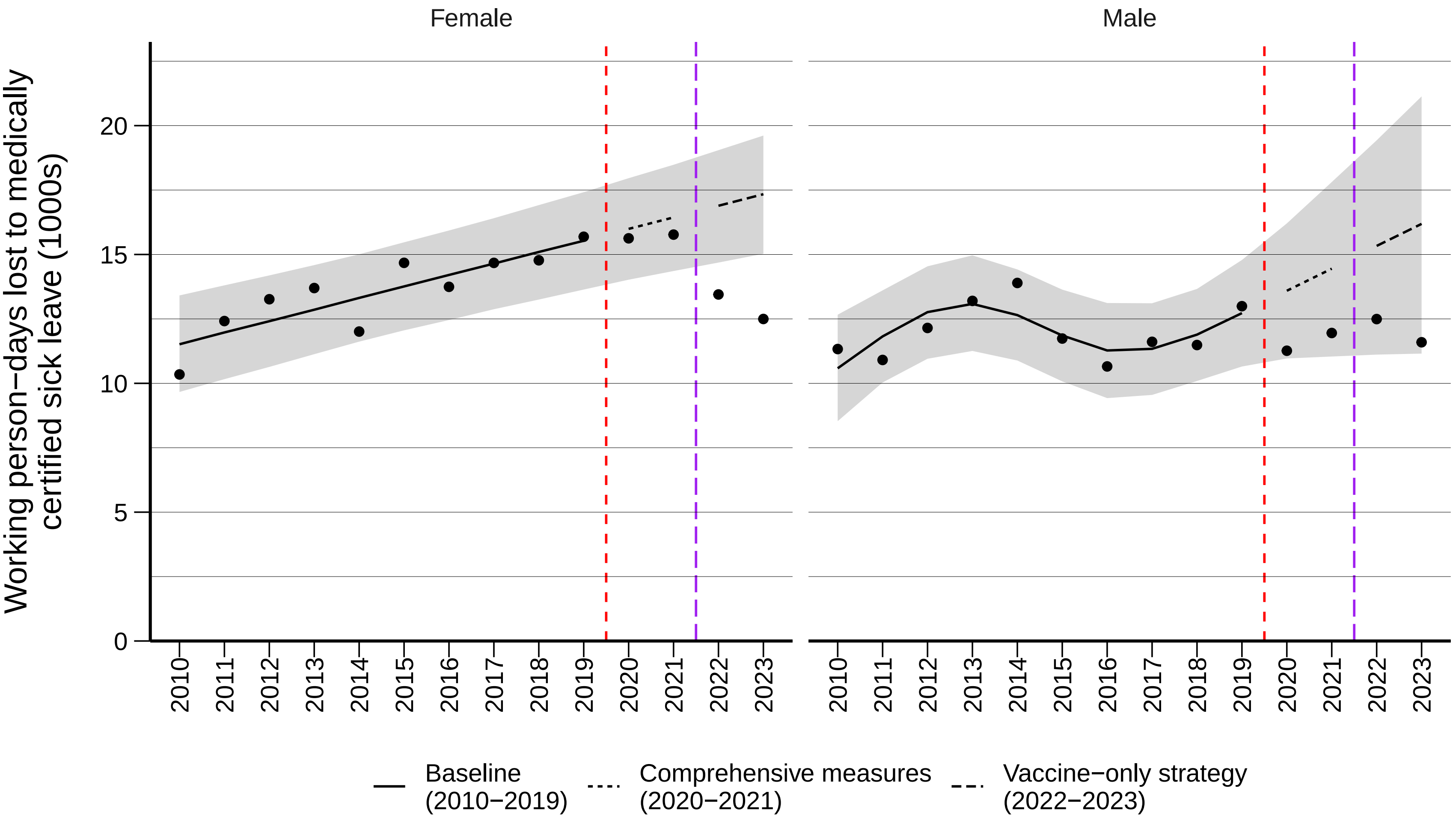

All numbers are rescaled to have an equivalent population to 2023.
Shaded area represents 90% prediction interval.

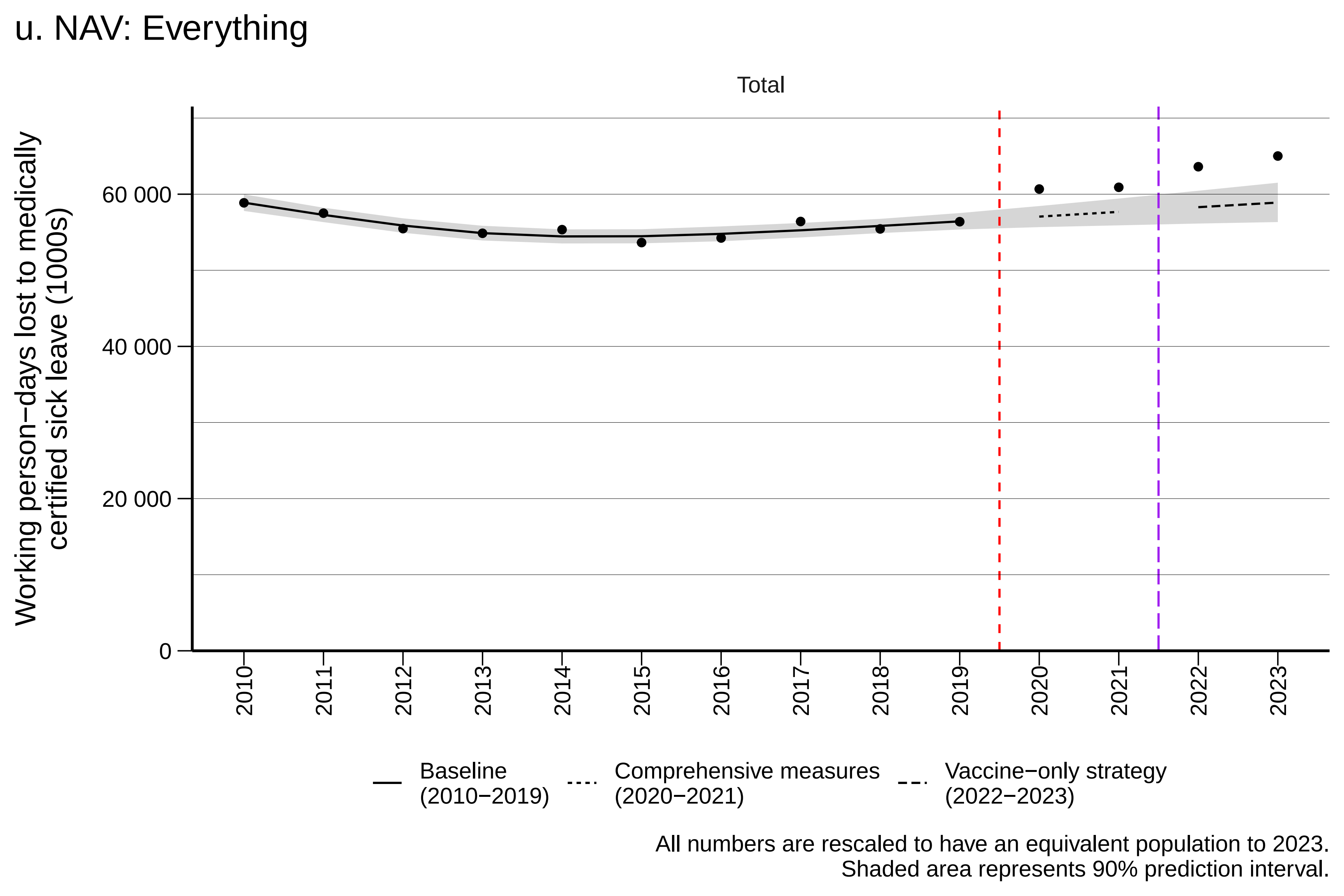

u. NAV: Everything
Total
Working person−days lost to medically certified sick leave (1000s)
60 000
40 000
20 000
0
2010
2011
2012
2013
2014
2015
2016
2017
2018
2019
2020
2021
2022
2023
Baseline (2010−2019)
Comprehensive measures (2020−2021)
Vaccine−only strategy (2022−2023)
All numbers are rescaled to have an equivalent population to 2023.
Shaded area represents 90% prediction interval.

v. NAV: Everything

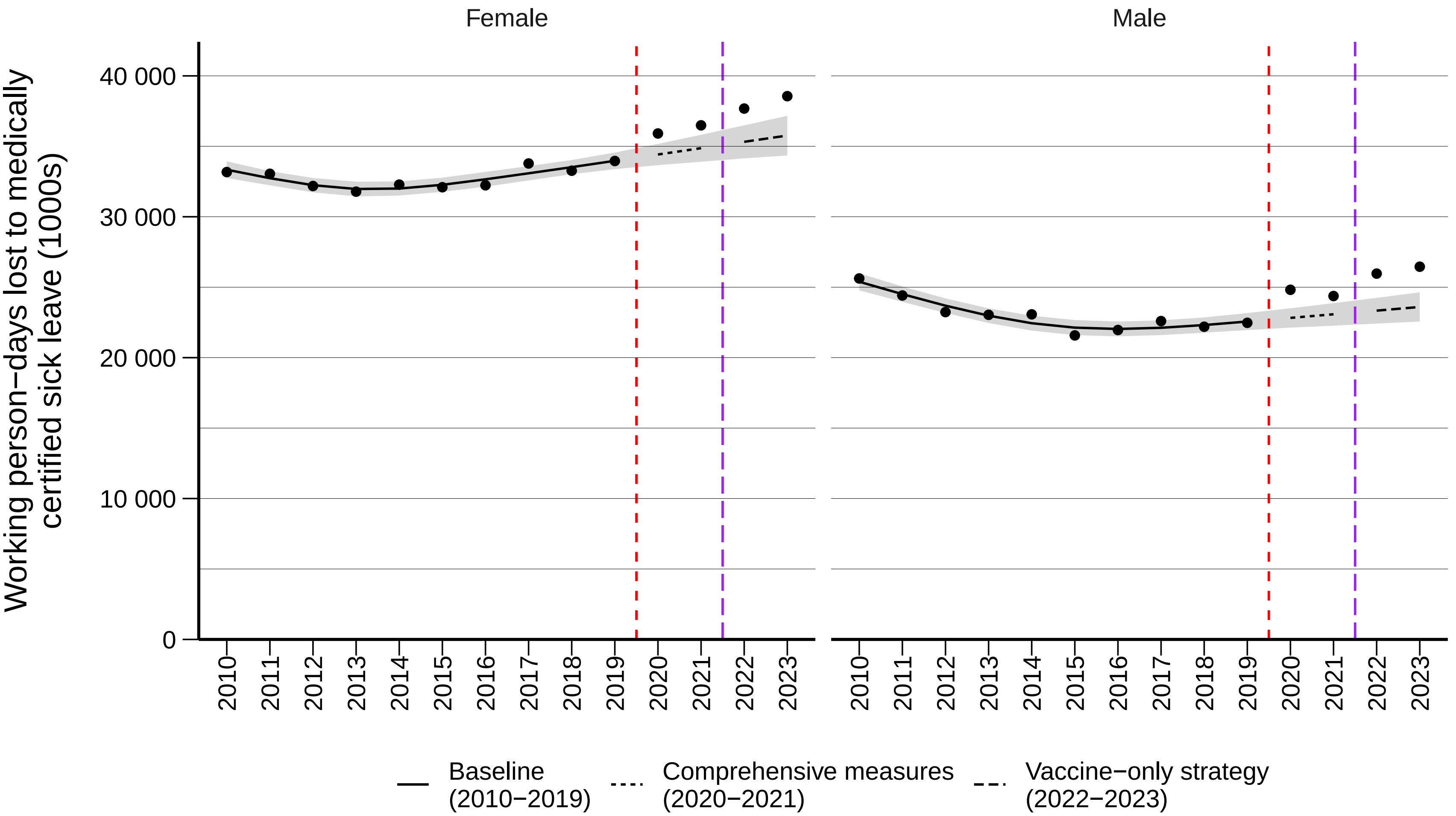

All numbers are rescaled to have an equivalent population to 2023.
Shaded area represents 90% prediction interval.

w. NAV: K76 Ischaemic heart disease w/o angina

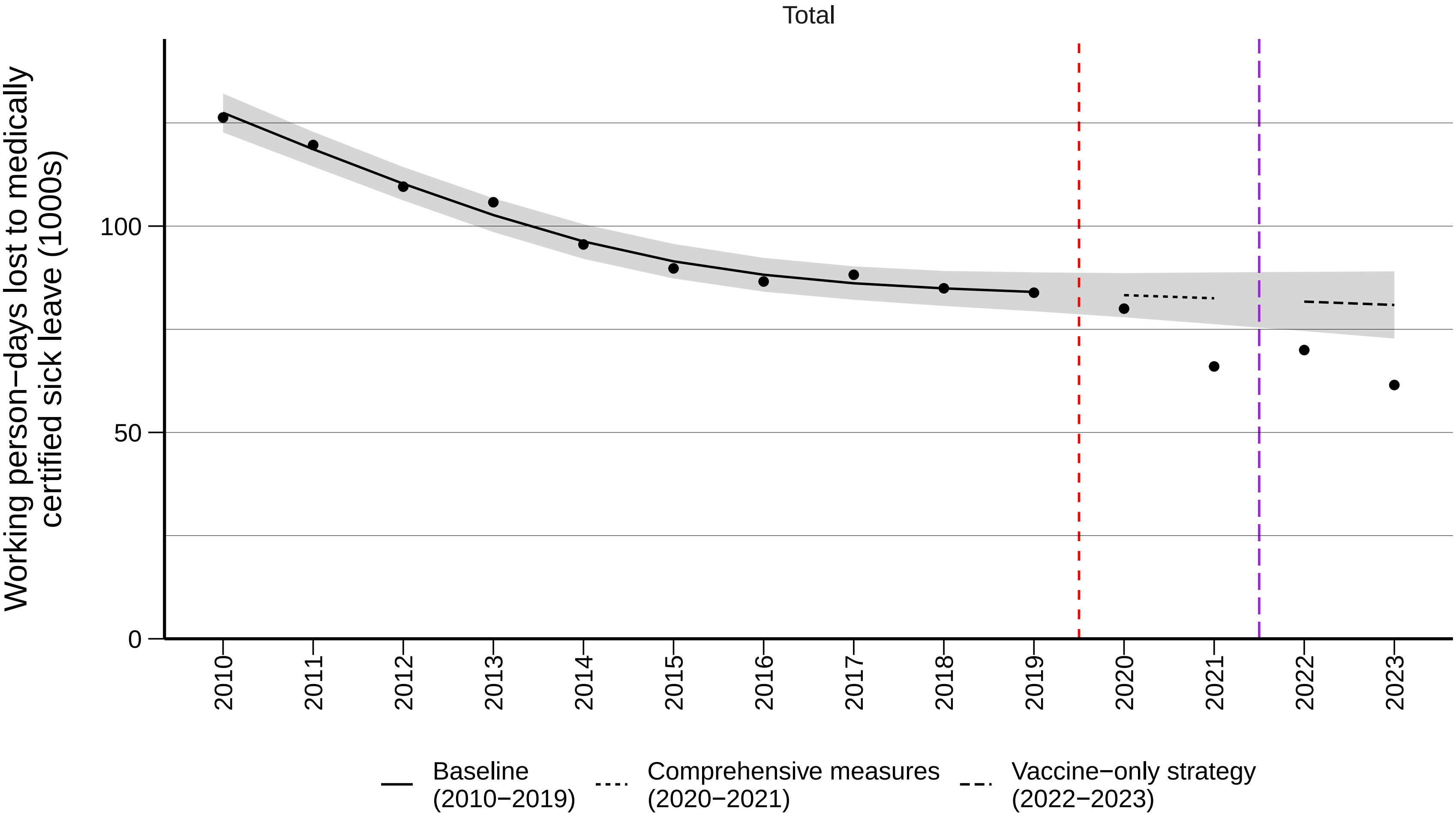

All numbers are rescaled to have an equivalent population to 2023.
Shaded area represents 90% prediction interval.

x. NAV: K76 Ischaemic heart disease w/o angina

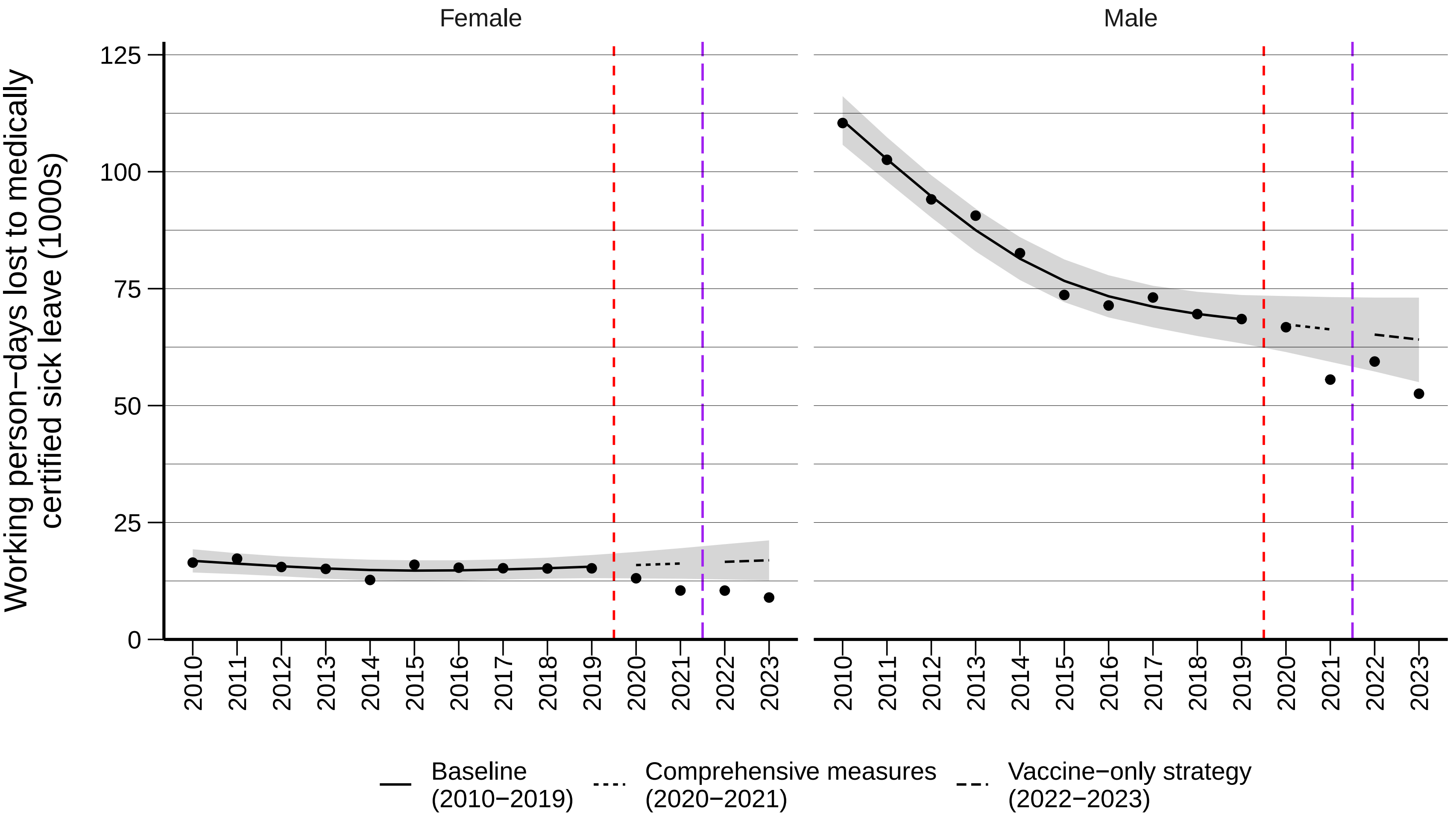

All numbers are rescaled to have an equivalent population to 2023.
Shaded area represents 90% prediction interval.

y. NAV: L01 Neck symptom/complain

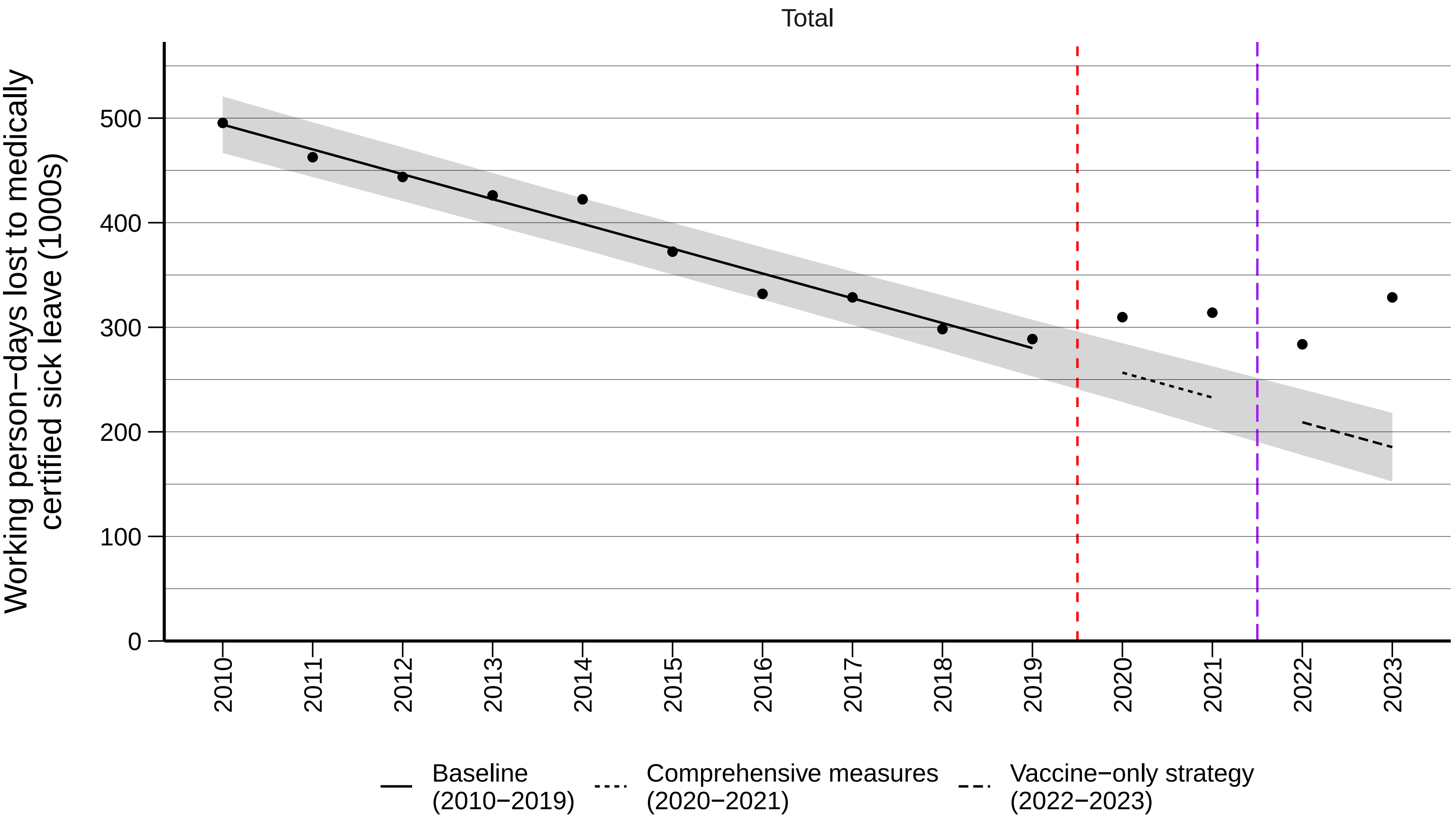

All numbers are rescaled to have an equivalent population to 2023.
Shaded area represents 90% prediction interval.

z. NAV: L01 Neck symptom/complain

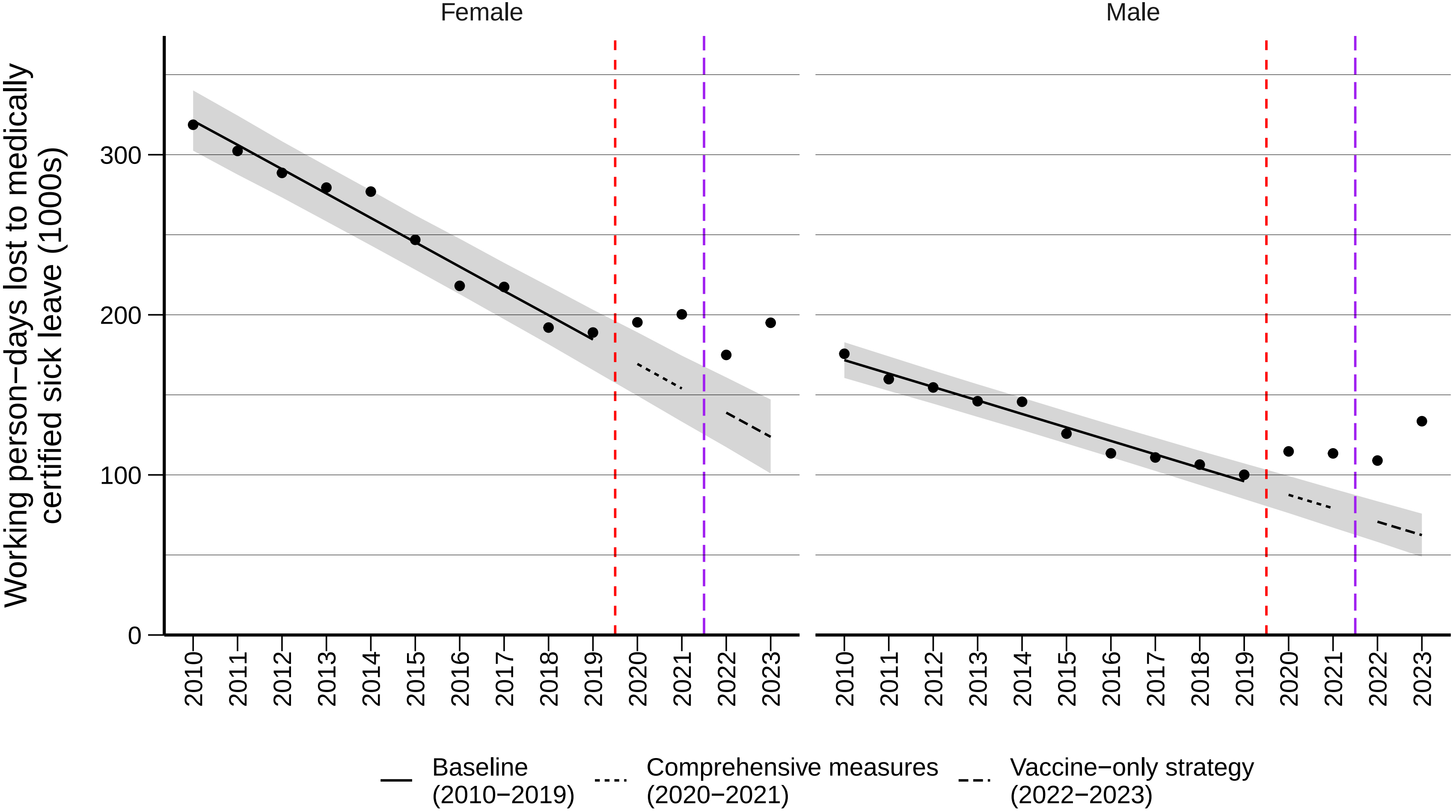

All numbers are rescaled to have an equivalent population to 2023.
Shaded area represents 90% prediction interval.

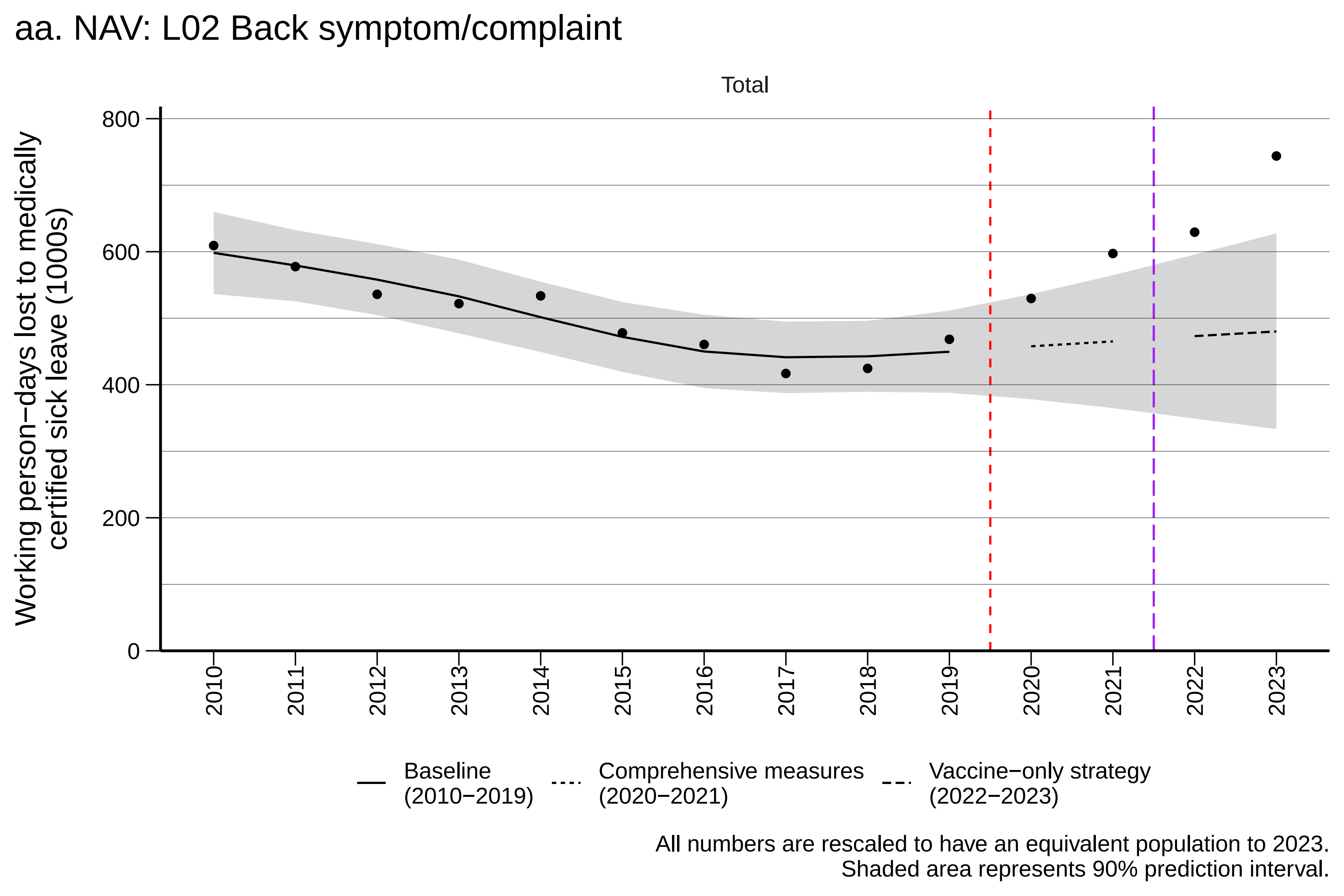

aa. NAV: L02 Back symptom/complaint
Total
Working person−days lost to medically certified sick leave (1000s)
0
200
400
600
800
2010
2011
2012
2013
2014
2015
2016
2017
2018
2019
2020
2021
2022
2023
Baseline (2010−2019)
Comprehensive measures (2020−2021)
Vaccine−only strategy (2022−2023)
All numbers are rescaled to have an equivalent population to 2023.
Shaded area represents 90% prediction interval.

ab. NAV: L02 Back symptom/complaint

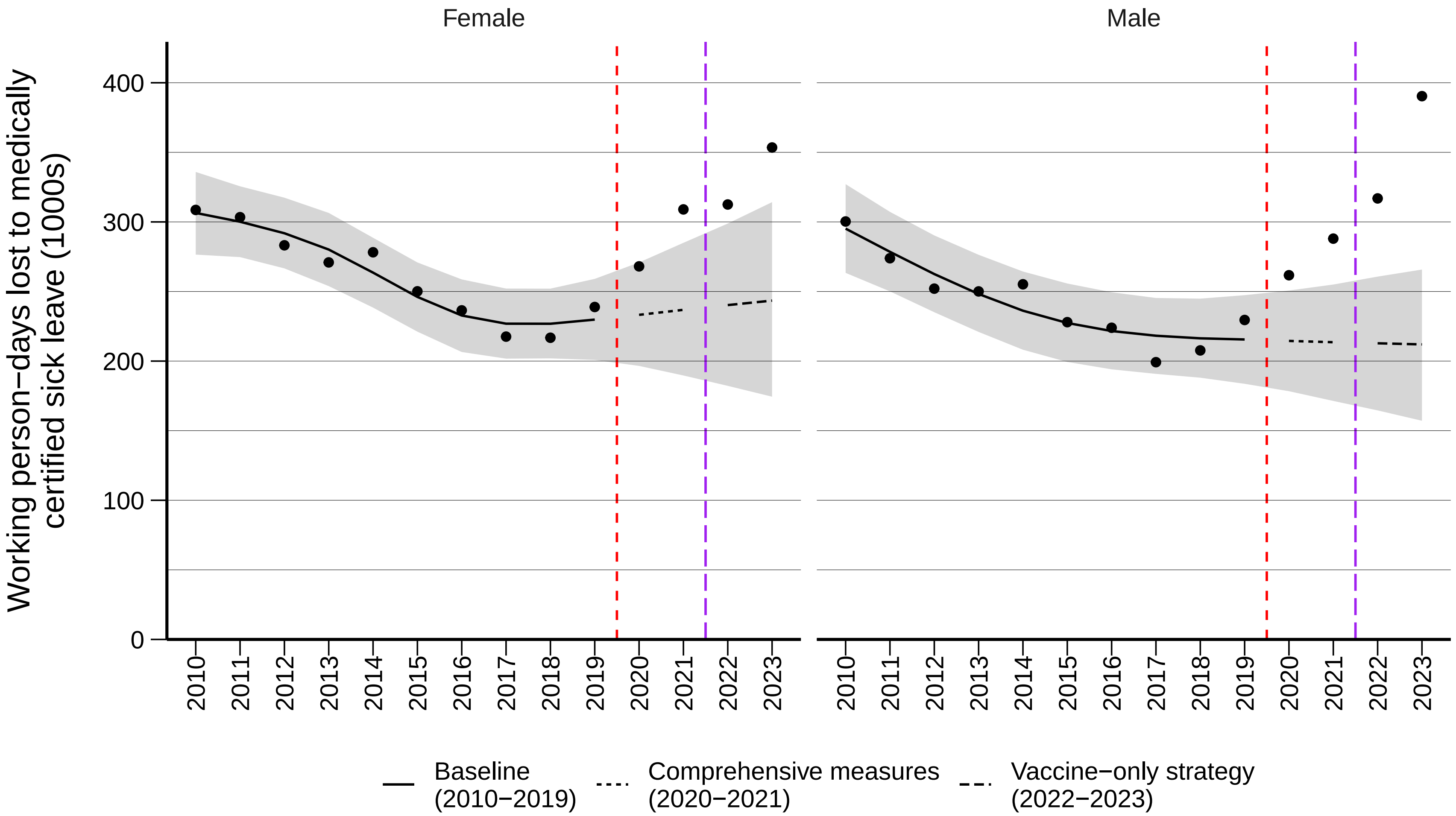

All numbers are rescaled to have an equivalent population to 2023.
Shaded area represents 90% prediction interval.

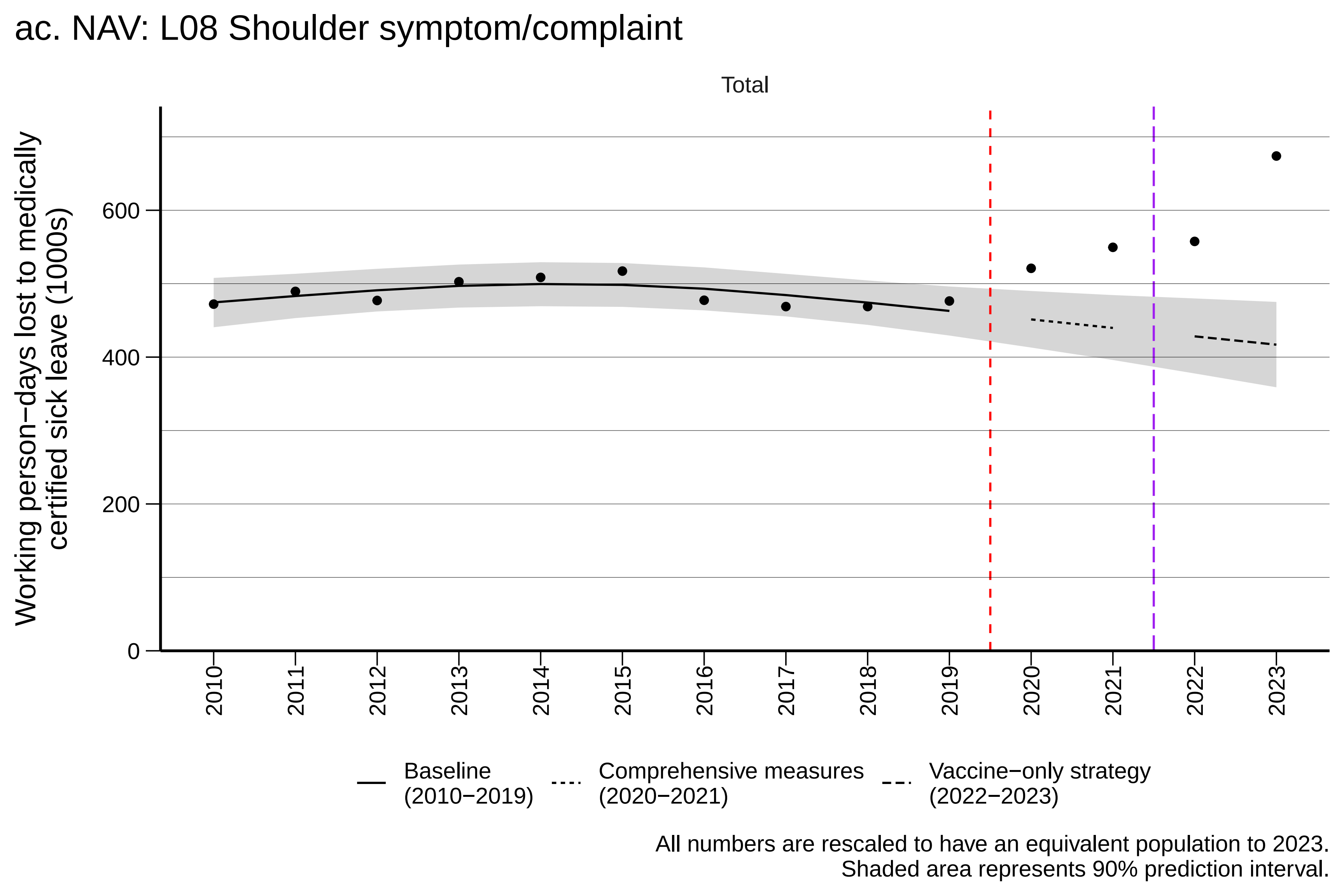

ac. NAV: L08 Shoulder symptom/complaint
Total
Working person−days lost to medically certified sick leave (1000s)
0
200
400
600
2010
2011
2012
2013
2014
2015
2016
2017
2018
2019
2020
2021
2022
2023
Baseline (2010−2019)
Comprehensive measures (2020−2021)
Vaccine−only strategy (2022−2023)
All numbers are rescaled to have an equivalent population to 2023.
Shaded area represents 90% prediction interval.

ad. NAV: L08 Shoulder symptom/complaint

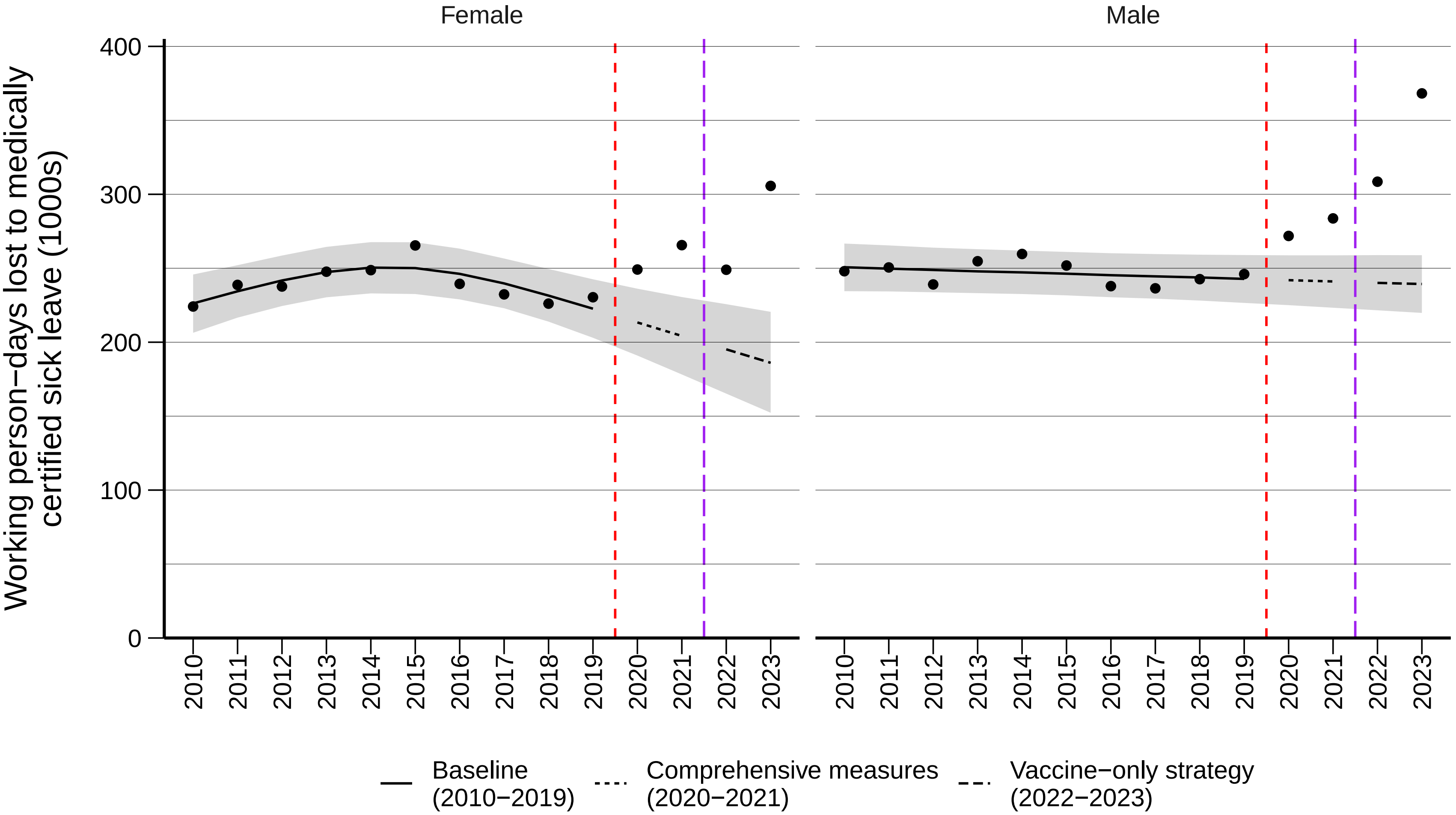

All numbers are rescaled to have an equivalent population to 2023.
Shaded area represents 90% prediction interval.

ae. NAV: L11 Wrist symptom/complaint

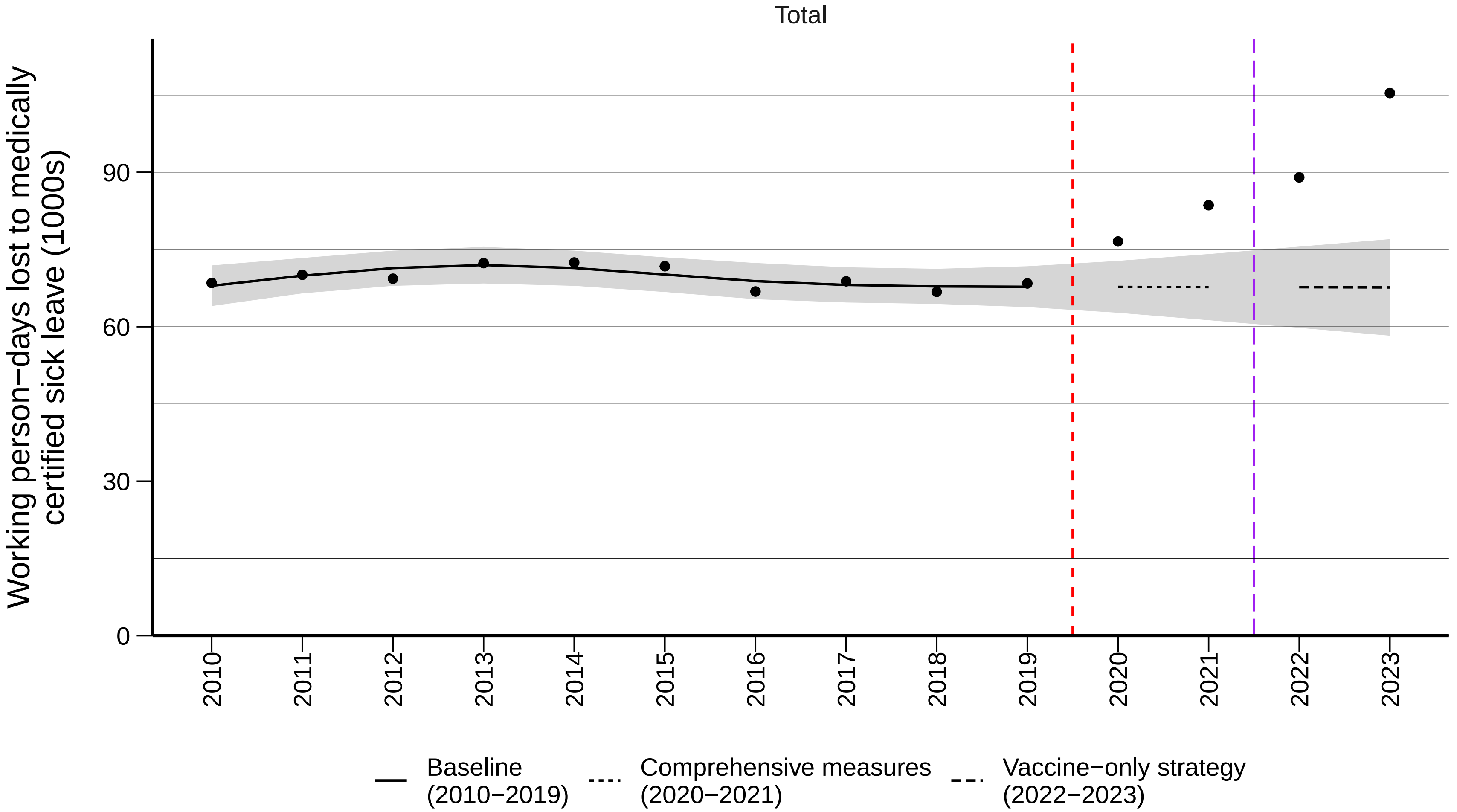

All numbers are rescaled to have an equivalent population to 2023.
Shaded area represents 90% prediction interval.

af. NAV: L11 Wrist symptom/complaint

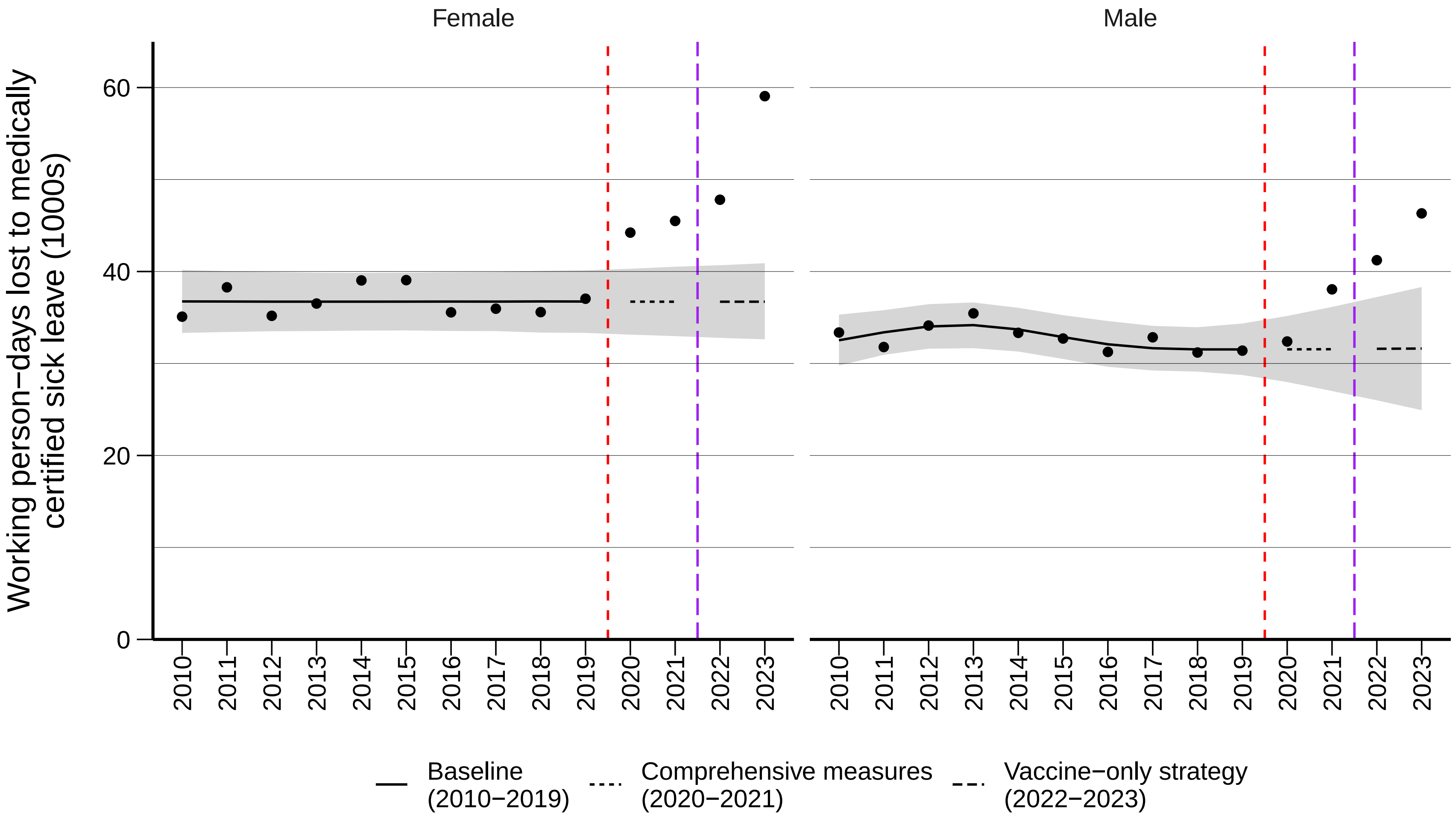

All numbers are rescaled to have an equivalent population to 2023.
Shaded area represents 90% prediction interval.

ag. NAV: L12 Hand/finger symptom/complaint

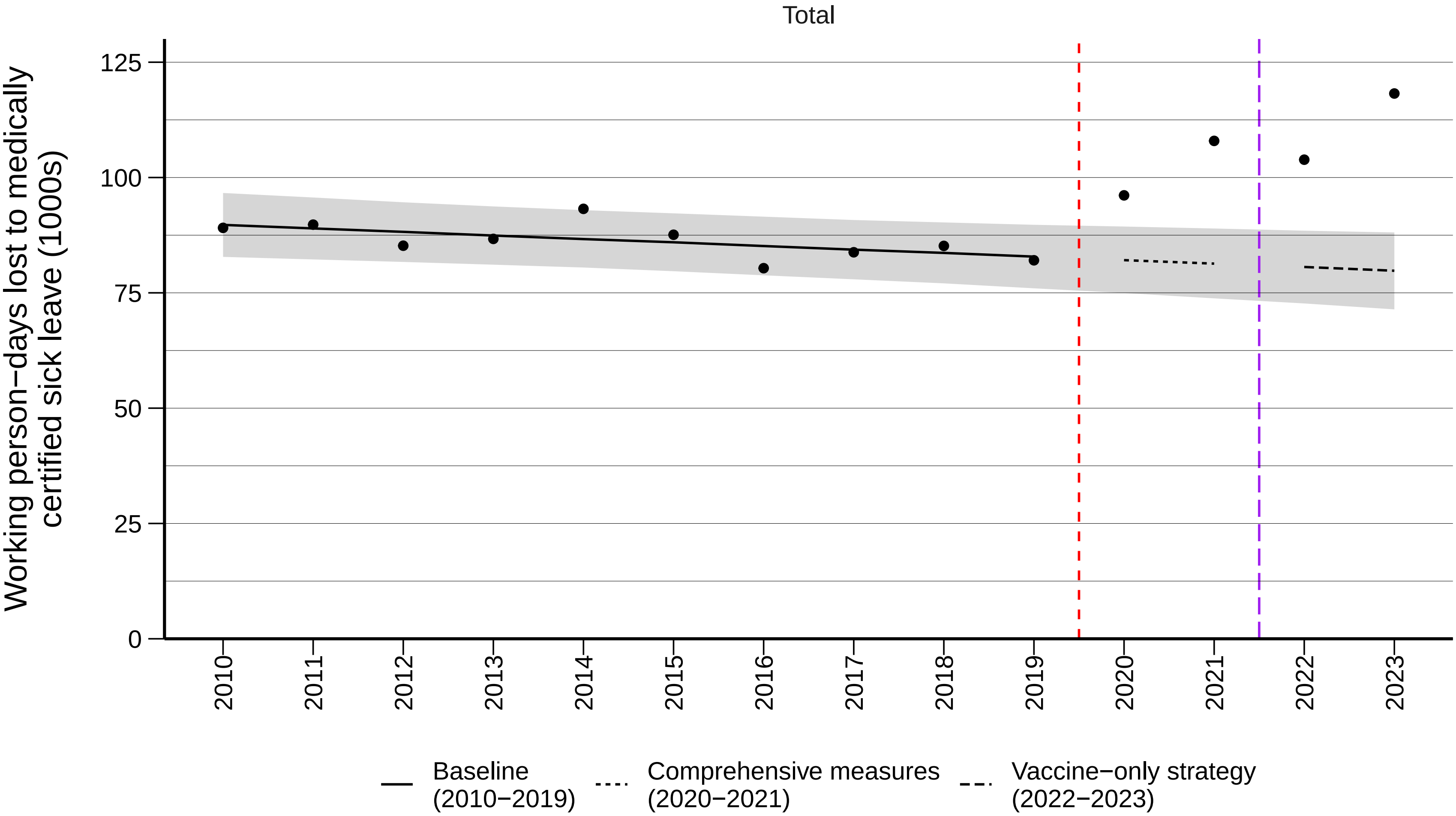

All numbers are rescaled to have an equivalent population to 2023.
Shaded area represents 90% prediction interval.

ah. NAV: L12 Hand/finger symptom/complaint

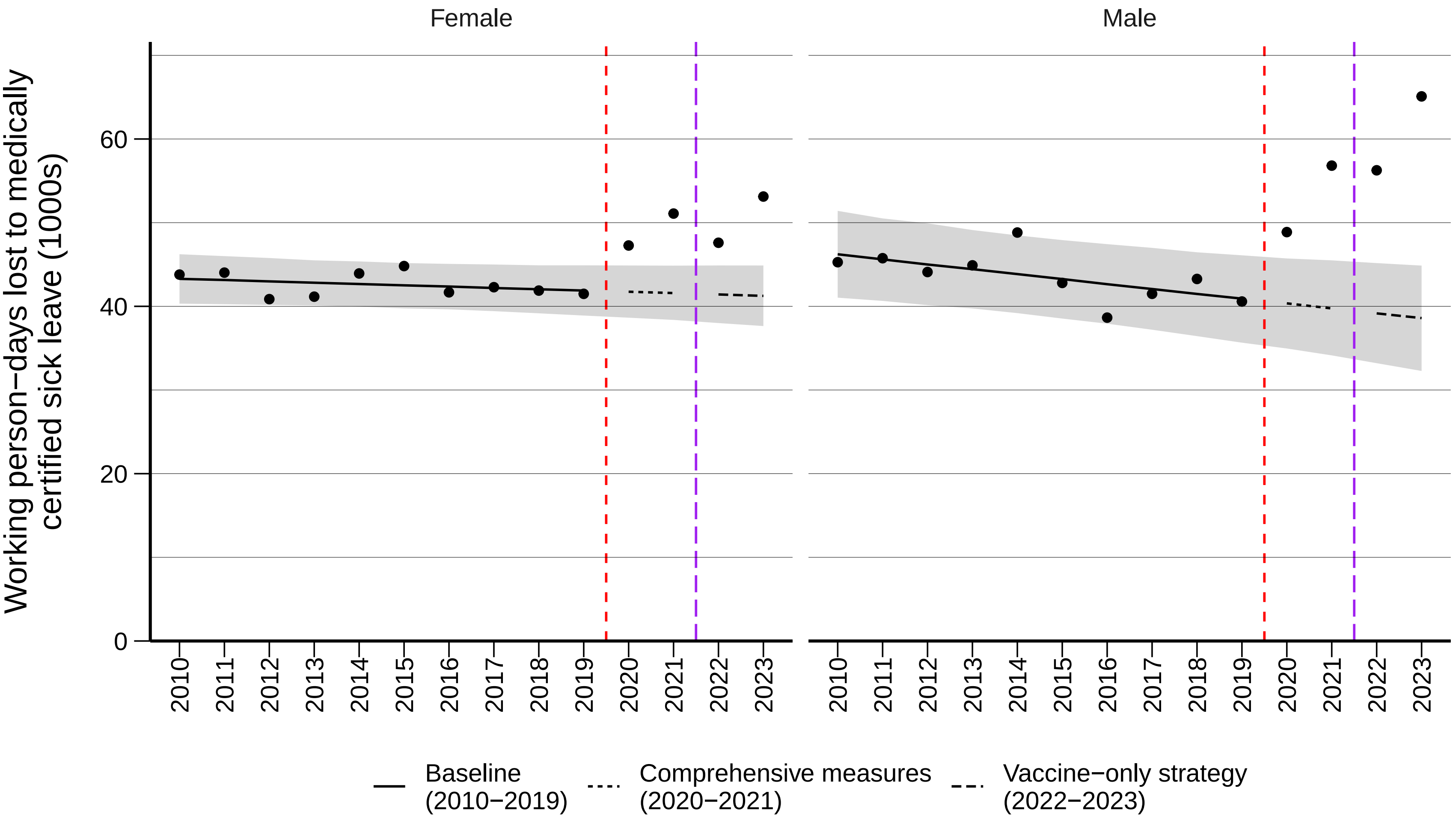

All numbers are rescaled to have an equivalent population to 2023.
Shaded area represents 90% prediction interval.

ai. NAV: L13 Hip symptom/complaint

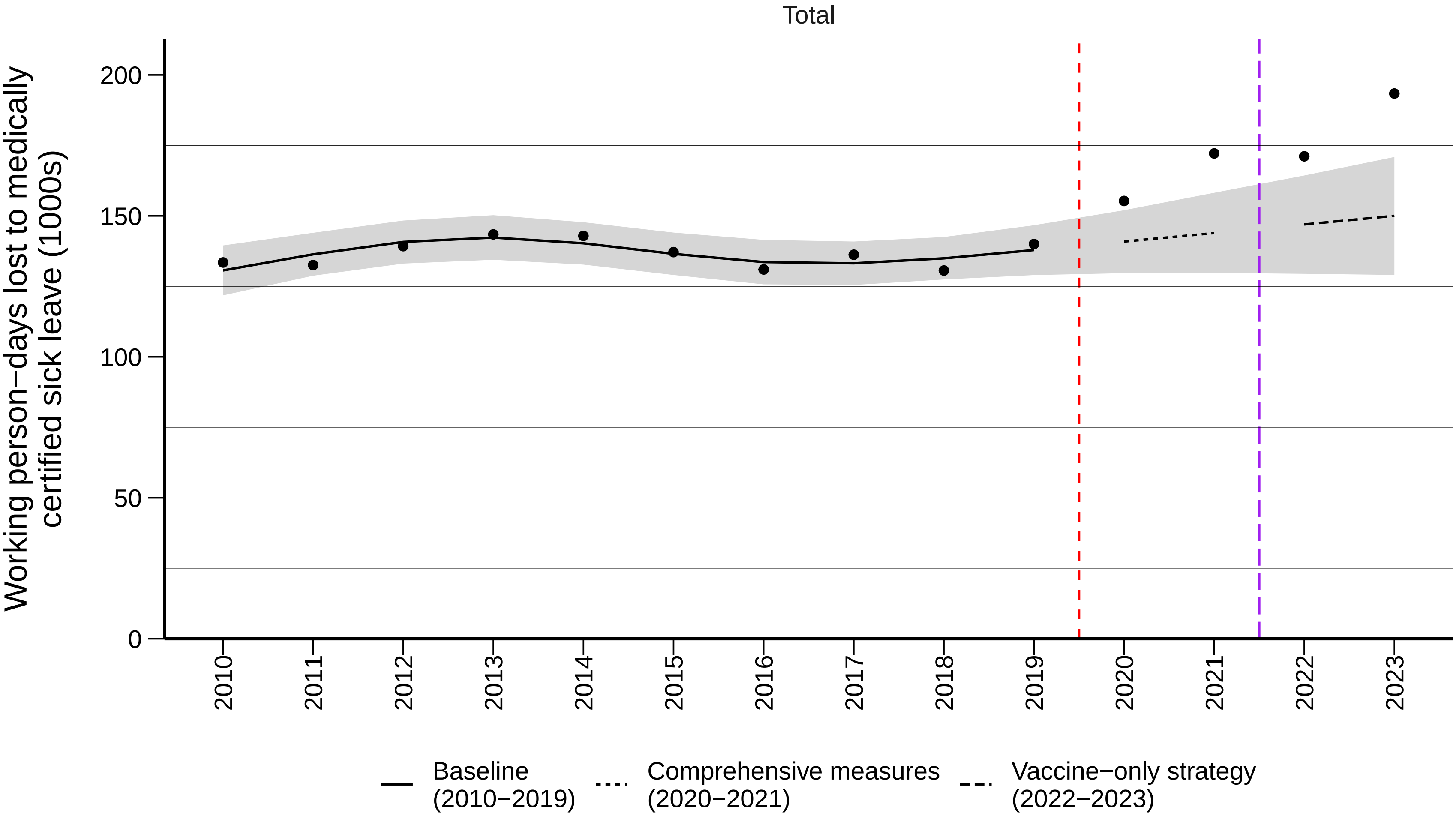

All numbers are rescaled to have an equivalent population to 2023.
Shaded area represents 90% prediction interval.

aj. NAV: L13 Hip symptom/complaint

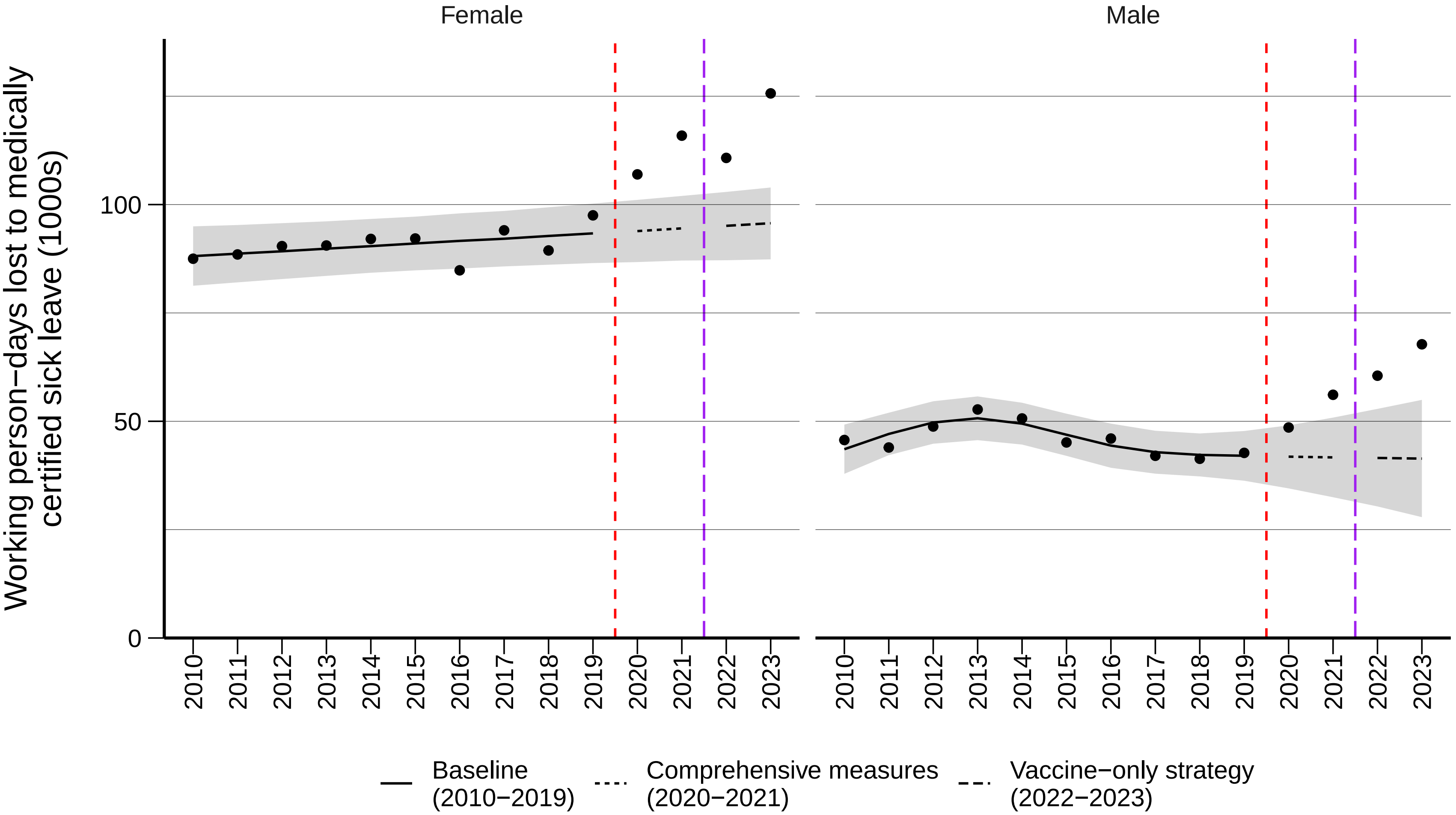

All numbers are rescaled to have an equivalent population to 2023.
Shaded area represents 90% prediction interval.

ak. NAV: L16 Ankle symptom/complaint

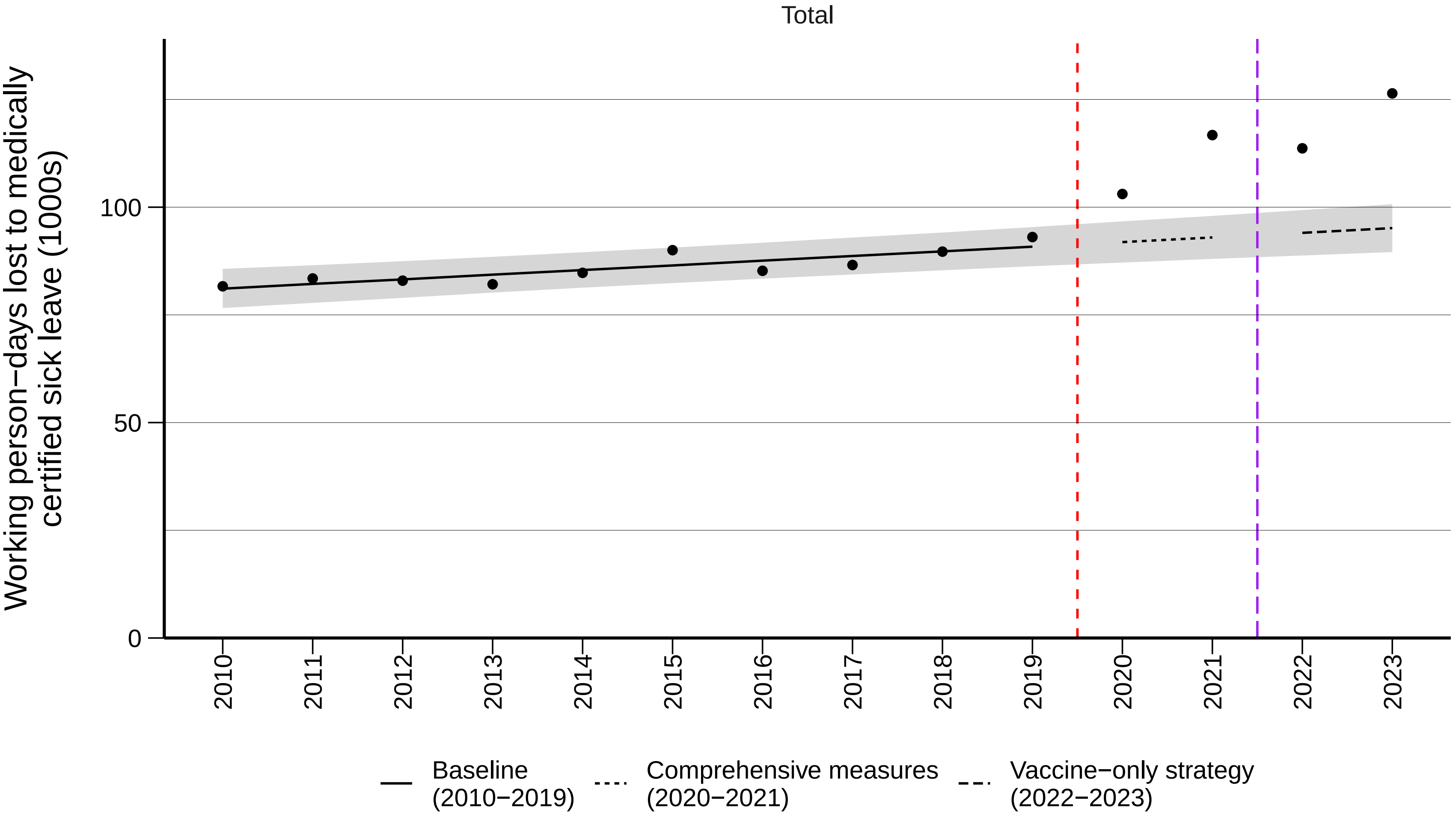

All numbers are rescaled to have an equivalent population to 2023.
Shaded area represents 90% prediction interval.

al. NAV: L16 Ankle symptom/complaint

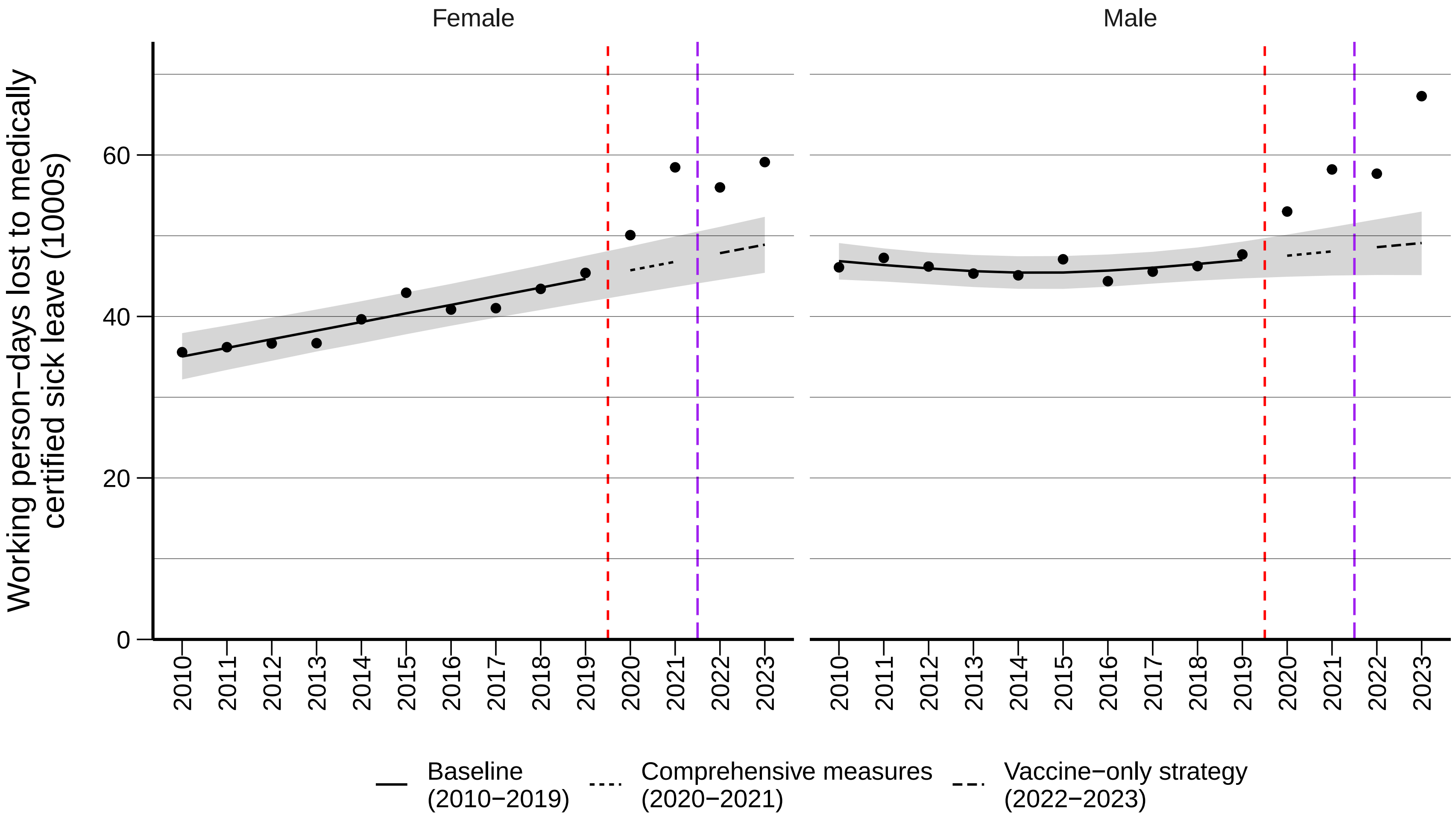

All numbers are rescaled to have an equivalent population to 2023.
Shaded area represents 90% prediction interval.

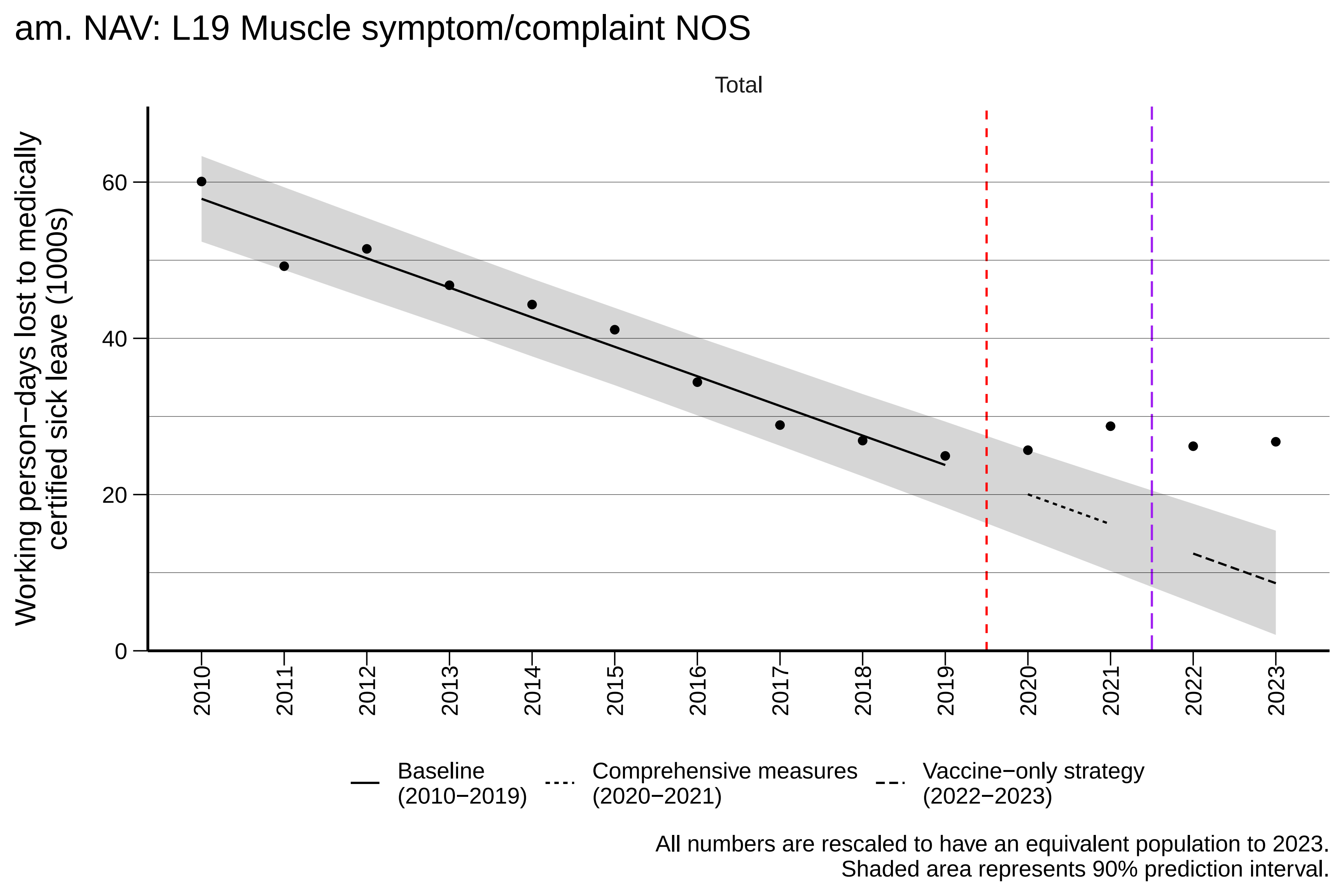
am. NAV: L19 Muscle symptom/complaint NOS
Total
Working person−days lost to medically certified sick leave (1000s)
60
40
20
0
2010
2011
2012
2013
2014
2015
2016
2017
2018
2019
2020
2021
2022
2023
Baseline (2010−2019)
Comprehensive measures (2020−2021)
Vaccine−only strategy (2022−2023)
All numbers are rescaled to have an equivalent population to 2023.
Shaded area represents 90% prediction interval.

an. NAV: L19 Muscle symptom/complaint NOS

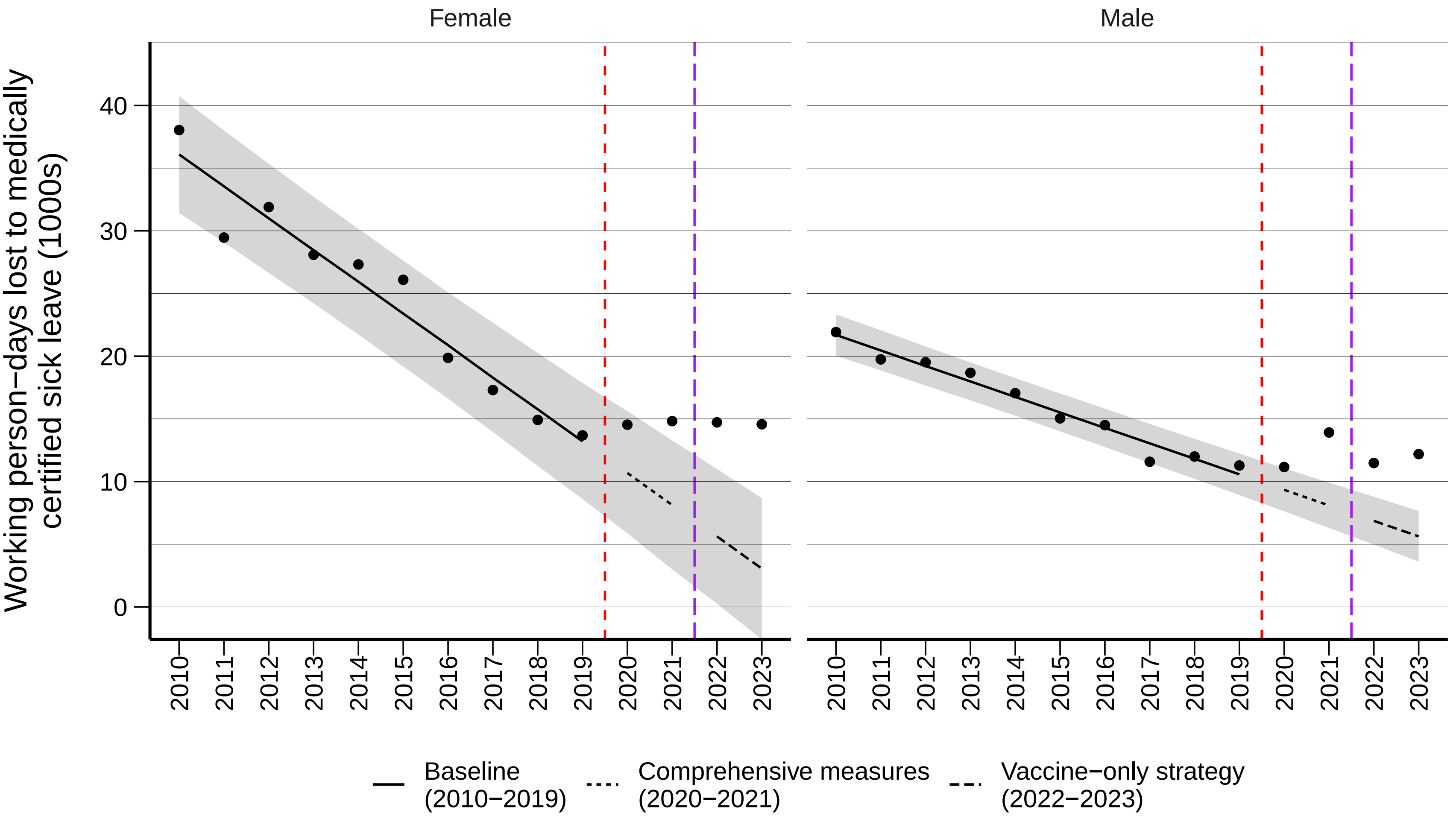

All numbers are rescaled to have an equivalent population to 2023.
Shaded area represents 90% prediction interval.

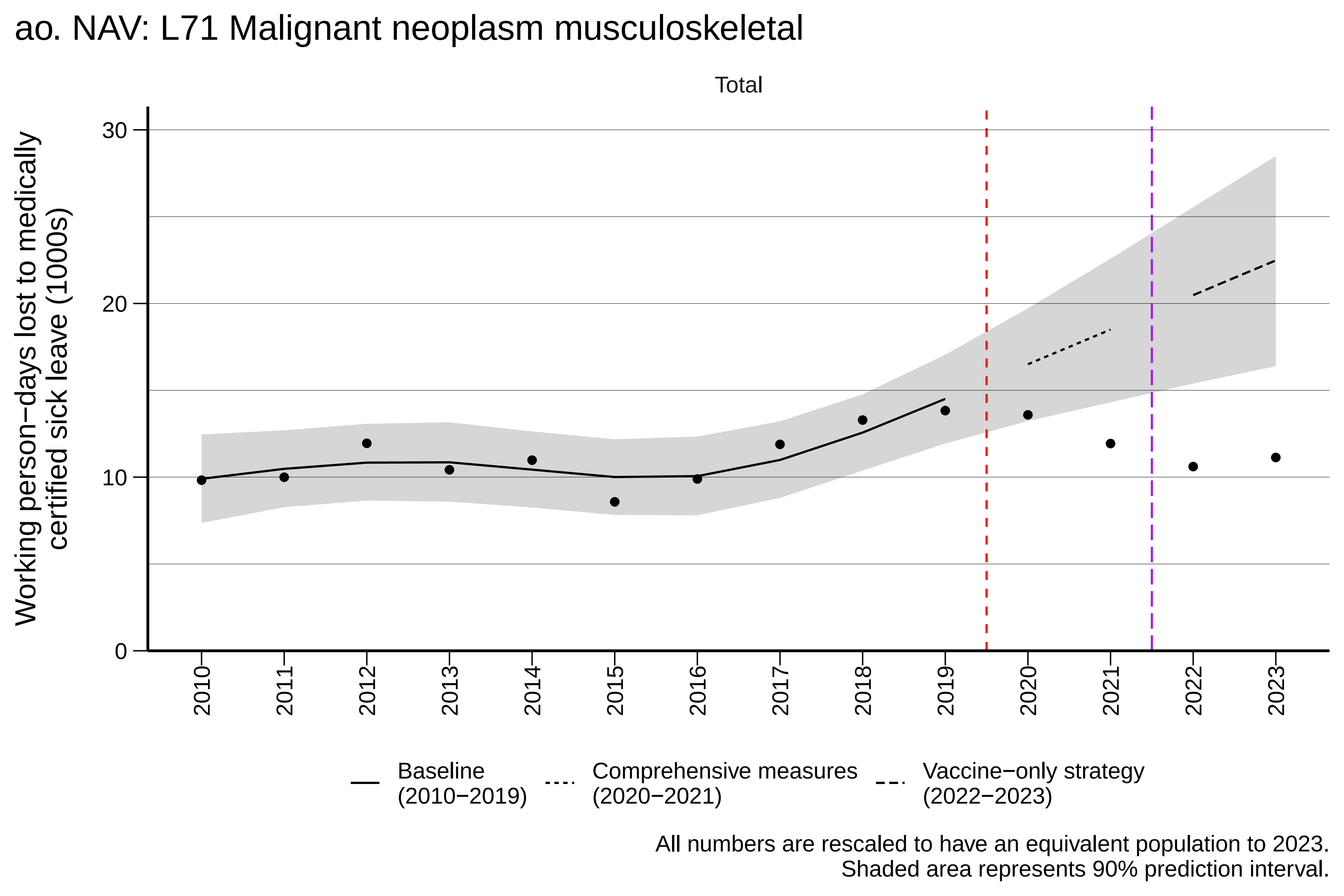

ao. NAV: L71 Malignant neoplasm musculoskeletal
Total
Working person−days lost to medically certified sick leave (1000s)
0
10
20
30
2010
2011
2012
2013
2014
2015
2016
2017
2018
2019
2020
2021
2022
2023
Baseline (2010−2019)
Comprehensive measures (2020−2021)
Vaccine−only strategy (2022−2023)
All numbers are rescaled to have an equivalent population to 2023.
Shaded area represents 90% prediction interval.

ap. NAV: L71 Malignant neoplasm musculoskeletal

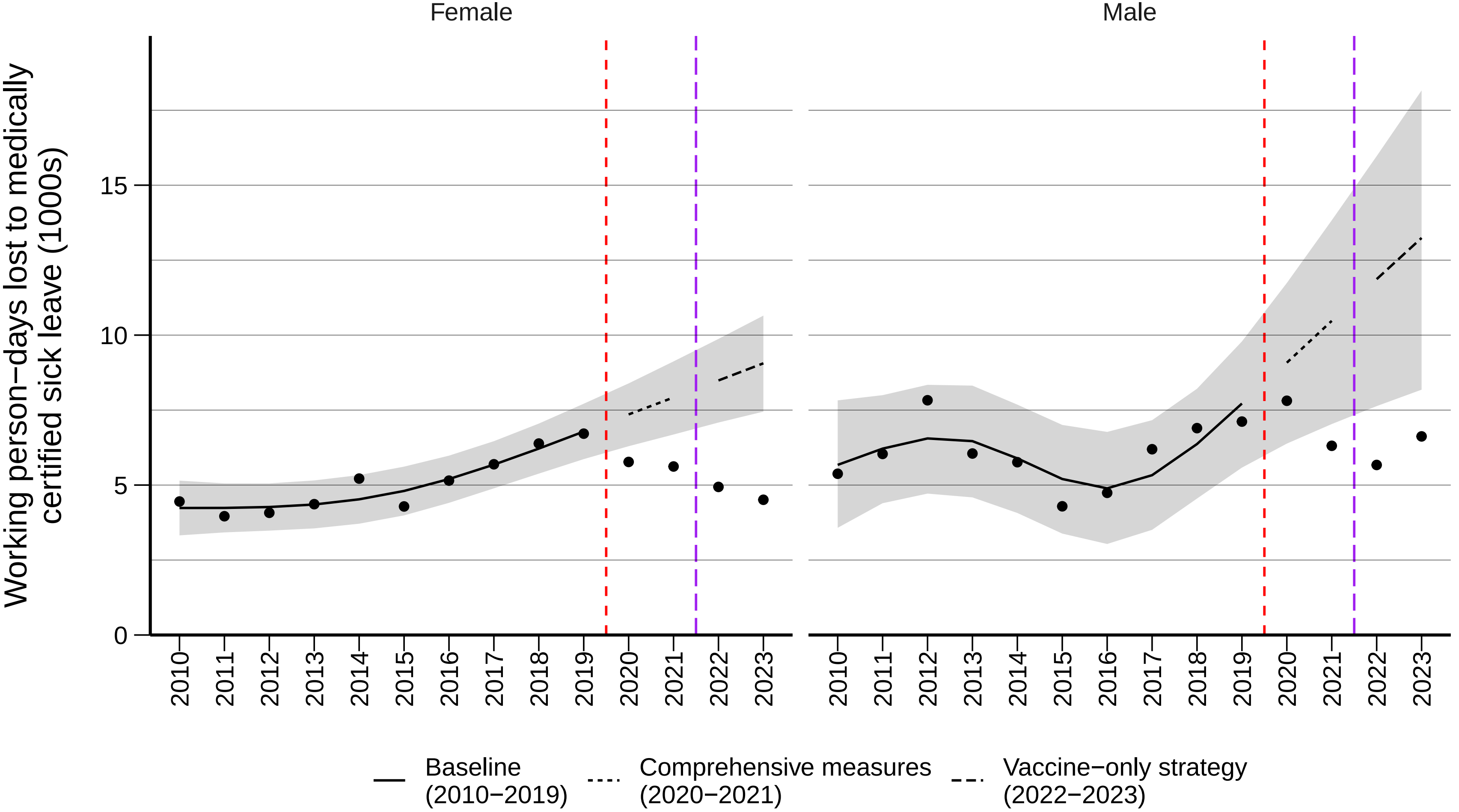

All numbers are rescaled to have an equivalent population to 2023.
Shaded area represents 90% prediction interval.

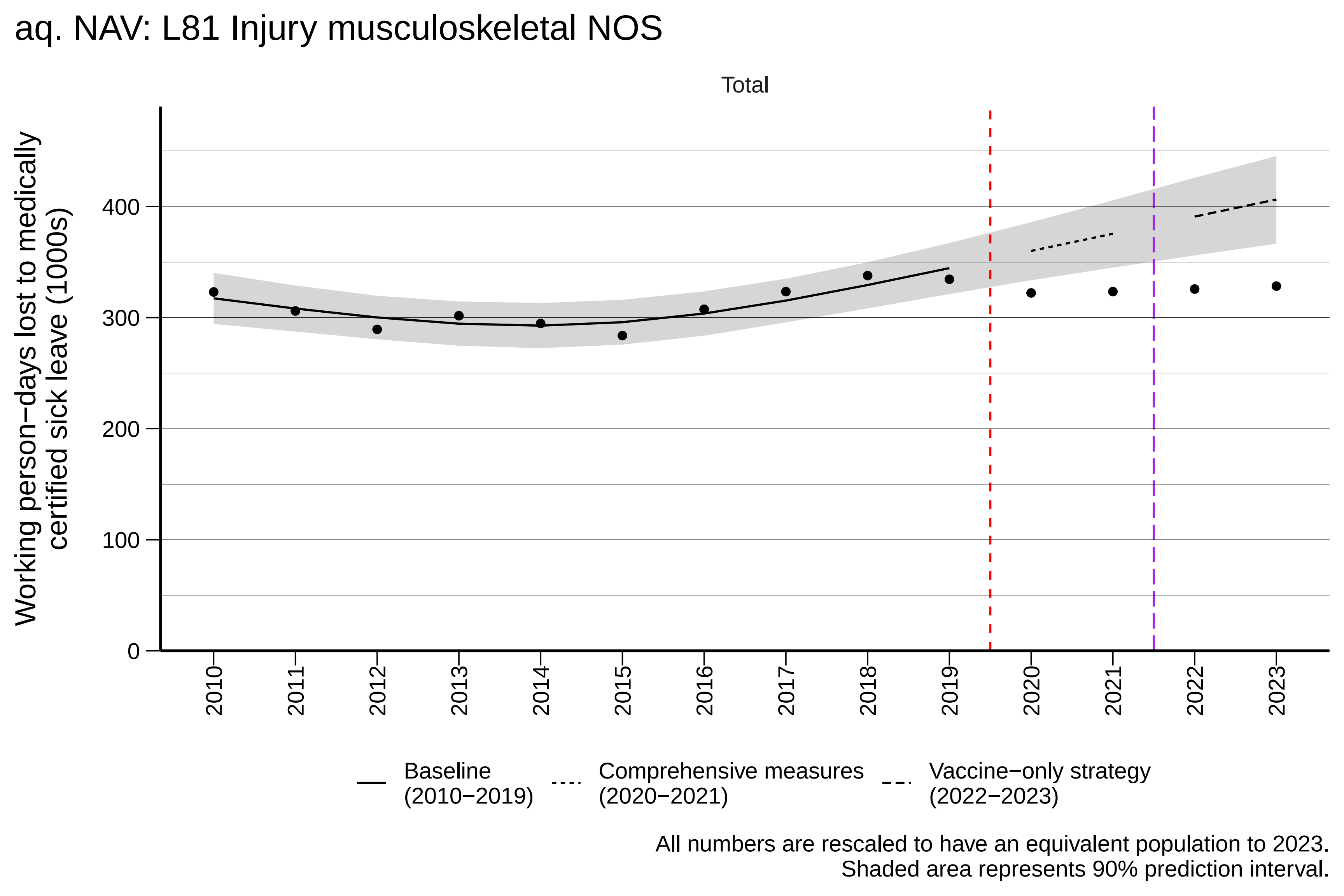

aq. NAV: L81 Injury musculoskeletal NOS
Total
Working person−days lost to medically certified sick leave (1000s)
0
100
200
300
400
2010
2011
2012
2013
2014
2015
2016
2017
2018
2019
2020
2021
2022
2023
Baseline (2010−2019)
Comprehensive measures (2020−2021)
Vaccine−only strategy (2022−2023)
All numbers are rescaled to have an equivalent population to 2023.
Shaded area represents 90% prediction interval.

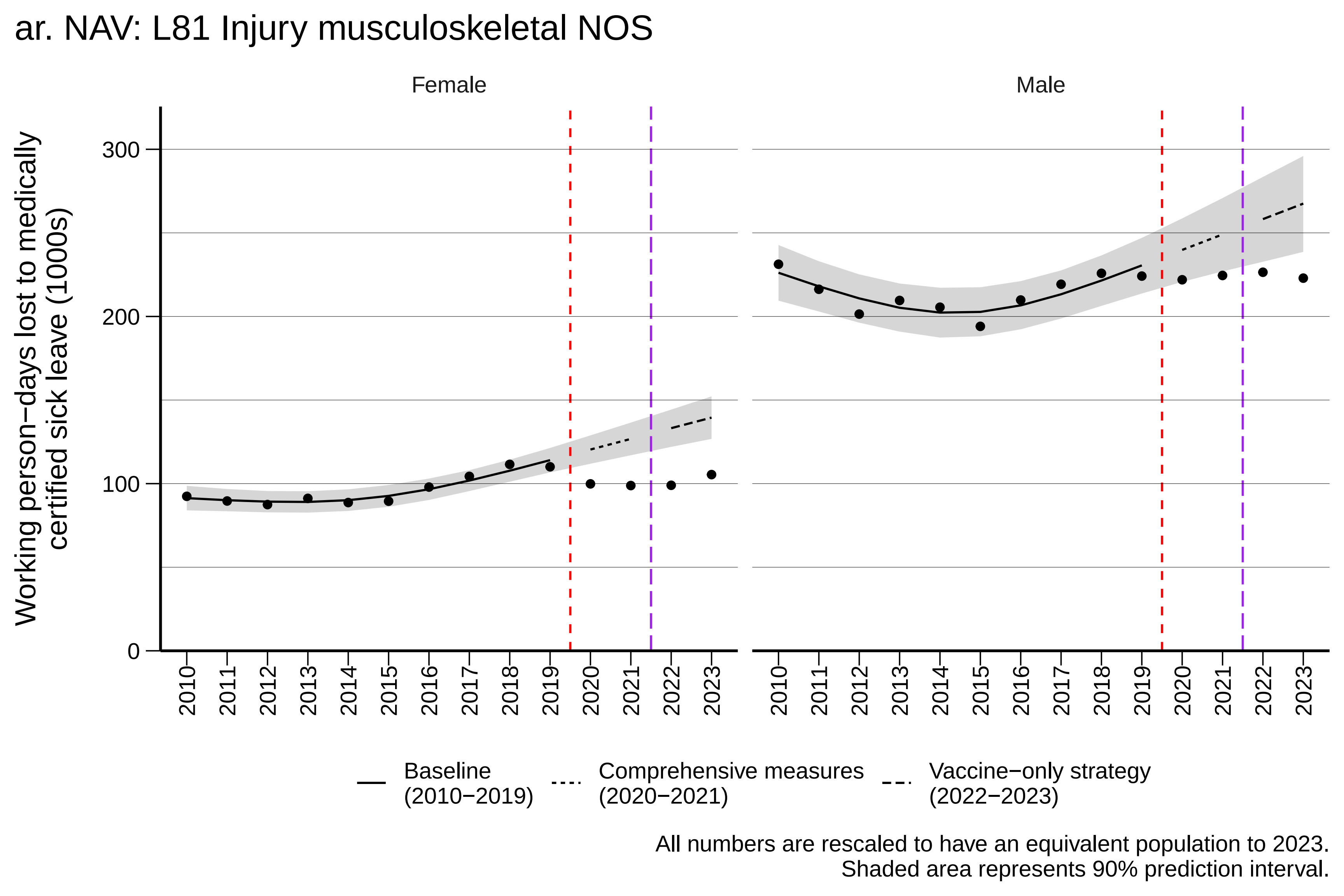
ar. NAV: L81 Injury musculoskeletal NOS
Female
Male
Working person−days lost to medically certified sick leave (1000s)
300
200
100
0
2010
2011
2012
2013
2014
2015
2016
2017
2018
2019
2020
2021
2022
2023
Baseline (2010−2019)
Comprehensive measures (2020−2021)
Vaccine−only strategy (2022−2023)
All numbers are rescaled to have an equivalent population to 2023.
Shaded area represents 90% prediction interval.

as. NAV: N01 Headache

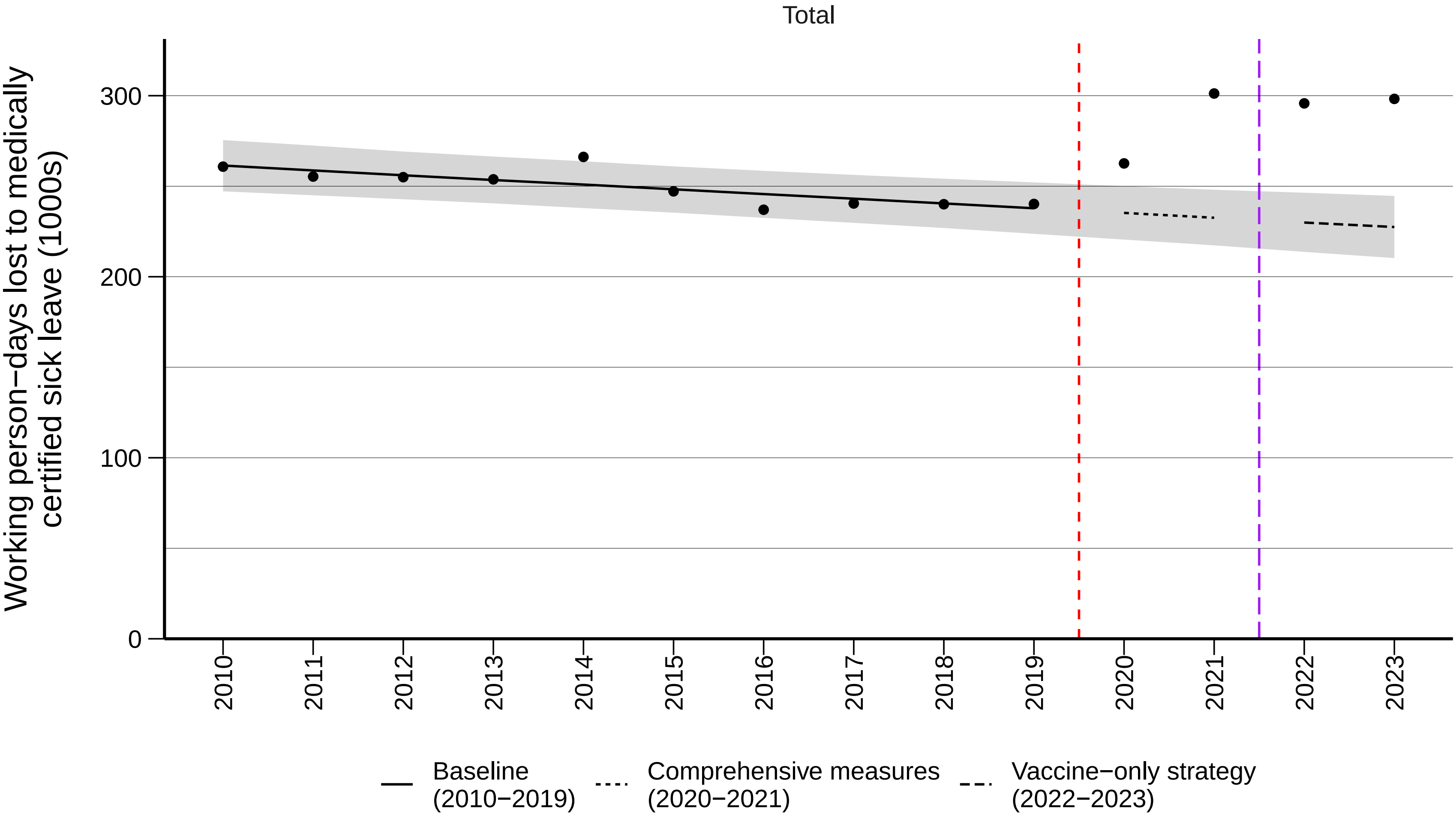

All numbers are rescaled to have an equivalent population to 2023.
Shaded area represents 90% prediction interval.

at. NAV: N01 Headache

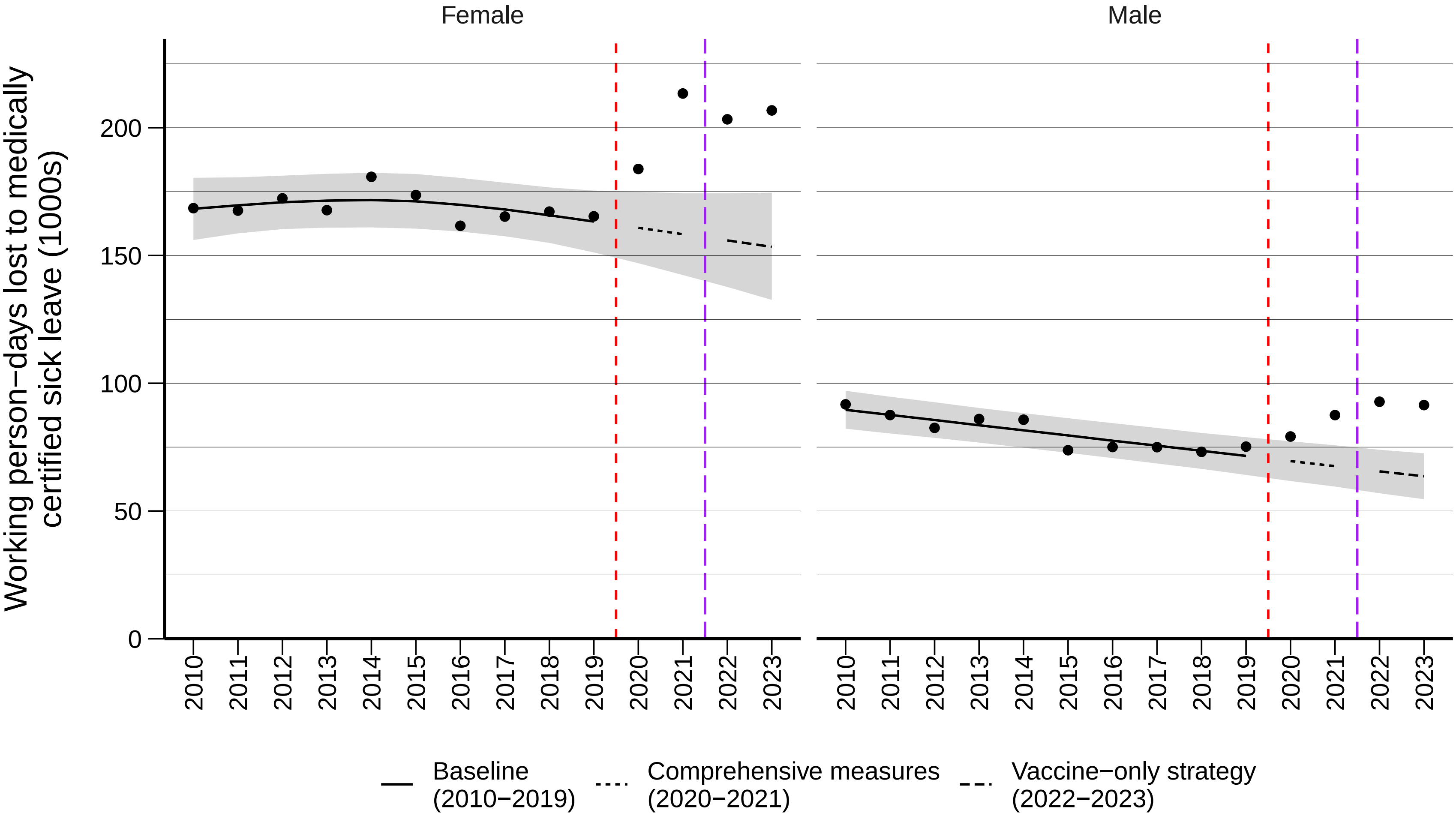

All numbers are rescaled to have an equivalent population to 2023.
Shaded area represents 90% prediction interval.

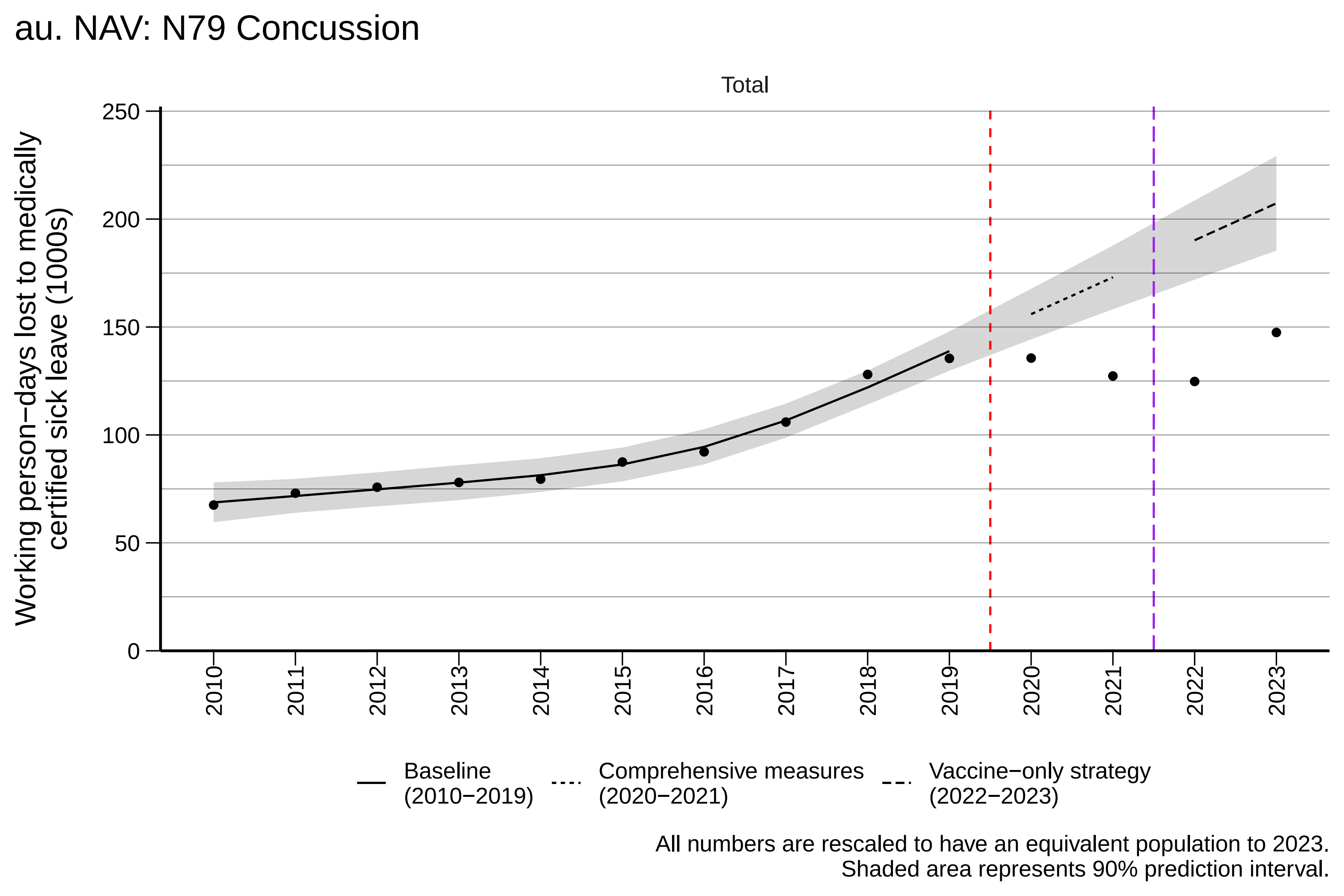
au. NAV: N79 Concussion
Total
Working person−days lost to medically certified sick leave (1000s)
0
50
100
150
200
250
2010
2011
2012
2013
2014
2015
2016
2017
2018
2019
2020
2021
2022
2023
Baseline (2010−2019)
Comprehensive measures (2020−2021)
Vaccine−only strategy (2022−2023)
All numbers are rescaled to have an equivalent population to 2023.
Shaded area represents 90% prediction interval.

av. NAV: N79 Concussion

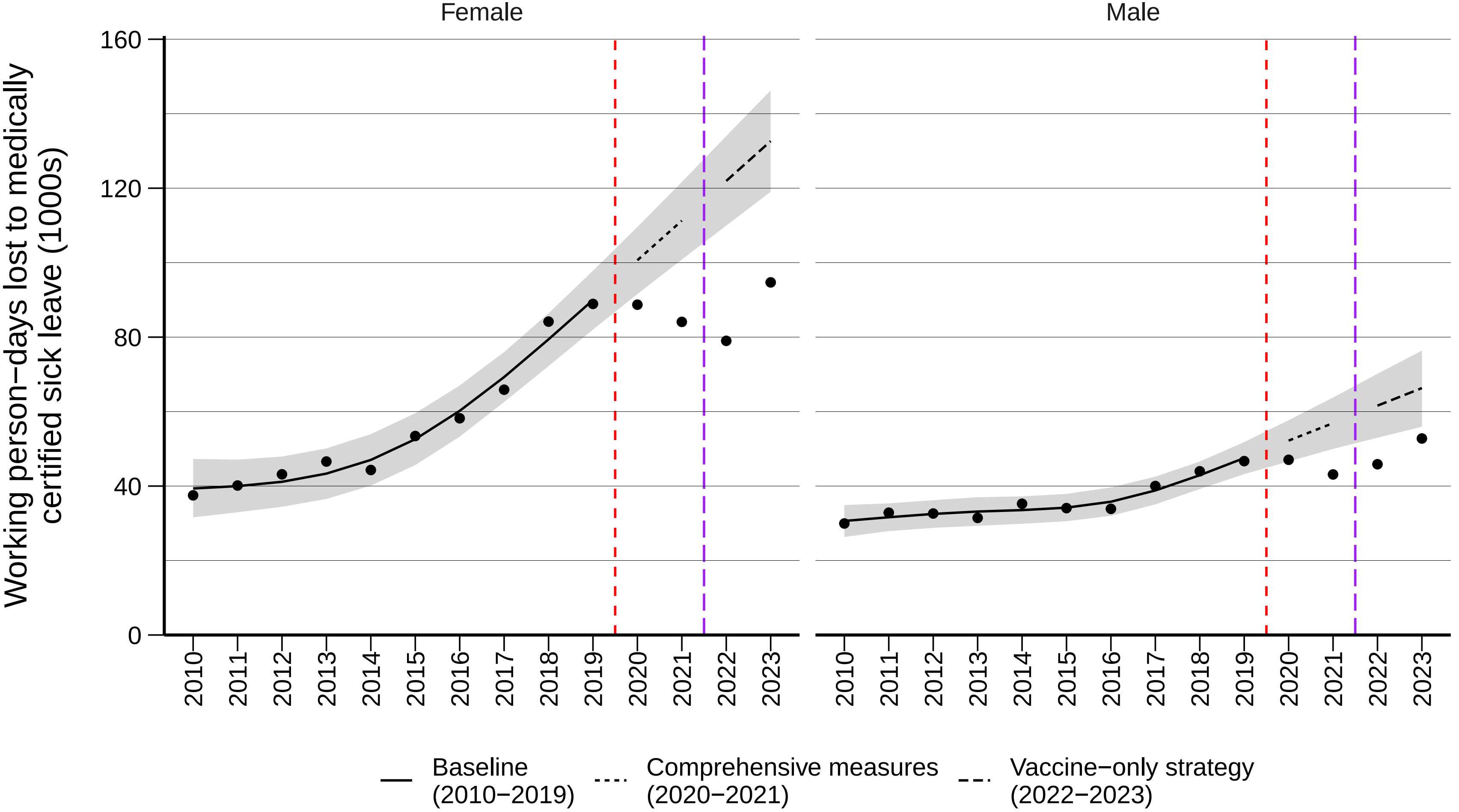

All numbers are rescaled to have an equivalent population to 2023.
Shaded area represents 90% prediction interval.

aw. NAV: P02 Acute stress reaction

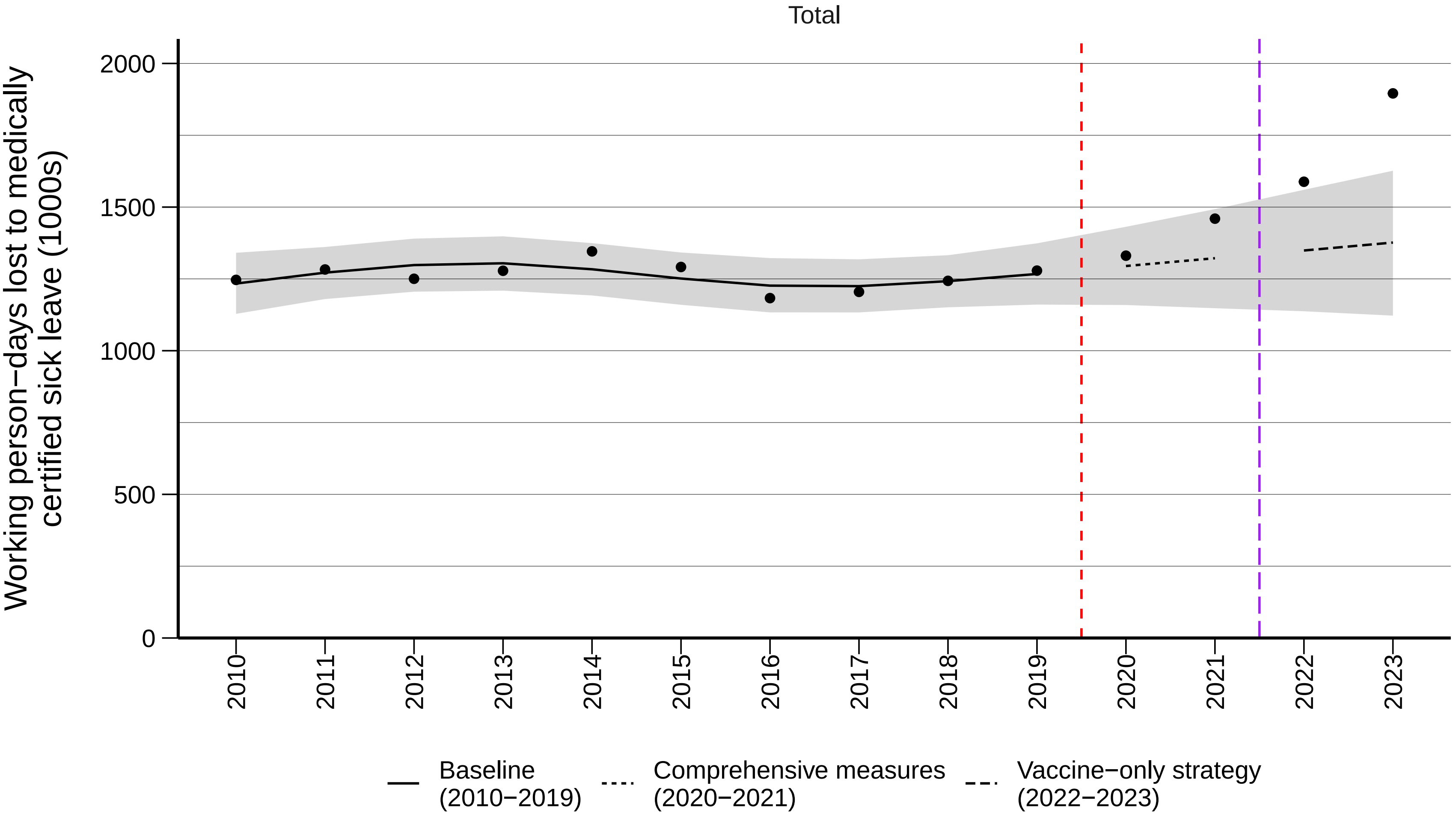

All numbers are rescaled to have an equivalent population to 2023.
Shaded area represents 90% prediction interval.

ax. NAV: P02 Acute stress reaction

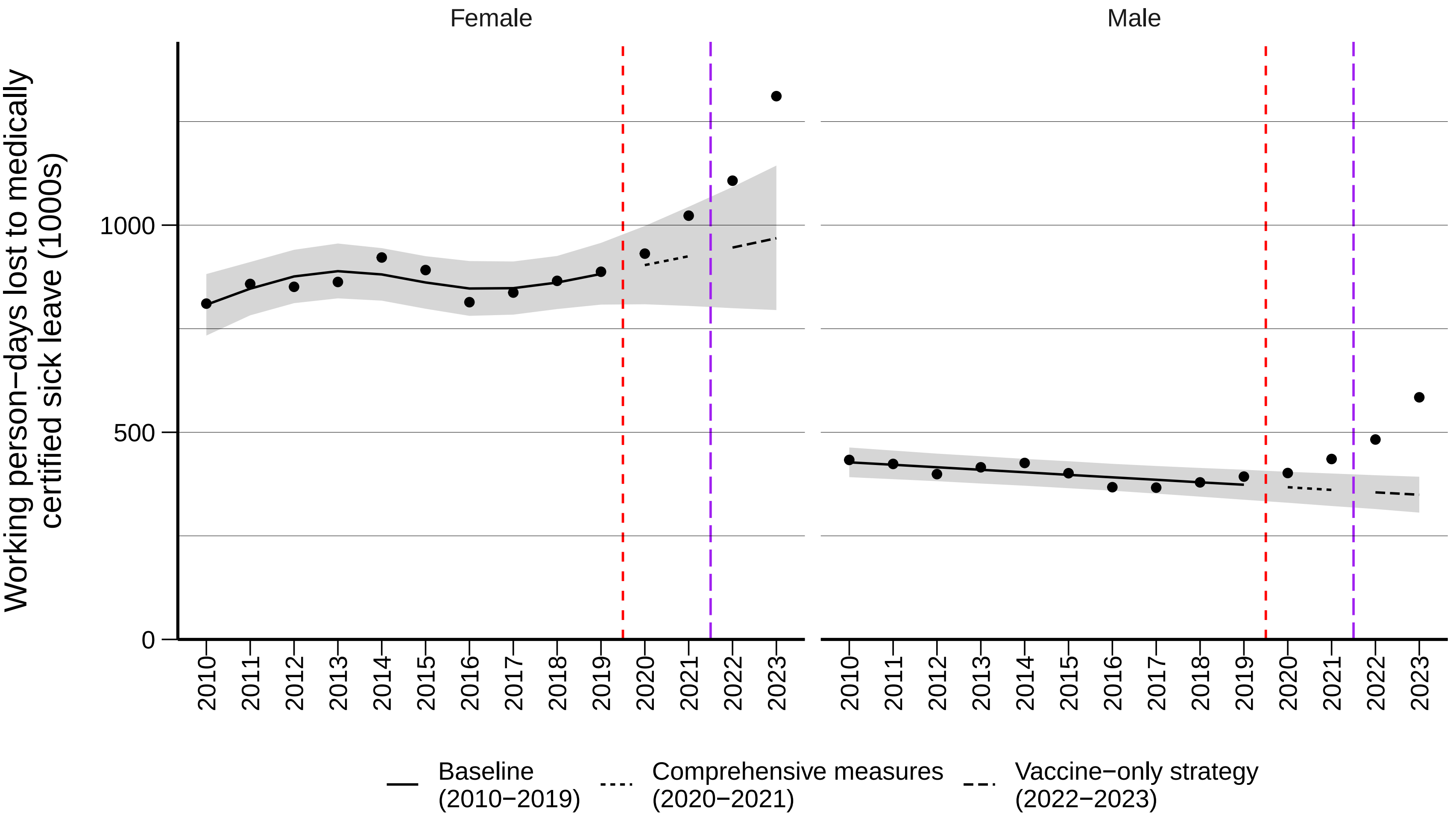

All numbers are rescaled to have an equivalent population to 2023.
Shaded area represents 90% prediction interval.

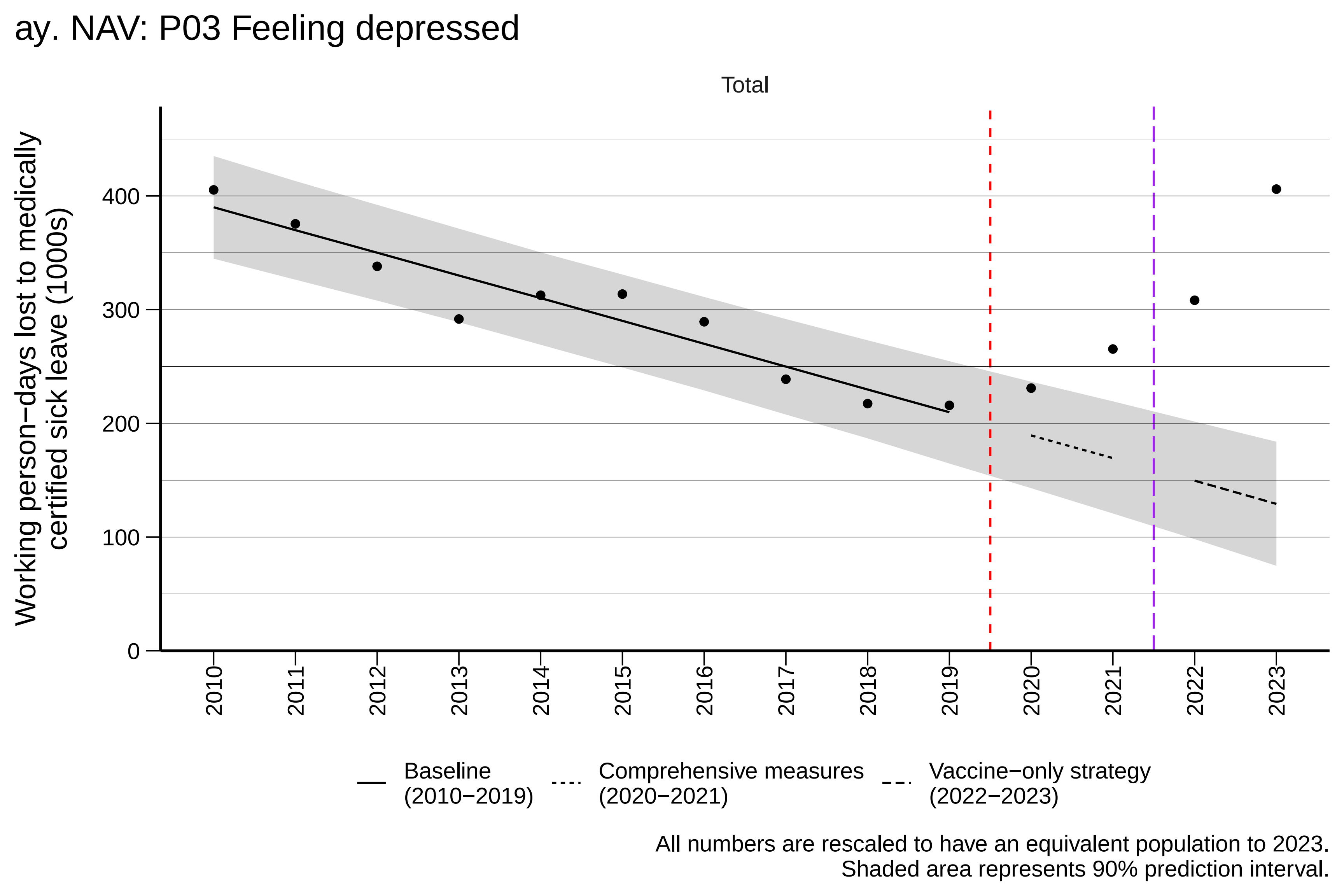
ay. NAV: P03 Feeling depressed
Total
Working person−days lost to medically certified sick leave (1000s)
0
100
200
300
400
2010
2011
2012
2013
2014
2015
2016
2017
2018
2019
2020
2021
2022
2023
Baseline (2010−2019)
Comprehensive measures (2020−2021)
Vaccine−only strategy (2022−2023)
All numbers are rescaled to have an equivalent population to 2023.
Shaded area represents 90% prediction interval.

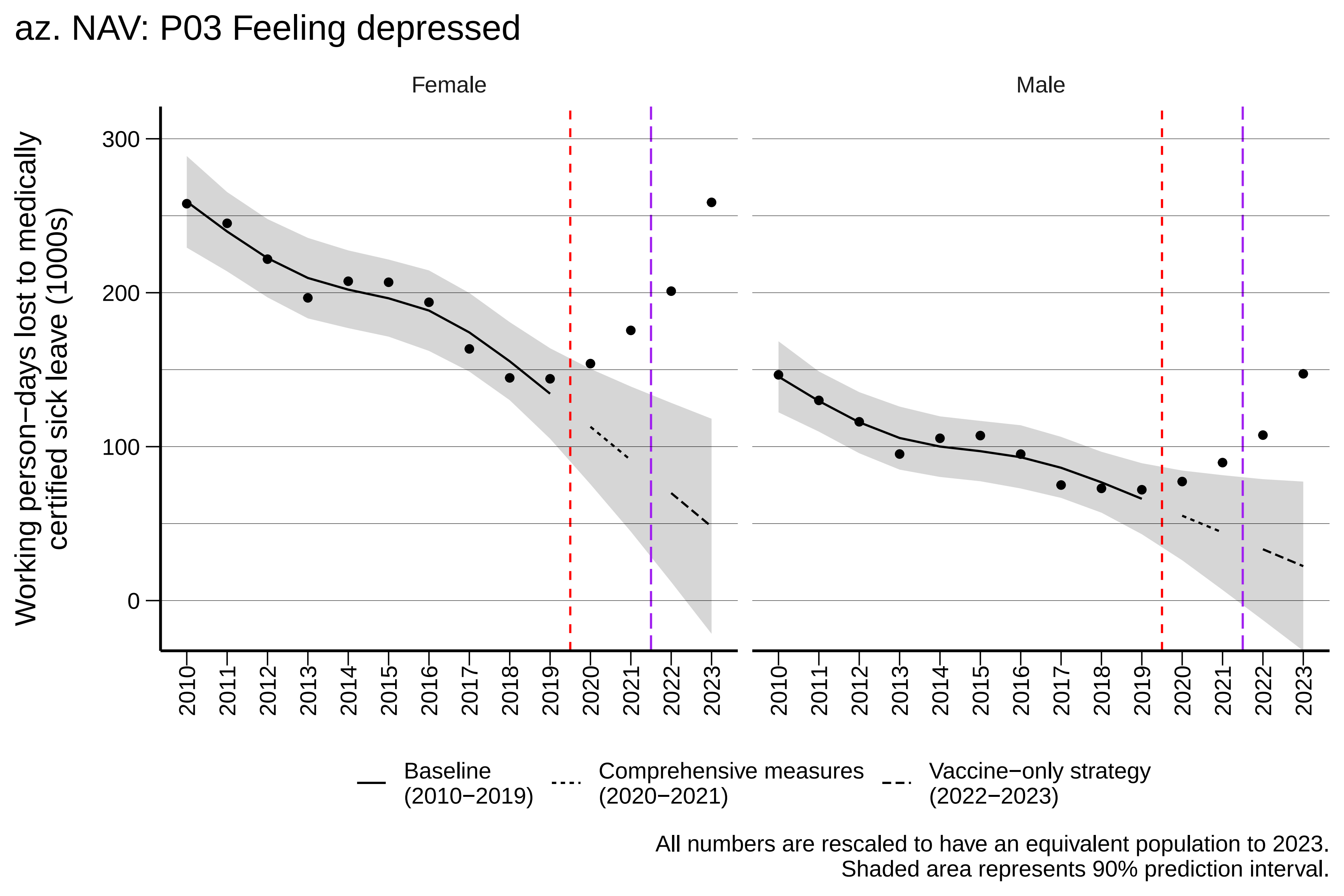
az. NAV: P03 Feeling depressed
Female
Male
Working person−days lost to medically certified sick leave (1000s)
300
200
100
0
2010
2011
2012
2013
2014
2015
2016
2017
2018
2019
2020
2021
2022
2023
Baseline (2010−2019)
Comprehensive measures (2020−2021)
Vaccine−only strategy (2022−2023)
All numbers are rescaled to have an equivalent population to 2023.
Shaded area represents 90% prediction interval.

ba. NAV: P19 Drug abuse

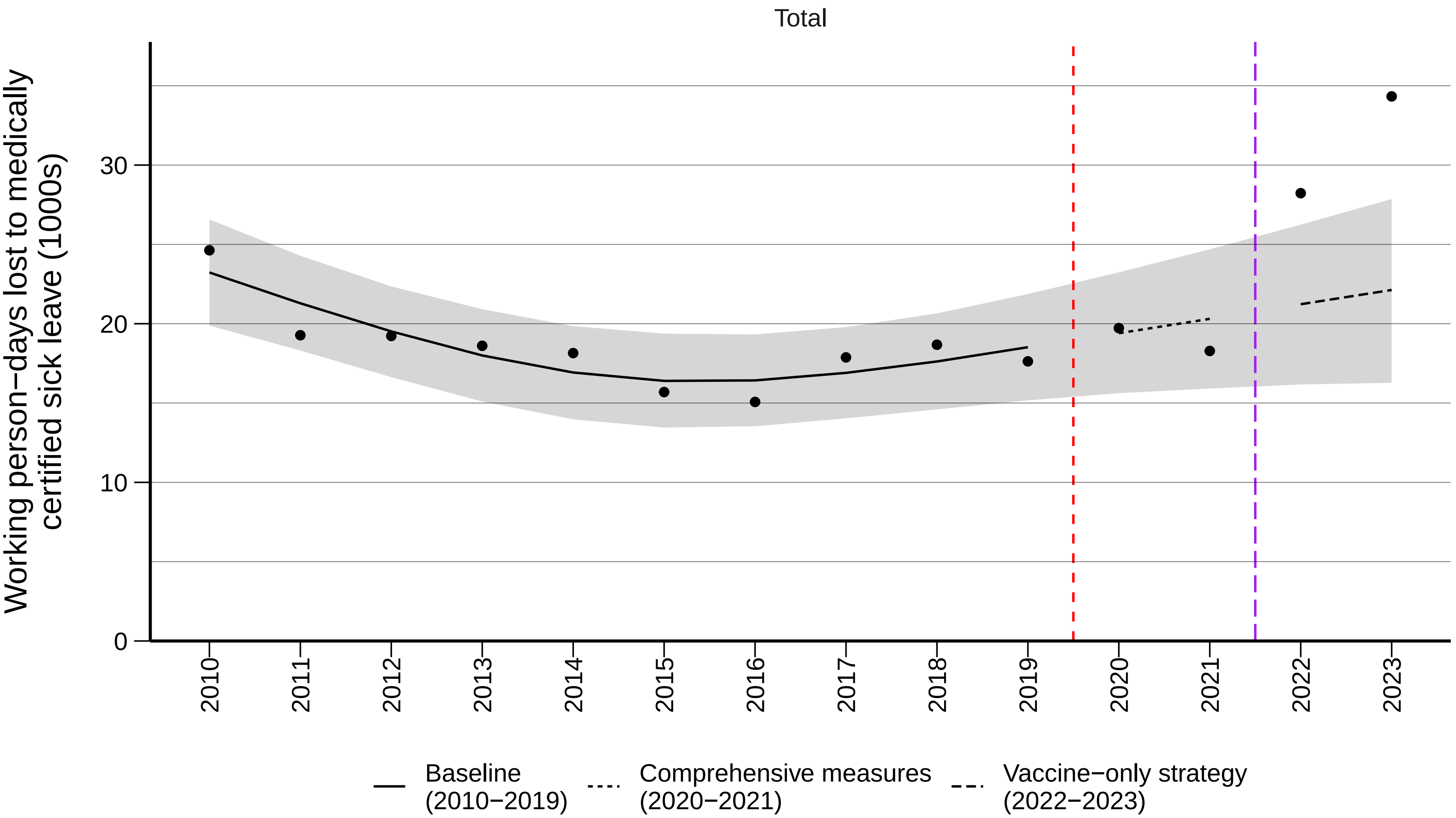

All numbers are rescaled to have an equivalent population to 2023.
Shaded area represents 90% prediction interval.

bb. NAV: P19 Drug abuse

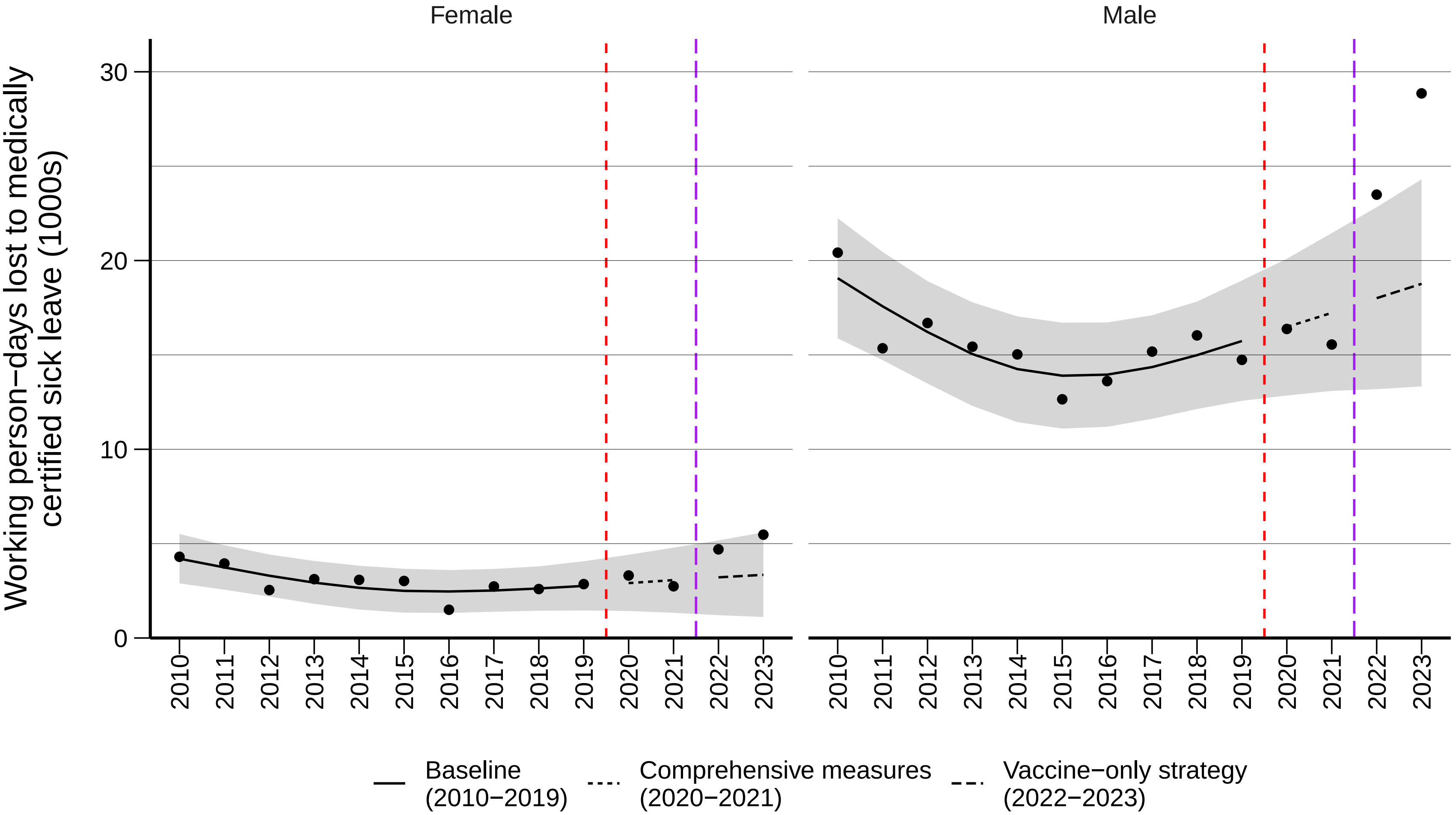

All numbers are rescaled to have an equivalent population to 2023.
Shaded area represents 90% prediction interval.

bc. NAV: P29 Psychological symptom/complt other

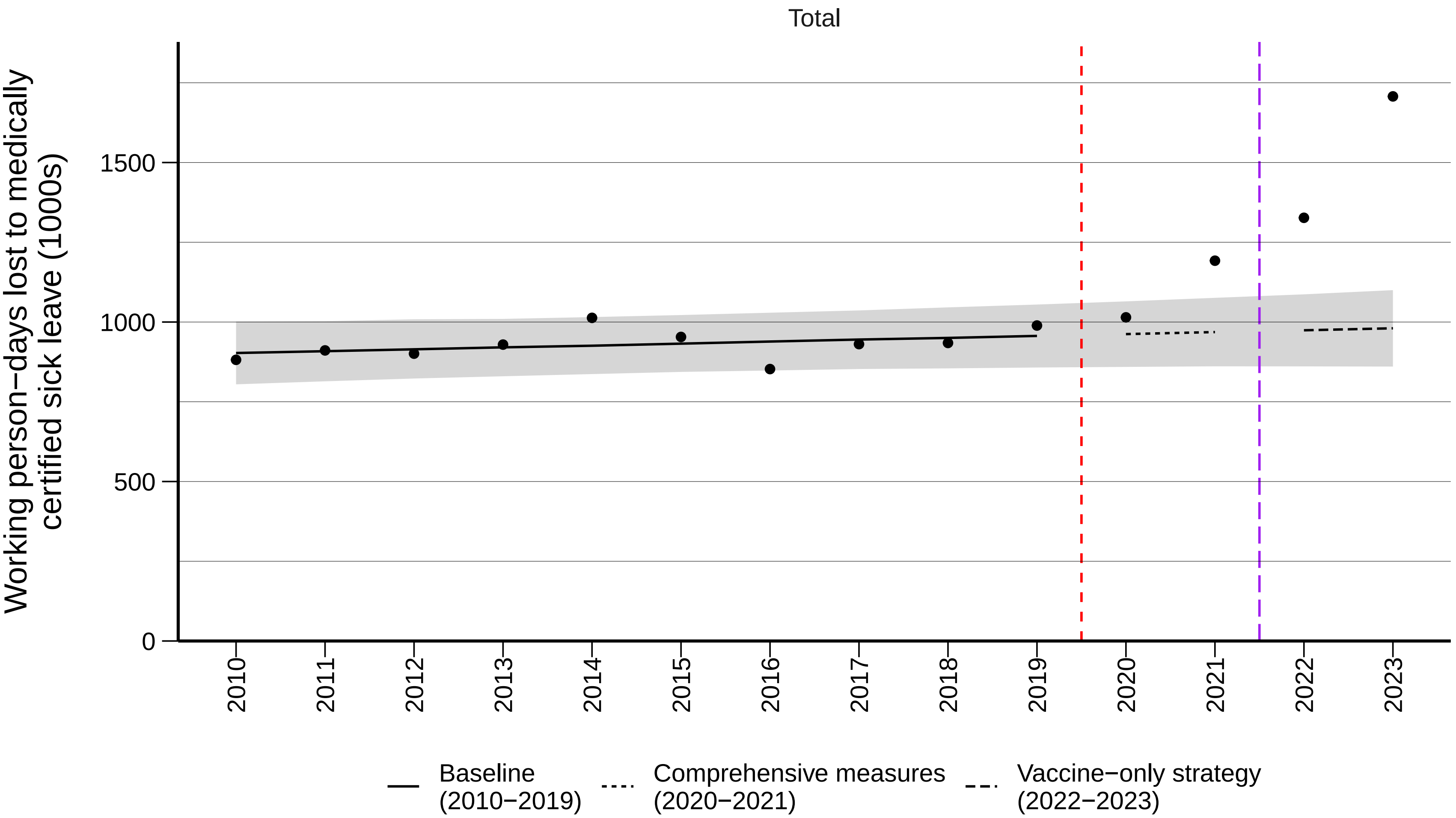

All numbers are rescaled to have an equivalent population to 2023.
Shaded area represents 90% prediction interval.

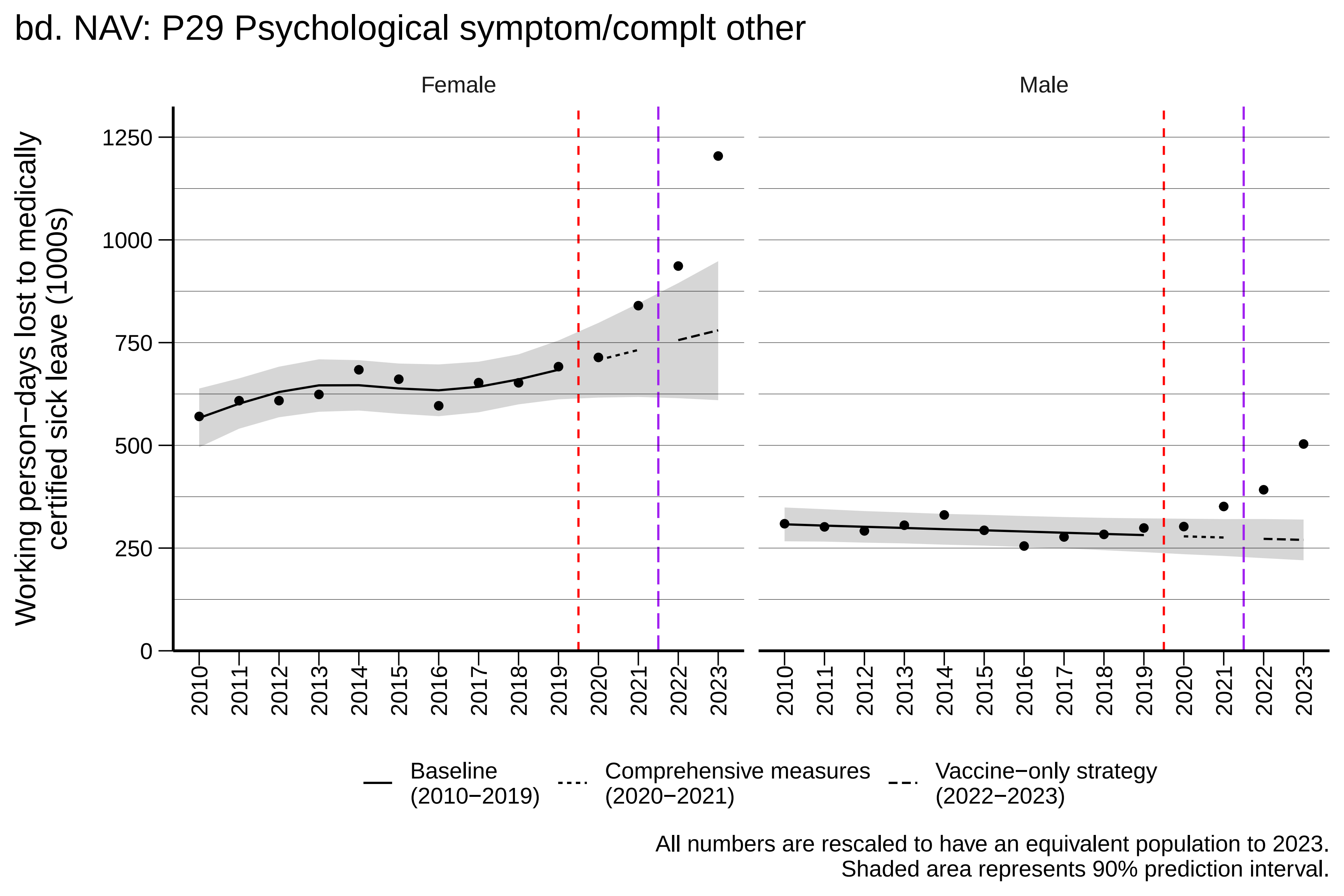

bd. NAV: P29 Psychological symptom/complt other
Female
Male
Working person−days lost to medically certified sick leave (1000s)
1250
1000
750
500
250
0
2010
2011
2012
2013
2014
2015
2016
2017
2018
2019
2020
2021
2022
2023
Baseline (2010−2019)
Comprehensive measures (2020−2021)
Vaccine−only strategy (2022−2023)
All numbers are rescaled to have an equivalent population to 2023.
Shaded area represents 90% prediction interval.

be. NAV: P81 Hyperkinetic disorder

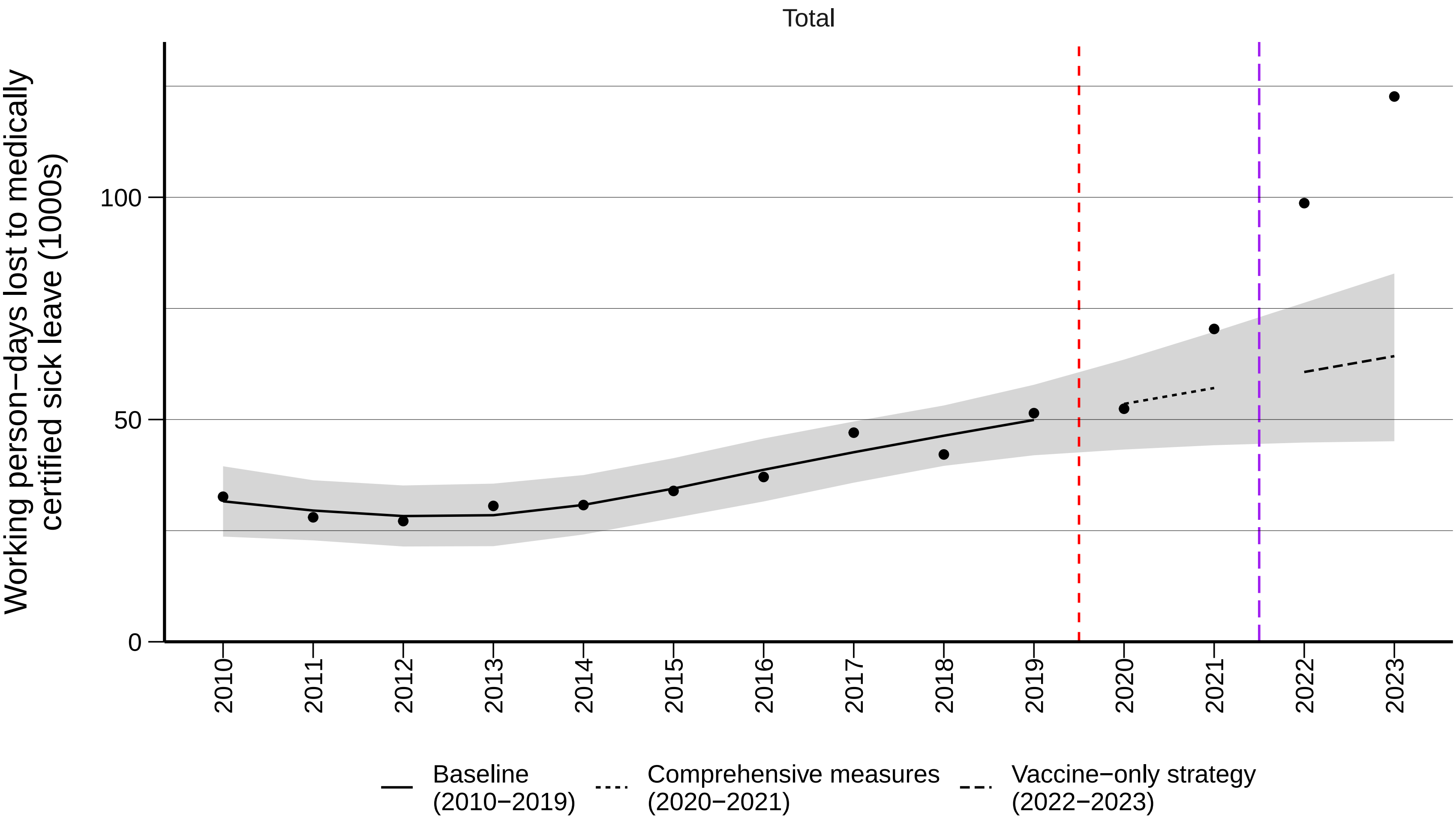

All numbers are rescaled to have an equivalent population to 2023.
Shaded area represents 90% prediction interval.

bf. NAV: P81 Hyperkinetic disorder

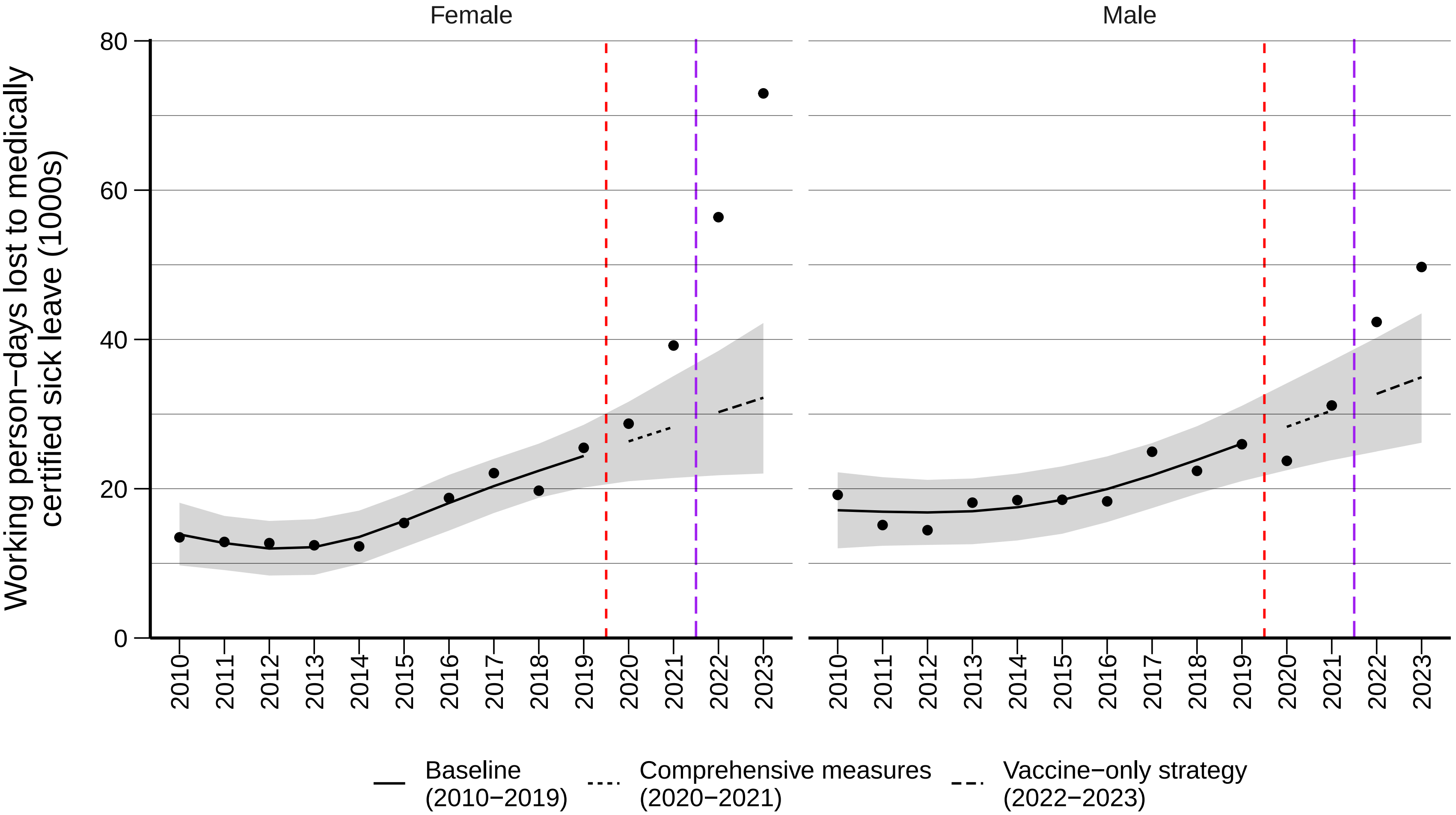

All numbers are rescaled to have an equivalent population to 2023.
Shaded area represents 90% prediction interval.

bg. NAV: R* Respiratory

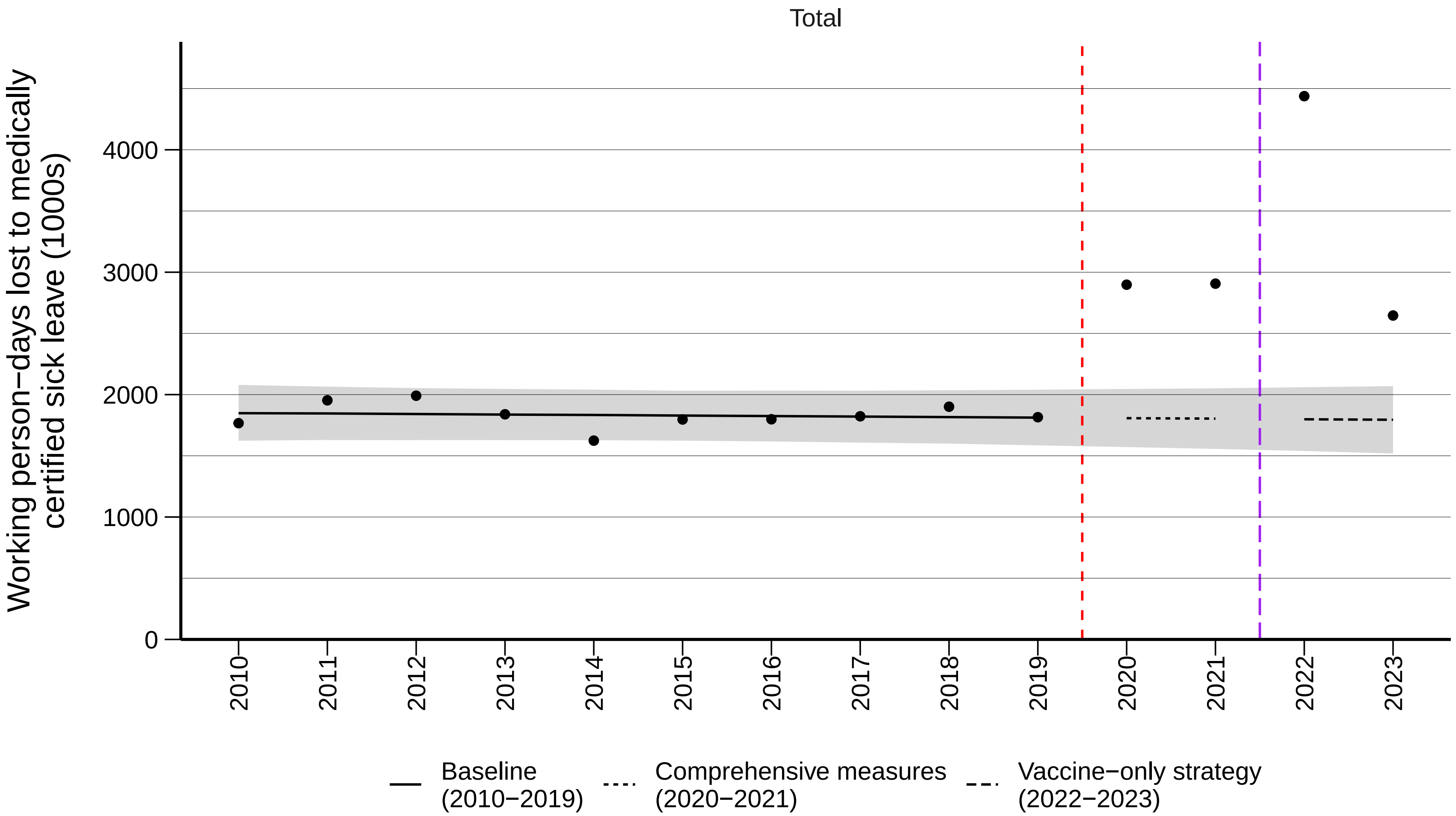

All numbers are rescaled to have an equivalent population to 2023.
Shaded area represents 90% prediction interval.

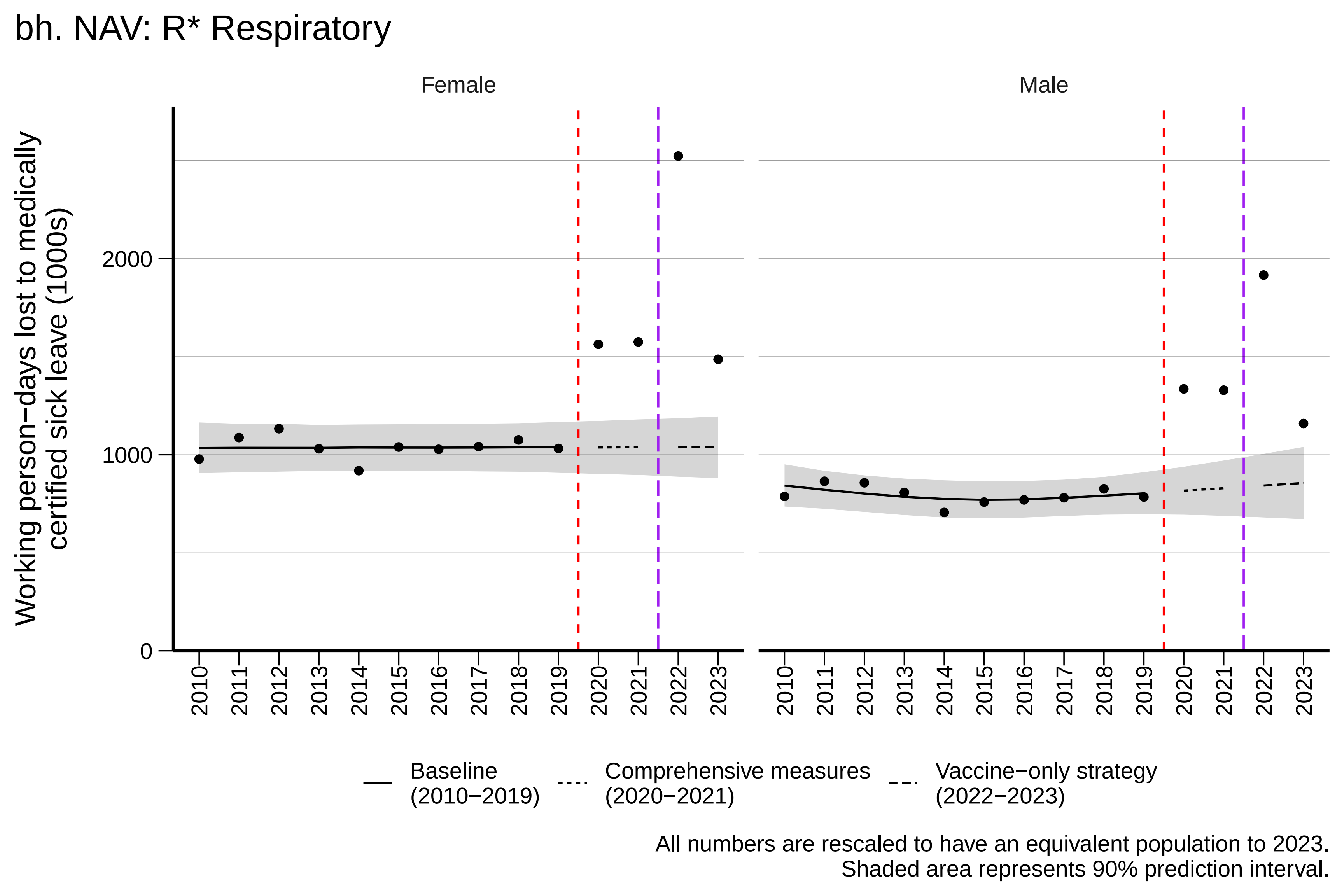

bh. NAV: R* Respiratory
Female
Male
Working person−days lost to medically certified sick leave (1000s)
0
1000
2000
2010
2011
2012
2013
2014
2015
2016
2017
2018
2019
2020
2021
2022
2023
Baseline (2010−2019)
Comprehensive measures (2020−2021)
Vaccine−only strategy (2022−2023)
All numbers are rescaled to have an equivalent population to 2023.
Shaded area represents 90% prediction interval.

bi. NAV: R72 Strep throat

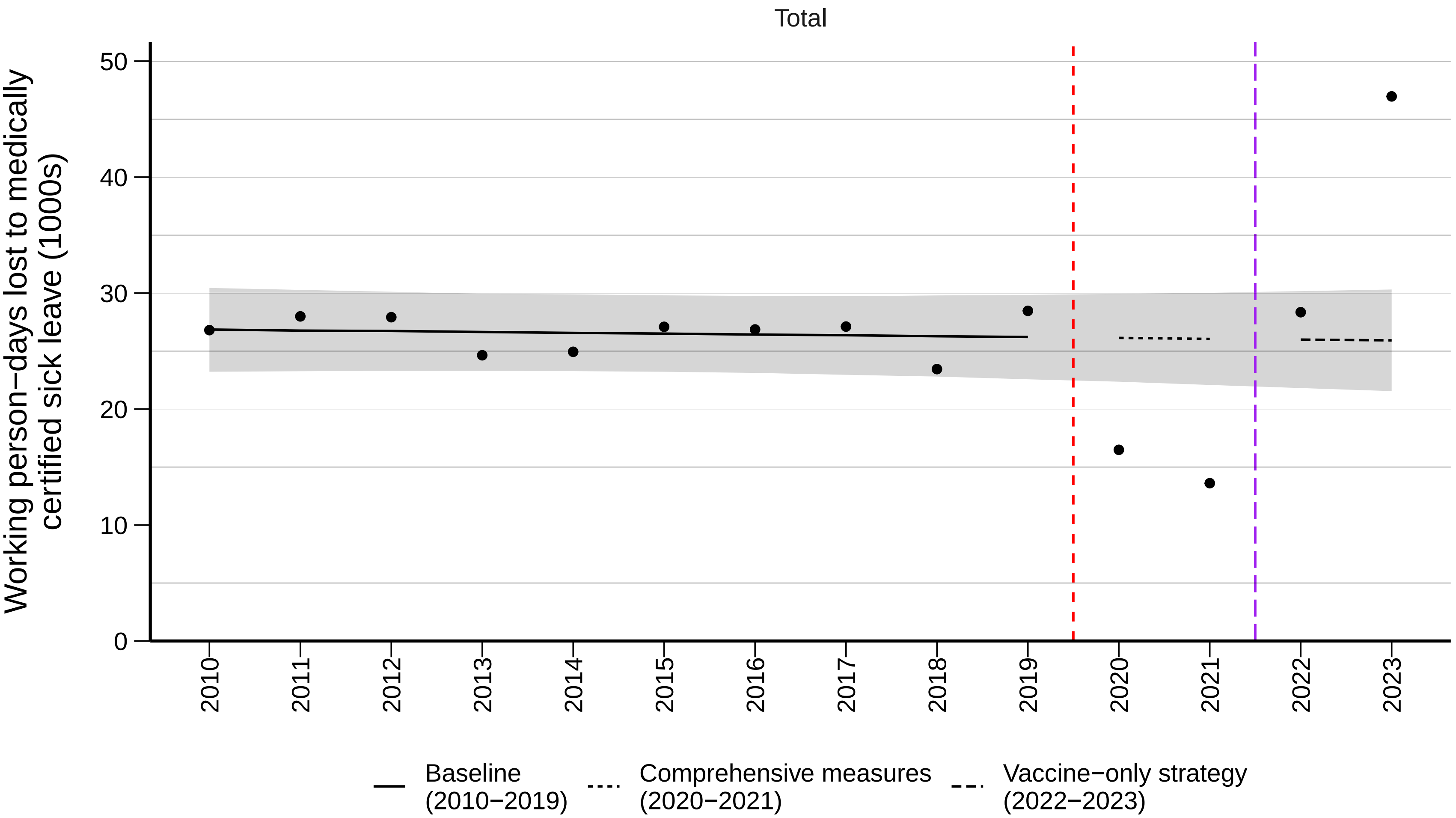

All numbers are rescaled to have an equivalent population to 2023.
Shaded area represents 90% prediction interval.

bj. NAV: R72 Strep throat

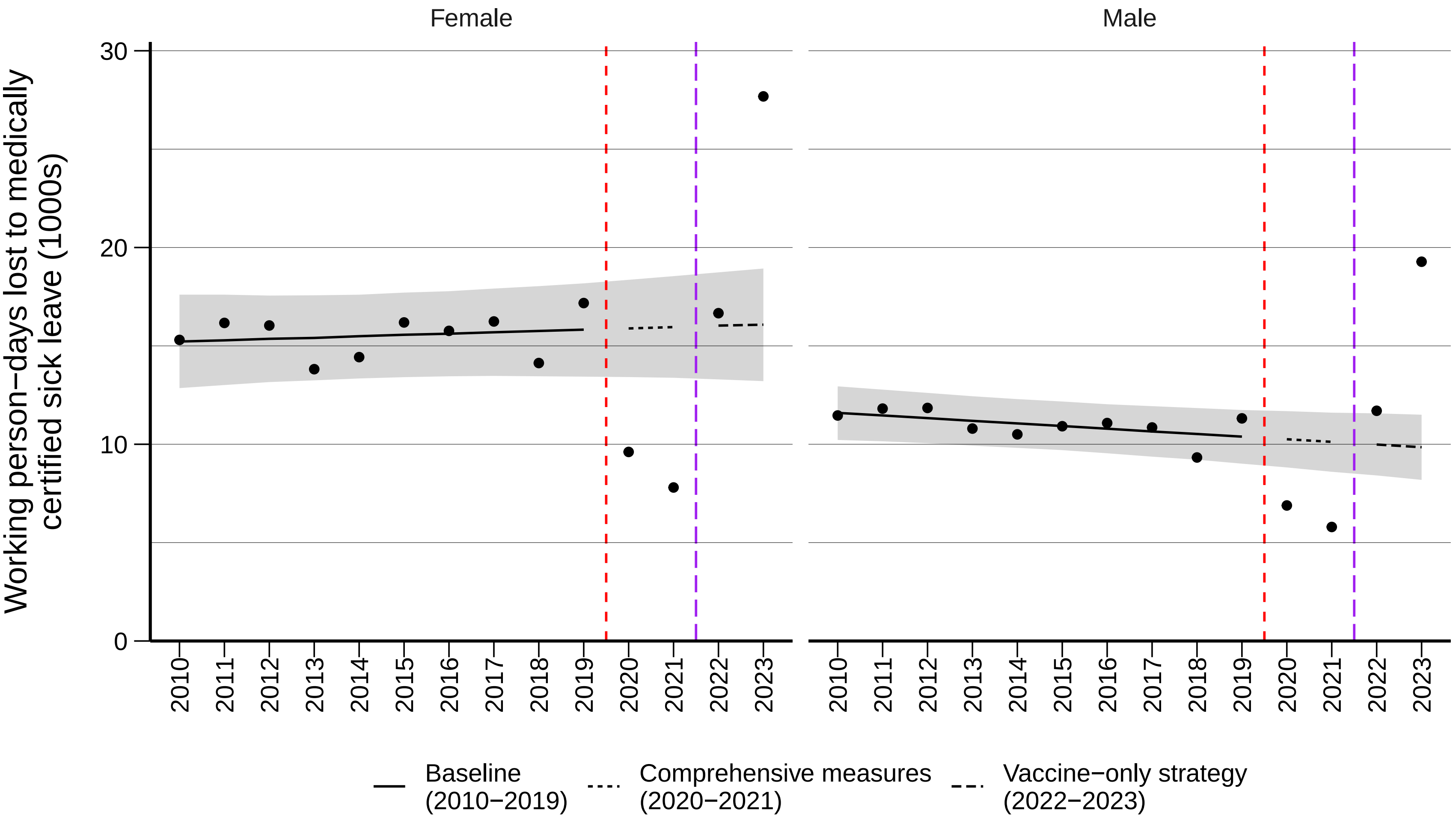

All numbers are rescaled to have an equivalent population to 2023.
Shaded area represents 90% prediction interval.

bk. NAV: R74 Upper respiratory infection acute

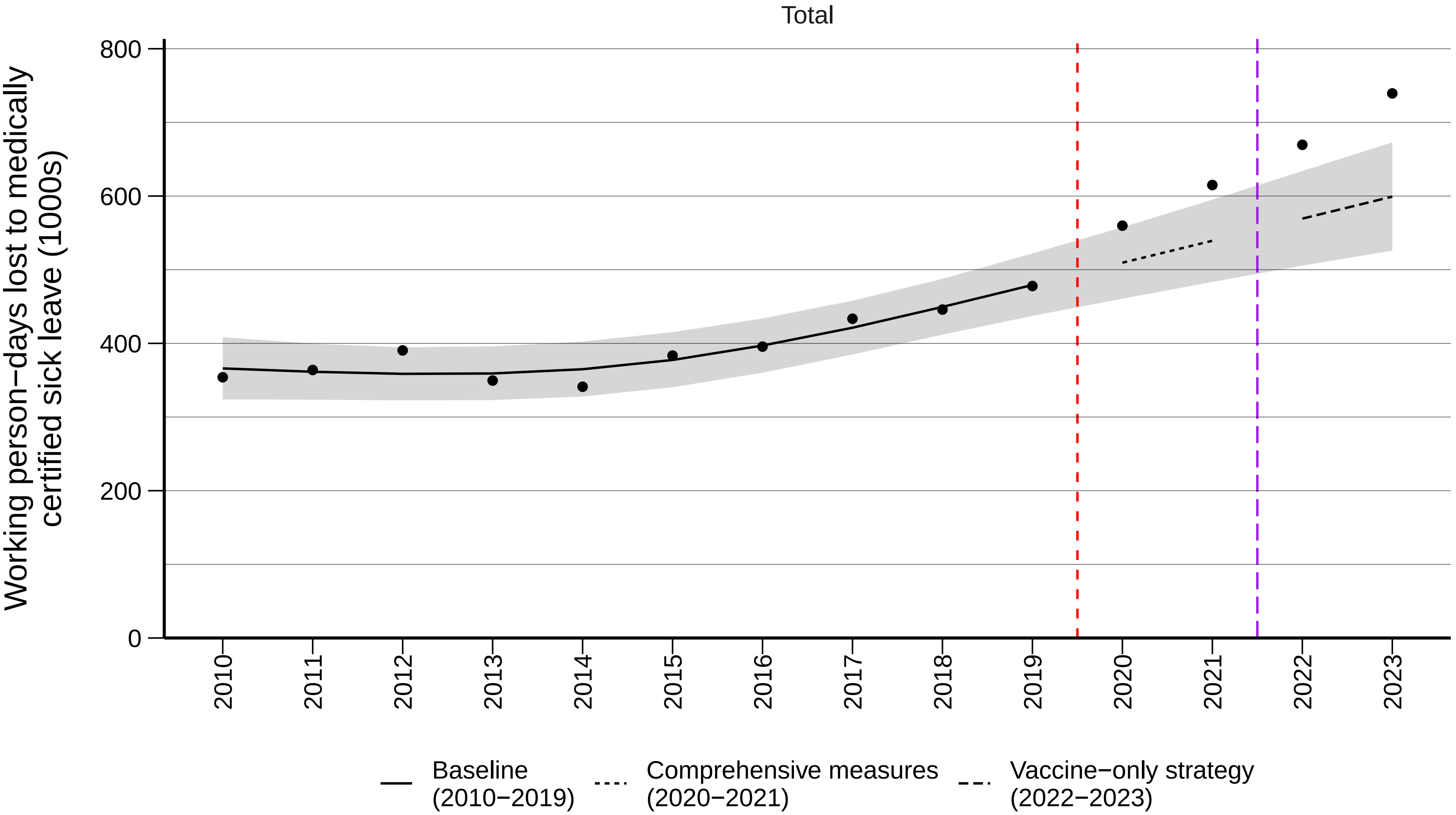

All numbers are rescaled to have an equivalent population to 2023.
Shaded area represents 90% prediction interval.

bl. NAV: R74 Upper respiratory infection acute

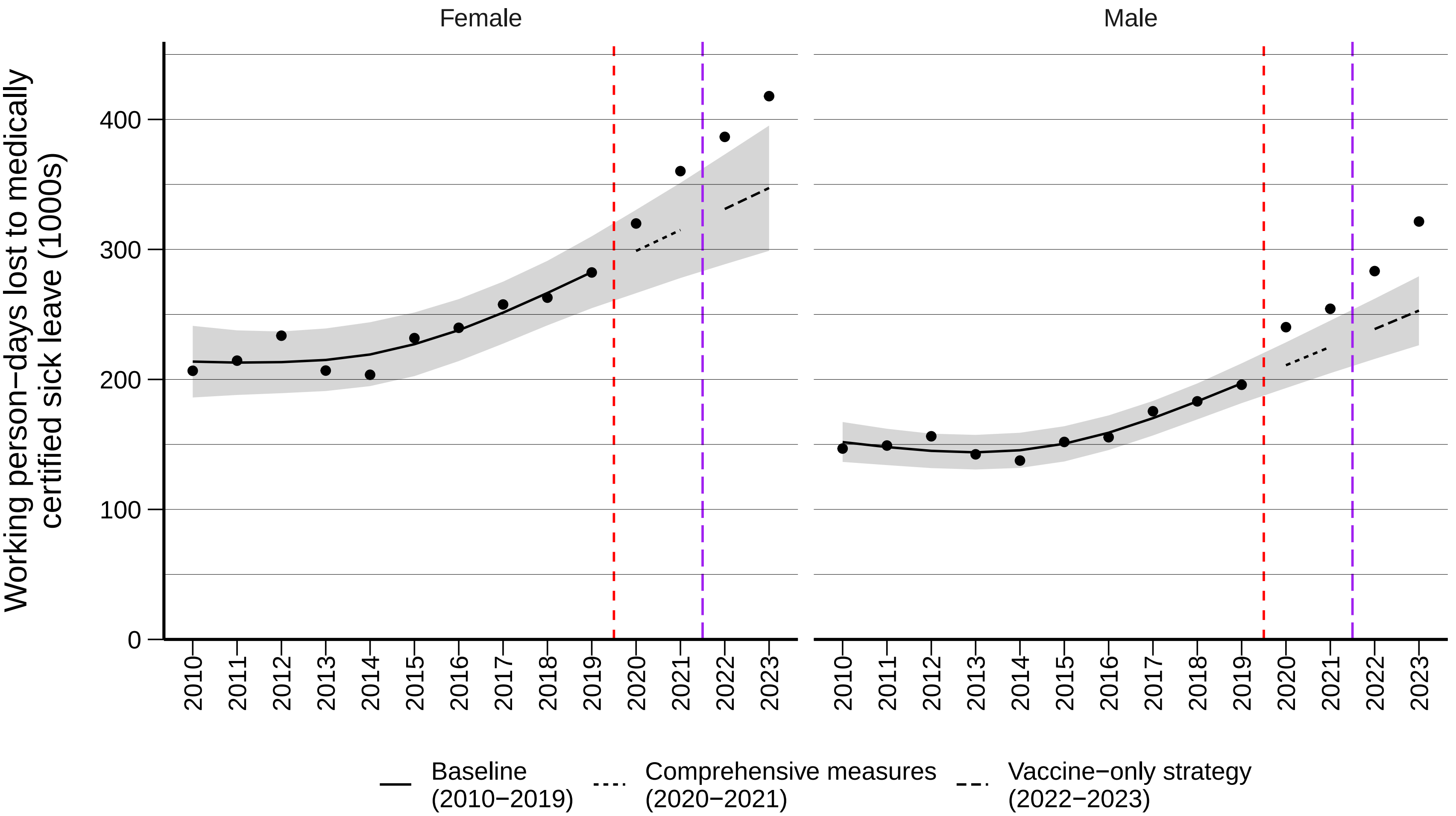

All numbers are rescaled to have an equivalent population to 2023.
Shaded area represents 90% prediction interval.

bm. NAV: R90 Hypertrophy tonsils/adenoids

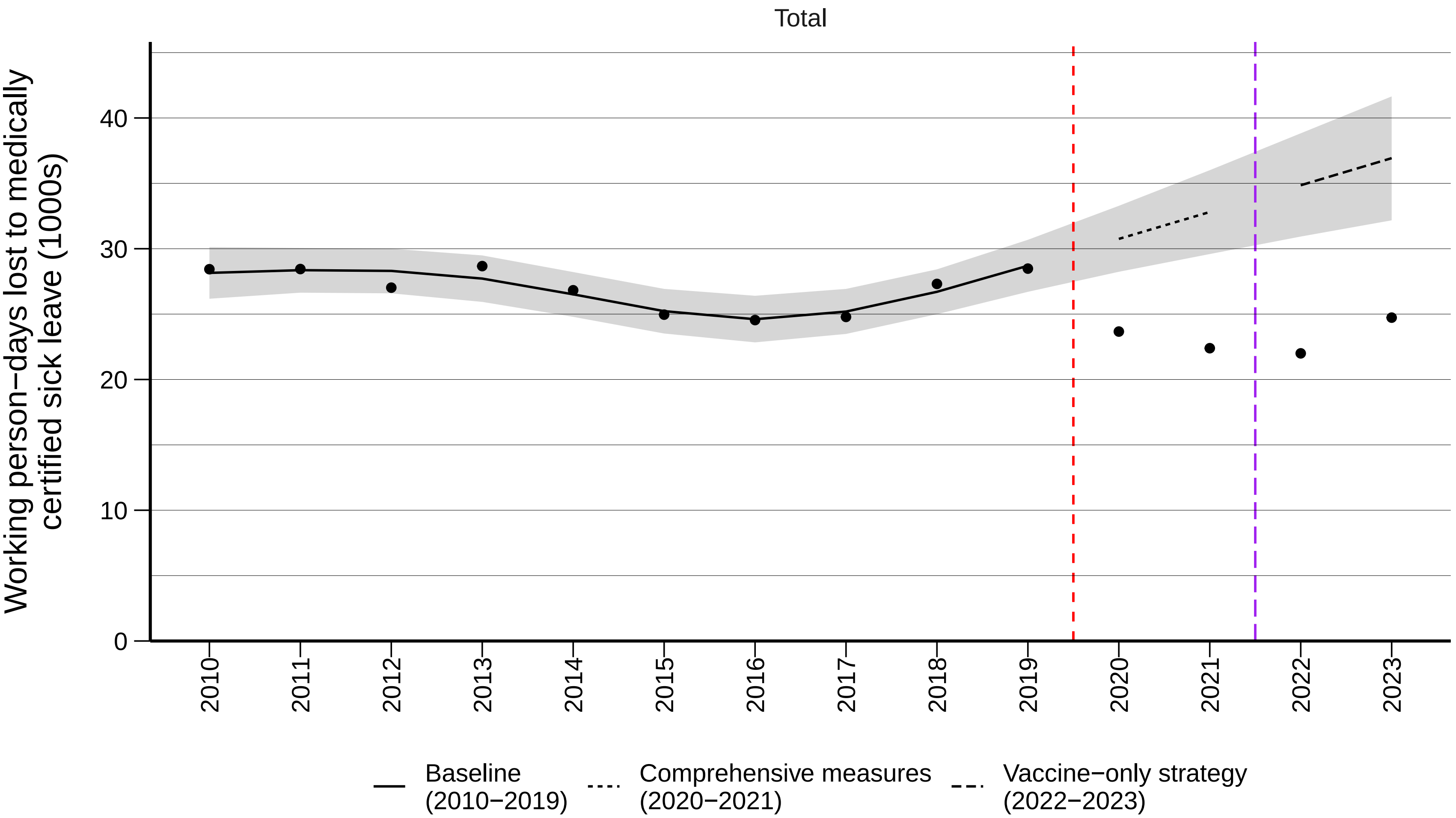

All numbers are rescaled to have an equivalent population to 2023.
Shaded area represents 90% prediction interval.

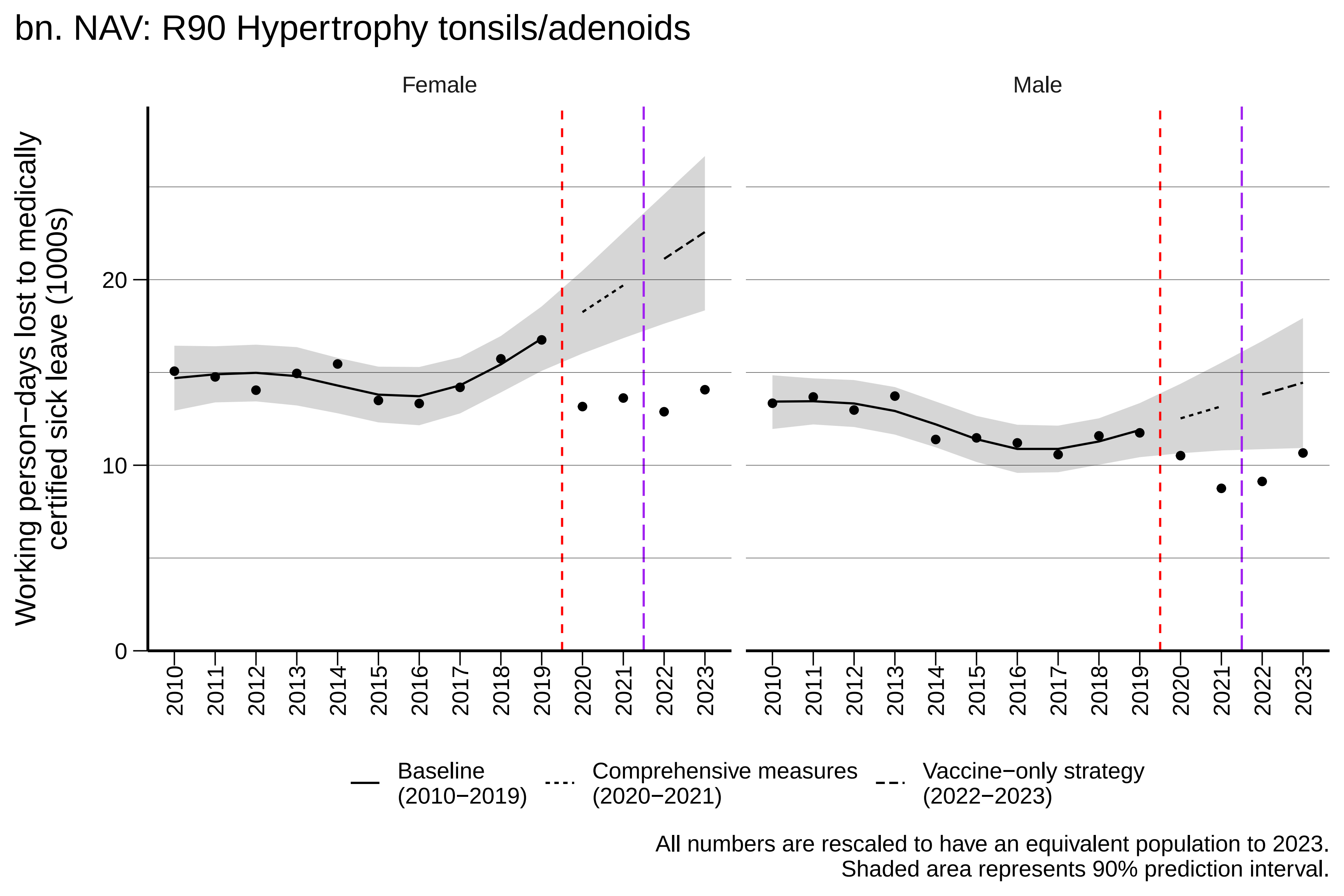

bn. NAV: R90 Hypertrophy tonsils/adenoids
Female
Male
Working person−days lost to medically certified sick leave (1000s)
0
10
20
2010
2011
2012
2013
2014
2015
2016
2017
2018
2019
2020
2021
2022
2023
Baseline (2010−2019)
Comprehensive measures (2020−2021)
Vaccine−only strategy (2022−2023)
All numbers are rescaled to have an equivalent population to 2023.
Shaded area represents 90% prediction interval.

bo. NAV: T85 Hyperthyroidism/thyrotoxicosis

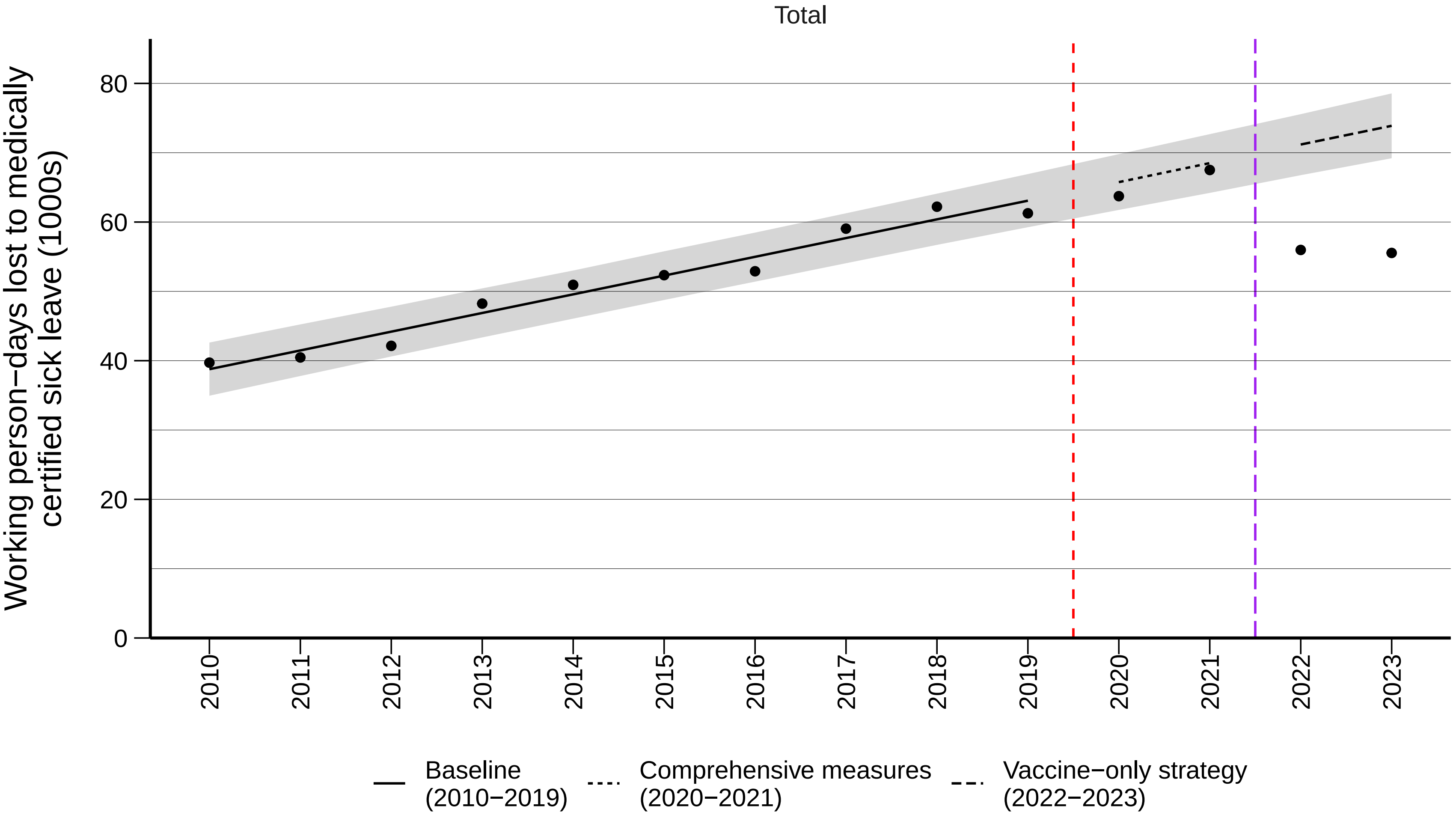

All numbers are rescaled to have an equivalent population to 2023.
Shaded area represents 90% prediction interval.

bp. NAV: T85 Hyperthyroidism/thyrotoxicosis

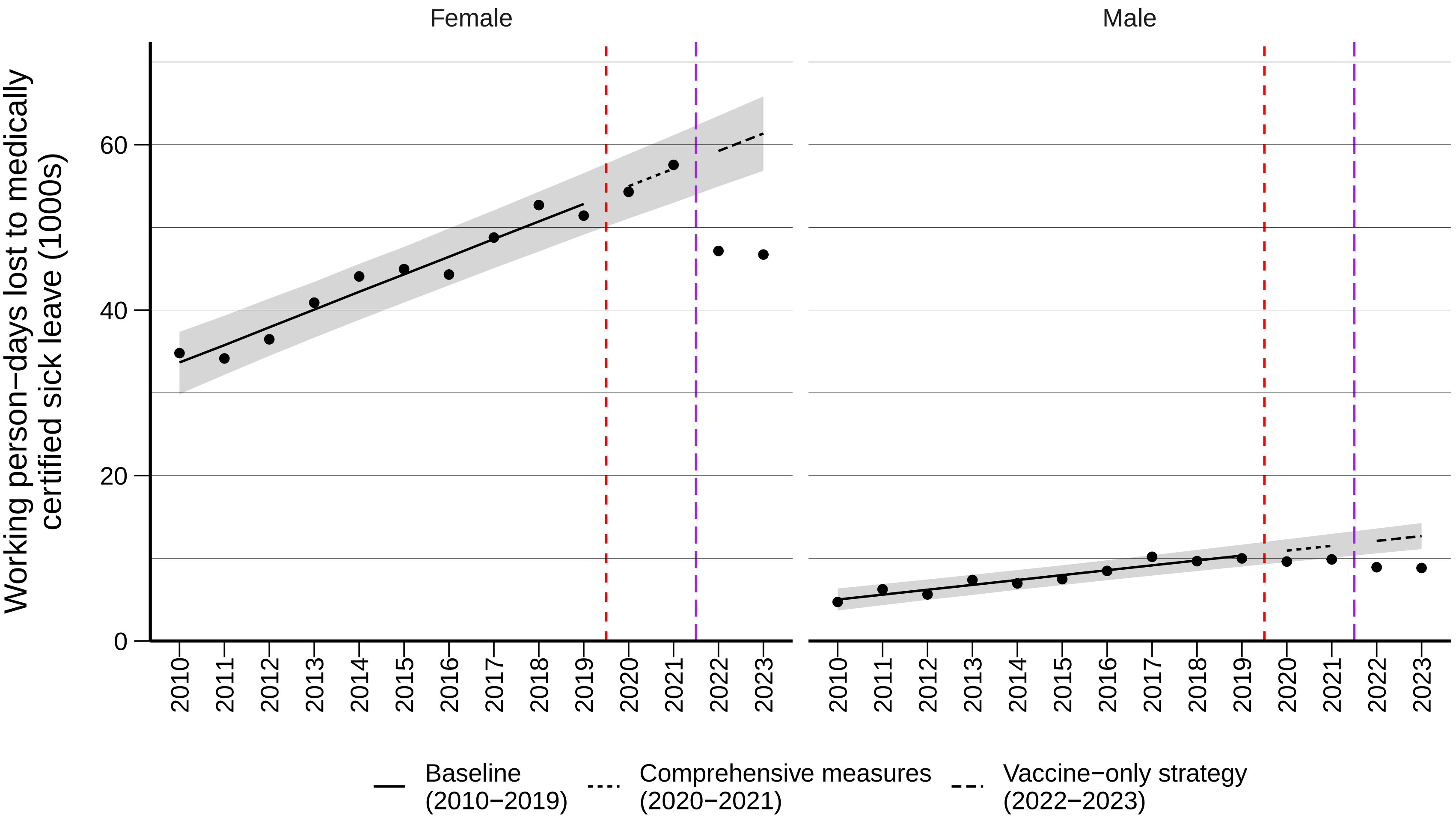

All numbers are rescaled to have an equivalent population to 2023.
Shaded area represents 90% prediction interval.

bq. NAV: T99 Endocrine/metab/nutrit. dis. other

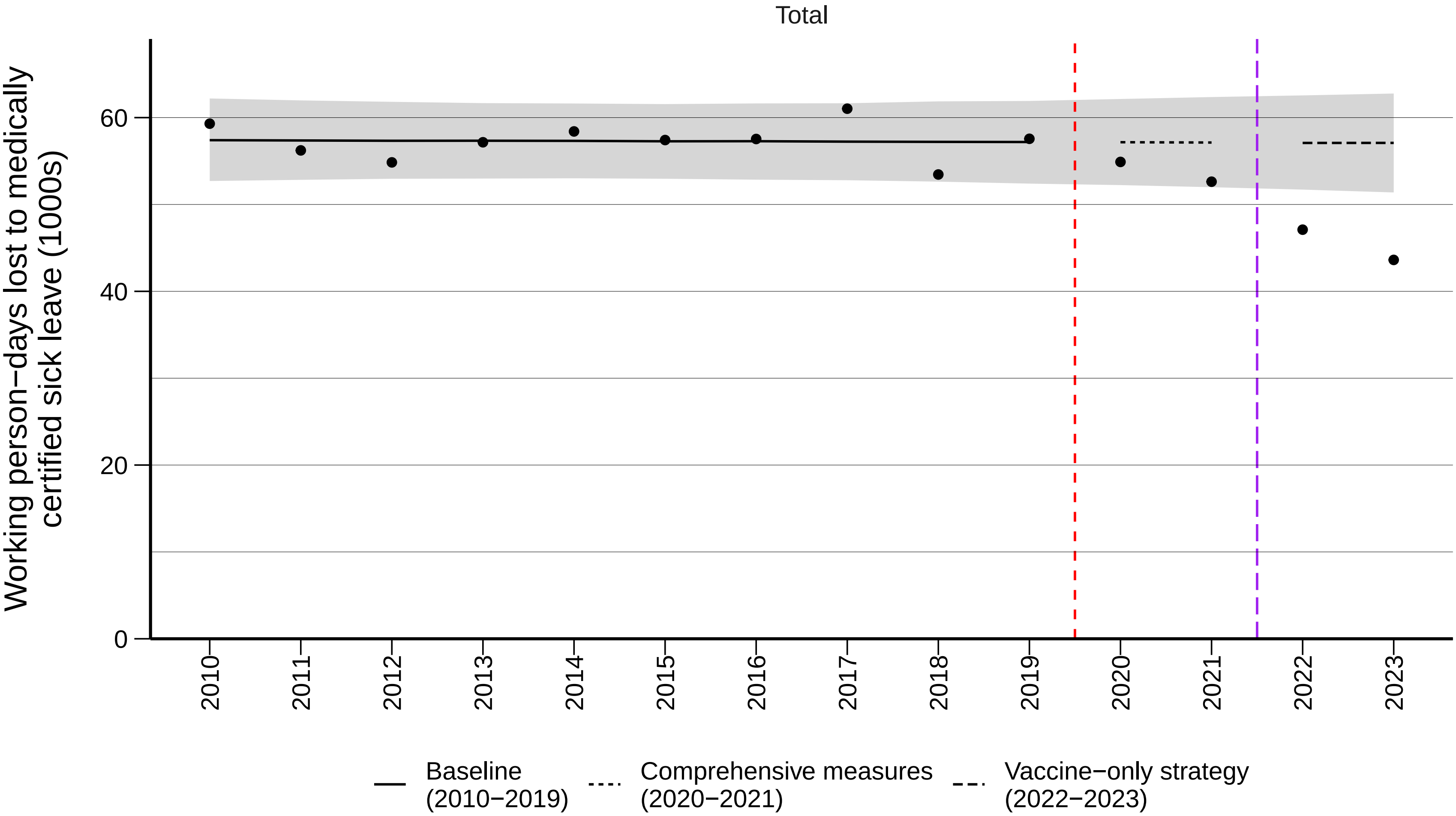

All numbers are rescaled to have an equivalent population to 2023.
Shaded area represents 90% prediction interval.

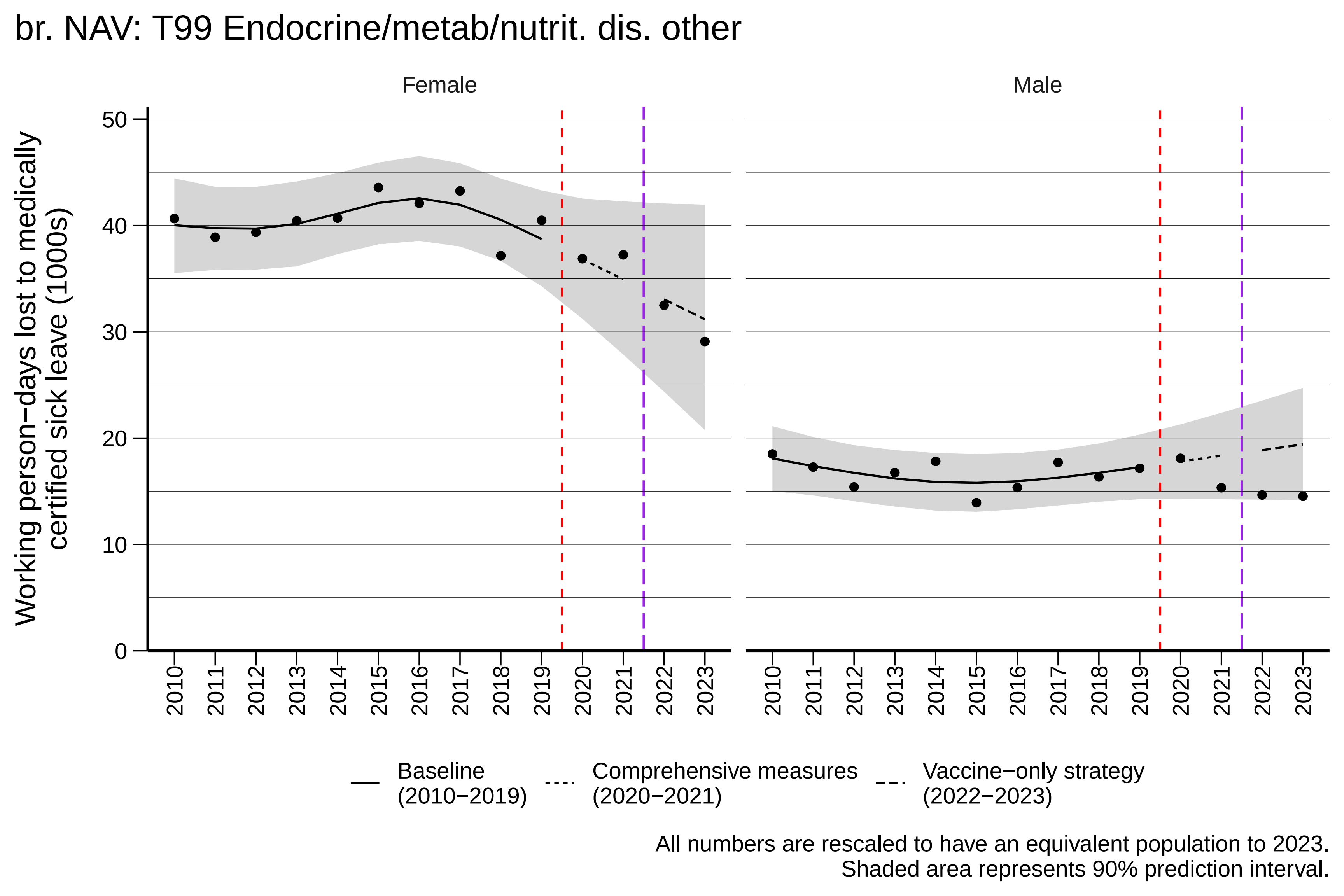

br. NAV: T99 Endocrine/metab/nutrit. dis. other
Female
Male
Working person−days lost to medically certified sick leave (1000s)
0
10
20
30
40
50
2010
2011
2012
2013
2014
2015
2016
2017
2018
2019
2020
2021
2022
2023
Baseline (2010−2019)
Comprehensive measures (2020−2021)
Vaccine−only strategy (2022−2023)
All numbers are rescaled to have an equivalent population to 2023.
Shaded area represents 90% prediction interval.

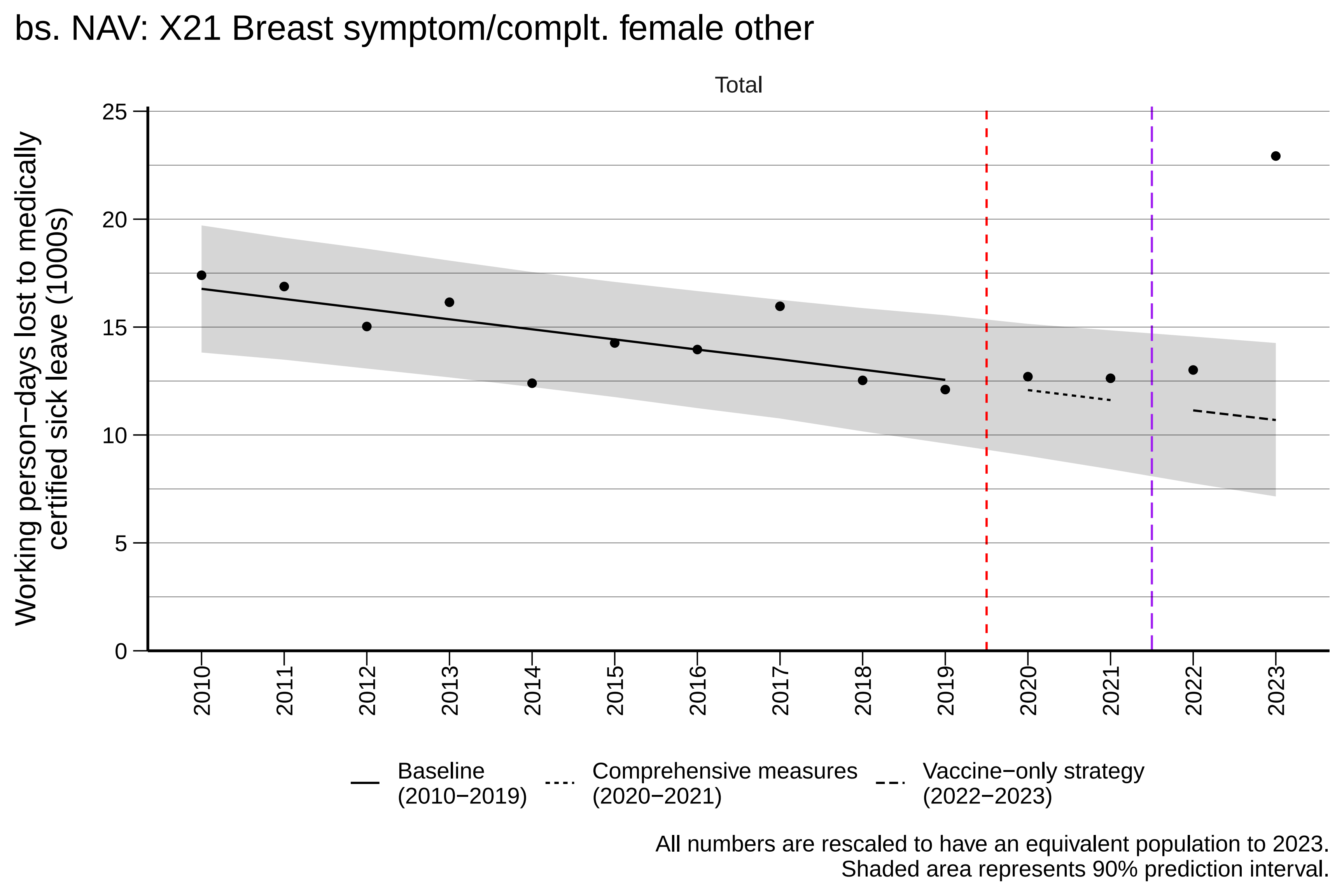

bs. NAV: X21 Breast symptom/complt. female other
Total
Working person−days lost to medically certified sick leave (1000s)
0
5
10
15
20
25
2010
2011
2012
2013
2014
2015
2016
2017
2018
2019
2020
2021
2022
2023
Baseline (2010−2019)
Comprehensive measures (2020−2021)
Vaccine−only strategy (2022−2023)
All numbers are rescaled to have an equivalent population to 2023.
Shaded area represents 90% prediction interval.

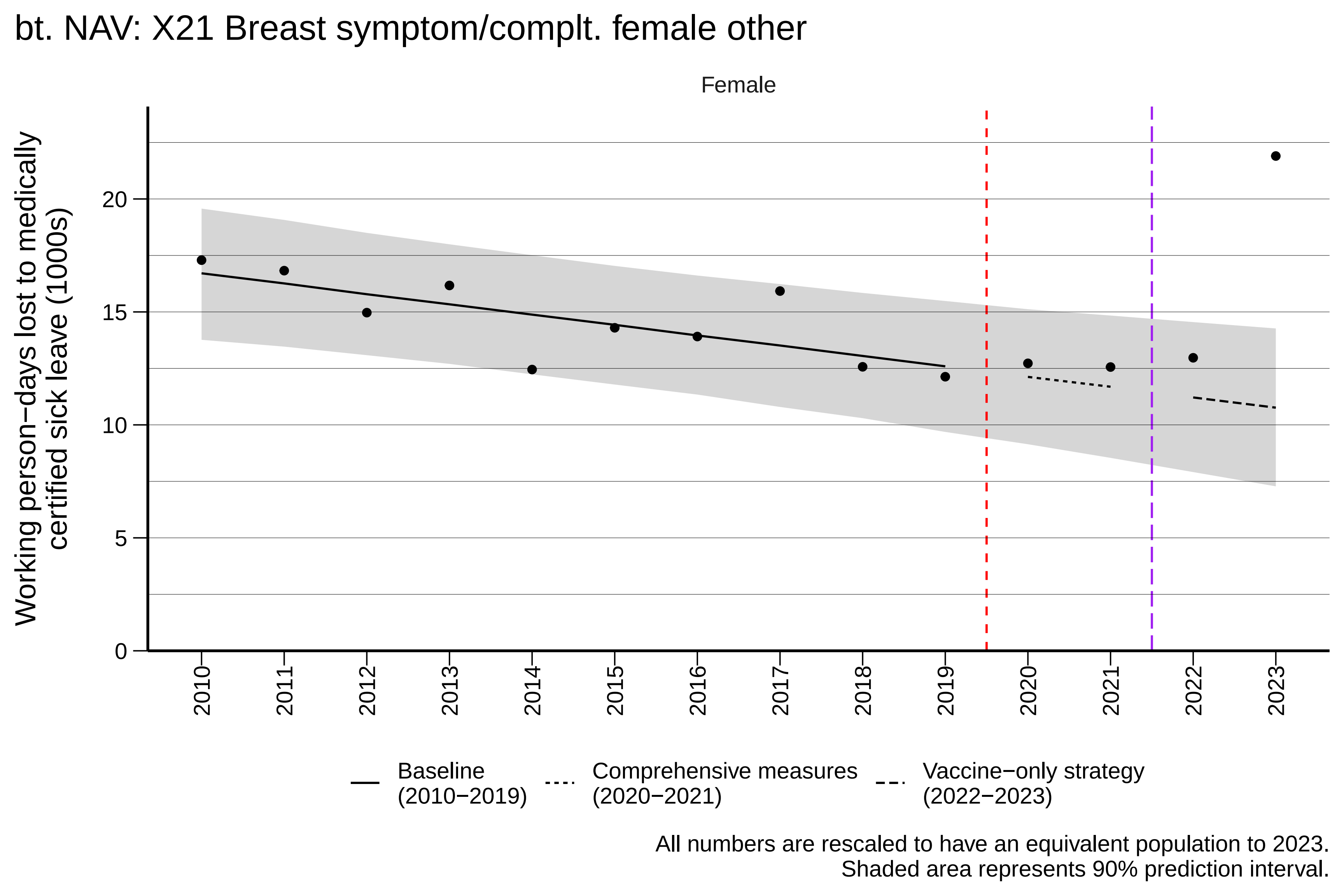

bt. NAV: X21 Breast symptom/complt. female other
Female
Working person−days lost to medically certified sick leave (1000s)
0
5
10
15
20
2010
2011
2012
2013
2014
2015
2016
2017
2018
2019
2020
2021
2022
2023
Baseline (2010−2019)
Comprehensive measures (2020−2021)
Vaccine−only strategy (2022−2023)
All numbers are rescaled to have an equivalent population to 2023.
Shaded area represents 90% prediction interval.

bu. NorSySS: A03 Fever

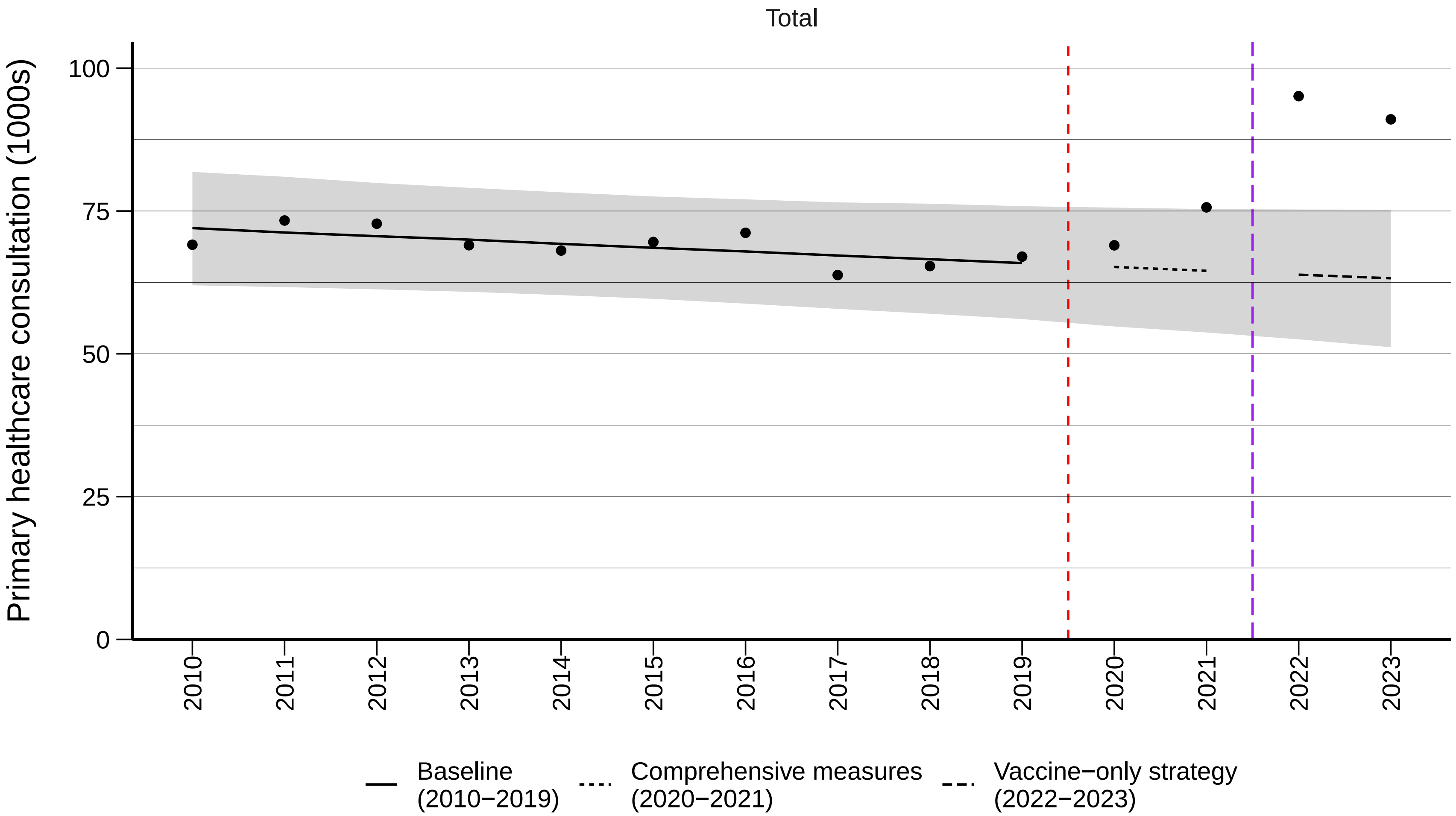

All numbers are rescaled to have an equivalent population to 2023.
Shaded area represents 90% prediction interval.

bv. NorSySS: A03 Fever

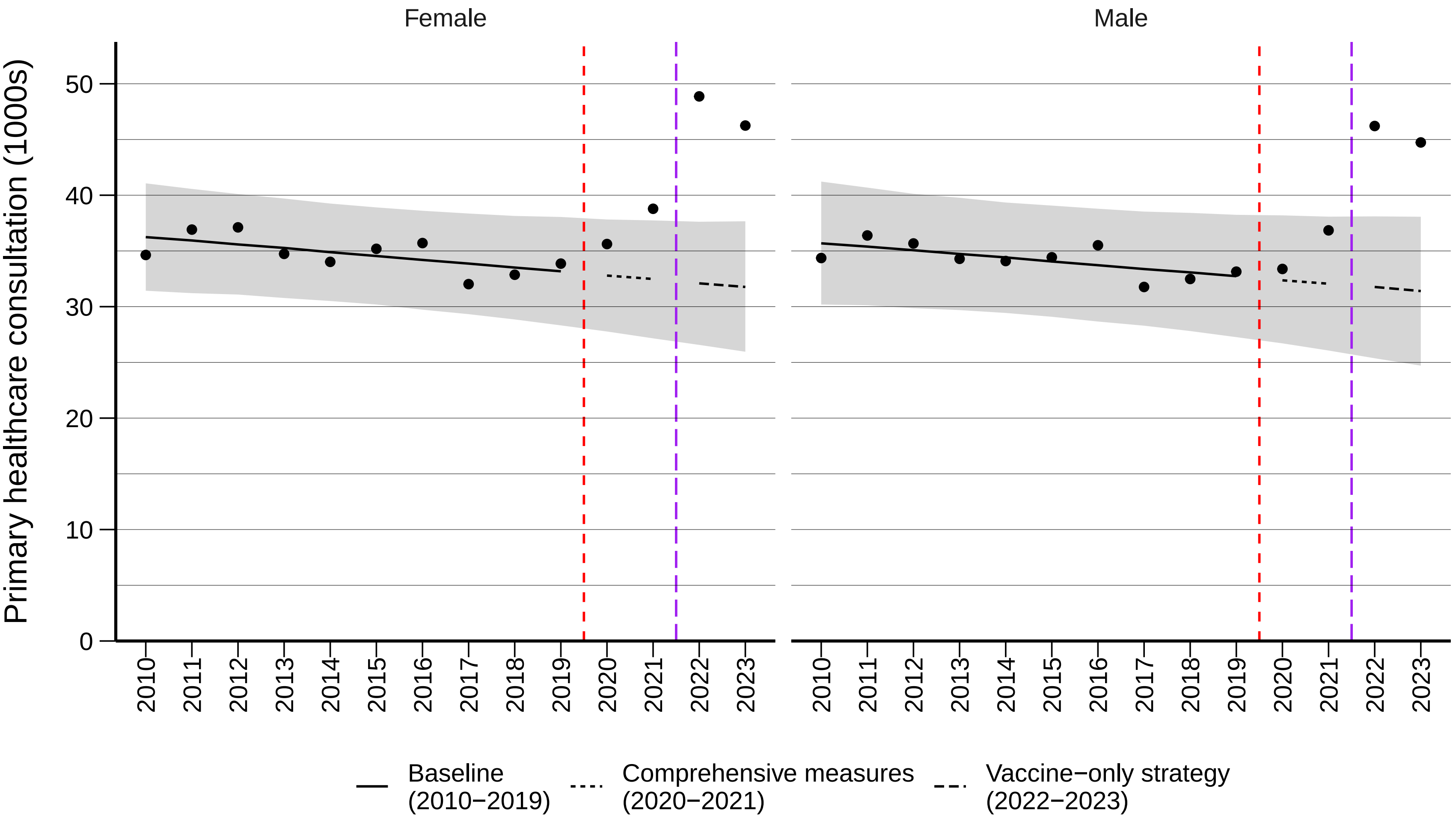

All numbers are rescaled to have an equivalent population to 2023.
Shaded area represents 90% prediction interval.

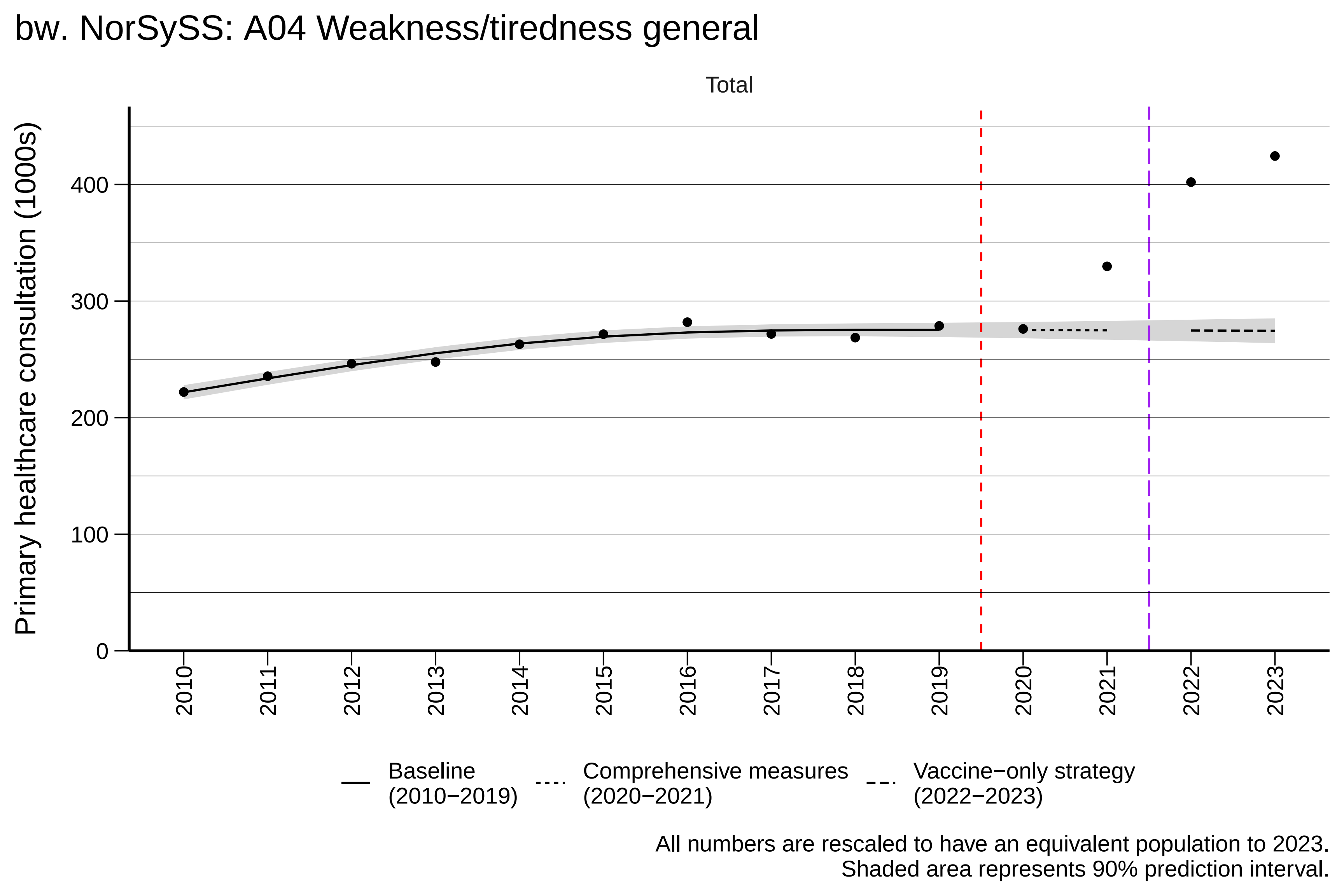

bw. NorSySS: A04 Weakness/tiredness general
Total
Primary healthcare consultation (1000s)
0
100
200
300
400
2010
2011
2012
2013
2014
2015
2016
2017
2018
2019
2020
2021
2022
2023
Baseline (2010−2019)
Comprehensive measures (2020−2021)
Vaccine−only strategy (2022−2023)
All numbers are rescaled to have an equivalent population to 2023.
Shaded area represents 90% prediction interval.

bx. NorSySS: A04 Weakness/tiredness general

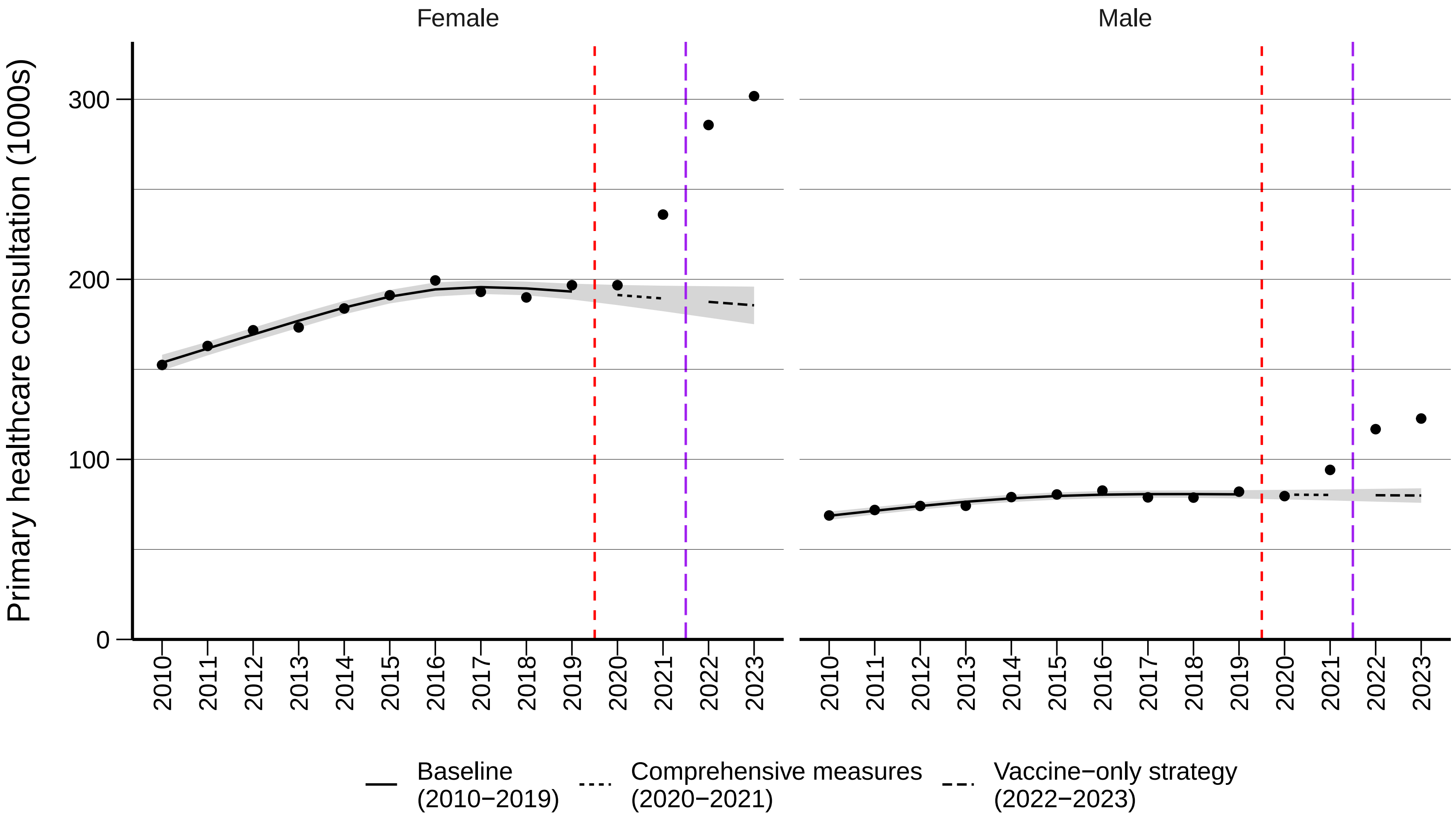

All numbers are rescaled to have an equivalent population to 2023.
Shaded area represents 90% prediction interval.

by. NorSySS: A05 Feeling ill

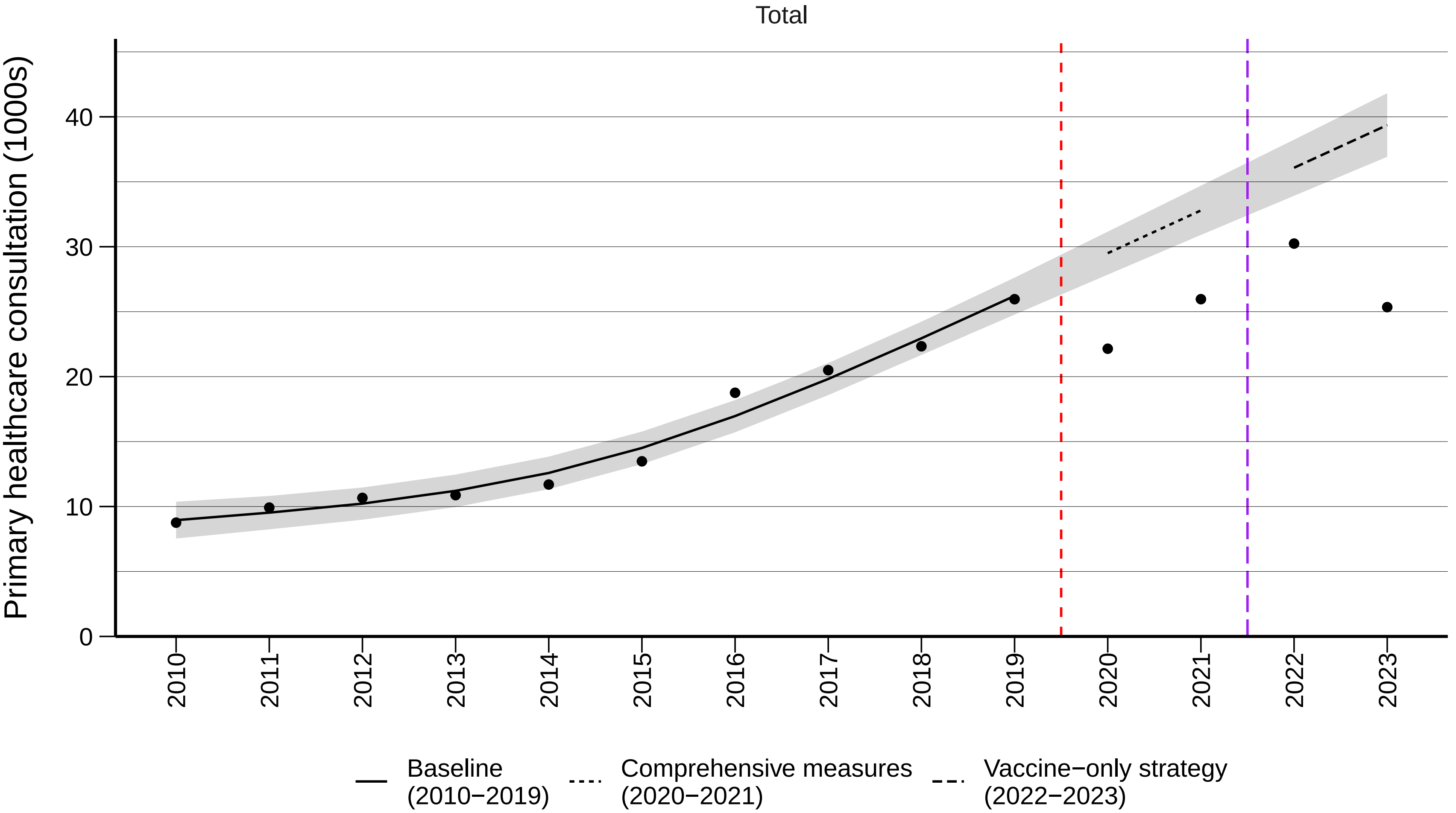

All numbers are rescaled to have an equivalent population to 2023.
Shaded area represents 90% prediction interval.

bz. NorSySS: A05 Feeling ill

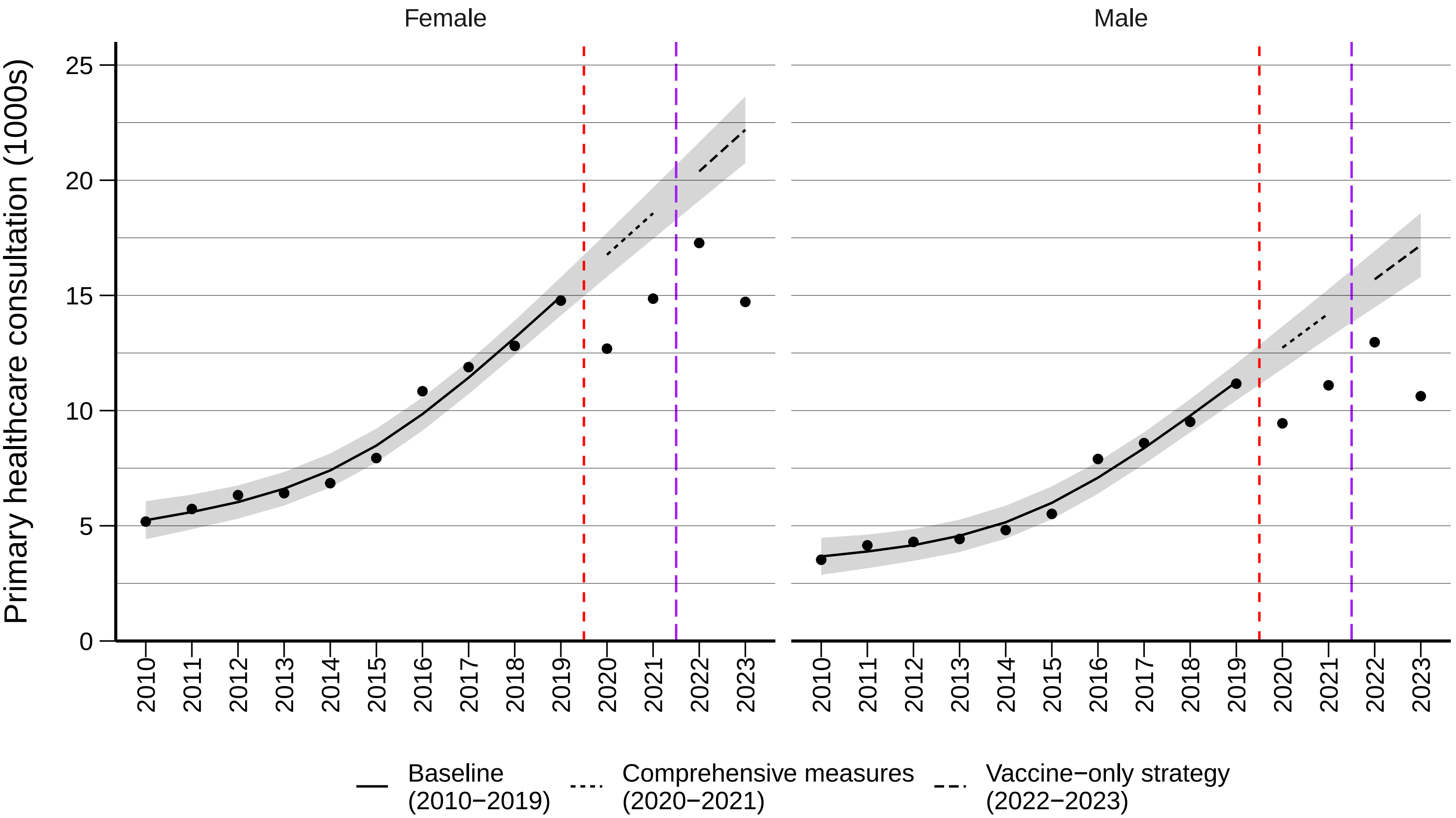

All numbers are rescaled to have an equivalent population to 2023.
Shaded area represents 90% prediction interval.

ca. NorSySS: A78 Infectious disease other/NOS

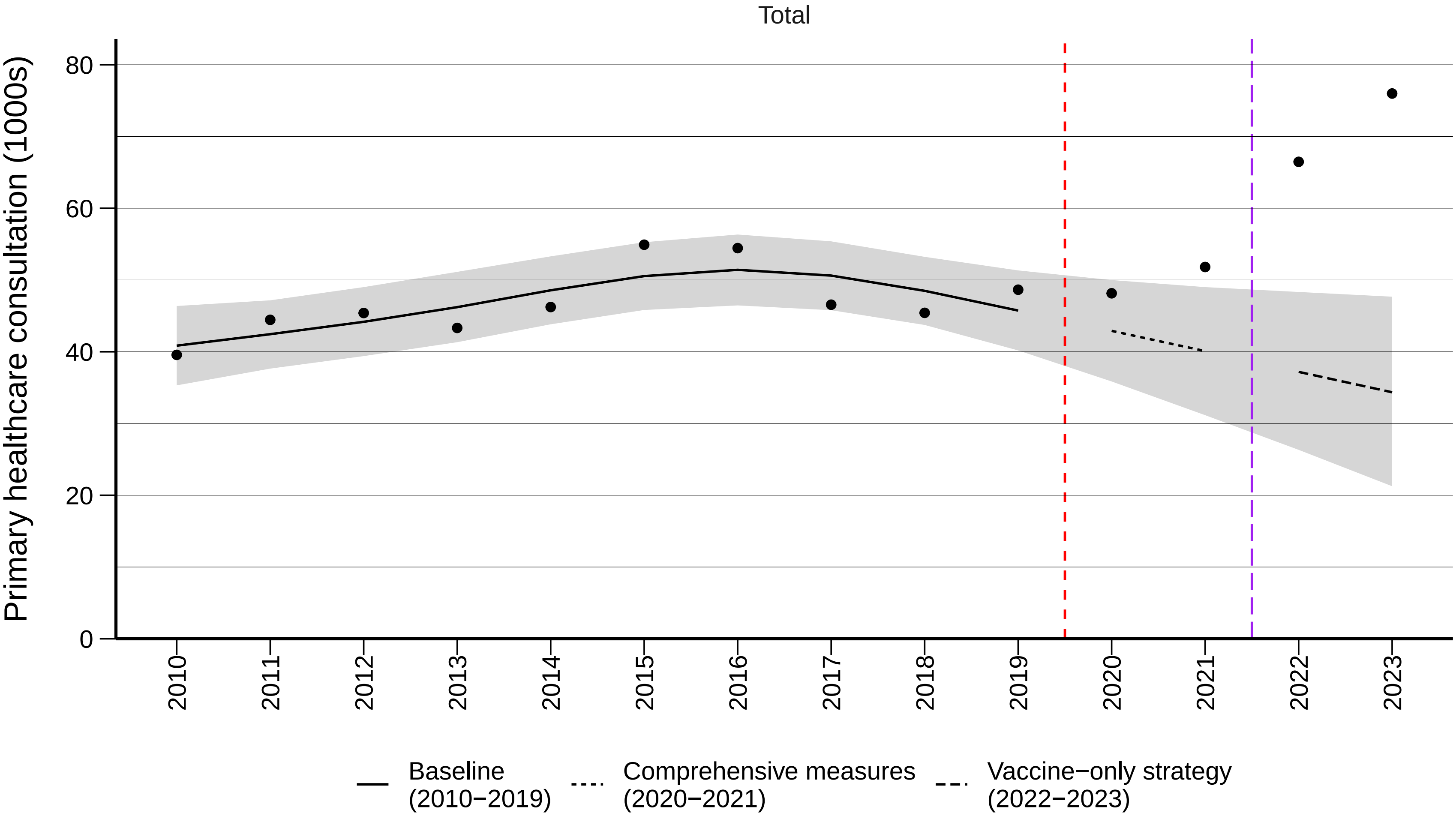

All numbers are rescaled to have an equivalent population to 2023.
Shaded area represents 90% prediction interval.

cb. NorSySS: A78 Infectious disease other/NOS

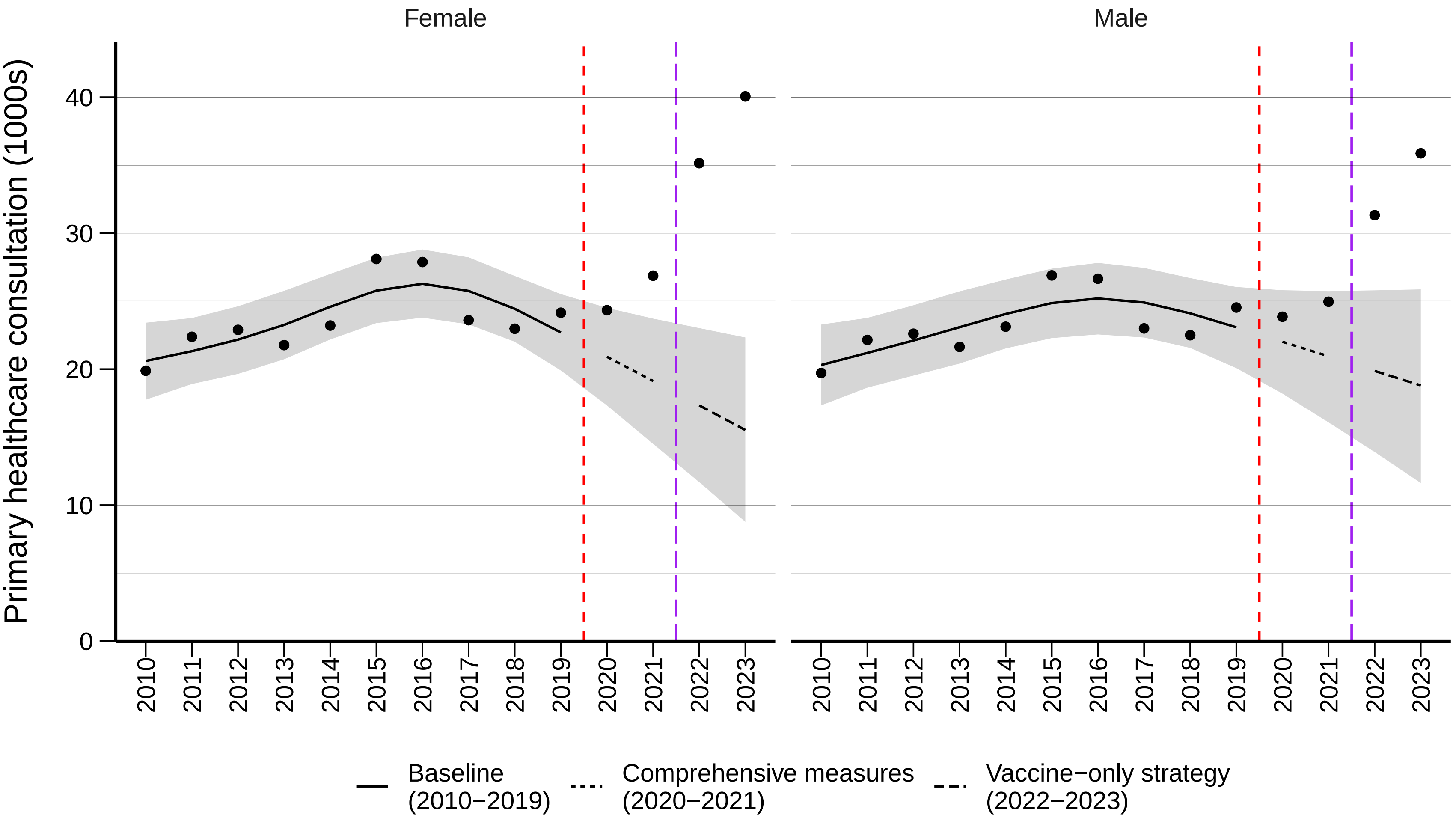

All numbers are rescaled to have an equivalent population to 2023.
Shaded area represents 90% prediction interval.

cc. NorSySS: D01 Abdominal pain/cramps general

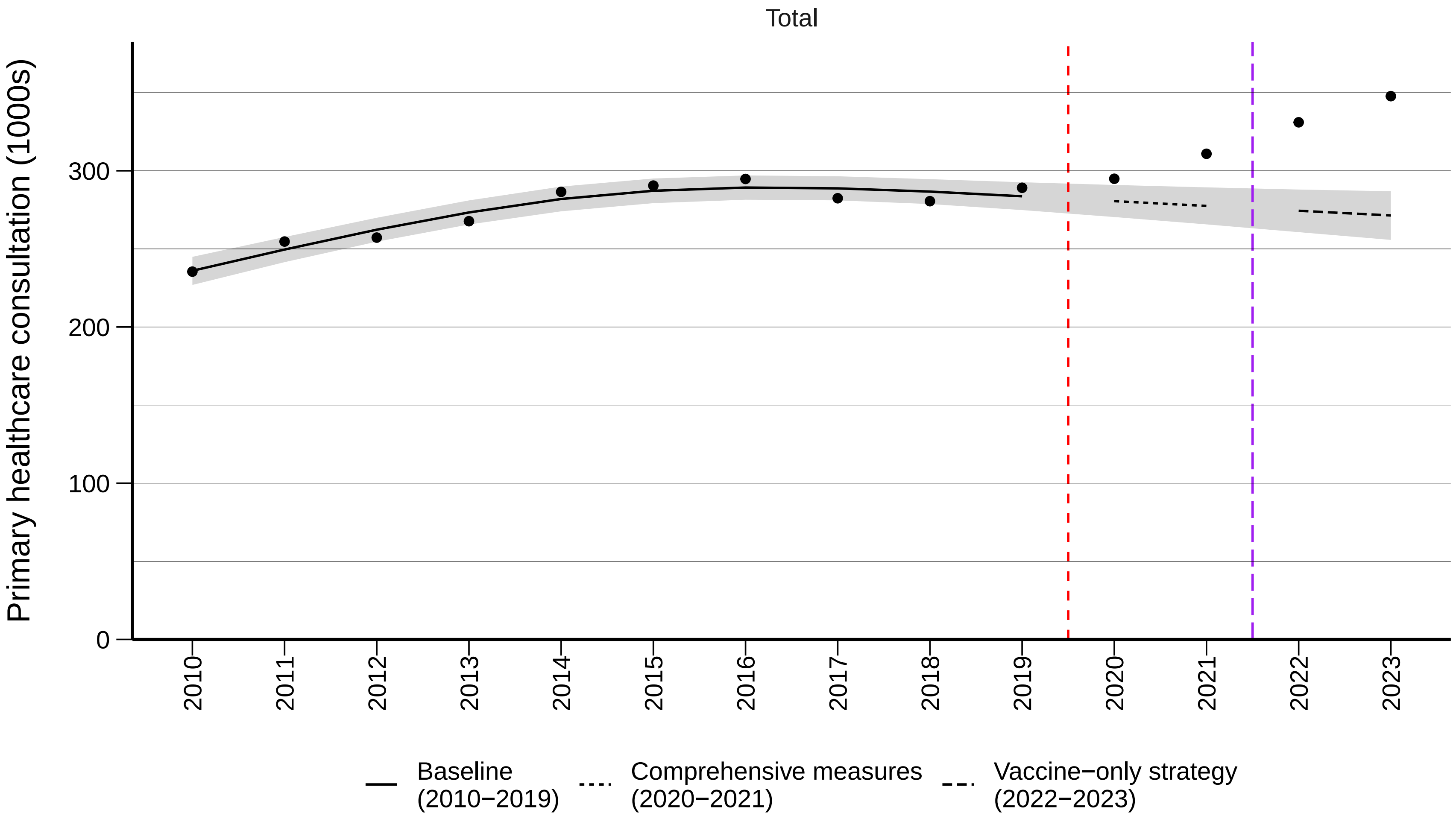

All numbers are rescaled to have an equivalent population to 2023.
Shaded area represents 90% prediction interval.

cd. NorSySS: D01 Abdominal pain/cramps general

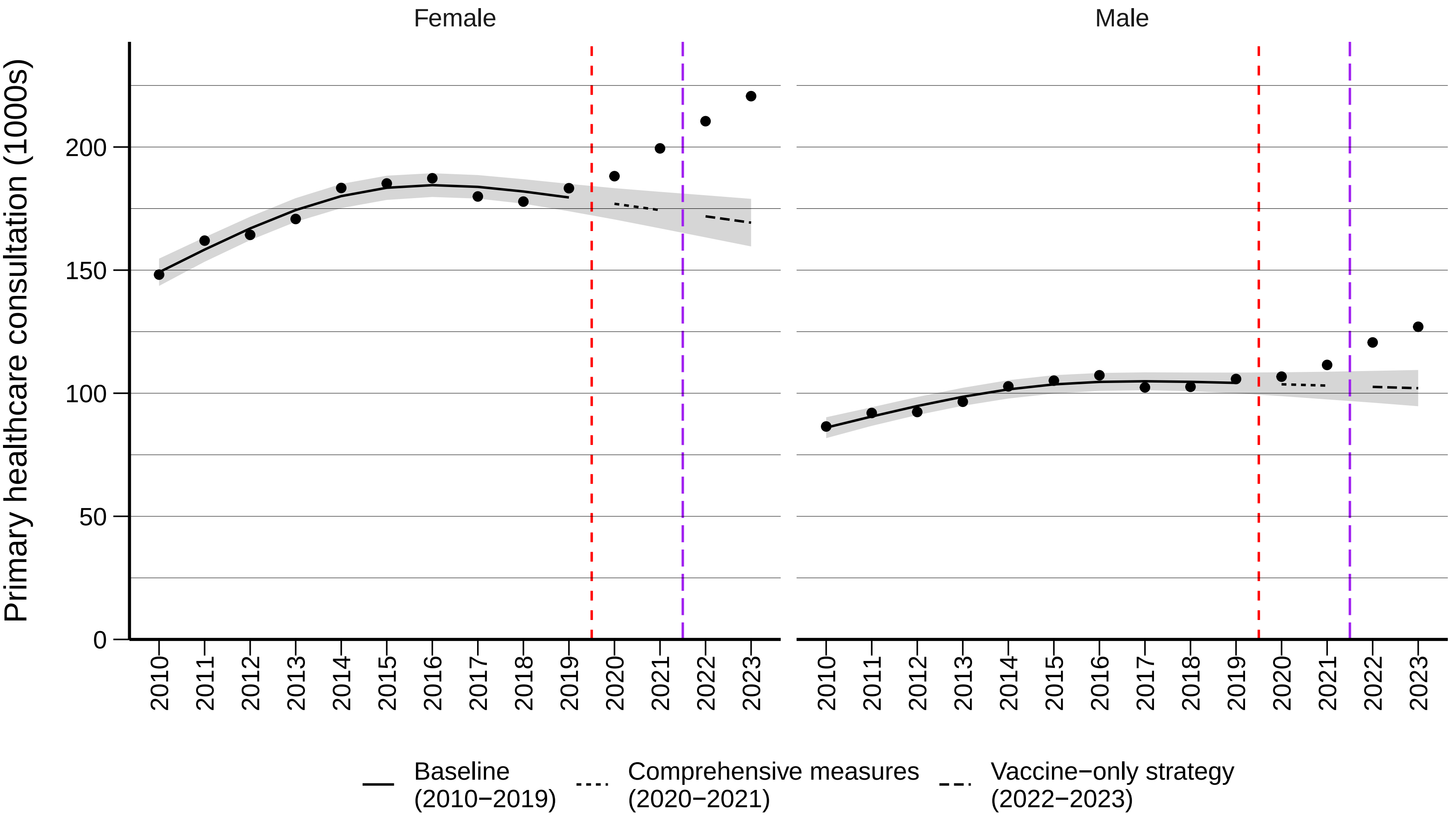

All numbers are rescaled to have an equivalent population to 2023.
Shaded area represents 90% prediction interval.

ce. NorSySS: D09 Nausea

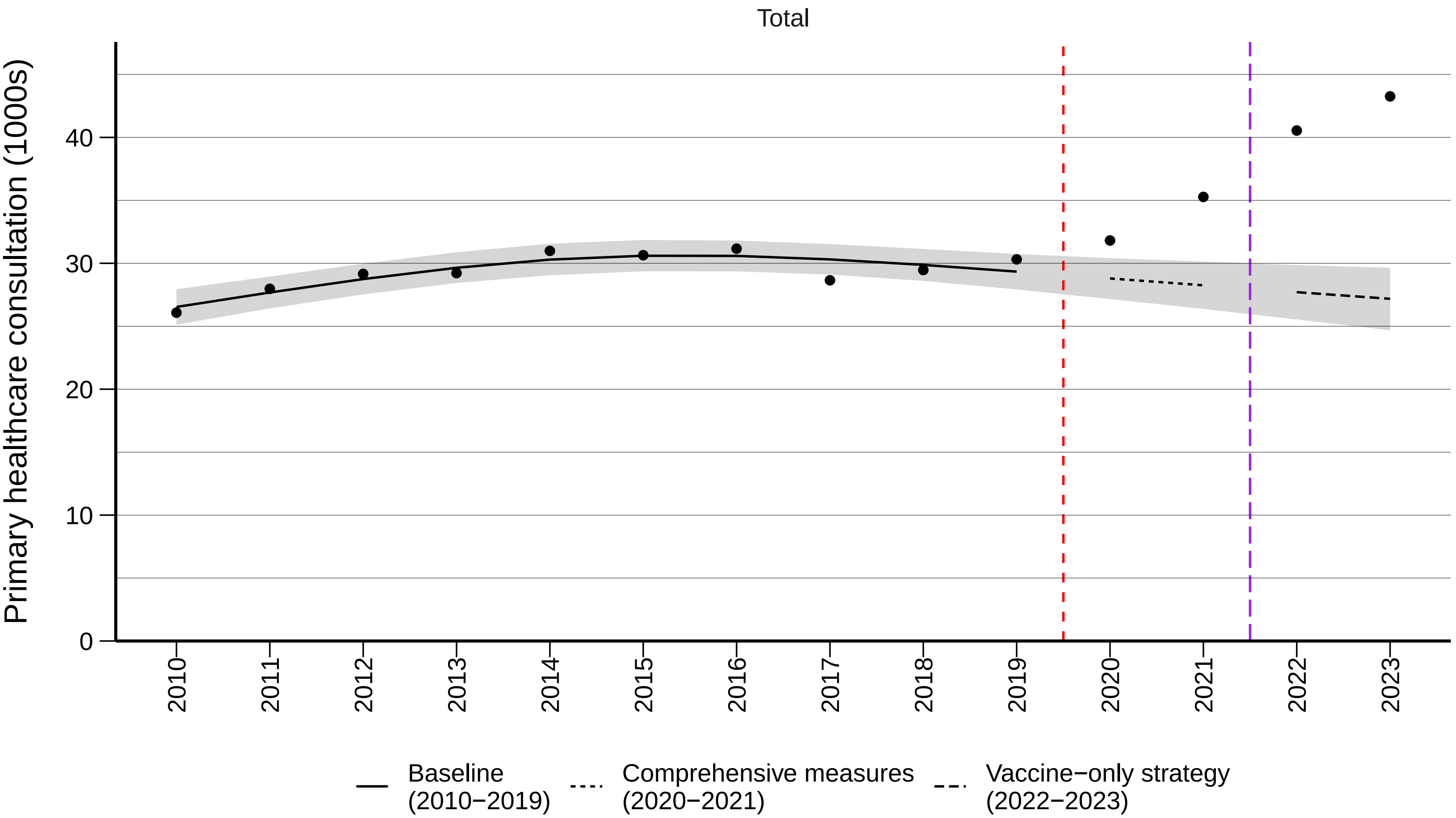

All numbers are rescaled to have an equivalent population to 2023.
Shaded area represents 90% prediction interval.

cf. NorSySS: D09 Nausea

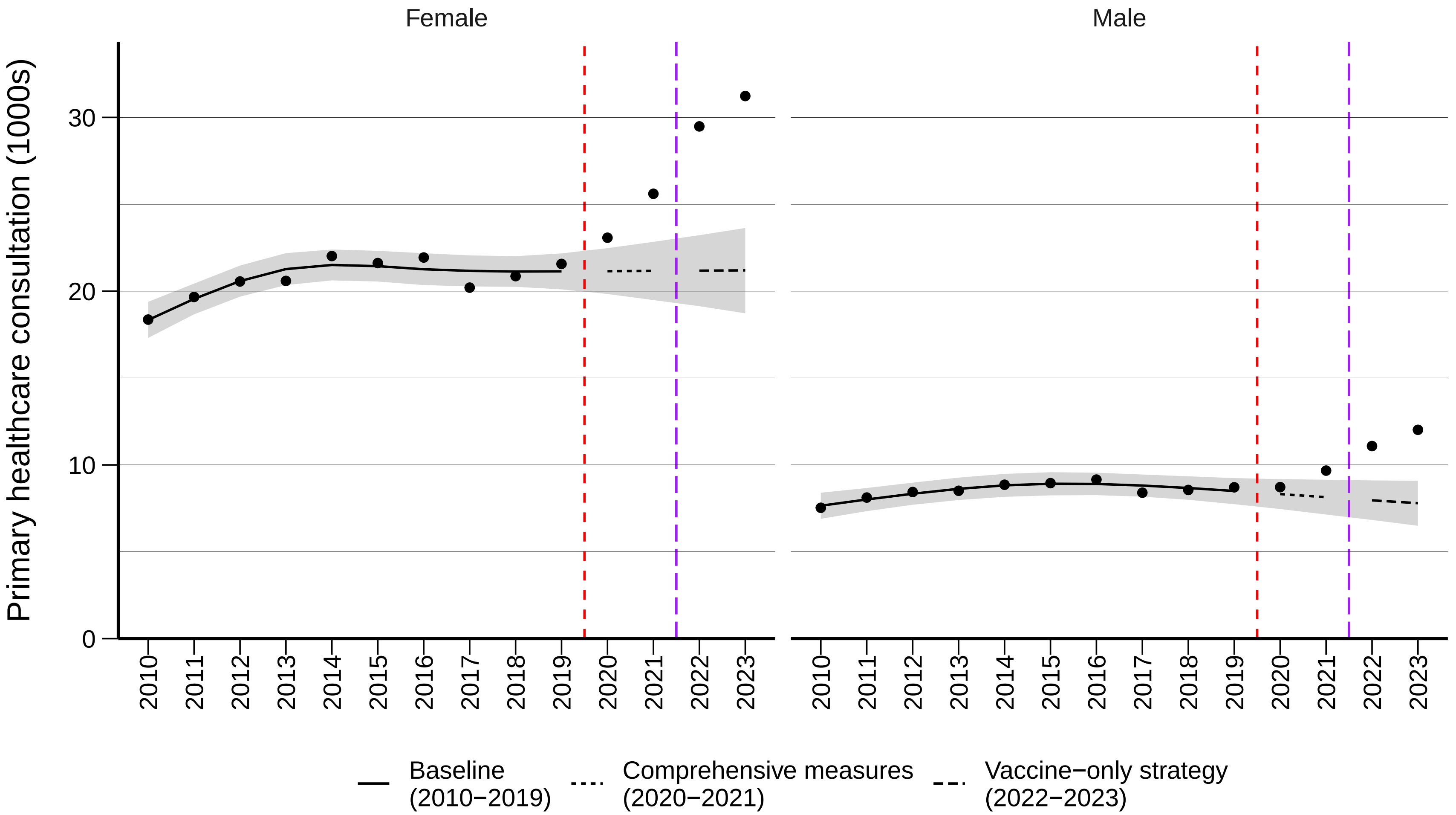

All numbers are rescaled to have an equivalent population to 2023.
Shaded area represents 90% prediction interval.

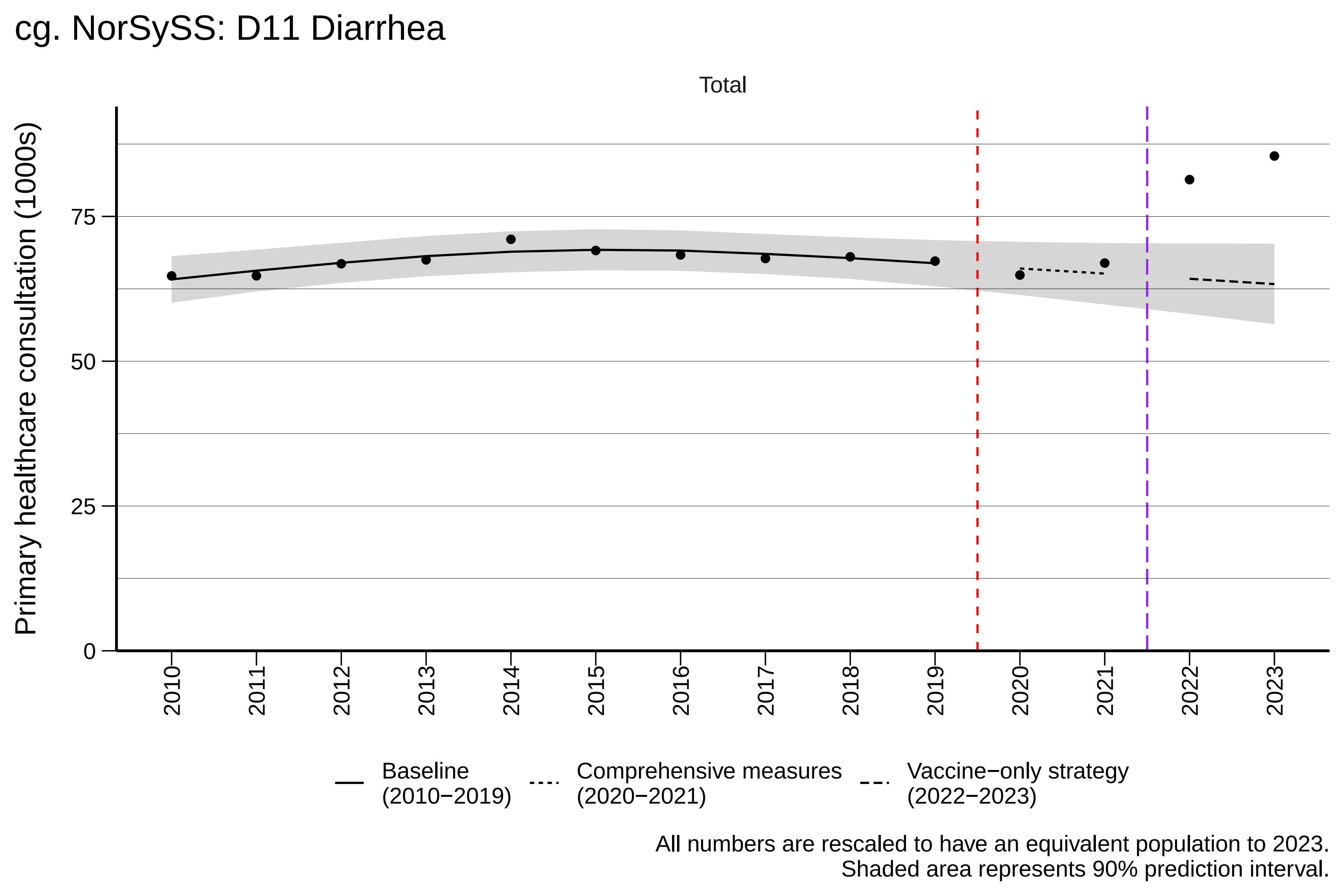
cg. NorSySS: D11 Diarrhea
Total
Primary healthcare consultation (1000s)
0
25
50
75
2010
2011
2012
2013
2014
2015
2016
2017
2018
2019
2020
2021
2022
2023
Baseline (2010−2019)
Comprehensive measures (2020−2021)
Vaccine−only strategy (2022−2023)
All numbers are rescaled to have an equivalent population to 2023.
Shaded area represents 90% prediction interval.

ch. NorSySS: D11 Diarrhea

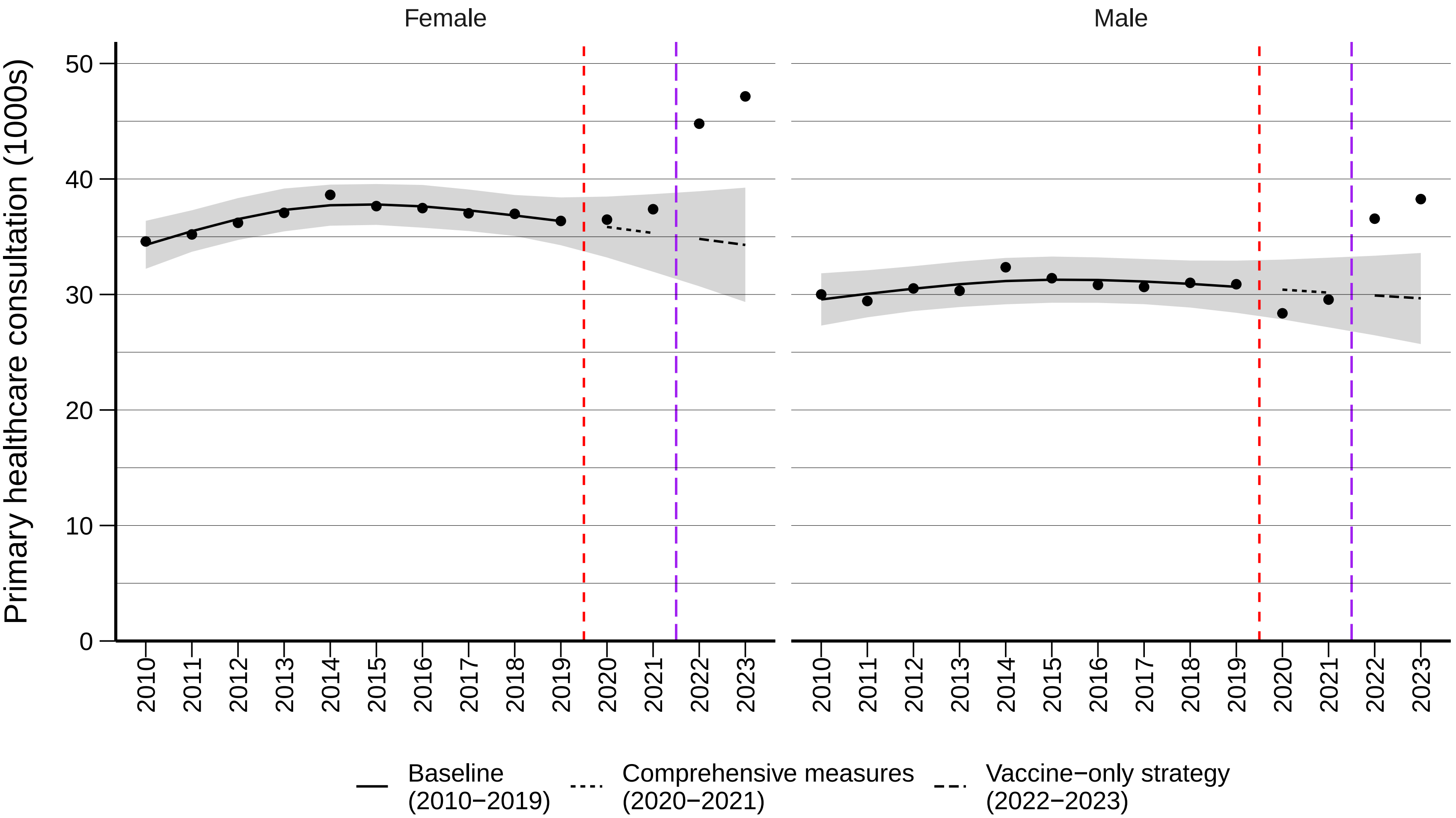

All numbers are rescaled to have an equivalent population to 2023.
Shaded area represents 90% prediction interval.

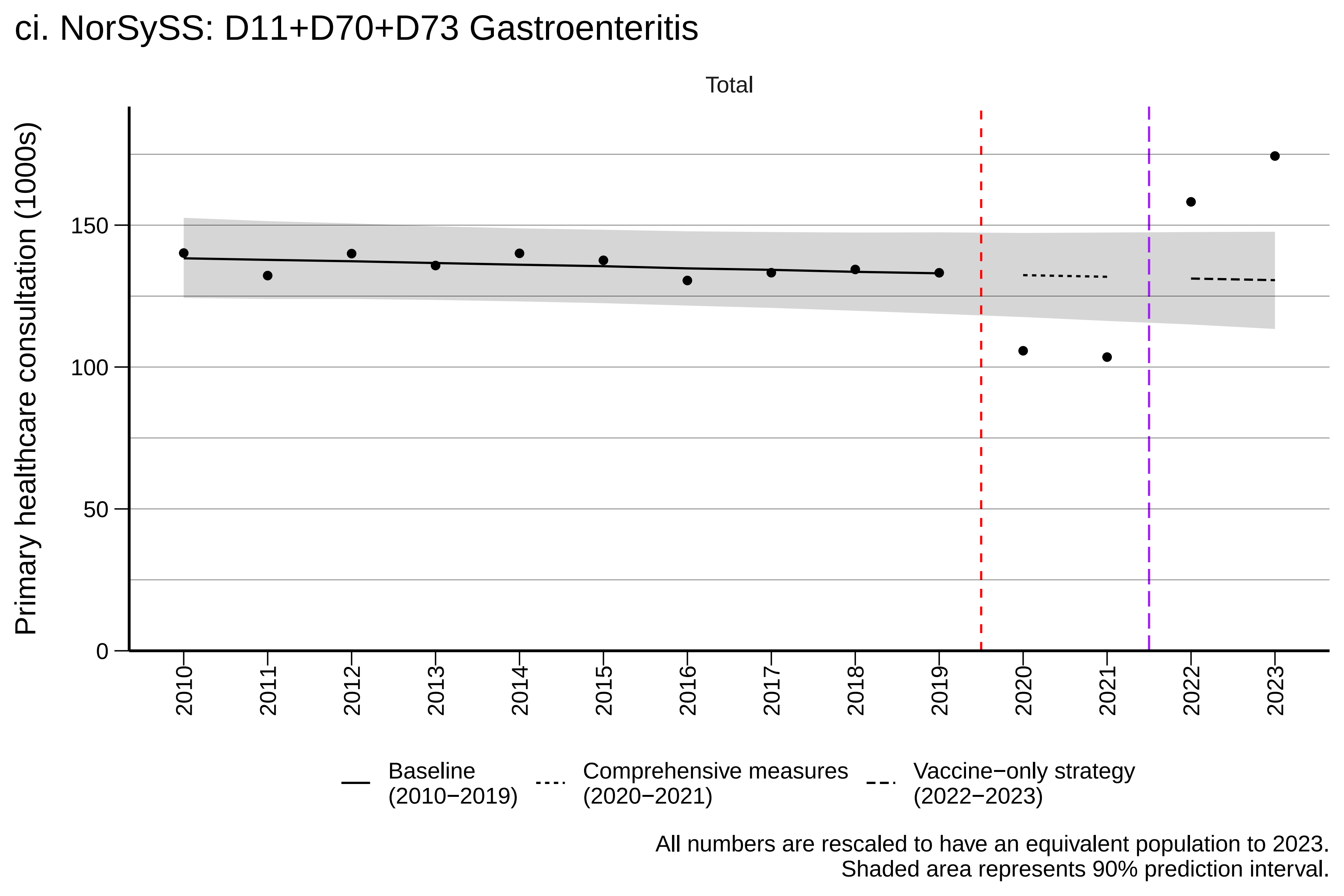
ci. NorSySS: D11+D70+D73 Gastroenteritis
Total
Primary healthcare consultation (1000s)
0
50
100
150
2010
2011
2012
2013
2014
2015
2016
2017
2018
2019
2020
2021
2022
2023
Baseline
(2010−2019)
Comprehensive measures
(2020−2021)
Vaccine−only strategy
(2022−2023)
All numbers are rescaled to have an equivalent population to 2023.
Shaded area represents 90% prediction interval.

cj. NorSySS: D11+D70+D73 Gastroenteritis

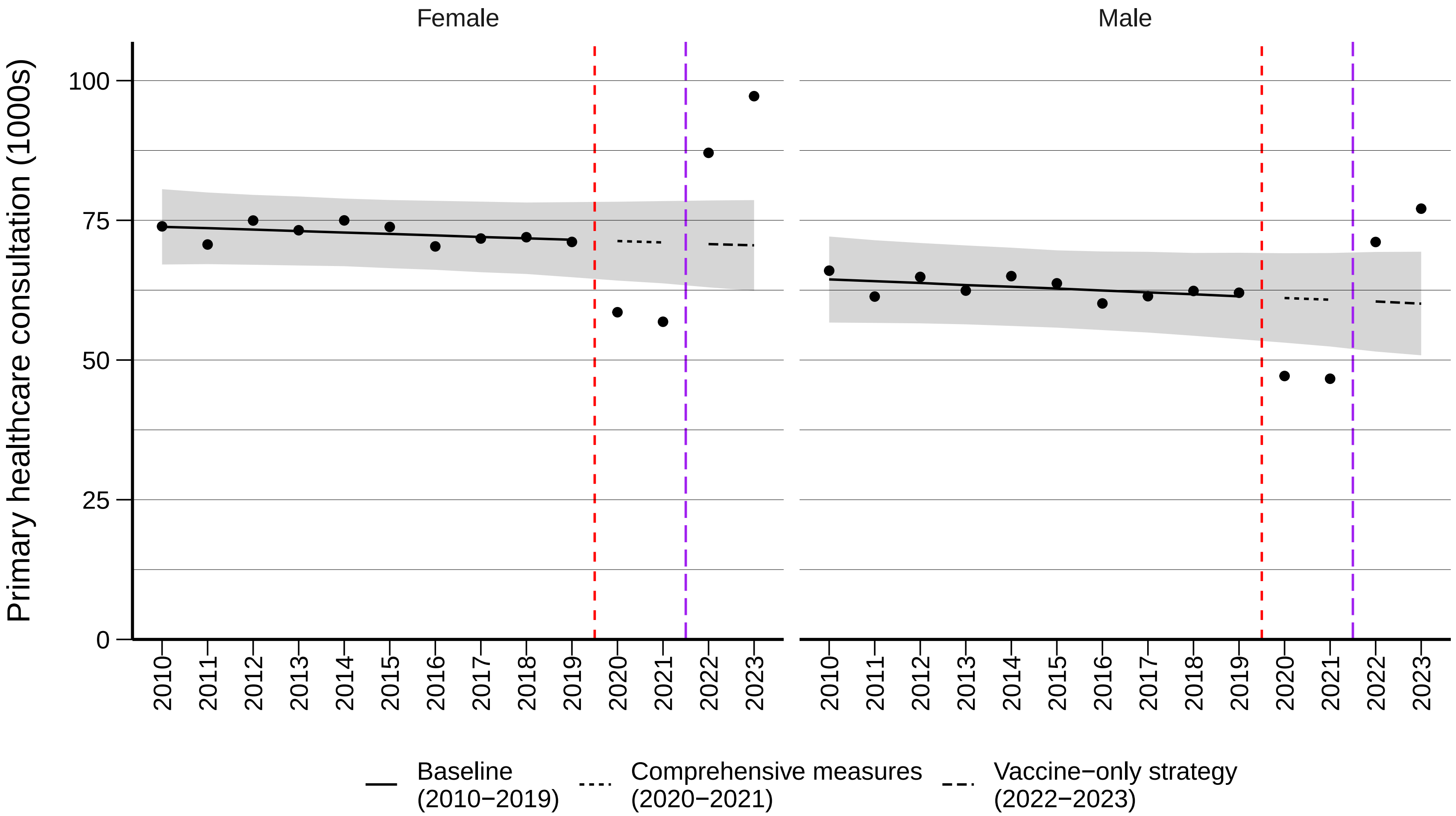

All numbers are rescaled to have an equivalent population to 2023.
Shaded area represents 90% prediction interval.

ck. NorSySS: D18 Change feces/bowel movements

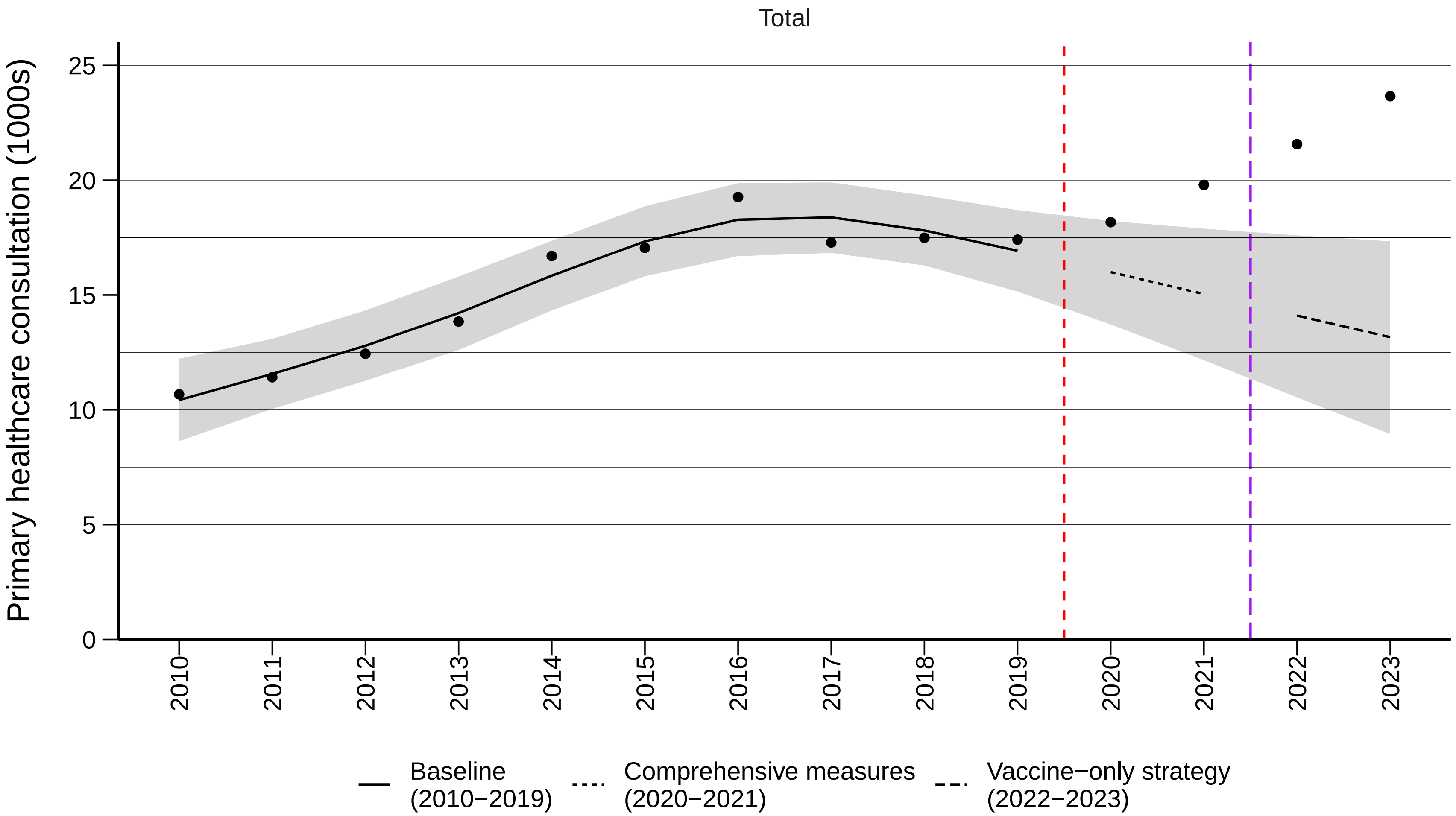

All numbers are rescaled to have an equivalent population to 2023.
Shaded area represents 90% prediction interval.

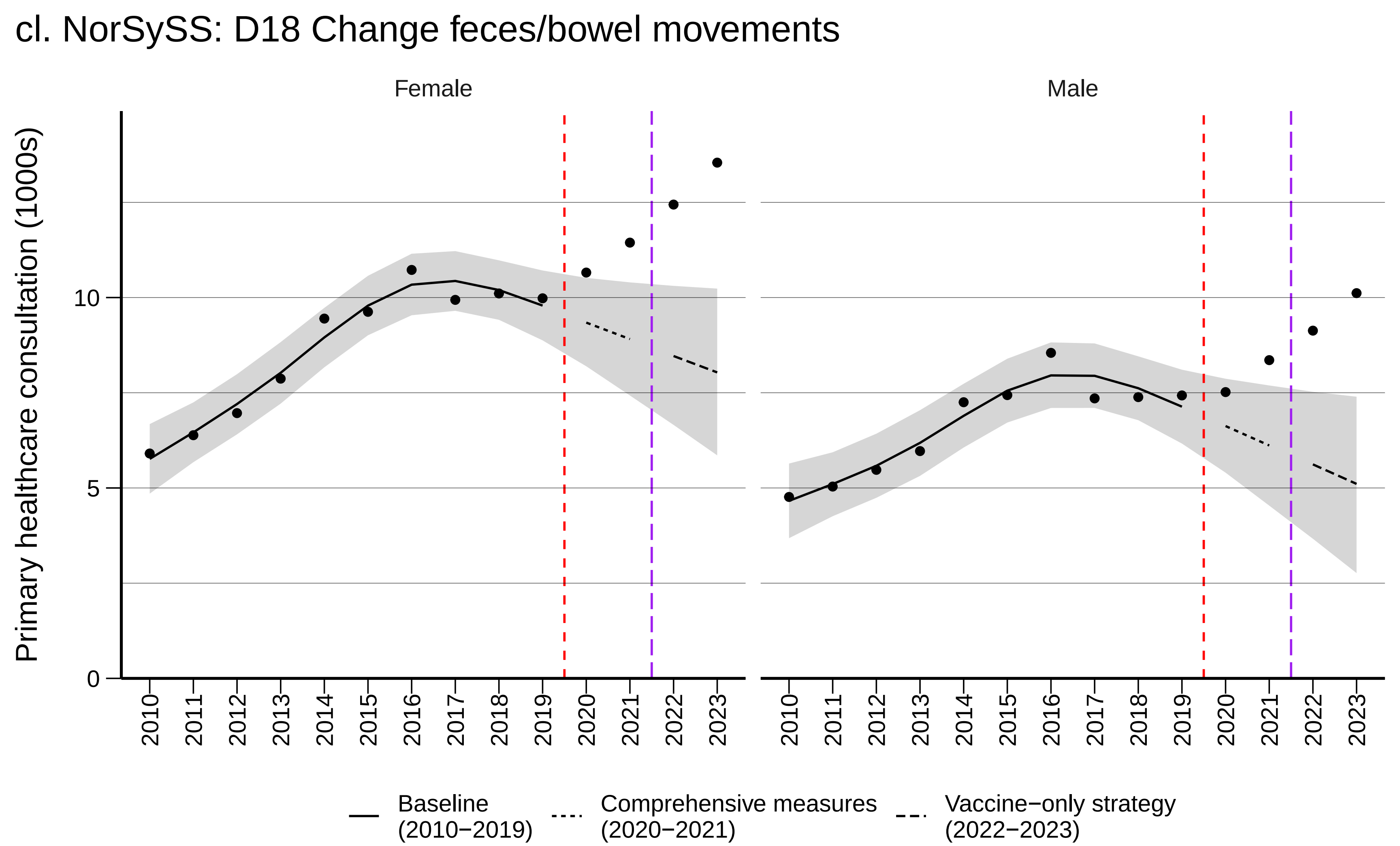

All numbers are rescaled to have an equivalent population to 2023.
Shaded area represents 90% prediction interval.

cm. NorSySS: D73 Gastroenteritis presumed infection

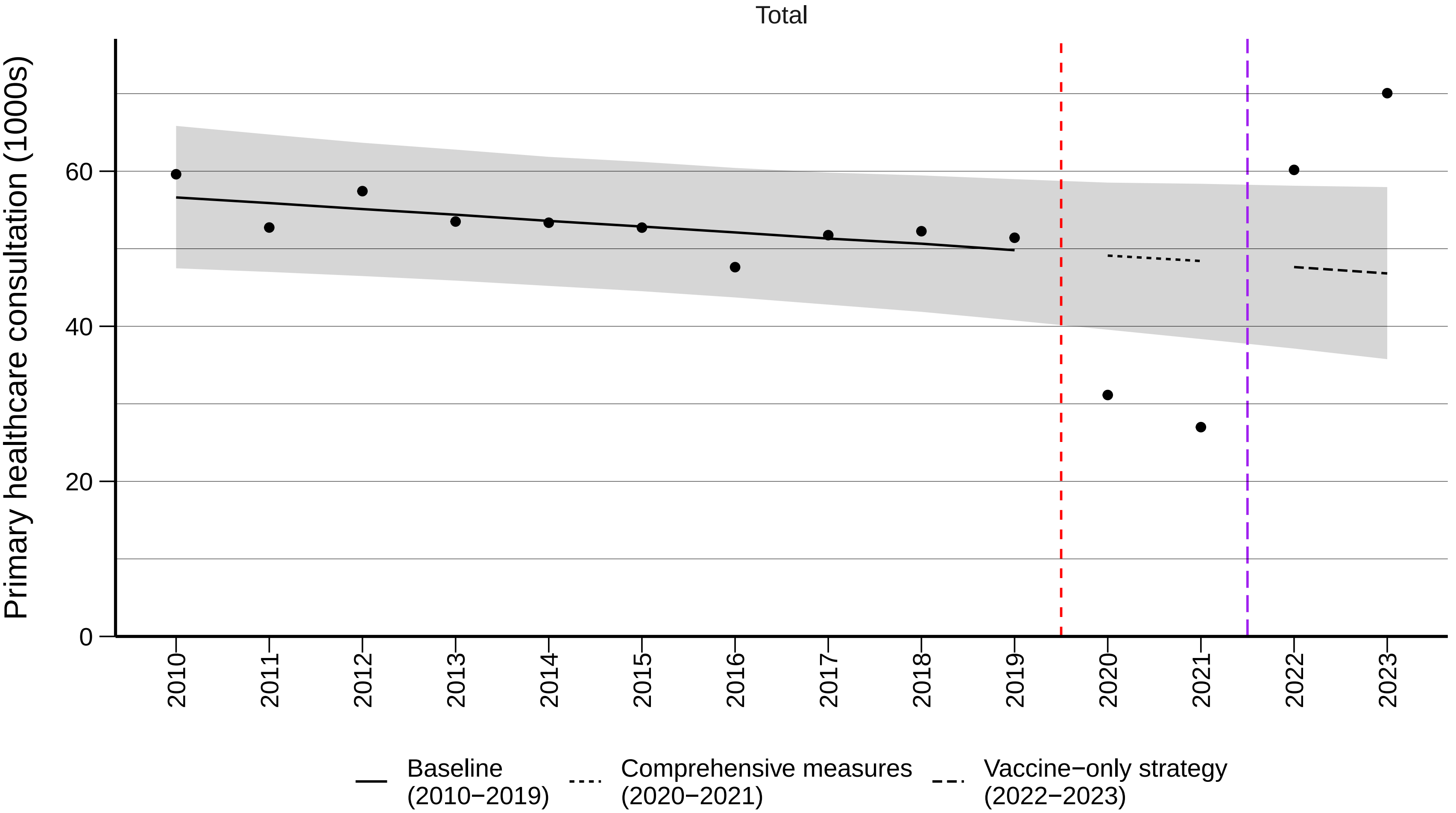

All numbers are rescaled to have an equivalent population to 2023.
Shaded area represents 90% prediction interval.

cn. NorSySS: D73 Gastroenteritis presumed infection

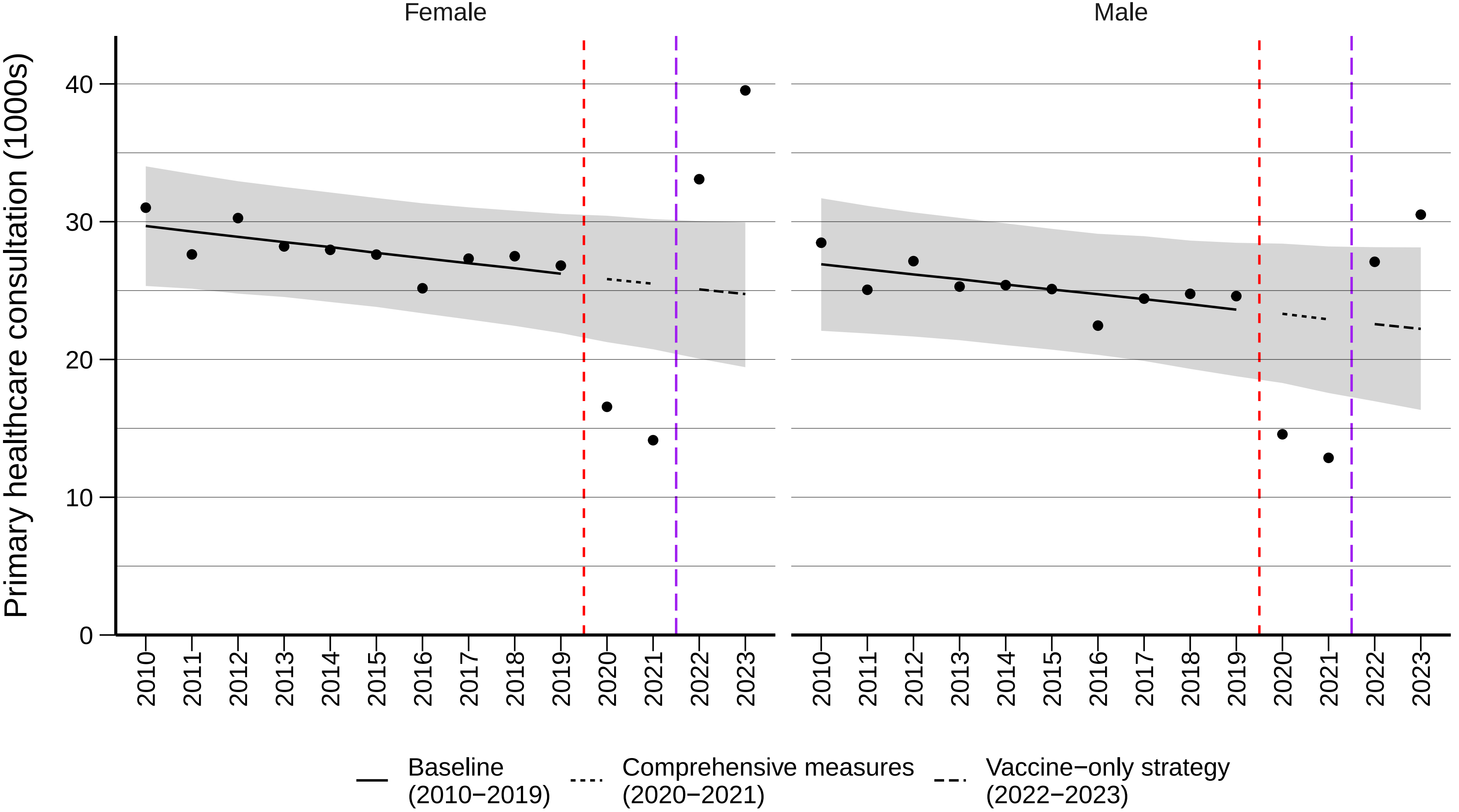

All numbers are rescaled to have an equivalent population to 2023.
Shaded area represents 90% prediction interval.

co. NorSySS: D99 Disease digestive system, other

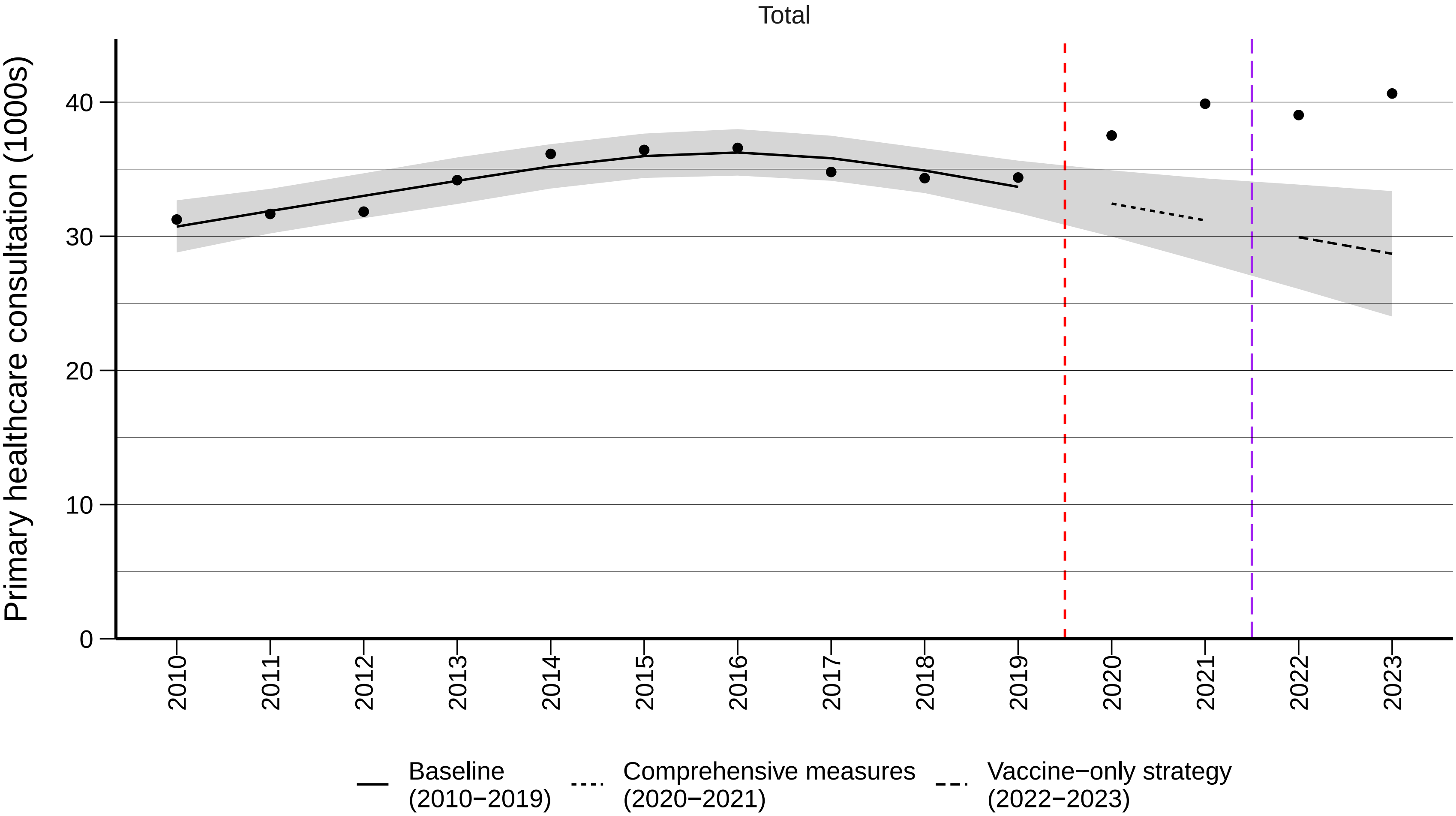

All numbers are rescaled to have an equivalent population to 2023.
Shaded area represents 90% prediction interval.

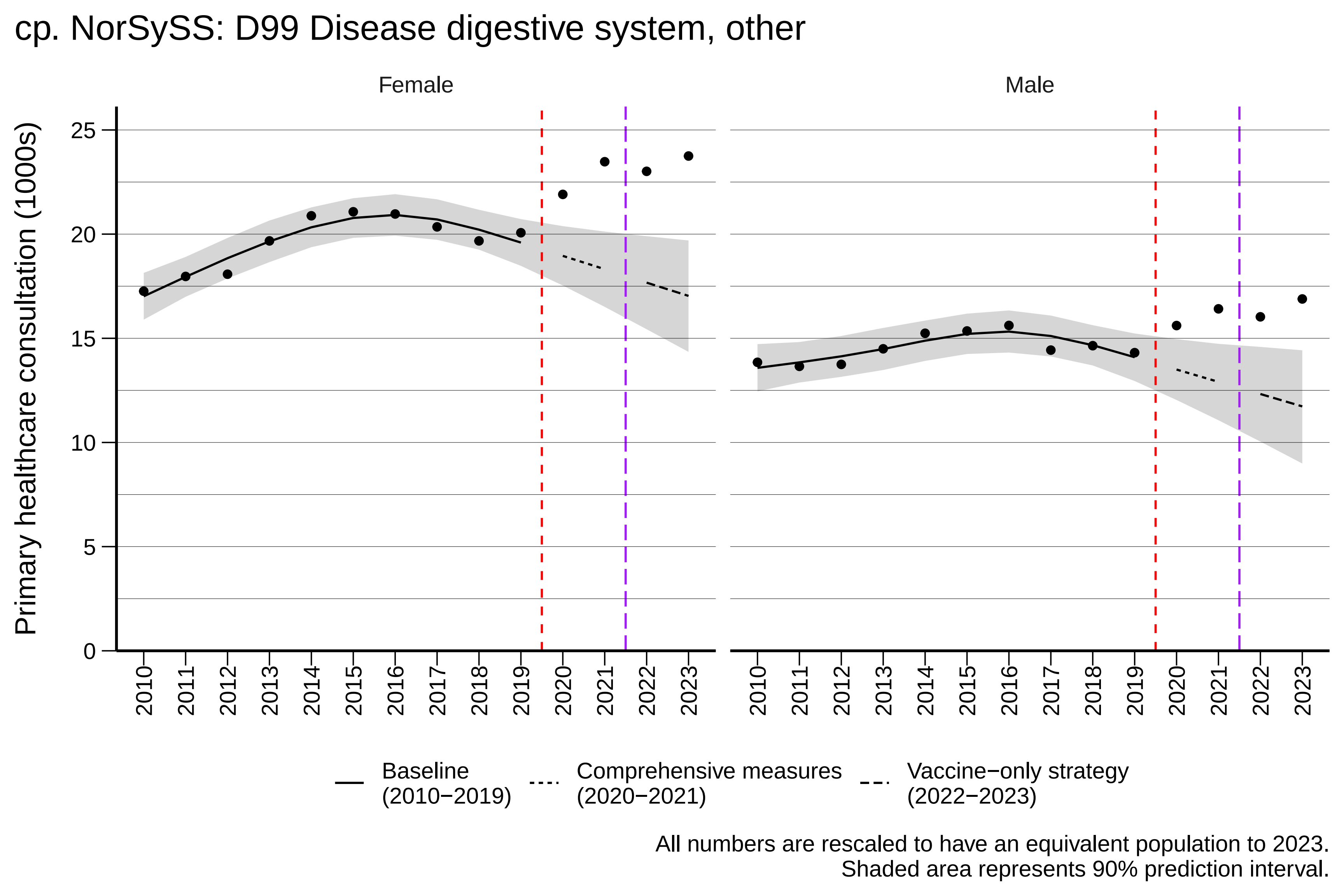

cp. NorSySS: D99 Disease digestive system, other
Female
Male
Primary healthcare consultation (1000s)
0
5
10
15
20
25
2010
2011
2012
2013
2014
2015
2016
2017
2018
2019
2020
2021
2022
2023
Baseline (2010−2019)
Comprehensive measures (2020−2021)
Vaccine−only strategy (2022−2023)
All numbers are rescaled to have an equivalent population to 2023.
Shaded area represents 90% prediction interval.

cq. NorSySS: H01 Ear pain/earache

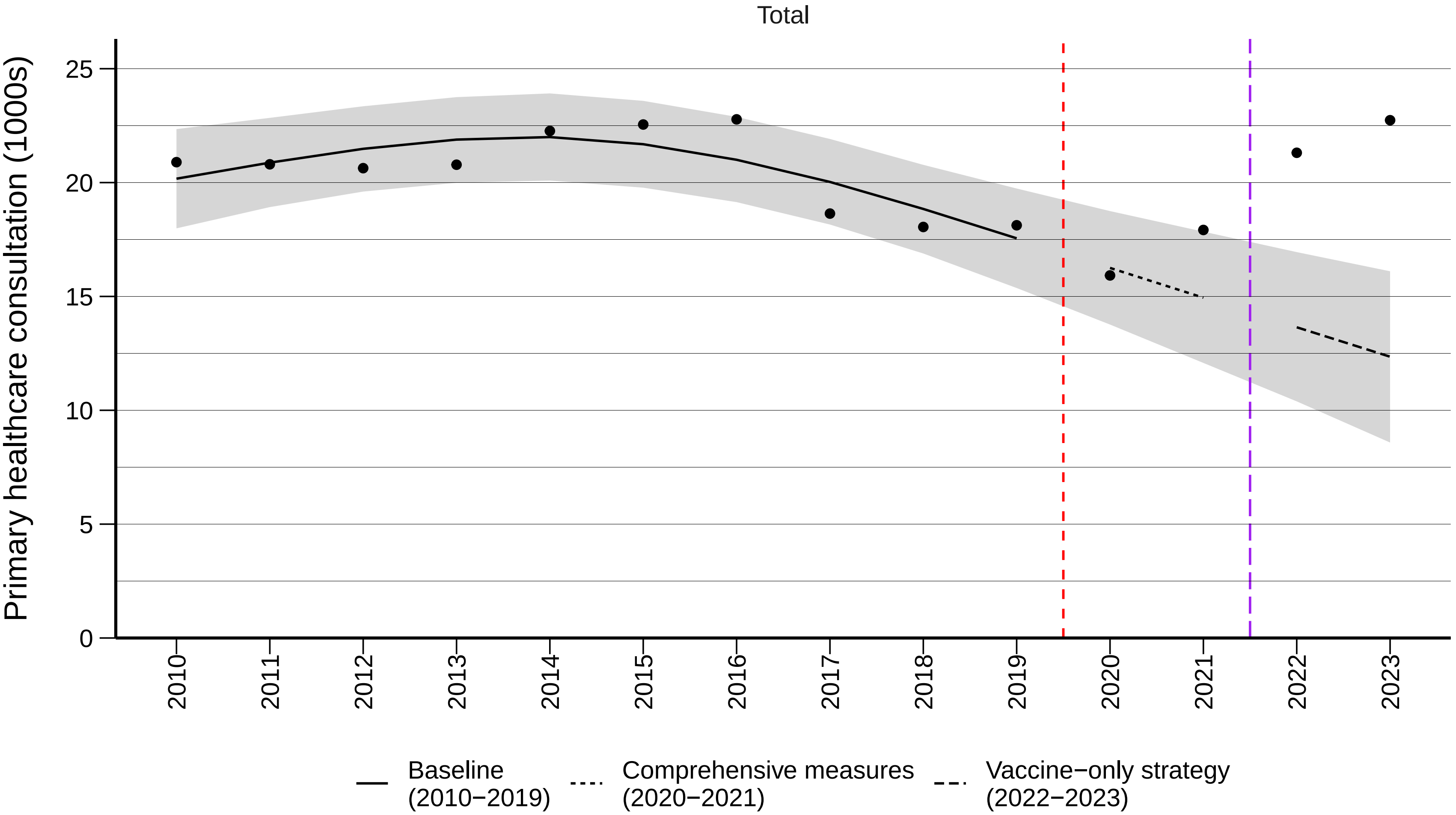

All numbers are rescaled to have an equivalent population to 2023.
Shaded area represents 90% prediction interval.

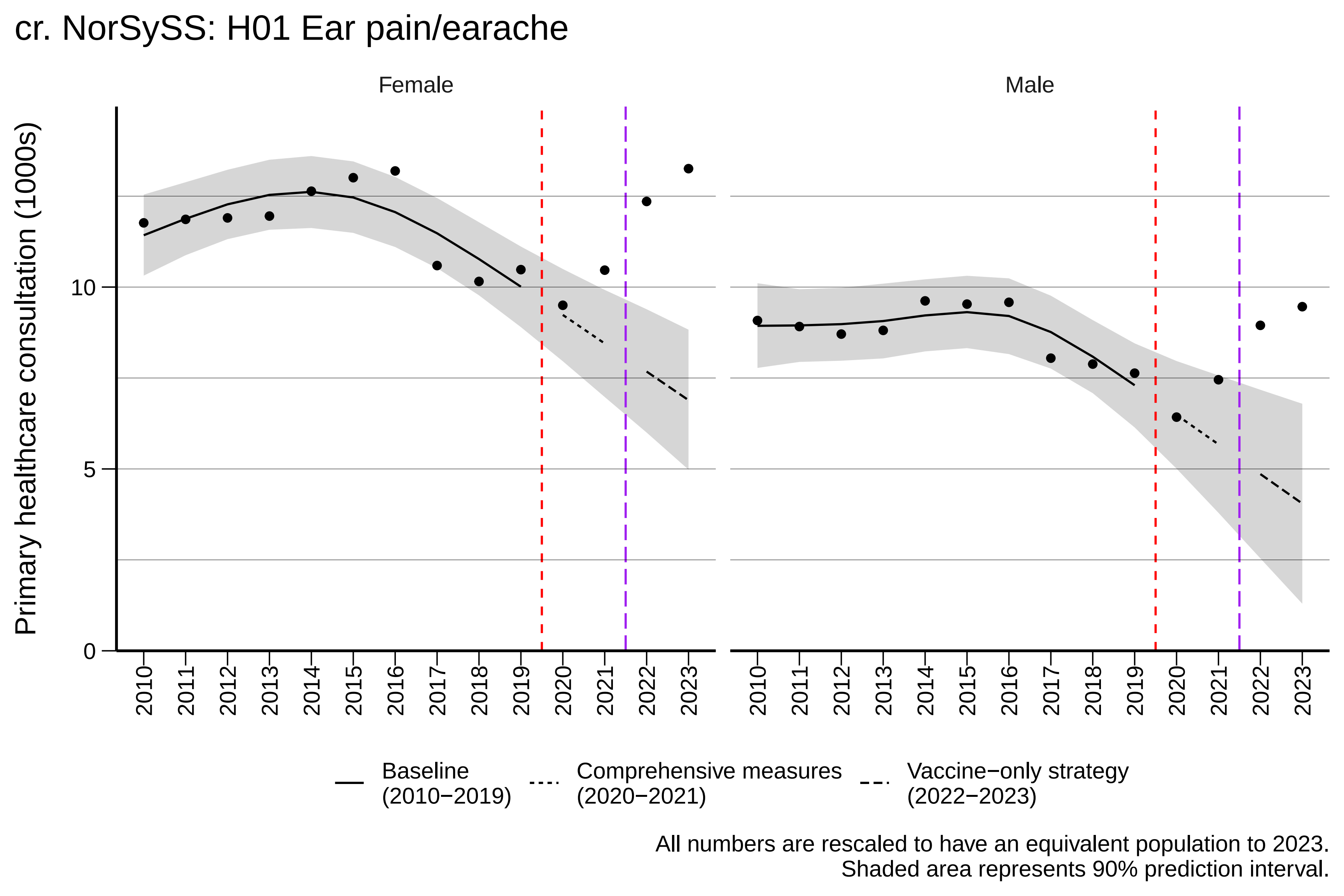

cr. NorSySS: H01 Ear pain/earache
Female
Male
Primary healthcare consultation (1000s)
0
5
10
2010
2011
2012
2013
2014
2015
2016
2017
2018
2019
2020
2021
2022
2023
Baseline (2010−2019)
Comprehensive measures (2020−2021)
Vaccine−only strategy (2022−2023)
All numbers are rescaled to have an equivalent population to 2023.
Shaded area represents 90% prediction interval.

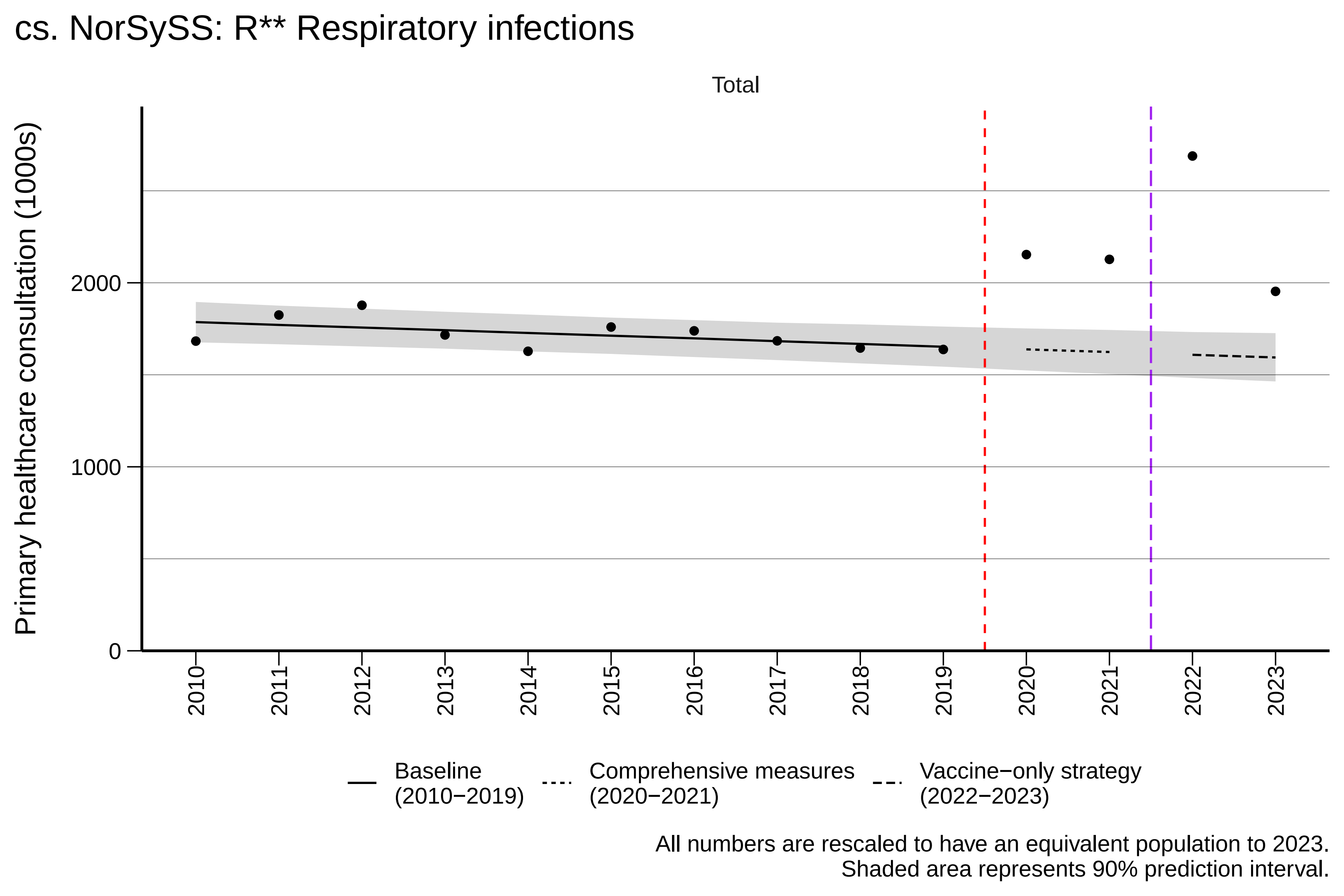
cs. NorSySS: R** Respiratory infections
Total
Primary healthcare consultation (1000s)
0
1000
2000
2010
2011
2012
2013
2014
2015
2016
2017
2018
2019
2020
2021
2022
2023
Baseline (2010−2019)
Comprehensive measures (2020−2021)
Vaccine−only strategy (2022−2023)
All numbers are rescaled to have an equivalent population to 2023.
Shaded area represents 90% prediction interval.

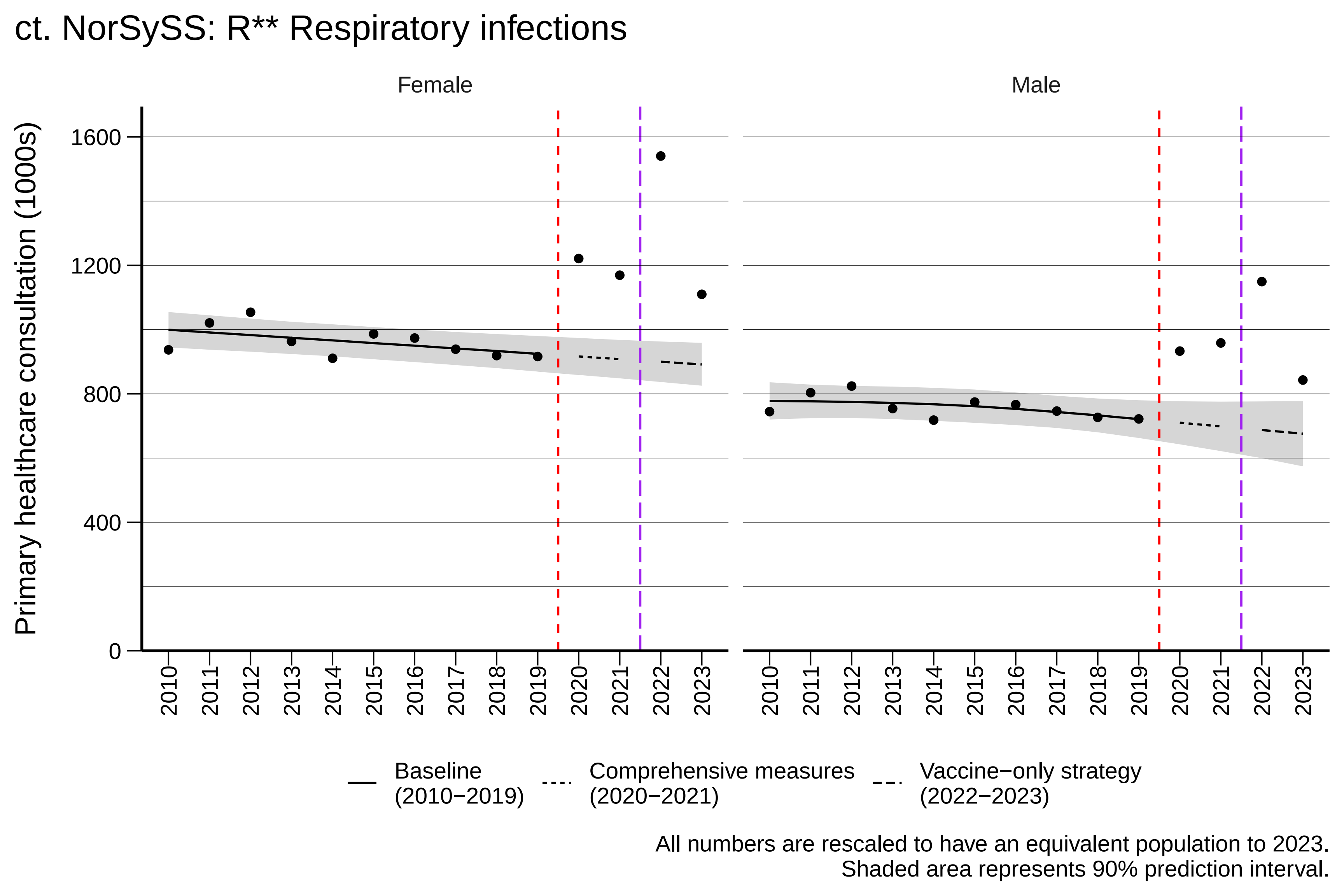
ct. NorSySS: R** Respiratory infections
Female
Male
Primary healthcare consultation (1000s)
0
400
800
1200
1600
2010
2011
2012
2013
2014
2015
2016
2017
2018
2019
2020
2021
2022
2023
Baseline
(2010−2019)
Comprehensive measures
(2020−2021)
Vaccine−only strategy
(2022−2023)
All numbers are rescaled to have an equivalent population to 2023.
Shaded area represents 90% prediction interval.

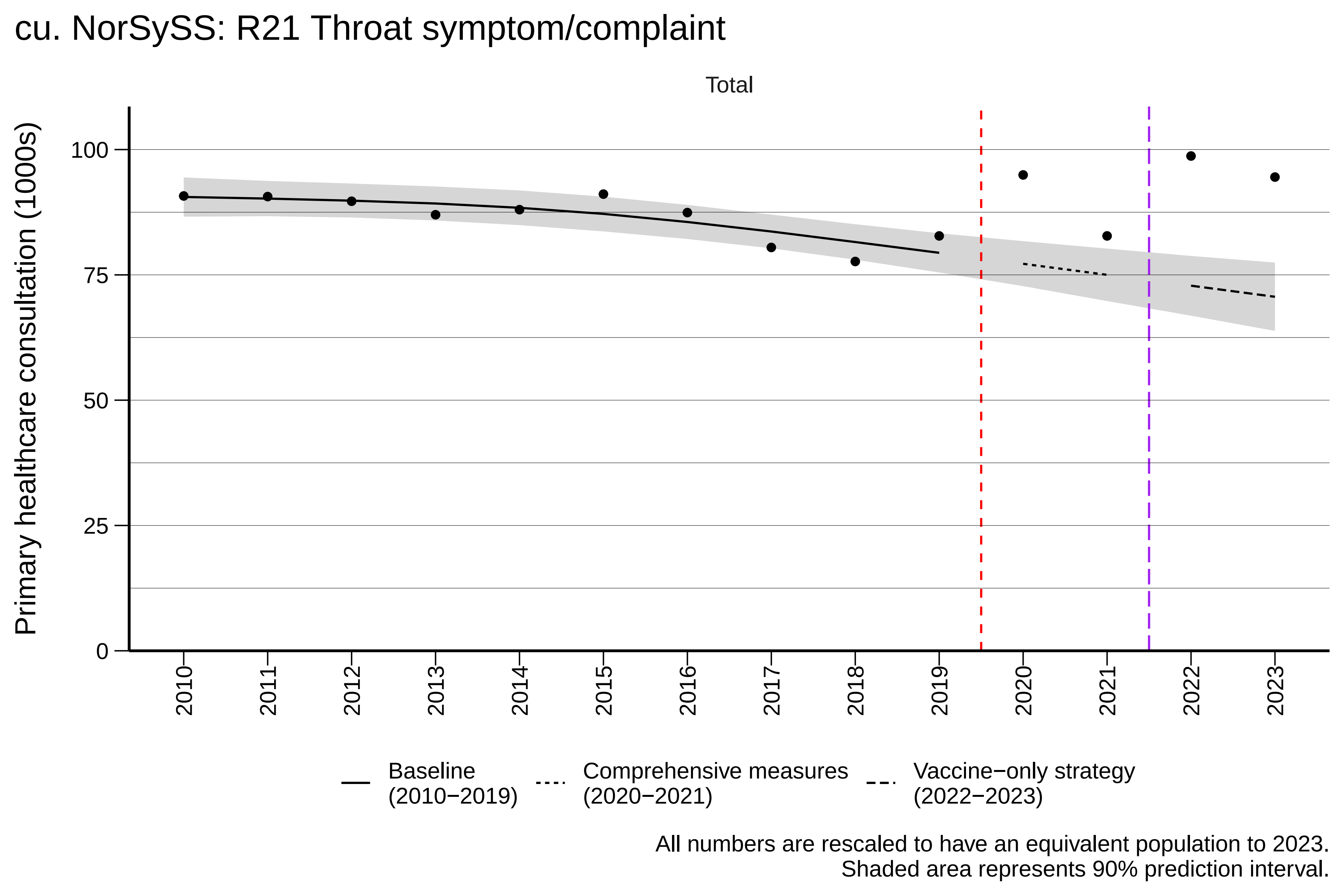
cu. NorSySS: R21 Throat symptom/complaint
Total
Primary healthcare consultation (1000s)
0
25
50
75
100
2010
2011
2012
2013
2014
2015
2016
2017
2018
2019
2020
2021
2022
2023
Baseline (2010−2019)
Comprehensive measures (2020−2021)
Vaccine−only strategy (2022−2023)
All numbers are rescaled to have an equivalent population to 2023.
Shaded area represents 90% prediction interval.

cv. NorSySS: R21 Throat symptom/complaint

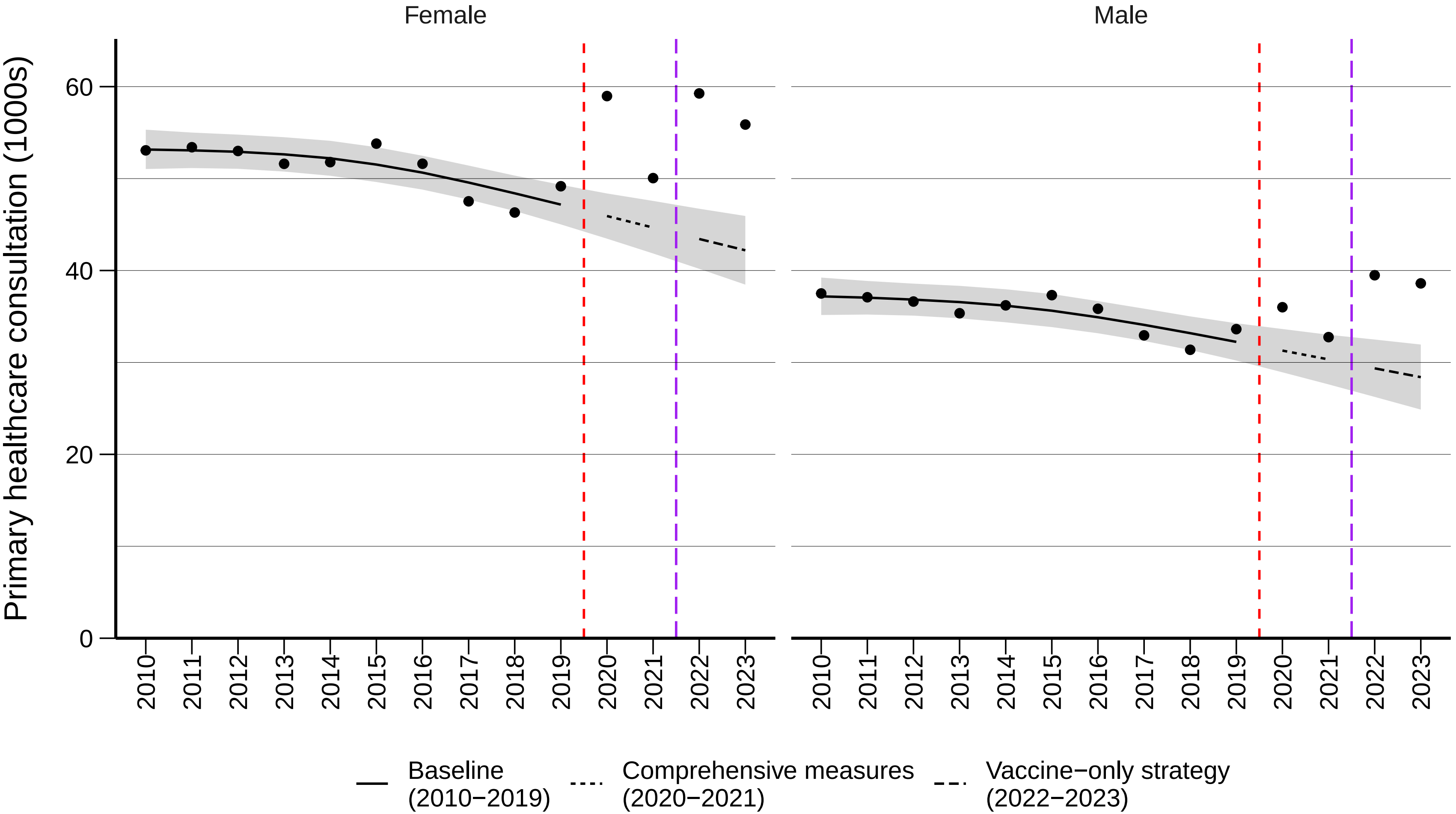

All numbers are rescaled to have an equivalent population to 2023.
Shaded area represents 90% prediction interval.

cw. NorSySS: R72 Strep throat

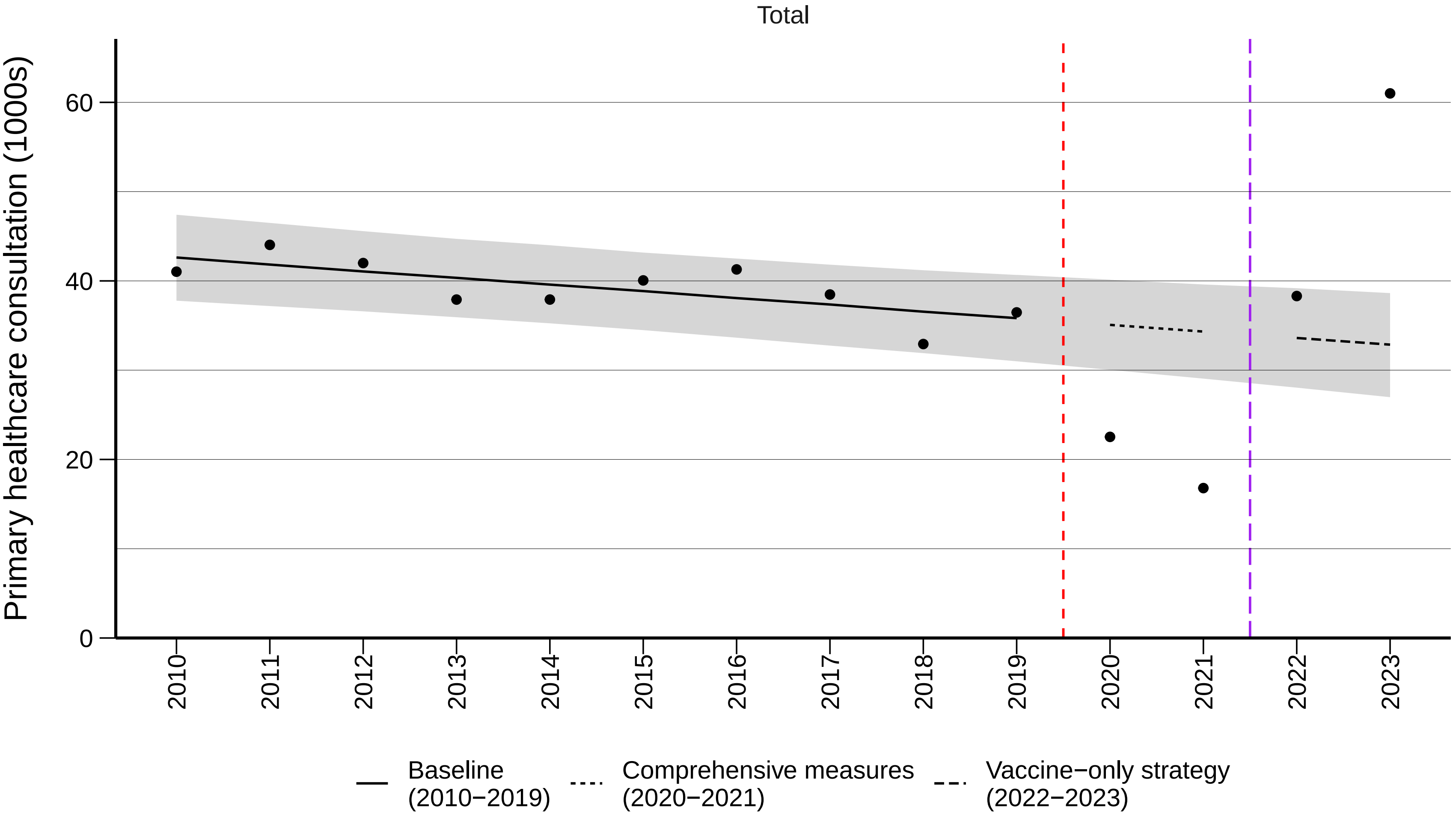

All numbers are rescaled to have an equivalent population to 2023.
Shaded area represents 90% prediction interval.

cx. NorSySS: R72 Strep throat

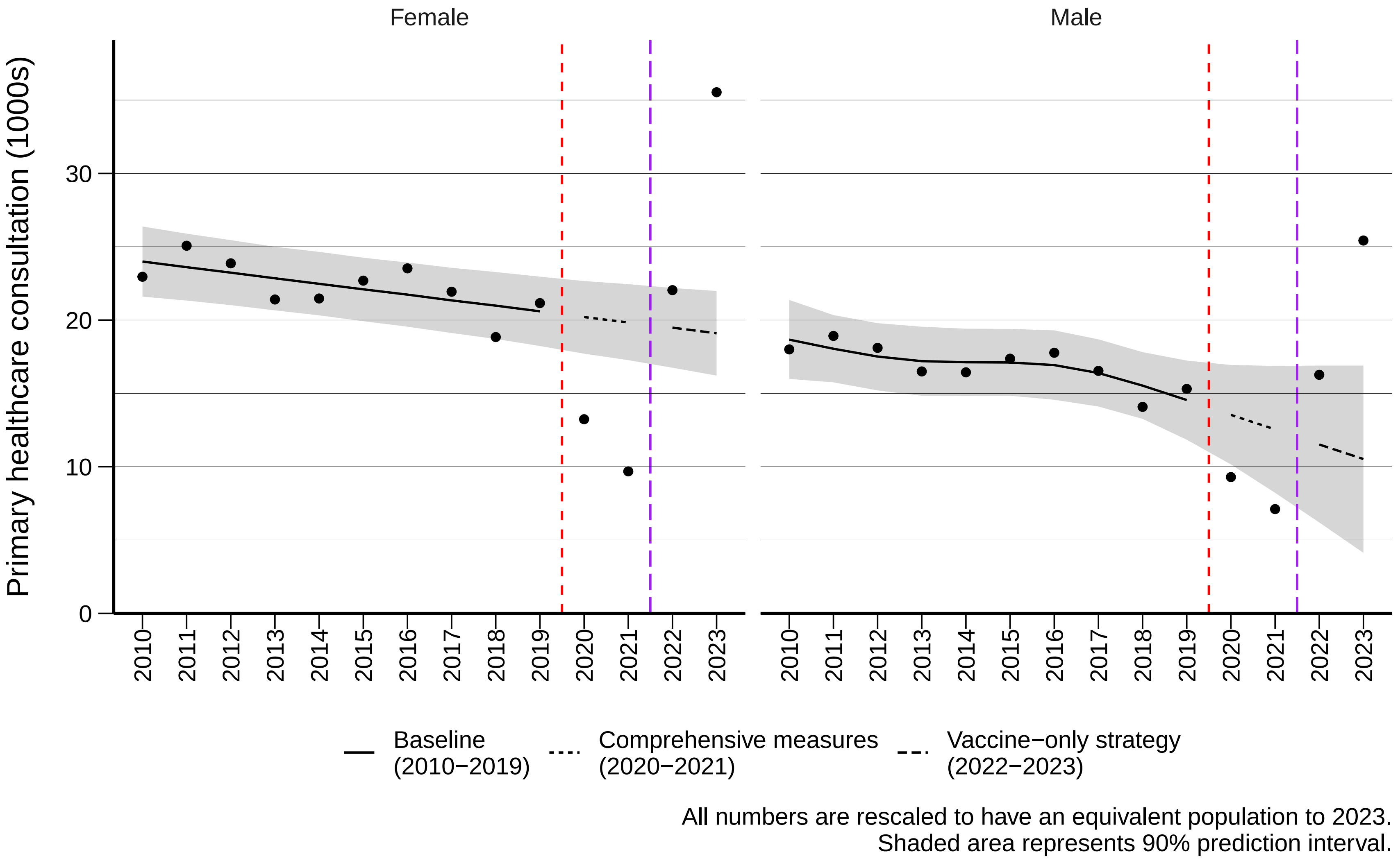

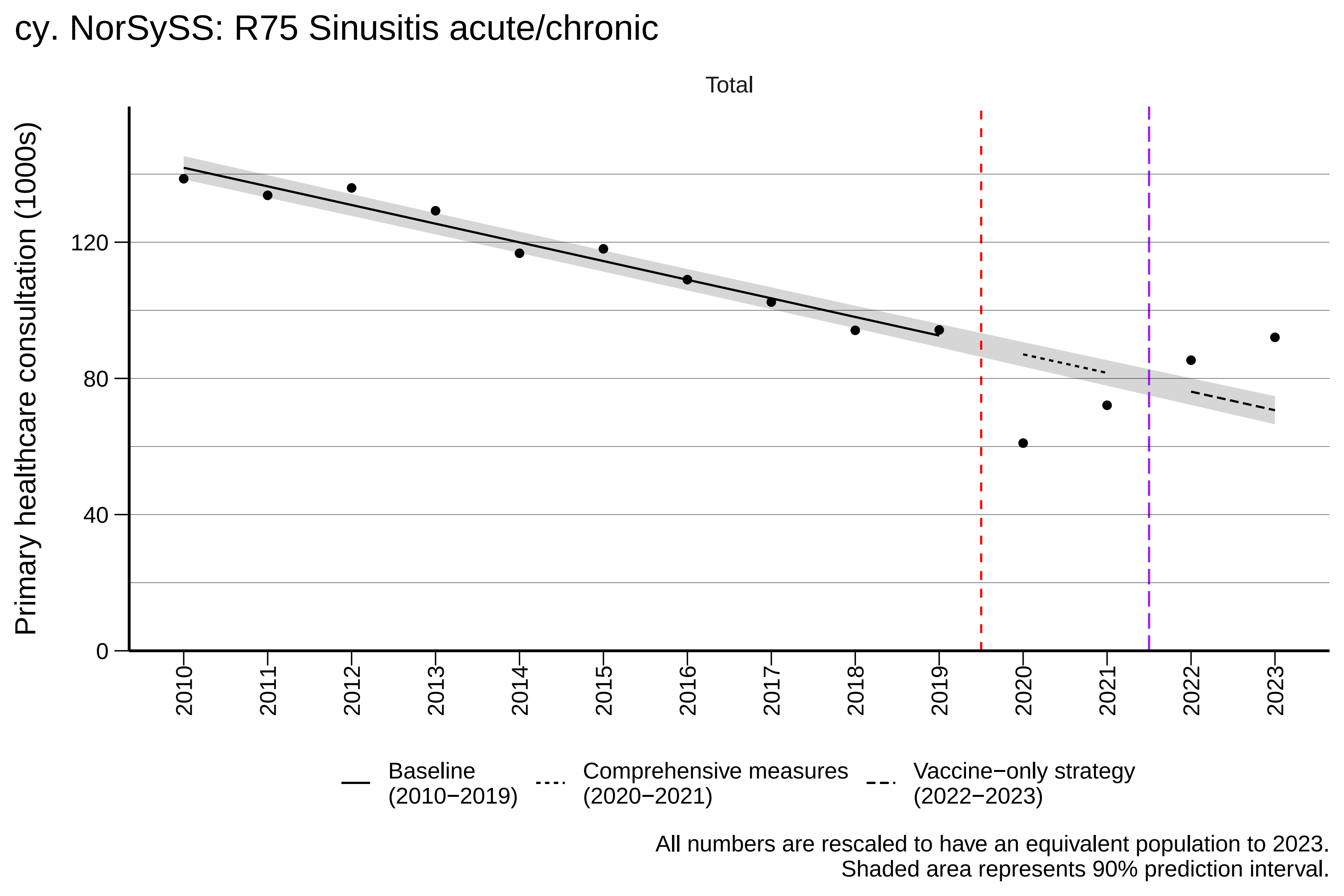
cy. NorSySS: R75 Sinusitis acute/chronic
Total
Primary healthcare consultation (1000s)
0
40
80
120
2010
2011
2012
2013
2014
2015
2016
2017
2018
2019
2020
2021
2022
2023
Baseline (2010−2019)
Comprehensive measures (2020−2021)
Vaccine−only strategy (2022−2023)
All numbers are rescaled to have an equivalent population to 2023.
Shaded area represents 90% prediction interval.

cz. NorSySS: R75 Sinusitis acute/chronic

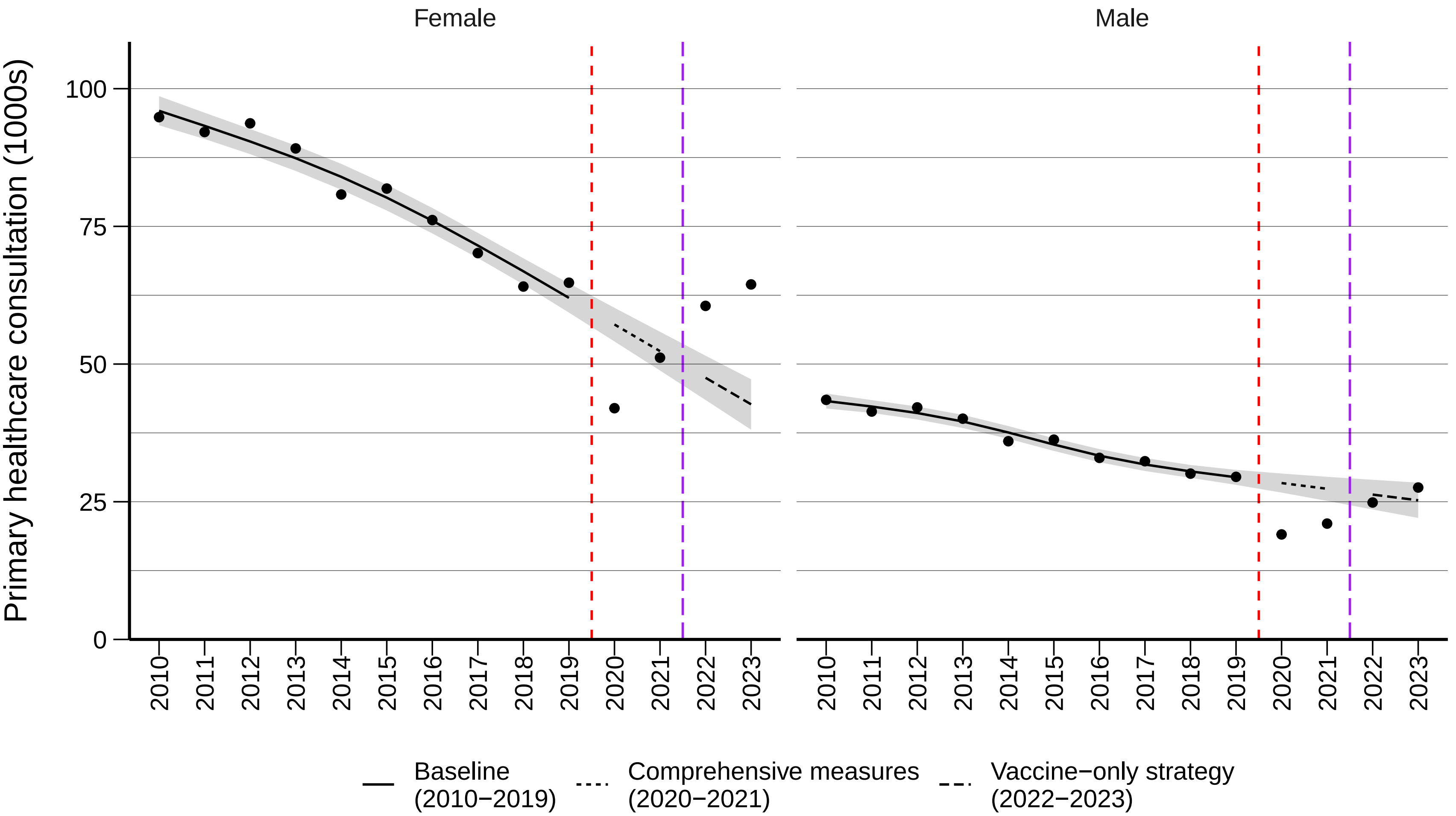

All numbers are rescaled to have an equivalent population to 2023.
Shaded area represents 90% prediction interval.

da. NorSySS: R96 Asthma

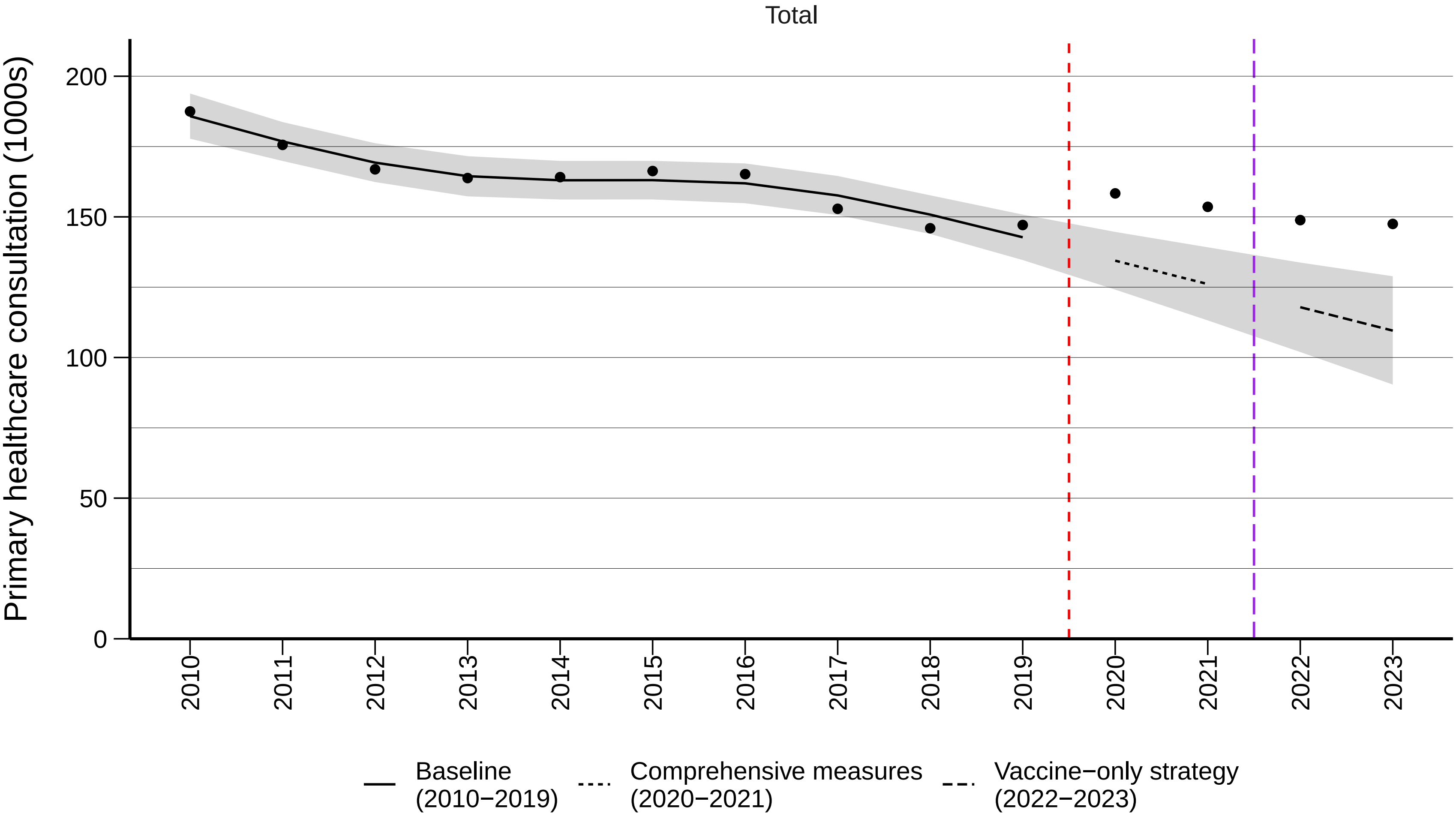

All numbers are rescaled to have an equivalent population to 2023.
Shaded area represents 90% prediction interval.

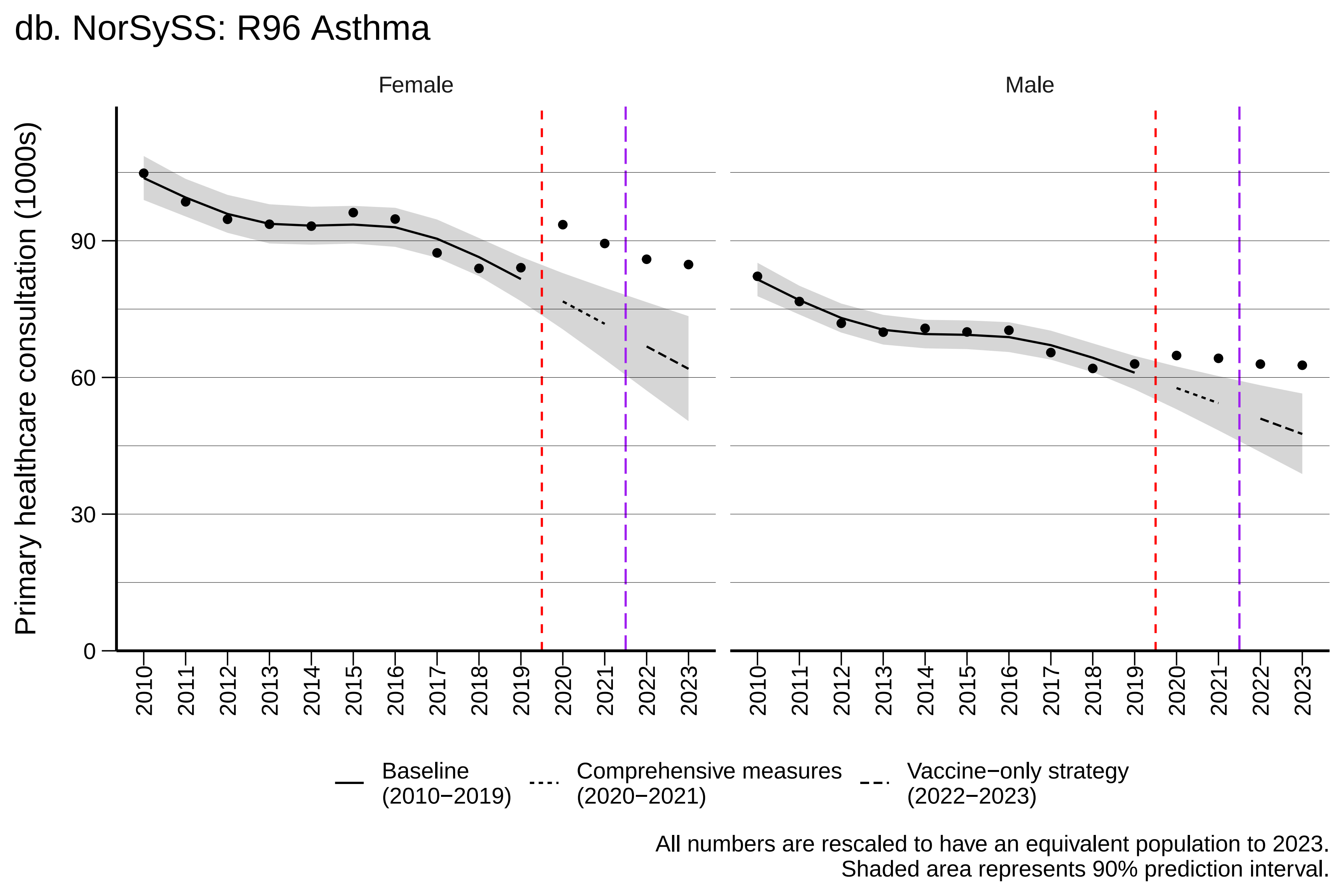

db. NorSySS: R96 Asthma
Female
Male
Primary healthcare consultation (1000s)
0
30
60
90
2010
2011
2012
2013
2014
2015
2016
2017
2018
2019
2020
2021
2022
2023
Baseline (2010−2019)
Comprehensive measures (2020−2021)
Vaccine−only strategy (2022−2023)
All numbers are rescaled to have an equivalent population to 2023.
Shaded area represents 90% prediction interval.

dc. NorSySS: S29 Skin symptom/complaint other

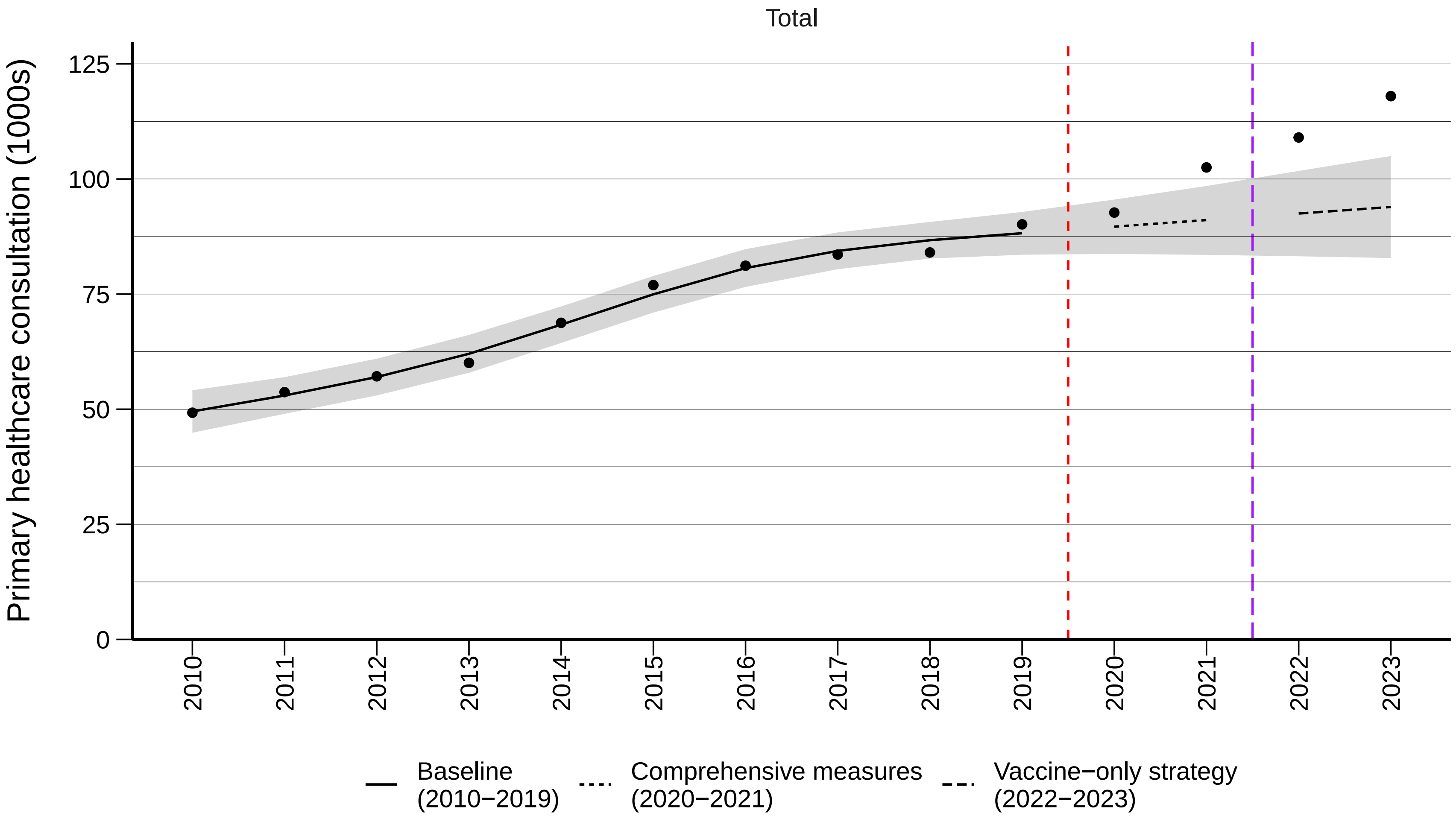

All numbers are rescaled to have an equivalent population to 2023.
Shaded area represents 90% prediction interval.

dd. NorSySS: S29 Skin symptom/complaint other

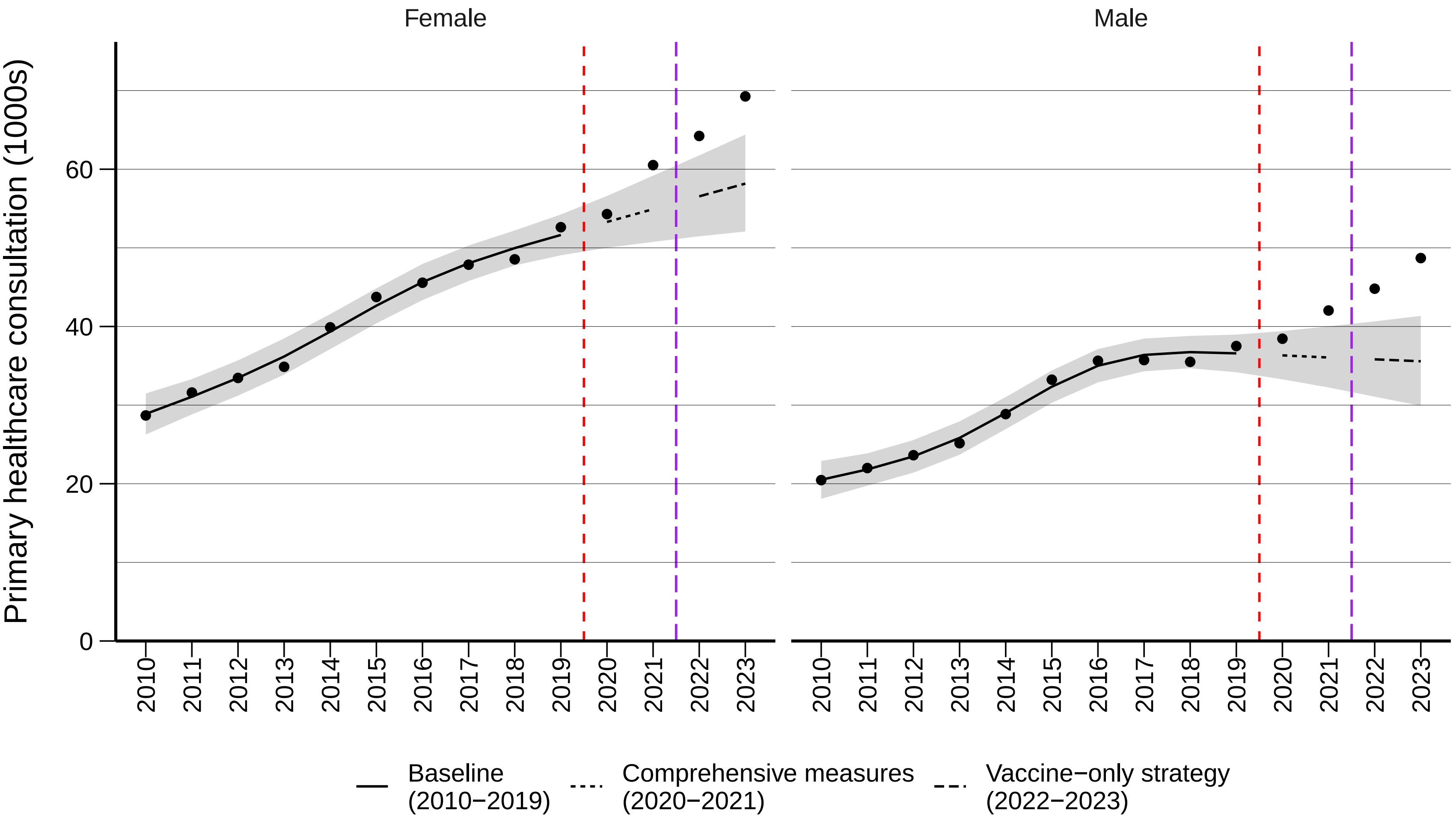

All numbers are rescaled to have an equivalent population to 2023.
Shaded area represents 90% prediction interval.
