# Supplementary material for: Aberrations in medically certified sick leave and primary healthcare consultations in Norway in 2023 compared to pre-COVID-19-pandemic trends
Source: Arch Public Health. 2024 Oct 22;82:187. doi: 10.1186/s13690-024-01411-4 (PMC11495095; doi:10.1186/s13690-024-01411-4)

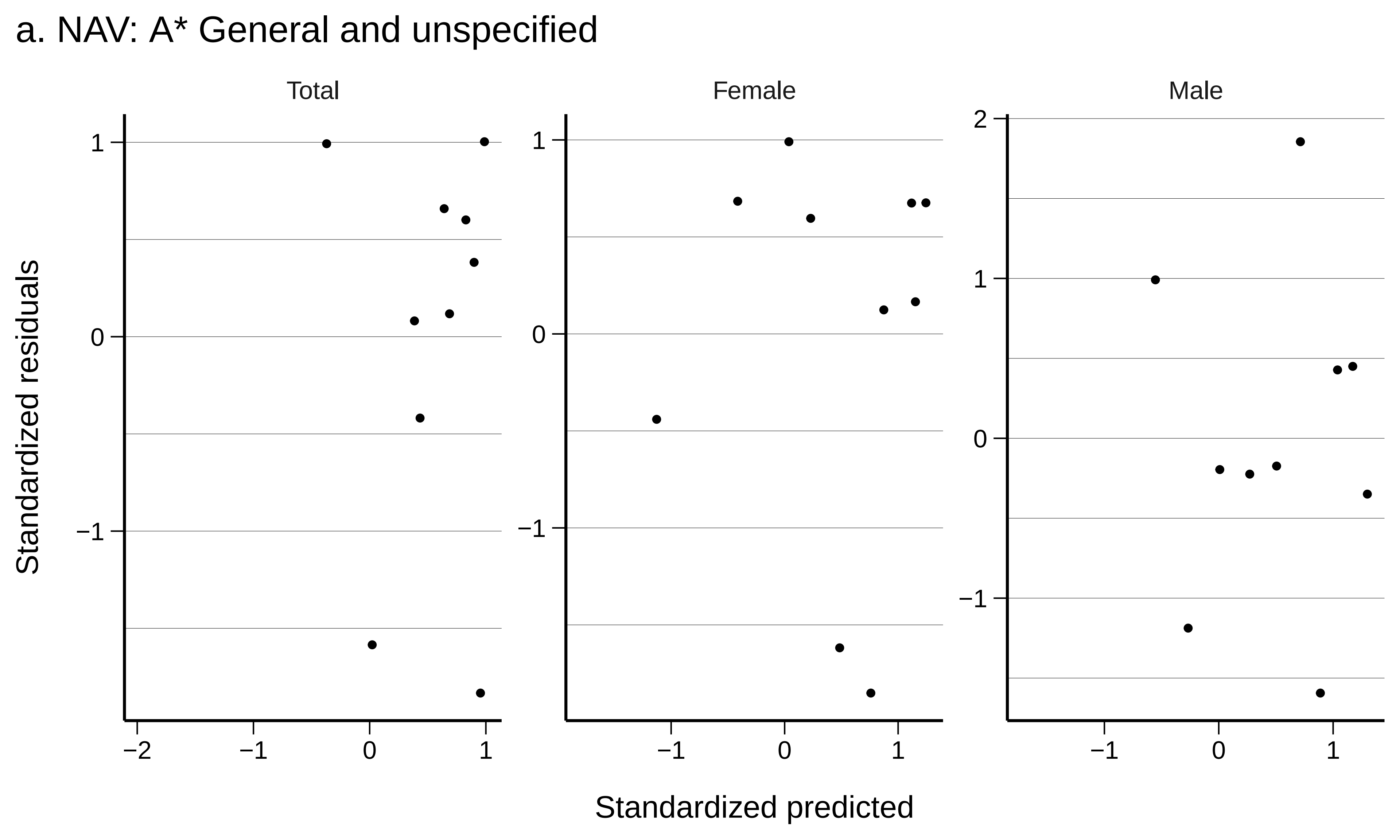

a. NAV: A* General and unspecified
Total
Female
Male
Standardized residuals
Standardized predicted

b. NAV: A* General and unspecified

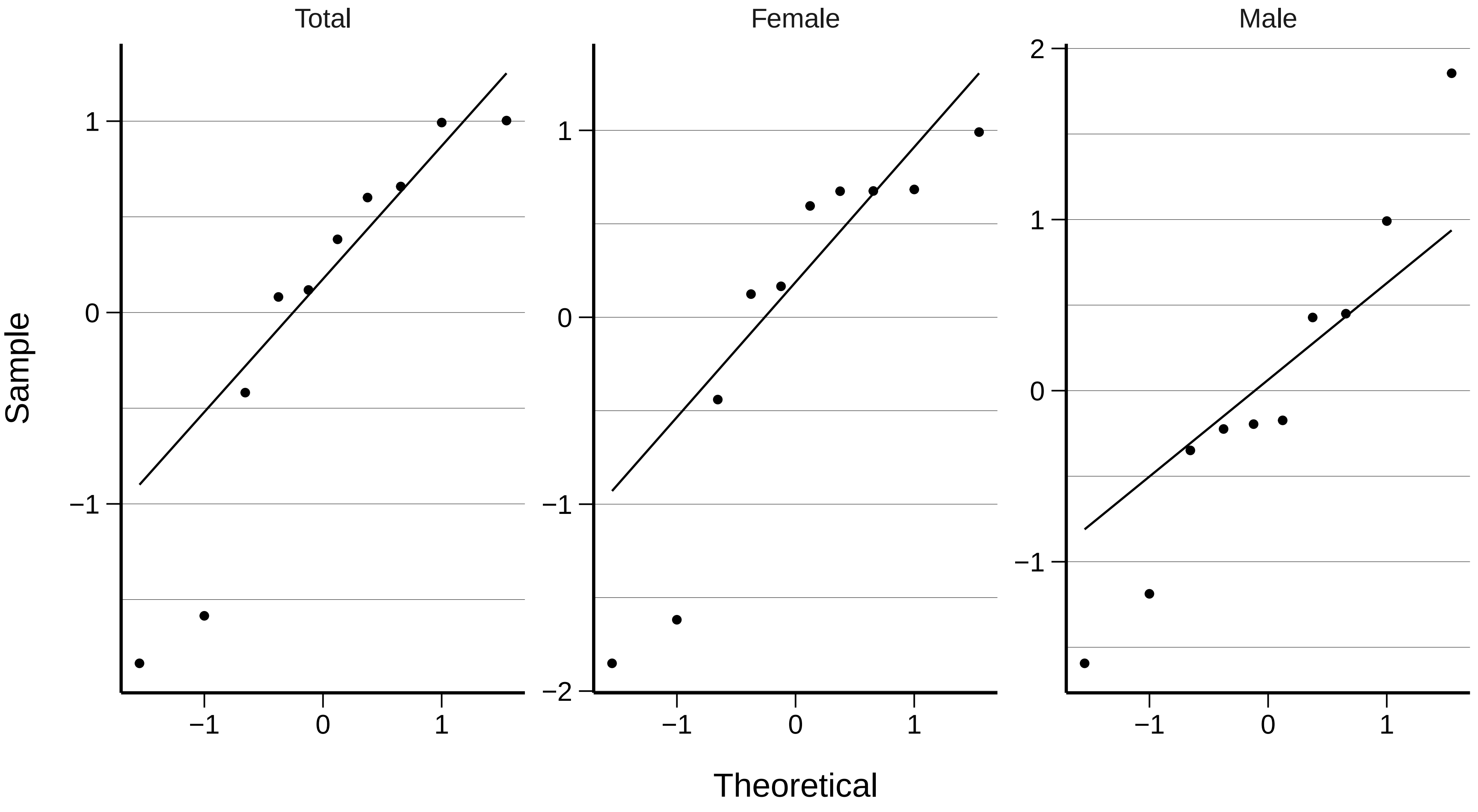

c. NAV: A01 Pain general/multiple sites

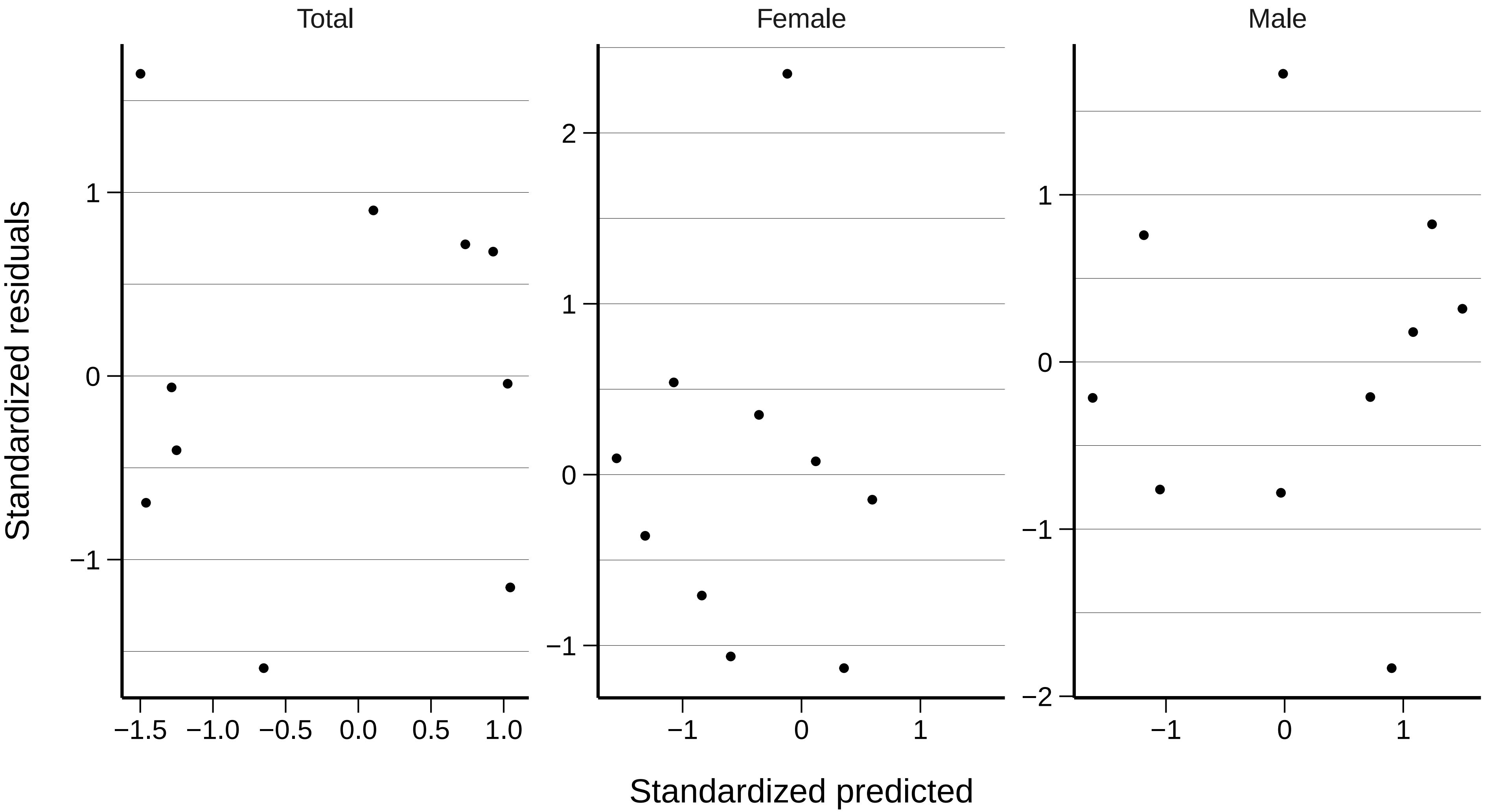

d. NAV: A01 Pain general/multiple sites

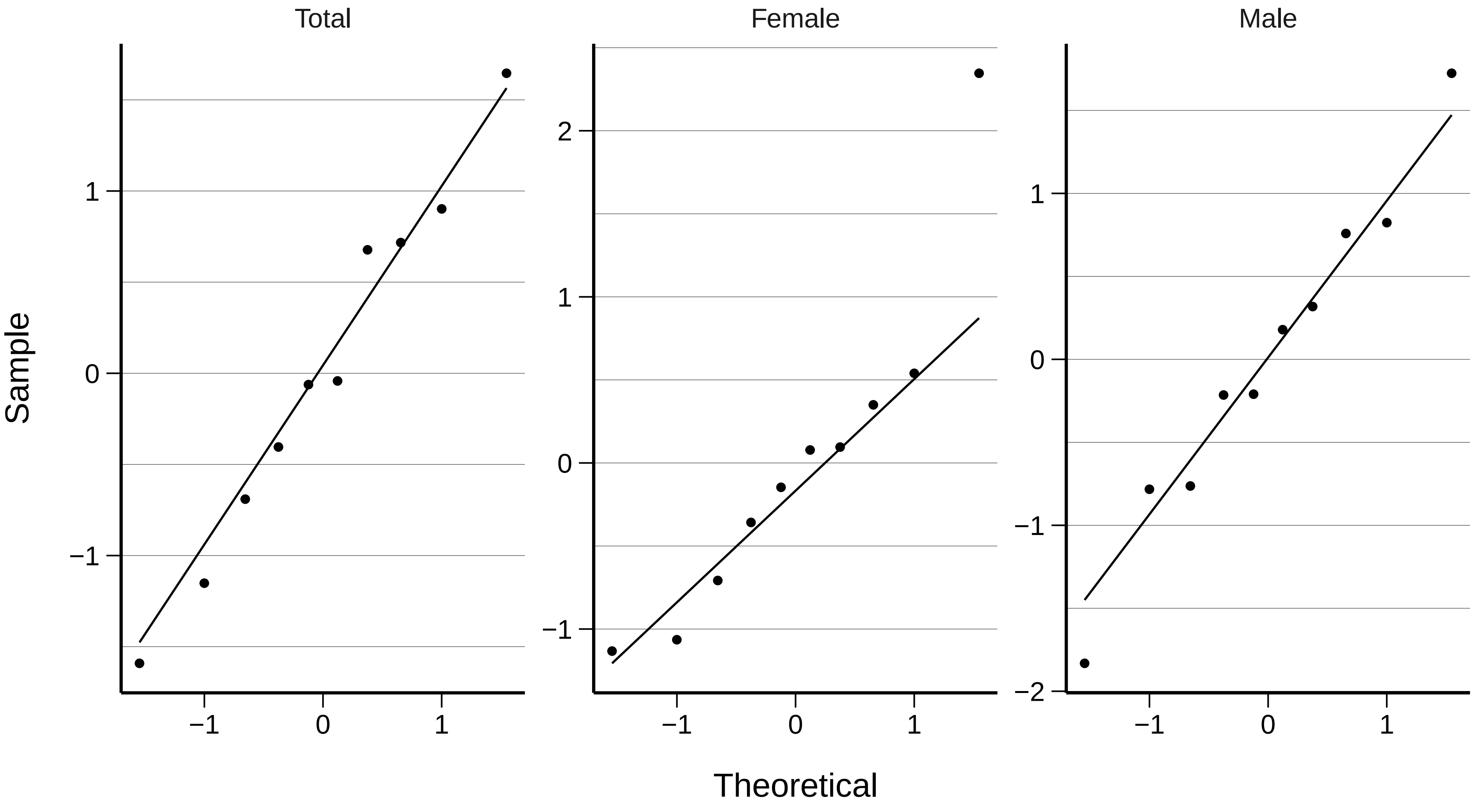

e. NAV: A03 Fever

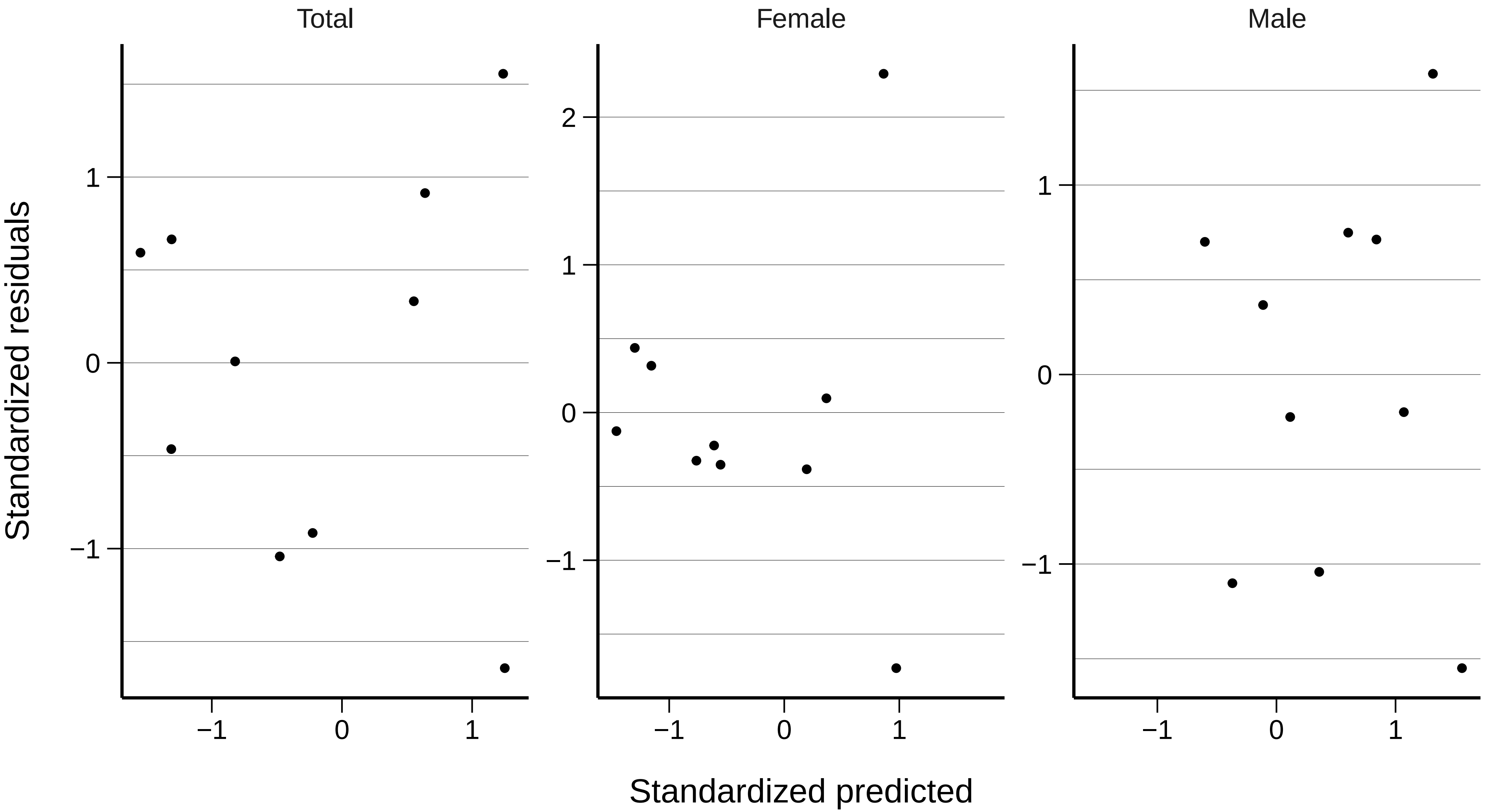

f. NAV: A03 Fever

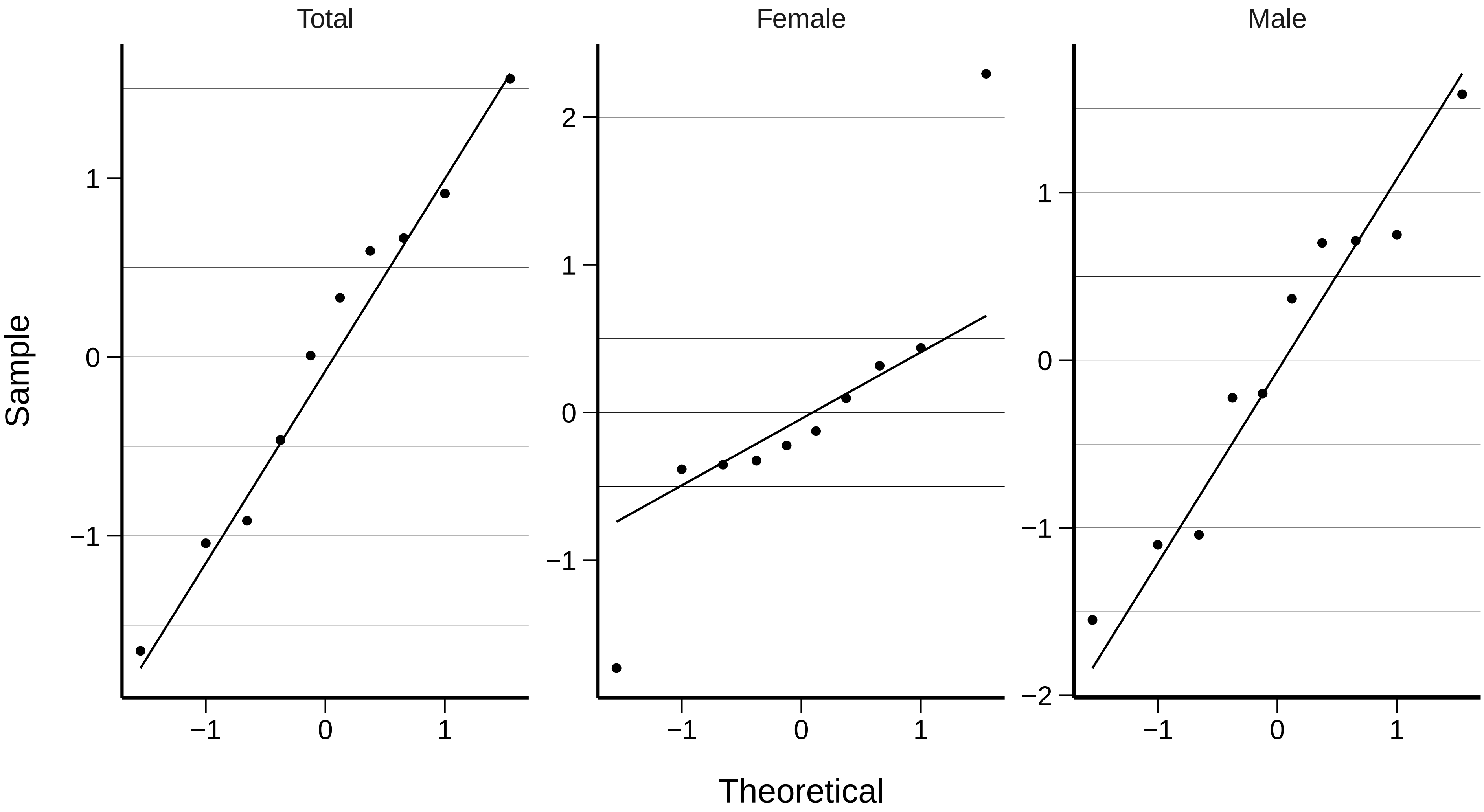

g. NAV: A04 Weakness/tiredness general

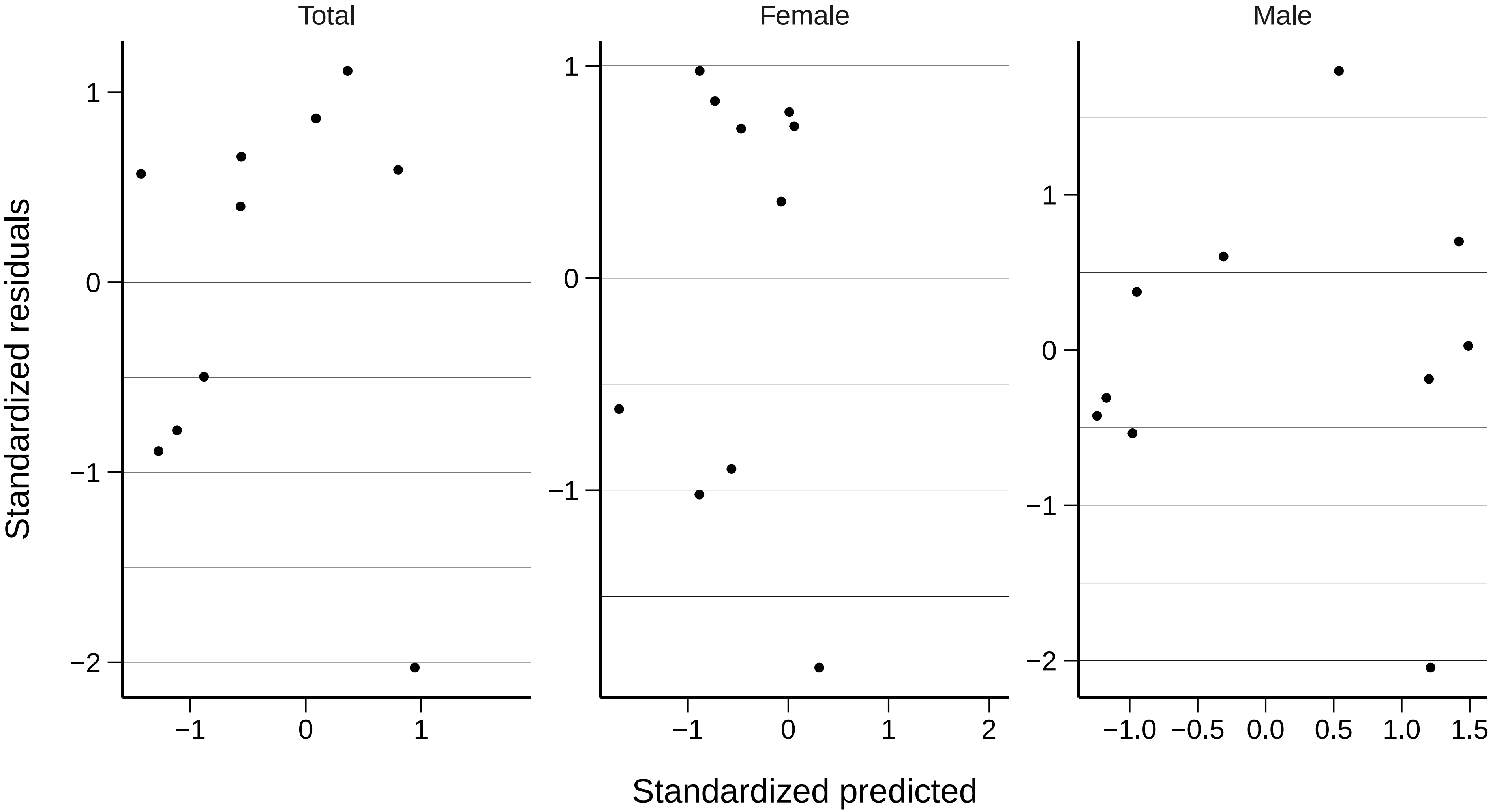

h. NAV: A04 Weakness/tiredness general

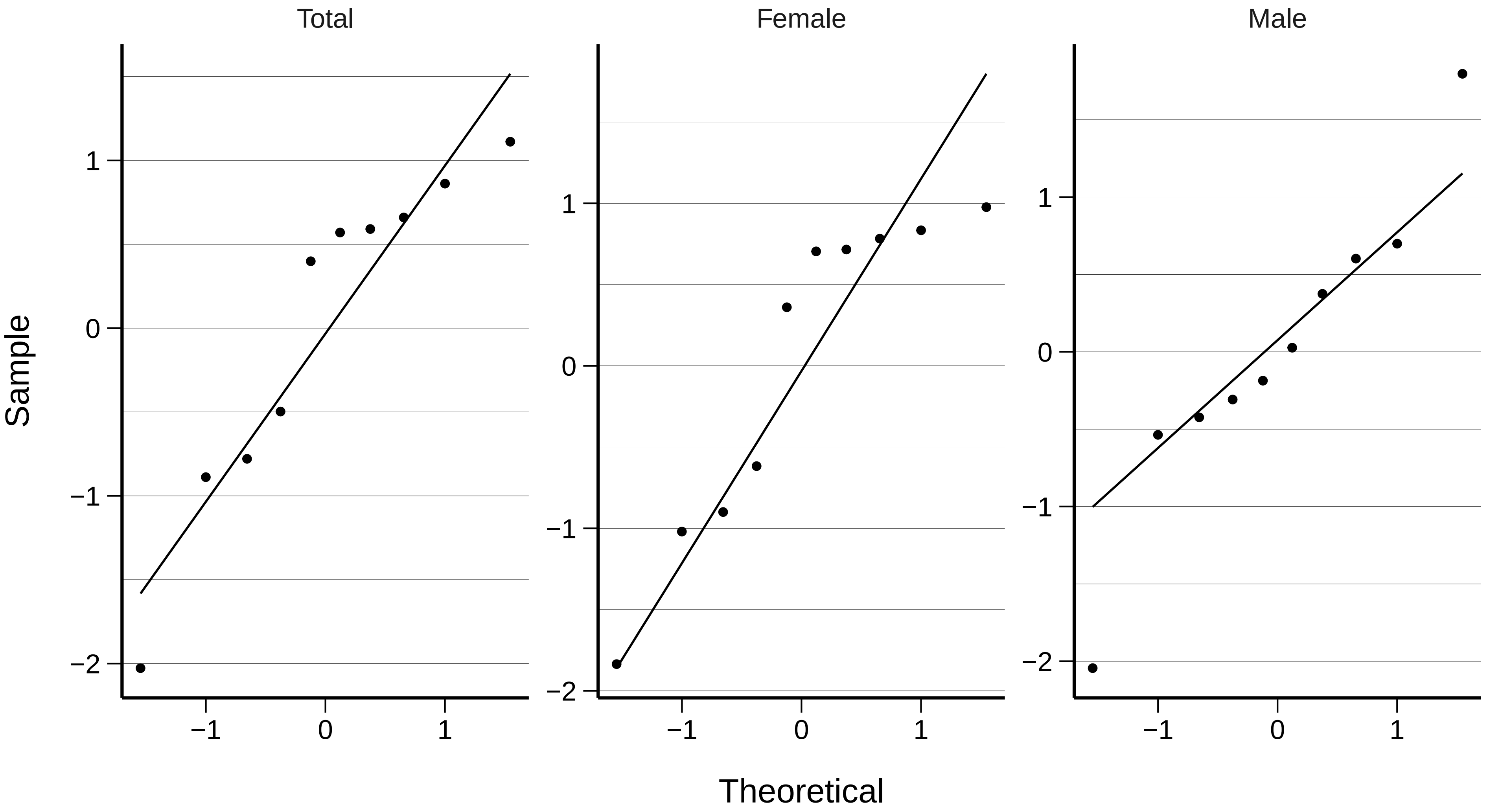

i. NAV: A77 Viral disease other/NOS

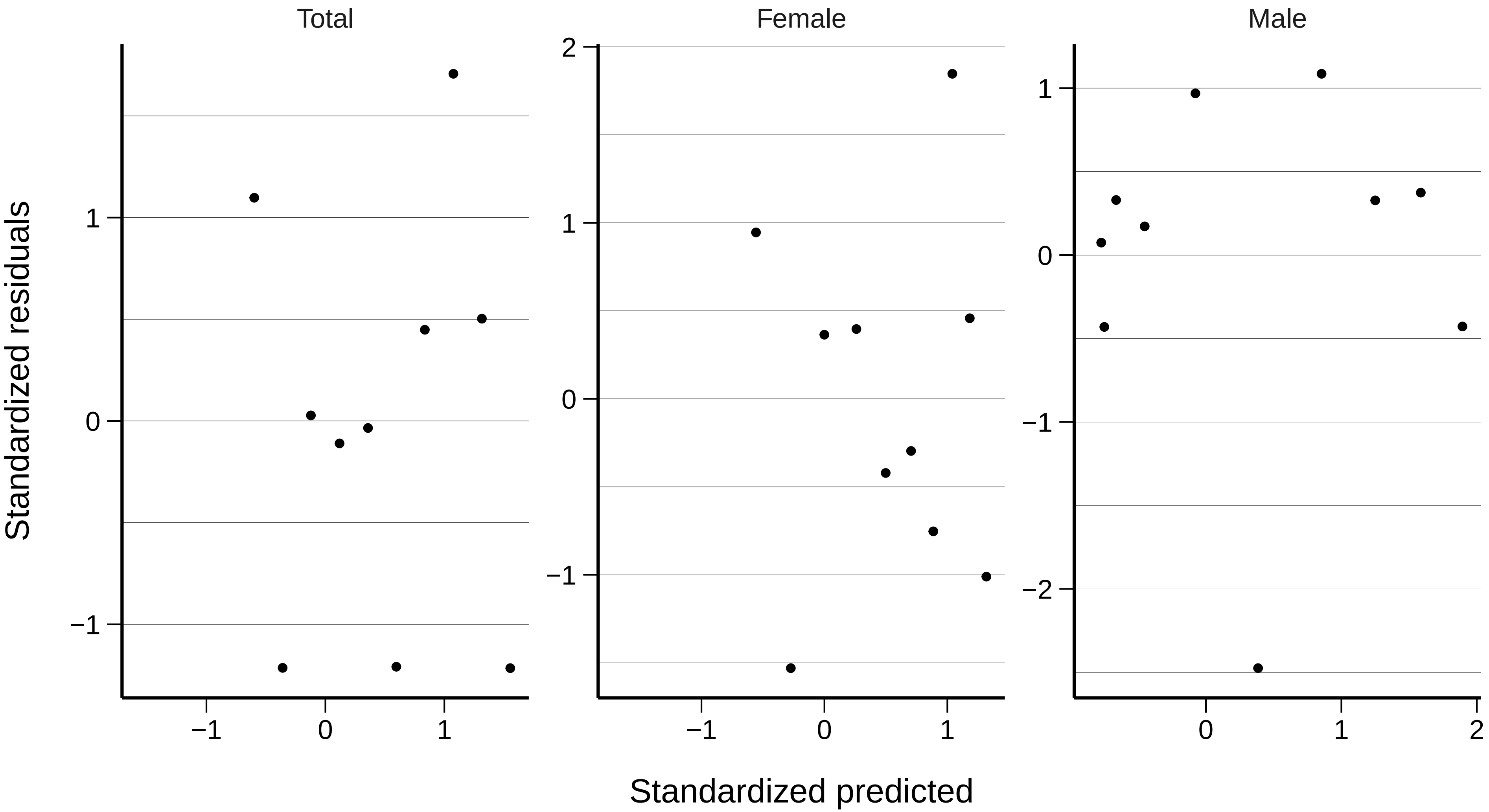

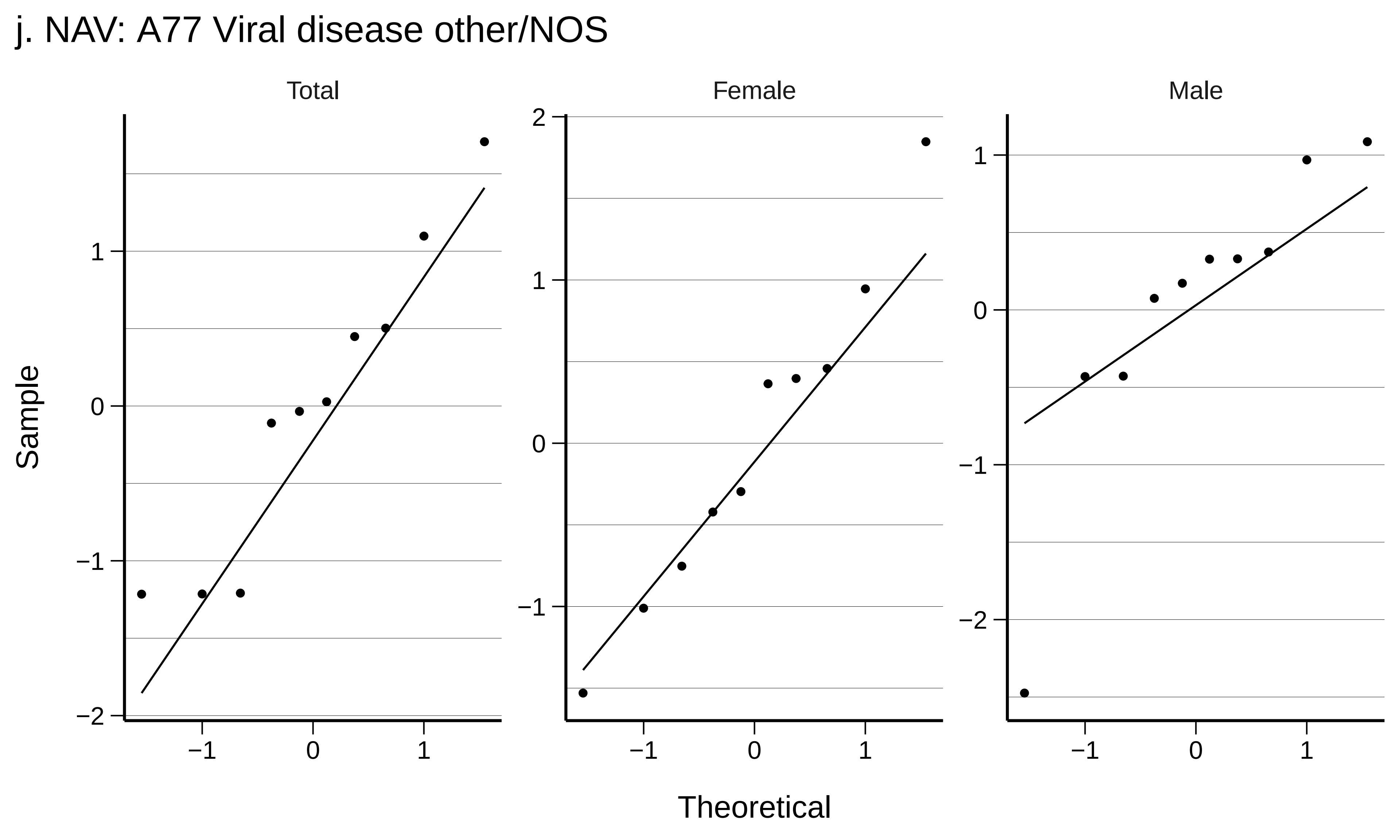
j. NAV: A77 Viral disease other/NOS
Total
Female
Male
Sample
Theoretical
1
0
−1
−2
2
1
0
−1
1
0
−1
−2
−1
0
1
−1
0
1
−1
0
1

k. NAV: A99 General disease NOS

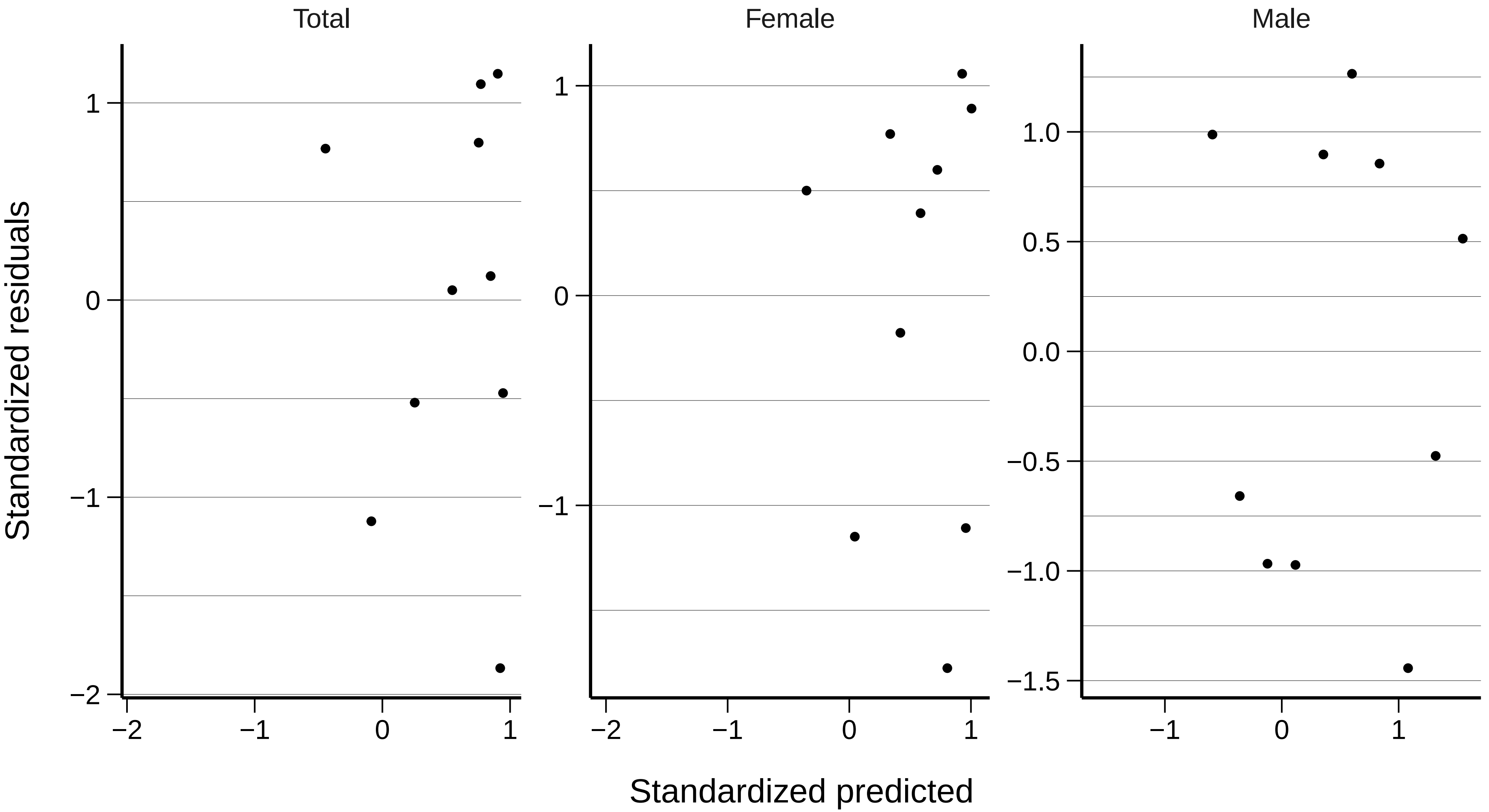

l. NAV: A99 General disease NOS

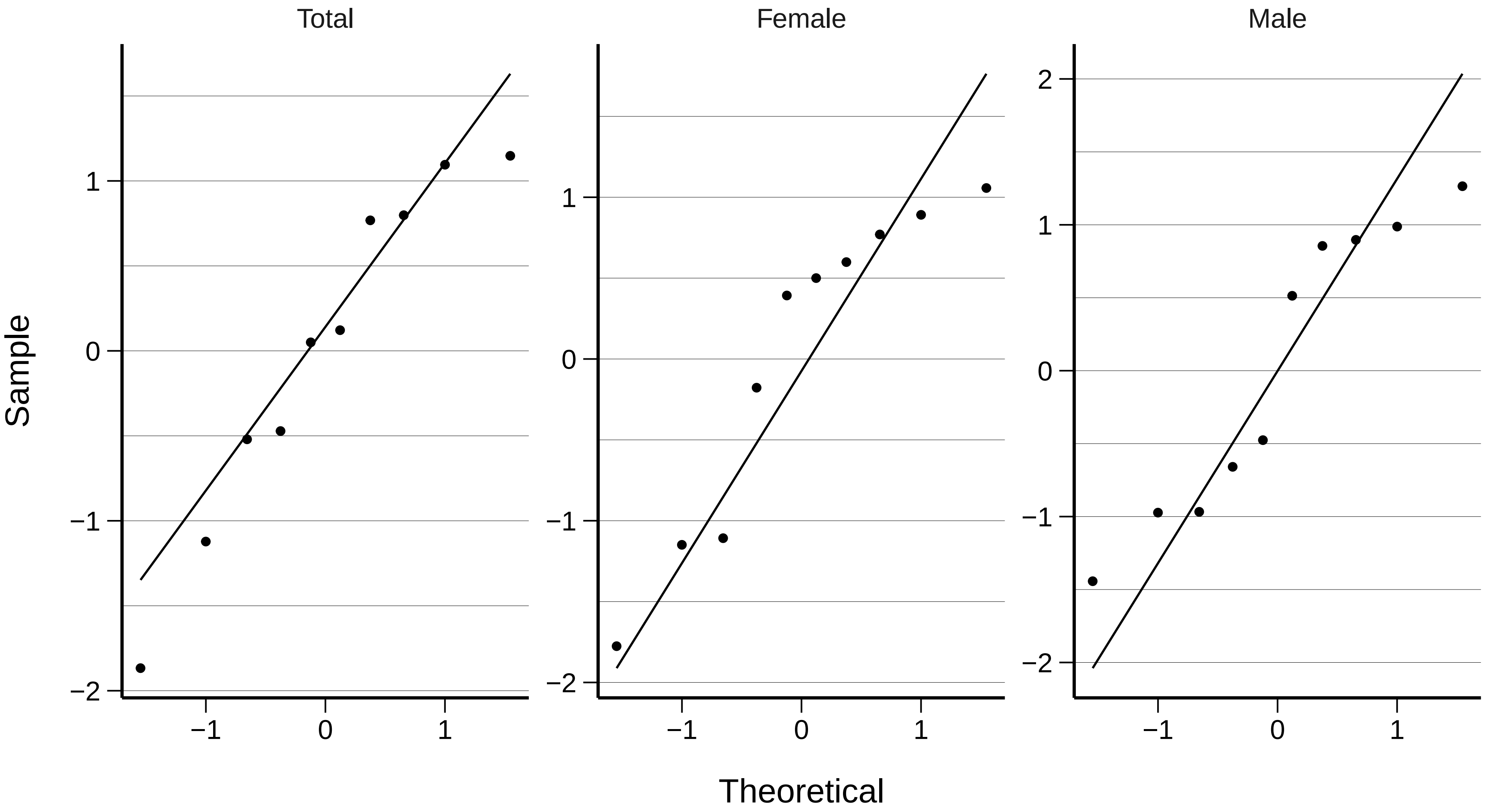

m. NAV: B99 Blood/lymph/spleen disease other

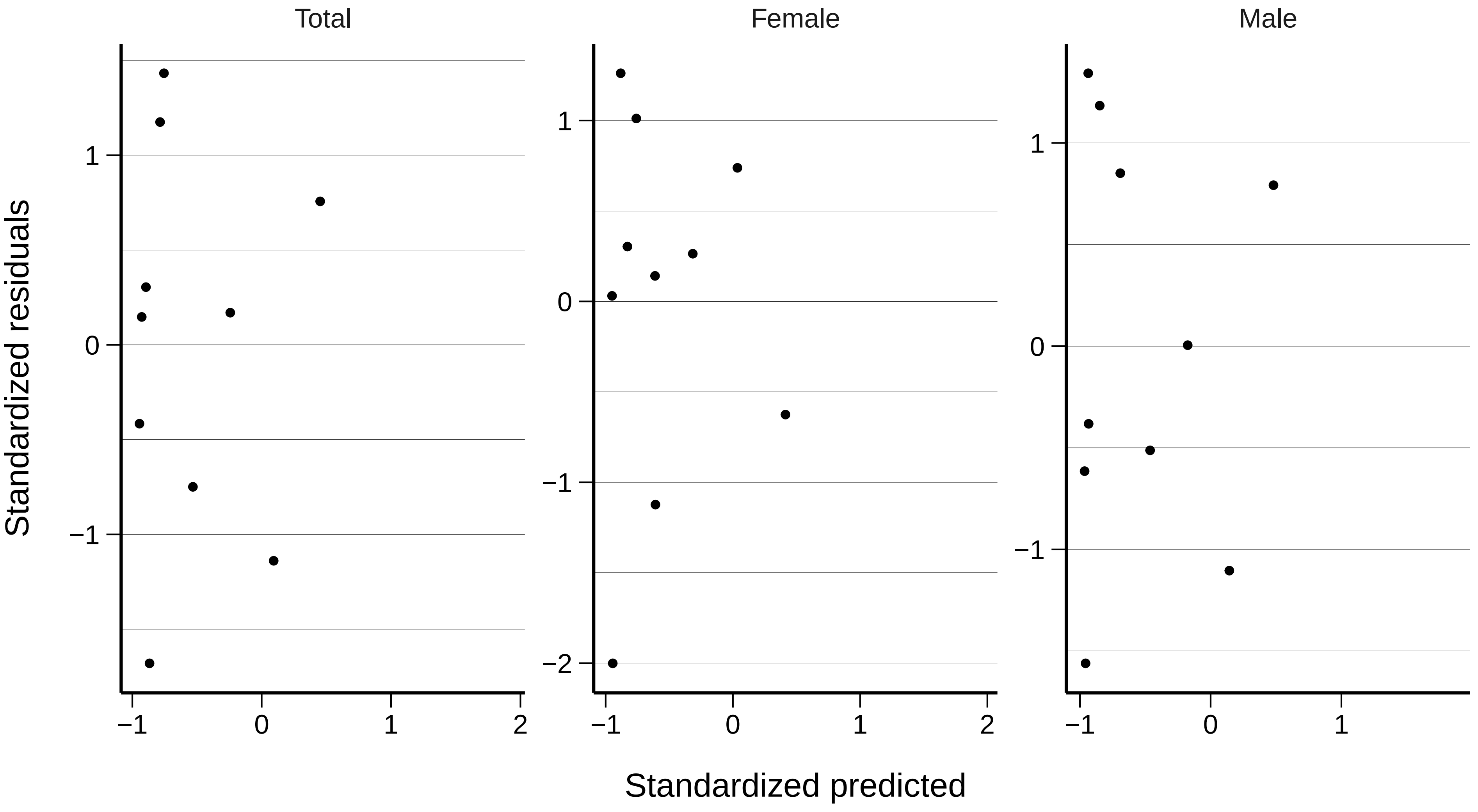

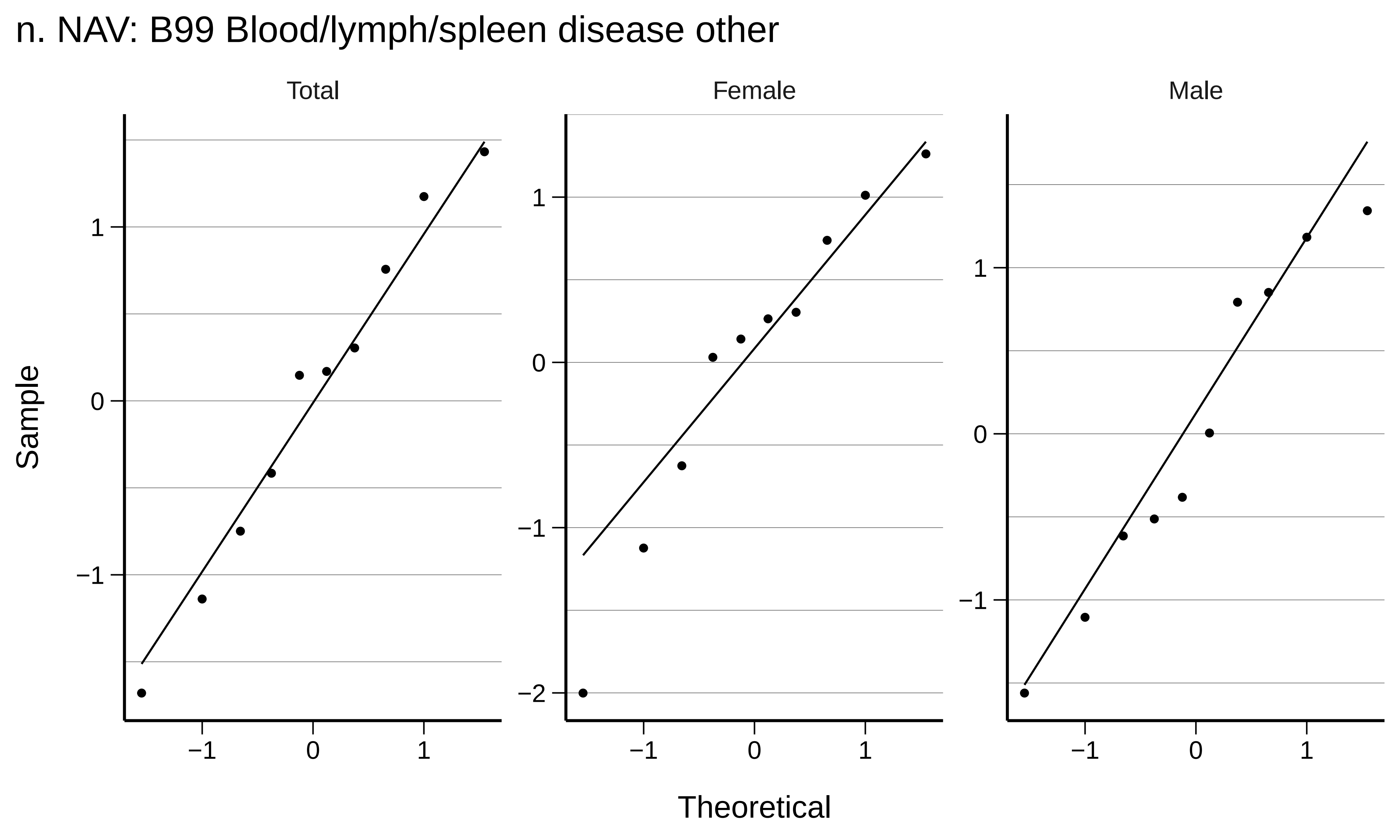
n. NAV: B99 Blood/lymph/spleen disease other
Total
Female
Male
Sample
Theoretical
1
0
−1
−2
−1
0
1

o. NAV: D04 Rectal/anal pain

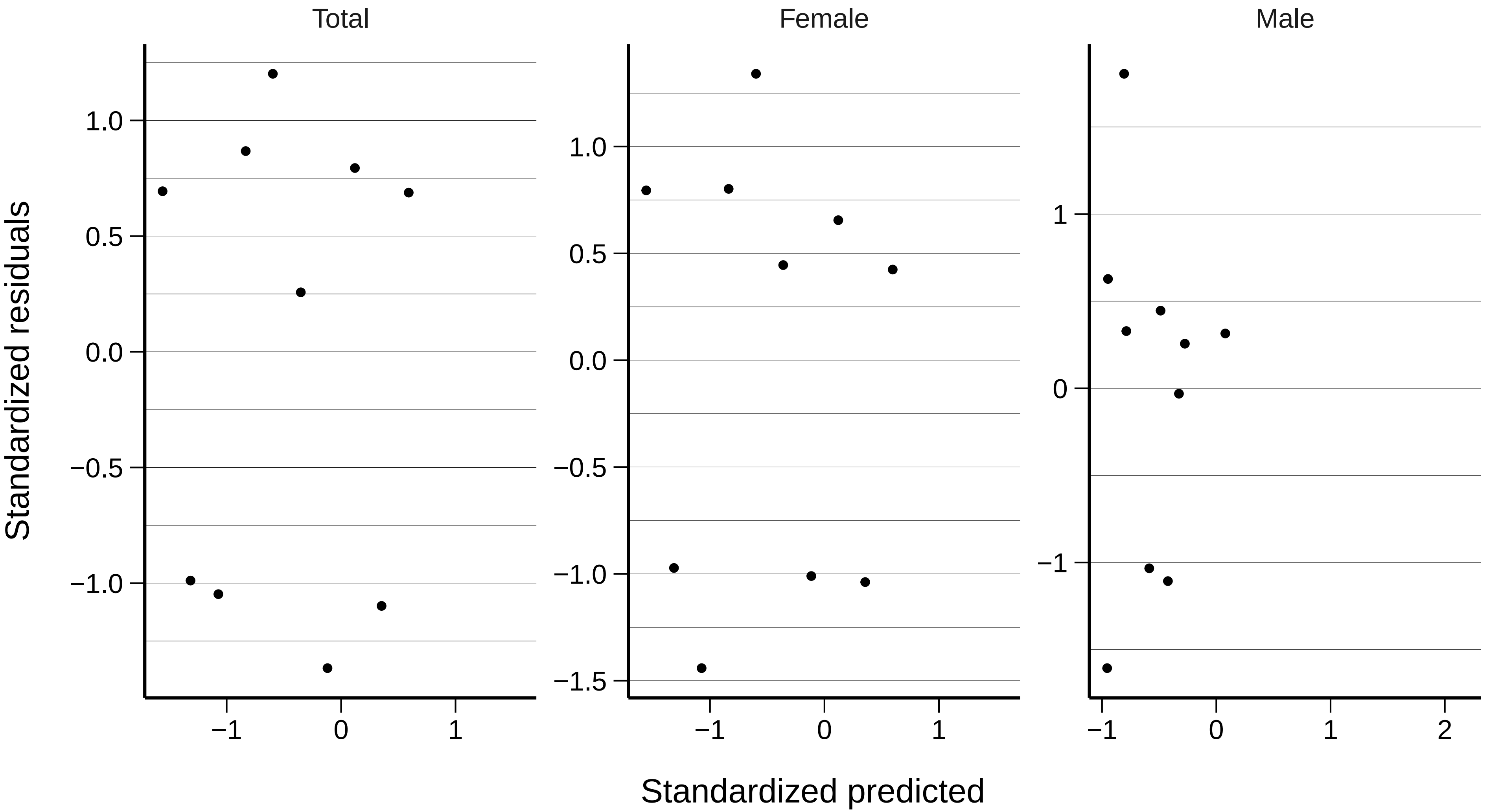

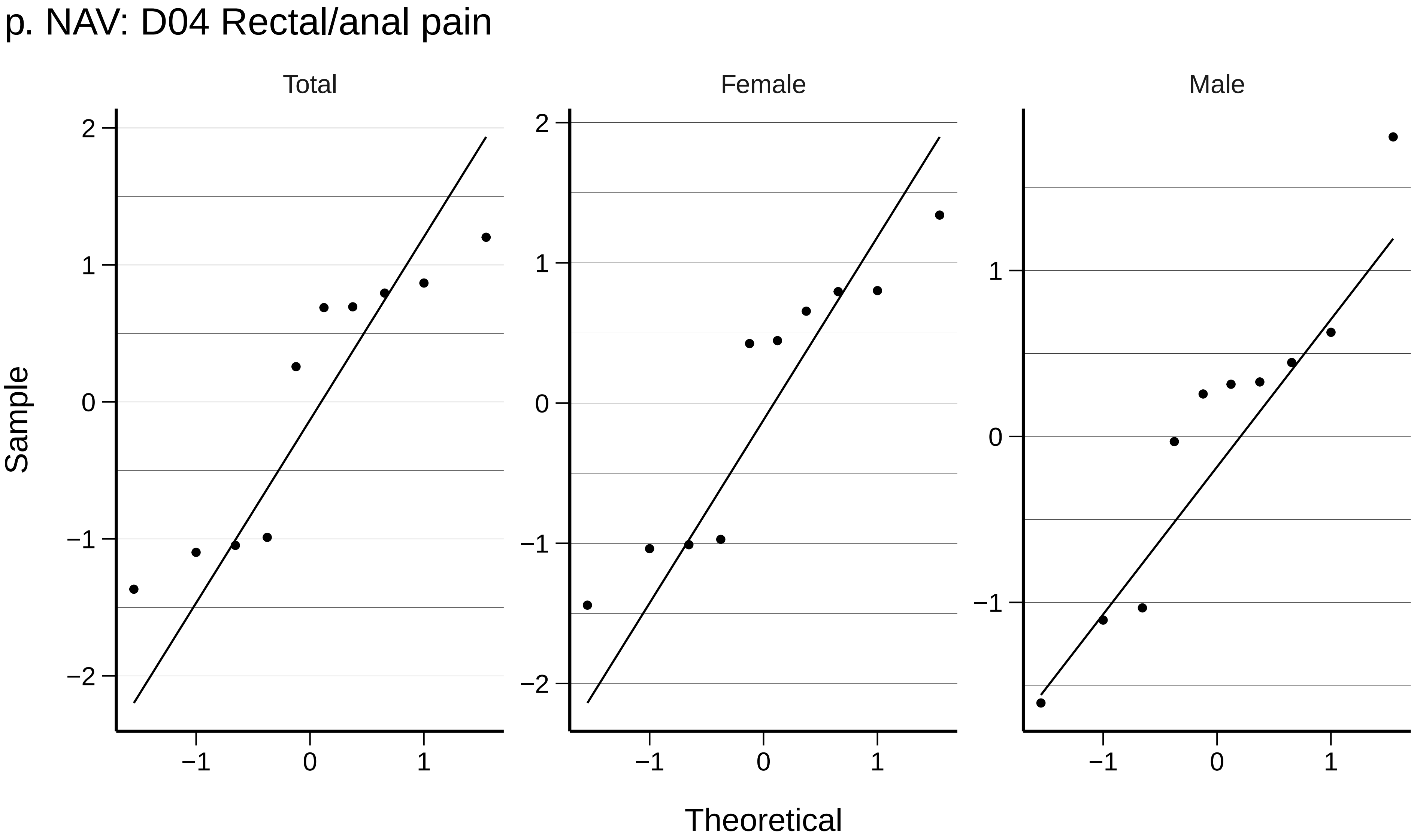

p. NAV: D04 Rectal/anal pain
Total
Female
Male
Sample
Theoretical
2
1
0
−1
−2
−1
0
1

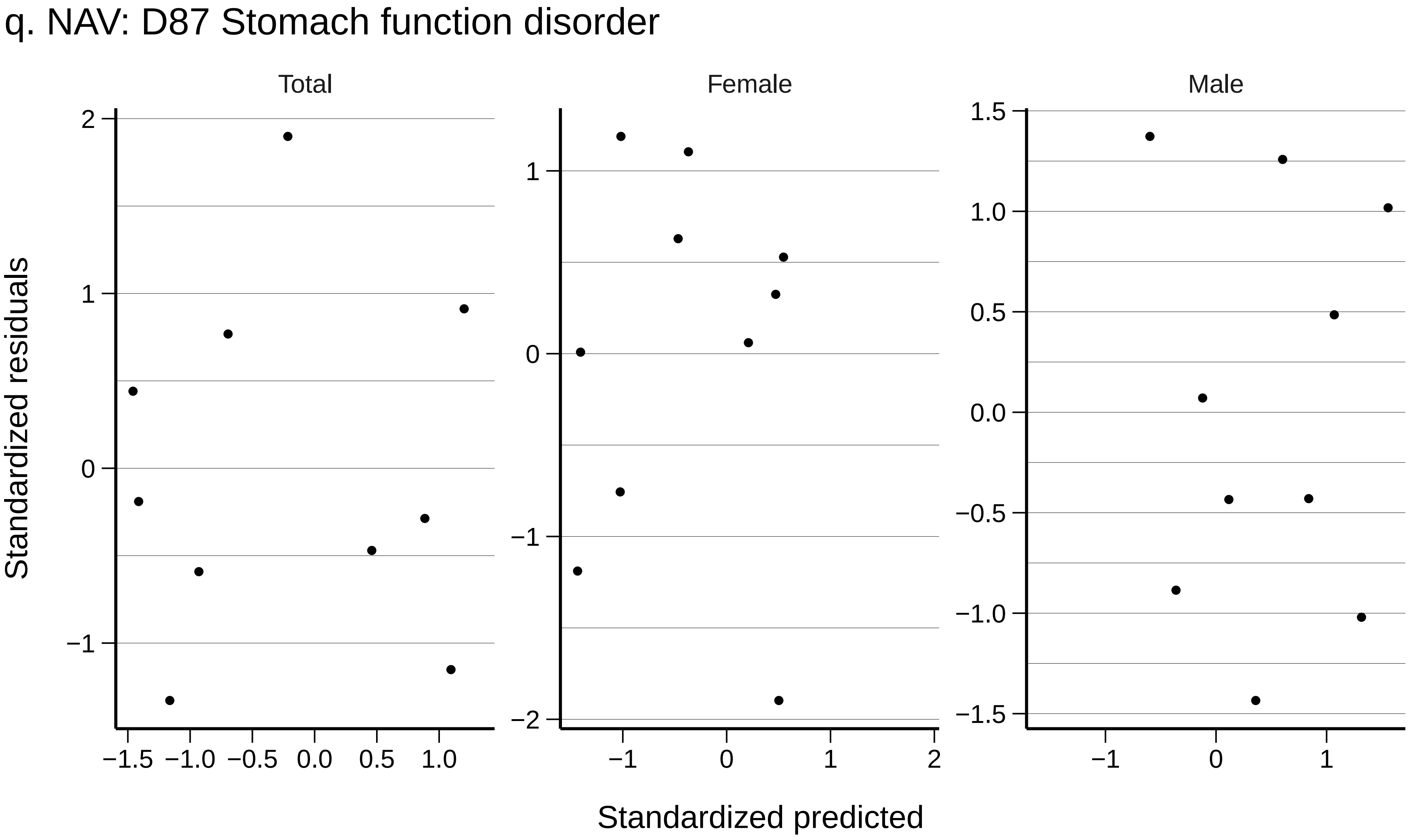
q. NAV: D87 Stomach function disorder
Total
Female
Male
Standardized residuals
Standardized predicted
2
1
0
−1
−1.5
−1.0
−0.5
0.0
0.5
1.0
−2
2
1.5
−1.5

r. NAV: D87 Stomach function disorder

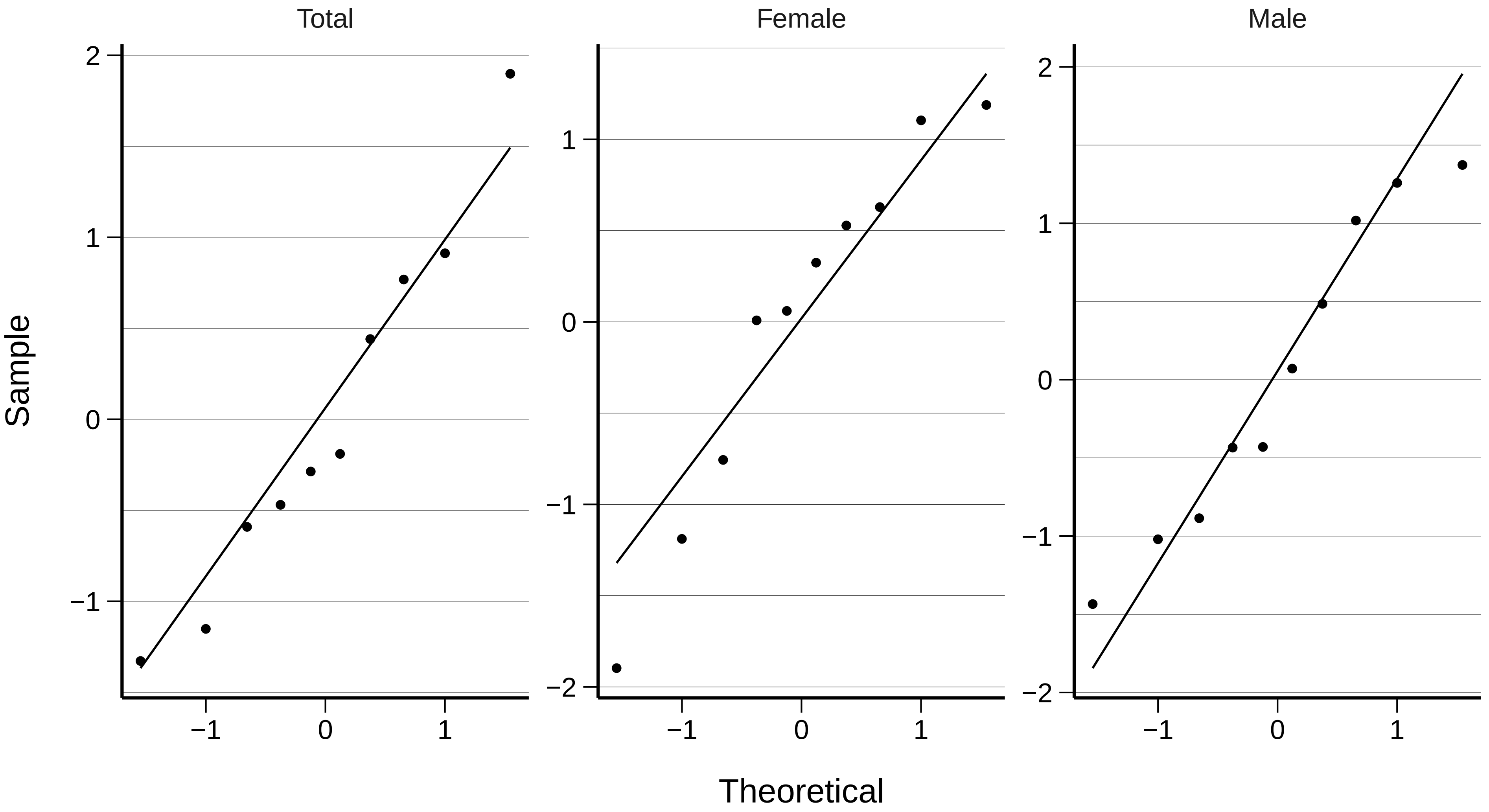

s. NAV: D92 Diverticular disease

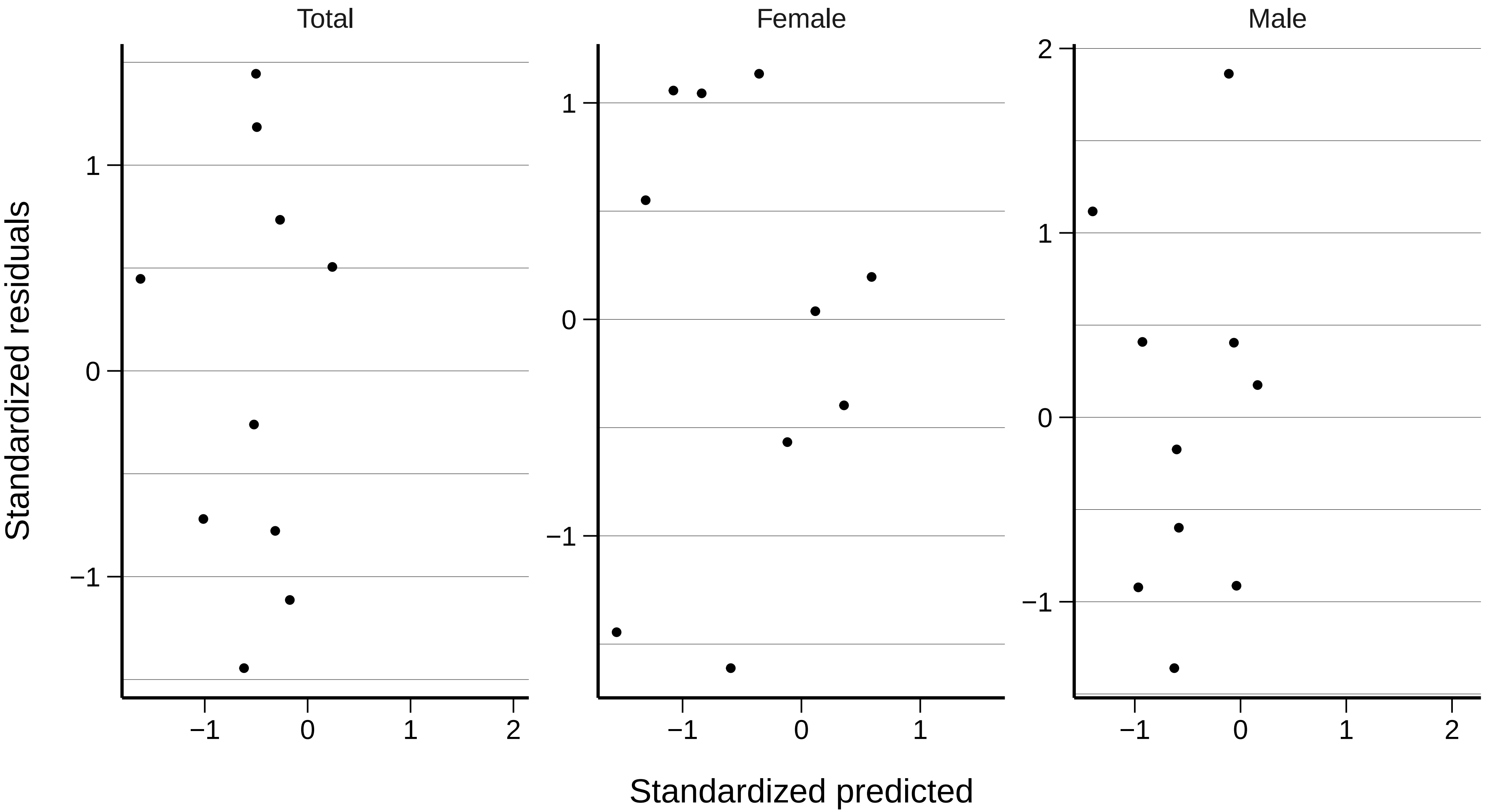

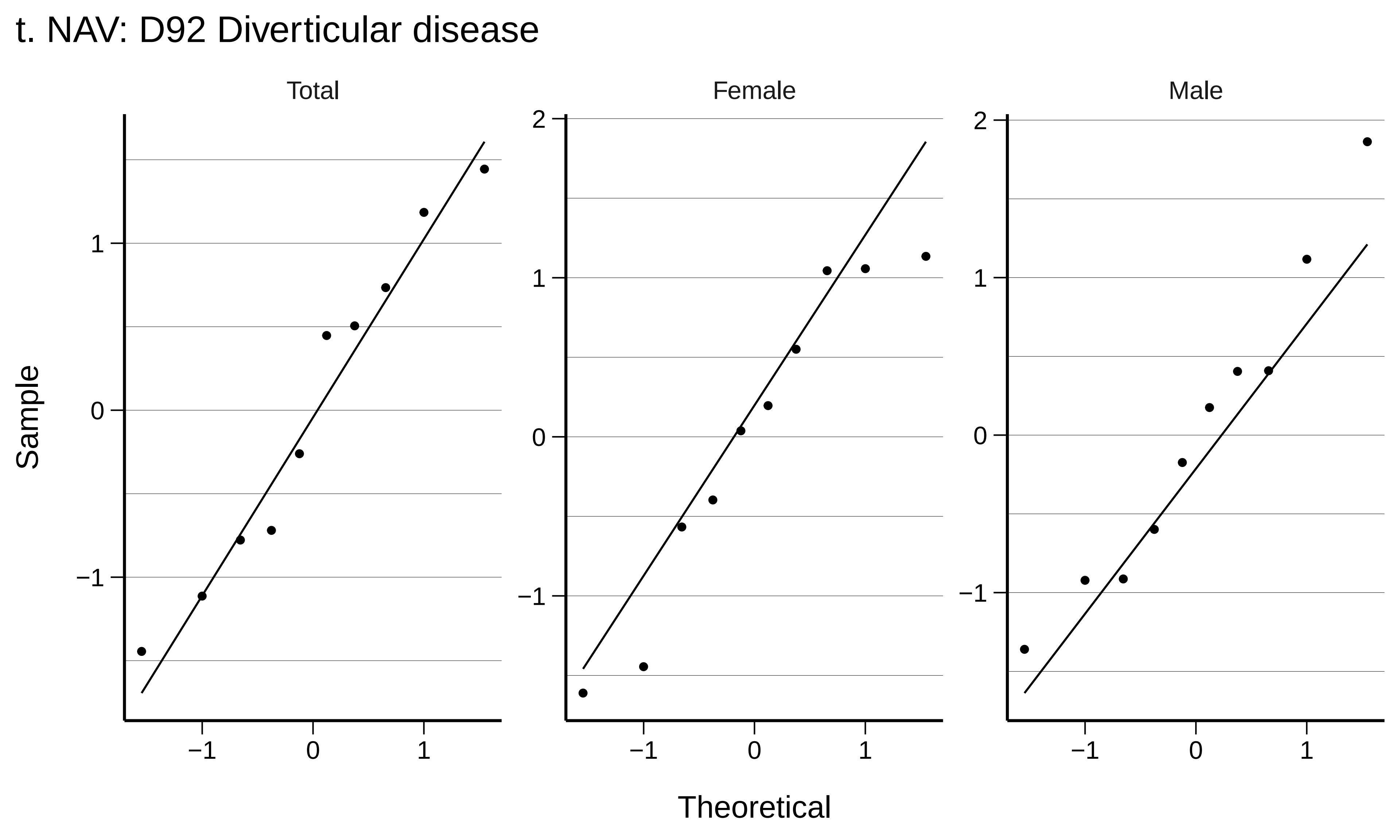

t. NAV: D92 Diverticular disease
Total
Female
Male
Sample
Theoretical
2
1
0
−1

u. NAV: Everything

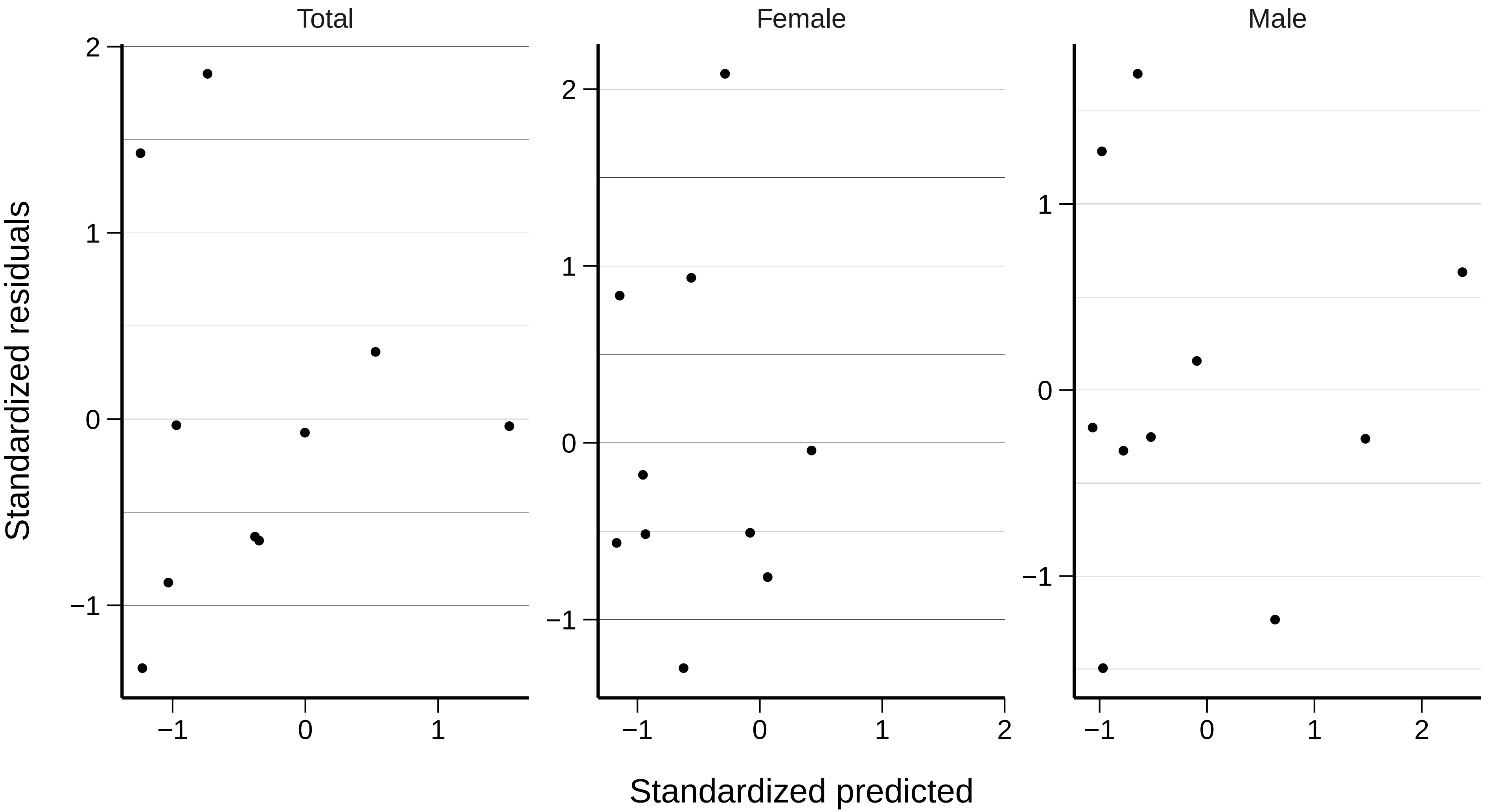

v. NAV: Everything

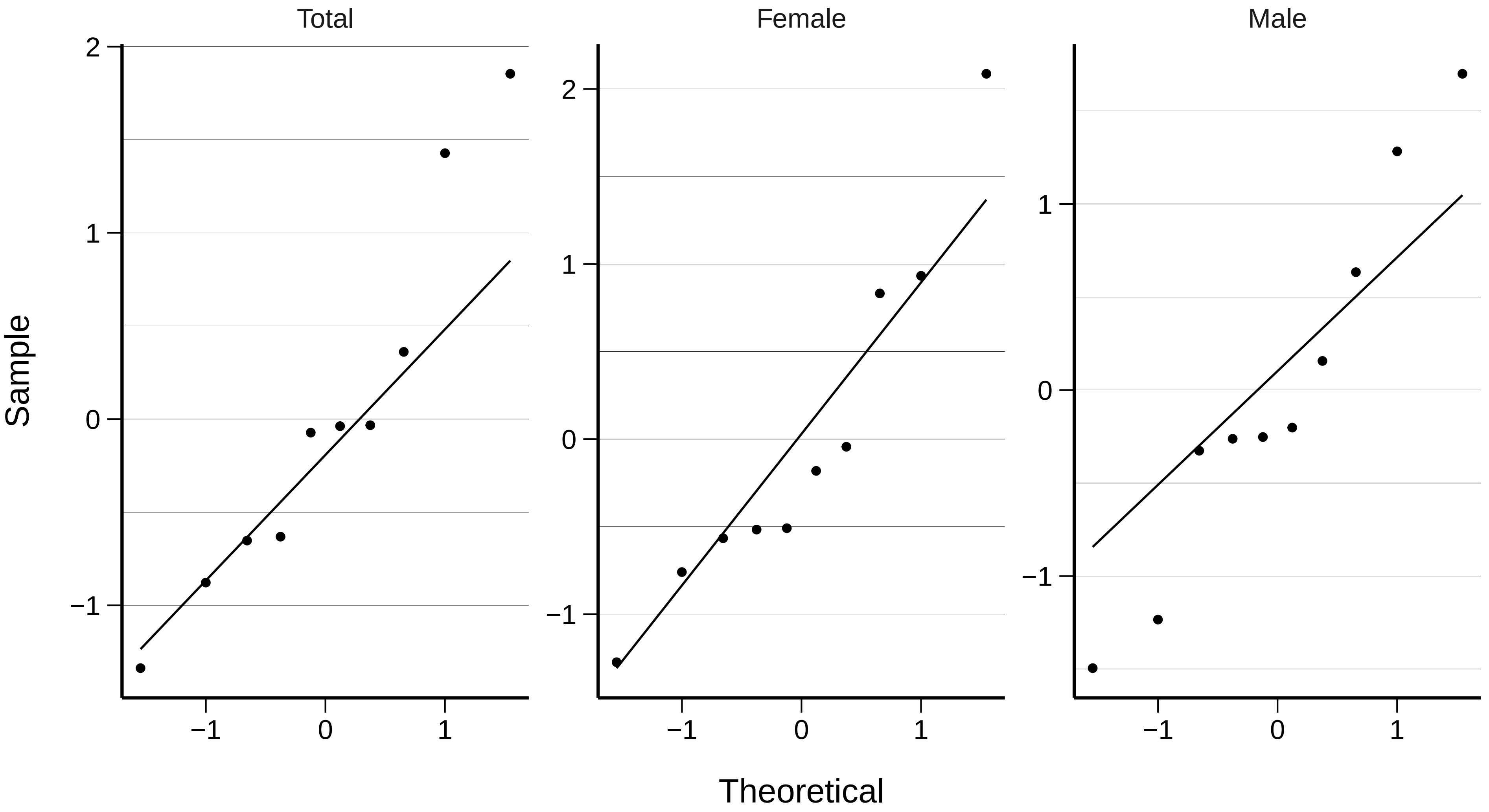

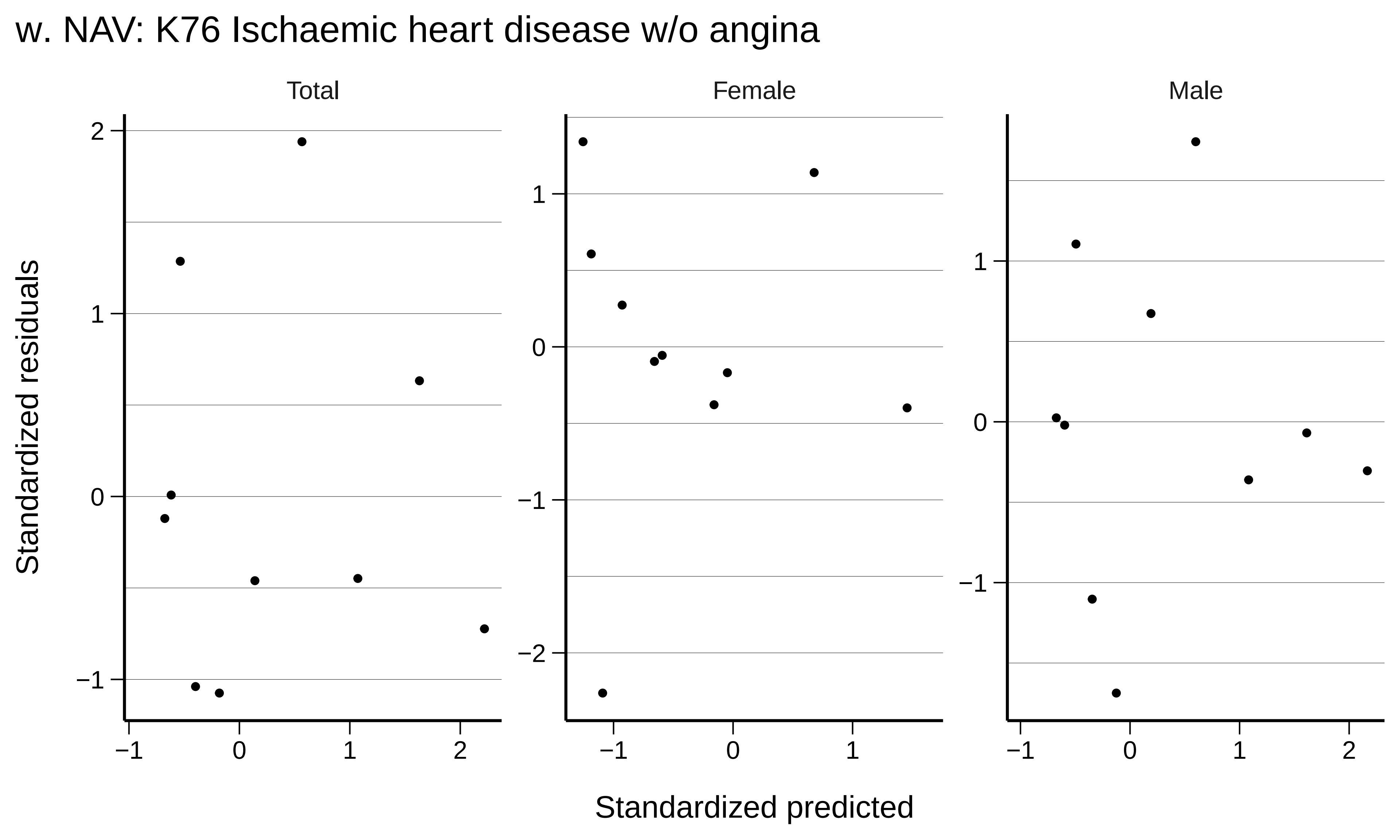

w. NAV: K76 Ischaemic heart disease w/o angina
Total
Female
Male
Standardized residuals
Standardized predicted
2
1
0
−1
−2

x. NAV: K76 Ischaemic heart disease w/o angina

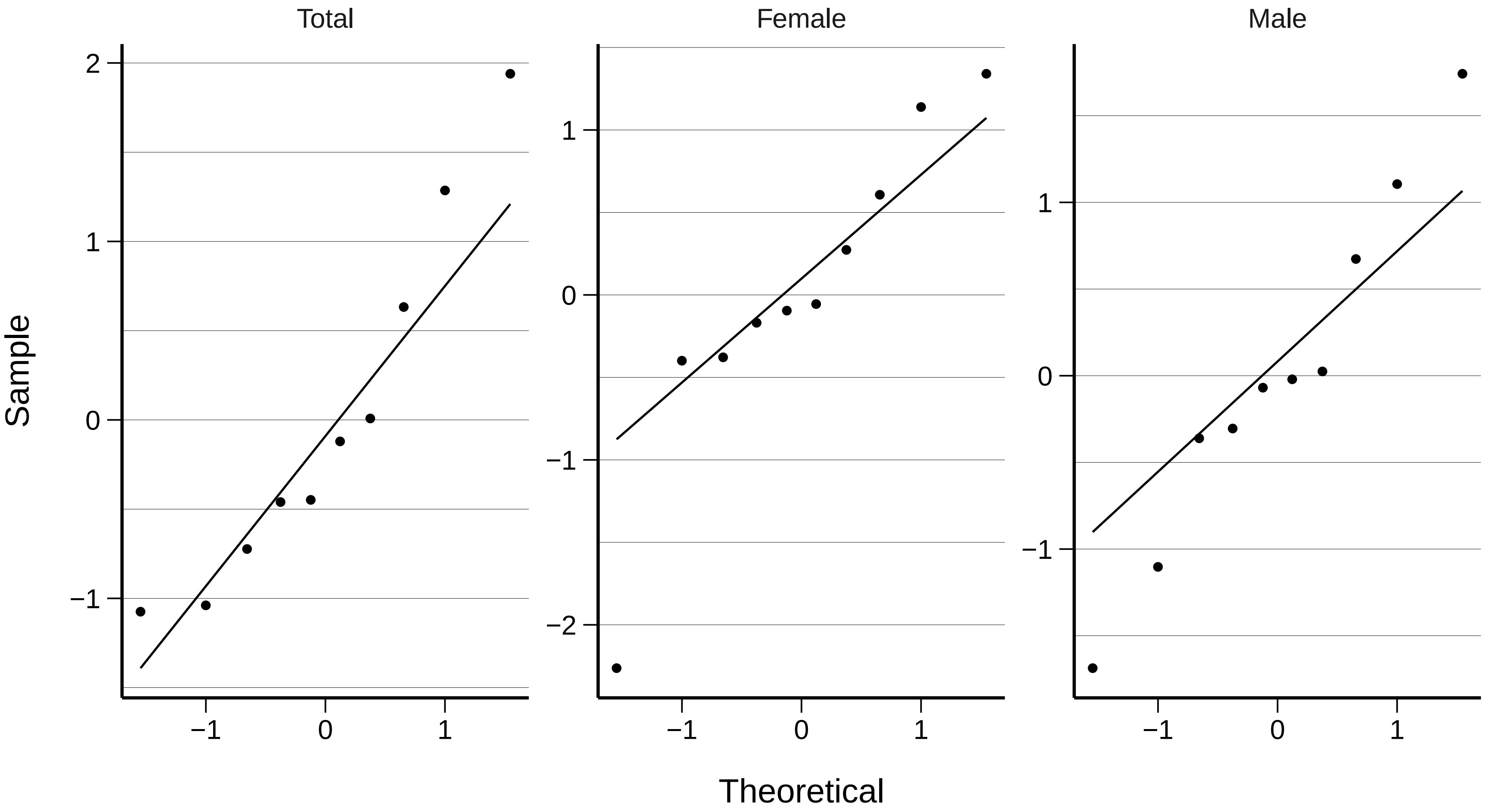

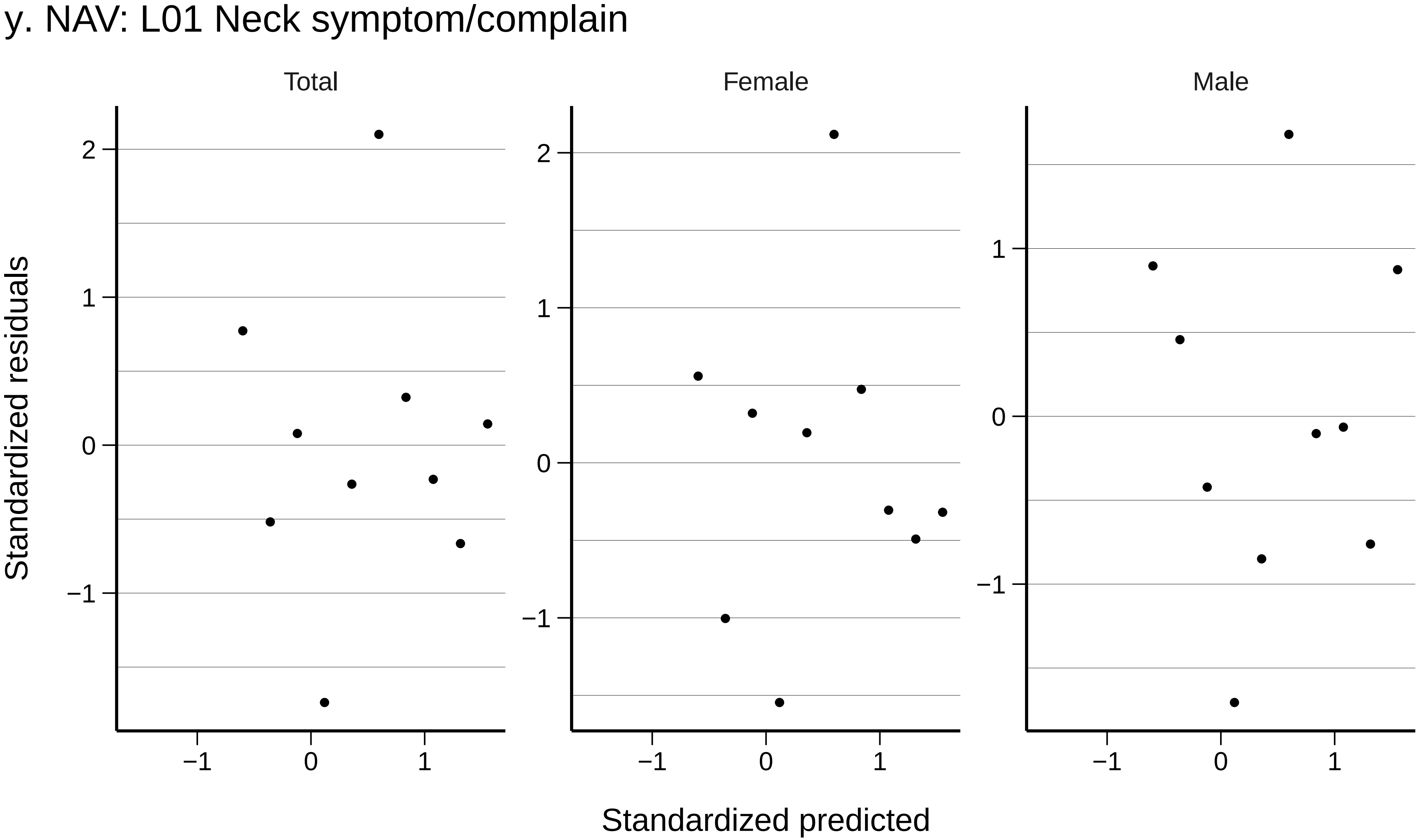
y. NAV: L01 Neck symptom/complain
Total
Female
Male
Standardized residuals
Standardized predicted
2
1
0
−1
−1
0
1

z. NAV: L01 Neck symptom/complain

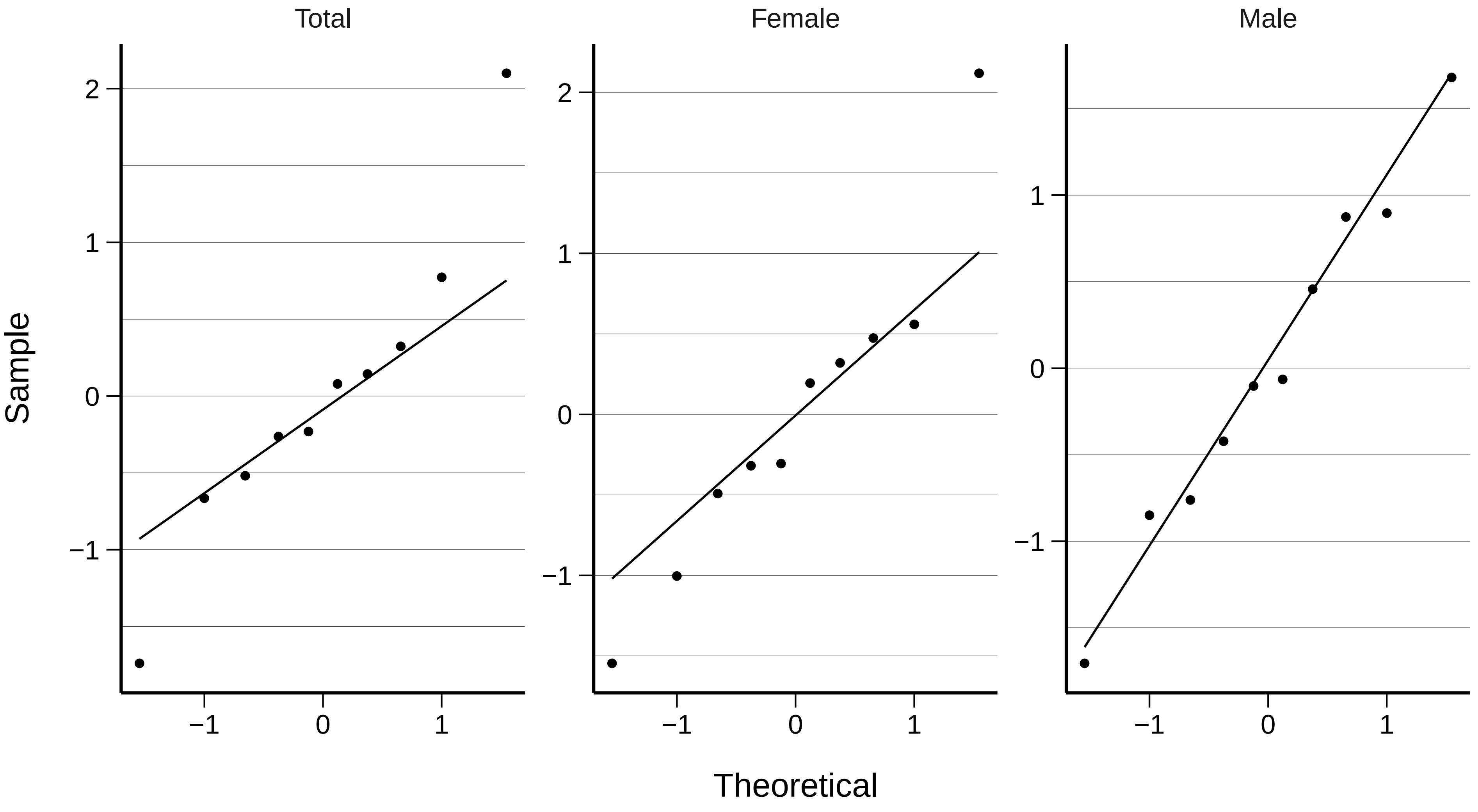

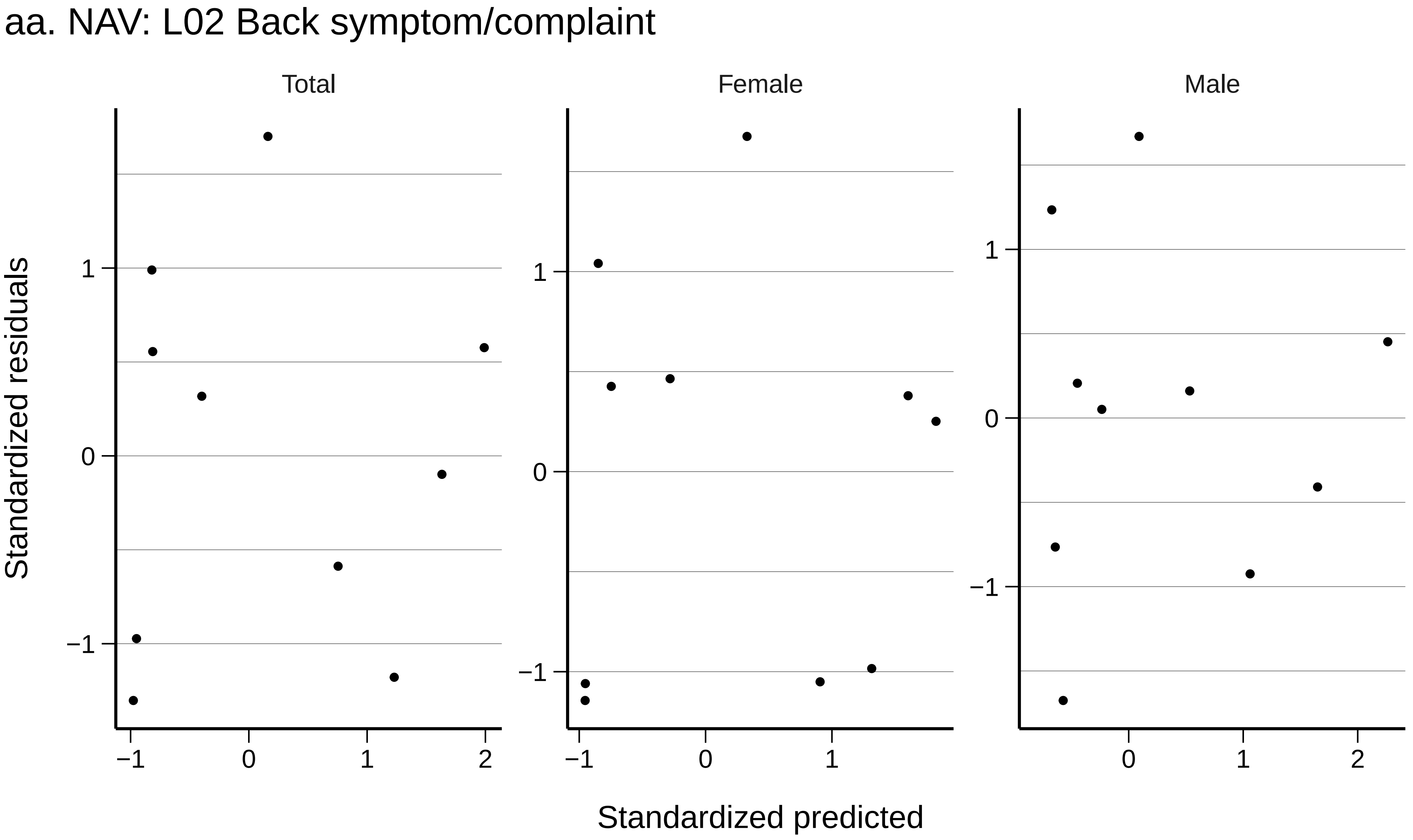
aa. NAV: L02 Back symptom/complaint
Total
Female
Male
Standardized residuals
Standardized predicted
1
0
−1
2

ab. NAV: L02 Back symptom/complaint

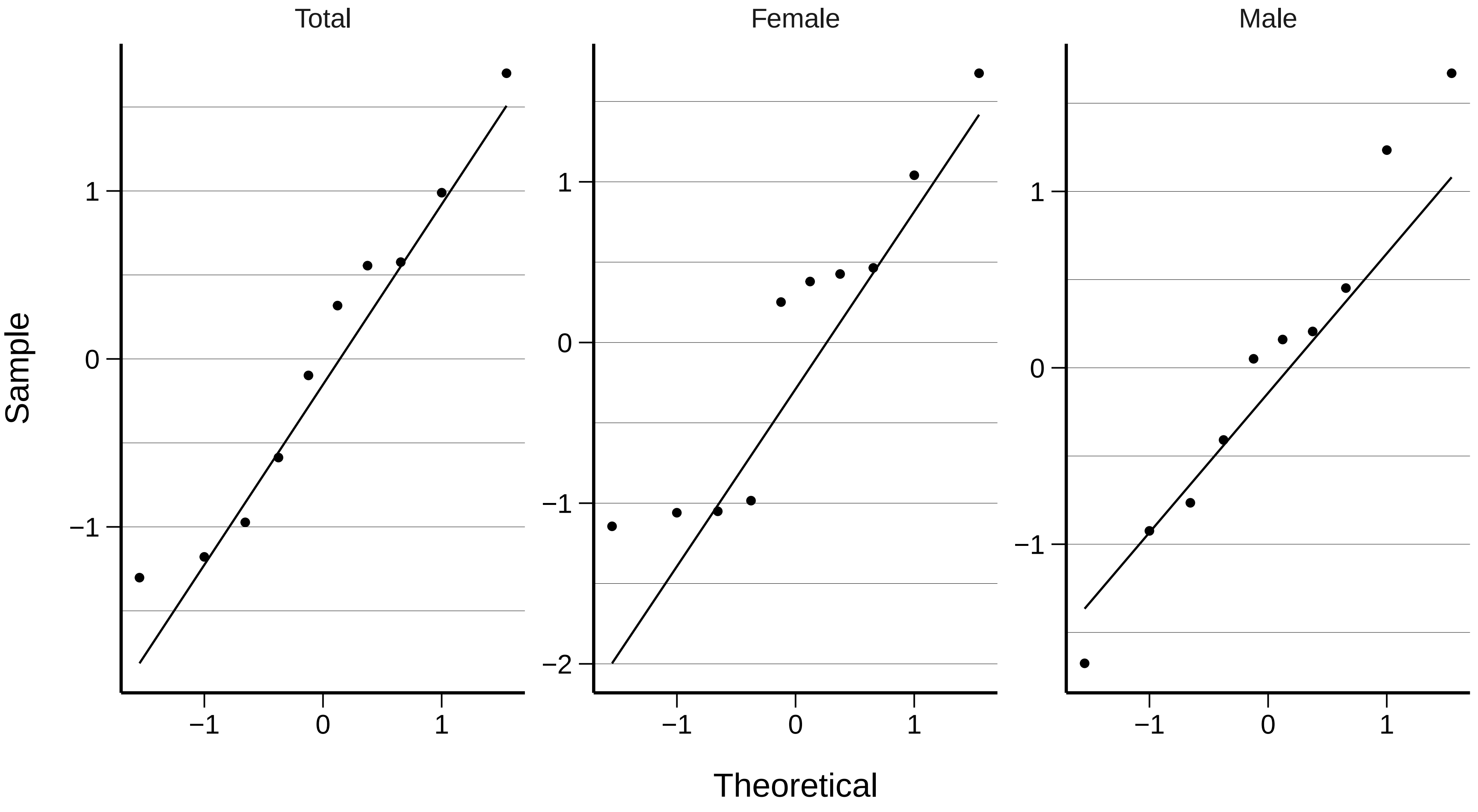

ac. NAV: L08 Shoulder symptom/complaint

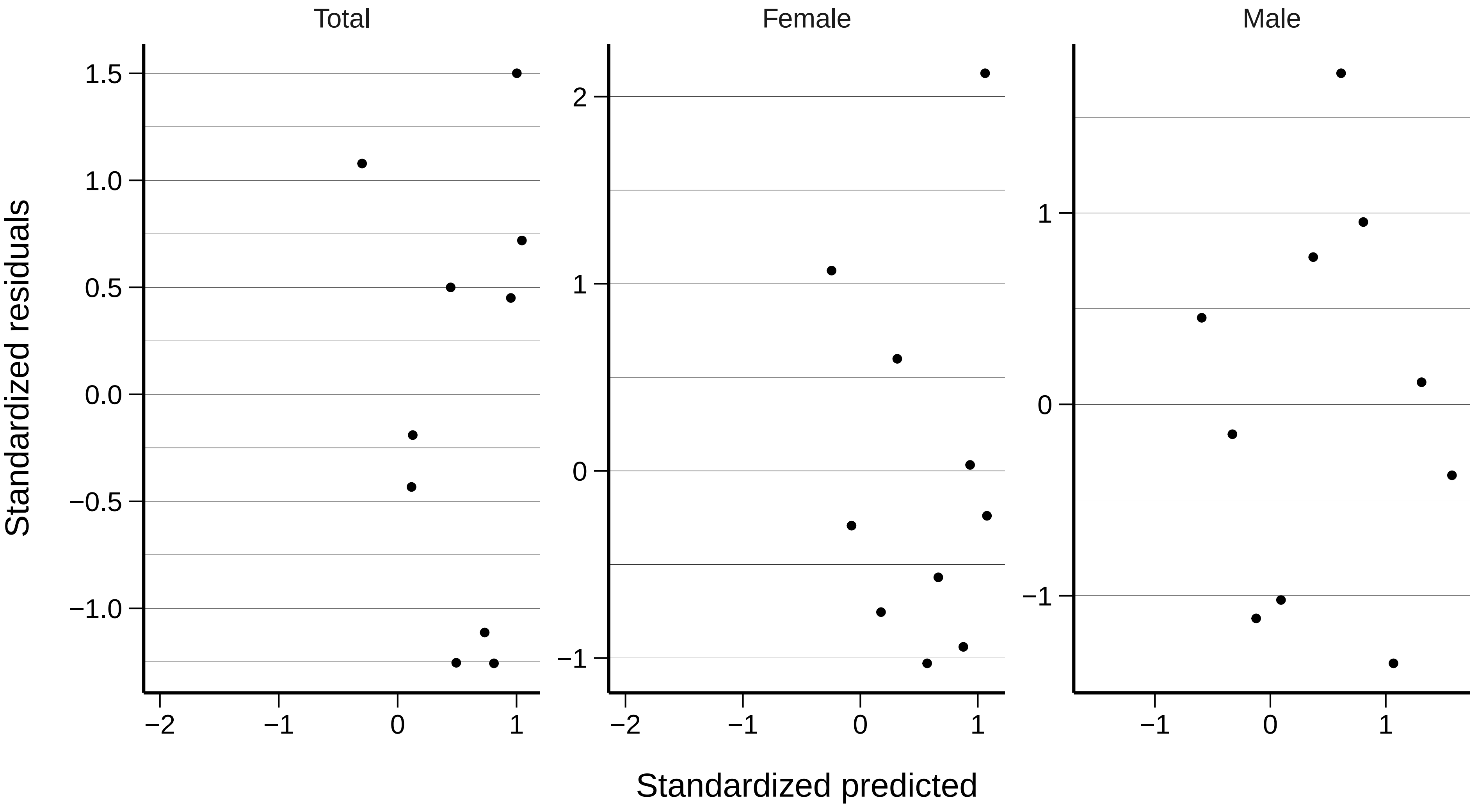

ad. NAV: L08 Shoulder symptom/complaint

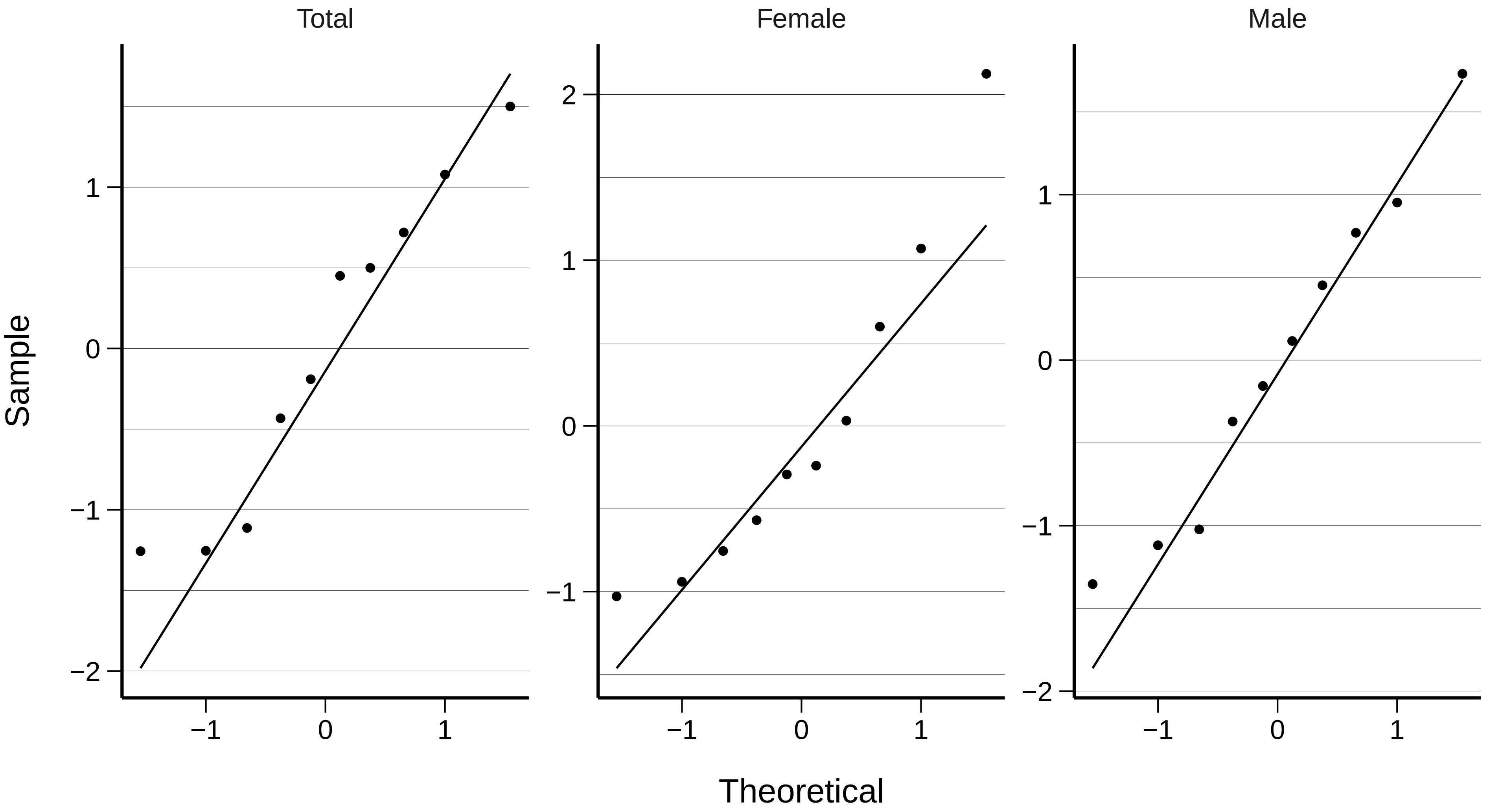

ae. NAV: L11 Wrist symptom/complaint

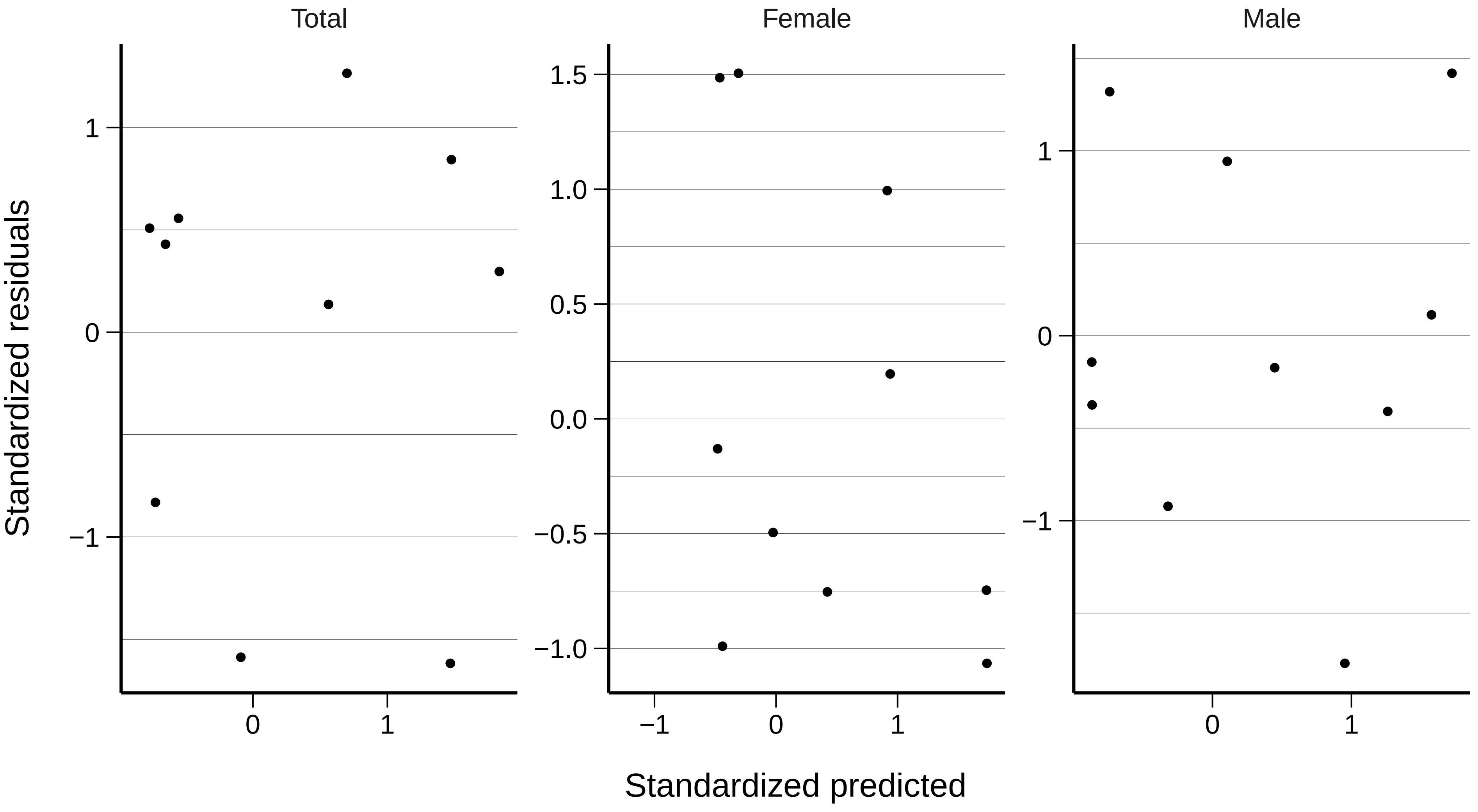

af. NAV: L11 Wrist symptom/complaint

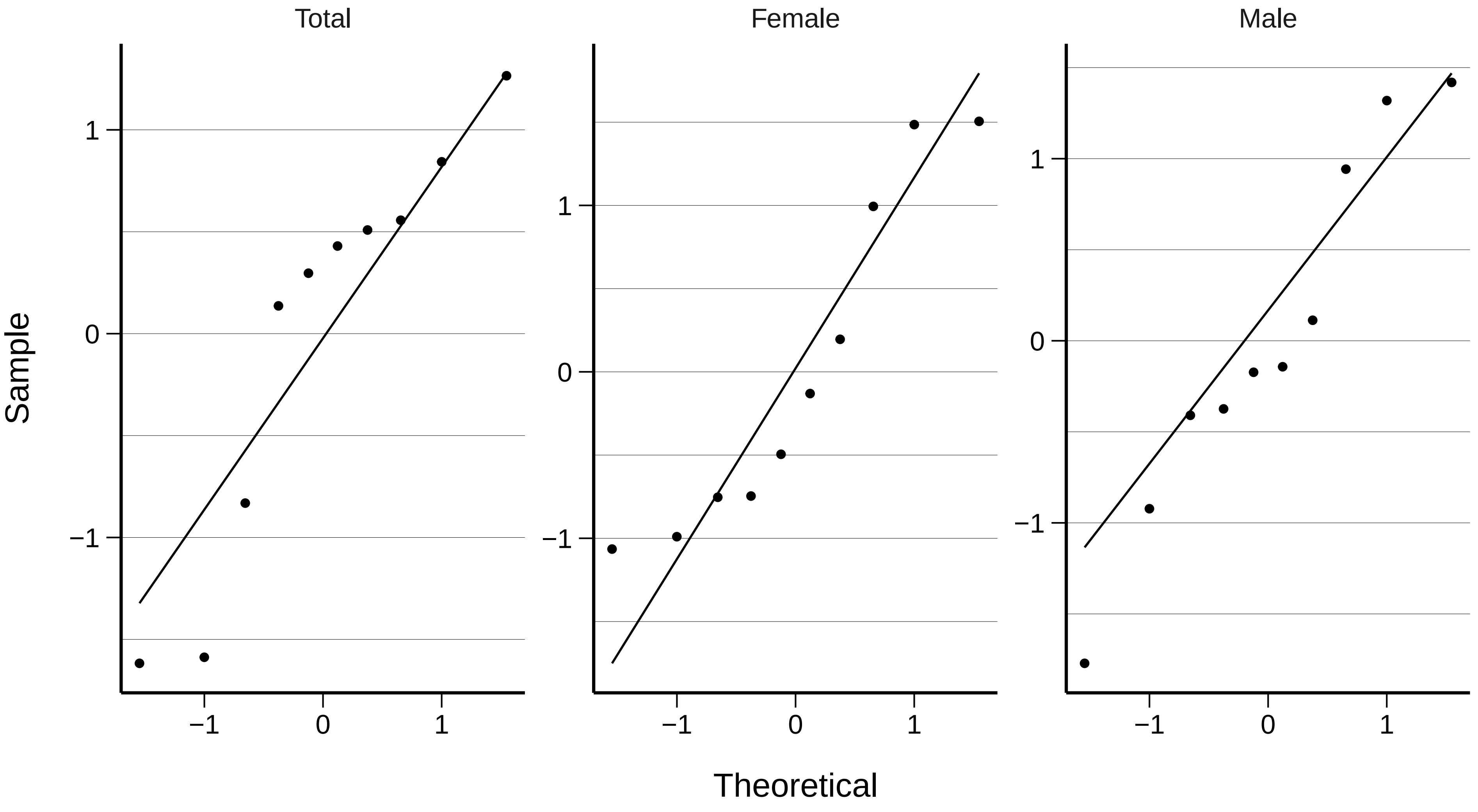

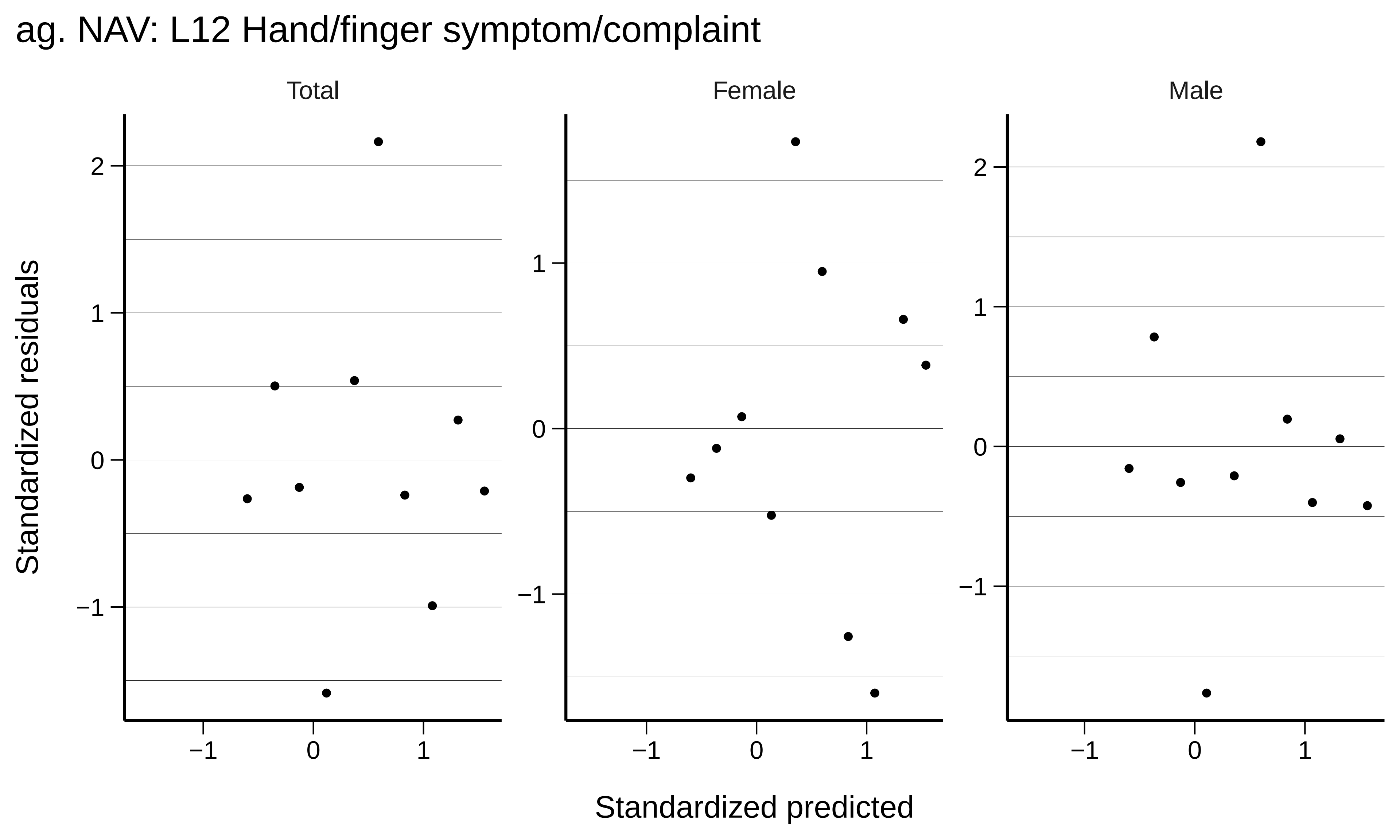
ag. NAV: L12 Hand/finger symptom/complaint
Total
Female
Male
Standardized residuals
Standardized predicted
2
1
0
−1
−1
0
1

ah. NAV: L12 Hand/finger symptom/complaint

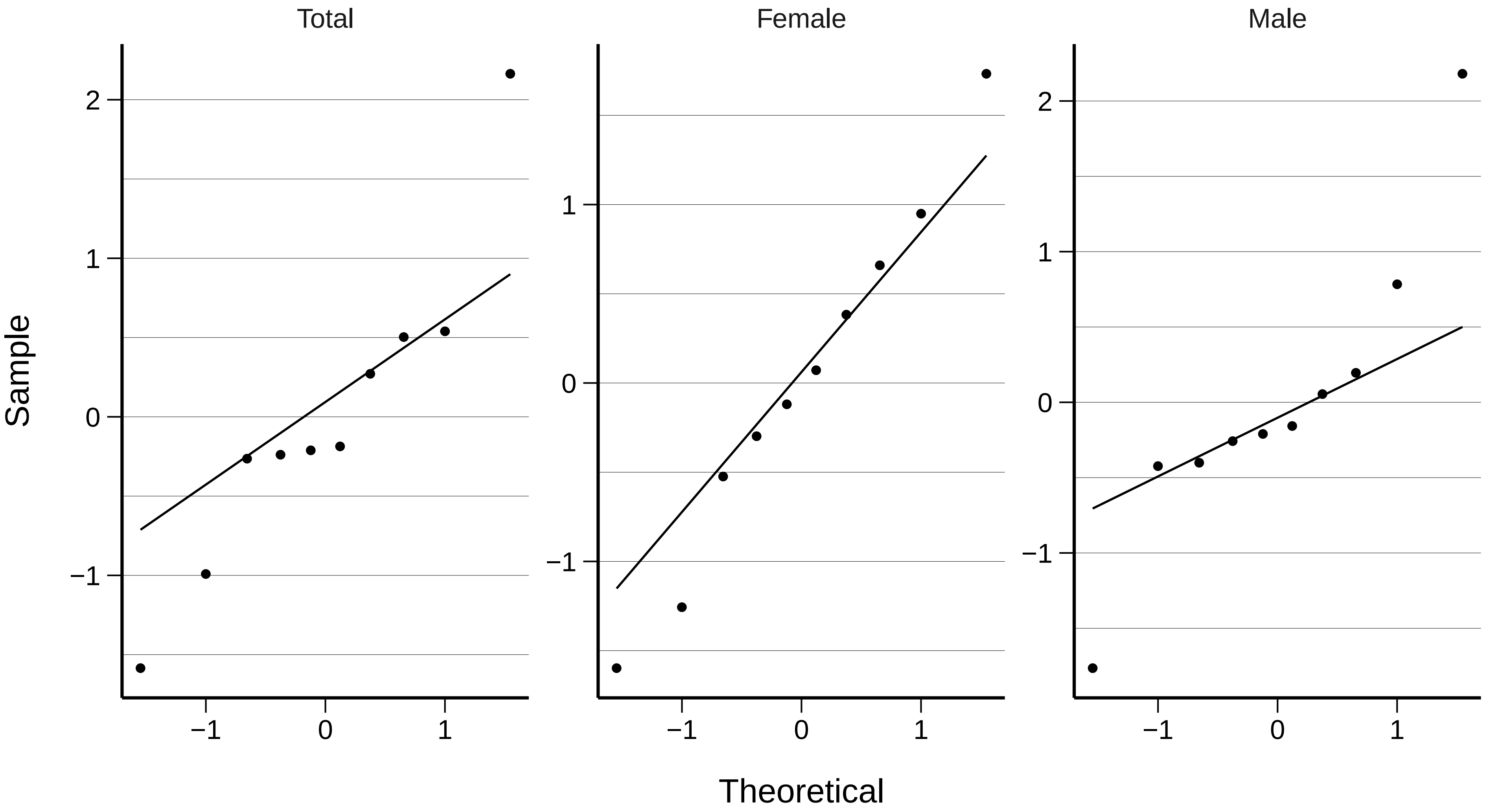

ai. NAV: L13 Hip symptom/complaint

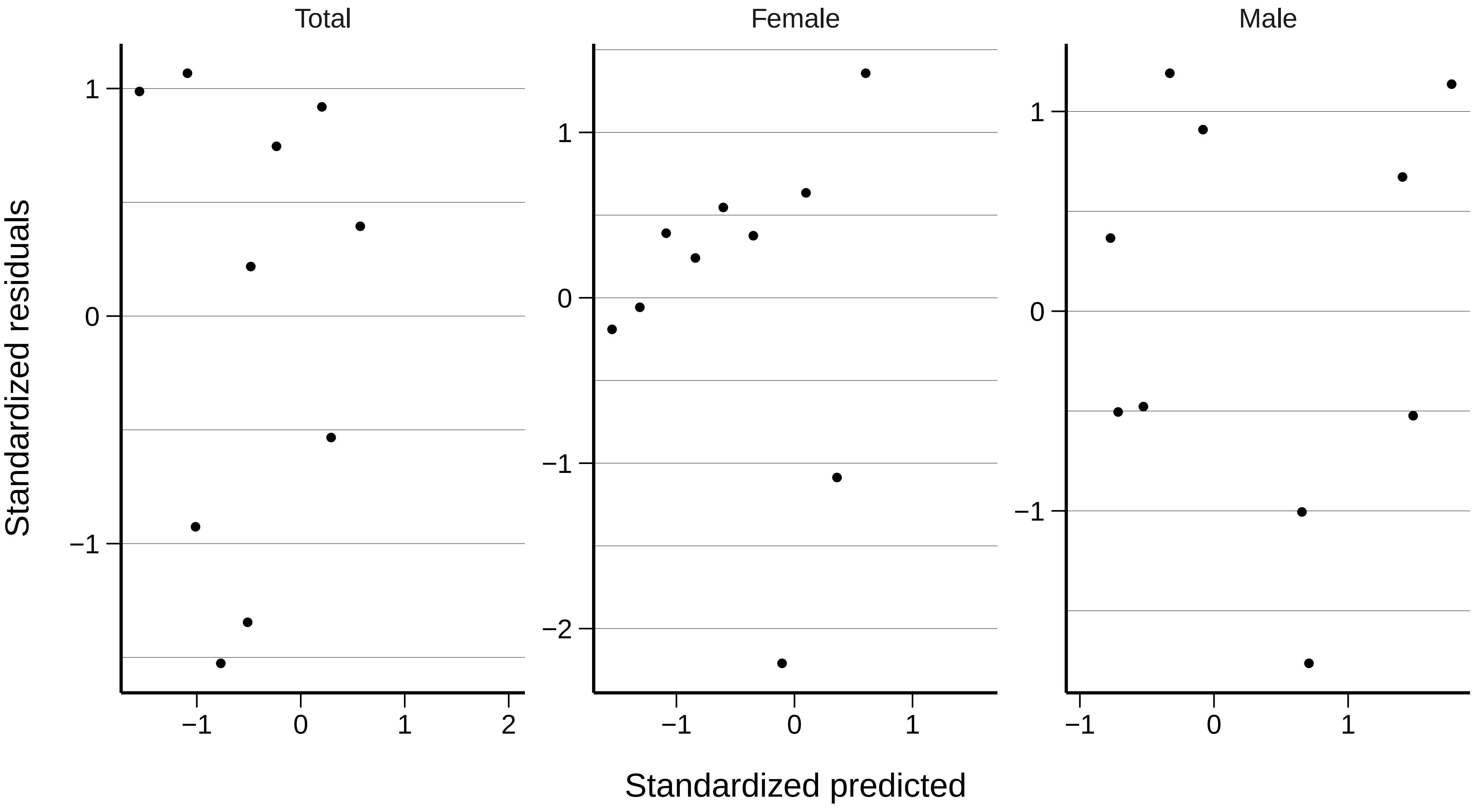

aj. NAV: L13 Hip symptom/complaint

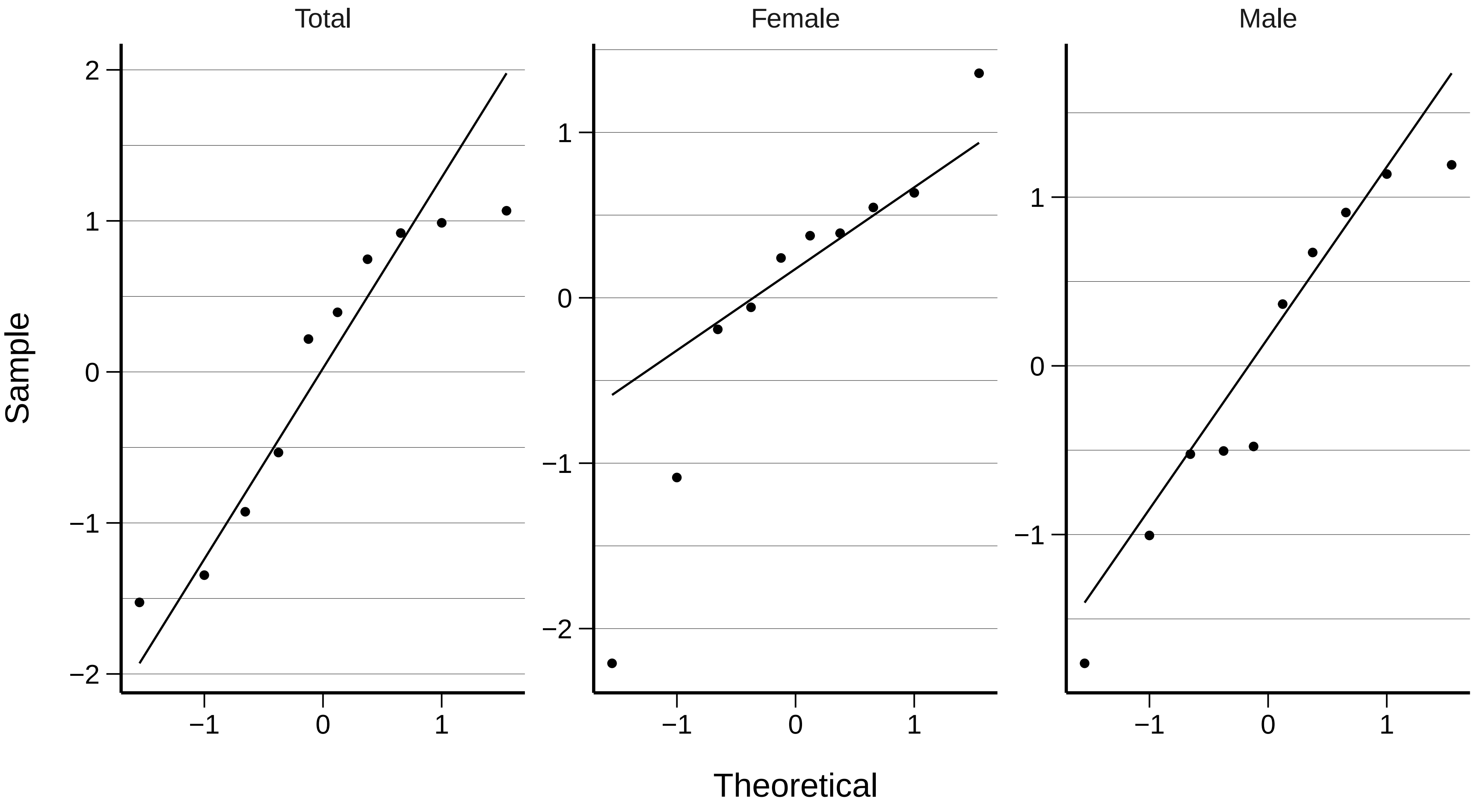

ak. NAV: L16 Ankle symptom/complaint

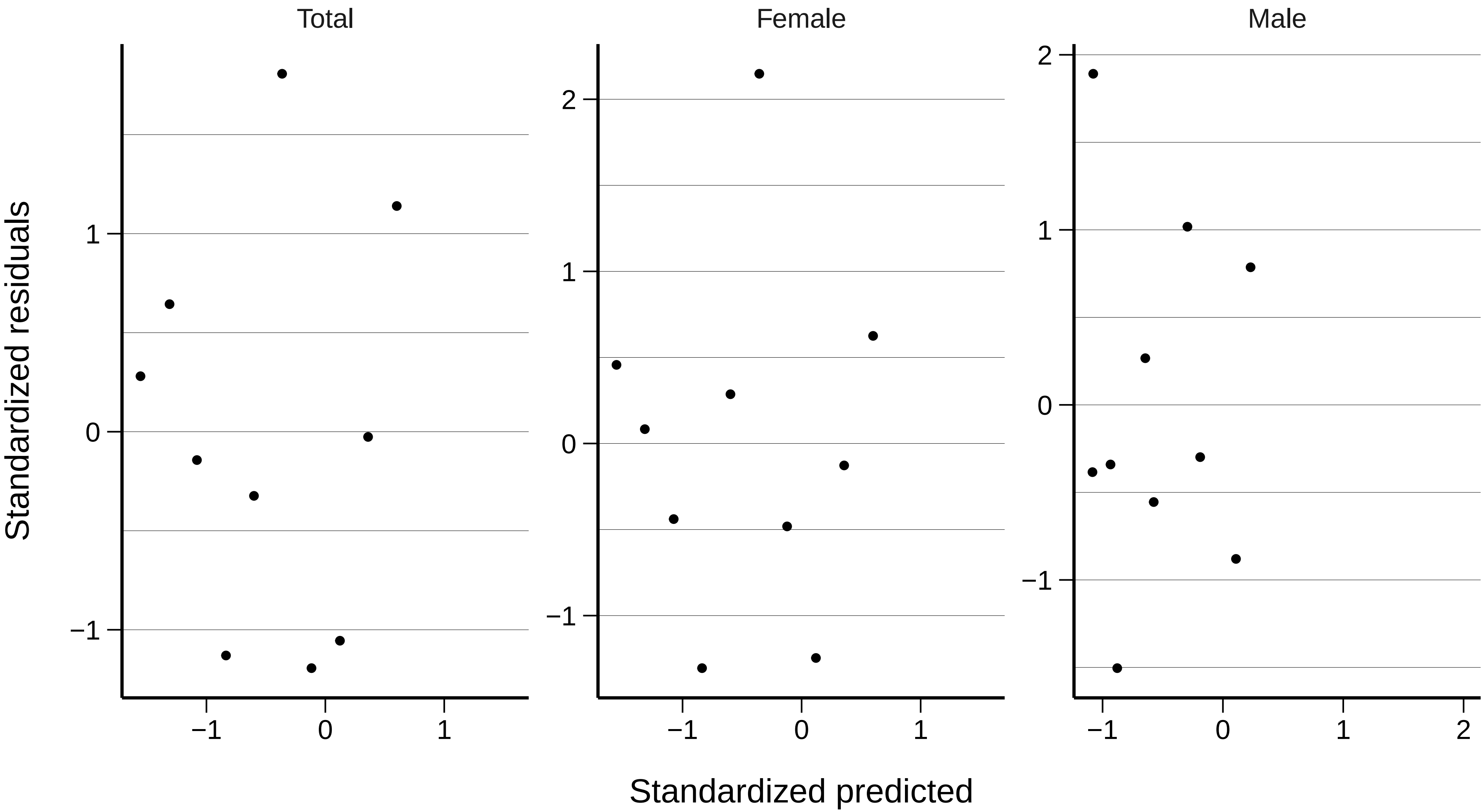

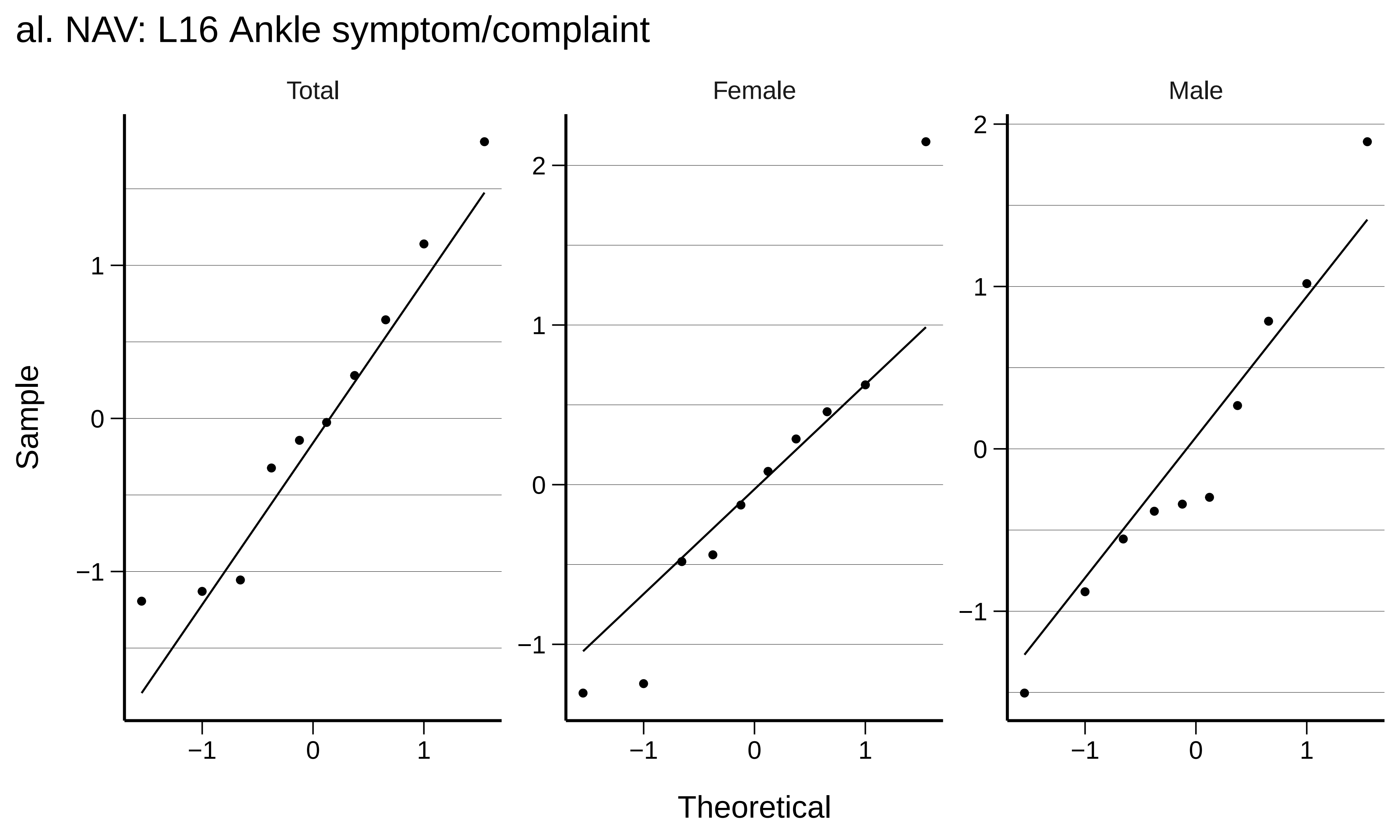
al. NAV: L16 Ankle symptom/complaint
Total
Female
Male
Sample
Theoretical
2
1
0
−1

am. NAV: L19 Muscle symptom/complaint NOS

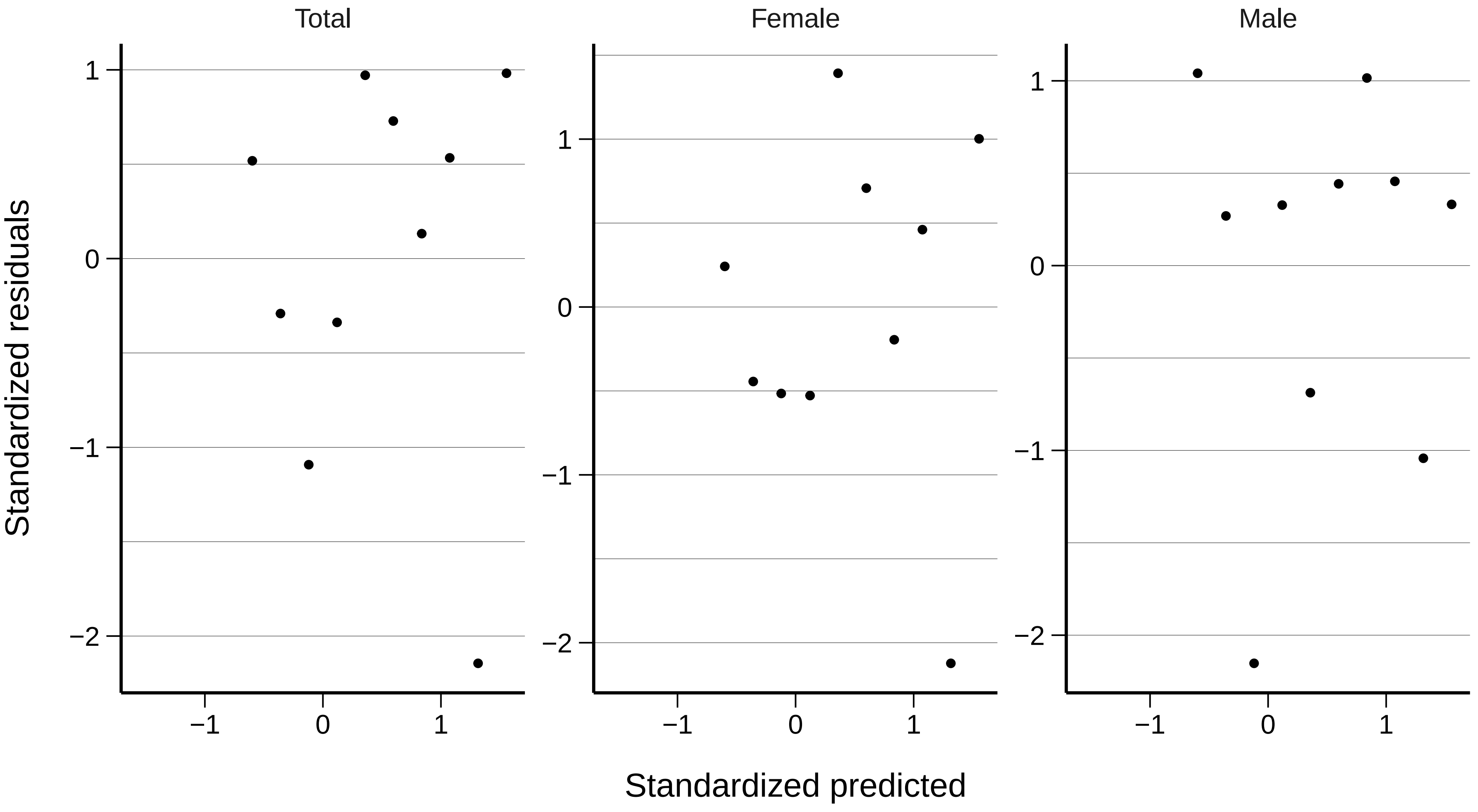

an. NAV: L19 Muscle symptom/complaint NOS

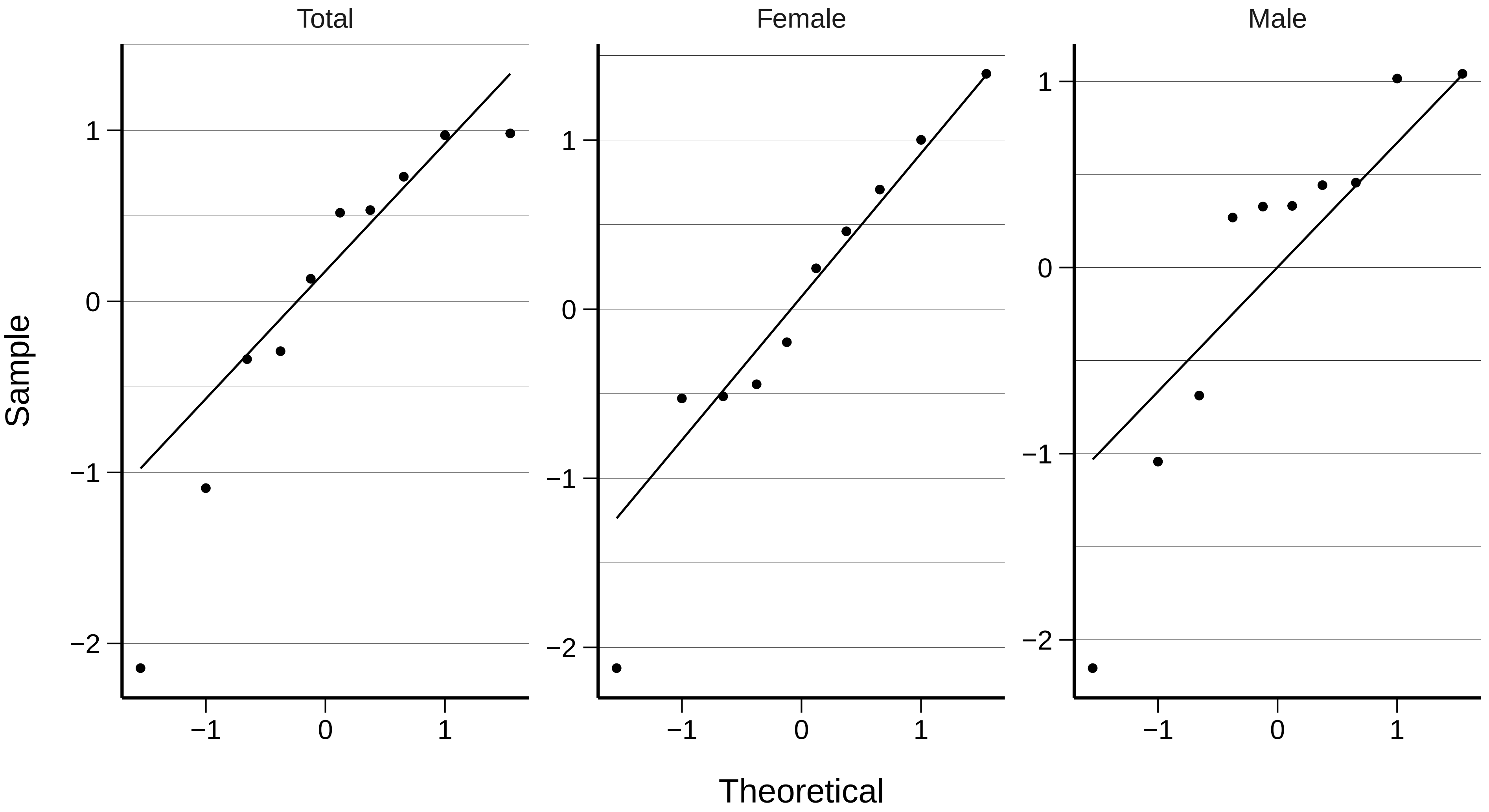

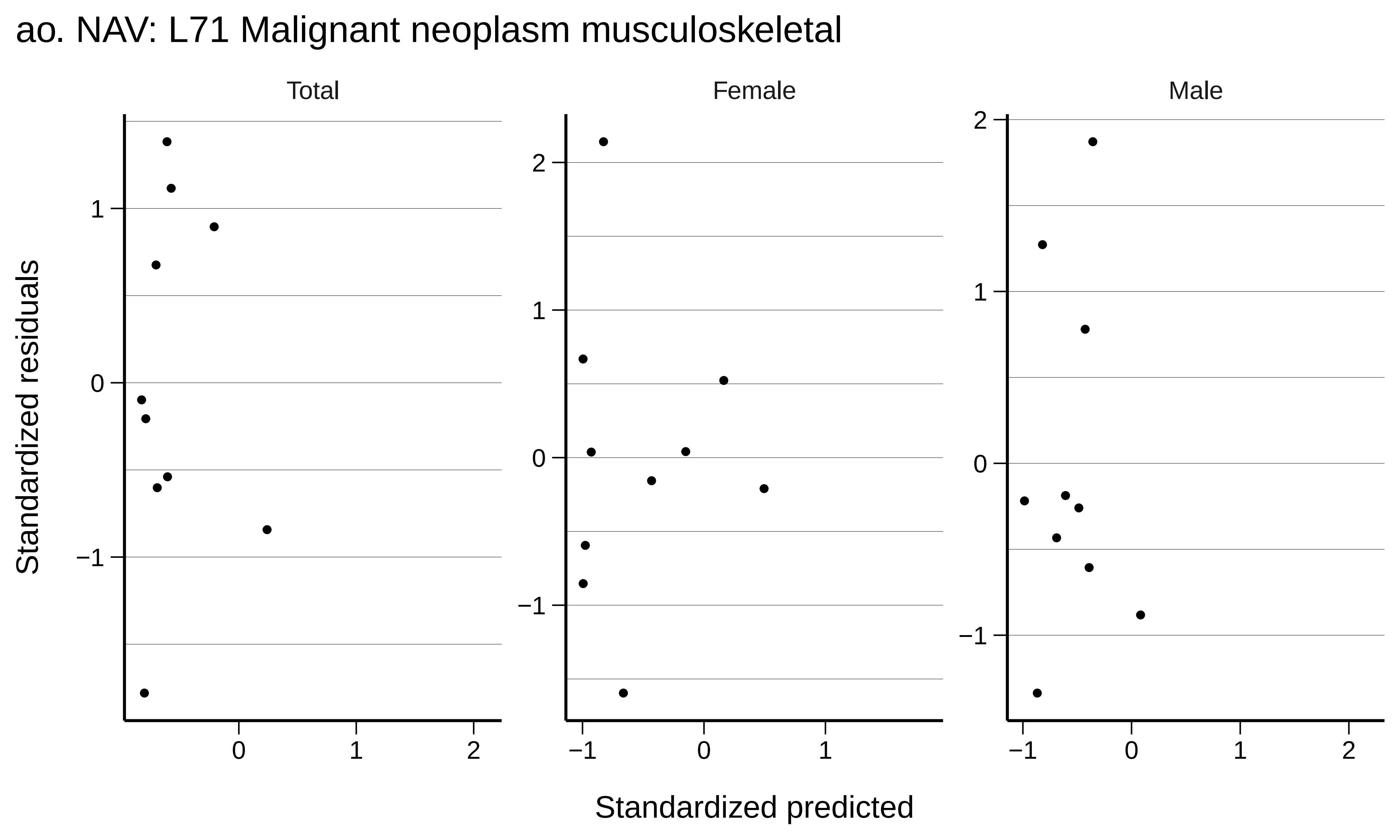
ao. NAV: L71 Malignant neoplasm musculoskeletal
Total
Female
Male
Standardized residuals
Standardized predicted
2
1
0
−1

ap. NAV: L71 Malignant neoplasm musculoskeletal

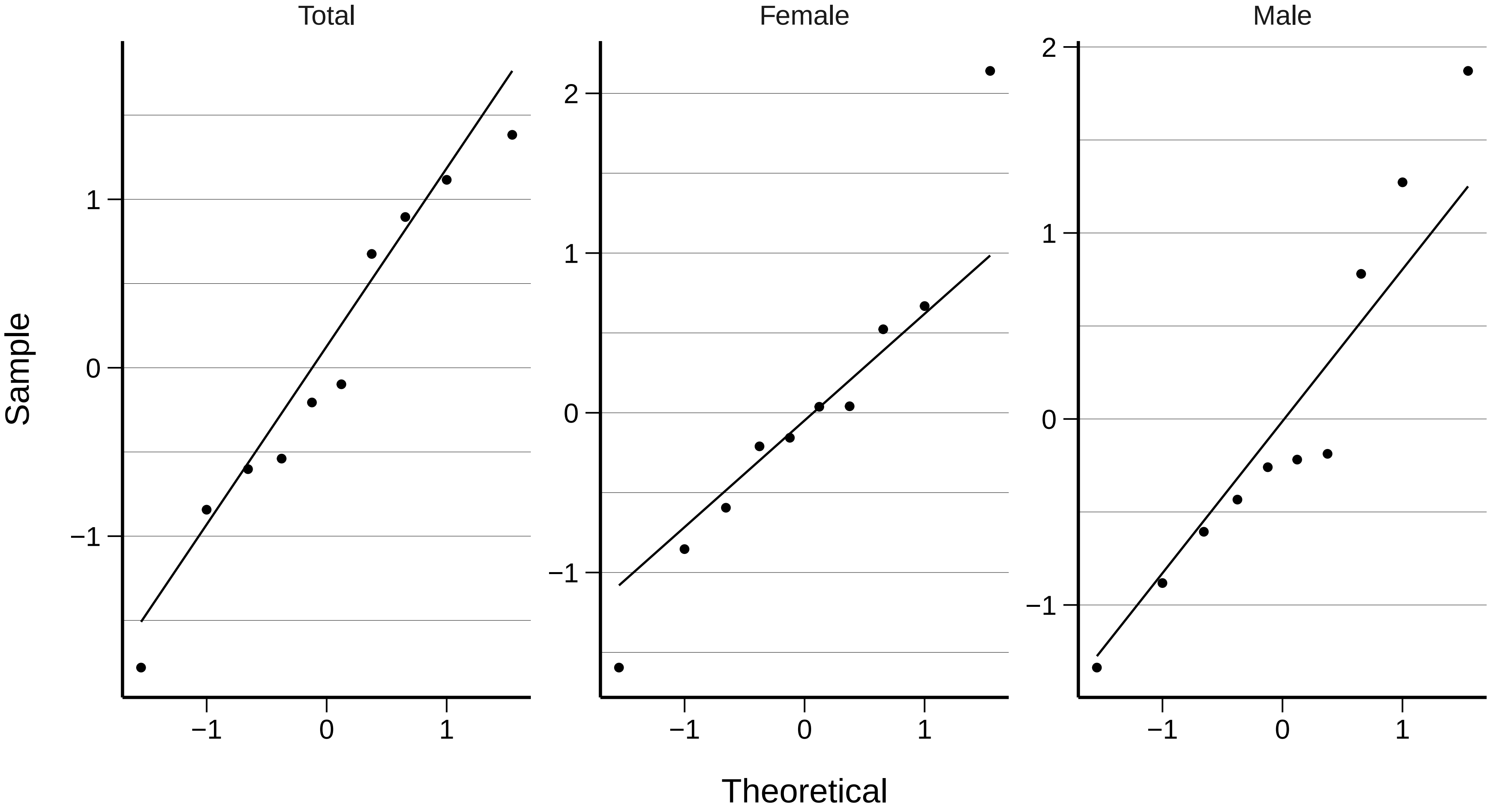

aq. NAV: L81 Injury musculoskeletal NOS

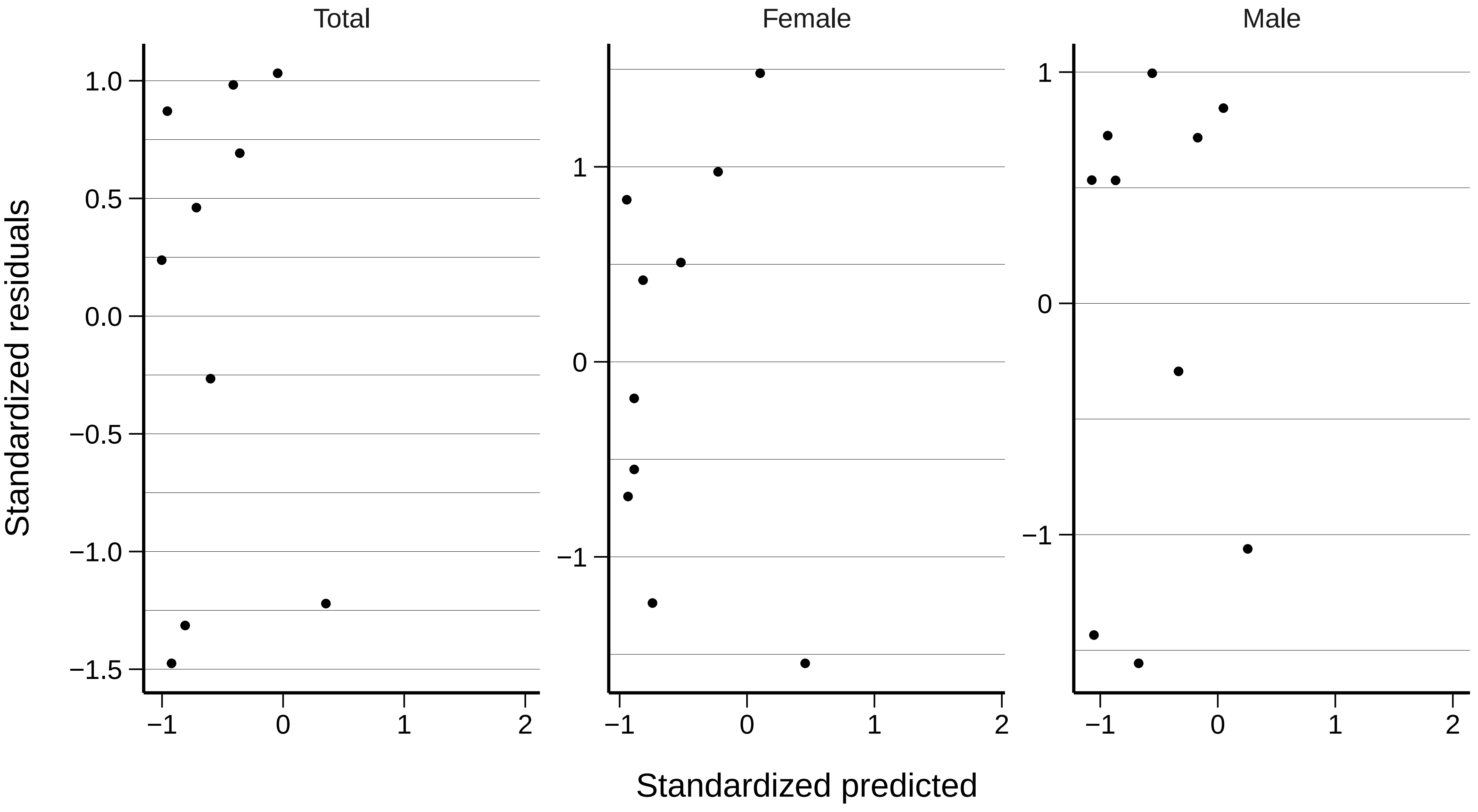

ar. NAV: L81 Injury musculoskeletal NOS

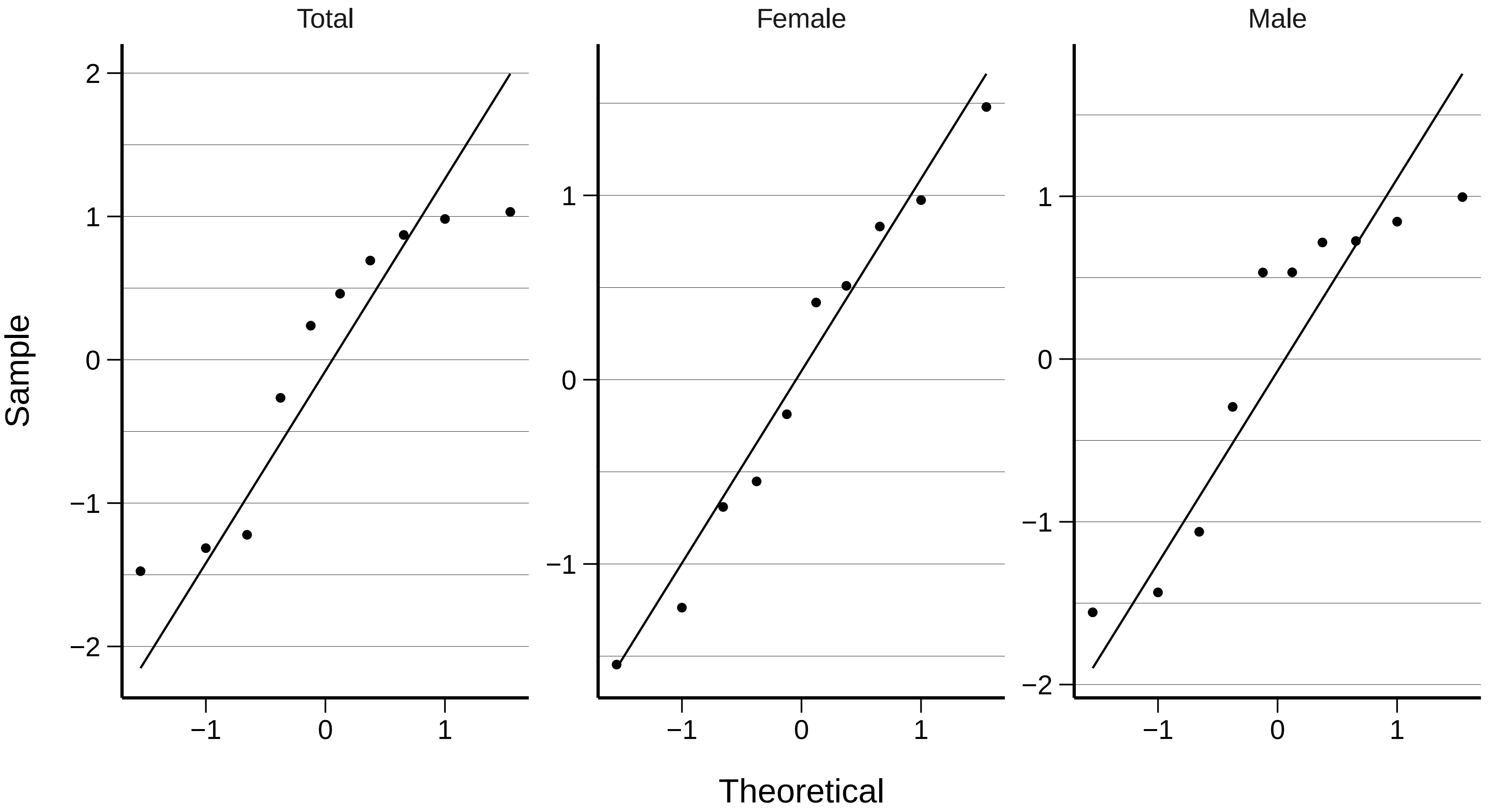

as. NAV: N01 Headache

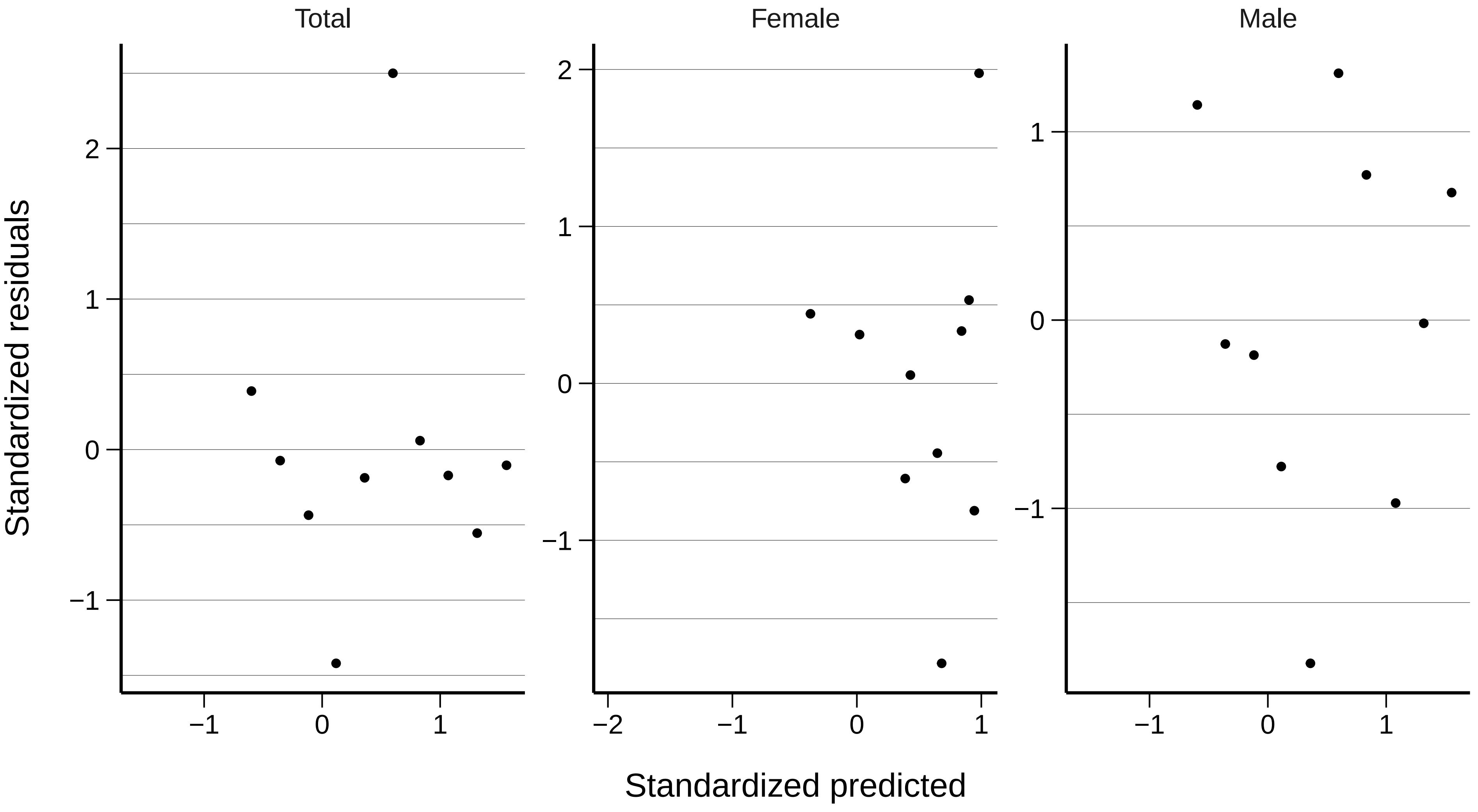

at. NAV: N01 Headache

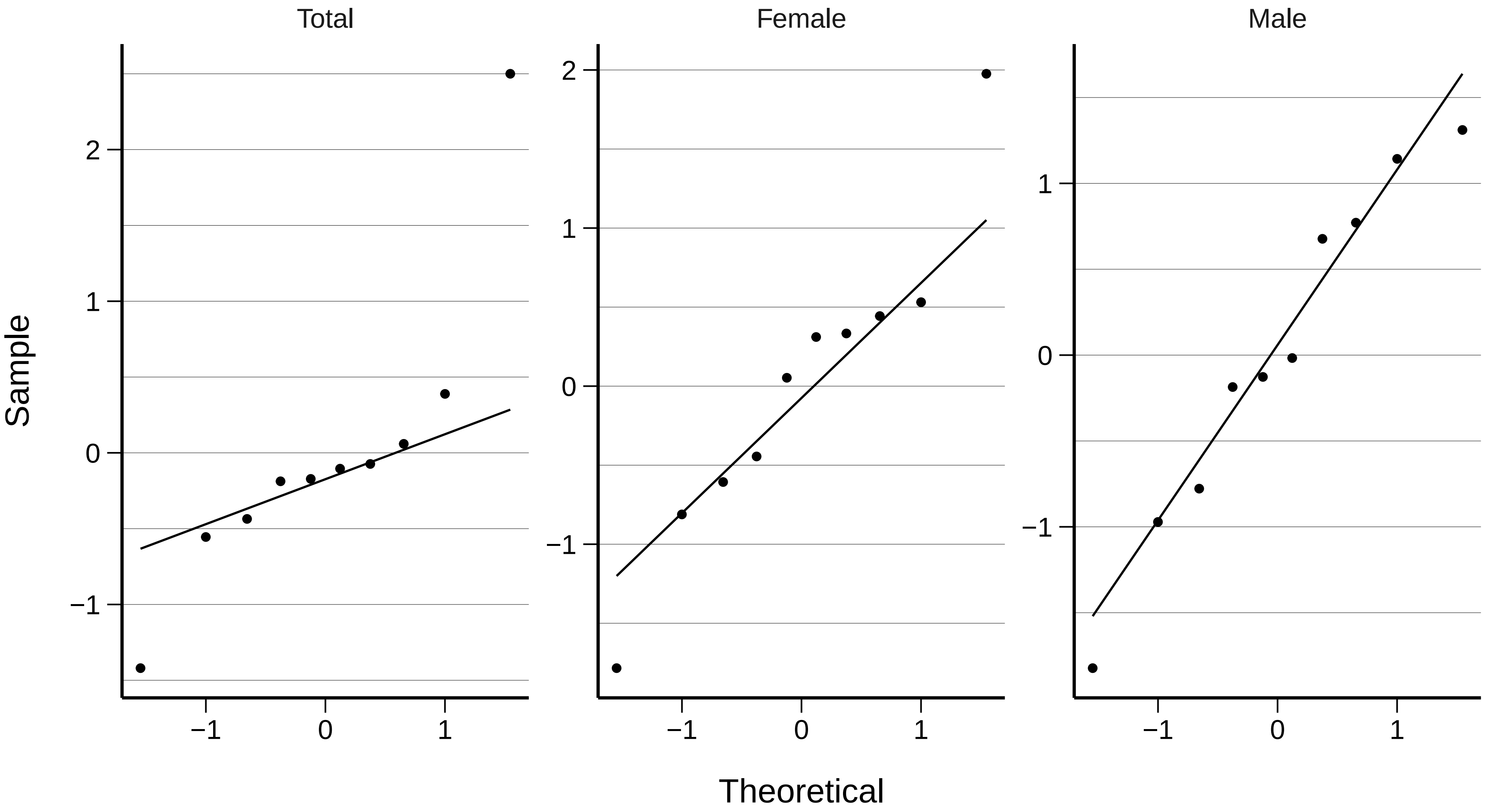

au. NAV: N79 Concussion

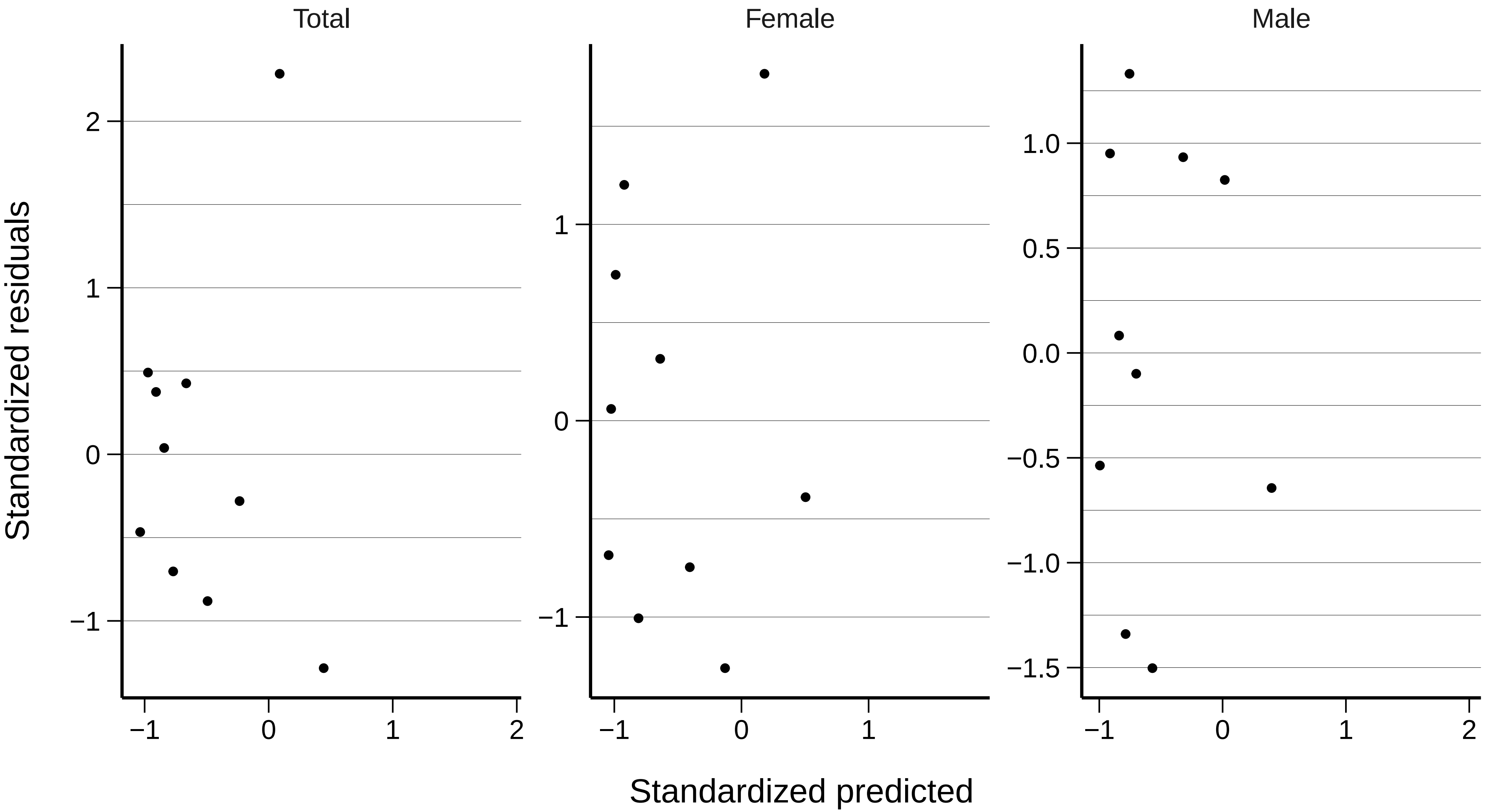

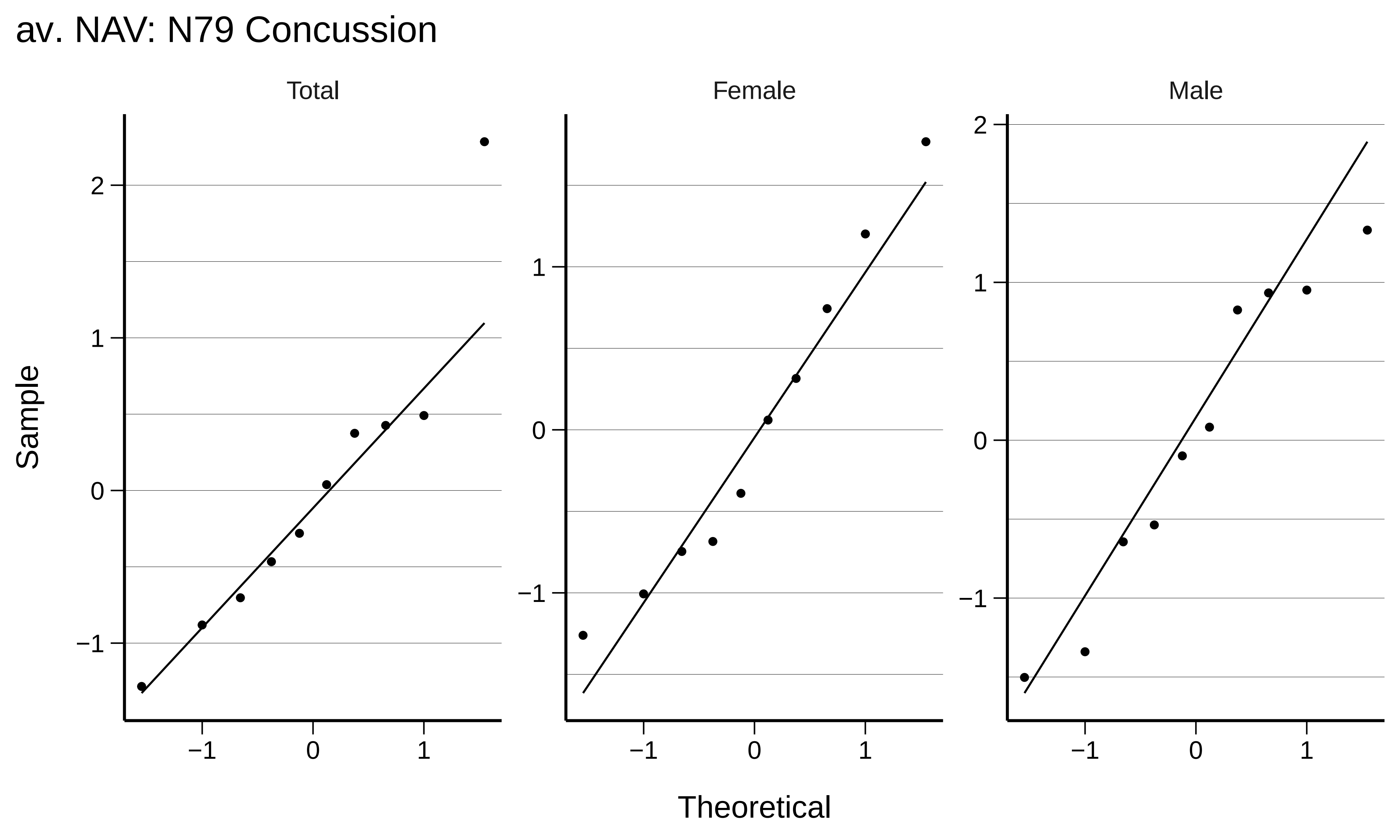
av. NAV: N79 Concussion
Total
Female
Male
Sample
Theoretical
2
1
0
−1
2
1
0
−1
−1
0
1

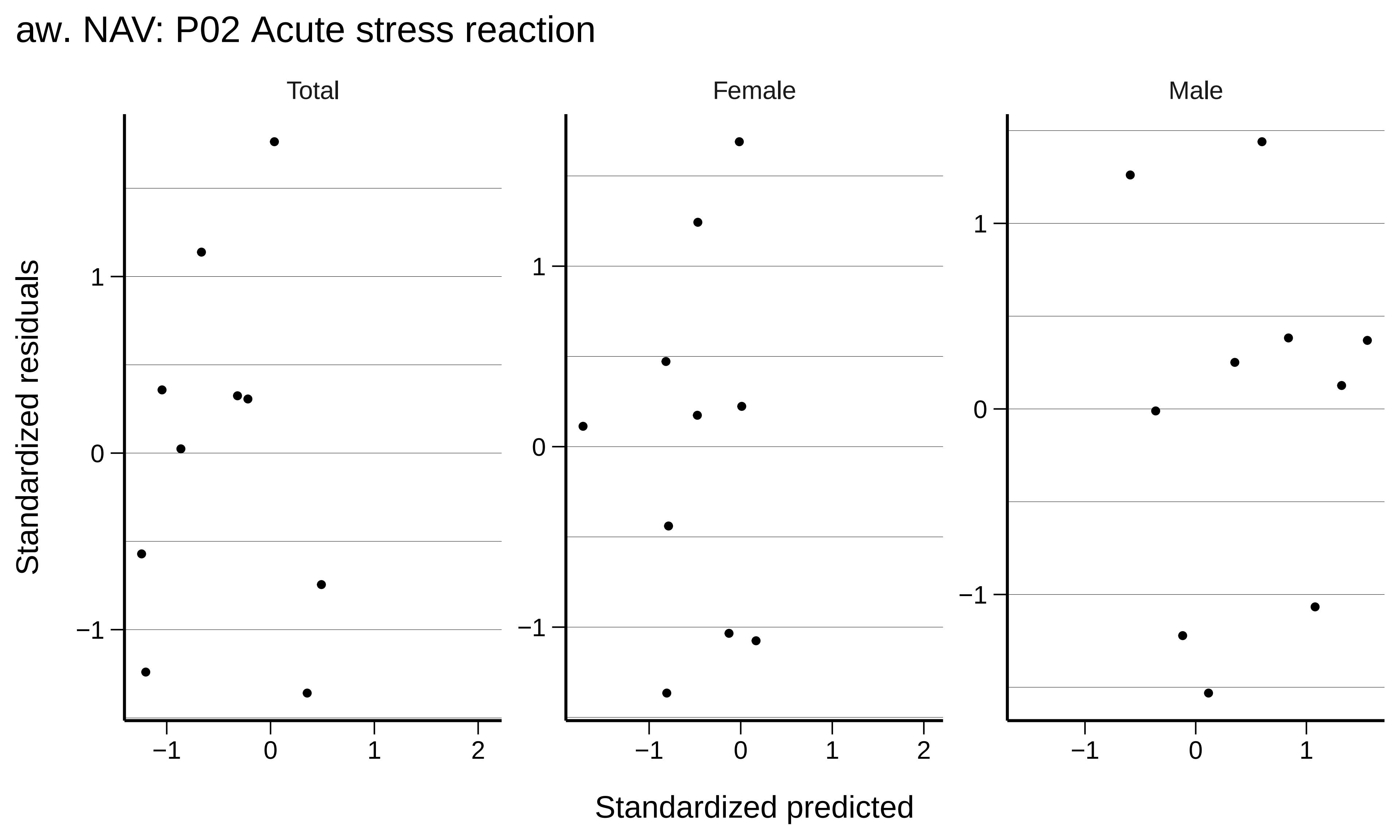
aw. NAV: P02 Acute stress reaction
Total
Female
Male
Standardized residuals
Standardized predicted
1
0
−1
−1
0
1
2

ax. NAV: P02 Acute stress reaction

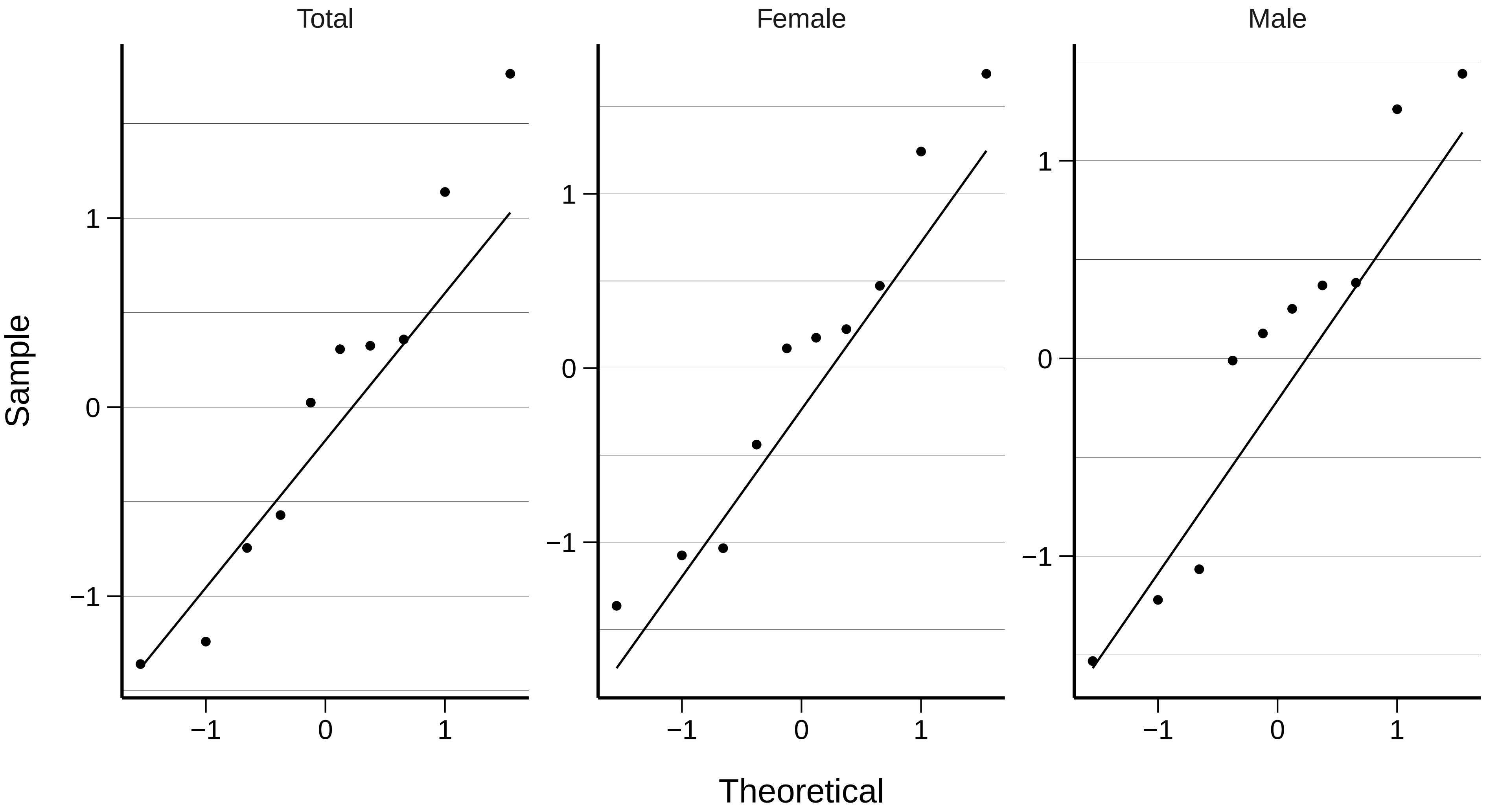

ay. NAV: P03 Feeling depressed

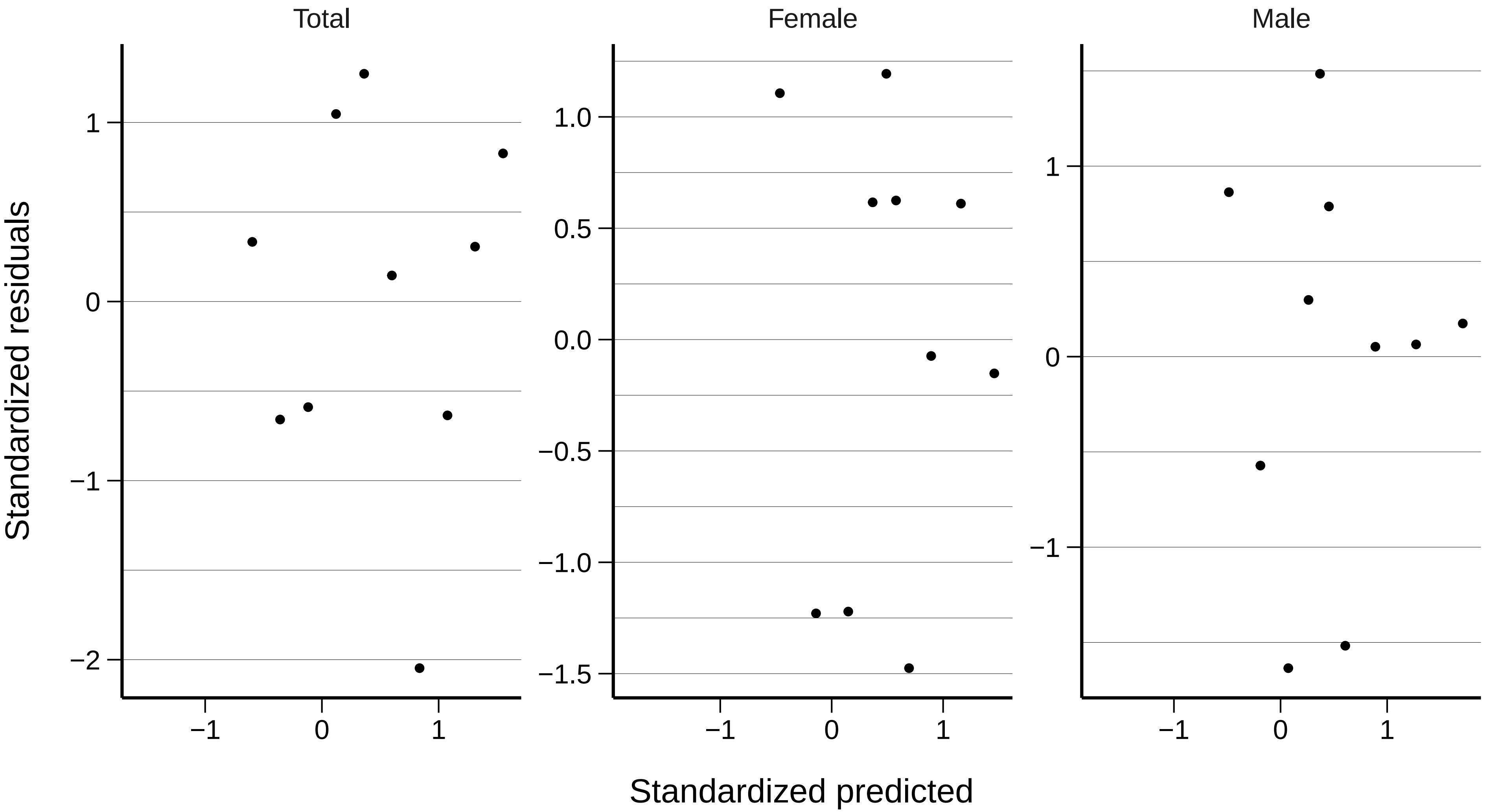

az. NAV: P03 Feeling depressed

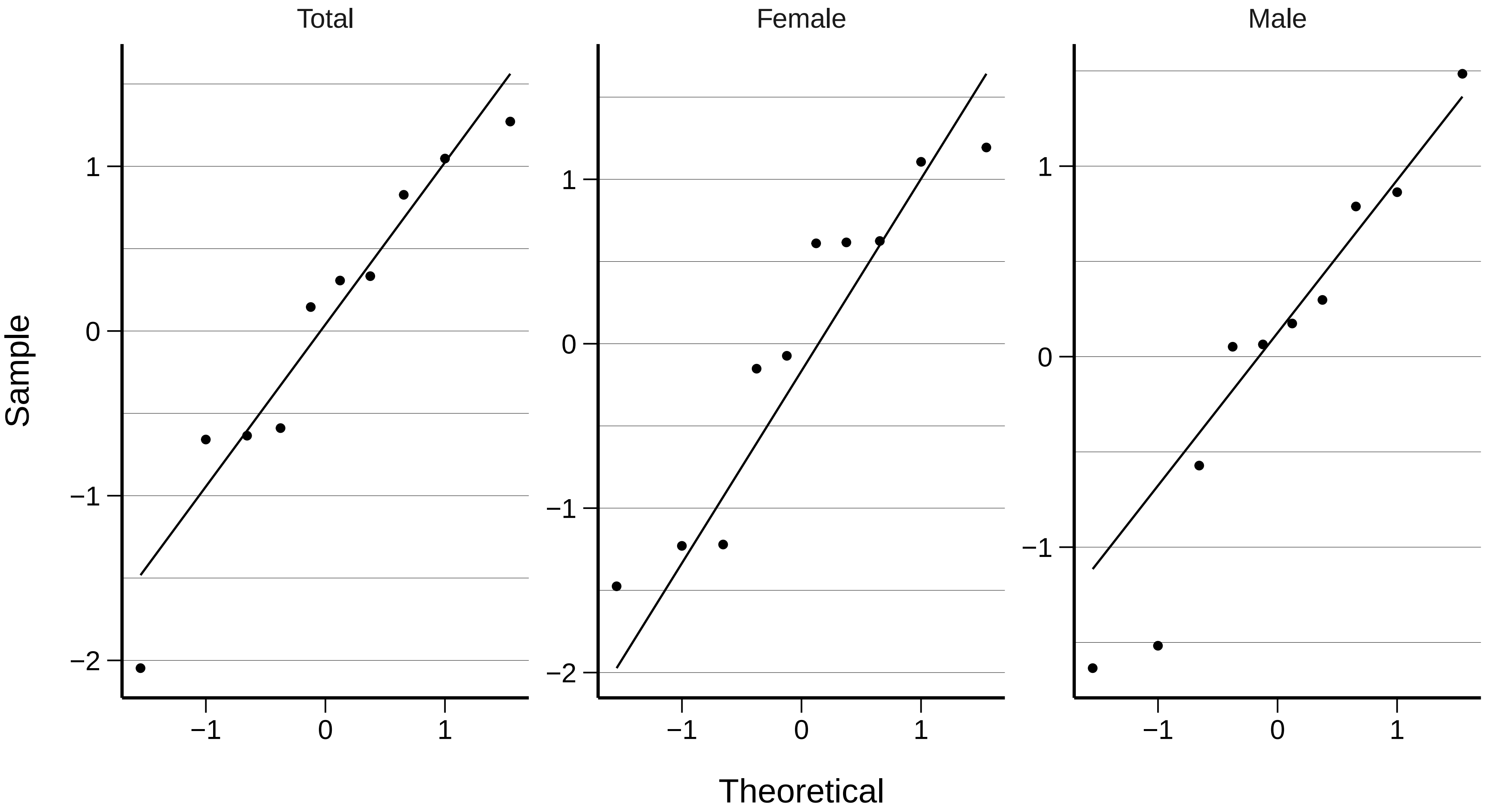

ba. NAV: P19 Drug abuse

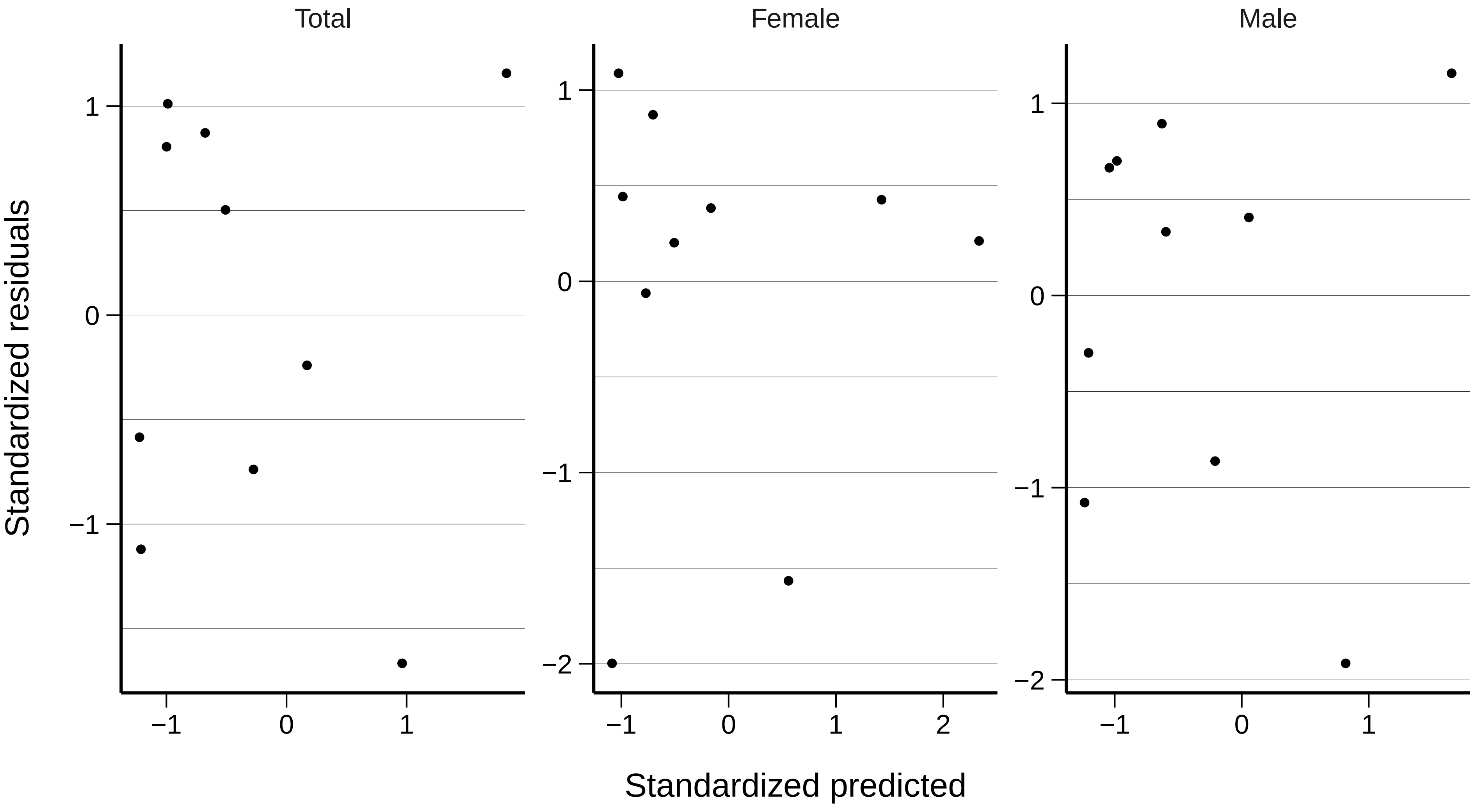

bb. NAV: P19 Drug abuse

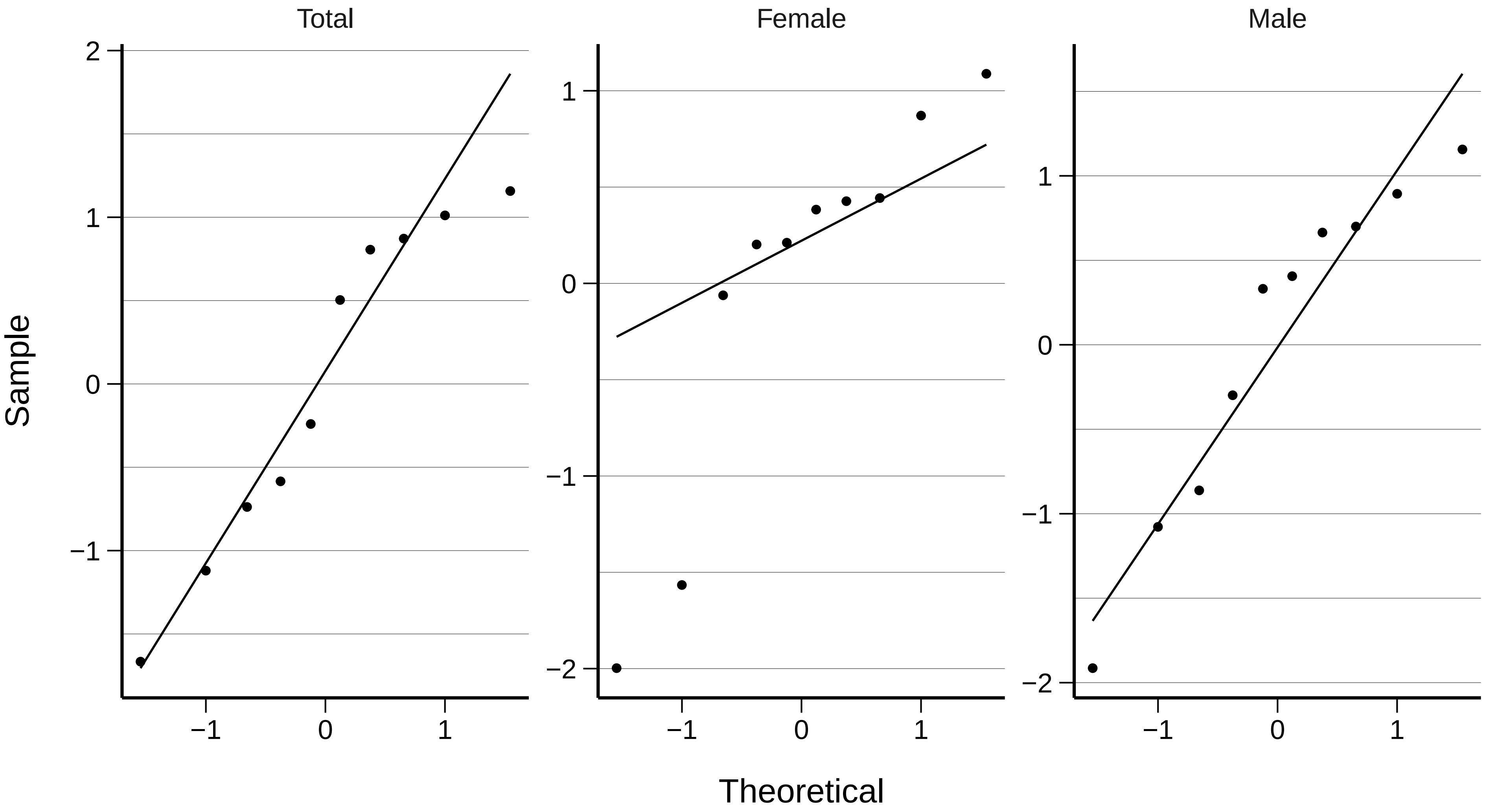

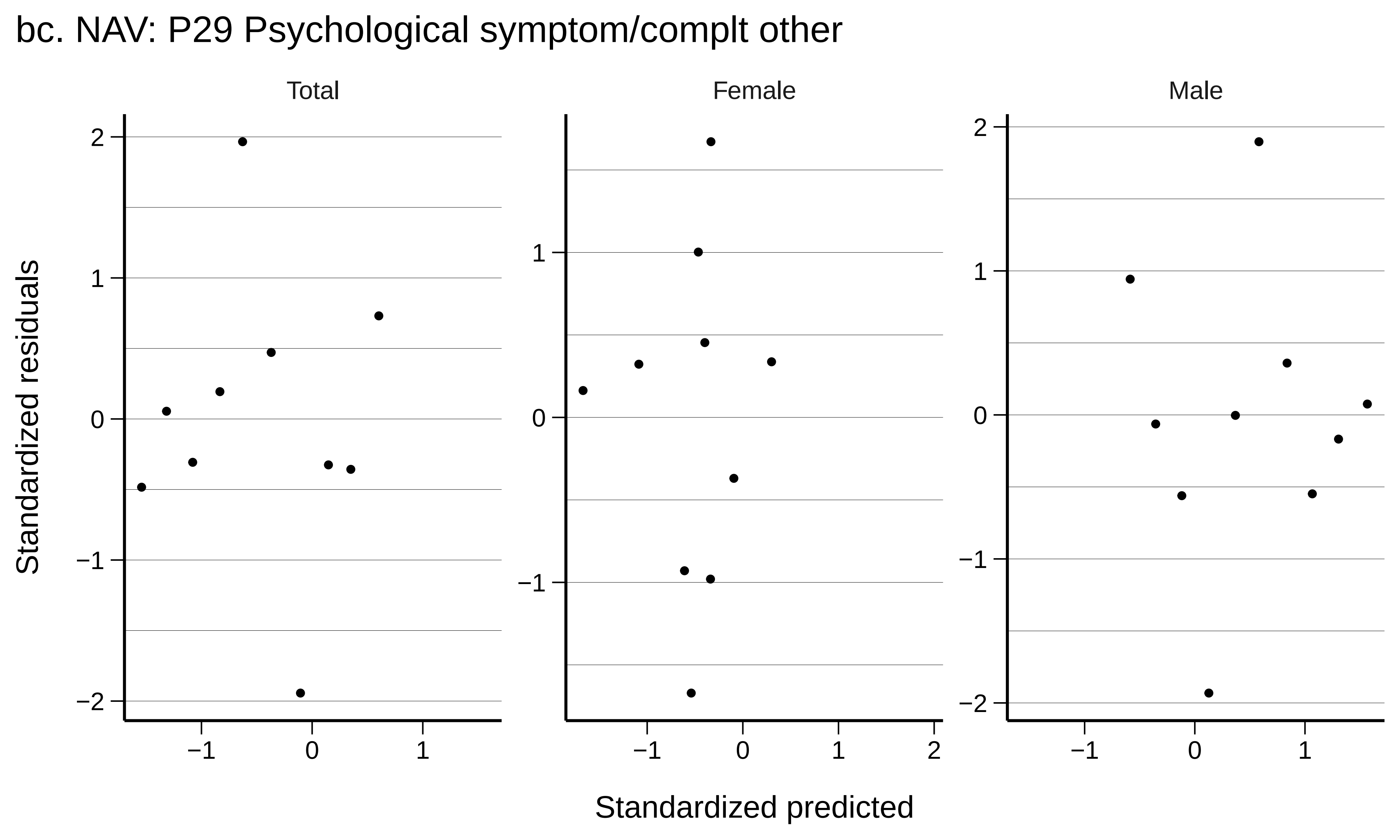
bc. NAV: P29 Psychological symptom/complt other
Total
Female
Male
Standardized residuals
Standardized predicted
2
1
0
−1
−2

bd. NAV: P29 Psychological symptom/complt other

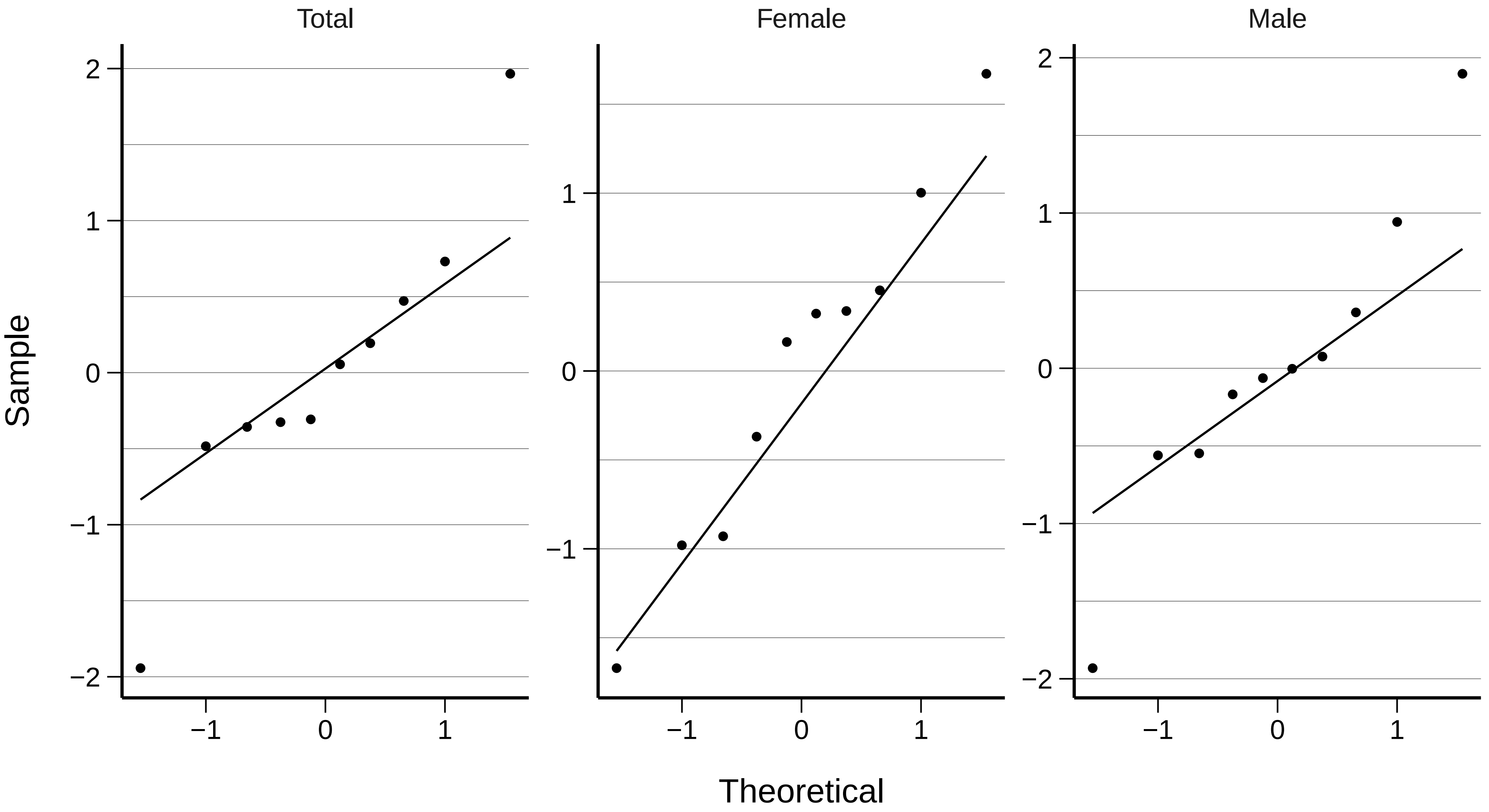

be. NAV: P81 Hyperkinetic disorder

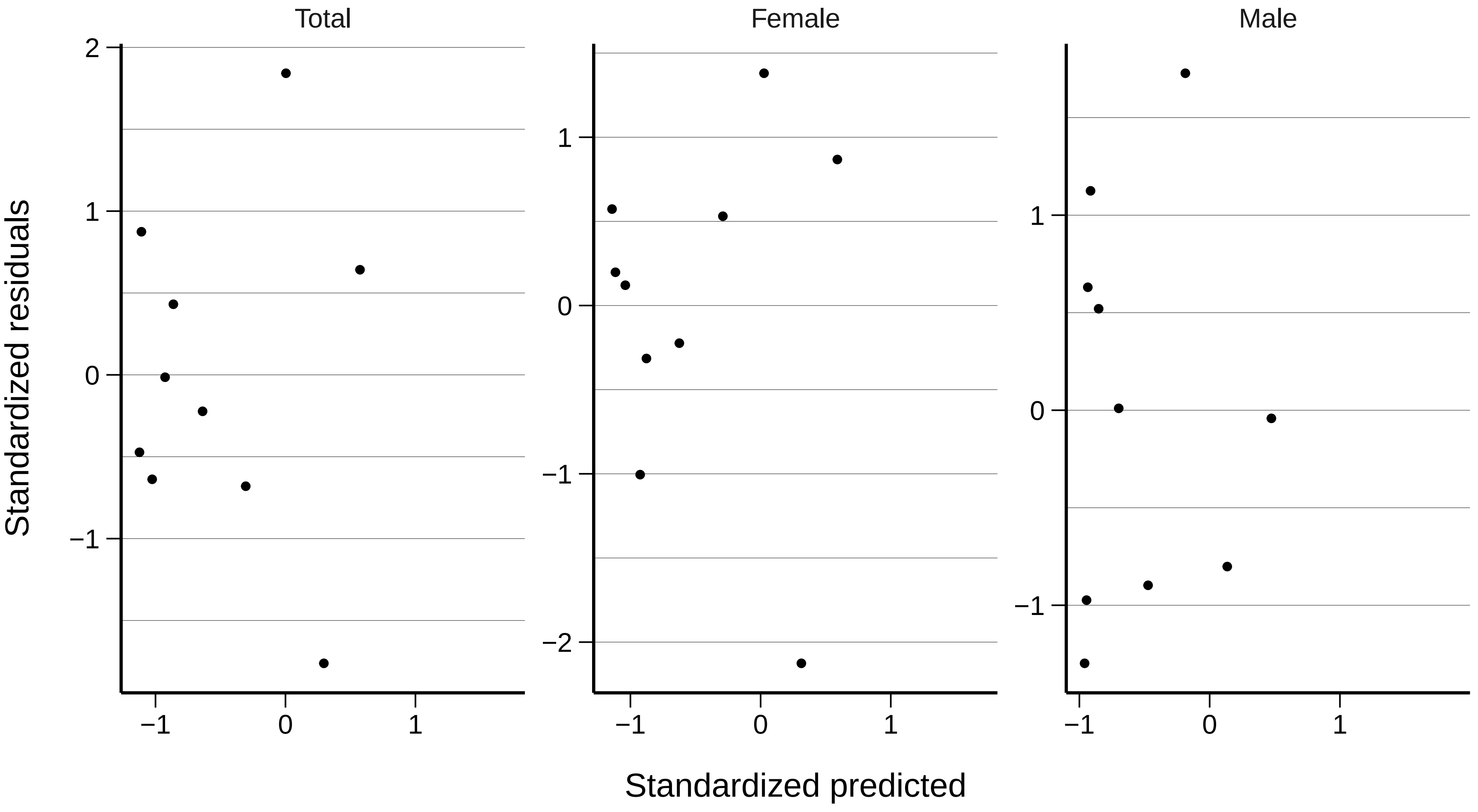

bf. NAV: P81 Hyperkinetic disorder

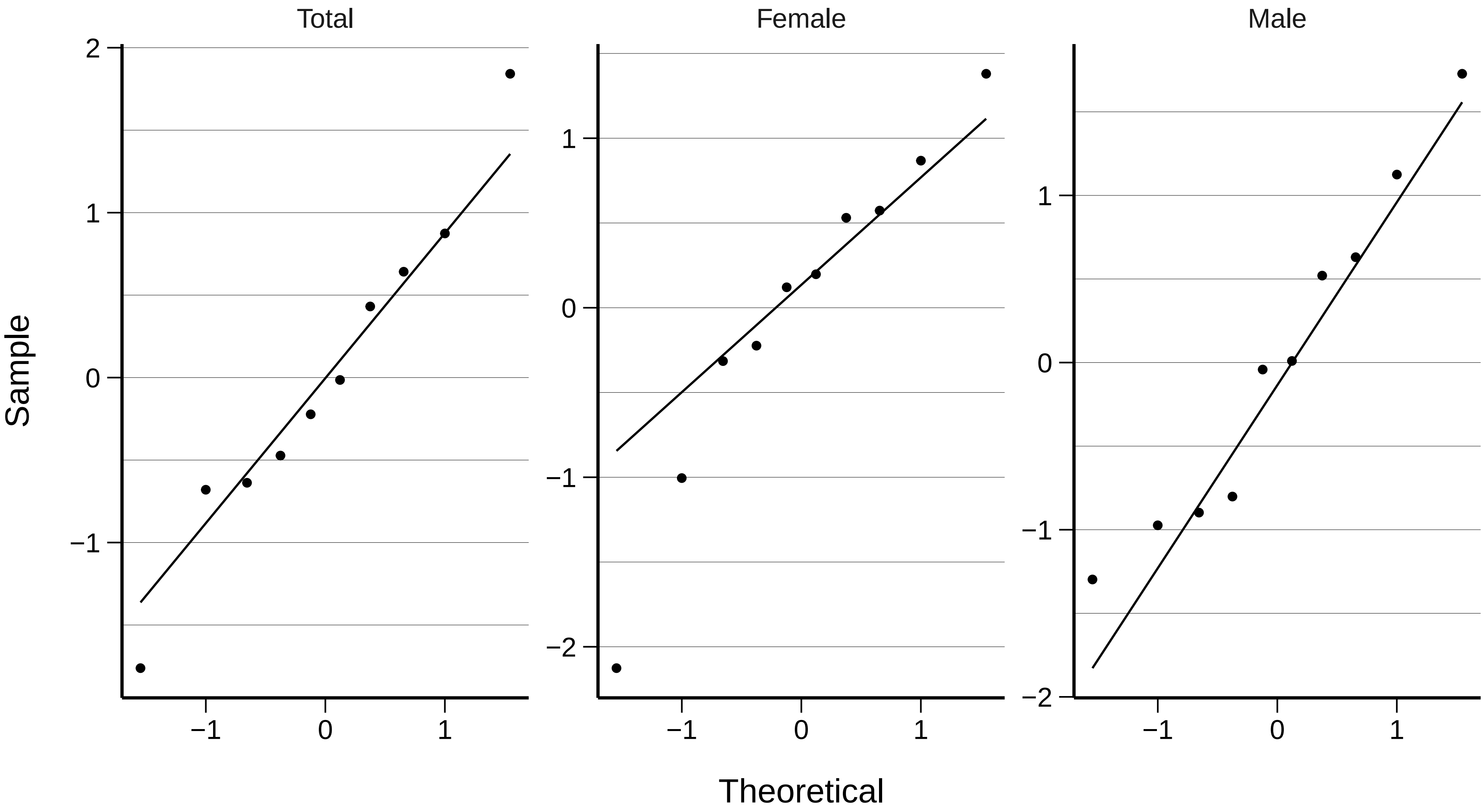

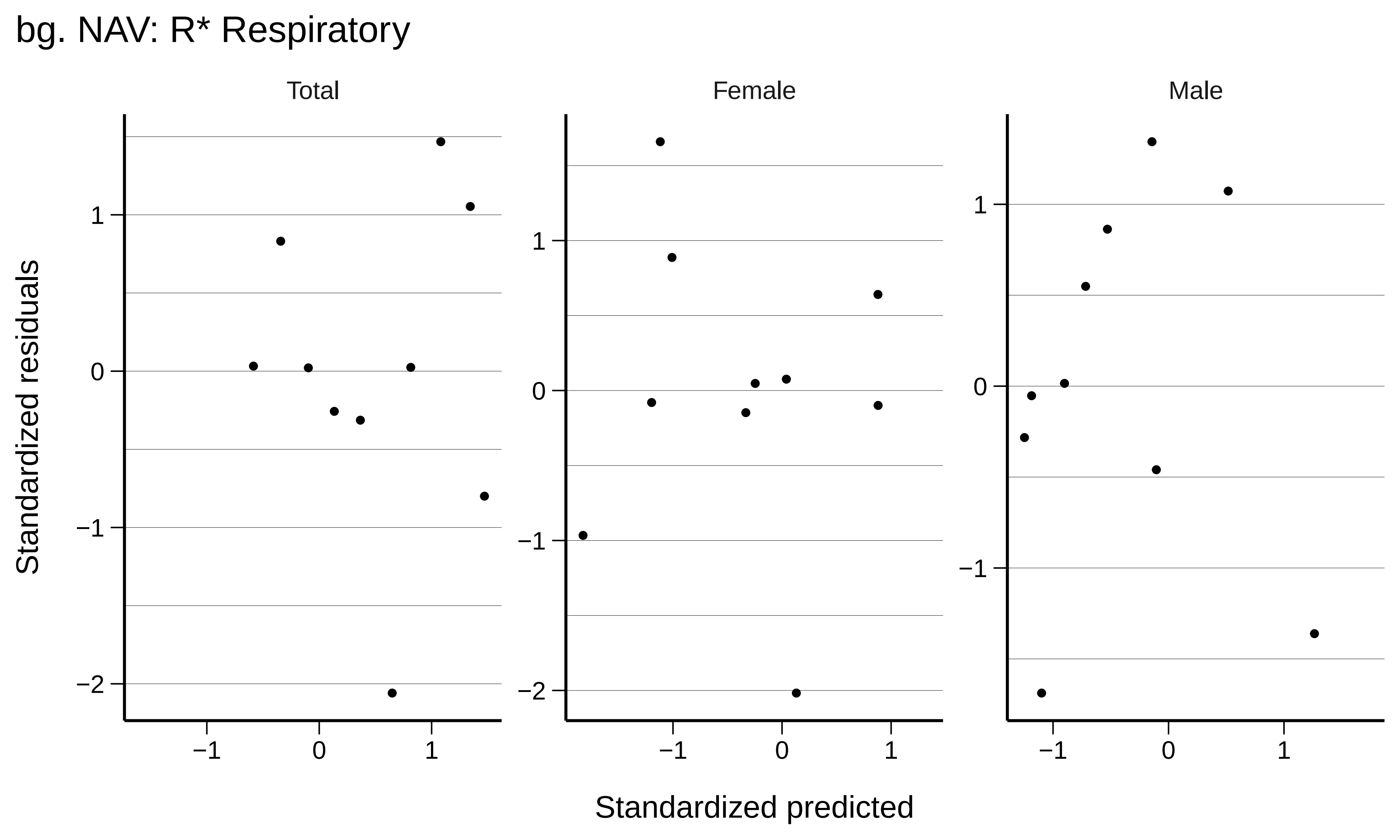

bg. NAV: R* Respiratory
Total
Female
Male
Standardized residuals
Standardized predicted
1
0
−1
−2

bh. NAV: R* Respiratory

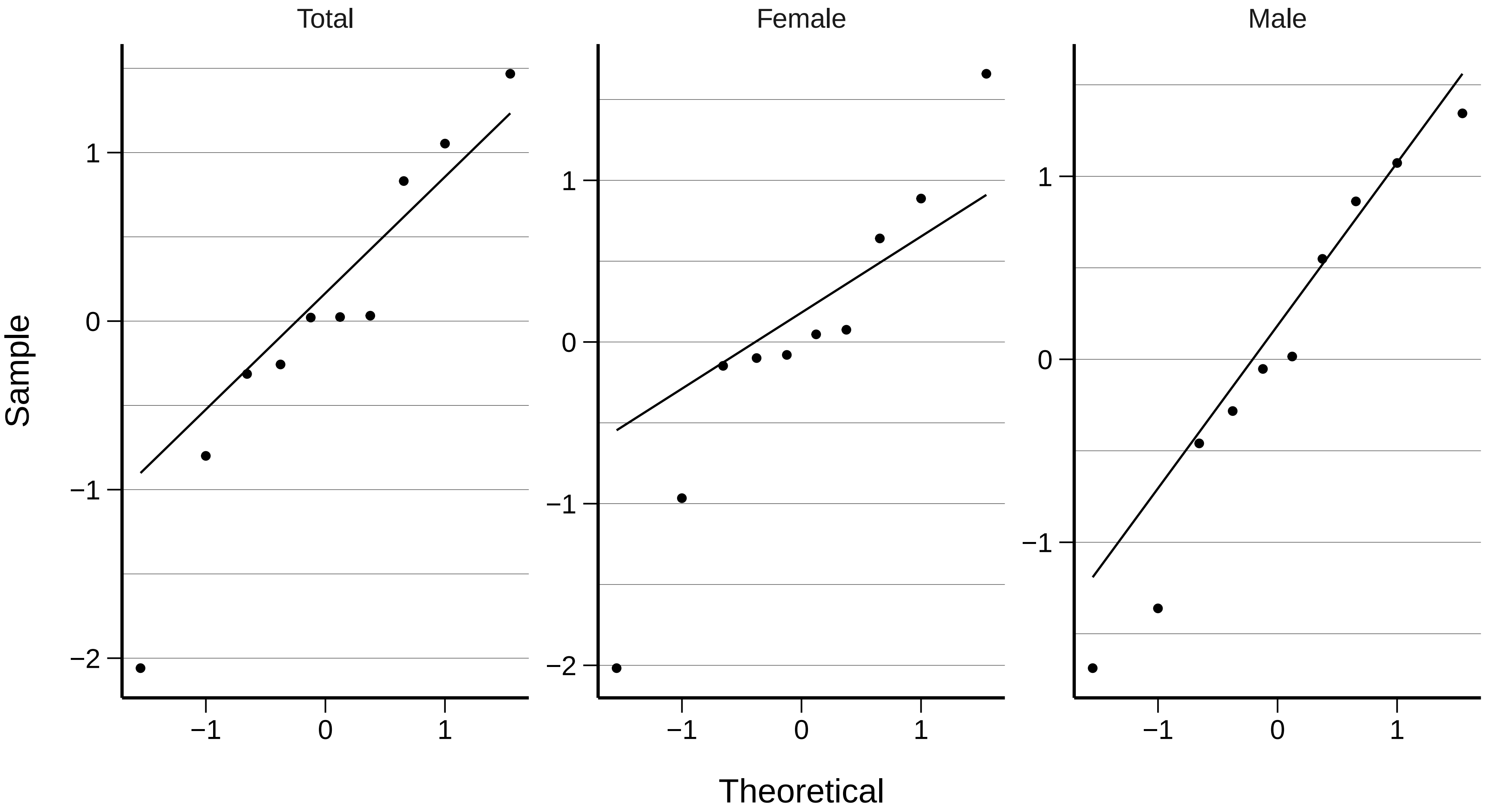

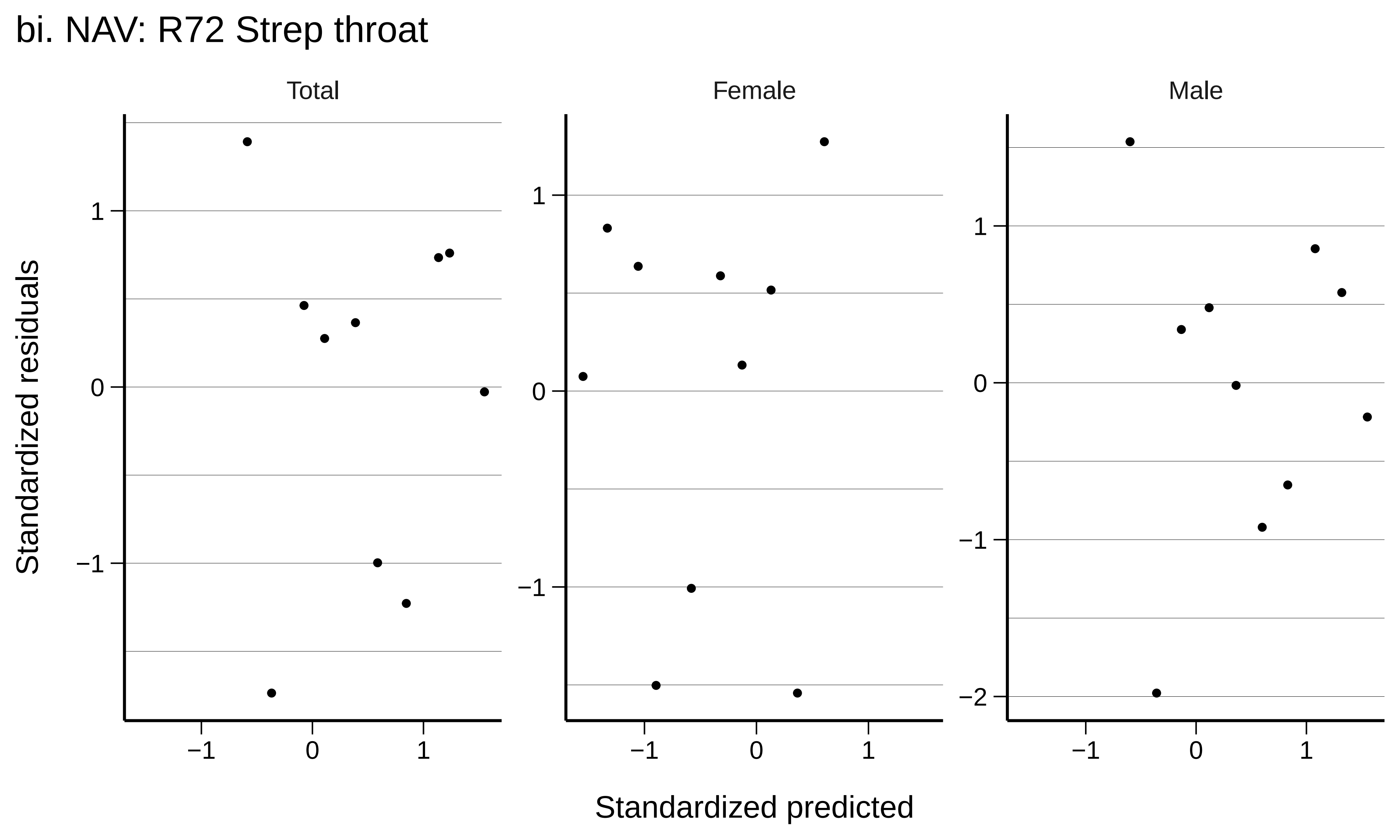
bi. NAV: R72 Strep throat
Total
Female
Male
Standardized residuals
Standardized predicted
1
0
−1
−2
−1
0
1

bj. NAV: R72 Strep throat

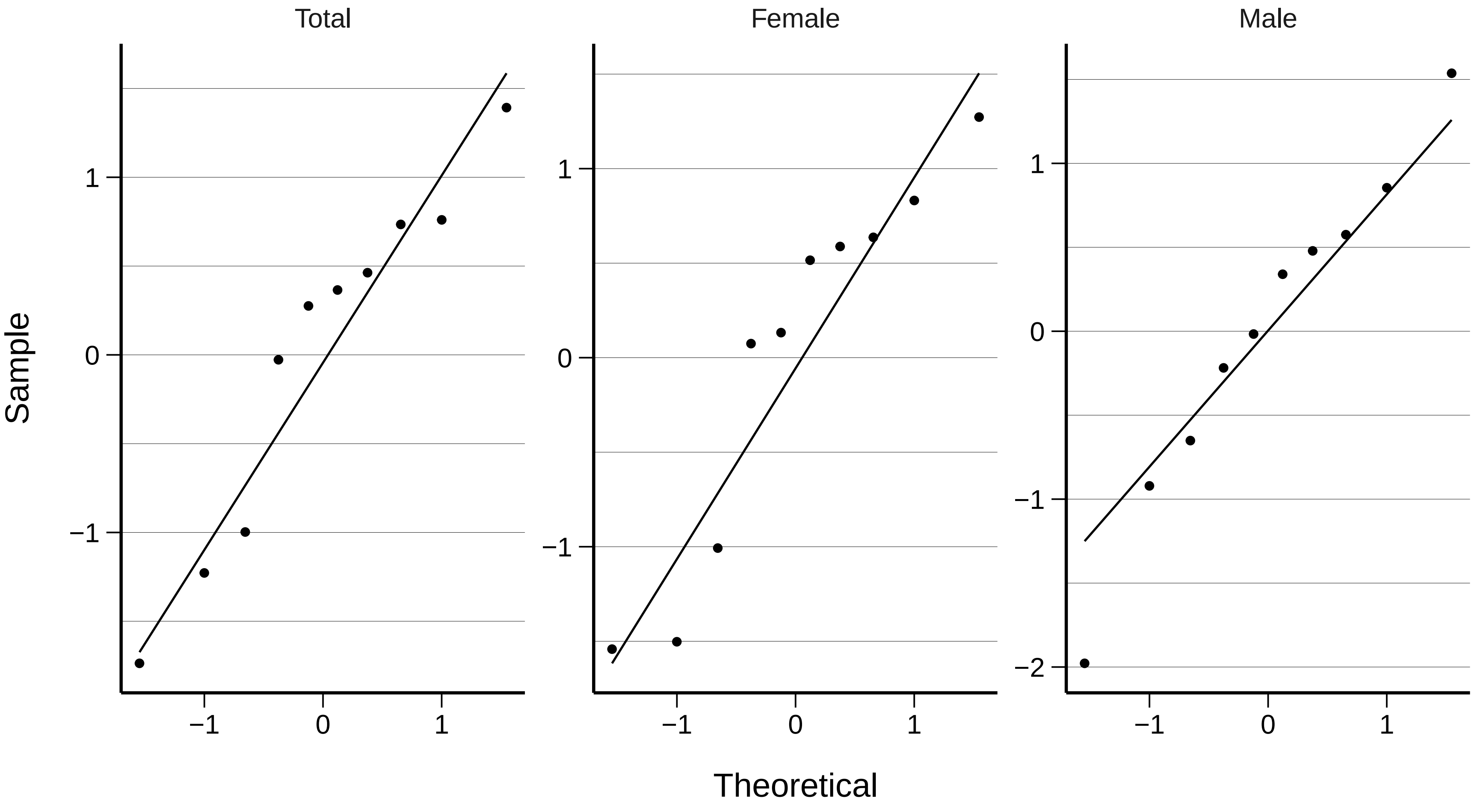

bk. NAV: R74 Upper respiratory infection acute

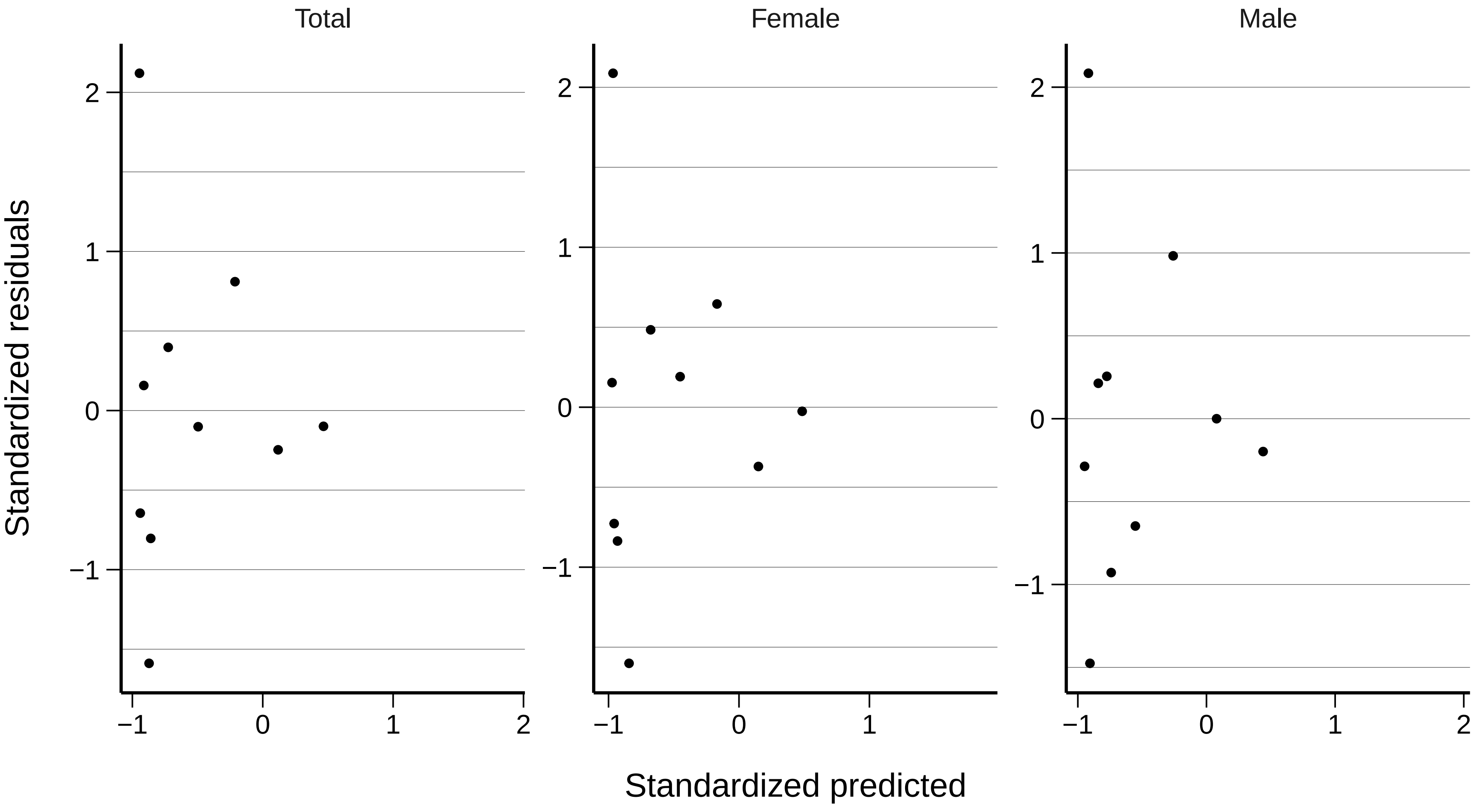

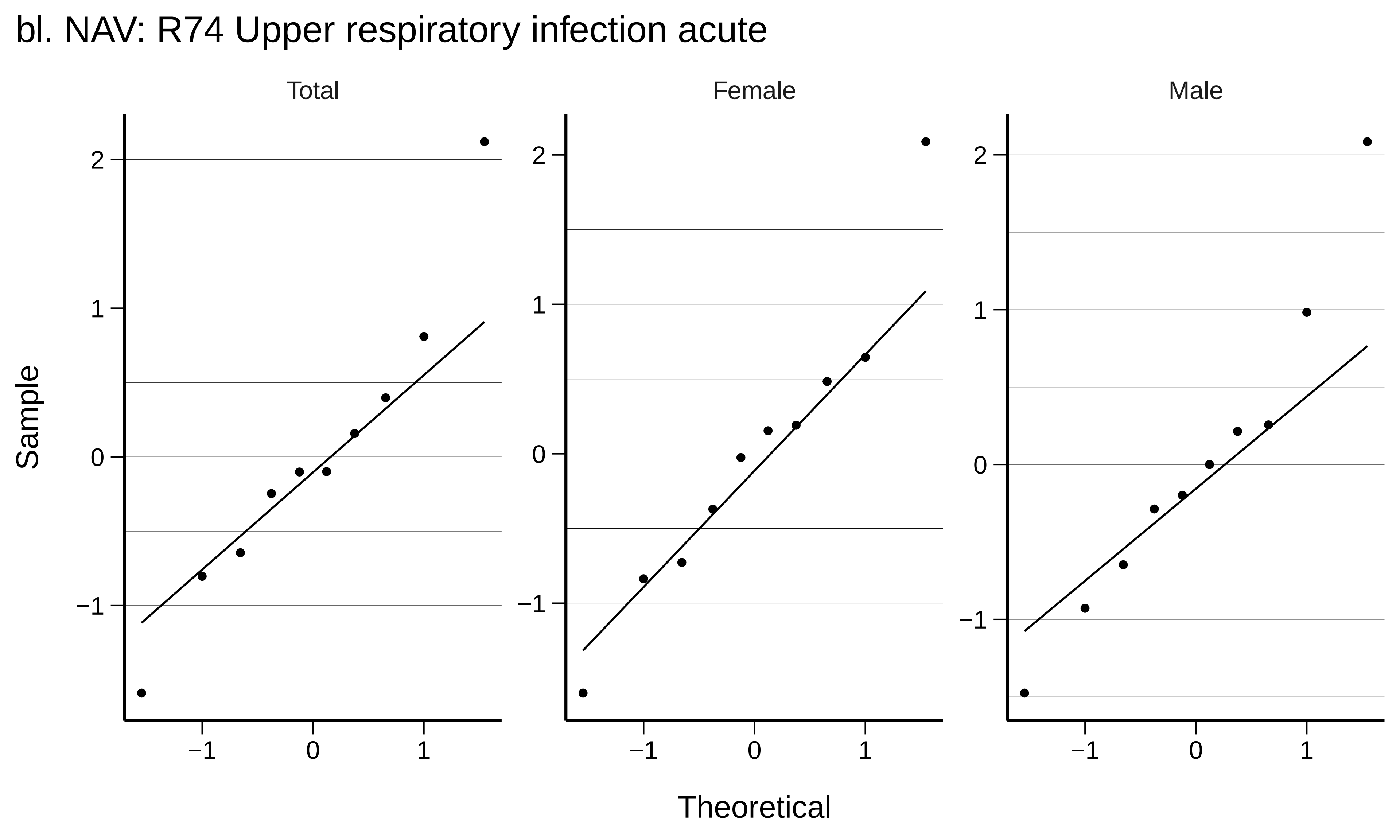

bl. NAV: R74 Upper respiratory infection acute
Total
Female
Male
Sample
Theoretical
2
1
0
−1
−1
0
1

bm. NAV: R90 Hypertrophy tonsils/adenoids

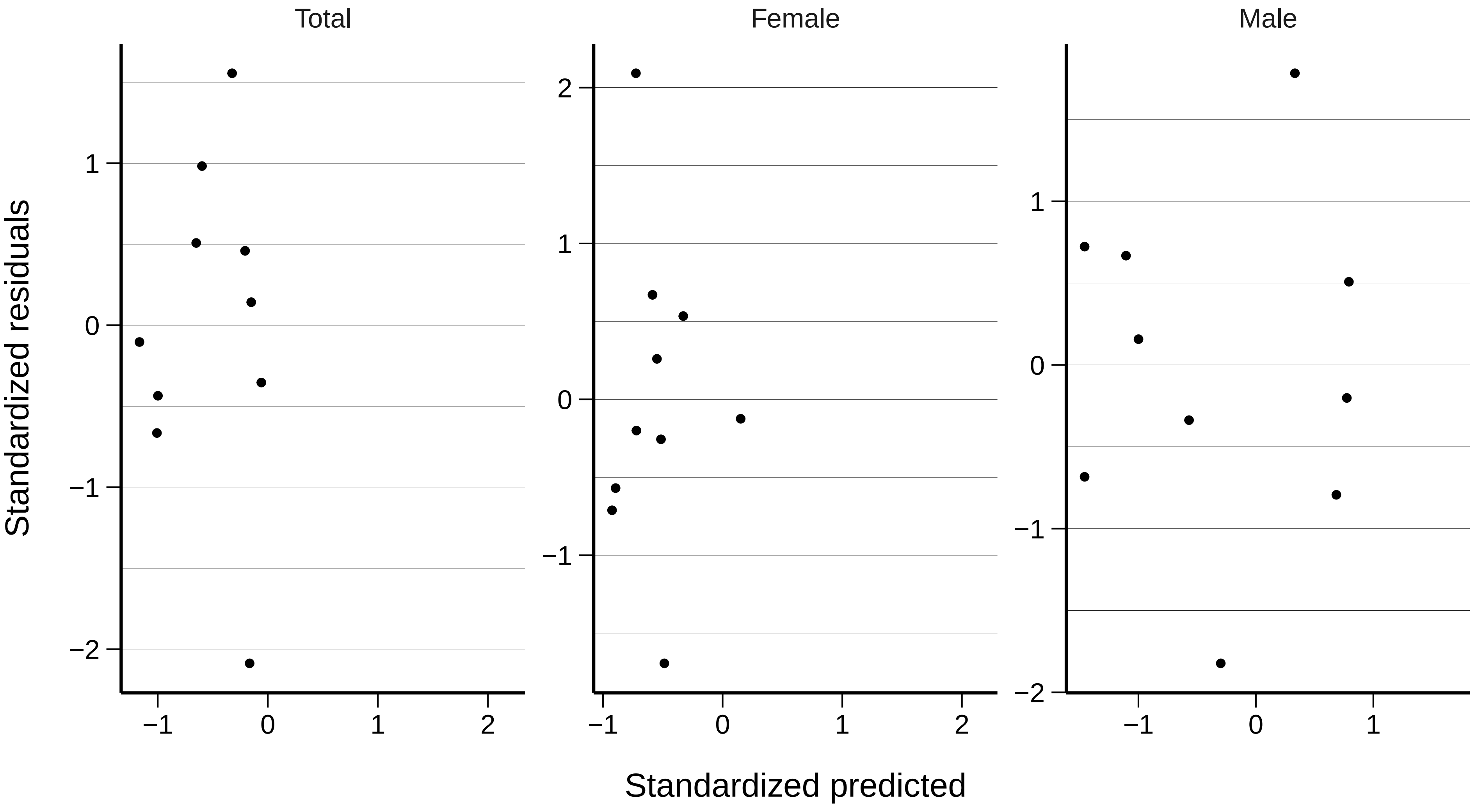

bn. NAV: R90 Hypertrophy tonsils/adenoids

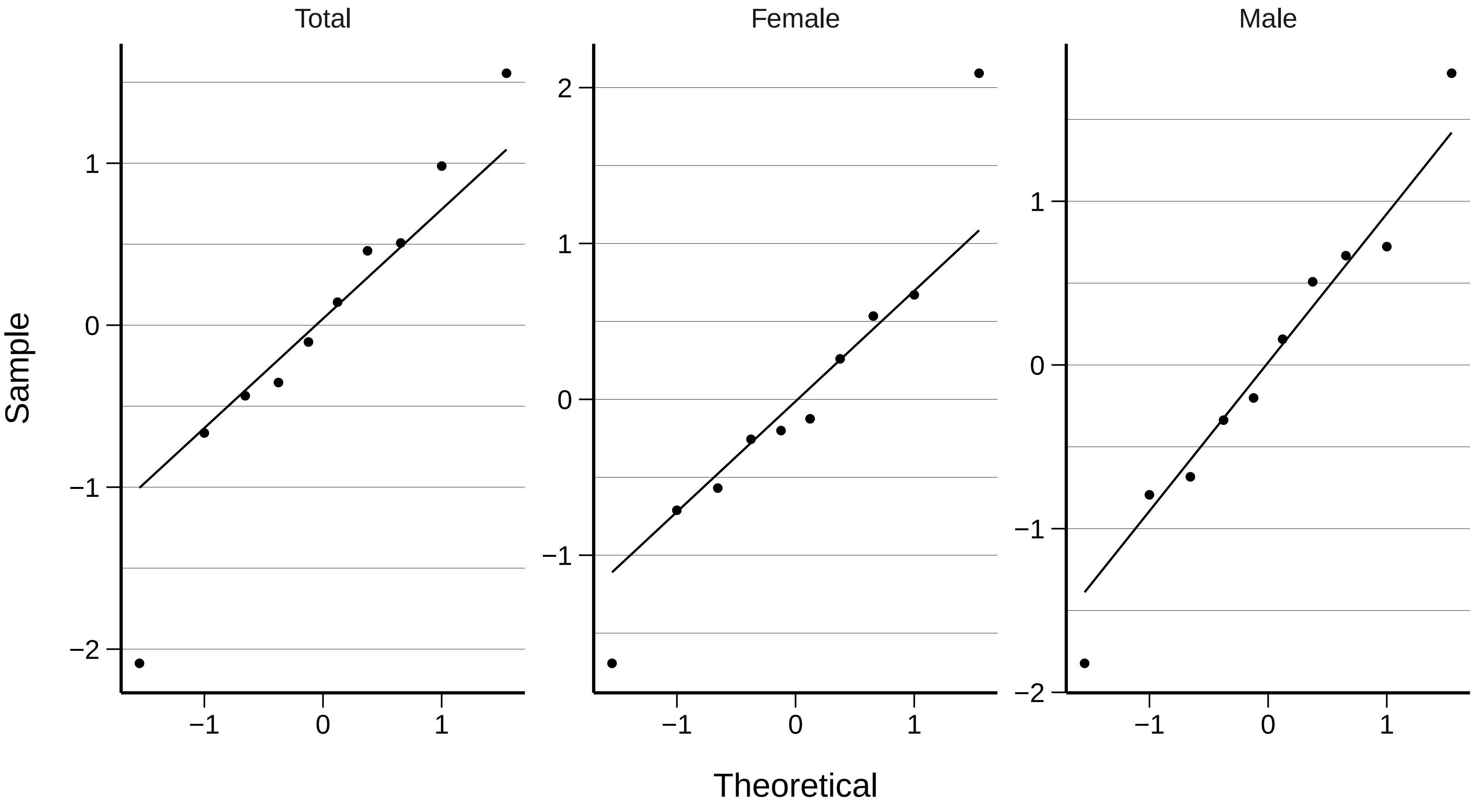

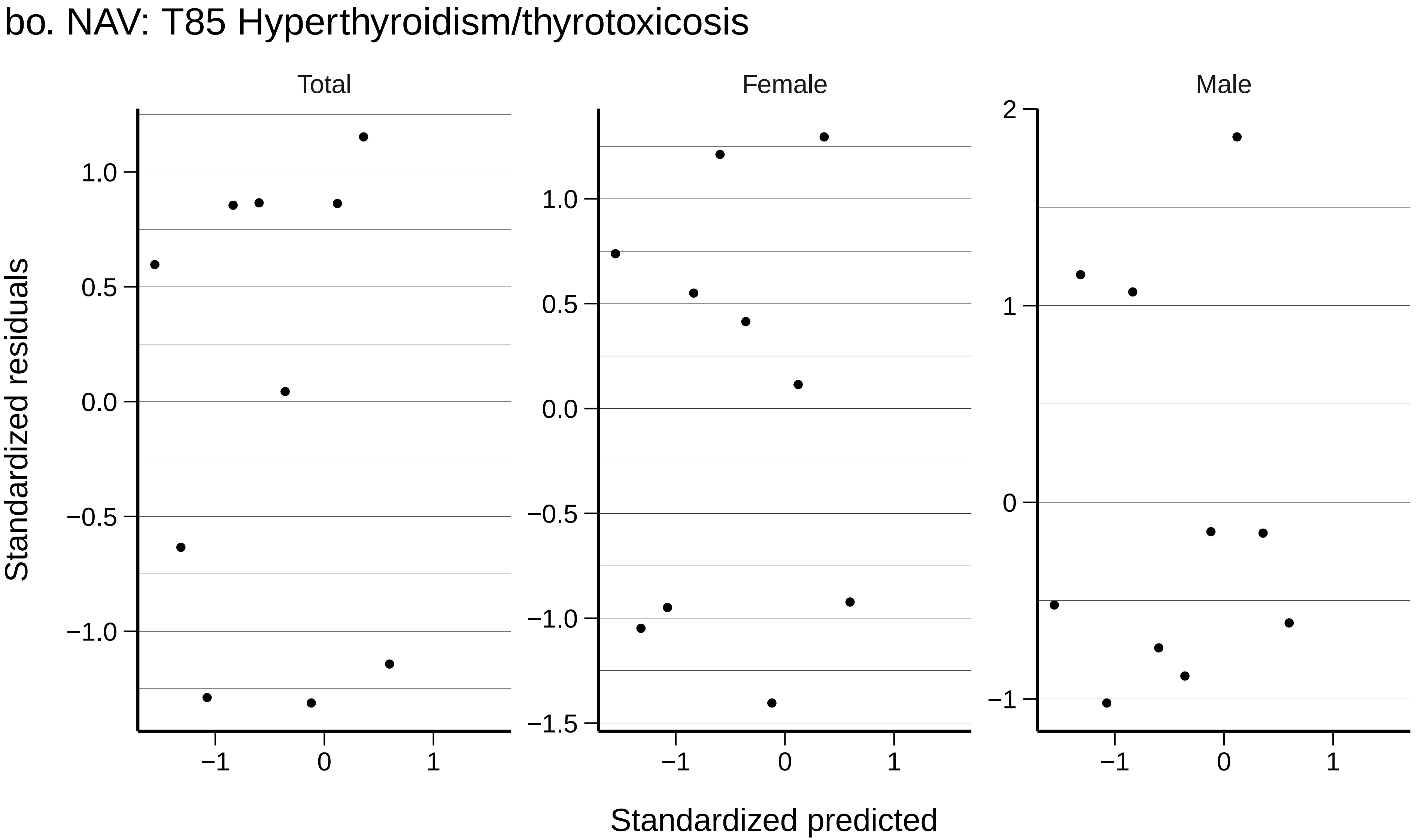
bo. NAV: T85 Hyperthyroidism/thyrotoxicosis
Total
Female
Male
Standardized residuals
Standardized predicted
1.0
0.5
0.0
−0.5
−1.0
−1.5
2
1
0
−1
−1
0
1

bp. NAV: T85 Hyperthyroidism/thyrotoxicosis

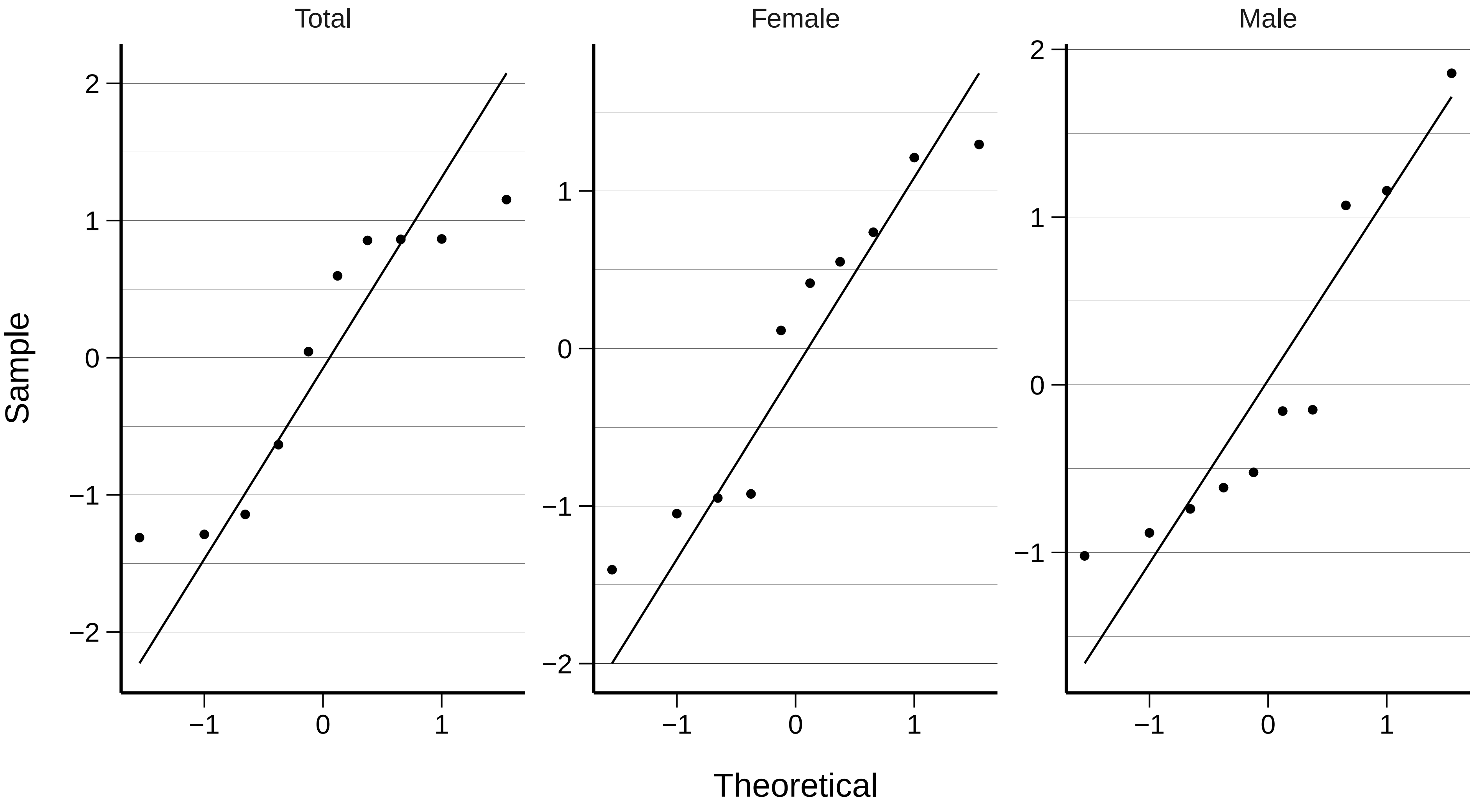

bq. NAV: T99 Endocrine/metab/nutrit. dis. other

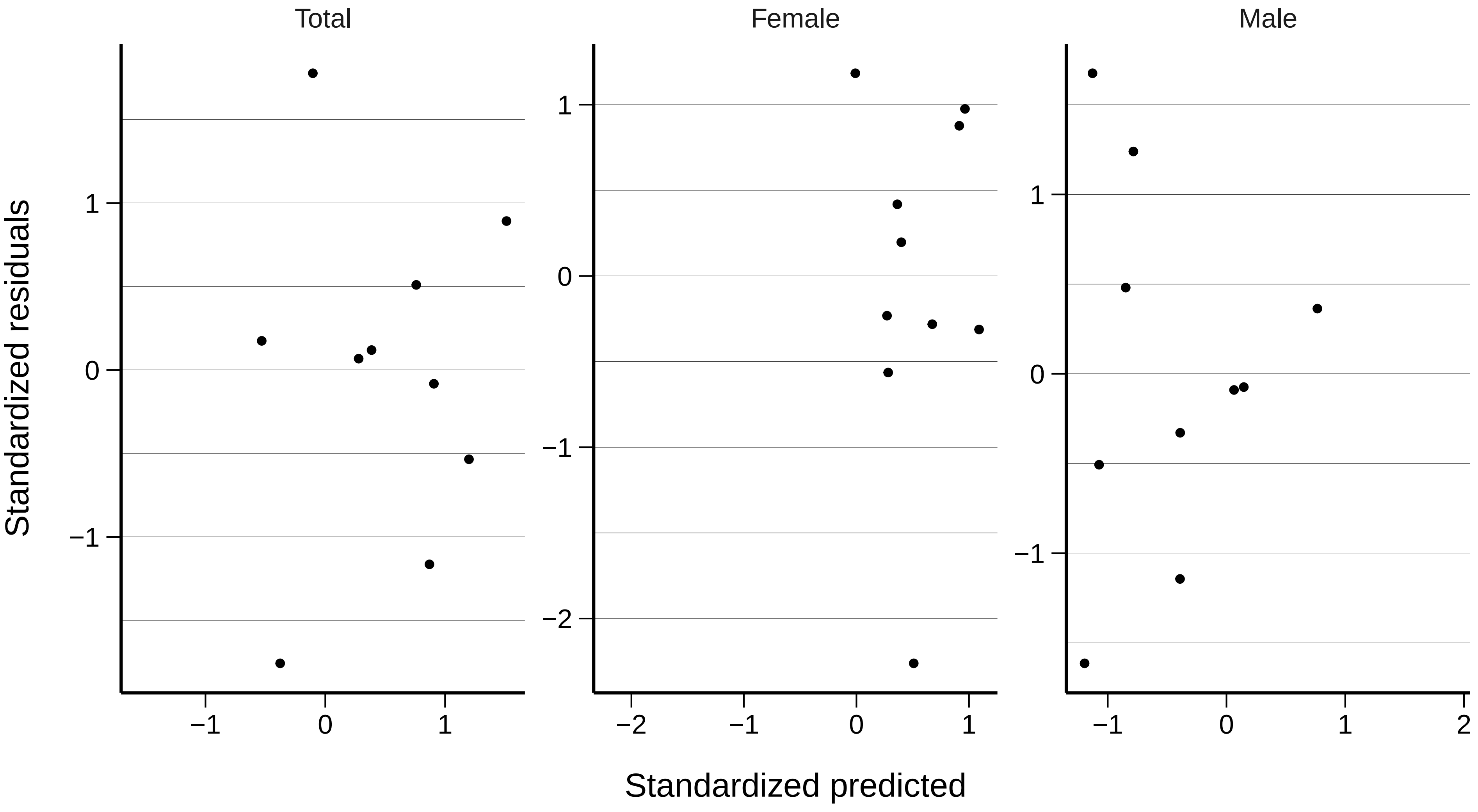

br. NAV: T99 Endocrine/metab/nutrit. dis. other

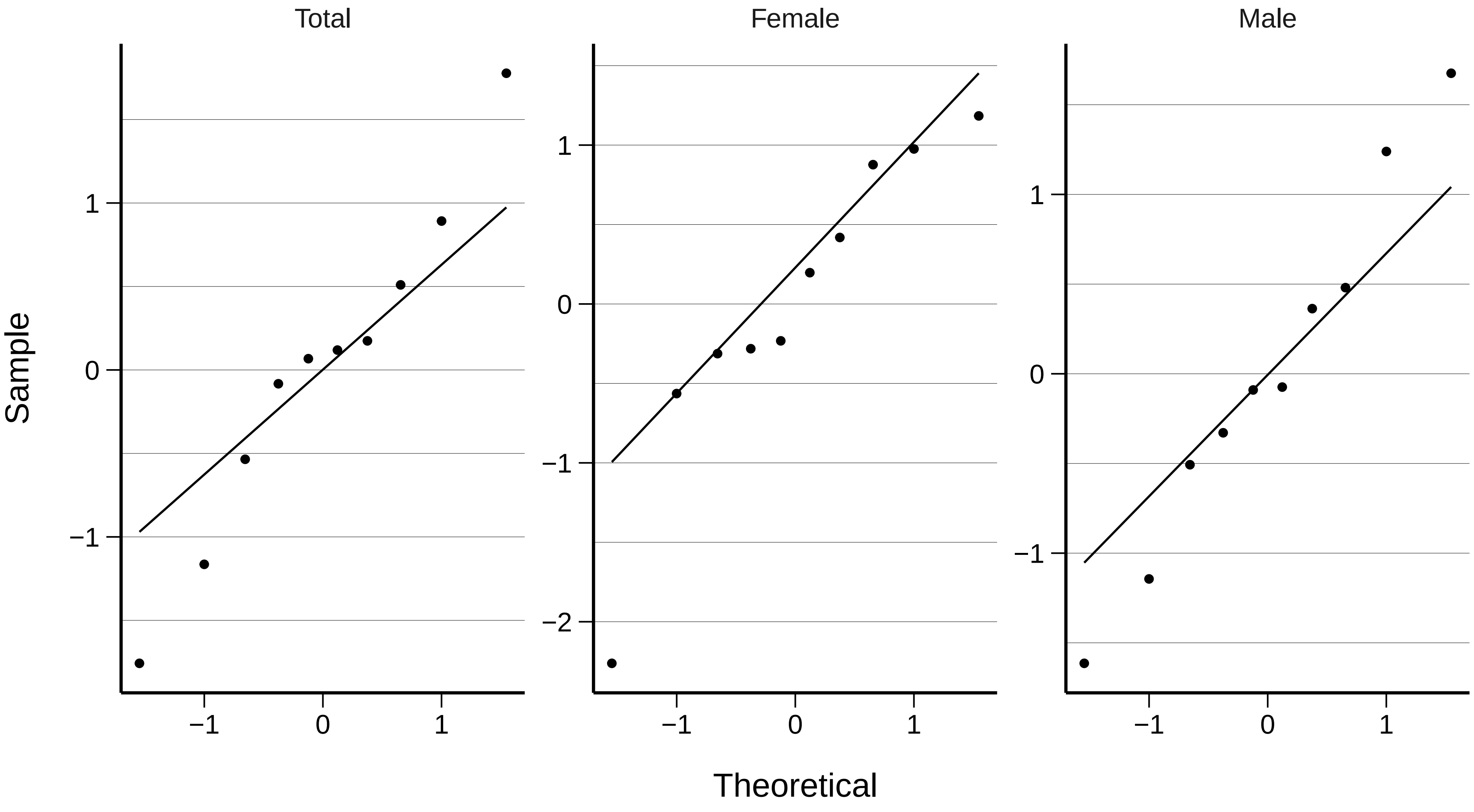

bs. NAV: X21 Breast symptom/complt. female other

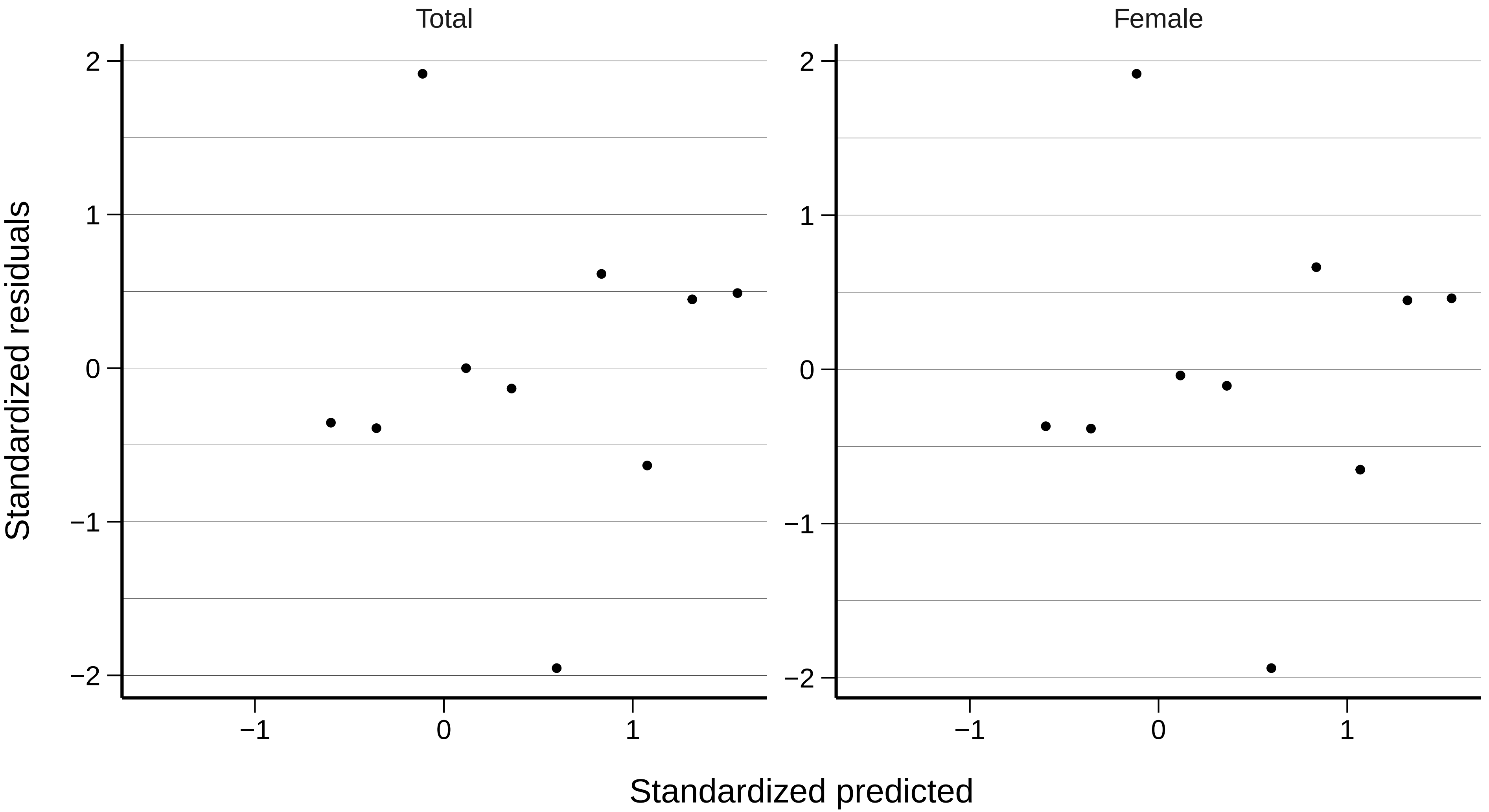

bt. NAV: X21 Breast symptom/complt. female other

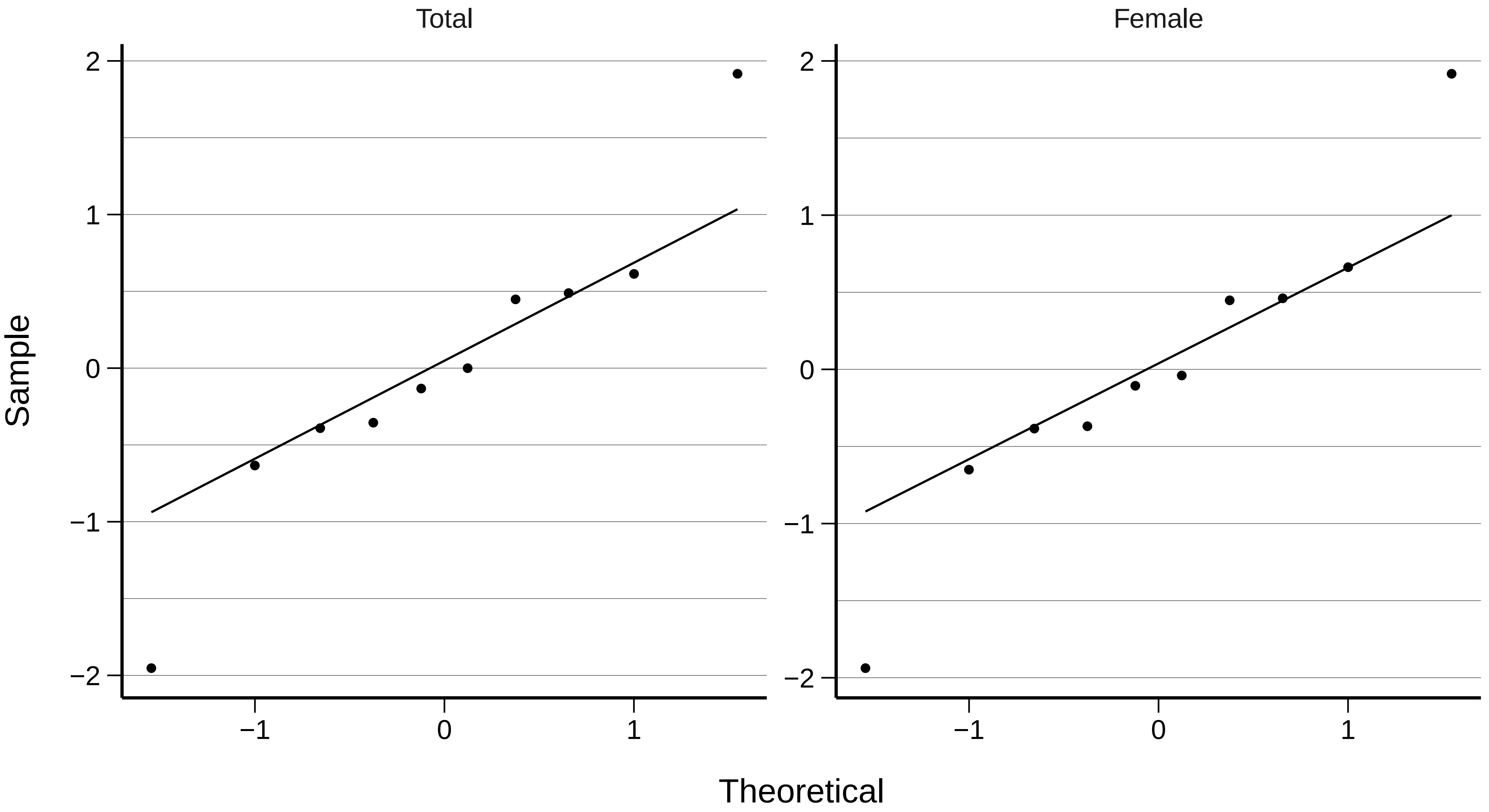

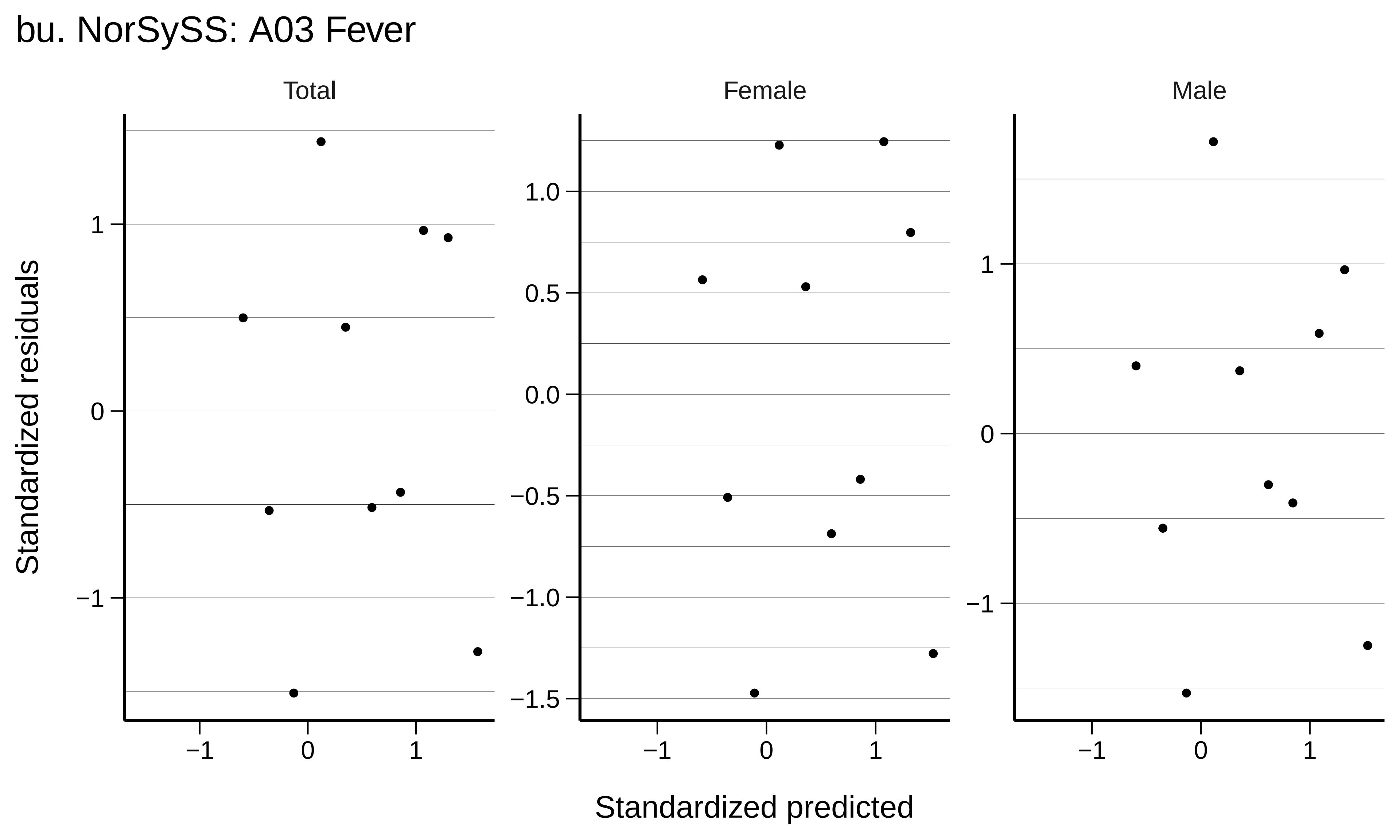

bu. NorSySS: A03 Fever
Total
Female
Male
Standardized residuals
Standardized predicted

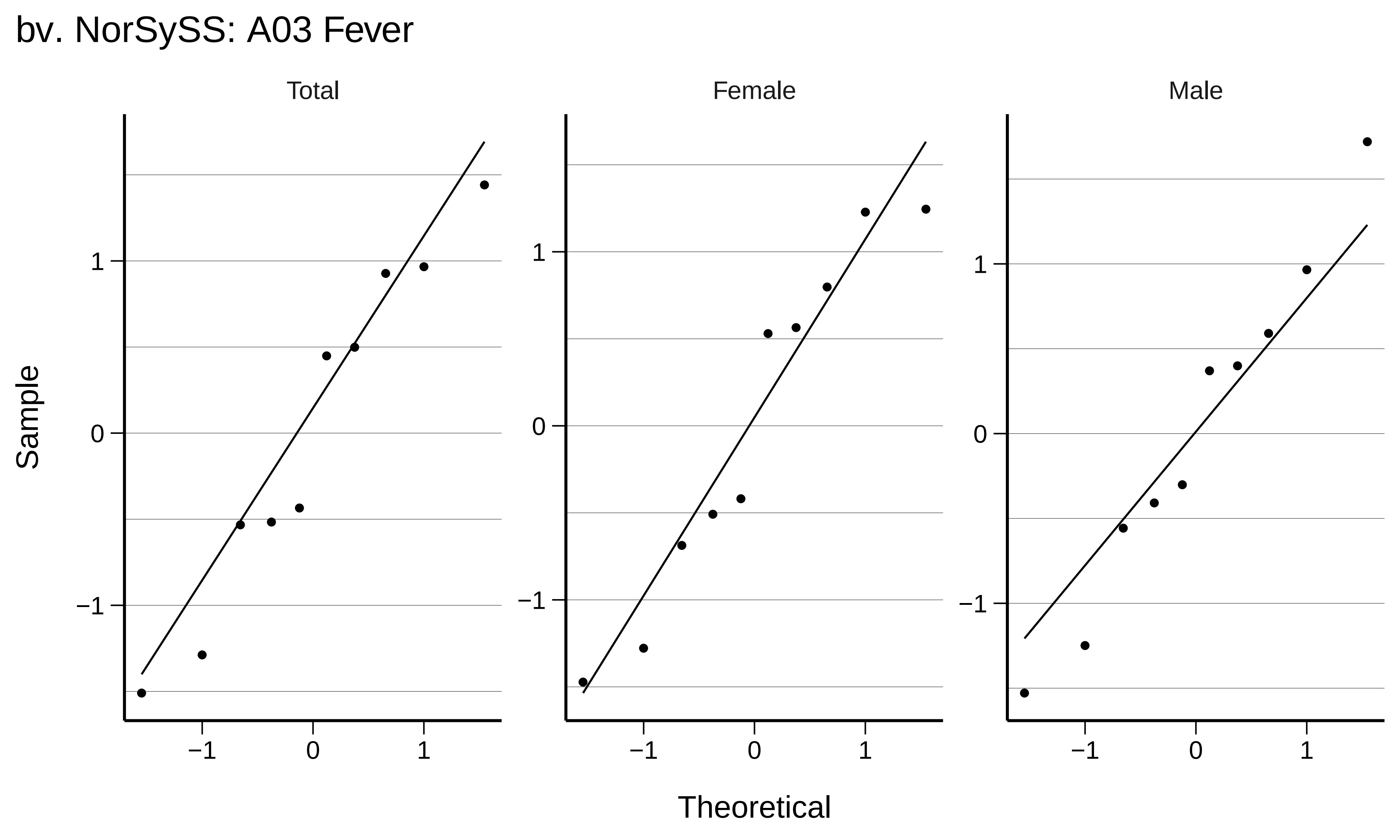

bv. NorSySS: A03 Fever
Total
Female
Male
Sample
Theoretical
1
0
−1

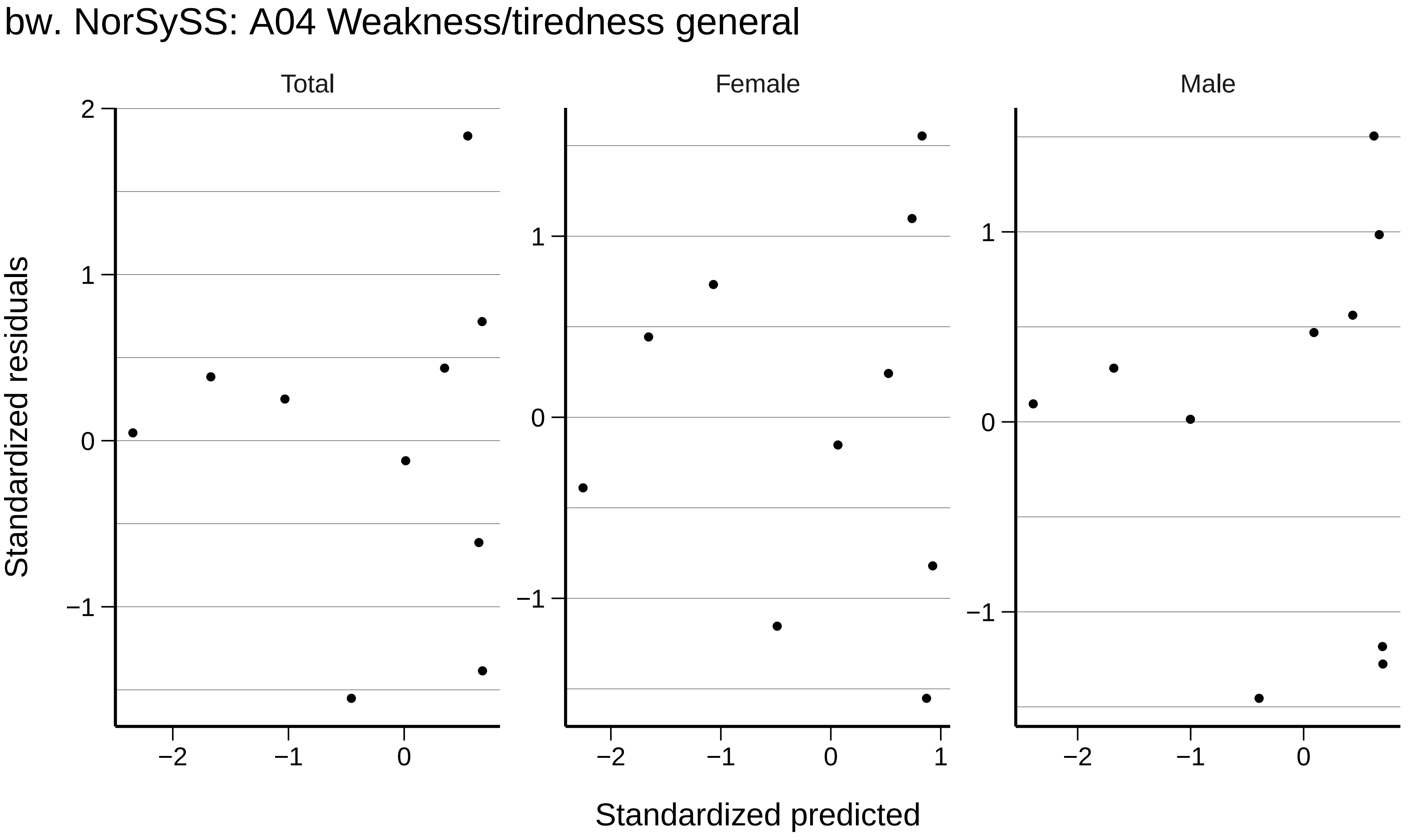
bw. NorSySS: A04 Weakness/tiredness general
Total
Female
Male
Standardized residuals
Standardized predicted
2
1
0
−1
−2
−1
0
1

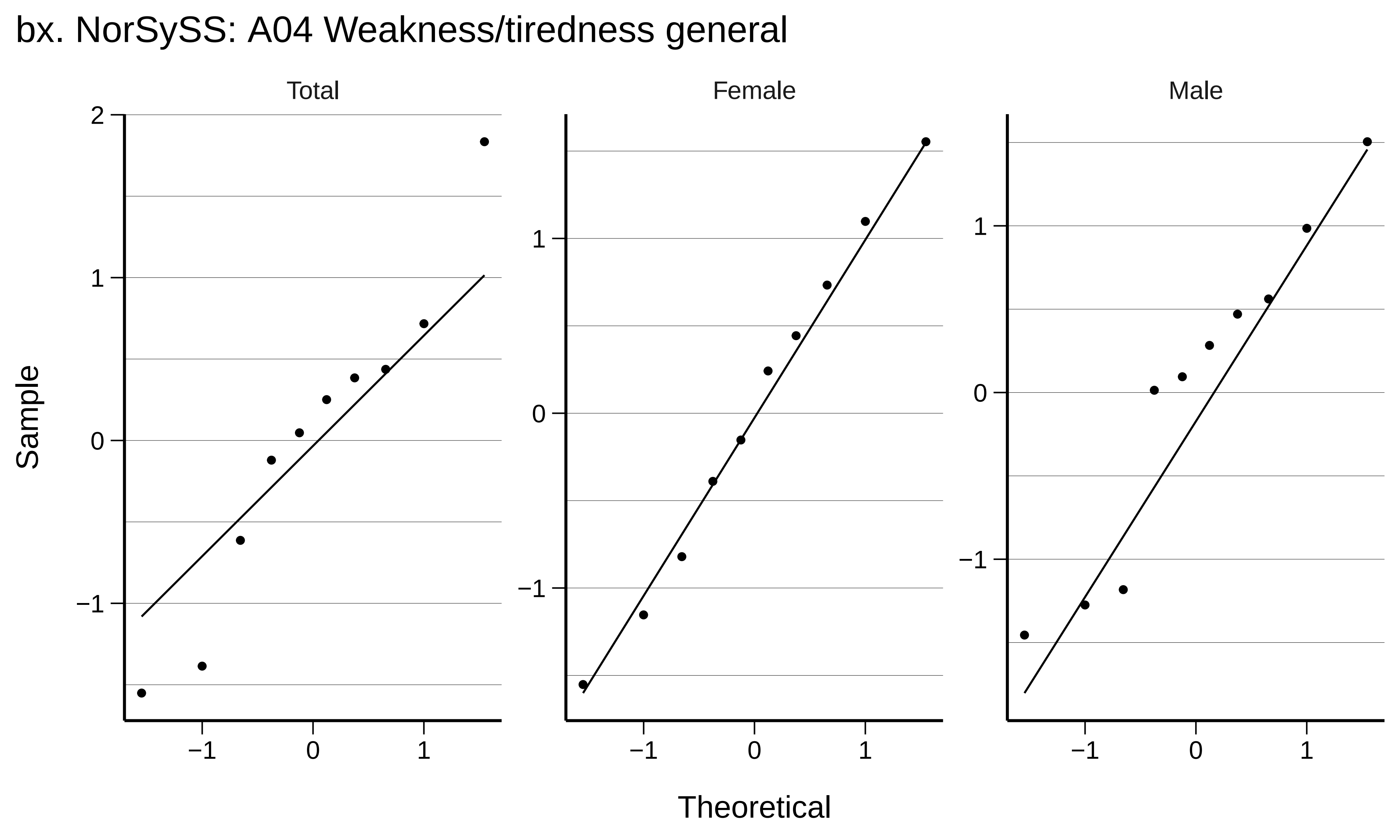

bx. NorSySS: A04 Weakness/tiredness general
Total
Female
Male
Sample
Theoretical
2
1
0
−1
−1
0
1

by. NorSySS: A05 Feeling ill

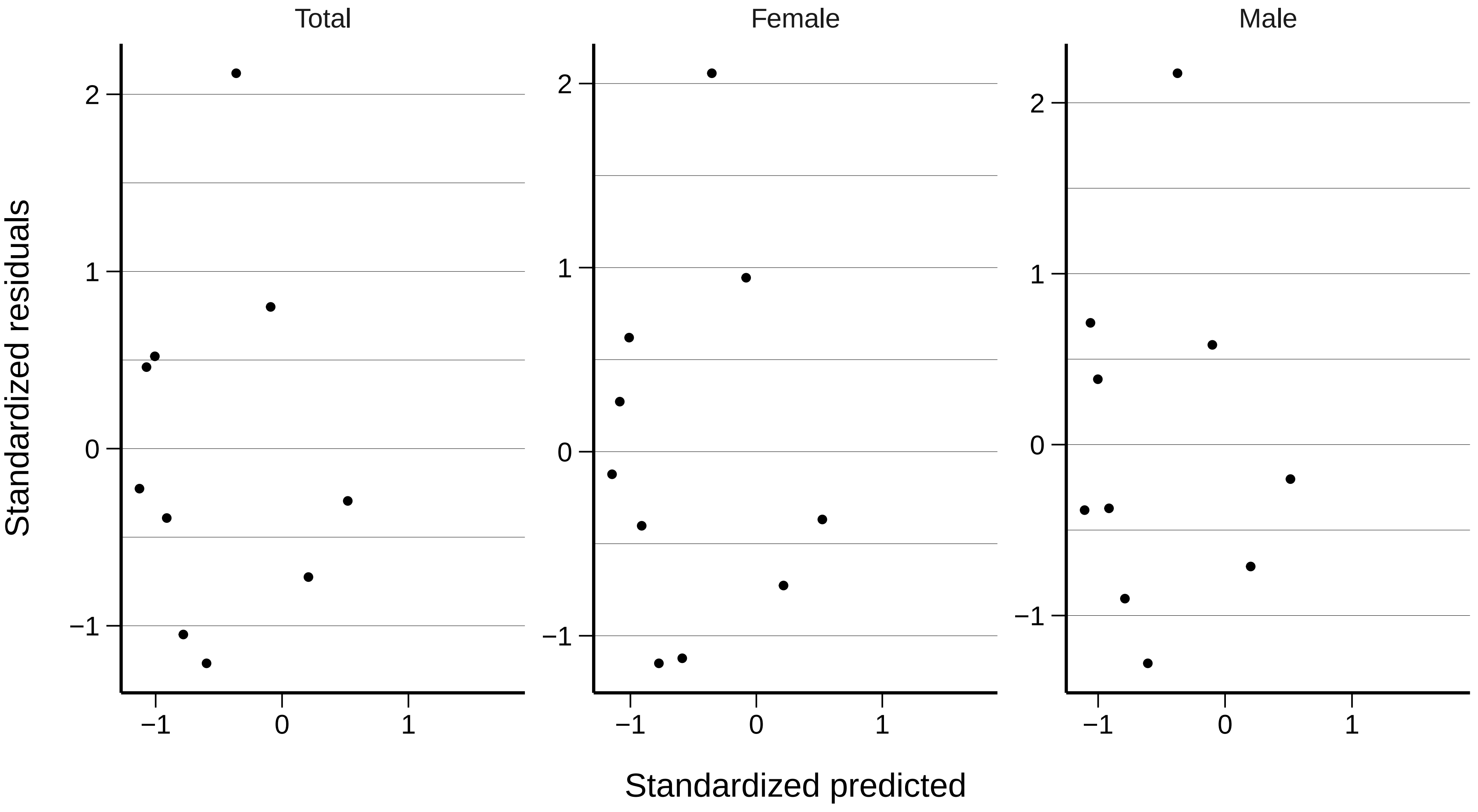

bz. NorSySS: A05 Feeling ill

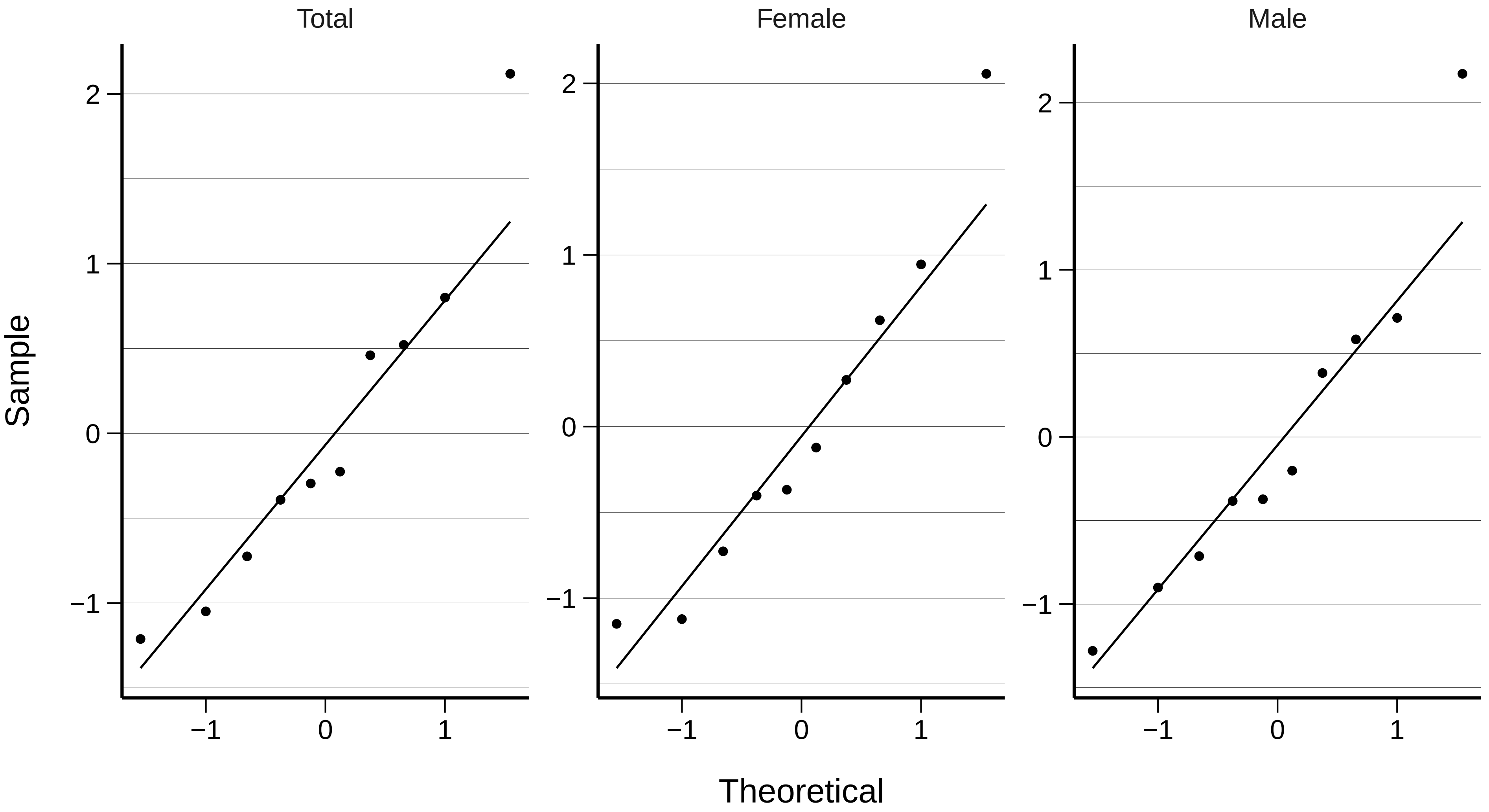

ca. NorSySS: A78 Infectious disease other/NOS

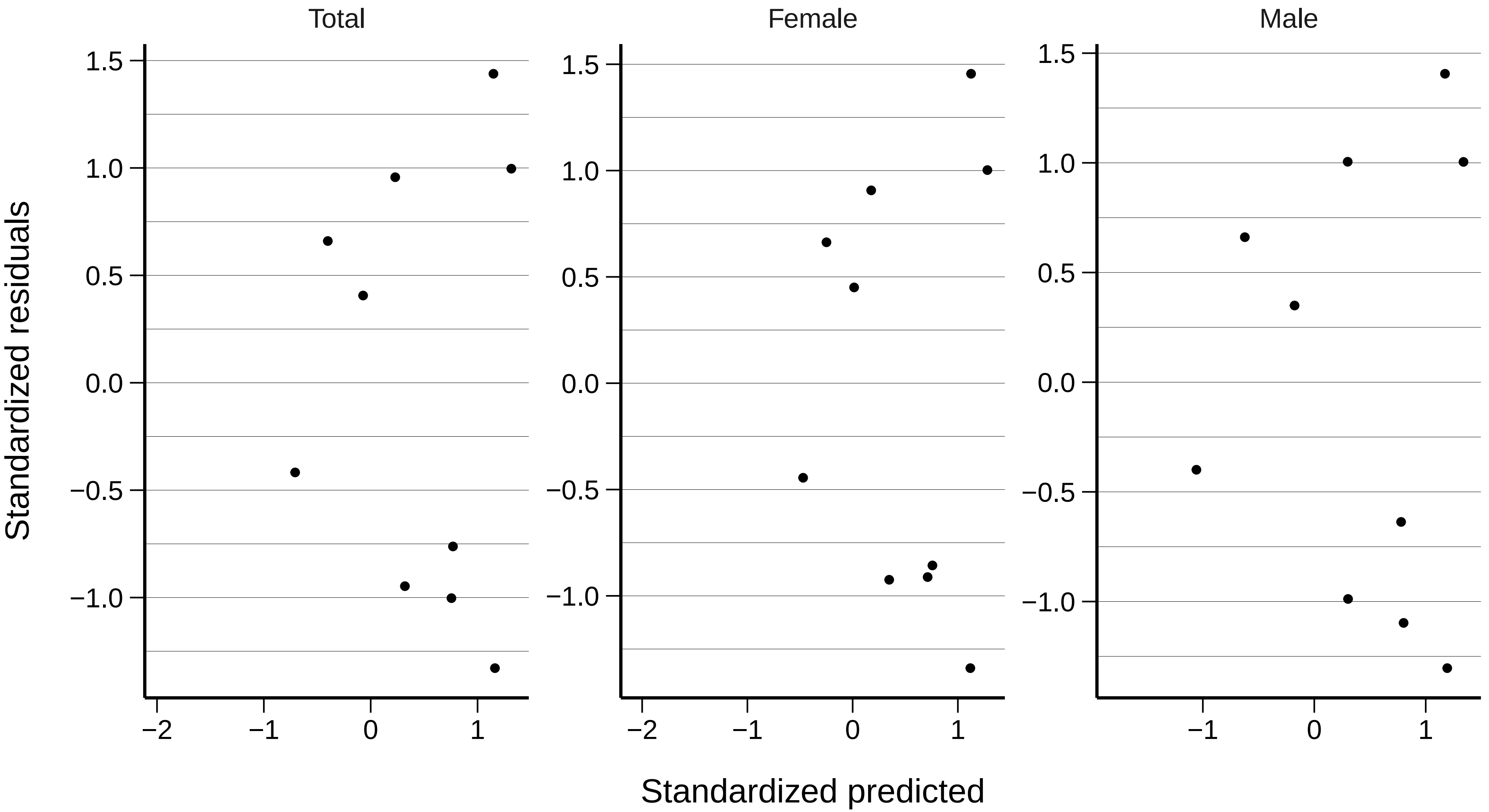

cb. NorSySS: A78 Infectious disease other/NOS

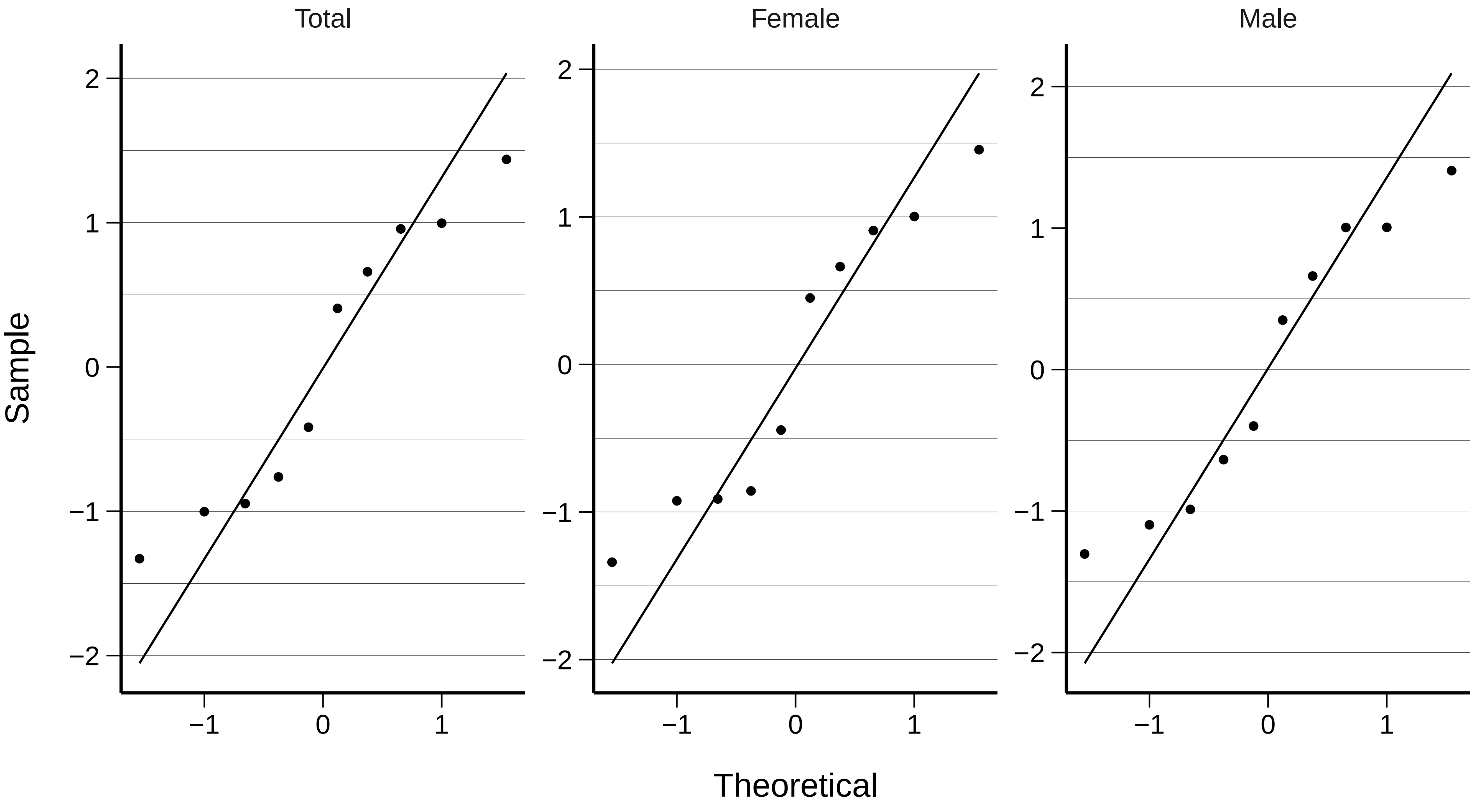

cc. NorSySS: D01 Abdominal pain/cramps general

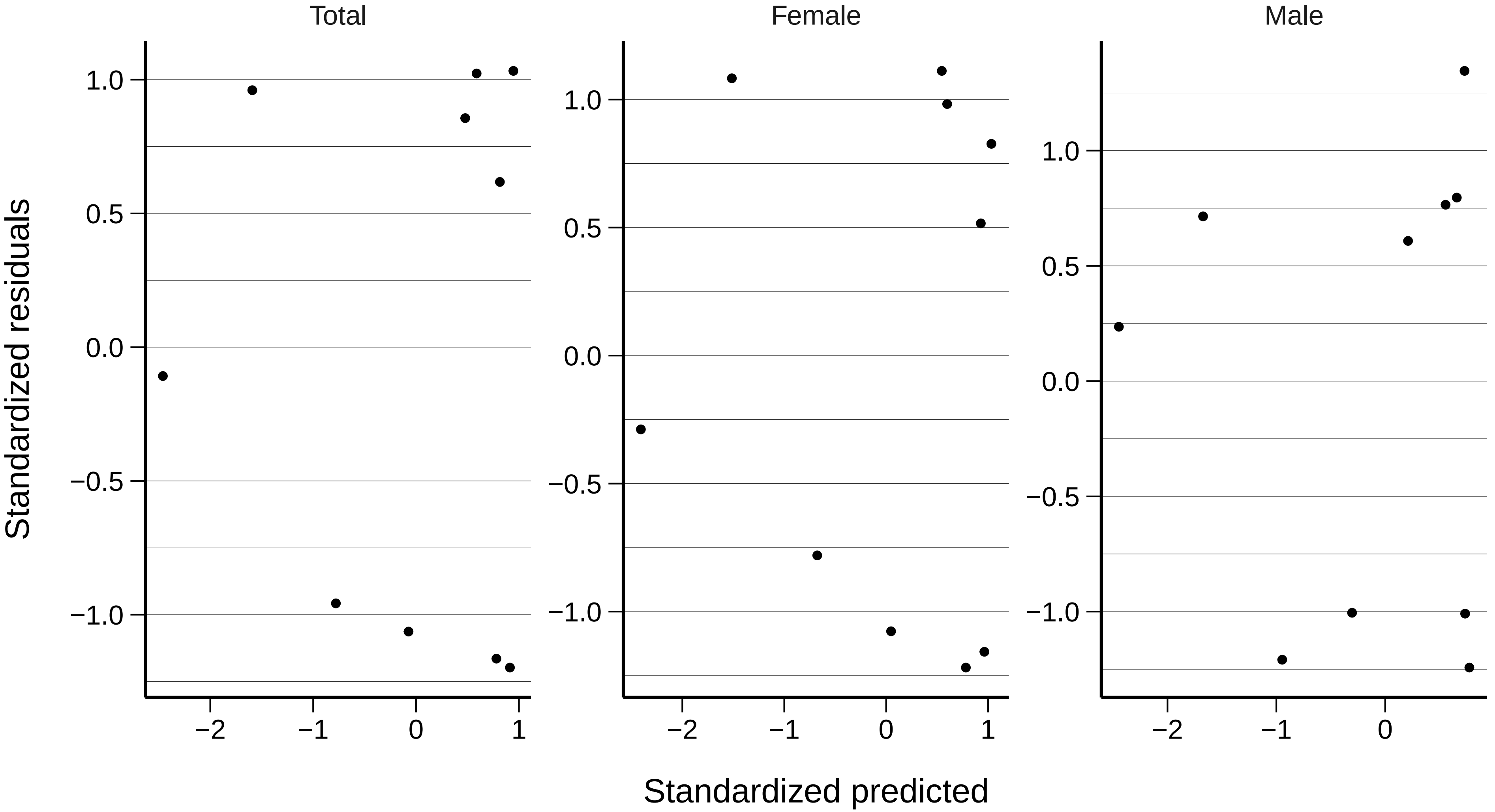

cd. NorSySS: D01 Abdominal pain/cramps general

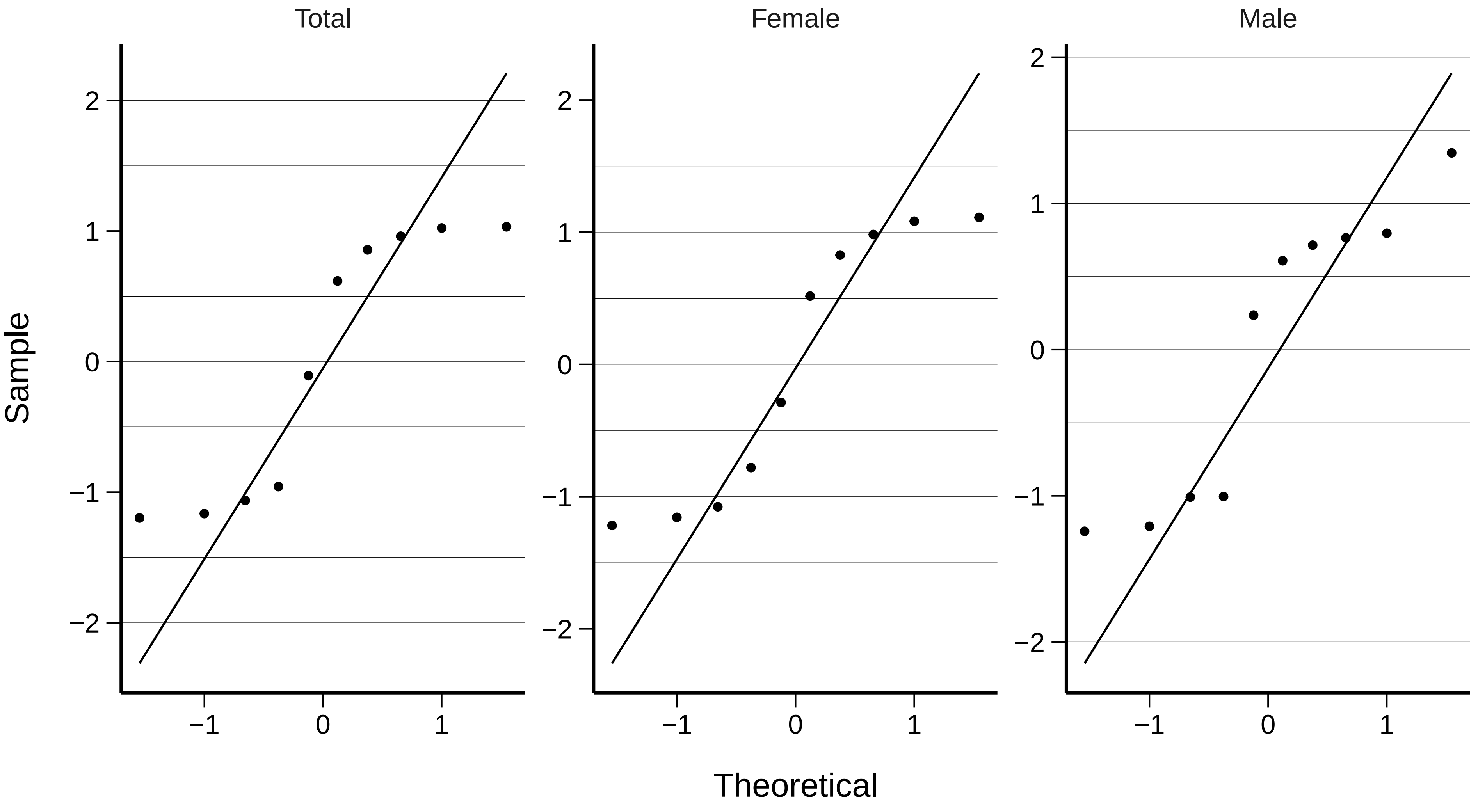

ce. NorSySS: D09 Nausea

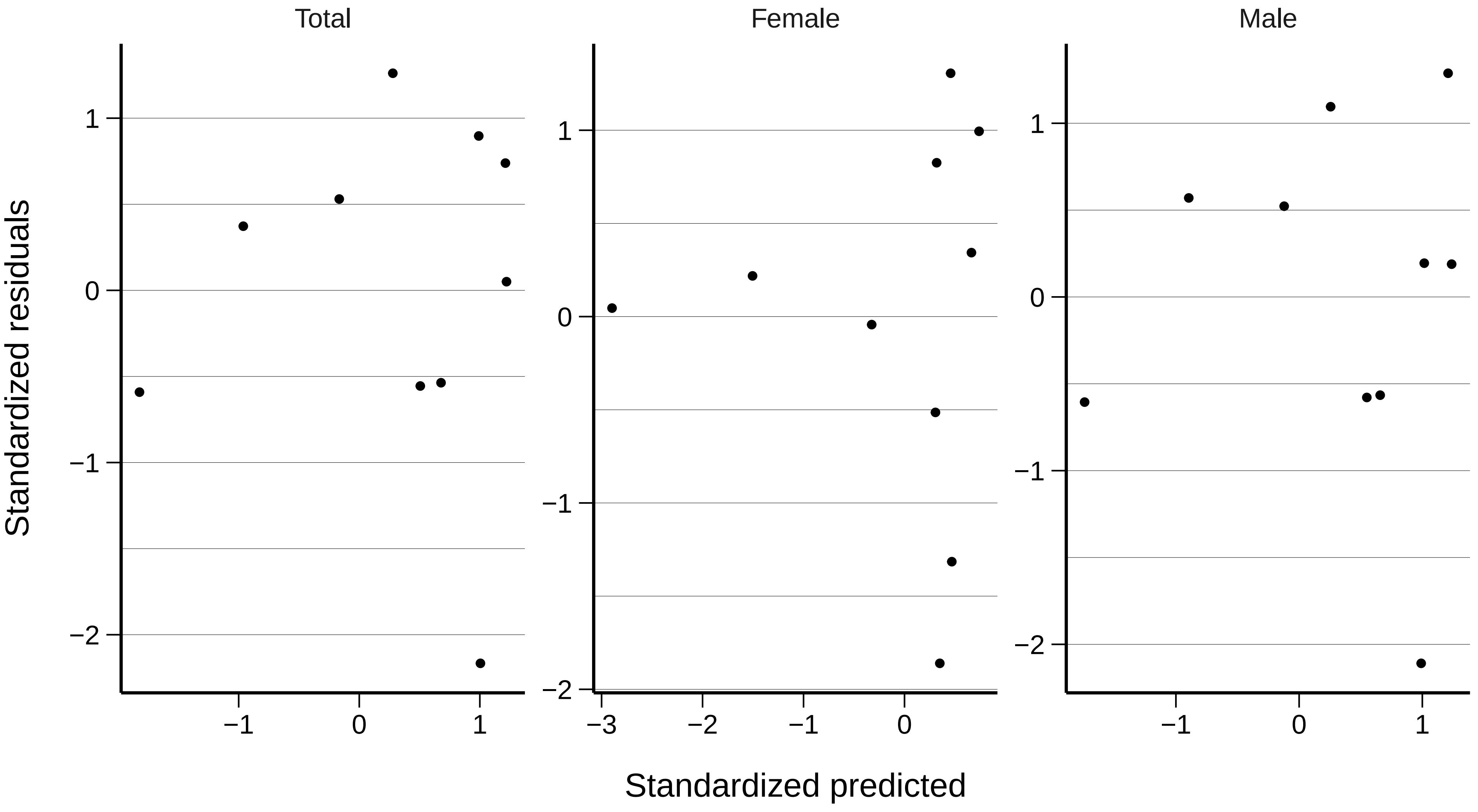

cf. NorSySS: D09 Nausea

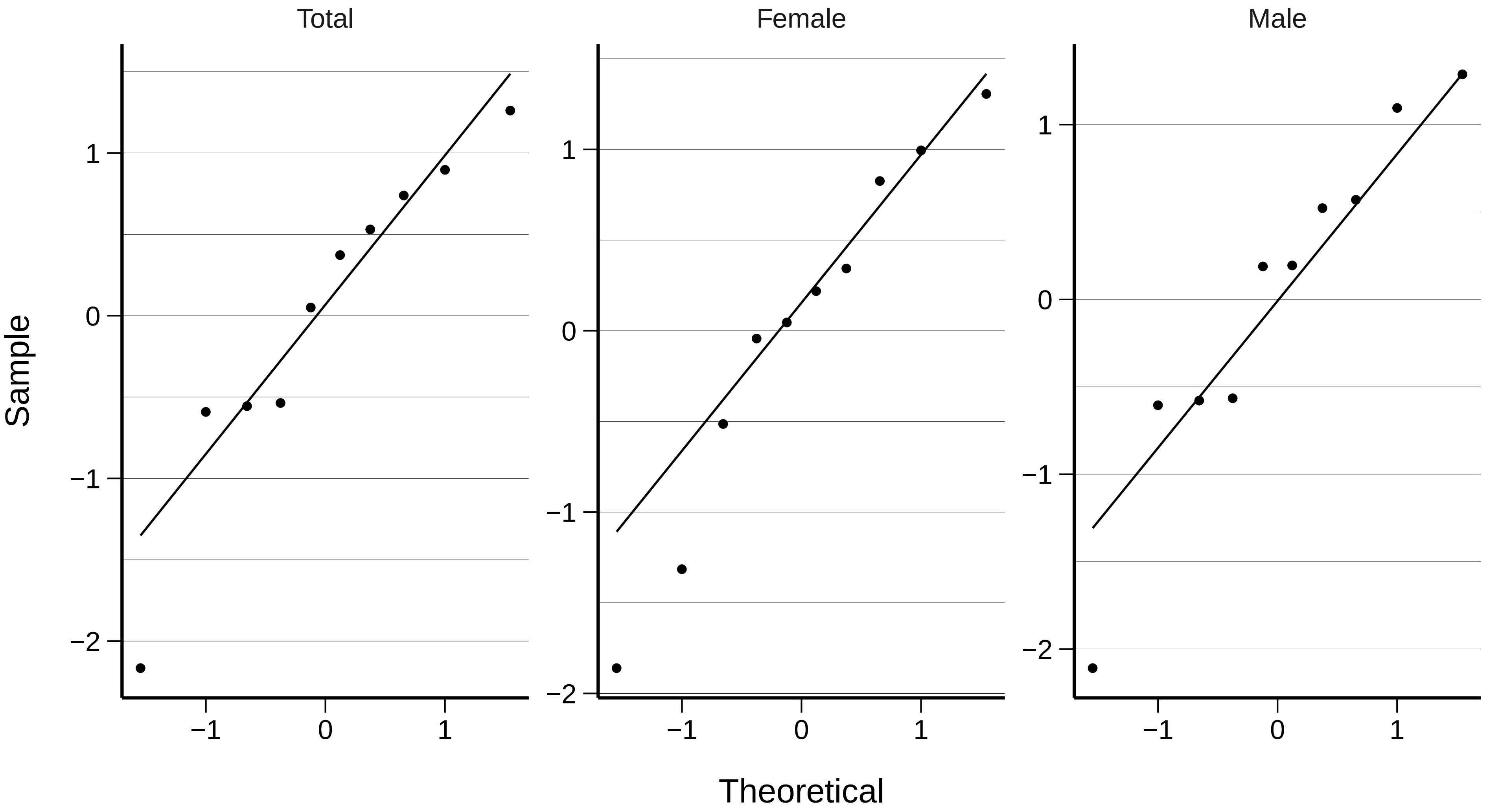

cg. NorSySS: D11 Diarrhea

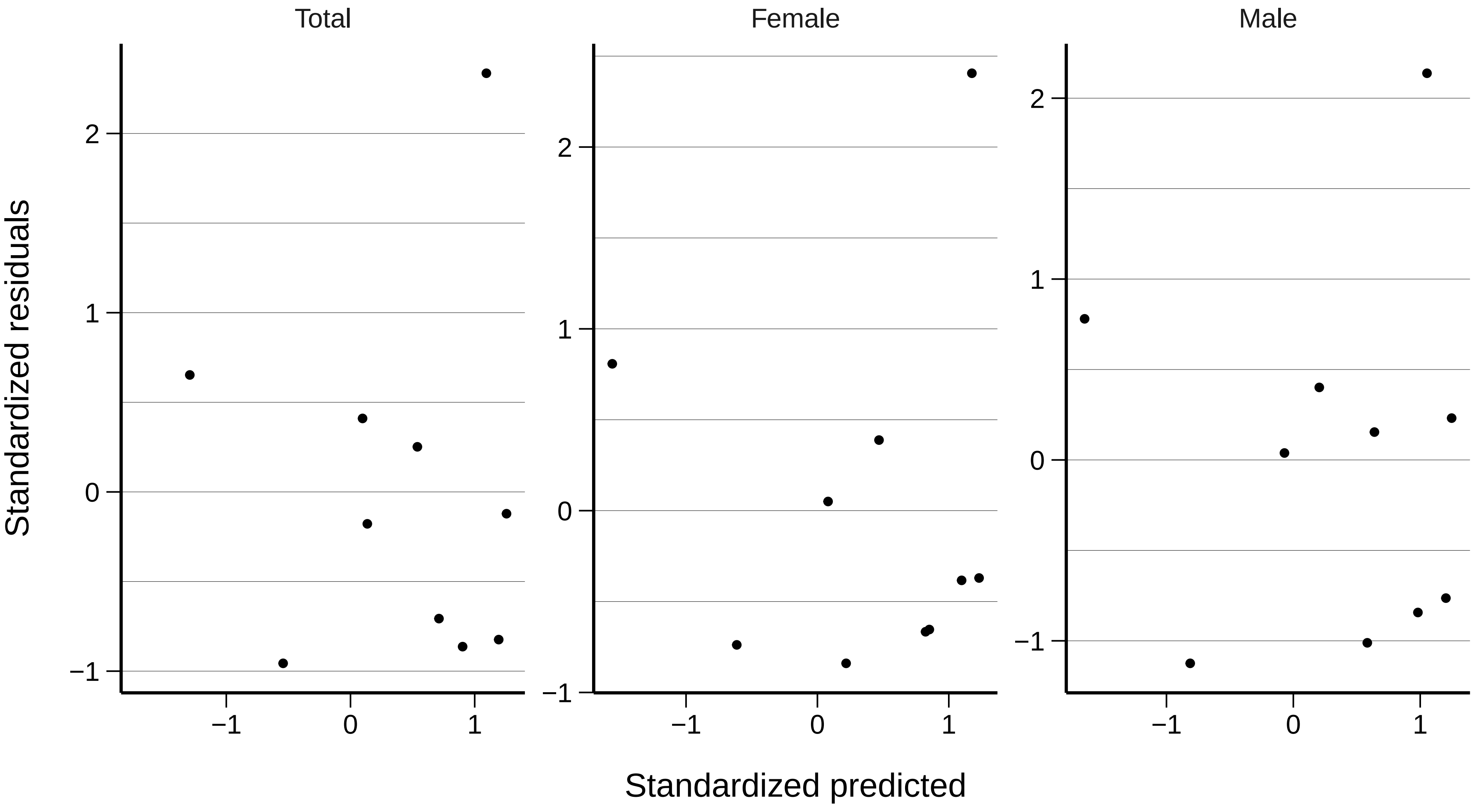

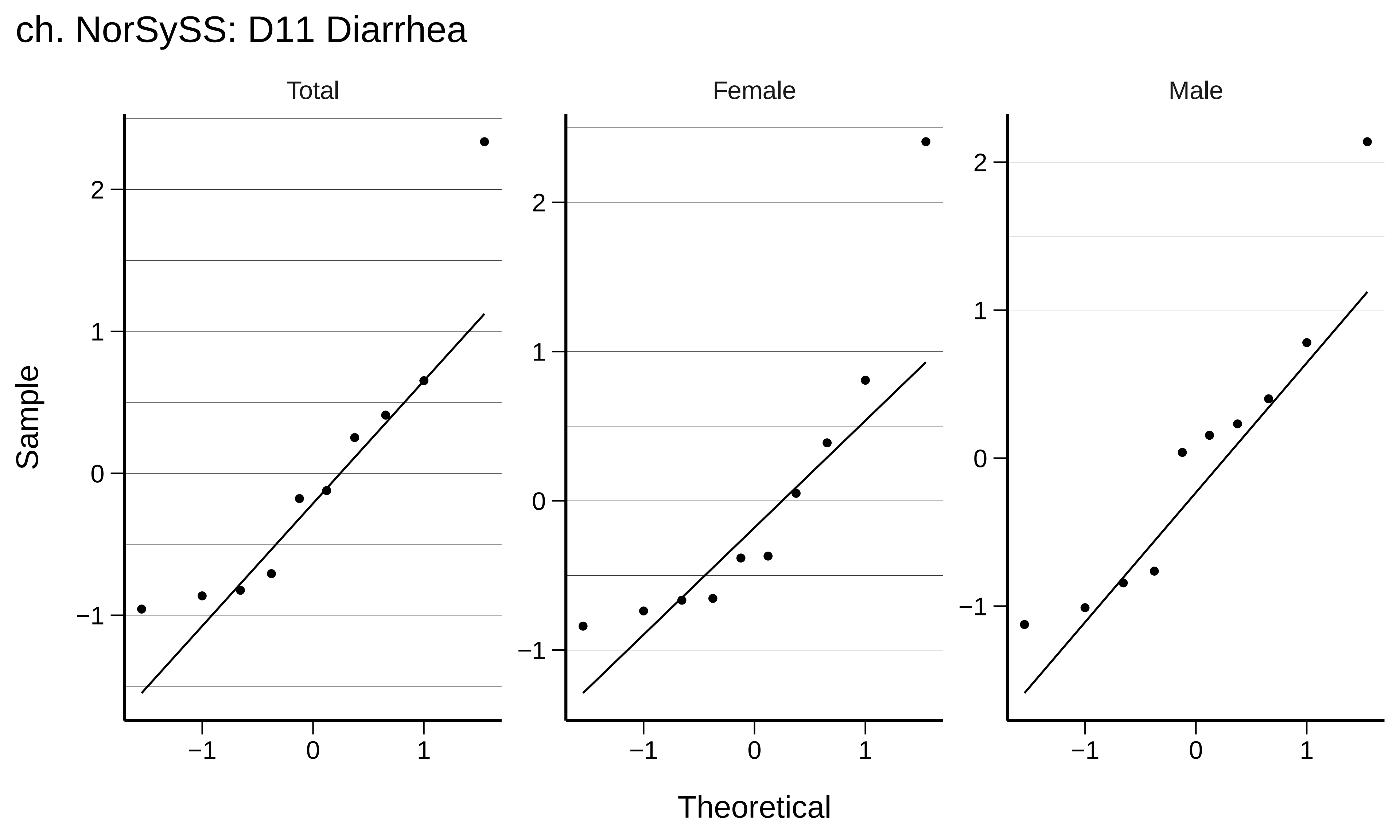
ch. NorSySS: D11 Diarrhea
Total
Female
Male
Sample
Theoretical
2
1
0
−1
−1
0
1

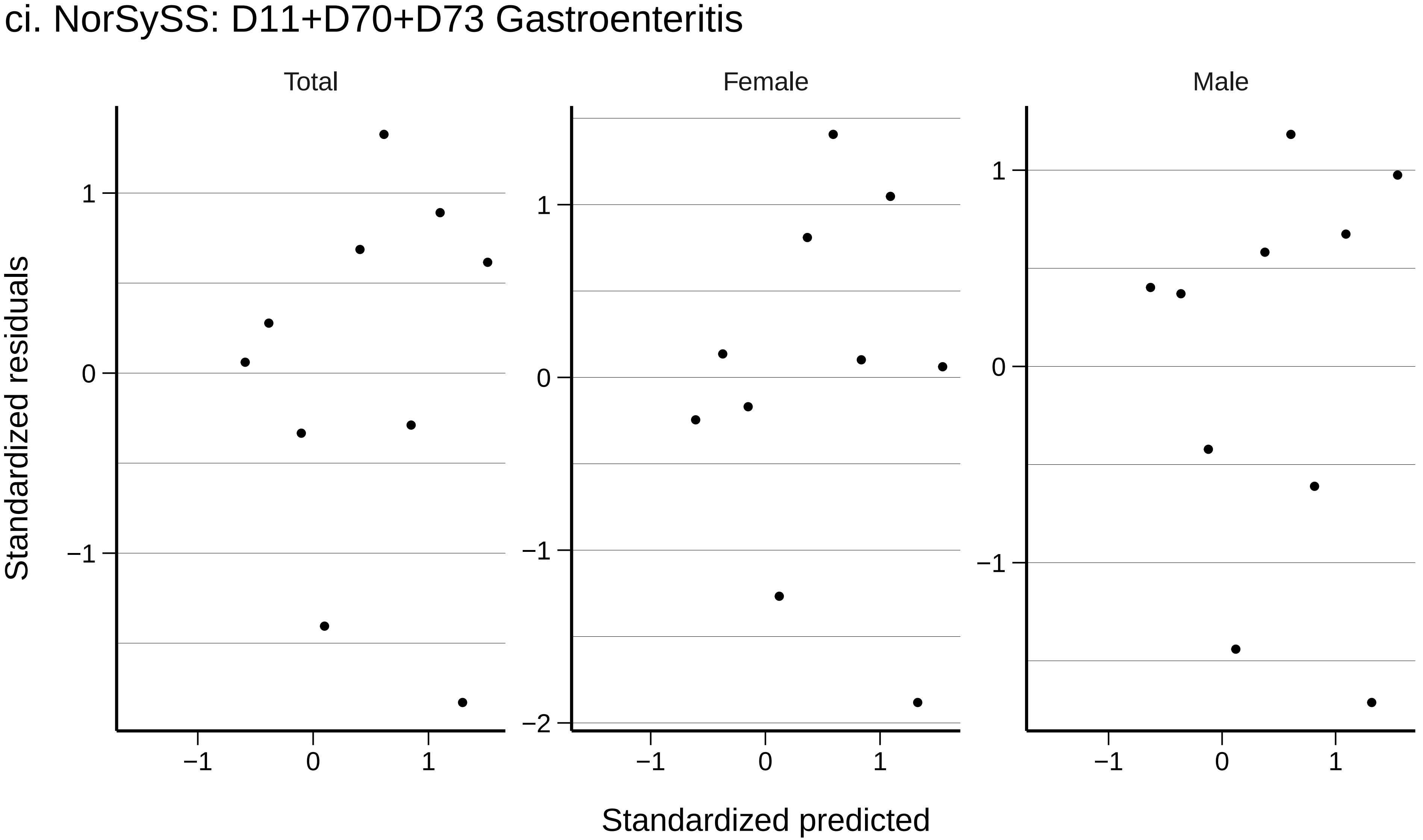
ci. NorSySS: D11+D70+D73 Gastroenteritis
Total
Female
Male
Standardized residuals
Standardized predicted
1
0
−1
−2
−1
0
1

cj. NorSySS: D11+D70+D73 Gastroenteritis

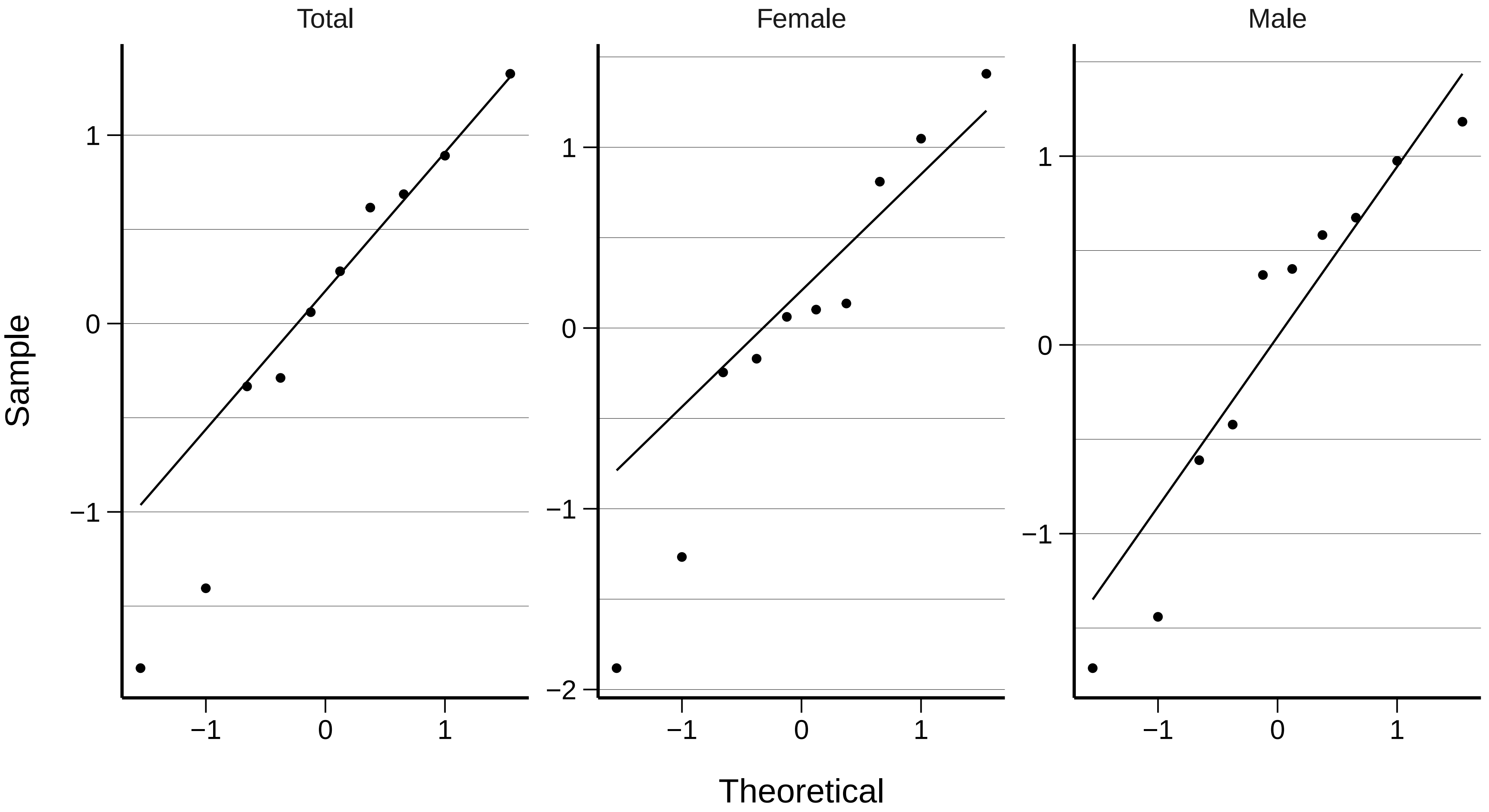

ck. NorSySS: D18 Change feces/bowel movements

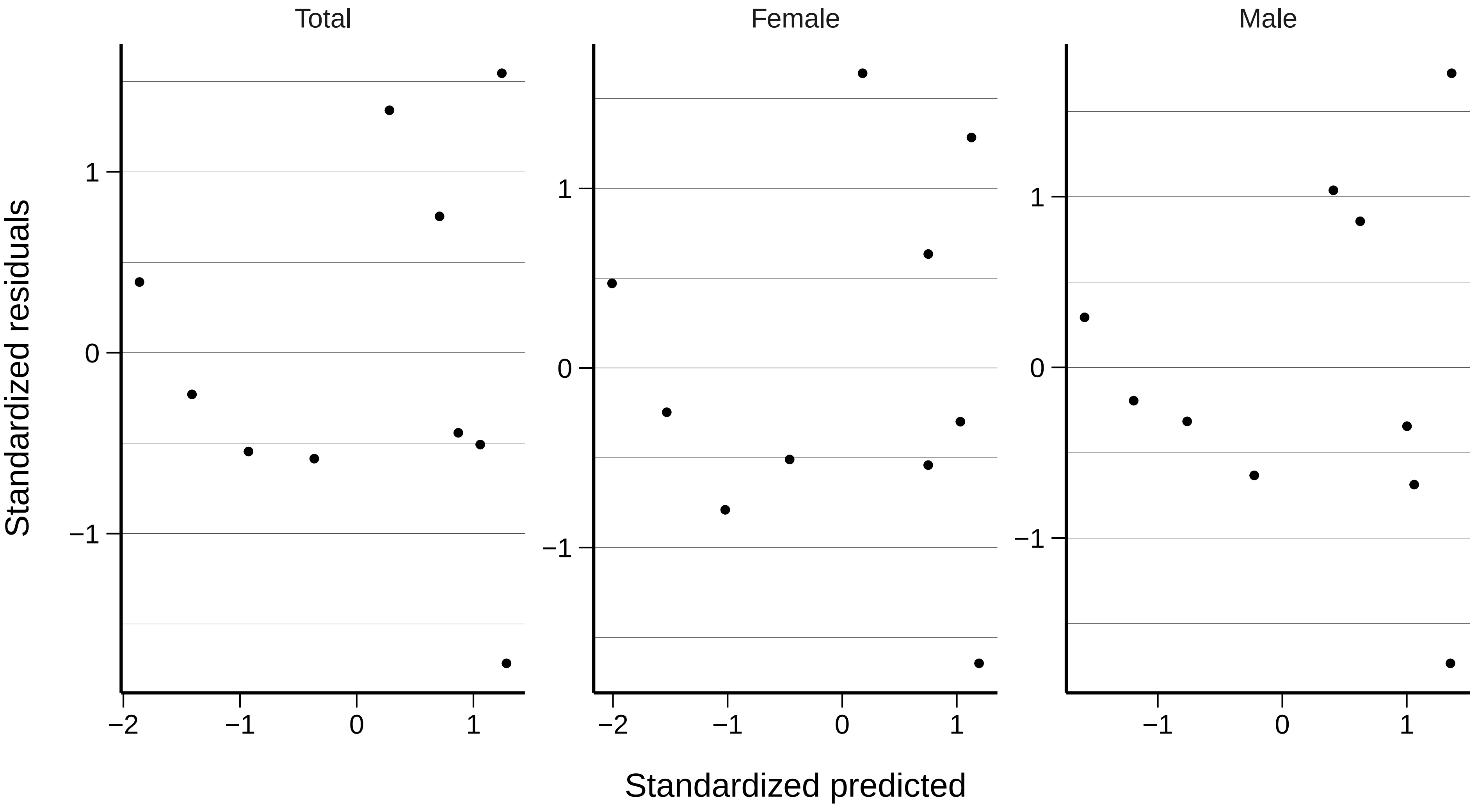

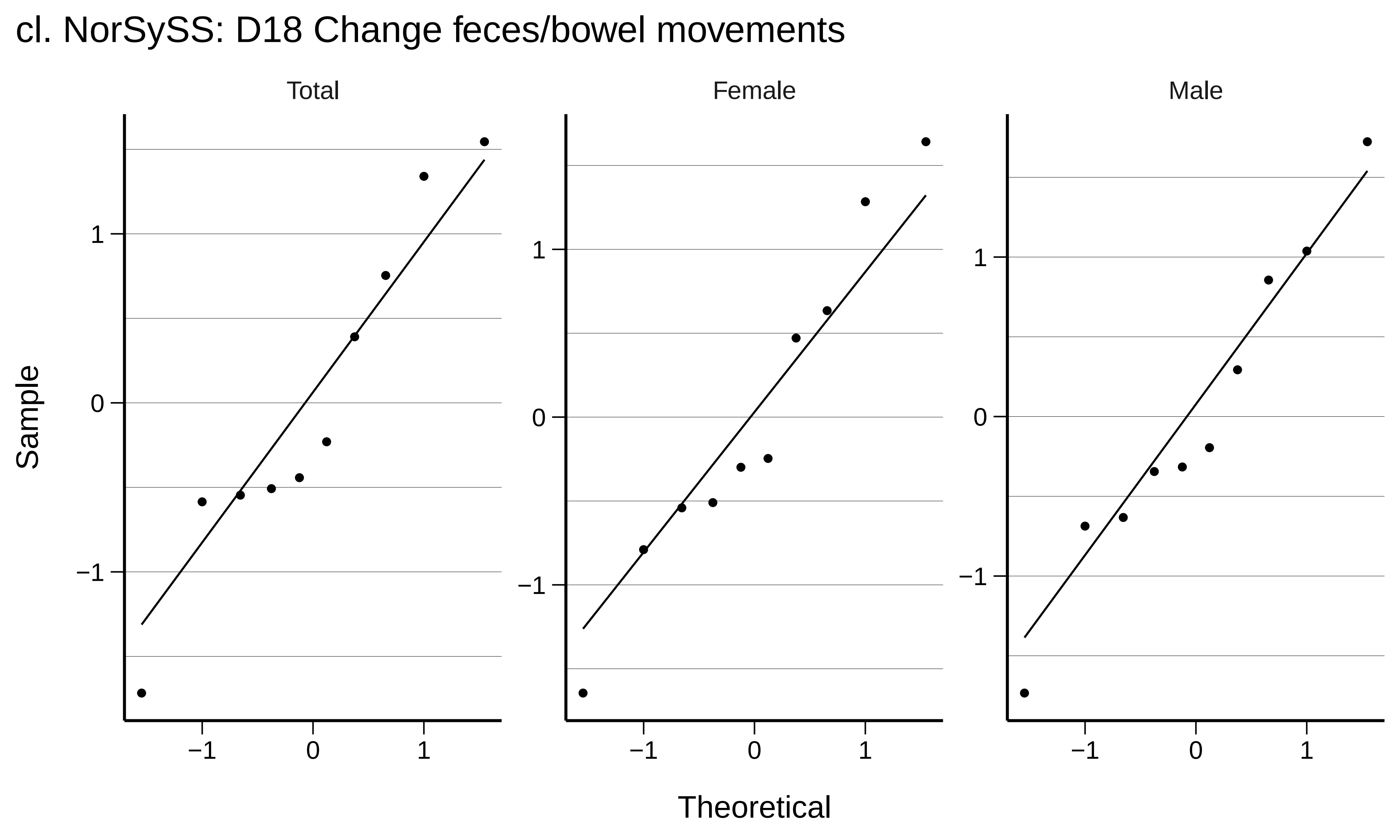

cl. NorSySS: D18 Change feces/bowel movements
Total
Female
Male
Sample
Theoretical
1
0
−1

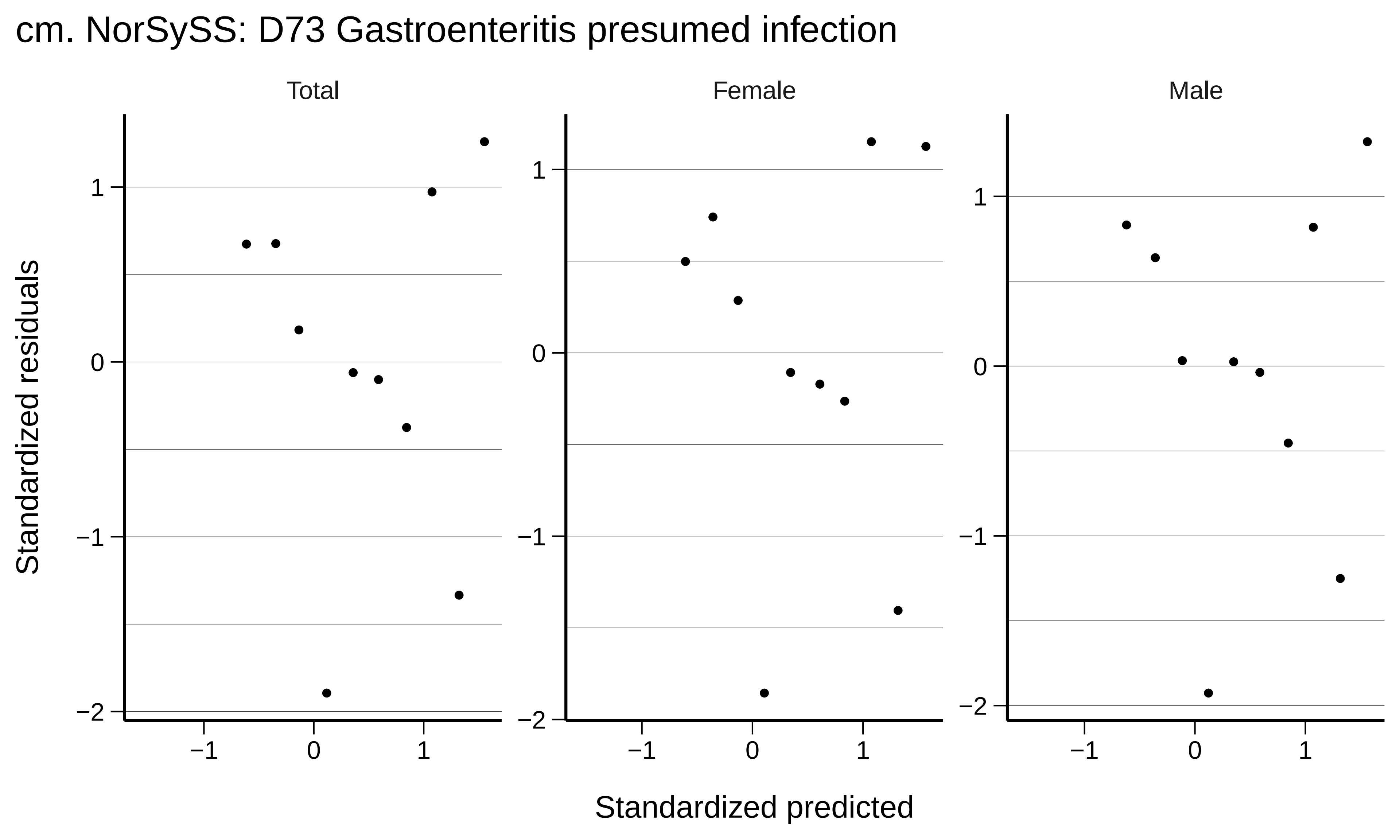
cm. NorSySS: D73 Gastroenteritis presumed infection
Total
Female
Male
Standardized residuals
Standardized predicted
1
0
−1
−2

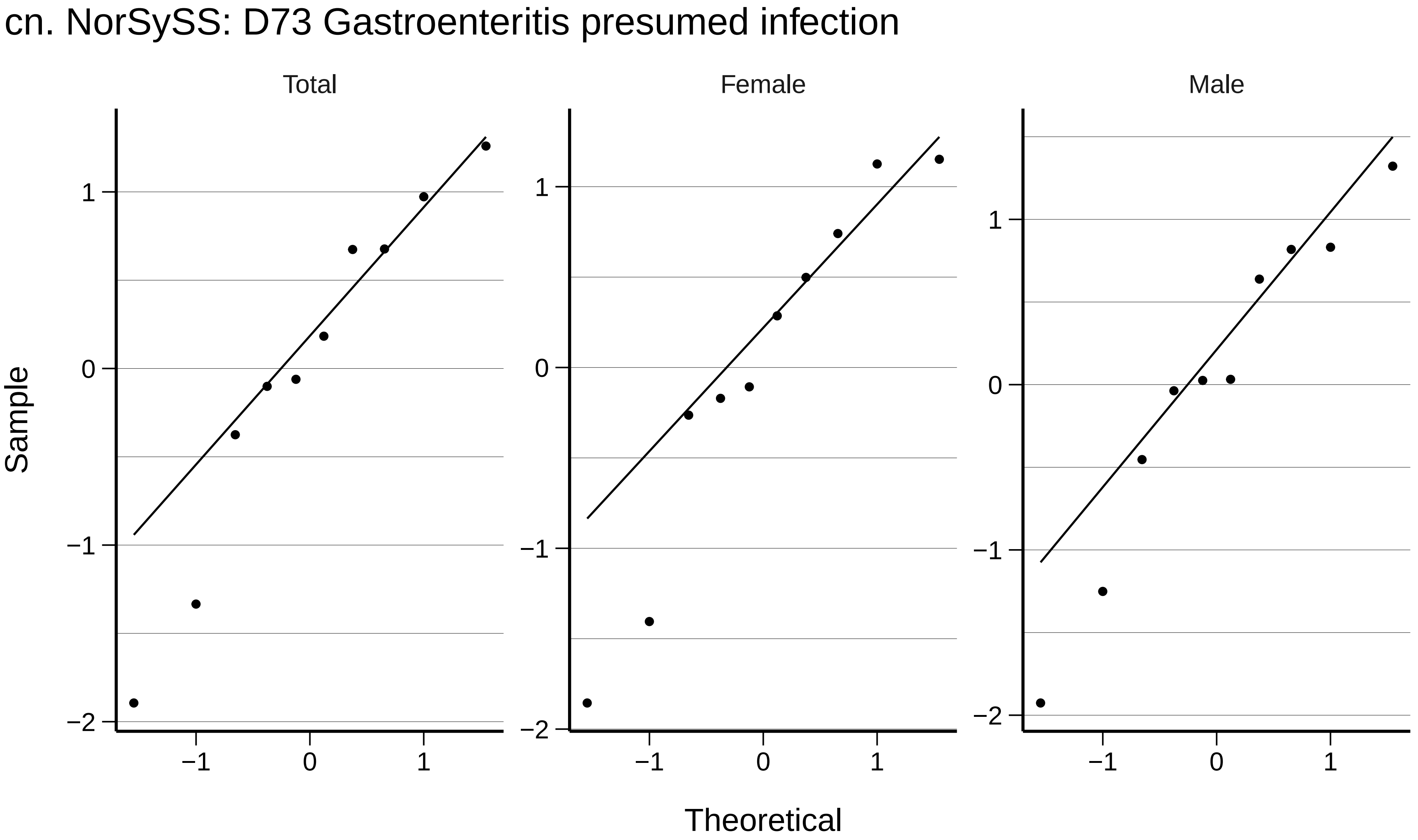
cn. NorSySS: D73 Gastroenteritis presumed infection
Total
Female
Male
Sample
Theoretical
1
0
−1
−2
−1
0
1

co. NorSySS: D99 Disease digestive system, other

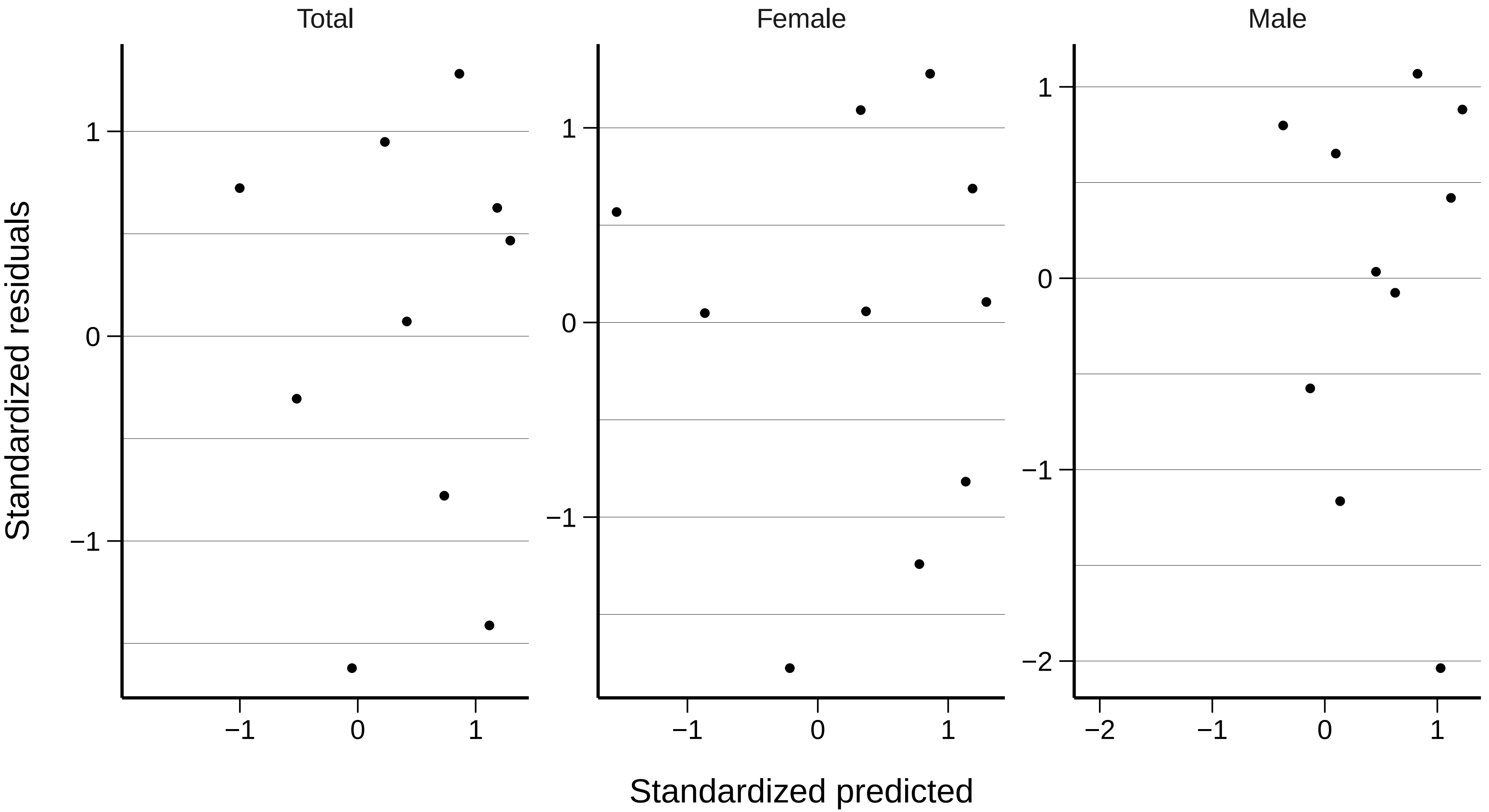

cp. NorSySS: D99 Disease digestive system, other

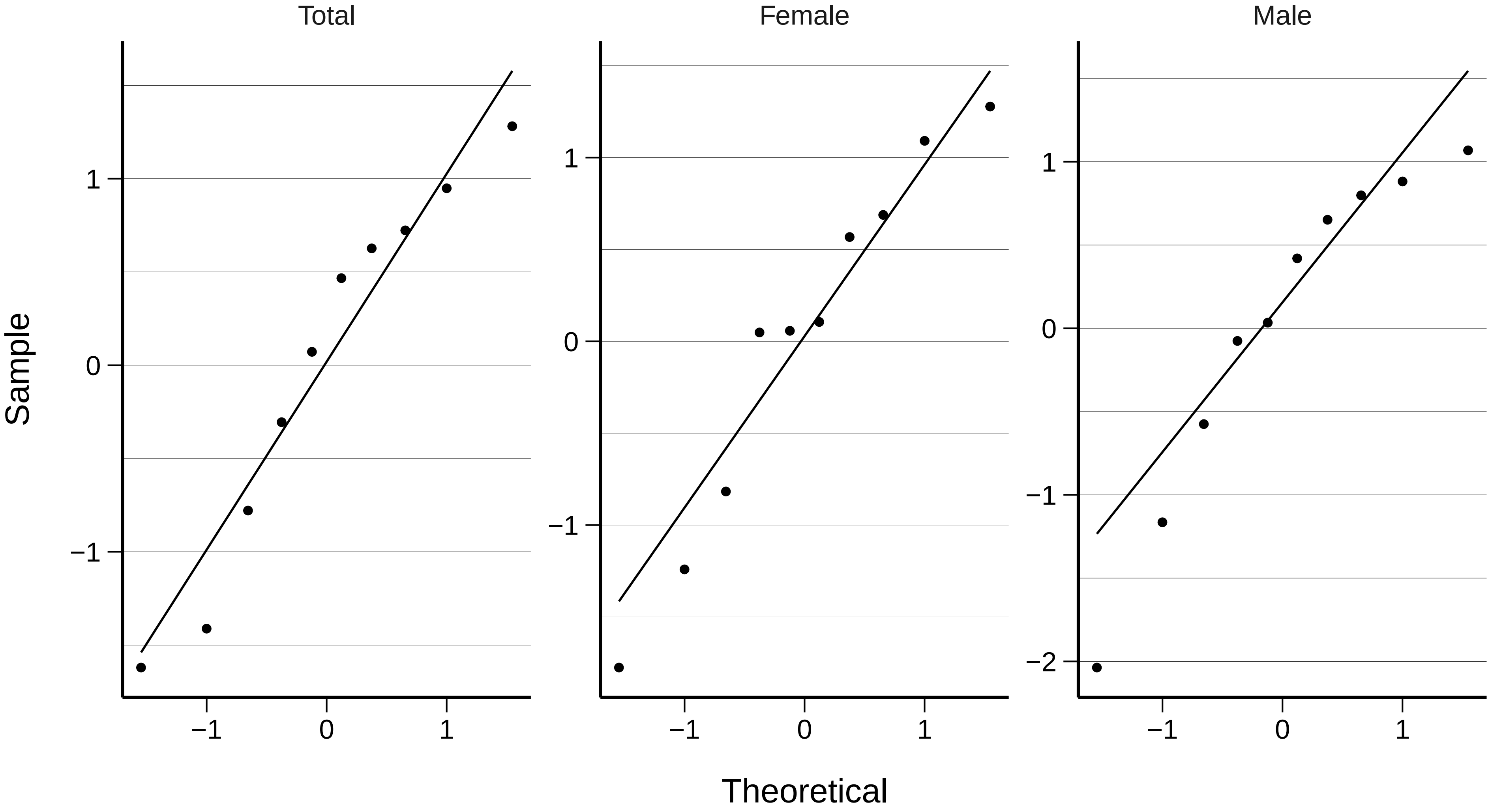

cq. NorSySS: H01 Ear pain/earache

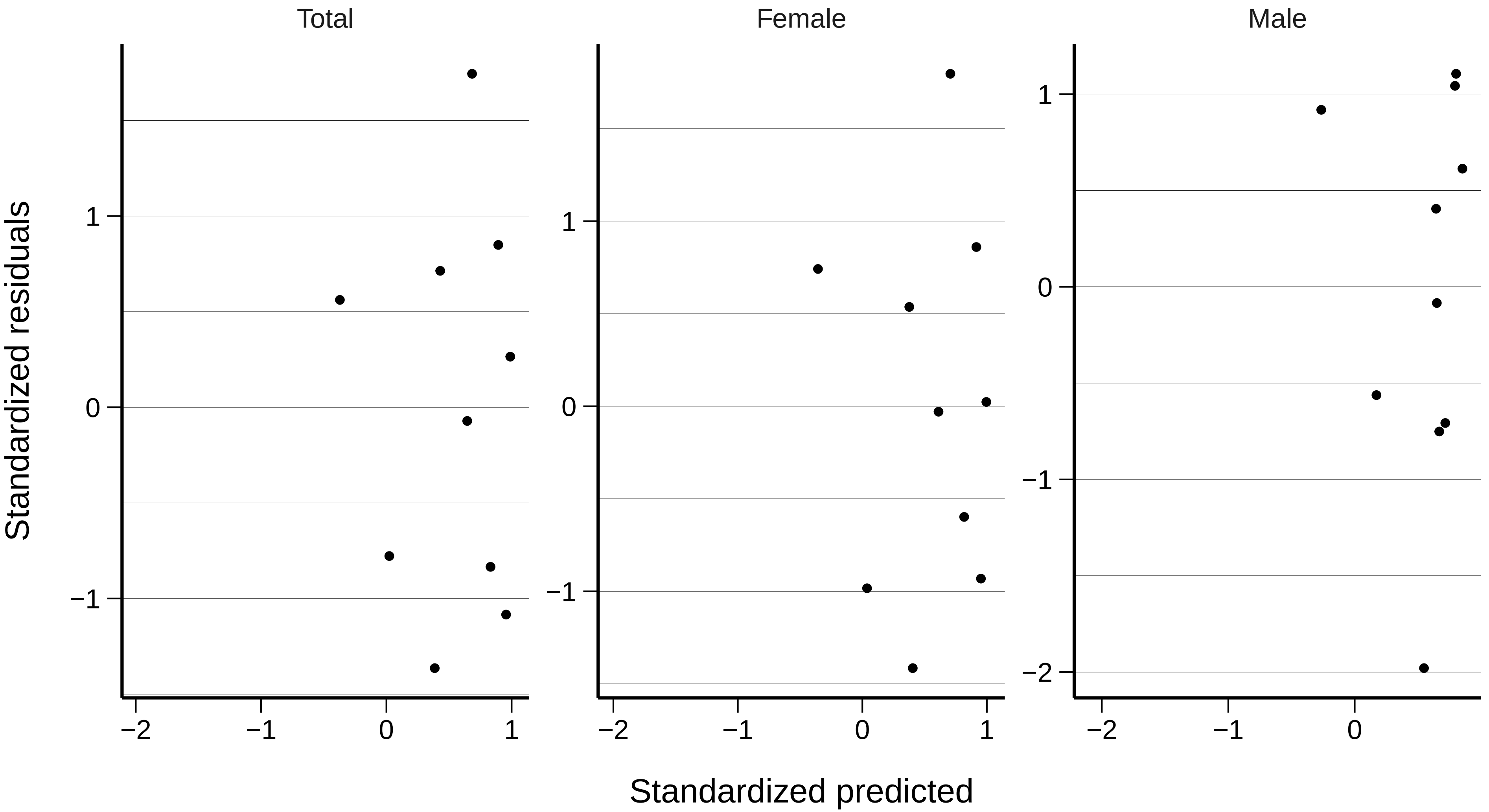

cr. NorSySS: H01 Ear pain/earache

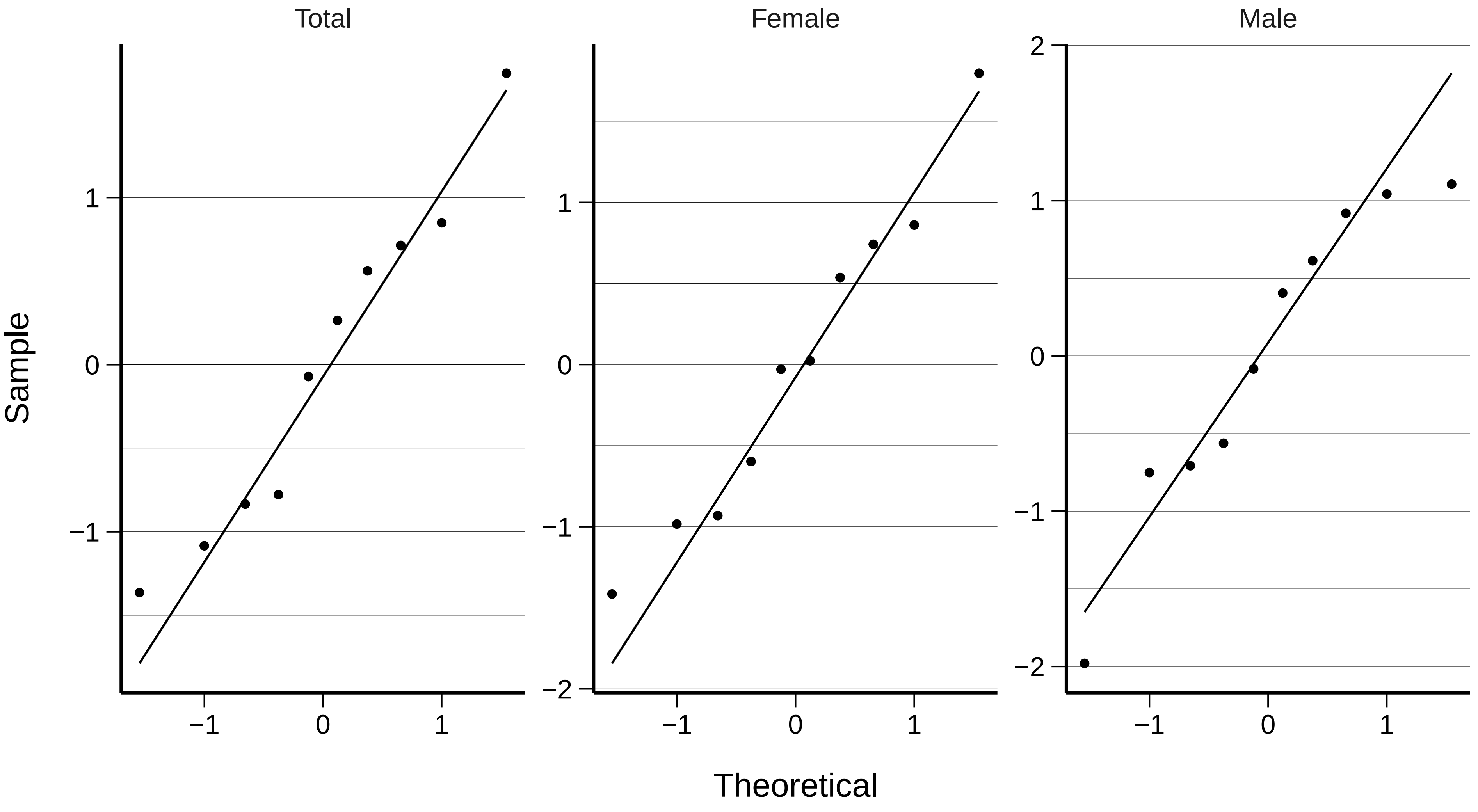

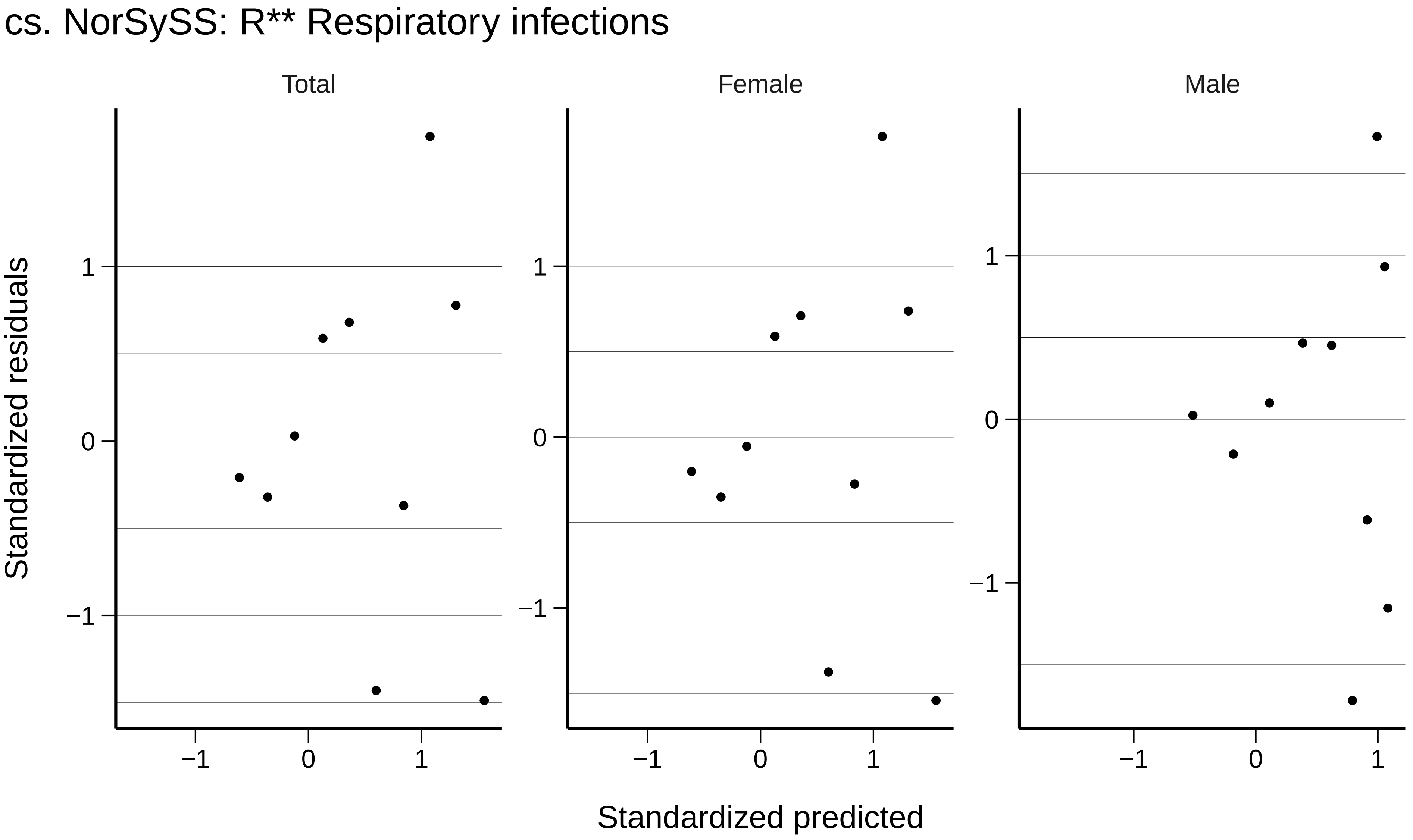

cs. NorSySS: R** Respiratory infections
Total
Female
Male
Standardized residuals
Standardized predicted
1
0
−1

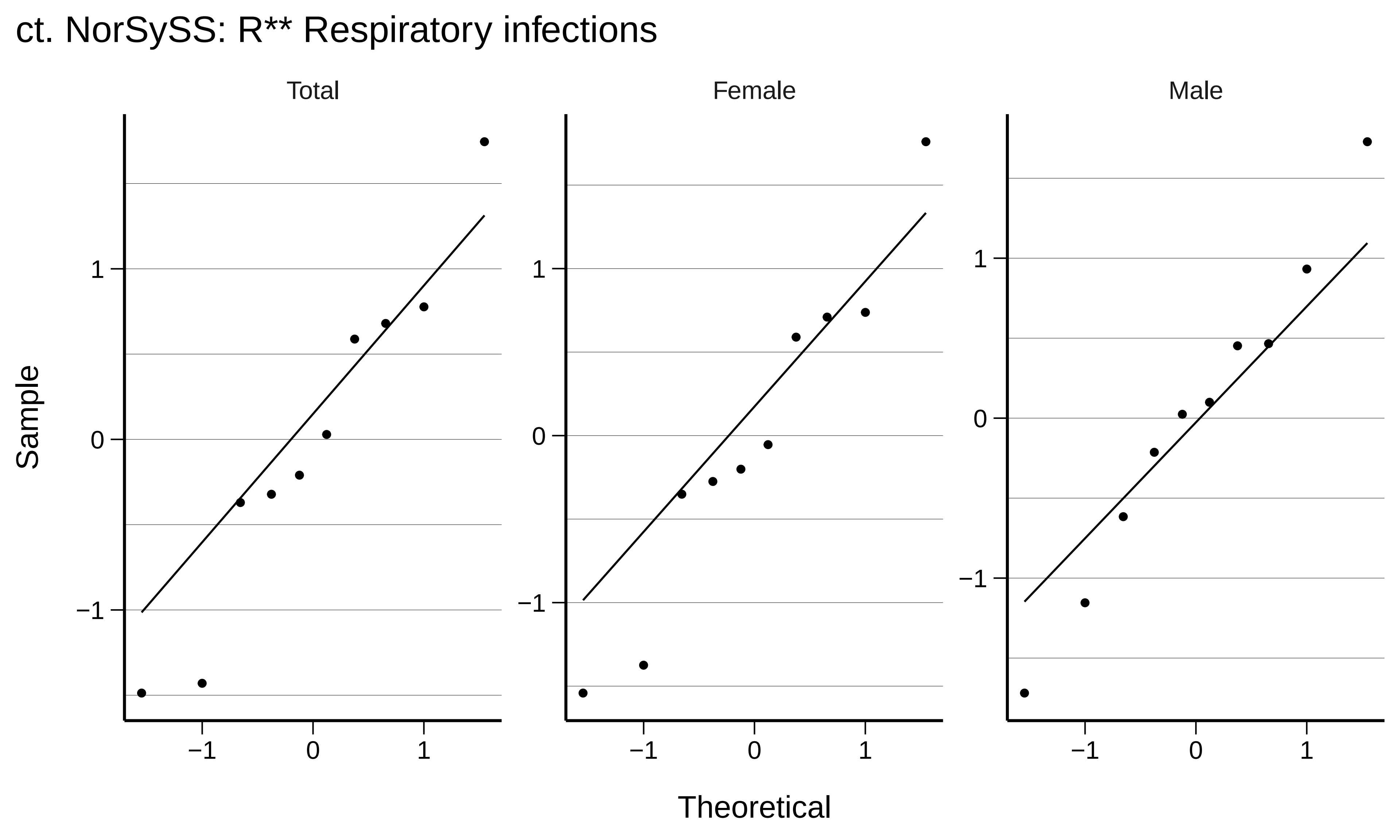
ct. NorSySS: R** Respiratory infections
Total
Female
Male
Sample
Theoretical
1
0
−1

cu. NorSySS: R21 Throat symptom/complaint

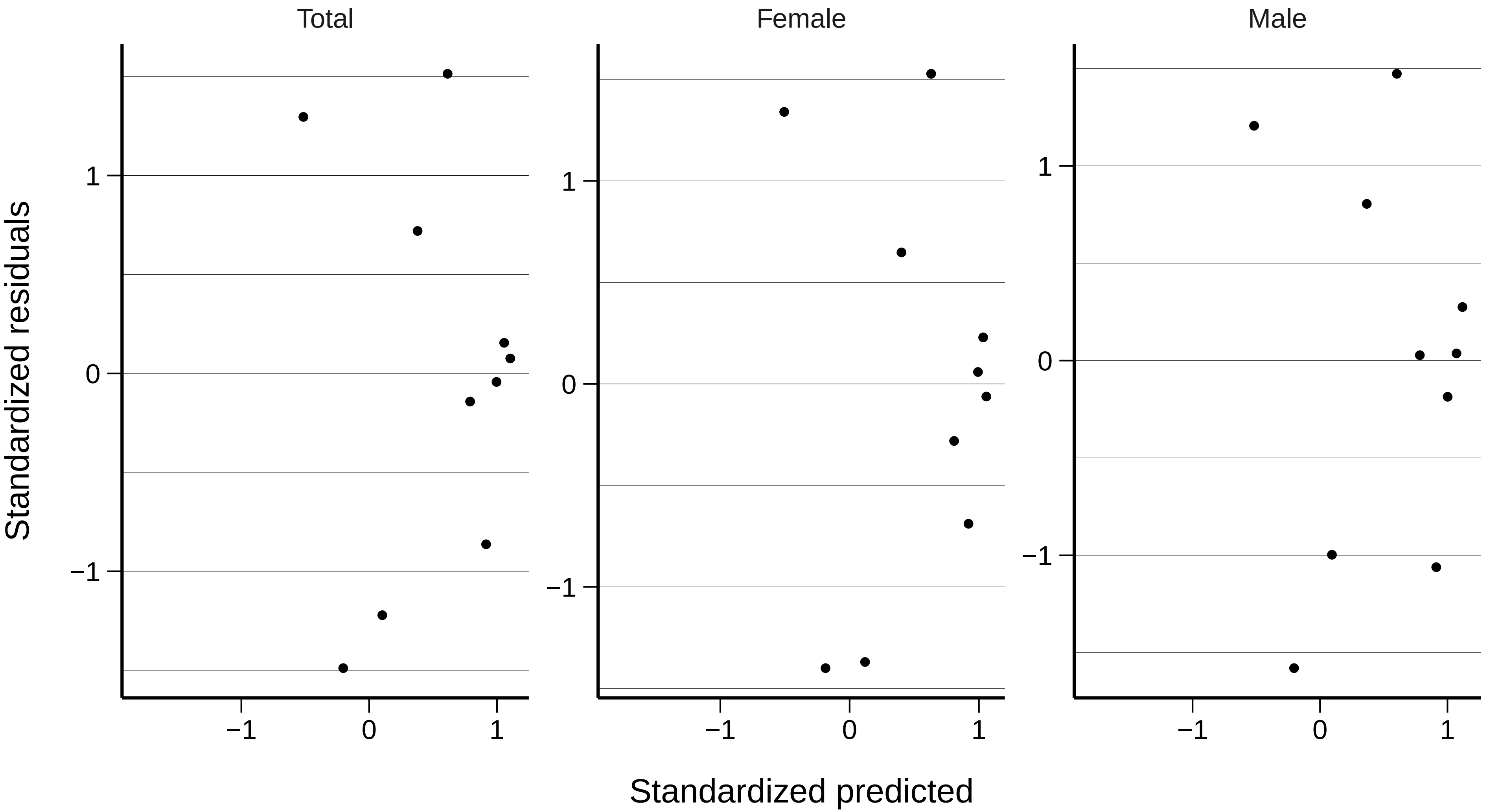

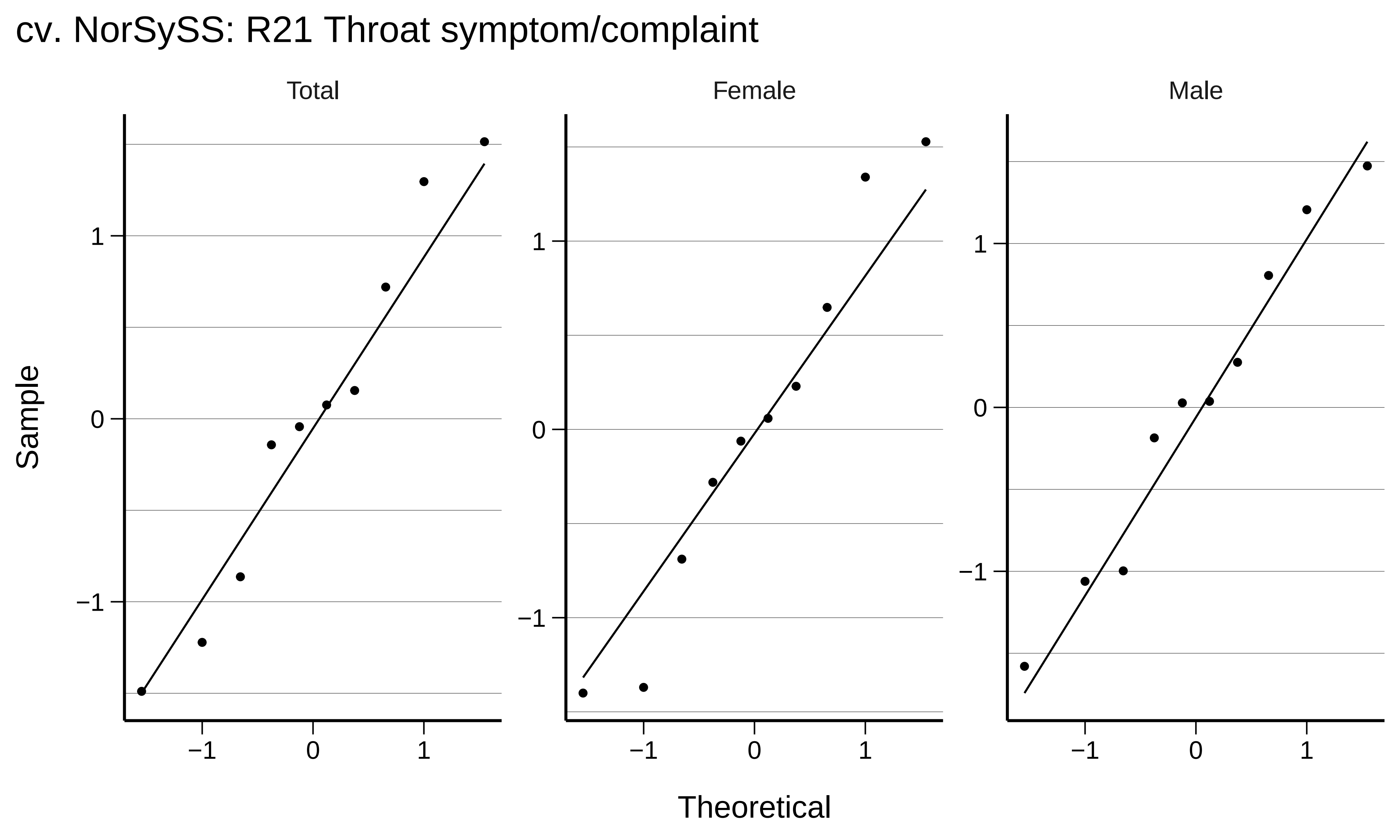
cv. NorSySS: R21 Throat symptom/complaint
Total
Female
Male
Sample
Theoretical
1
0
−1
−1
0
1

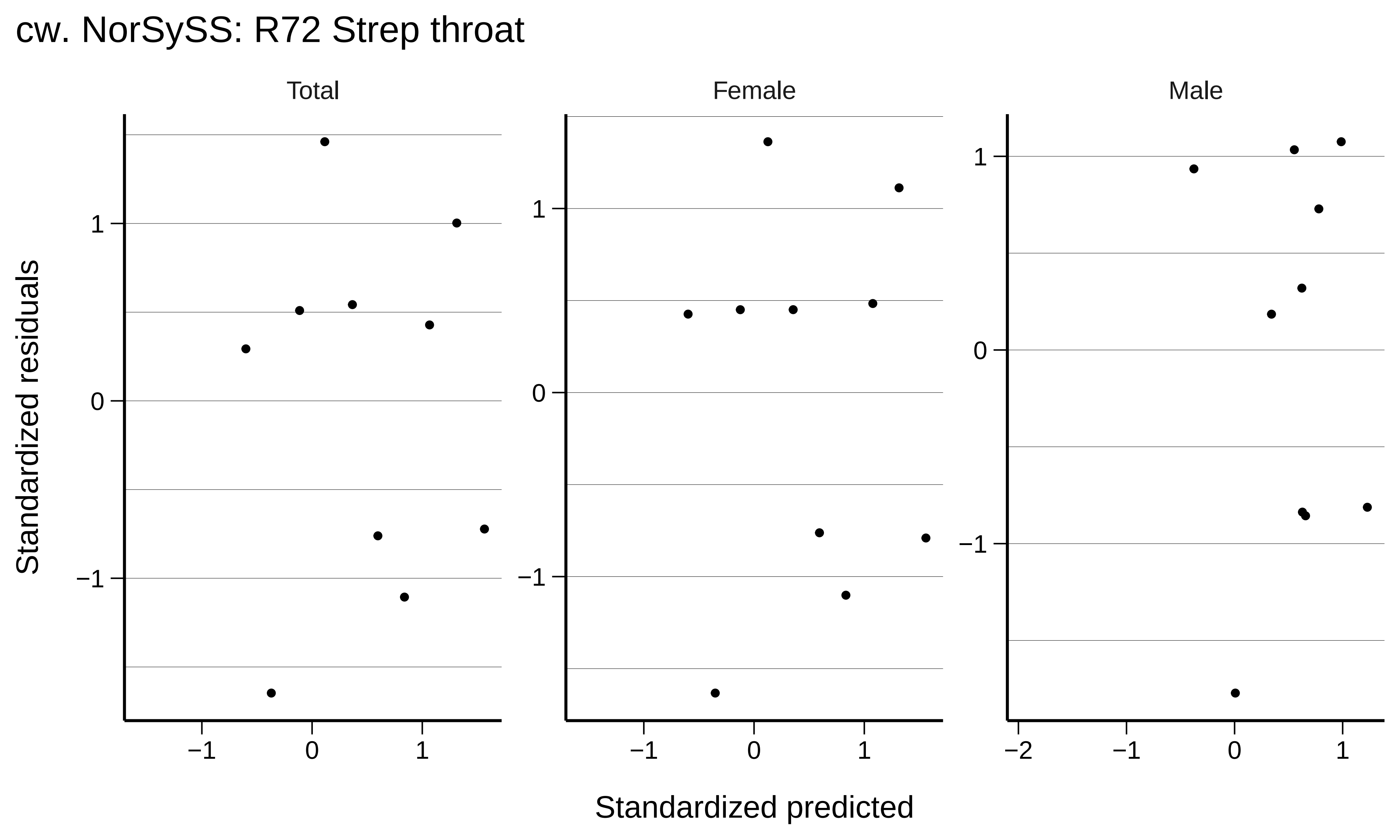
cw. NorSySS: R72 Strep throat
Total
Female
Male
Standardized residuals
Standardized predicted
1
0
−1
−2

cx. NorSySS: R72 Strep throat

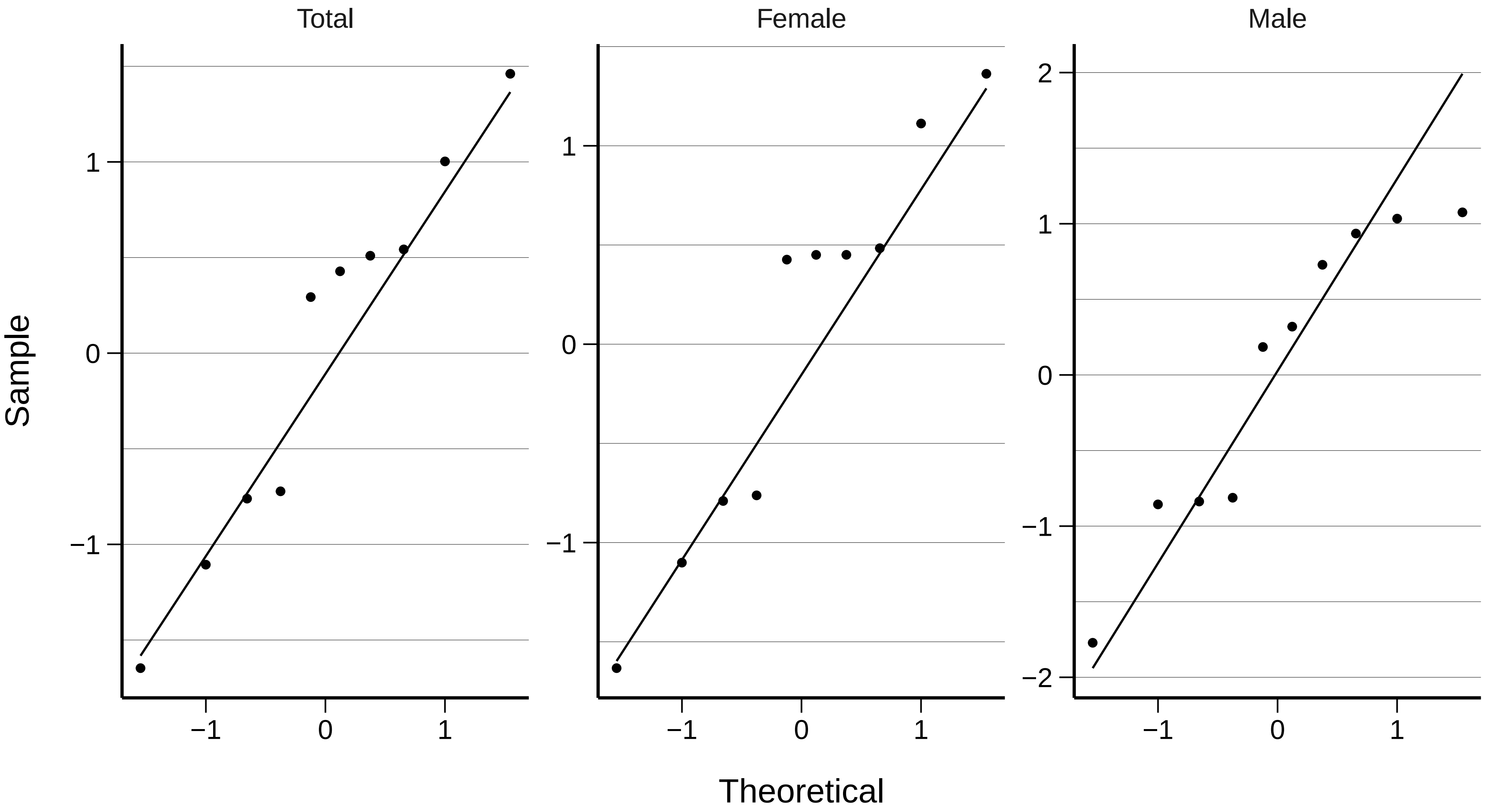

cy. NorSySS: R75 Sinusitis acute/chronic

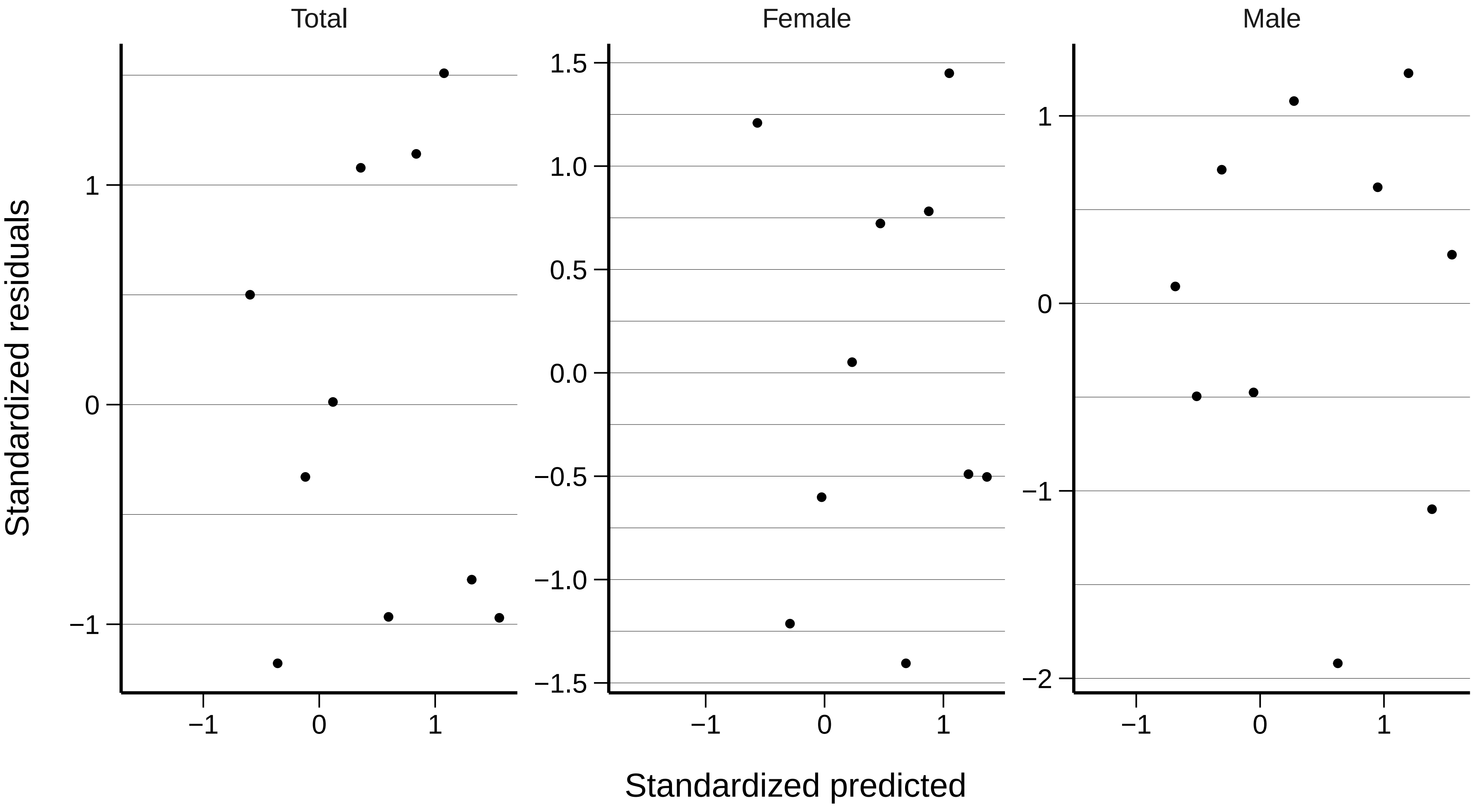

cz. NorSySS: R75 Sinusitis acute/chronic

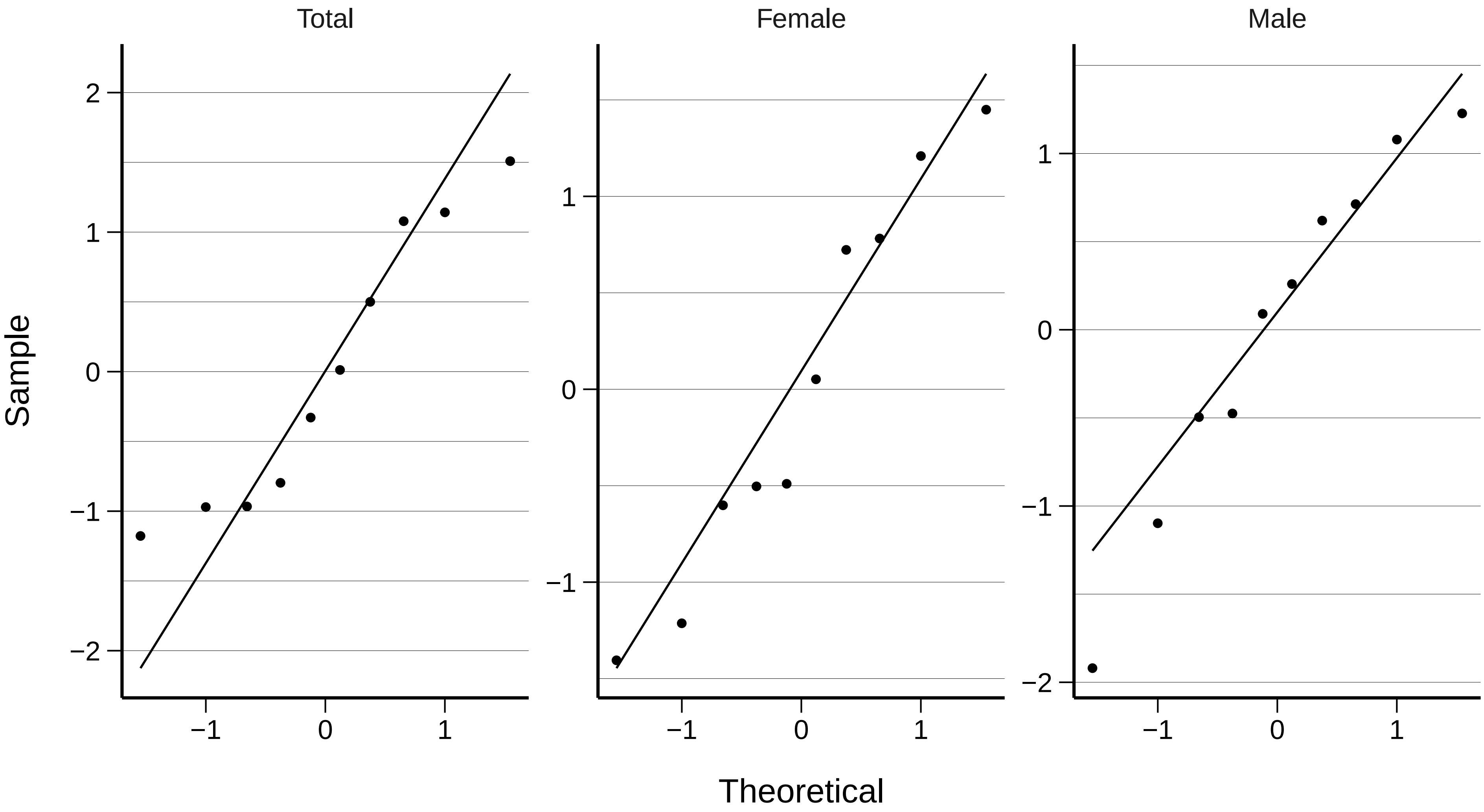

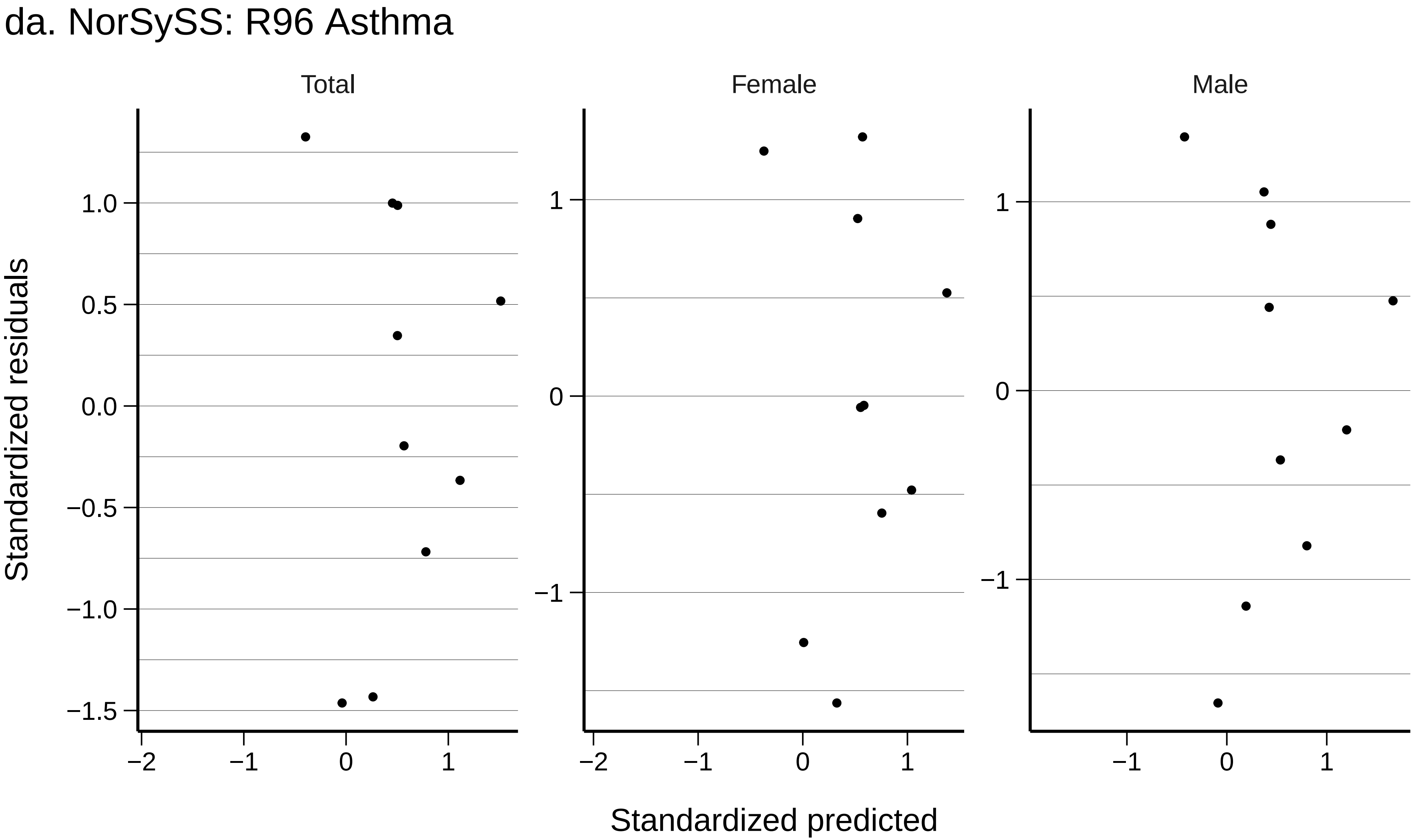
da. NorSySS: R96 Asthma
Total
Female
Male
Standardized residuals
Standardized predicted
1.0
0.5
0.0
−0.5
−1.0
−1.5
1
0
−1
−2

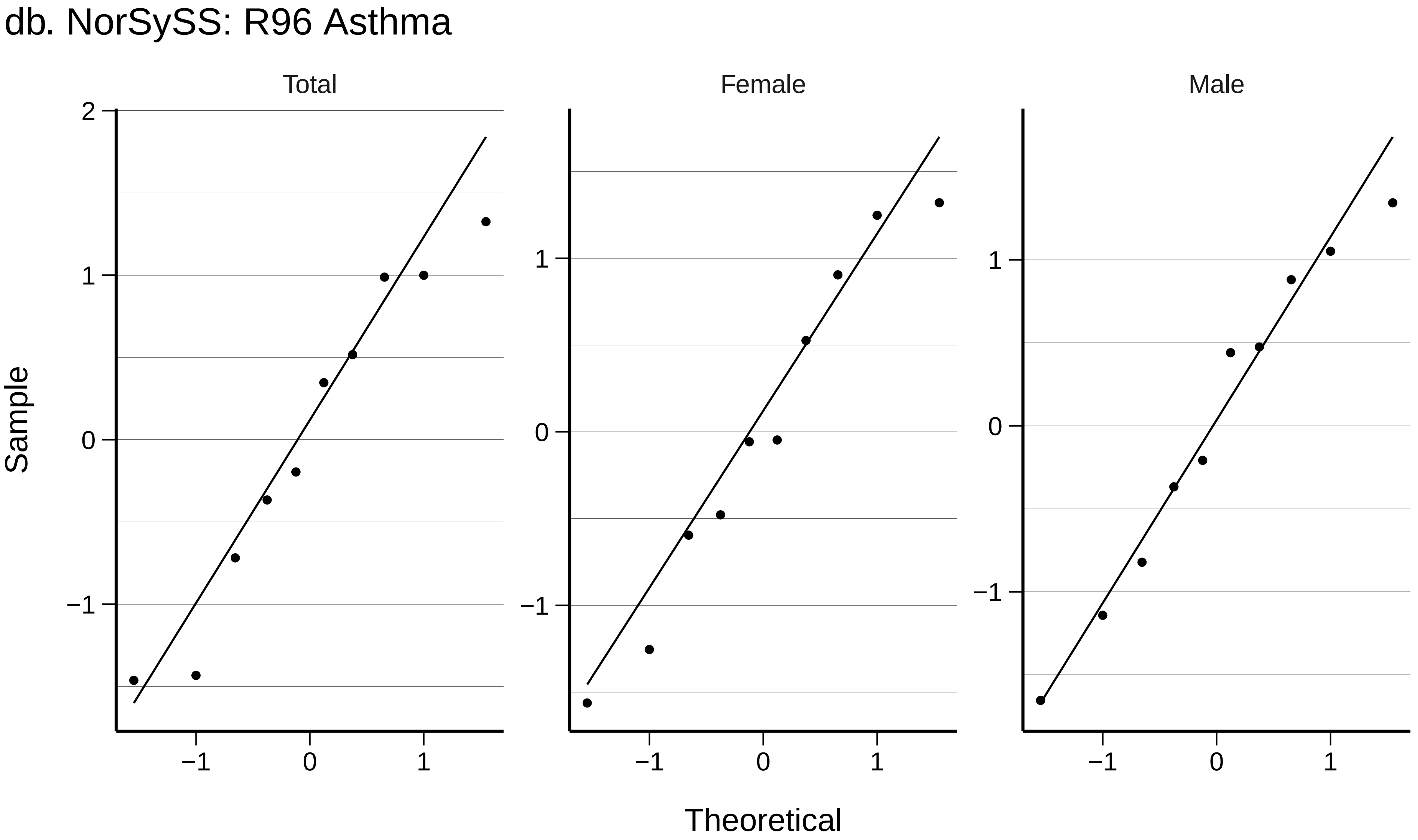
db. NorSySS: R96 Asthma
Total
Female
Male
Sample
Theoretical
2
1
0
−1
0
−1
0
1

dc. NorSySS: S29 Skin symptom/complaint other

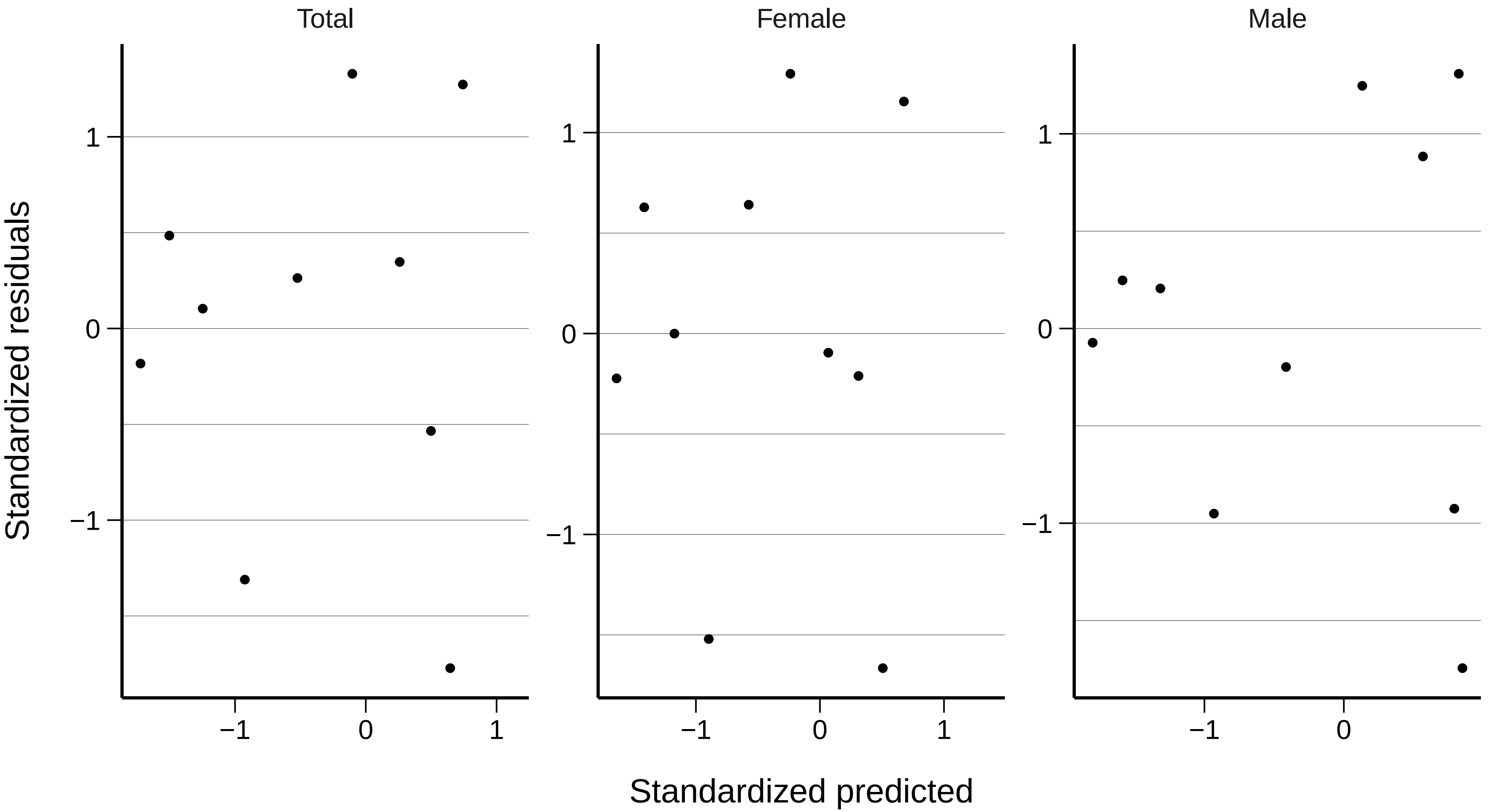

dd. NorSySS: S29 Skin symptom/complaint other

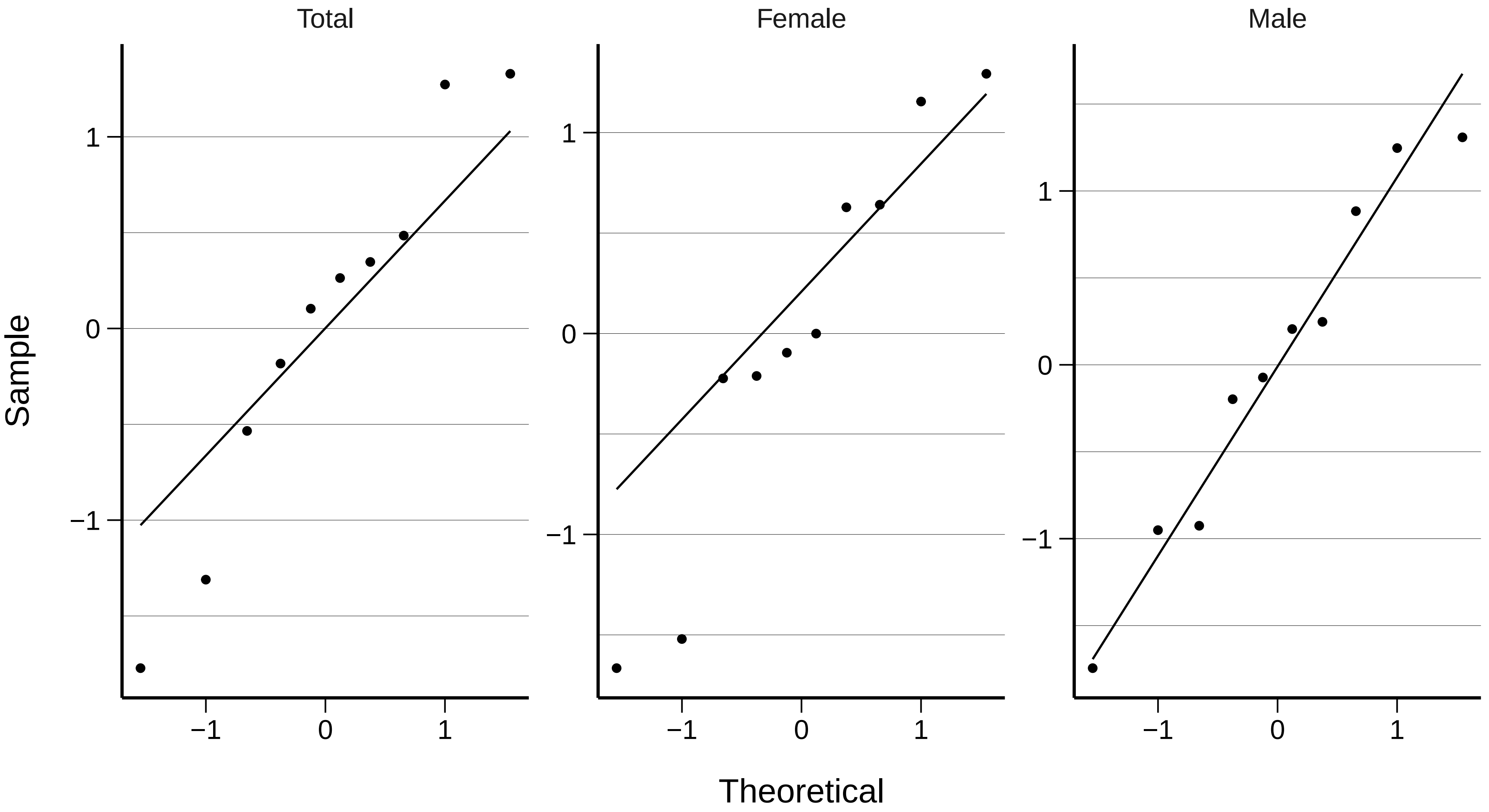

Supplement: Supplementary file 5 — Additional File 5. Residual diagnostic plots for the regressions in Additional File 4. [file 13690_2024_1411_MOESM5_ESM.pdf]
