# Supplementary material for: Aberrations in medically certified sick leave and primary healthcare consultations in Norway in 2023 compared to pre-COVID-19-pandemic trends
Source: Arch Public Health. 2024 Oct 22;82:187. doi: 10.1186/s13690-024-01411-4 (PMC11495095; doi:10.1186/s13690-024-01411-4)
Supplement: Supplementary file 6 — Additional File 6. Medically certified sick leave and community spread of COVID-19 (assuming 80% and 70% vaccine effectiveness) from 2020-Q1 to 2023-Q4. [file 13690_2024_1411_MOESM6_ESM.pdf]

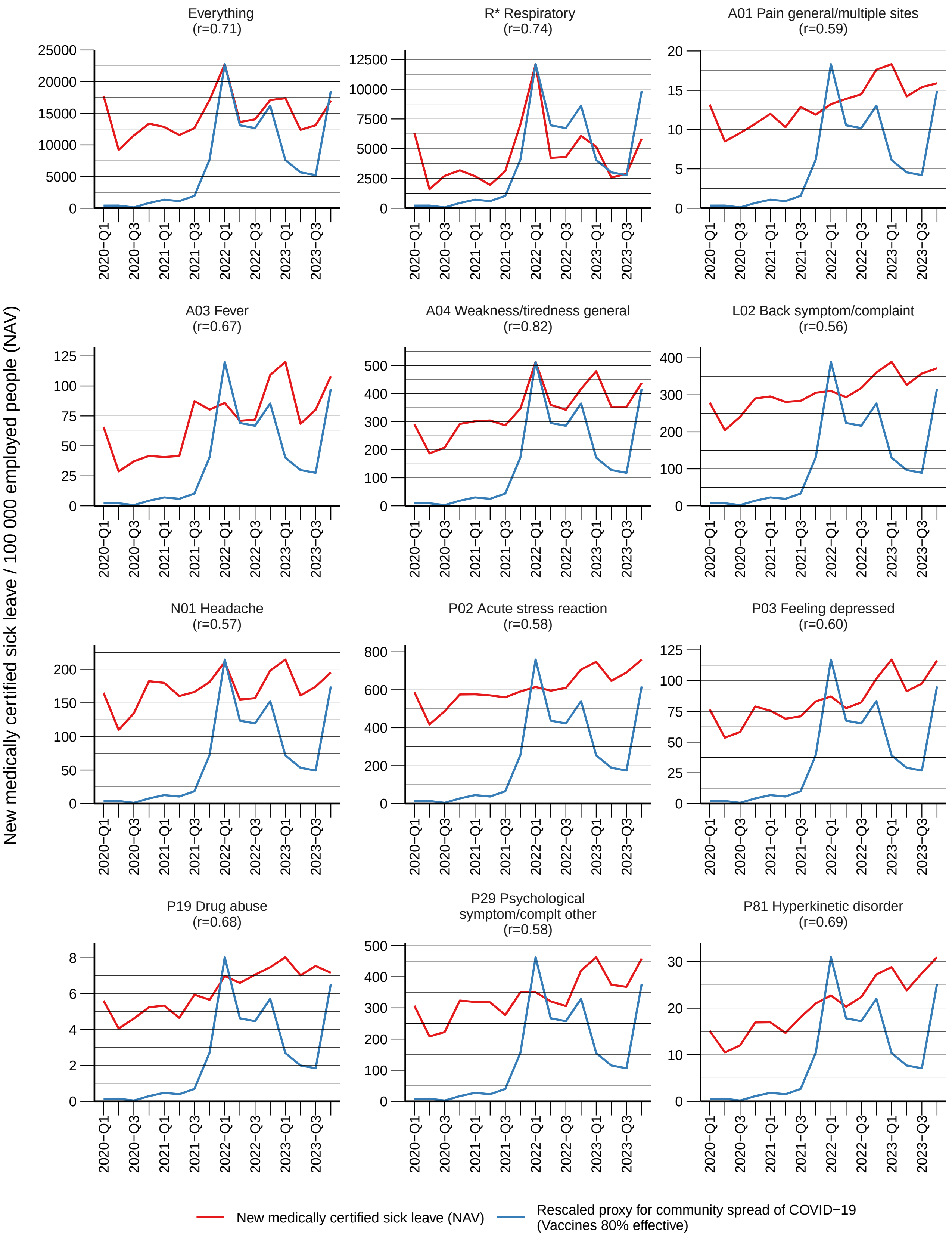

Everything
(r=0.71)
R* Respiratory
(r=0.74)
A01 Pain general/multiple sites
(r=0.59)
A03 Fever
(r=0.67)
A04 Weakness/tiredness general
(r=0.82)
L02 Back symptom/complaint
(r=0.56)
N01 Headache
(r=0.57)
P02 Acute stress reaction
(r=0.58)
P03 Feeling depressed
(r=0.60)
P19 Drug abuse
(r=0.68)
P29 Psychological
symptom/complt other
(r=0.58)
P81 Hyperkinetic disorder
(r=0.69)
2020−Q1
2020−Q3
2021−Q1
2021−Q3
2022−Q1
2022−Q3
2023−Q1
2023−Q3
New medically certified sick leave / 100 000 employed people (NAV)
New medically certified sick leave (NAV)
Rescaled proxy for community spread of COVID−19
(Vaccines 80% effective)

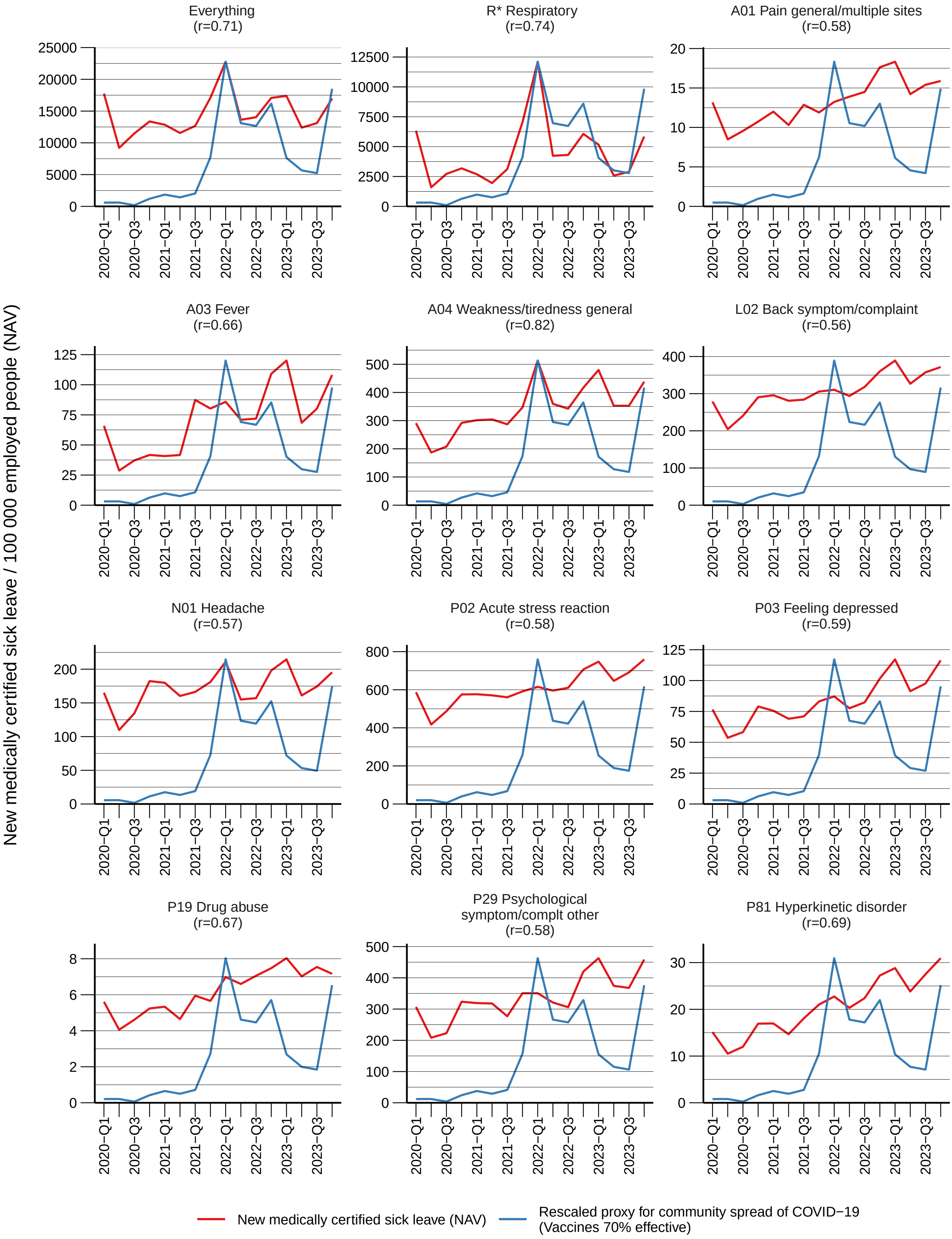

New medically certified sick leave / 100 000 employed people (NAV)
Everything (r=0.71)
R* Respiratory (r=0.74)
A01 Pain general/multiple sites (r=0.58)
A03 Fever (r=0.66)
A04 Weakness/tiredness general (r=0.82)
L02 Back symptom/complaint (r=0.56)
N01 Headache (r=0.57)
P02 Acute stress reaction (r=0.58)
P03 Feeling depressed (r=0.59)
P19 Drug abuse (r=0.67)
P29 Psychological symptom/complt other (r=0.58)
P81 Hyperkinetic disorder (r=0.69)
2020−Q1
2020−Q3
2021−Q1
2021−Q3
2022−Q1
2022−Q3
2023−Q1
2023−Q3
New medically certified sick leave (NAV)
Rescaled proxy for community spread of COVID−19 (Vaccines 70% effective)
